# Implications of divergence of methionine adenosyltransferase in archaea

**DOI:** 10.1002/2211-5463.13312

**Published:** 2021-11-05

**Authors:** Bhanu Pratap Singh Chouhan, Madhuri Gade, Desirae Martinez, Saacnicteh Toledo‐Patino, Paola Laurino

**Affiliations:** ^1^ Protein Engineering and Evolution Unit Okinawa Institute of Science and Technology Graduate University Onna Japan

**Keywords:** ancestral sequence reconstruction, catalytic interface, divergence, enzyme evolution, methionine adenosyltransferase

## Abstract

Methionine adenosyltransferase (MAT) catalyzes the biosynthesis of S‐adenosyl methionine from l‐methionine and ATP. MAT enzymes are ancient, believed to share a common ancestor, and are highly conserved in all three domains of life. However, the sequences of archaeal MATs show considerable divergence compared with their bacterial and eukaryotic counterparts. Furthermore, the structural significance and functional significance of this sequence divergence are not well understood. In the present study, we employed structural analysis and ancestral sequence reconstruction to investigate archaeal MAT divergence. We observed that the dimer interface containing the active site (which is usually well conserved) diverged considerably between the bacterial/eukaryotic MATs and archaeal MAT. A detailed investigation of the available structures supports the sequence analysis outcome: The protein domains and subdomains of bacterial and eukaryotic MAT are more similar than those of archaea. Finally, we resurrected archaeal MAT ancestors. Interestingly, archaeal MAT ancestors show substrate specificity, which is lost during evolution. This observation supports the hypothesis of a common MAT ancestor for the three domains of life. In conclusion, we have demonstrated that archaeal MAT is an ideal system for studying an enzyme family that evolved differently in one domain compared with others while maintaining the same catalytic activity.

## Graphical abstract

We investigated archaeal methionine adenosyltransferase (MAT) divergence by structural analysis and ancestral sequence reconstruction. The dimer interface of MAT containing the active site (usually well conserved) diverged considerably between the bacterial/eukaryotic MATs and archaeal MAT. The archaeal MAT ancestor showed substrate specificity, which was lost during evolution, supporting the hypothesis of a common MAT ancestor for the three domains of life.

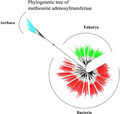

**Abbreviations**
ArchAnc: common archaeal ancestor
ASR: ancestral sequence reconstruction
AU: approximately unbiased
BactAnc: bacteria ancestors
CrenAnc: Crenarchaea
EukaAnc: eukarya ancestors
EuryAnc: Euryarchaea
LUCA: universal common ancestor
MAT: methionine adenosyltransferase
MjMAT: *Methanococcus jannaschii*
NTPs: nucleotide triphosphates
PDB: Protein Data Bank
pfMAT: *Pyrococcus furiosus*
SAM: *S*‐adenosyl methionine
SSNs: sequence similarity networks

Common descent is one of the fundamental aspects of Darwinian evolution. This theory emphasizes that modern‐day species share a common ancestry [1]. The same principle applies to enzymes: modern enzyme superfamilies across the three domains of life evolved from a set of enzymes that were already present in a last universal common ancestor (LUCA) that can be dated to over 3.5 billion years ago [2, 3]. Many efforts have been made to infer the minimal set of LUCA enzymes [3, 4]. The main hypothesis is that the evolutionary trajectory of enzymes (i.e., gene trees) is closely associated with and influenced by the evolution of their respective host organisms (i.e., species trees) [5]. However, in reality, gene evolution is much more complicated and disagreement between species trees and gene trees (noncongruence) can occur because of a wide range of factors such as gene duplication, lateral gene transfer [6, 7], and hybridization [8]. Within this framework, a few studies have reported an unusual distribution of some enzymes among the three domains of life, whereby the archaeal enzyme shows a divergence from the more closely related eukaryotic and bacterial orthologs [9, 10, 11, 12]. One example is the methionine adenosyltransferase (MAT) enzyme.

The unusual sequence similarity of MAT enzymes among the three domains is striking; the archaeal enzyme is almost equidistant from the bacterial and eukaryotic enzymes, with ˜ 20% sequence identity, while the bacterial and eukaryotic enzymes share greater than ˜ 60% identity [13].

Methionine adenosyltransferase is a ubiquitous enzyme, present across all three domains of life, that catalyzes the biosynthesis of *S*‐adenosyl methionine (SAM) from l‐methionine and ATP. SAM is a primary methyl donor in nature, and it is also considered to be an ancient cofactor [14]. SAM‐dependent enzymes are likely included in the LUCA [15]. Most of these ancient SAM‐dependent enzymes are radical methyltransferases [15, 16, 17, 18]. Nowadays, SAM is involved in four important functions (Scheme S1) [19]: (a) methylation of fundamental biomolecules (proteins, DNA, RNA) [20, 21]; (b) polyamine synthesis [22]; (c) glutathione biosynthesis reactions [23]; and (d) radical chemical reactions [24]. Moreover, methylated molecules or macromolecules by SAM play an important role in cellular processes such as epigenetics, signal transduction, protein function, and genetics [25]. The importance of these biological processes makes the SAM‐synthesizing enzyme, namely MAT, an important and essential enzyme for cell growth and survival [26].

The structure of the MAT enzyme is unique and classified by the SCOP database in α/β class [27]. Each monomer of MAT is composed of a βαββαβ secondary structure [28]. No sequence conservation is observed among the repeats. Like many other enzymes in nature, MAT is active as an oligomer (either as a homo‐oligomer or as a hetero‐oligomer; Fig. 1) [29]. The oligomeric state provides a clear advantage over the monomeric state in terms of fitness, stability, and functions [30, 31]. In dimeric MAT, helixes are exposed to the surface and strands form a hydrophobic interface [32]. The MAT homodimer consists of monomeric subunits (α‐subunits) paired together along a large flat hydrophobic interface (henceforth referred to as large interface; Fig. 1) wherein the active sites are enclosed [13]. A homotetramer exists as a dimer of homodimers; therefore, it has been suggested that the preferred functional quaternary structure is an oligomer (possibly an obligate dimer) [33].

**Fig. 1:**
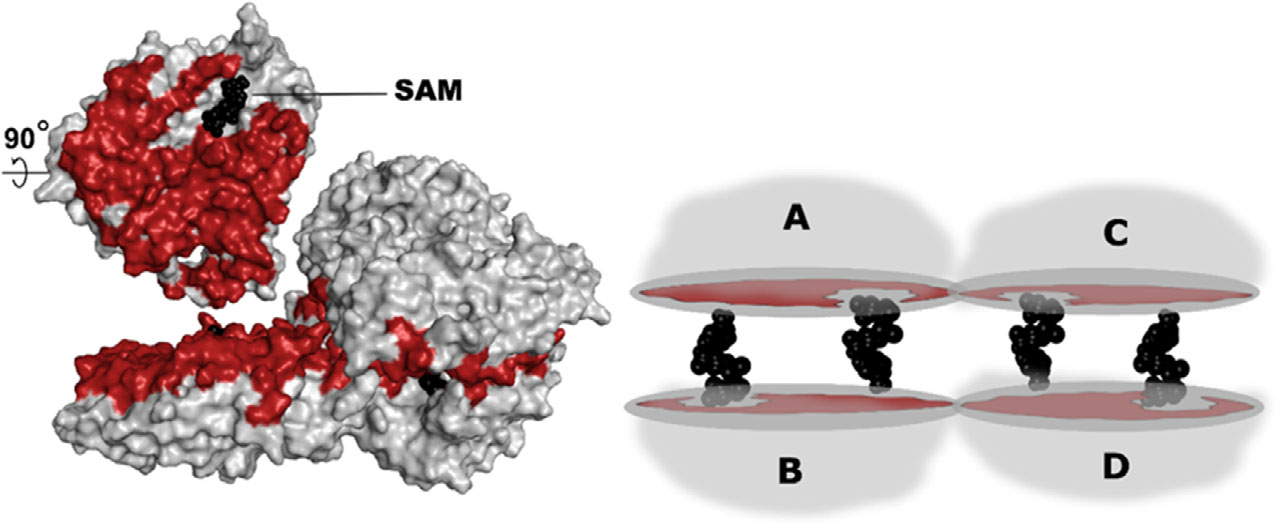
MAT homotetramer configuration. Schematic representation of the MAT homotetramer (right) and surface/cartoon view (left)—the large interface region (between chains A and B) is highlighted in red, while SAM is highlighted in black. The large interface formed by chains A and B (also chains C and D), which have an area of ˜ 1900–2000 Å^2^, consists of ˜ 70–100 residues. Representative structures have been utilized here, while the archaeal pfMAT structure (PDB ID: 6S83 from *Pyrococcus furiosus*) utilizes 83 residues to give rise to the large interface. These numbers are 90 residues in case of bacterial eMAT (*Escherichia* *coli*, PDB ID: 1RG9) and 77 in the case of eukarya hMAT2A (*Homo* *sapiens*, PDB ID: 4NDN).

It has been suggested that all extant MAT enzymes share a common ancestry [33] despite expressing a highly divergent form in archaea, as mentioned above. This further indicates that the adaptation of enzymes during evolution can lead to the accumulation of mutations at the surface or interface regions, especially near the active sites. This phenomenon can allow for the divergence and acceptance of different substrates or functions [34, 35, 36, 37, 38, 39]. Despite this contrast, it has also been established that archaeal MAT can still perform the same catalytic reaction as their orthologous counterparts (from bacteria and eukarya); therefore, the structural and functional implications of this divergence are not well understood. These observations prompted us to take a closer look at the evolutionary trends adapted by the active site residues and the large interface region of MAT. Therefore, we probed the putative evolutionary trajectory of archaeal MAT more systematically by conducting investigations at various levels, including sequence studies and structural comparisons, assessment of physiochemical properties, and ancestral sequence reconstruction (ASR). We further elucidated this potential evolutionary trajectory by resurrecting the ancestral archaeal MAT sequences and characterizing their biochemical properties. In conclusion, we showed that the archaeal MAT can display high degrees of divergence at the sequence/structural level and yet perform the same catalytic reactions as their orthologous counterparts.

## Results and Discussion

### Probing the MAT sequence space

**Fig. 2:**
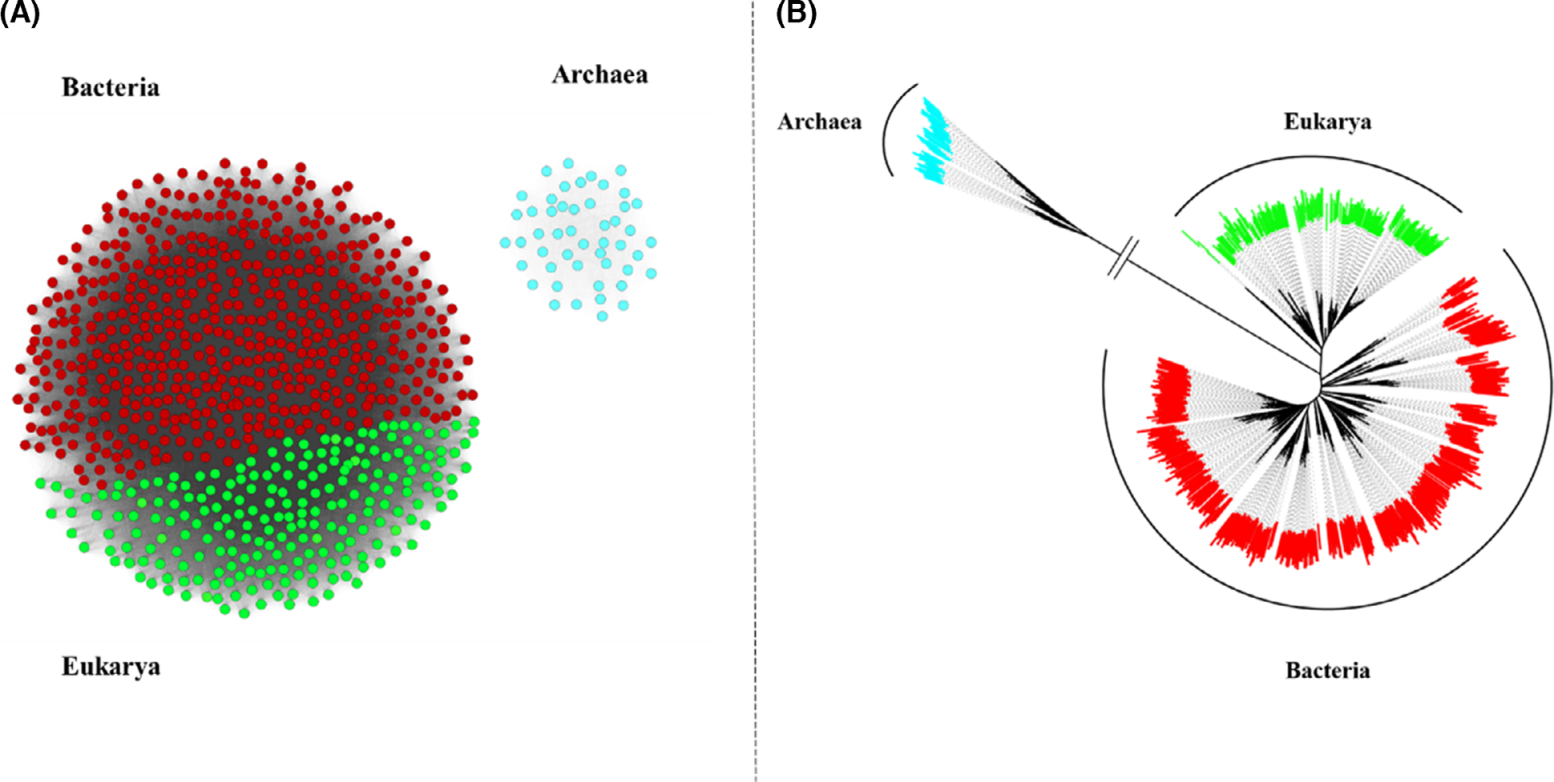
Schematic representation of observed sequence space in MAT. (A) SSN of MATs from the three domains—the sequences from eukarya (green) and bacteria (red) form a distinct cluster, while the sequences from archaea (cyan) form a separate cluster. This SSN was created with the EFI enzyme similarity server and visualized using the Cytoscape program. Clustering pattern within the network at different levels of sequence similarity (similarity %) can be observed in the supplementary information (Fig. S1). (B) Unrooted ML tree for MATs from the three domains of life. This unrooted tree was built by applying the ML method with IQ‐TREE, followed by visualization using FigTree viewer program and iTOL server. The archaeal MATs branch off, thereby highlighting their divergence, an observation that was further substantiated through SSN. The archaeal MATs are represented in cyan, eukarya in green, and bacteria in red.

In the present study, we conducted extensive searches to systematically probe the MAT sequence space across the three domains. The sequences were collected by combining the outcomes of different databases (nr db, eggnog, orthodb, etc.). We built sequence similarity networks (SSNs) upon compiling the first input dataset (˜800 sequences), which allowed us to check the MAT sequence space distribution across the three domains (Fig. 2A). During this analysis, we considered that the archaeal domain has not been sequenced as extensively as the other two domains of life [40]. Because phylogenetic trees may not be an optimum tool to highlight the overall sequence distribution pattern, we utilized two approaches, EFI and CLANS [41], to visualize the sequence distribution pattern (Fig. S1). Despite the variation in the percentage sequence identity clustering ranging from 100% to 40% ID, the overall topology remained consistent with two major clusters representing: (a) ‘bacteria + eukarya’ and (b) archaea. The SSN outcomes reflected the sequence identities as observed in nature, with archaea found to be nearly equidistant from eukarya and bacteria, at ˜ 20% sequence identity. The SSN method provided an overall view of the topology for the dataset of interest and underlined the uniqueness of the archaeal domain.

### Inferring the ancestral MAT sequences

**Fig. 3:**
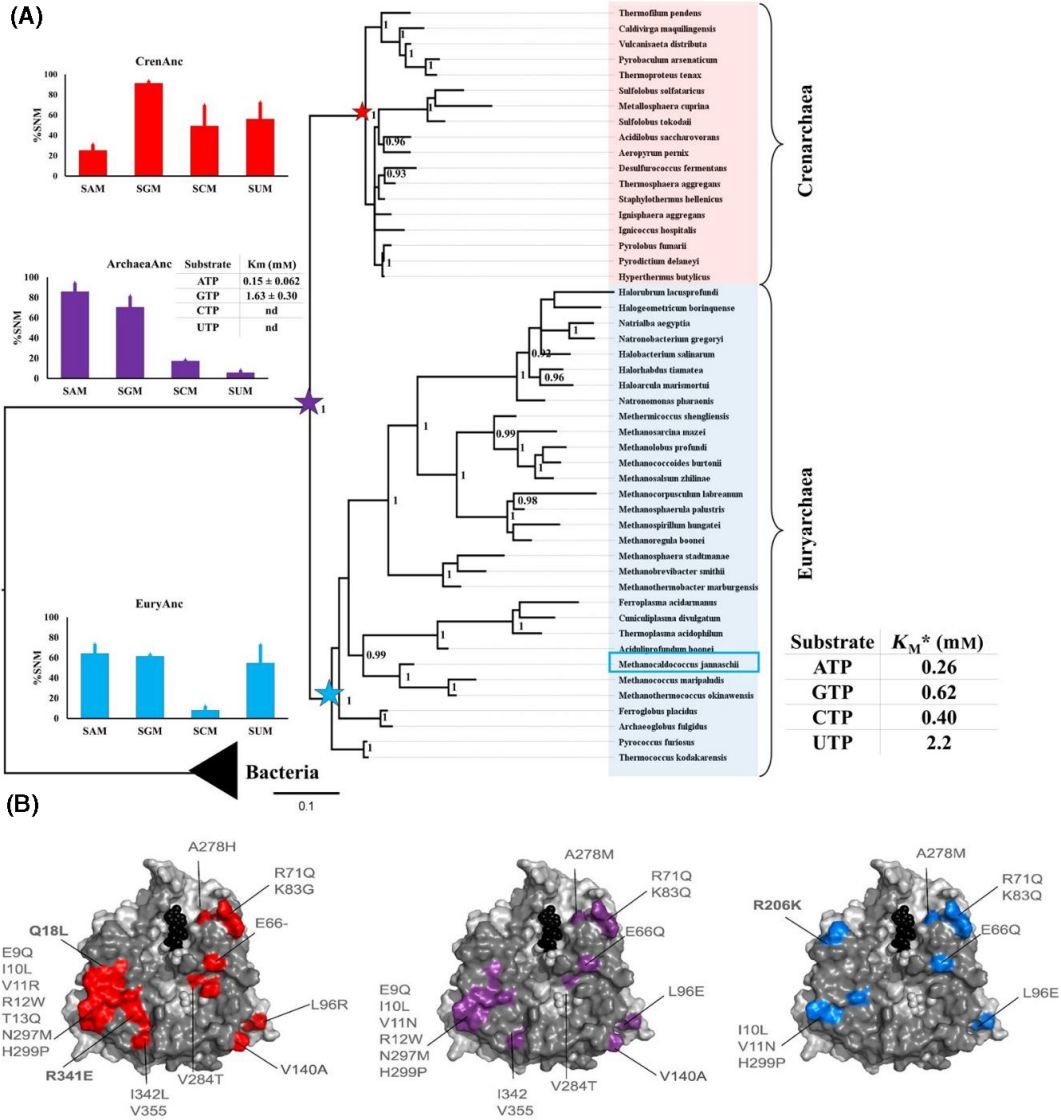
Phylogenetic tree of archaeal MAT with bacteria domain as outgroup and activity assays of the inferred ancestors. (A) The maximum‐likelihood phylogenetic tree topology of archaeal MAT with bifurcation into two major clades, that is, Crenarchaea and EuryAnc, and with bacteria as an outgroup. This topology is further supported by another phylogenetic tree based on the 16s rRNA sequences from the corresponding species. Here, *we highlight the activity of *MjMAT* as representative of domain archaea, with the data as reported previously [48]. Ancestral sequences were inferred for the ancestor of Crenarchaea MAT (CrenArc in red star), EuryAnc MAT (EuryArc in blue star), and the common ancestor for the archaeal MAT (ArcheaArc in violet star). Bayesian posterior probability values > 90% are shown. One time point assay for ancestral sequence activities was performed with corresponding enzyme (20 µm) using NTPs (5 mm) and methionine (10 mm) in HEPES (100 mm), KCl (50 mm), and MgCl_2_(10 mm), pH 8, at 37 °C for hMAT1A and 55 °C for archaea, 1 h. For kinetics analysis, ArcheaArc MAT (0.5 µm), ATP, and GTP (0.1–2 mm) and methionine (10 mm) using the same above‐mentioned buffer and conditions were used. The experiments were performed in duplicates. The error bars represent SD. Tables show the extrapolated *K*_M_ for ArcheaArc. The production of SAM and SGM was analyzed by UPLC, and data were fitted to the Michaelis–Menten equation using graphpad software 7.02. (B) Mutations found by the ancestral reconstruction analysis are highlighted on the available structure of pfMAT (PDB: 6S83). The structures are represented as gray surface and show the large interface. CrenAnc MAT interface mutations are highlighted in red, EuryAnc in blue, and ArchaeaAnc in violet.

We reconstructed the phylogenetic relationships by utilizing MAT sequences from the three domains of life (Fig. 2B). In the case of archaea, two major phyla [Crenarchaea and Euryarchaea (EuryAnc)] were tested for the overall tree topology based on the MAT sequences (gene tree), as well as corresponding 16s rRNA sequences (species tree) from the SILVA database (not shown here). Herein, the two topologies clearly indicated that the tree bifurcates into two distinct clades for Crenarchaea and EuryAnc; that is, the major phyla are well classified into their respective monophyletic groups. Subsequently, we prepared another set of trees by utilizing bacterial MAT sequences as an outgroup to extract the putative common archaeal ancestor ArchAnc (Fig. 3, violet star). We also extracted the ancestral sequences of Crenarchaea (CrenAnc) and EuryAnc MAT at the designated nodes highlighted by using a red and blue star, respectively (Fig. 3).

The sequence similarities of the ancestral sequences (including interface residues and active site residue comparisons) are detailed in the other sections.

**Fig. 4:**
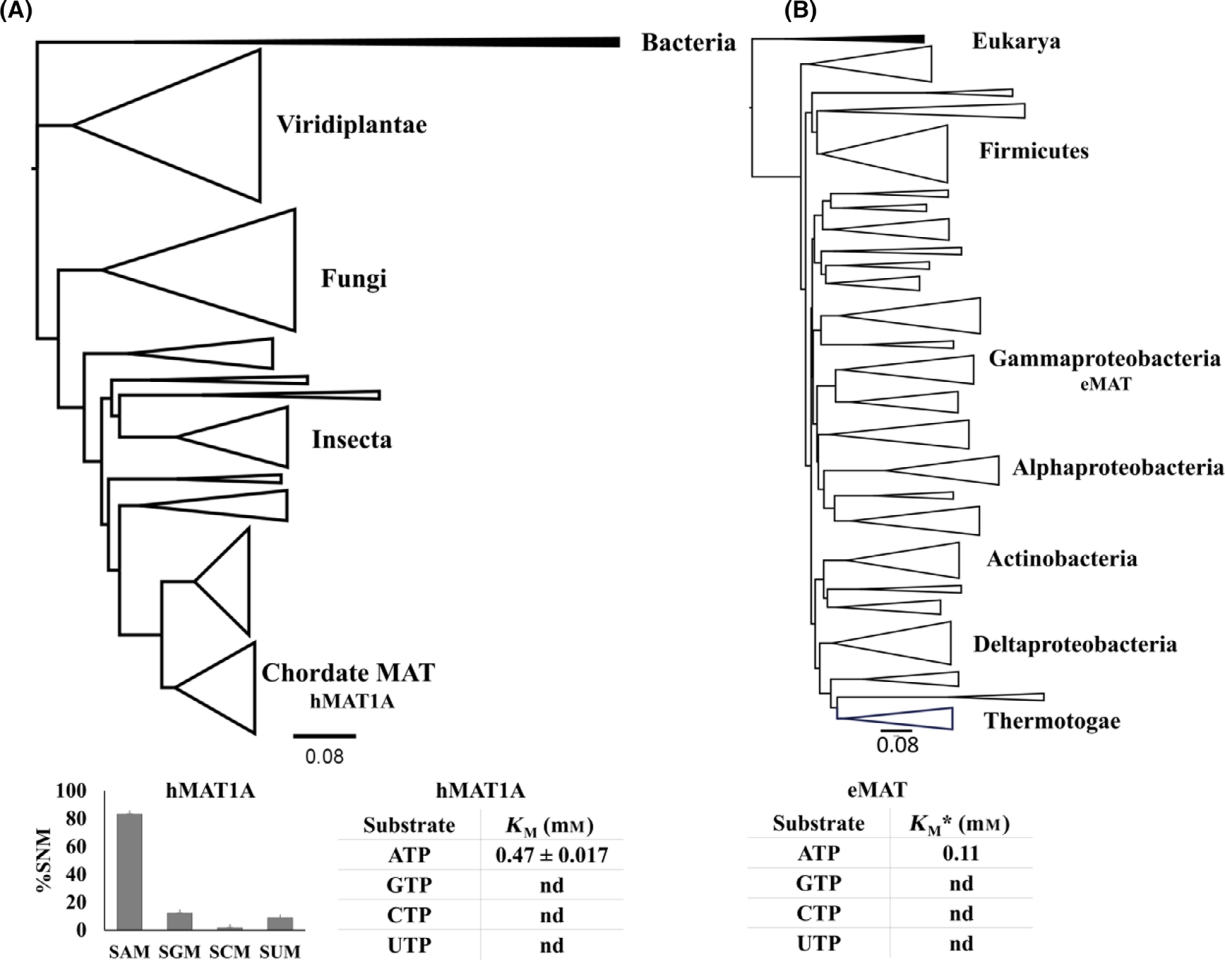
Phylogenetic trees based on the MAT sequences for eukarya and bacteria domains. (A) ML phylogenetic tree of MAT sequences from domain eukarya, with bacterial MAT sequences as an outgroup (highlighted in black). The major clades have been annotated according to their distribution in the tree topology. We reported the activity of hMAT1A as representative of domain eukarya. One time point assay for hMAT1A activities was performed using 20 µm of hMAT1A with NTPs (5 mm) and methionine (10 mm) in HEPES (100 mm), KCl (50 mm), and MgCl_2_ (10 mm), pH 8, at 37 °C, 1 h. For kinetics analysis, hMAT1A (0.5 µm), ATP (0.1–2 mm), and methionine (10 mm) using the same above‐mentioned buffer and conditions were used. The experiments were performed in duplicates. The error bars represent SD. Tables showing the extrapolated *K*_M_ for hMAT1A. (B) ML phylogenetic tree of MAT sequences from bacteria domain, with eukarya MAT sequences utilized as an outgroup (highlighted in black). *We also highlight the *K*_M_ of ATP from eMAT as a representative of bacteria domain, as reported previously [48]. These results suggest that both the enzymes, hMAT1A and eMAT, are not promiscuous, as they display specificity toward ATP. The trees were built using the ML method with the IQ‐TREE program. Tree topologies were visualized using FigTree program.

For bacterial and eukarya MAT sequences, we prepared a separate phylogenetic tree (based only on MAT sequences). MAT sequences from these two domains constituted two distinct clusters (supported by principal component analysis clustering—data not shown), as observed in the tree topology as well (Fig. 4), which was then utilized to infer the common MAT ancestors for eukarya (EukaAnc) and bacteria (BactAnc).

### Extant MAT sequences vs ancestors: active sites and evolutionary rates

**Fig. 5:**
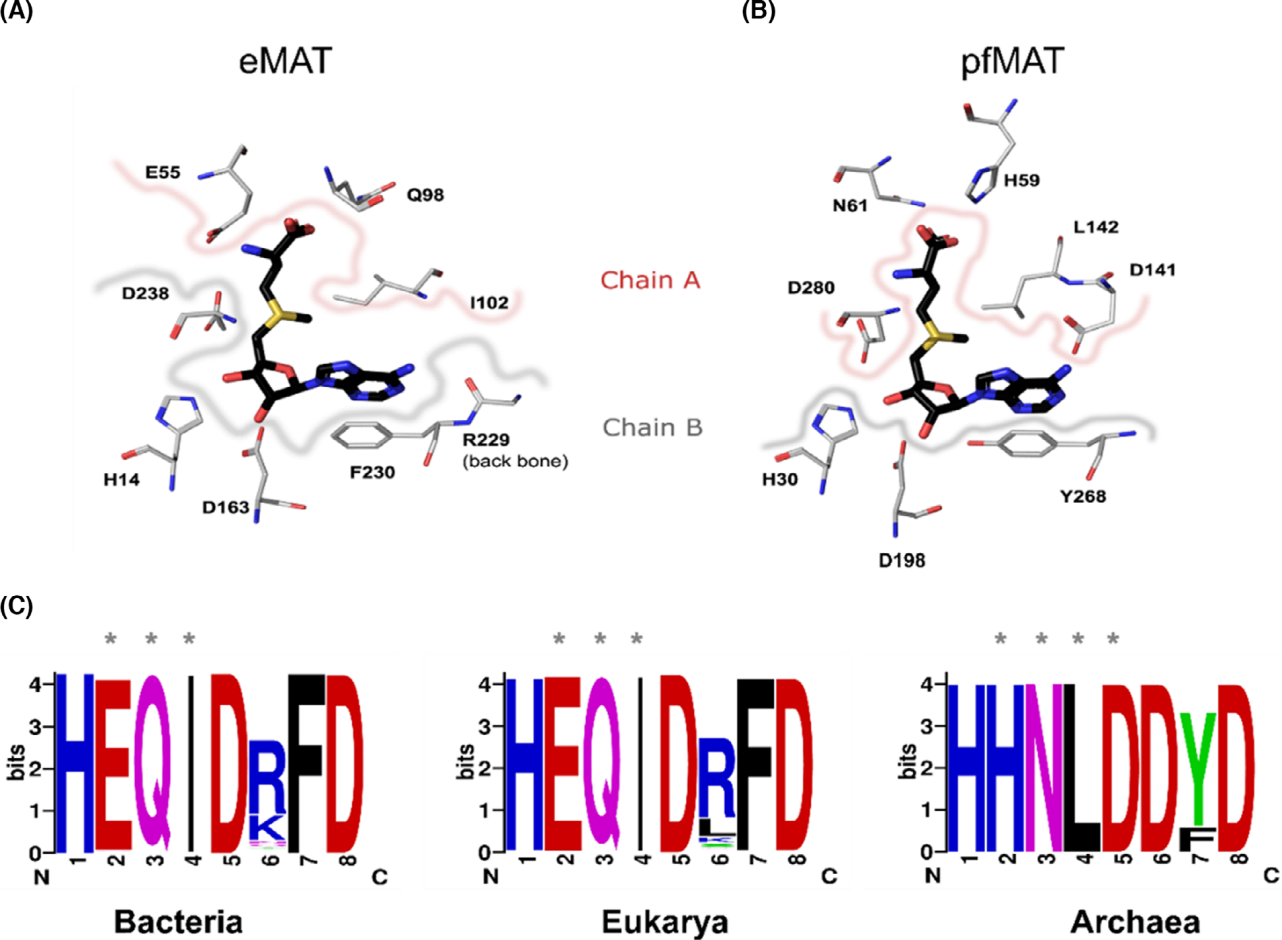
Comparison of the active site residues in the different domains. (A) Active site residues involved in the interaction with SAM. SAM is highlighted in black, while the active site residues from eMAT (PDB: 1RG9) have been highlighted in metal gray. The residues derived from chain A (E55, Q98, I102) have been demarcated with a pinkish border, and residues from chain B (H14, D163, R229, F230, D238) have been demarcated with a grayish border. (B) Equivalent active site residues from pfMAT [33] (PDB: 6S83) that interact with SAM have also been highlighted from chain A (H59, N61, D141, L142, D280) and chain B (H30, D198, Y268) using the same color codes. (C) The active site residues identified based on structural alignment, from all three domains of life, have been highlighted as WebLogos (https://weblogo.berkeley.edu/logo.cgi). Residues derived from the second monomeric subunit have been marked using ‘*’. WebLogo was generated using alignment of 526 sequences from bacteria (Appendix S1), 273 sequences from eukarya (Appendix S2), and 49 sequences from archaea (Appendix S3). Details of sequence search are in the Methods section.

We probed the evolutionary trend(s) for amino acid sites in MAT enzymes across the three domains of life. These trends were visualized by mapping the interface sites, as well as active sites on a representative X‐ray structure (Fig. 5, Fig. S2). Additionally, we probed the level of conservation for active site residues in extant and ancestral sequences.

#### Active site residues

In the case of archaea, we observed that the MAT active site residues were highly conserved among the two major branches, that is, Crenarchaea and EuryAnc (residue numbers correspond to *Pyrococcus furiosus* (pfMAT) structure: Protein Data Bank (PDB): 6S83, [33]). These include the following residues involved in the interaction with SAM from chain A: H59, N61, D141, L142, and D280 and chain B: H30, D168, and Y268. Furthermore, these active site residues were completely conserved in the putative archaeal ancestor. A similar trend was observed in the cases of both eukarya and bacteria. This provides us with a key insight that despite observing differences in the active site residues, MAT enzymes from the three domains can still essentially retain the same catalytic reaction (Fig. 5).

#### Evolutionary rates

To determine whether part of the protein evolved at a different rate compared with the rest of the structure, we determined the evolutionary rate distribution of all residues in the MAT structure (Fig. S2). Interestingly, most sites located along the large interface tend to evolve slowly (highlighted in blue), while the opposite trend is observed as we move further away from the large interface. For instance, some of the residues located along the surface α‐helices and loop regions tend to experience higher evolutionary rates. This pattern can be seen in MAT enzymes from all three domains of life, thereby indicating that conservation at a large interface is probably critical for enzyme function despite the domain that the enzyme belongs to.

### Large interface: charge and hydrophobicity distribution

In case of MAT enzymes, the constituent monomers pair up in an inverted configuration by exposing the α‐helices toward the surface, while the β‐strands interact to form a hydrophobic large interface that harbors the active site residues, thereby making this homodimer the obligate functional unit [32]. Herein, we noticed that divergence is not only limited to active residues, or the residues located close to the substrate, but most of the interdimer interface (large interface) residues also diverge across the three domains of life. For instance, in cases of bacteria (eMAT, PDB: 1RG9 [42]) and eukarya (hMAT1A, PDB: 6SW5 [43]) 38 out of the 51 aligned residues were identical. In contrast, archaea (pfMAT, PDB: 6S83) shares only 12 identical residues with bacteria (eMAT) and eukarya (hMAT1A; Figs S3 and S4). This further prompted us to perform a systematic analysis of the large interface region across the three domains of life, and thus, we assessed the charge and hydrophobicity distribution across the large interface and compared it with the ancestral sequences to gain more insights into the evolutionary patterns involved. We studied 17 experimentally solved MAT structures from PDB to probe the physiochemical properties, by creating two datasets: (a) for identification of 51 structurally aligned positions along the large interface (which includes up to five noninterface residues as well) and (b) for identification of 24 structurally aligned ‘interface‐only’ residue positions.

#### Large interface region (extant)

**Fig. 6:**
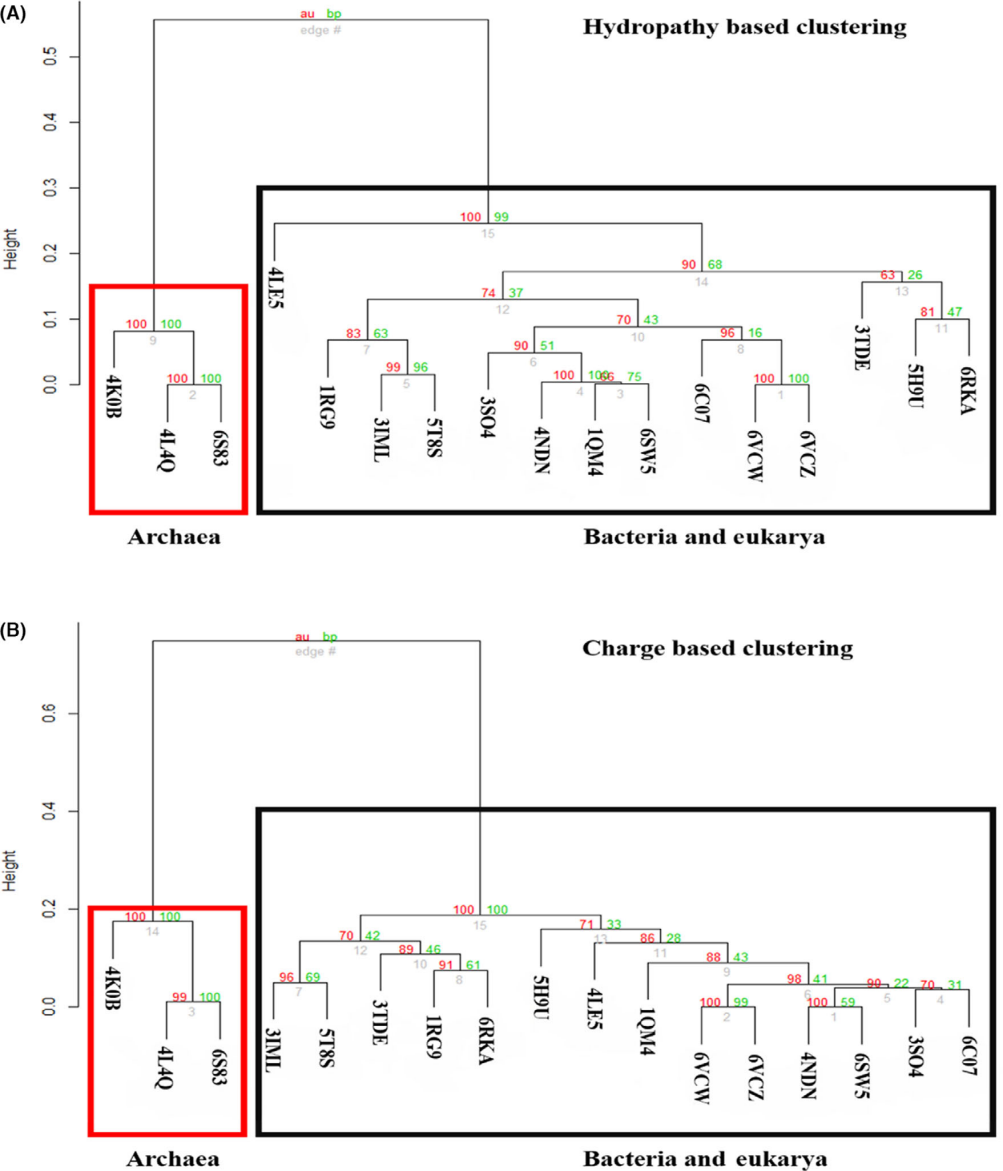
Clustering analysis of hydropathy (A) and charge (B) of representative structures from the three domains of life. Clustering analysis of the physiochemical properties (hydropathy and charge) of 51 structurally aligned large interface residues from chain A of 17 MAT structures (PDB list in the supplementary information). Based on the 51 aligned positions, we calculated the per‐site hydropathy using the Kyte–Doolittle scale from the ProtScale server. In addition, we calculated the per‐site charges for the 17 MAT structures using the EMBOSS charge server. Subsequently, we conducted a clustering analysis using the pvclust package in r. This further provides a statistical score in terms of AU (AU scores depicted in red), *P*‐value, and BP (bootstrap probability scores depicted in green) value, for comparison of the clusters, which reveals that the hydropathy (A) and charge distribution (B) clustered together for bacterial and eukarya MAT structures, with archaeal MAT structures clustered separately. In both cases, the archaeal cluster was provided with high support values, in terms of both the AU and BP parameters, and 100% support for a distinct archaeal cluster with respect to the two physiochemical properties.

Based on the 51 structurally aligned positions, even at the sequence level, the large interfaces of bacterial and eukarya MATs have a striking sequence identity of ˜ 50–70% (Figs S4 and S5). However, the archaeal interface sequences are nearly equidistant from the other two domains at ˜ 15–30% sequence identity, despite having a comparable size in terms of area (˜ 1660–2970 Å^2^). The hierarchical clustering plots (based on the pvclust package in r; Fig. 6 [44]) show that the sequence identities further dictate the physiochemical properties as well. For instance, a look at both the *P*‐values provided by pvclust for comparison of the clusters, that is, approximately unbiased (AU) *P*‐value and bp (bootstrap probability) value, reveals that the hydrophobicity and charge distribution are clustered together for bacterial and eukarya MAT structures, with the archaeal MAT structures clustering off separately. An additional analysis was also conducted for the second dataset, with an alignment of 24 ‘interface‐only’ residues (Fig. S6), and in this case too, we observed similar results, with the archaeal MAT structures clustering off separately.

#### Ancestral vs extant (large interface)

In this study, we compared the large interface residues extracted from the ancestral sequences against their extant counterparts. A comparison of the large interface residues from the bacterial MAT ancestor with the corresponding residues from eMAT highlighted a difference of 13 out of 51 residues (Fig. S4). Likewise, we also observed similar changes in eukarya: hMAT1A vs eukarya MAT ancestor, with seven residue changes (out of 51 residues); and archaea: pfMAT vs archaeal MAT ancestor, with six changes (out of 51 residues).

In conclusion, these observations provide crucial insight that sequence identities further influence the distribution of corresponding physiochemical properties. Therefore, the hydrophobicity and charge distribution, as described in the hierarchical clustering (based on pvclust), are important in understanding the evolutionary trends adapted by the large interface. However, we observed that the active sites remained conserved to some extent across MAT enzymes.

### Structural comparison supports sequence divergence

Structural comparisons of MAT full domains from archaea, bacteria, and eukarya were performed to identify their common and unique structural features. We noticed that at a structural level, bacteria and eukarya show a higher degree of structural similarity compared with archaea (Fig. S7). In addition, an N‐terminal extension in archaea segment‐swaps into the C‐terminal subdomain in contrast to bacteria and eukarya, which lacks the N‐terminal beta‐strand. In return, bacterial and eukaryotic MATs display an extra C‐terminal helix. These unique features may explain why archaeal MAT is not dissected into subdomains by either the SCOP or ECOD domain criteria. Topology connections according to PDBsum [45] are shown in Fig. S7.

We further extended our structural comparisons to the subdomain level. In this analysis, constituent domains (according to ECOD [46, 47]) from bacterial and eukaryotic MATs were used as templates to manually dissect archaeal subdomains (Fig. S8). Constituent structural domains were then compared all‐on‐all within (Fig. S8), as well as across, archaea, bacteria, and eukarya (Fig. S9). Subdomain superposition showed that despite their identical topology, subdomains I to III within archaea, bacteria, and eukarya differ considerably in their secondary structure element orientation in space. In contrast, the subdomains across archaea, bacteria, and eukarya are much more similar. In this study, bacteria and eukarya displayed a higher degree of structural agreement within themselves than with archaea.

The structural comparison suggests that MAT emerged partially by duplications of subdomains within the same domain of life or those putatively shared by eukarya and bacteria, but not by archaeal enzymes. This observation supports the outcome of the analysis at the sequence level.

### Change in substrate specificity along the archaeal phylogenetic tree

It has been previously reported that the archaeal *Methanococcus* *jannaschii* (*MjMAT*) is promiscuous toward different nucleotides; that is, it is able to accept different nucleotide triphosphates (NTPs), while the bacterial ortholog eMAT is specific for the double ring adenine nucleotide base [13, 48]. This further prompted us to study the specificity of the purine bases, ATP, and GTP within the archaeal phylogenetic tree and to determine whether this information could provide us with more clues toward the origin and evolution of MAT (Fig. 3). We expressed and tested the activity of the ancestral archaeal MAT sequences and compared them against the representative extant MAT sequences from the three domains of life (Figs 3 and 4). Data on the specificity of the representative bacterial and archaeal extant MAT have already been reported in literature [13, 48]. We chose hMAT1A as a representative of eukarya. Since our purpose was to analyze the change in specificity within archaeal domain, we expressed and tested the activity of the ancestral MAT sequences for EuryAnc, CrenAnc, and the common archaeal ancestor (ArchaeaAnc) (Figs S10–S13, Figs S14–S17). First, we performed one end‐point assay with EuryAnc, CrenAnc ArchaeaAnc, and hMAT1A, where we observed CrenAnc displays similar promiscuities toward all the bases as the extant MAT enzyme (Fig. 3), while EuryAnc was losing promiscuity toward cytidine triphosphate (CTP). Interestingly, ArchaeaAnc acquires specificity for ATP and GTP (*K*_M_ ATP ArchaeaAnc 0.15 mm, *K*_M_ GTP ArcheaArc 1.63 mm; Fig. 3A and Figs S14 and S18). In contrast, eukarya hMAT1A shows specificity for the ATP substrate (*K*_M_ ATP hMAT1A 0.47 mm (Fig. 4A and Figs S17 and S18). Unfortunately, the bacterial and eukaryotic ancestral MAT sequences could not be expressed in the soluble and insoluble fractions. Therefore, this could not be further studied. Furthermore, in the archaeal phylogenetic tree, we detected a change in specificity, starting from a more specific ancestor toward a promiscuous extant enzyme. We observed that the specificity for ATP vs GTP goes from a 10‐fold difference in the ancestor ArchaeaAnc to no difference in *MjMAT*, which indicates that the ancestor was specific toward purine bases, but through the course of evolution, modern‐day archaeal MAT became promiscuous toward different bases. Therefore, these data hint at the hypothesis that all the modern‐day MAT enzymes from the three domains shared a common ancestor specific for the nucleobase and that archaeal MATs diverged in sequence, structure, and substrate specificity.

### Additional factors that influence the evolutionary trajectory of MAT

In this study, we have shown that various factors could have contributed to the unusual evolutionary trajectory of the MAT enzyme across the three domains of life. However, some additional aspects could have played a significant role in shaping the evolutionary trajectory of the MAT enzyme in archaea, such as codon usage bias, tRNA bias, or an alternative/distinct SAM metabolism. It has been shown that in general, the archaeal genomes display a higher GC‐rich tendency in contrast to bacterial and eukaryotic genomes. This tendency has a direct influence on codon usage. Codon usage bias also has a direct association with tRNA abundance for protein translation optimization [49, 50]. This includes avoiding slowly translated codons and utilizing codons with the most cognate abundant tRNAs from the genome. Additionally, in highly expressed genes, favored codons are easily recognized by abundant tRNA molecules [51, 52, 53]. This bias could be further augmented by the time and speed of gene expression, as it could also play a critical role in the presence of more abundant and less diverse tRNAs as well [54]. Another possible factor that might have influenced the divergence of archaeal MAT is the altered SAM metabolism. Although we acknowledge the presence of a different methyl donor in archaea, tetrahydromethanopterin [54], no clear connection to the SAM metabolism was found to support the hypothesis of the alternative MAT evolutionary trajectory. Additionally, since their divergence from LUCA, the selection and adaptive forces operating on bacterial/eukaryotic and archaeal clades may have differed substantially due to a variety of factors, including different environmental and metabolic constraints, differences in the regulation of activity, changes in the oligomerization state, and intracellular turnover. It is well known that bacterial MAT is strictly dependent on GroEL [55] and this dependency might play a role as an adaptive force; however, no information is available for the archaeal MAT. Therefore, we anticipate that these additional factors could also play a key role in guiding the evolution of highly diverged archaeal MAT, as well as other enzymes.

In brief, we expected the large interface region from MAT to be well conserved across the three domains of life, primarily because of the following reasons: (a) It accommodates the active site residues; (b) facilitates the same catalytic reaction, and (c) is composed of a large flat hydrophobic region composed of β‐sheets, which are known to evolve more slowly than the α‐helical regions localized at the surface in the case of MAT [56, 57]. However, our results clearly show that the active site interface is surprisingly divergent in the case of archaeal MAT, in contrast to bacterial/eukaryotic MAT. The present work highlights how these unique MAT features make this enzyme family an ideal system for studying one exceptional case where the canonical domain relationships are disobeyed.

## Methods

### Database

Model data are available in the PMDB database under the accession numbers PM0083365, PM0083366, and PM0083367

### Materials

ATP, GTP, CTP, uridine triphosphate (UTP), methionine, SAM, HEPES, MgCl2, KCl, IPTG, Tris/HCl, Na_2_HPO_4_, NaH_2_PO_4_, potassium phosphate, NaCl, imidazole, β‐mercaptoethanol, DTT, kanamycin, glycerol, NaOH, HCl, ammonium acetate, Bacto agar, Bacto tryptone, and Bacto yeast extract all other chemicals, and HPLC grade solvents were purchased from commercial sources and used as supplied unless otherwise mentioned. Page Ruler prestained protein ladder, 10–180 kDa was purchased from Thermo Fisher Scientific (Waltham, MA, USA). BL21 (DE3) competent cells were bought from New England Biolabs (NEB, Ipswich, MA, USA). Benzonase and cOmplete His‐Tag Purification Resin (NiNTA) were purchased from Sigma‐Aldrich (St. Louis, MO, USA). Protein inhibitor cocktail (PIC) and lysozyme were purchased from Nacalai Tesque, Inc (Kyoto, Japan). 12% Mini‐PROTEAN TGX Precast Protein Gels, 12‐well, were purchased from Bio‐Rad (Hercules, CA, USA). Amicon centrifugal filters were purchased from Merck. In‐Fusion HD Cloning Kit was purchased from Takara Bio (Kusatsu, Japan). All experiments were performed using ultrapure water purification system from a Milli‐Q Integral MT10 type 1 (Millipore, Burlington, MA, USA).

#### Sequence data collection

Methionine adenosyltransferase sequences from *M. jannaschii* (UniProt: Q58868, METE_METJA), *Escherichia coli* (UniProt: Q58605, METK_METJA), and MAT1A from *Homo sapiens* (UniProt: Q00266, METK1_HUMAN) were used to conduct NCBI BLAST [58] searches across the nonredundant database. Furthermore, the database search conditions were filtered based on the NCBI recommended e‐value cutoff, that is, 1e‐5, and only search results with query coverage > 90% and sequence identity > 55% were considered in constructing the initial sequence dataset. The collected sequences were then subjected to reciprocal BLAST searches to confirm orthologous relationship(s), as it is a common computational method for predicting putative orthologs. METK sequences for archaea from the phylogenetic group *asgard* (superphylum) were not considered in this study owing to the lack of taxonomic classifications associated with the sequences that were reported as hits in the NCBI BLAST searches.

#### Sequence analyses

The CD‐HIT program [59] was utilized to reduce sequence redundancy, by clustering at an 80% sequence identity cutoff threshold, with default settings. The Gblocks program [60] was implemented to identify highly conserved sites across sequence alignments, with assistance from the secondary structure information (from experimentally solved crystal structures) to guide the alignments. Sequence alignment was carried out using the MAFFT computer program [61], followed by a manual check to identify any errors [59]. To reduce the sequence redundancy, we used CD‐HIT, by clustering at 80% sequence identity cutoff threshold with default settings. The Gblocks program [60] was used to identify highly conserved sites across sequence alignments with assistance from the secondary structure information (from experimentally solved crystal structures) to guide the alignments. Sequence alignment was carried out using the MAFFT [61], followed by manual curation to check for any errors. Sequence alignments for WebLogo of active site residues and interface residues are provided in Appendices [Link], [Link], [Link].

#### SSN studies

Sequence similarity networks were constructed based on the output obtained from the EFI‐Enzyme Similarity Tool (EST) server [62], and subsequently, the cytoscape program (https://cytoscape.org/) was utilized to explore the SSNs via the organic layout. Here, we managed the initial dataset using CD‐HIT‐based clustering by selecting a sequence identity threshold of 80%. The ‘organic’ layout algorithm was chosen for graphical clustering.

#### Phylogenetic analysis and ancestral sequence reconstruction

ModelTest computer program [63] was implemented to select the best evolutionary model for sequence alignments based on the Bayesian inference criterion. The LG model [64] with invariant sites (+I) for discrete gamma categories (+G4) was selected to construct the maximum‐likelihood (ML) phylogenetic trees. Initially, we constructed ML‐based trees with the IQ‐TREE program.

#### Phylogenetic analysis and ancestral sequence reconstruction

ModelTest [63] was used to pick out the best evolutionary model for sequence alignments based on the Bayesian inference criterion. The LG model [64] with invariant sites (+I) for discrete gamma categories (+G4) was selected to construct maximum likelihood (ML) phylogenetic trees. Initially, we constructed ML‐based trees with the IQ‐TREE program [65] (using 10,000 bootstrap replicates) for the datasets, to inspect the topology distribution within the dataset. The confidence level for the nodes was assessed using Felsenstein's bootstrap method [66], and the consensus tree was redrawn using FigTree (http://tree.bio.ed.ac.uk/software/figtree/). The tree file from the IQ‐TREE program output was parsed to obtain the ancestral sequences at the highlighted nodes. In addition, the phylogenetic program MEGA X was consulted to check for ancestral sequences [67]. The NCBI taxonomy database was also consulted to check for major phylogenetic classifications [68].

#### Molecular modeling and structural studies

Ancestral sequences (of interest) were modeled using the Swiss model at the ExPASy server (https://swissmodel.expasy.org/). 3D models were visualized using pymol (https://pymol.org/2), to identify, tabulate, and study the key interaction residues. The overall quality of the 3D models depends directly on the shared sequence identity between the target sequence and the sequence of the template structure; therefore, we utilized template structures with high sequence similarity. Interface residues were identified using pymol and the PISA server (https://www.ebi.ac.uk/pdbe/pisa/). Structural superposition studies were performed using the mtm‐align server [69]. The 17 sequences for Fig. 6, Figs S4 and S5 were collected using blastp and the PDB database for sequence search. *Escherichia* *coli* (PDB 1RG9), human (PDB 4NDN), and pfMAT (PDB 6S83) sequences were used as input sequences for each search, and one representative sequence for each organism was manually selected. Two isoform structures were selected for human MAT, hMAT1A (6SW5), and hMAT2A (4NDN).

#### Charge and hydrophobicity calculations

Per‐site hydrophobicity and hydrophilicity calculations were carried out using the ProtScale program [70] from the Expasy server (https://web.expasy.org). We implemented the default ‘Kyte–Doolittle’ scale [71] for the calculations, with a residue window size of three, while the rest of the settings were left as default. Charge distribution per‐site was calculated using the charge server from EMBOSS (http://www.bioinformatics.nl/cgi‐bin/emboss/charge). A window length of size 3 was implemented, and the rest of the settings were left as default.

#### Evolutionary rate calculations

The site‐specific evolutionary rates were inferred using the IQ‐TREE program, by implementing the estimated model parameters and applying an empirical Bayesian approach to assign site rates as mean over rate categories.

#### Hierarchical clustering with pvclust

An r language package was utilized to assess the hierarchical clustering pattern, as it provides statistical support for each cluster through *P*‐values. It provides two types of *P*‐values: AU *P*‐value and BP value. The AU *P*‐value, which is computed using multiscale bootstrap resampling, is a better approximation to the unbiased *P*‐value than the BP value computed using normal bootstrap resampling.

#### Structural analysis

Structural superpositions were carried out employing the PDBeFold [72] server with default parameters, employing pdbs chain A of 1MXA, 2P02, and 4L4Q. Subdomain comparison was assessed following ECOD domain delimitation and manual dissection of archaeal MAT (Fig. S8).

#### Enzyme cloning

CrenArc, EuryArc, ArcheaArc MAT, and the hMAT1A codon‐optimized gene for *E. coli* were ordered from Twist Bioscience, San Francisco, and cloned into pet28a vector by infusion cloning using In‐Fusion HD Cloning Kit by following the kit protocol. Primers used for cloning are listed in Table 1.

**Table 1:** Primers for infusion cloning.

Name	Froward primer	Reverse primer
Pet28a_linearization	5′‐TCCGTCGACAAGCTTGCGGCCGCAC‐3′	5′‐GCGGCACCAGGCCGCTGCTGTGATG‐3′
MAT_amplification	5′‐GCAAGCTTGTCGACGGAGCTCGAATTC‐3′	5′‐GCAAGCTTGTCGACGGAGCTCGAATTC‐3′

#### Protein expression and purification

CrenArc, EuryArc, and ArcheaArc MAT were expressed and purified following the previously published protocol [48]. Briefly, the plasmid encoding the genes for CrenArc, EuryArc, and ArcheaArc MAT in *E. coli* BL21(DE3) cells was growing at 37 °C in LB medium with 50 µg·mL^−1^ kanamycin. Once OD_600_ reached 0.6, culture was cooled down to 16 °C and induction was done with 1 mm IPTG overnight at 16 °C with shaking at 230 r.p.m. Cells were harvested by centrifugation at 4648 *g* for 20 min and stored at −80 °C until use. The bacterial pellet was resuspended in lysis buffer that contains Tris/HCl (50 mm), NaCl (300 mm), imidazole (10 mm), lysozyme (0.3 mg·mL^−1^), benzonase (2 U), and PIC, pH 8. The cells were incubated with lysis buffer for 30 min to 1 h, followed by sonication (Digital Sonifier Models 250, 5 s, on 5 s off for 5 min) on ice. Cell debris was removed by centrifugation at 20,960 *g* for 1 h. The supernatant was loaded onto the NiNTA chelating column, which was equilibrated with lysis buffer previously. The column was washed with wash buffer 1 that contained Tris/HCl (50 mm), NaCl (300 mm), and imidazole (50 mm), pH 8. The second wash with wash buffer 2 contained Tris/HCl (50 mm), NaCl (300 mm), and imidazole (100 mm), pH 8. Finally, the column was eluted with elution buffer containing Tris/HCl (50 mm), NaCl (300 mm), imidazole (500 mm), and glycerol (10%), pH 8. The protein content was checked by the NanoDrop 2000 spectrophotometer (Thermo Scientific), and elution buffer containing proteins was dialyzed using Amicon centrifugal filters 30,000 MWCO with dialysis buffer containing Tris/HCl (50 mm), DTT (1 mm), and glycerol (10%), pH 8, and stored at −80 °C.

hMAT1A pellet was processed like archaea but used lysis buffer containing 20 mm NaH_2_PO_4_, 300 mm NaCl, 10 mm imidazole, 1 mg·mL^−1^ lysozyme, 1 µL benzonase, and PIC, pH 7.8, and incubated with lysis buffer for 30 min to 1 h. Cells were lysed by sonication (Digital Sonifier Models 250, 5 s on 5 s off for 5 min) on ice. After centrifugation at 12,000 r.p.m. for 1 h, the supernatant was loaded onto the NiNTA column. First, the column was washed with wash buffer 1 containing NaH_2_PO_4_ (50 mm), NaCl (300 mm), and imidazole (50 mm), pH 8, then washed with wash buffer 2 containing NaH_2_PO_4_ (50 mm), NaCl (300 mm), and imidazole (100 mm), pH 8. Finally, the column was eluted with elution buffer containing NaH_2_PO_4_ (50 mm), NaCl (300 mm), imidazole (500 mm), and glycerol (10%), pH 8. Fractions containing protein were concentrated with exchange buffer Tris/HCl (25 mm) and KCl (80 mm), pH 8, using Amicon centrifugal filters 30,000 MWCO. The purity of the protein was confirmed by SDS/PAGE. Protein concentration was 2.5, 6.2, 4.4, and 14 mg·mL^−1^ for CrenArc, EuryArc, ArcheaArc, and hMAT1A, respectively.

#### MAT activity assay

Activity assay was performed as previously reported [73]. Briefly, ATP/GTP/CTP/UTP (5 mm), methionine (10 mm), HEPES (100 mm), MgCl_2_ (10 mm), KCl (50 mm), and MAT (20 µm) were mixed in water, following which the pH of the mixture was adjusted to 8. Reactions were incubated at 37 °C for eukarya and at 55 °C for archaea in a thermomixer comfort system (Eppendorf, Hamburg Germany) for 1 h. The reaction was quenched with acetonitrile, followed by centrifugation at 12,000 r.p.m. for 5 min to precipitate the enzymes. Finally, the supernatant was filtered through a 0.22‐µm filter (Merck) and injected into the ultra‐performance liquid chromatography (UPLC) for analysis. UPLC method (UPLC Acquity H class, Waters, MA USA): Diluted reaction aliquots were analyzed using UPLC with a HILIC column (SeQuant^®^ ZIC®‐cHILIC 3 µm, 100 Å 150 × 2.1 mm PEEK‐coated HPLC column). An isocratic method was used with 35% solvent A (100 mm ammonium acetate, pH 5.3) and 65% solvent B (acetonitrile) for 15 min. Each injection had a volume of 3 µL, with a flow rate of 0.2 mL·min^−1^, and was detected at a wavelength of 260 nm.

For kinetic analysis, SGM was purified using the UPLC method mentioned above and standard curves were plotted for SGM and SAM. For kinetic assay of ArchaeaAnc (0.5 µm) and hMAT1A (0.5 µm), concentrations of the ATP and GTP were in the range of 0.1 to 2 mm and a saturating concentration of methionine 10 mm was used. Using the above‐mentioned buffer, temperature was 55 °C for ArchaeaAnc and 37 °C for hMAT1A. Data were analyzed by UPLC, and the kinetic parameters were determined using the Michaelis–Menten equation using the graphpad prism 7.02 (San Diego, CA, USA; https://www.graphpad.com).

## Conflict of interest

The authors declare no conflict of interest.

## Author contributions

BPSC and PL conceived the idea of this study. BPSC, MG, and DM performed the experiments. BPSC, MG, ST‐P, and PL analyzed the data. BPSC, MG, STP, and PL wrote the manuscript and prepared the figures.

## Supporting information


**Scheme S1**. *S*‐adenosylmethionine (SAM) biosynthesis and its functions by secondary metabolites.
**Fig. S1**. Sequence similarity networks for MAT enzymes.
**Fig. S2**. The evolutionary trends adapted by the interface sites (located along the large interface of the homo‐tetramer) as well the active sites.
**Fig. S3**. An interface view of three representative MAT structures and Weblogo of large interface residues from the three domains of life.
**Fig. S4**. Structural alignment of the residues depicting the 51 interface residues from 17 MAT crystal structures.
**Fig. S5**. Structure‐based sequence alignment.
**Fig. S6**. Clustering analysis of hydropathy (A) and charge (B) of representative structures from the three domains of life.
**Fig. S7**. Structural comparison of MAT representatives from the three domains of life.
**Fig. S8**. Comparison of MAT subdomains within domains of life.
**Fig. S9**. Structural comparison of MAT subdomains across the domains of life.
**Fig. S10**. SDS/PAGE of ArchaeaAnc MAT protein purification.
**Fig. S11**. SDS/PAGE of CrenAnc MAT protein purification.
**Fig. S12**. SDA/PAGE of EuryAnc MAT protein purification.
**Fig. S13**. SDS/PAGE of hMAT1A purification.
**Fig. S14**. UPLC chromatogram of the reaction between NTP, methionine, and ArchaeaAnc.
**Fig. S15**. UPLC chromatogram of the reaction between NTP, methionine, and CrenAnc.
**Fig. S16**. UPLC chromatogram of the reaction between NTP, methionine, and EuryAnc.
**Fig. S17**. UPLC chromatogram of the reaction between NTP, methionine, and hMAT1A.
**Fig. S18**. Kinetic parameters for the SAM and SGM formation by ArchaeaAnc and hMAT1A.

**Appendix S1**. Bacteria.

**Appendix S2**. Archaea.

**Appendix S3**. Eukarya.

## Data Availability

The data generated/used in this study are available in the article and online supplementary materials. In addition, any other data underlying this article will be readily shared upon request from the corresponding author.
