# Supplementary material for: Implications of divergence of methionine adenosyltransferase in archaea
Source: FEBS Open Bio. 2021 Nov 5;12(1):130–45. doi: 10.1002/2211-5463.13312 (PMC8727953; doi:10.1002/2211-5463.13312)
Supplement: Supplementary file 1 — Scheme S1. S‐adenosylmethionine (SAM) biosynthesis and its functions by secondary metabolites. Fig. S1. Sequence similarity networks for MAT enzymes. Fig. S2. The evolutionary trends adapted by the interface sites (located along the large interface of the homo‐tetramer) as well the active sites. Fig. S3. An interface view of three representative MAT structures and Weblogo of large interface residues from the three domains of life. Fig. S4. Structural alignment of the residues depicting the 51 interface residues from 17 MAT crystal structures. Fig. S5. Structure‐based sequence alignment. Fig. S6. Clustering analysis of hydropathy (A) and charge (B) of representative structures from the three domains of life. Fig. S7. Structural comparison of MAT representatives from the three domains of life. Fig. S8. Comparison of MAT subdomains within domains of life. Fig. S9. Structural comparison of MAT subdomains across the domains of life. Fig. S10. SDS/PAGE of ArchaeaAnc MAT protein purification. Fig. S11. SDS/PAGE of CrenAnc MAT protein purification. Fig. S12. SDA/PAGE of EuryAnc MAT protein purification. Fig. S13. SDS/PAGE of hMAT1A purification. Fig. S14. UPLC chromatogram of the reaction between NTP, methionine, and ArchaeaAnc. Fig. S15. UPLC chromatogram of the reaction between NTP, methionine, and CrenAnc. Fig. S16. UPLC chromatogram of the reaction between NTP, methionine, and EuryAnc. Fig. S17. UPLC chromatogram of the reaction between NTP, methionine, and hMAT1A. Fig. S18. Kinetic parameters for the SAM and SGM formation by ArchaeaAnc and hMAT1A. [file FEB4-12-130-s003.pdf]

Supplementary material for:

Implications of divergence of methionine adenosyltransferase in archaea

Bhanu Pratap Singh Chouhan, Madhuri Gade, Desirae Martinez, Saacnicteh Toledo-Patino and Paola Laurino*

Protein Engineering and Evolution Unit, Okinawa Institute of Science and Technology Graduate University, 1919-1 Tancha, Onna, Okinawa, Japan 904-0495.

Corresponding author: Paola Laurino **Email:** paola.laurino@oist.jp

Sequences of the enzymes used in this study.

hMAT1A sequence
MNGPVDGLCDHSLSEGVFMFTSESVGEGHPDKICDQISDAVLDAHLKQDPNAKVACET
VCKTGMVLLCGEITSMAMVDYQRVVRDTIKHIGYDDSAKGFDFKTCNVLVALEQQSPDI
AQCVHLDRNEEDVGAGDQGLMFGYATDETEECMPLTIILAHKLNARMADLRRSGLLPW
LRPDSKTQVTVQYMQDNGAVIPVRIHTIVISVQHNEDITLEEMRRALKEQVIRAVVPAKY
LDEDTVYHLQPSGRFVIGGPQGDAGVTGRKIIVDTYGGWGAHGGGAFSGKDYTKVDR
SAAYAARWVAKSLVKAGLCRRVLVQVSYAIGVAEPLSISIFTYGTSQKTERELLDVVHKN
FDLRPGVIVRDLDLKKPIYQKTACYGHFGRSEFPWEVPRKLVF

ArchaeaAnc sequence
MRNIVVEQLNWTPVEEQQVELVERKGLGHPDYIADGISEAVSRALCKYYLERFGTILHH
NTDQVQVVGGQASPRFGGGEVIQPIYILLSGRATTEVDGEKVPIGTIALKAAKDWLREN
FRFLDPERHVIIDCRIGQGSADLVGVFERGKSVPLANDTSFGVGFAPLSTTERLVFETER
FLNSKFKKKYPAVGEDIKVMGLRRGKKITLTIAAAMVSRFVKDMDEYLSVKEEVKDAVQ
DLASKYTPYDVEVYVNTADKPEKGIFYLTVTGTSAEMGDDGSTGRGNRCNGLITPMRP
MSMEATAGKNPVSHVGKIYNILANQIAQRIYEEVKGVKEVYVRLLSQIGKPIDQPLIANVQ
VIPEDGYLTSDMKREIEAIADEWLANITKITEMILEGKVSLF

EuryAnc sequence
MRNIVVEELNRTPIEEQQVELVERKGIGHPDSIADGIAEAVSRALCKEYMERFGAILHHN
TDQVQVVGGQAHPRFGGGEVIQPIYILLSGRATKEVDGEKIPVDTIALKAAKDYLRETFR
HLDLERHVIIDCRIGQGSVDLVGVFNRQKPVPLANDTSFGVGYAPLSETERLVFETERFL
NSEFKKKYPAVGEDIKVMGLRKGDKITLTIAAAMVDRYVSNMDEYLEVKEEIKDAVKDLA
SKYTDREVEVYVNTADDPEKGCFYLTVTGTSAEMGDDGSVGRGNRCNGLITPNRPMS
MEATAGKNPVSHVGKIYNILANQIAQDIAEEVEGVKEVYVRILSQIGKPIDQPLVASVQVI
PEDGYSISDMEREVKEIADEWLANITKITEMILEGKISVF

CrenAnc sequence
MRNIVVEQLRWQPVEELQVELVERKGLGHPDYIADAISEAASRELSKYYLERFGTILHH
NLDKVLVVGGQASPRFGGGEVIQPIYILVSGRATTEVDGEKVPIGTIILKAAKDWIRENFR
FLDPERHVIIDYRVGQGSADLVGIFELGKSVPLANDTSFGVGFAPLSTTERLVFETERLL
NSKFKAKFPAVGEDVKVMGLRRGKKIKLTIAAAIISRFVKDMDEYLSVKEEVKDAVLDLA
SKIAPYDVEVYVNTADKPEKGIFYLTVTGTSAEHGDDGATGRGNRANGLITPMRPMSM
EATAGKNPVSHVGKIYNVLANQIAQRIYEEVKGVKEVYVELLSQIGKPINEPLIANVQVIP
EEGELTSDMKREIEAIADEELDRITKITEMILEGKVSLF

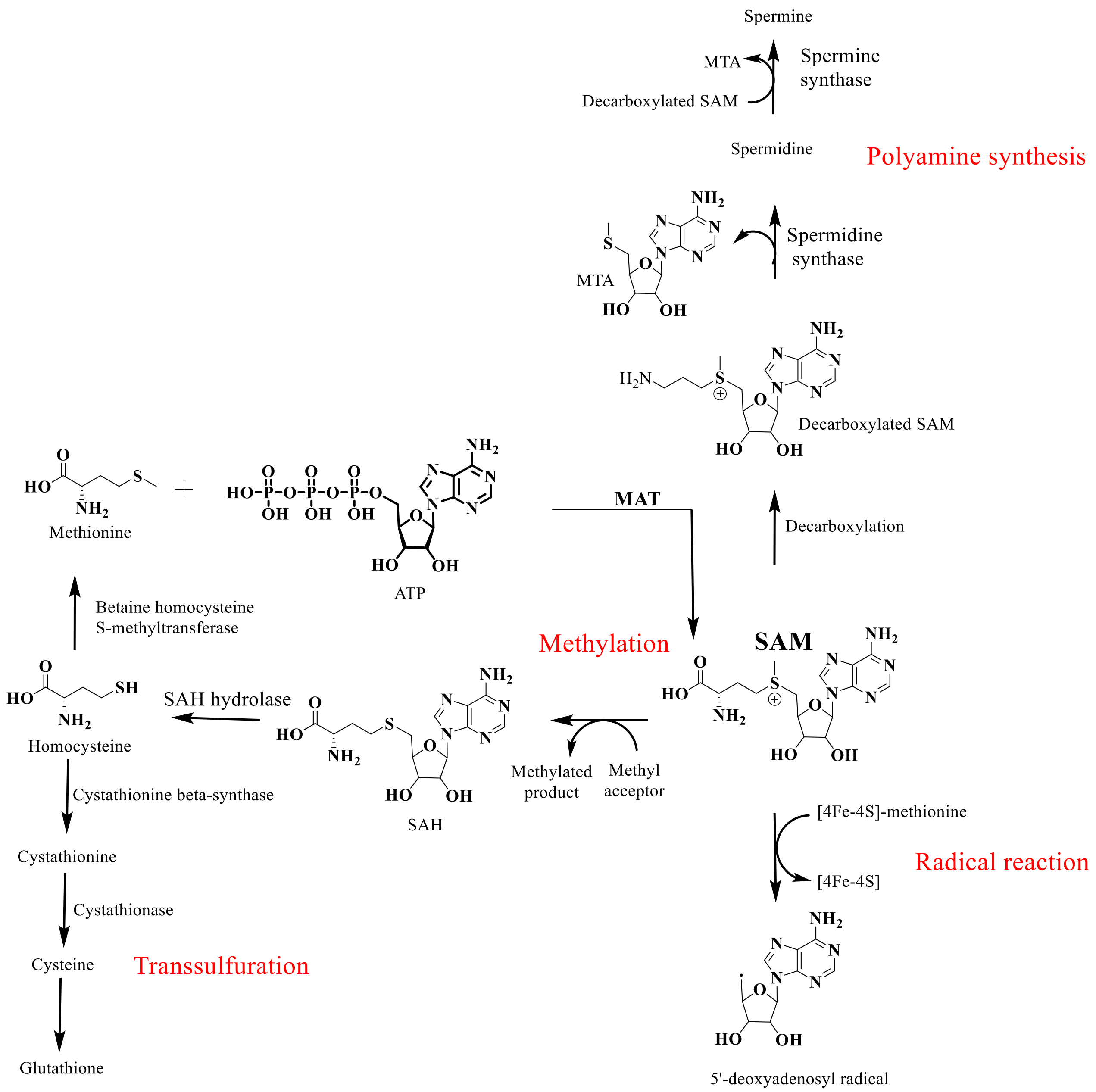

Scheme S1 S-adenosylmethionine (SAM) biosynthesis and its functions by secondary metabolites.

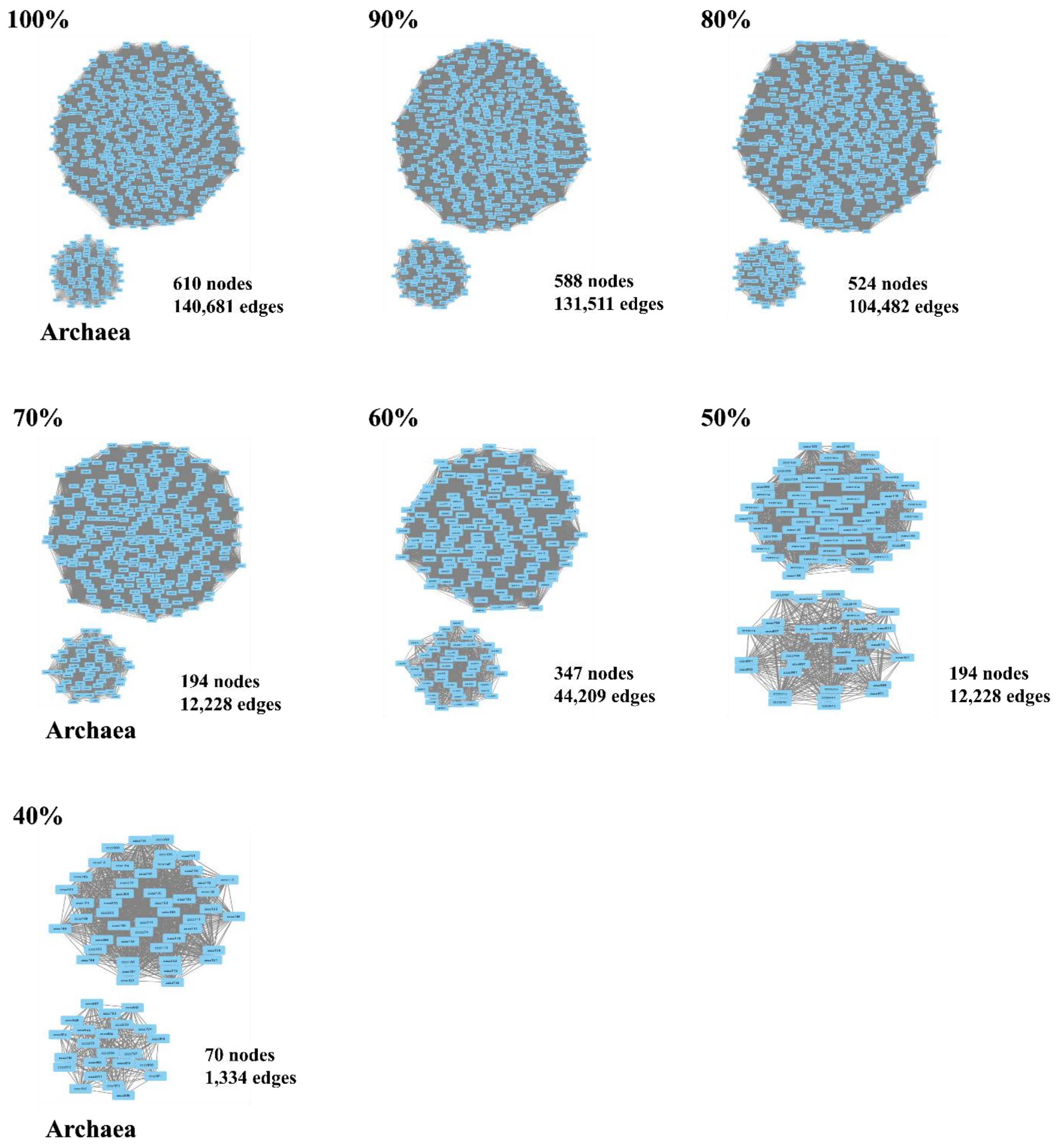

Figure. S1 Sequence similarity networks for MAT enzymes. The sequence networks created with EFI tool (post CD-HIT dataset reduction) and visualized at different sequence identity percentage (with default recommendations from EFI web server), visualized with cytoscape. As noted in the figure the two sub-networks form distinct topologies. Each topology (100%-40%) is further annotated with the respective number of nodes and edges.

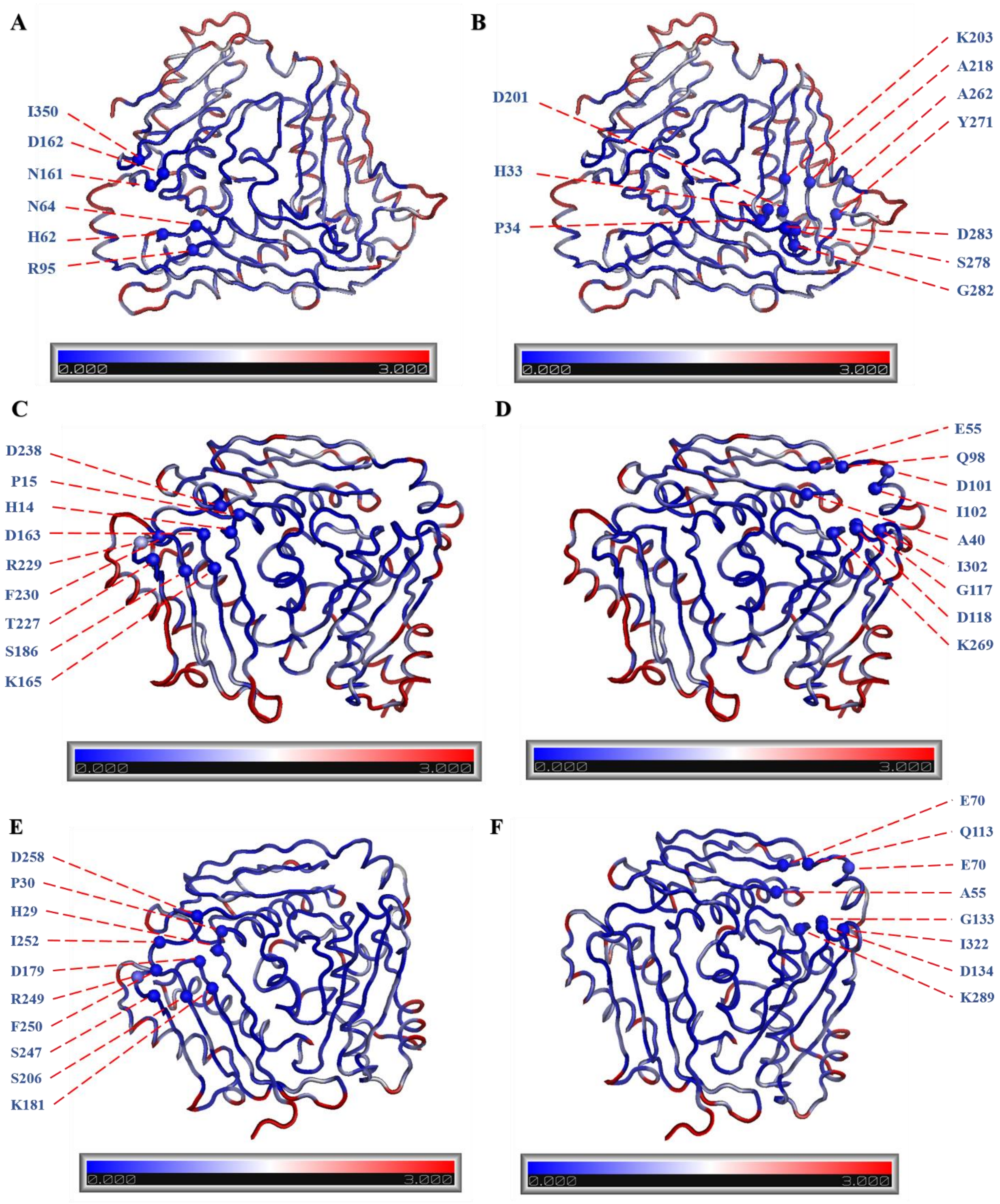

Figure. S2 The evolutionary trends adapted by the interface sites (located along the large interface of the homo-tetramer) as well the active sites. Upon normalizing the ML evolutionary rates, we mapped them on to the representative structures (as a function of B-factors). Here, the rates per-site are designated with a scale wherein the relatively

‘slowly’ evolving regions are highlighted in blue (~ 0 ML rates), while the sites experiencing ‘faster’ evolutionary rates are highlighted in red (~3 ML rates). Residues located within a ~4Å of SAM are highlighted in blue and labelled accordingly.

Highlighted here, are the evolutionary rates with an emphasis on the interface region of the tkMAT from archaea *Thermococcus kodakarensis* (PDB: 4L4Q) represented in panels **A** and **B**. The highlighted residues are mentioned below:

Chain A: H62, N64, R95, N161, D162, I350
Chain B: H33, P34, D201, K203, A218, A262, Y271, S278, G282, D283

eMAT from bacteria *Escherichia coli* (PDB: 1RG9) represented in panels **C** and **D**. The highlighted residues are mentioned below:

Chain A: H14, P15, D163, K165, S186, T227, R229, F230, D238,
Chain B: A40, E55, Q98, D101, I102, G117, D118, K269, I302

region of the hMAT2A from eukarya *Homo sapiens* (PDB: 4NDN) represented in panels **E** and **F**. The highlighted residues are mentioned below:

Chain A: H29, P30, D179, K181, S206, S247, R249, F250, I252, D258,
Chain B: A55, E70, Q113, D116, I117, G133, D134, K289, I322

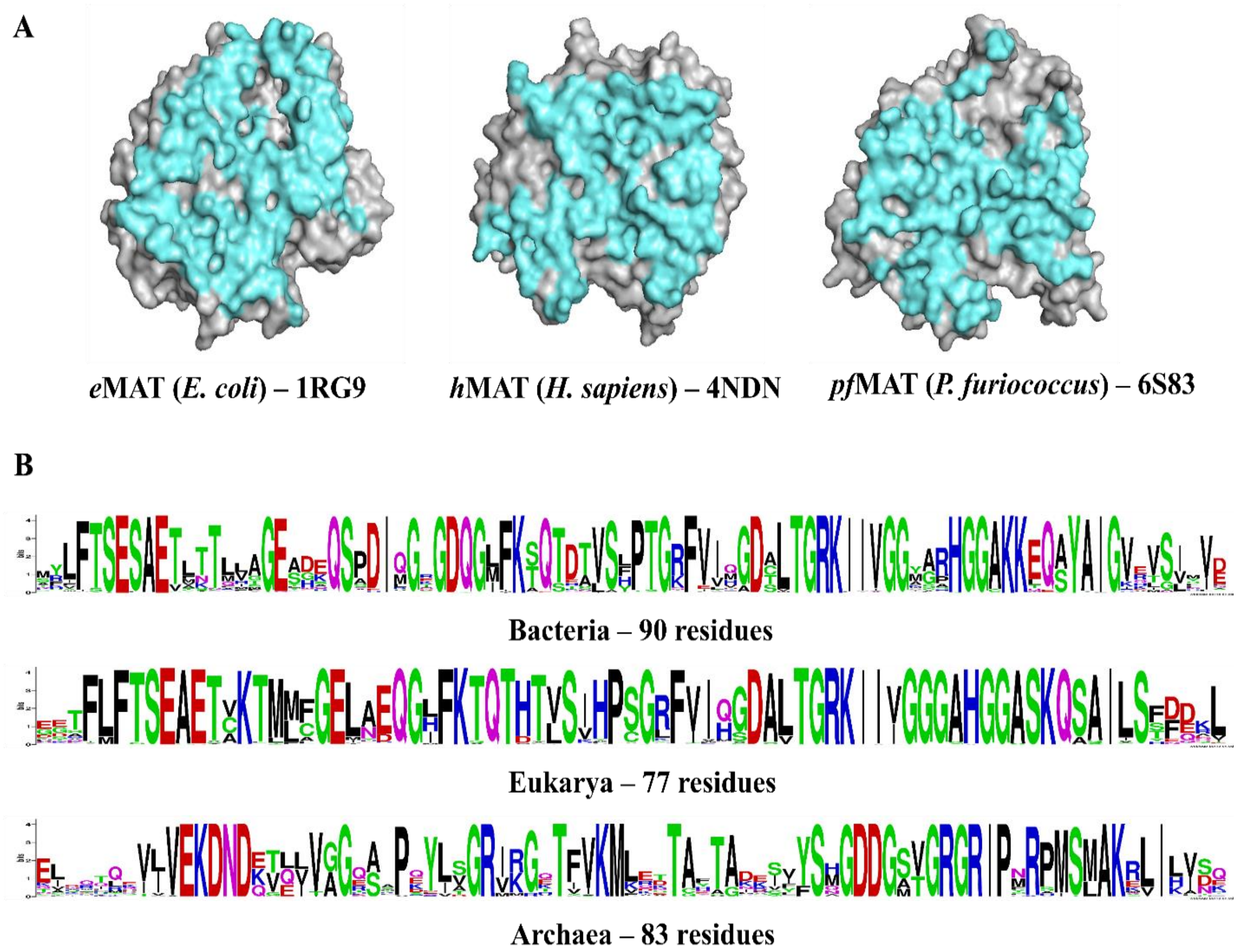

Figure. S3 An interface view of three representative MAT structures and Weblogo of large interface residues from the three domains of life (A) An interface view of three representative MAT structures from the three domains of life with *e*MAT from *E. coli* (PDB: 1RG9), *h*MAT2A from *H. sapiens* (PDB: 4NDN) and *pf*MAT from *P. furiosus* (PDB: 6S83), the interface residues are highlighted in cyan. PISA server was utilized to identify and extract the interface residues [https://www.ebi.ac.uk/pdbe/pisa/].

(B) The large interface residues are highlighted as WebLogo across the three domains based on sequence alignments performed for 90 interface residues in case of bacteria with corresponding residues for *e*MAT, 77 interface residues for *h*MAT2A and 83 interface residues for *pf*MAT. It is worth mentioning here that the residues are only extracted from one chain i.e., chain A and depending on the confirmation and the presence or absence of a ligand – the residue list may change. 526 sequences from bacteria (appendix 1), 49 sequences from archaea (appendix 2) and 273 sequences from eukarya (appendix 3) were

used to make WebLogo for interface residues. Details about the sequences search are in method sections.

A

Chains A vs B (51 Positions)

```
E. coli  (1RG9)                HLFTEAETYKMLGGEAGQGLFKTDAVFPTDCTGRKGGARGGAKQSAIGTSM
B. pseudomallei (3IML)         YLFTEAETLNLVAGEADQGLFKTDTVLPTDCTGRKGGAPGGAKQSAIGTSM
M. tuberculosis (3TDE)         RLFTEAETLTQHVGEGGQGLFKTDTVLPTDATGRKGGARGGAKQAAIGVGF
C. jejuni (4LE5)               YLFTEAEVFAKVGGEFNQGIFKTHTVLPTDSTGRKGGSPGGAKQSAIGTSS
T. thermophilus (5H9U)         RLVTEAETLTLFAGEADQGLFKTKTVLPSDTTGRKGGVPGGAKEAAIGVSR
N. gonorohhea (5T8S)           YLFTEAETLNLVAGEYDQGLFKTDTVLPTDCTGRKGGAPGGAKQSAIGTSS
U. urealitium (6RKA)           KIITEAEVLCLVAGENNQGIFKSETLISSDTTGRKGGGHGGAKQAAIGVAY
H. sapiens  (MAT2A - 4NDN)     FLFTEAETVKMLAGEAEQGLFKTHTVHPSDATGRKGGGAGGAKQSAIGLSS
H. sapiens  (MAT1A - 6SW5)     FMFTEAETVKMLCGEAEQGLFKTHTVHPSDATGRKGGGAGGAKQSAIGLSS
R. norvegicus (1QM4)           FMFTEAETVKMLCGEAEQGLFKTHTVHPSDATGRKGGGAGGAKQSAIGLSS
C. parvum(6C07)                FLFSEAETCKFMFGEKEQGMFKTHTLLPSDATGRKGGGAGGAKQSGIGLSY
E. hystolytica(3SO4)           FFFTEAETAKLLLGECEQGIFKTHTVYPSDATGRKGGGAGGAKQSAIGLSN
M. truncatula (6VCW)           FLFTEAETCKMMFGENEQGHFKTHTLHPSDATGRKGGGAGGAKQSAIGLSF
A. thaliana (6VCZ)             FLFTEAETCKMMFGENEQGHFKTHTLHPSDATGRKGGGAGGAKQSAIGLSF
P. furiosus  (6S83)            QVELENQVEVYLSGRRGTSFVKLIDTYTADDVGRGITRHSMAKRLQIGLVS
T. kodakarensis (4L4Q)         KVELENQVEVYLSGRRGTSFVKLIDTYTADDVGRGITRHSMAKRLQIGLVS
S. solfataricus (4K0B)         QVELENKTLVYIAGRKGTSFVKLVDTYTGDDTGRGITRPSLAKQLQIGLIN
                               #      # ## ##     # #### ## #  ####    # #   ## # #
```

B

```
Bacteria ancestor (model)      YLFTEAETLTLIAGEAGQGMFKTDTVHPTDCTGRKGGGAGGAKQAAIGLSM
E. coli* (1RG9)                HLFTEAETYKMLGGEAGQGLFKTDAVFPTDCTGRKGGARGGAKQSAIGTSM

Eukarya ancestor (model)       FLFTEAETAKMMFGEAEQGIFKTDTVHPSDCTGRKGGGAGGAKQSAIGLSF
H. sapiens* (MAT1A - 6SW5)     FMFTEAETVKMLCGEAEQGLFKTHTVHPSDATGRKGGGAGGAKQSAIGLSS

Archaea ancestor (model)       QVELENQVQVYLSGRRGTSFVKLITTYTADDTGRGITRPSMAKRLQIGLIN
P. furiosus* (6S83)            QVELENQVEVYLSGRRGTSFVKLIDTYTADDVGRGITRHSMAKRLQIGLVS
```

Figure. S4 Structural alignment of the residues depicting the 51 interface residues from 17 MAT crystal structures. (A) Alignment of the residues from the structural alignment depicting the 51 interface residues from 17 MAT crystal structures considered in this study. Here the organisms name is shown with their respective MAT PDB ID's. In the sequence alignment the conserved positions between bacteria and eukarya MAT are highlighted in red with shared conserved positions from archaea MAT highlighted in blue. Within the alignment the residues which were *not* identified as interface residues by PISA server are highlighted in bold and underlined. We chose a cutoff for including a maximum of five non-interface residues and this data set was utilized to test for the hierarchical clustering (using pvclust package from R language) with charge and hydrophobicity (physiochemical properties). Additionally, we also highlight 24 interface residues in the

alignment by '#' at the bottom of the alignment. A second dataset consisting of these 24 'interface residues only' positions was also utilized to conduct the clustering test (Fig. S6).

(B) A comparison of the 51 positions between representatives of the modern-day MAT from the three domains of life and respective ancestral sequences (from their domains). Here, Archaea ancestral MAT is ArchaeaAnc and the alignment depicts 6 changes in contrast to pfMAT (PDB: 6S83) with eukarya ancestral MAT depicting 8 changes when compared with hMAT1A (PDB: 6SW5) and bacteria ancestral MAT depicting 13 when compared with eMAT (PDB: 1RG9).

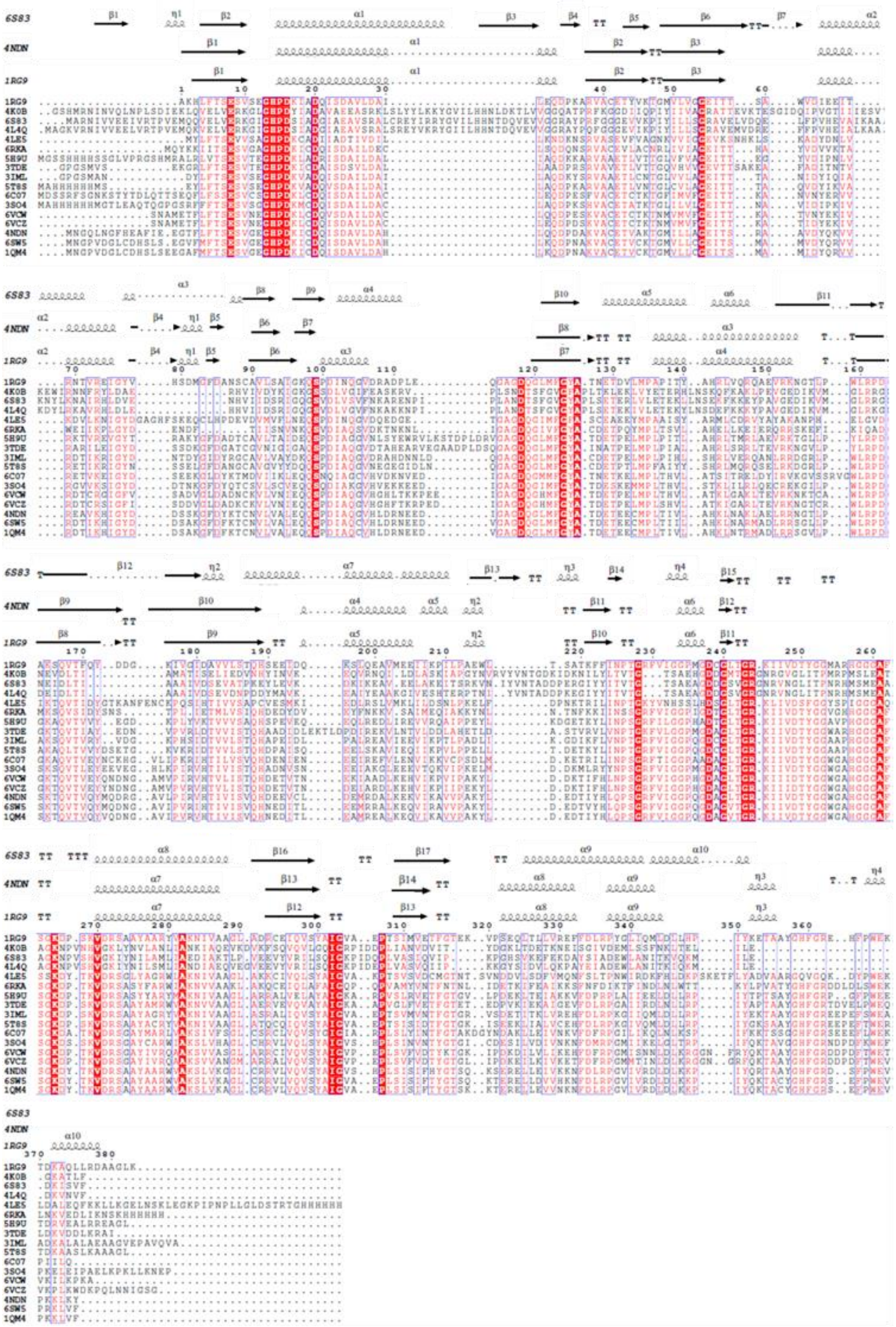

Figure. S5 Structure-based sequence alignment. Structure-based sequence alignment of MAT of the 17 PBDs structures considered in this study. Red letters show sequence identity and red background show similarity in the alignment. Secondary structure

elements of 1RG9, 4NDN AND 6S83 were compared with MAT structure-based sequence. The alignment was performed using ESprint 3.0. The 17 sequences were collected using blastp and the PDB database for sequence search. E. coli (PDB 1RG9), human (PDB 4NDN) and pfMAT (PDB 6S83) sequences were used as input sequences for each search and one representative sequence for each organism was manually selected. Two isoform structures were selected for human MAT, hMAT1A (PDB 6SW5) and hMAT2A (PDB 4NDN).

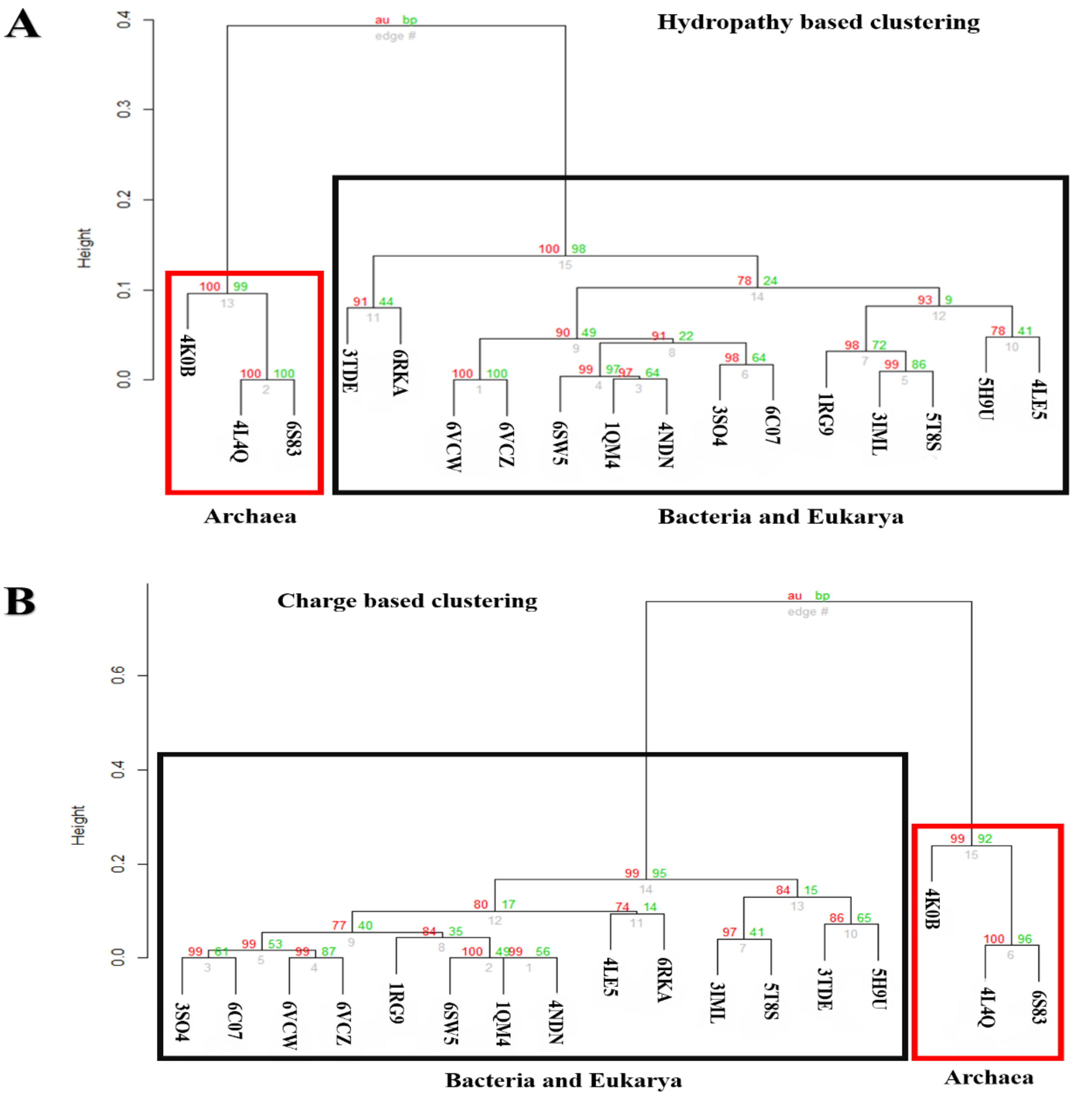

Figure. S6 Clustering analysis of hydropathy (A) and charge (B) of representative structures from the three domains of life.

Clustering analysis of the physiochemical properties (Hydropathy and Charge) of 24 structurally aligned large-interface residues from chain A of 17 MAT structures (PDBs list in the SI). Based on the 51 aligned positions we calculated the per-site hydropathy with the help of Kyte-Doolittle scale from the protscale server and per-site charges were calculated with EMBOSS charge server for the 17 MAT structures. Subsequently, we conducted clustering analysis with the help of pvclust package in R. This further provides a statistical score in terms of AU (approximately unbiased - in red) p-value and BP (bootstrap probability - in green) value for comparison of the clusters which reveals that the hydropathy **(A)** and charge distribution **(B)** cluster together for bacteria and eukarya MAT structures with archaea MAT structures clustering separately. In both the cases the archaea cluster is provided with high support values with both the AU and BP parameters, 100 % support for a distinct archaea cluster with respect to the two physiochemical properties each.

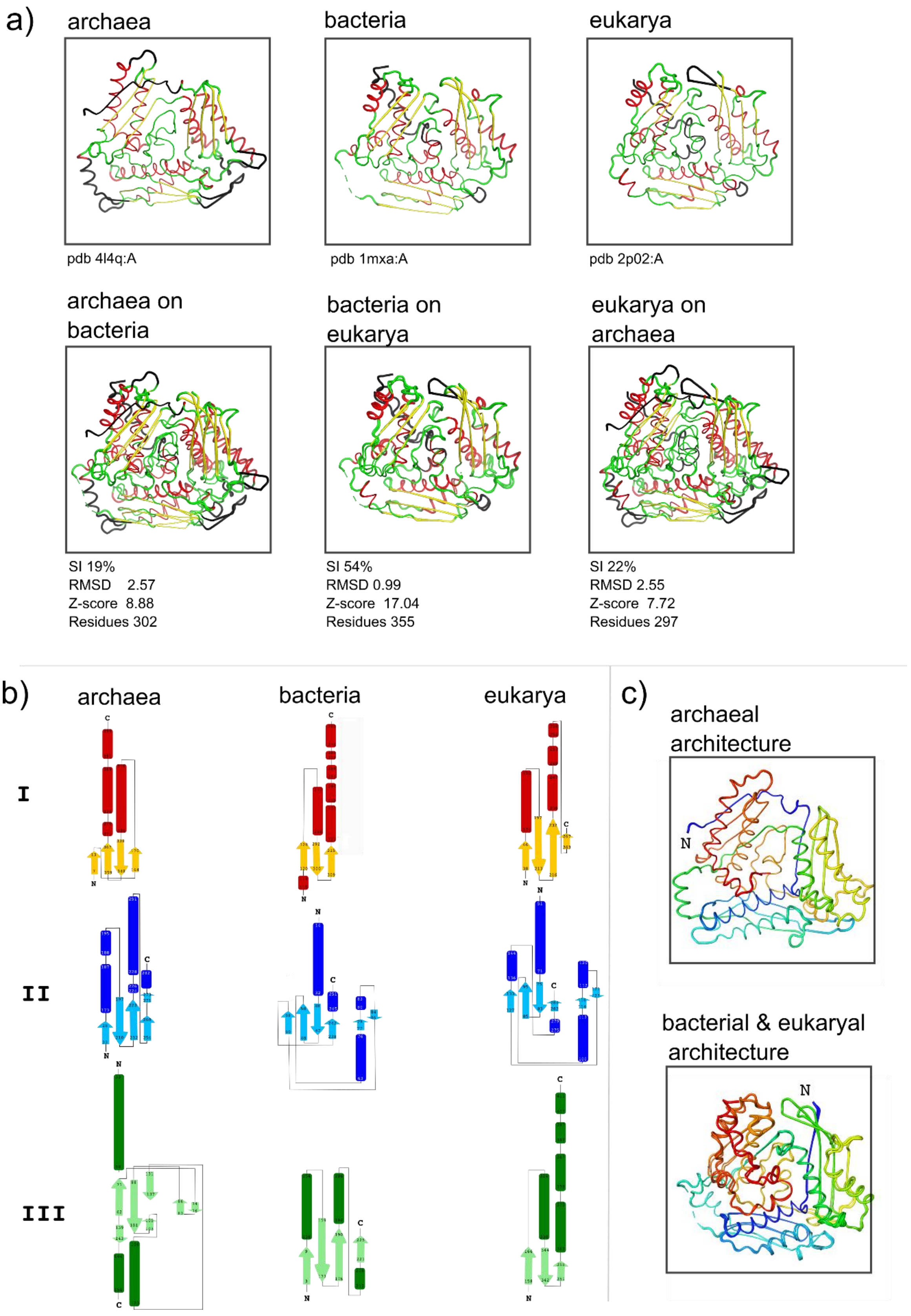
a)
archaea
bacteria
eukarya
pdb 4l4q:A
pdb 1mxa:A
pdb 2p02:A
archaea on bacteria
bacteria on eukarya
eukarya on archaea
SI 19%
RMSD 2.57
Z-score 8.88
Residues 302
SI 54%
RMSD 0.99
Z-score 17.04
Residues 355
SI 22%
RMSD 2.55
Z-score 7.72
Residues 297
b)
archaea
bacteria
eukarya
I
II
III
c)
archaeal architecture
N
bacterial & eukaryal architecture
N

Figure. S7 Structural comparison of MAT representatives from the three domains of life. (a) MAT structures for all domains are illustrated, followed by their structural superimpositions. Insertions larger than 3 amino acids, not present in all domains, are highlighted in black. Unexpectedly, bacterial and eukarya MATs show a better superposition compared to archaeal MAT. These features are not anecdotal for the selected pdbs (4l4q, 1mxa and 2p02). In fact, this structural tendency is observed for all to-date known MAT protein structures. (b) Topology connections for ach subdomain according to PDBsum (c) N to C-terminus rainbow coloring displays how archaeal MAT N-terminus segment-swapping into the vicinal sub-domain in contrast to their bacterial and eukaryal counterparts.

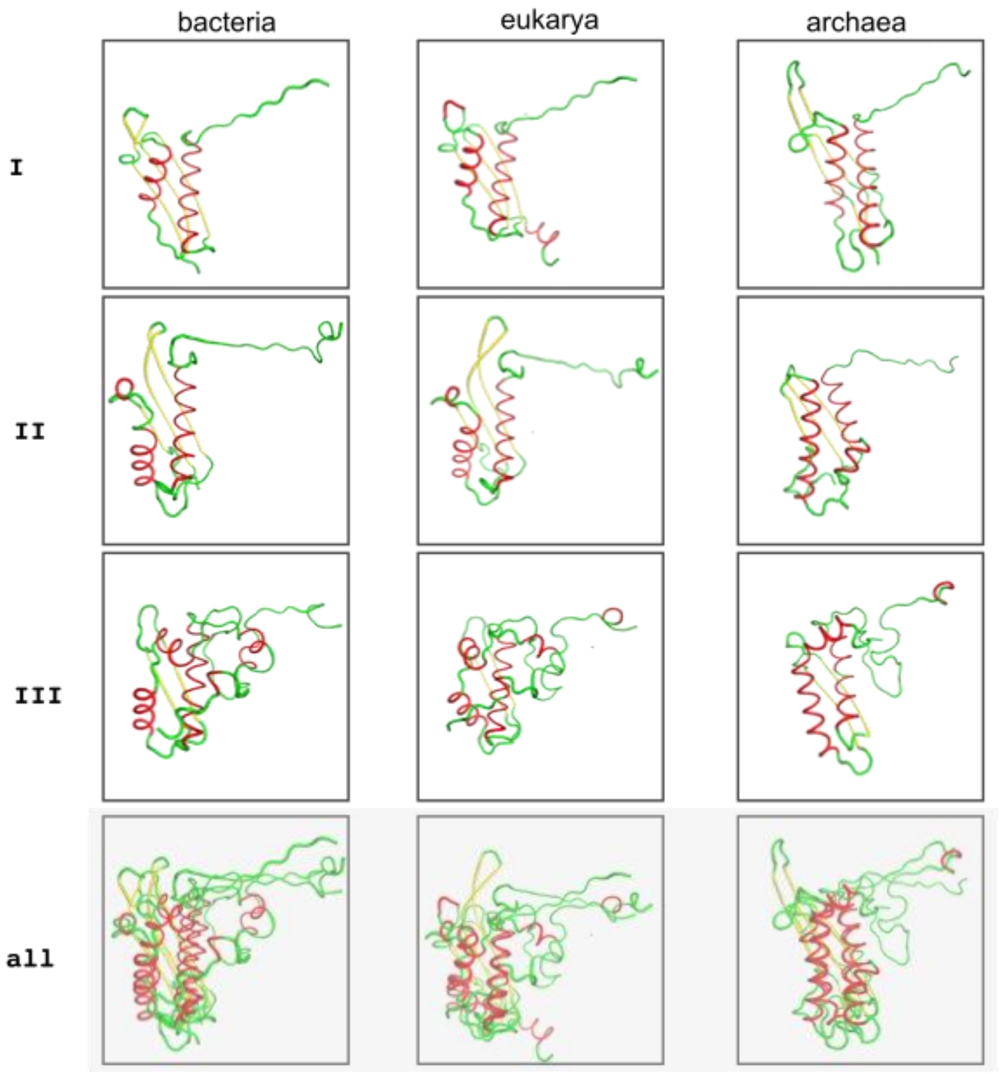

I on II	SI 7%<br>RMSD 2.97<br>Z-score 3.28<br>Residues 73	SI 6%<br>RMSD 2.47<br>Z-score 3.82<br>Residues 68	SI 18%<br>RMSD 2.75<br>Z-score 4.22<br>Residues 88
I on III	SI 12%<br>RMSD 3.21<br>Z-score 2.77<br>Residues 62	SI 14%<br>RMSD 2.78<br>Z-score 3.25<br>Residues 62	SI 17%<br>RMSD 2.51<br>Z-score 2.99<br>Residues 77
II on III	SI 15.5%<br>RMSD 2.94<br>Z-score 2.83<br>Residues 79	SI 7%<br>RMSD 3.11<br>Z-score 2.20<br>Residues 82	SI 10%<br>RMSD 2.77<br>Z-score 3.86<br>Residues 81

Figure. S8 Comparison of MAT subdomains within domains of life. Here we compare ECOD MAT subdomains I to III from bacteria (ID: e1mxaA1-3), eukarya (ID: e2p02A1-3), and manually dissected subdomains from archaea (pdb: 4l4qA, residues: 20-144; 154-272; and 278-405). Subdomain delimitation for archaea was performed employing bacterial and human ECOD domains as templates. Even though the topology within

subdomains is the same, Secondary structure element (SSE) orientation in space differs considerably. In fact, none of the superimposed units shows an RMSD value below 2.5 Å.

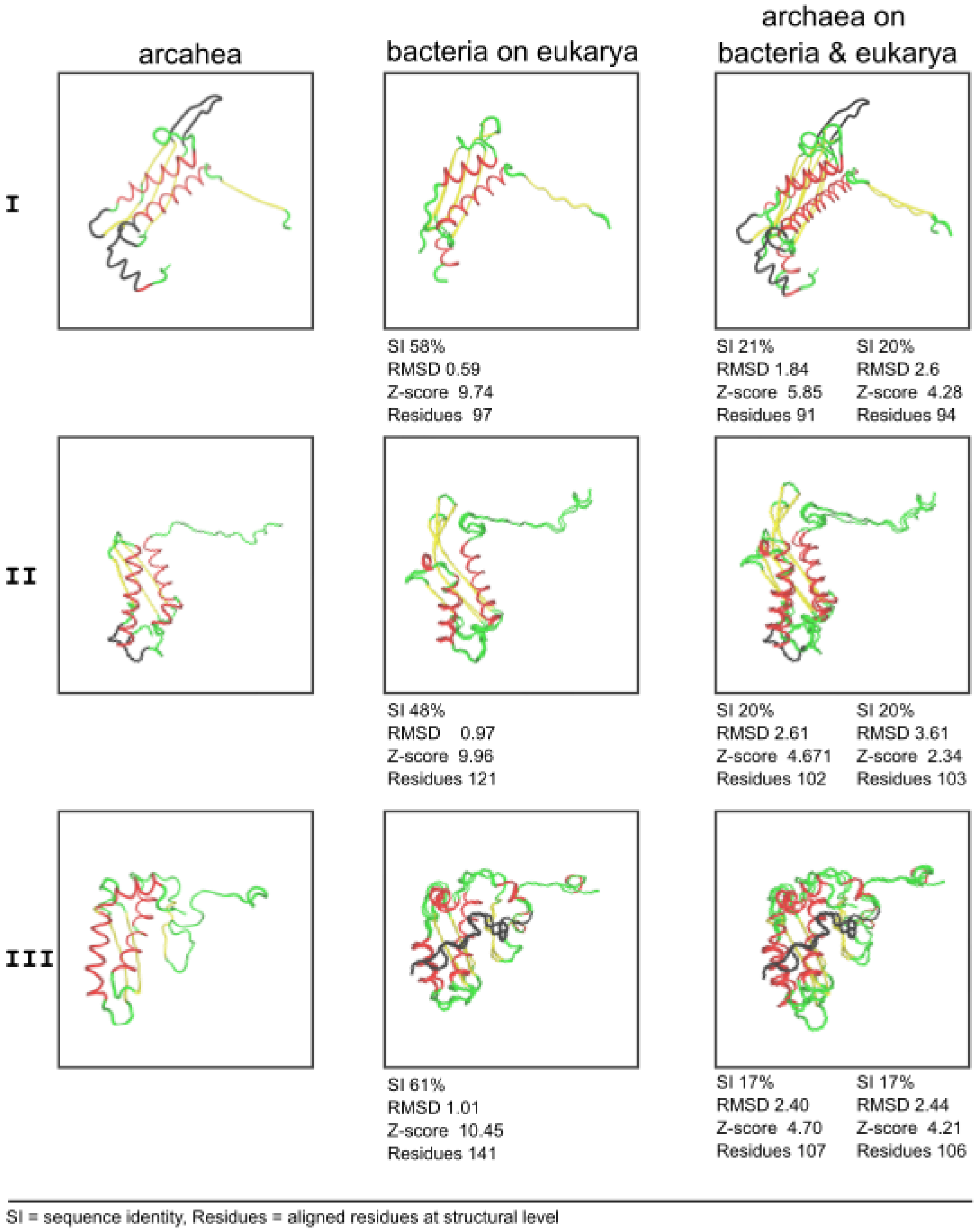

Figure. S9 Structural comparison of MAT subdomains across the domains of life. Here we compared ECOD domains I to III from eukarya (ID: e2p02A1-3), bacteria (ID: e1mxaA1-3) and manually dissected subdomains from archaea (Fig. S7). Even though all MAT subdomains can be superimposed across domains of life, superposition between bacteria and eukarya clearly shows a better structural agreement compared to archaea,

which correlate with their sequence identities. It is worth noticing that superposition of subdomains is largely better across domains of life than within them (Fig. S7). Insertions larger that 3 amino acids, not present in all domains, are highlighted in black.

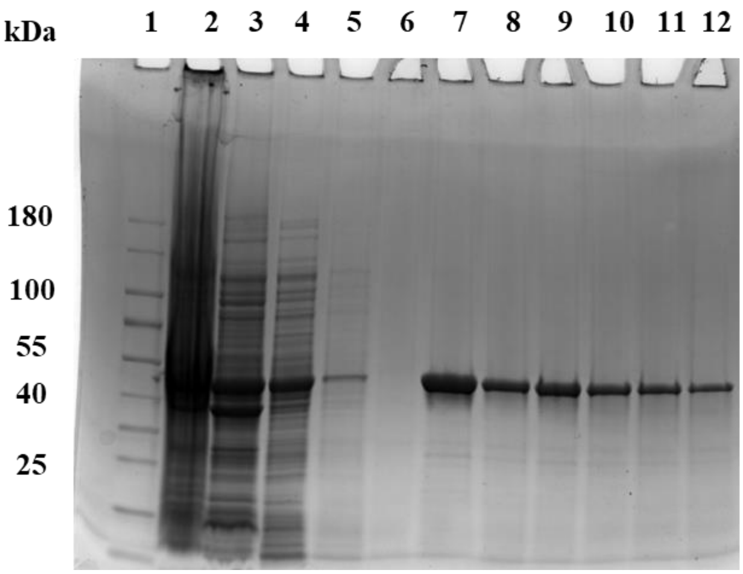

Figure. S10 SDS–polyacrylamide gel electrophoresis (SDA-PAGE) of ArchaeaAnc MAT protein purification. 12.5 % SDS–PAGE, Coomassie blue-stained. Lane 1- prestained molecular marker, lane 2- bacterial pellet, lane 3-supernatant, lane 4- flow through, lane 5-wash 1, lane 6- wash 2, lane 7- elution 1, lane 8- elution 2, lane 9- elution 3, lane 10- elution 4, lane 11- elution 5, lane 12-elution 6.

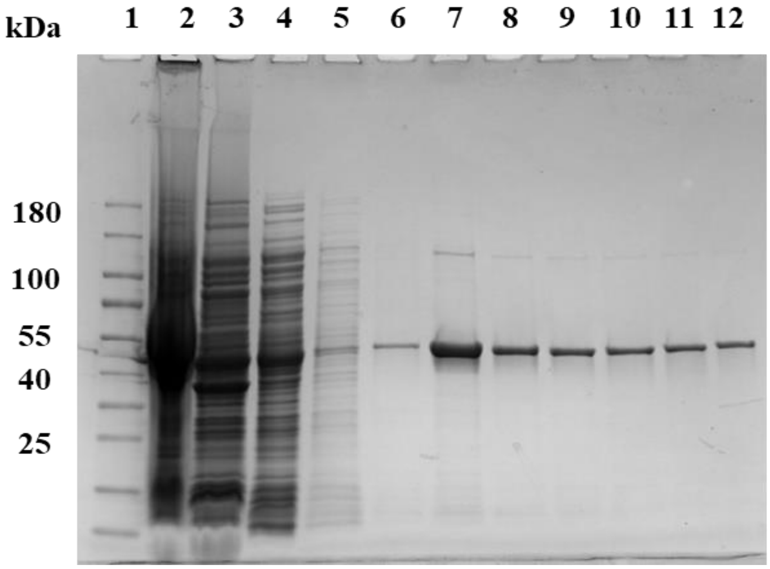

Figure. S11 SDS–polyacrylamide gel electrophoresis (SDA-PAGE) of CrenAnc MAT protein purification. 12.5 % SDS–PAGE, Coomassie blue-stained. Lane 1-prestained molecular marker, lane 2- bacterial pellet, lane 3-supernatant, lane 4- flow through, lane 5-wash 1, lane 6- wash 2, lane 7- elution 1, lane 8- elution 2, lane 9- elution 3, lane 10-elution 4, lane 11- elution 5, lane 12-elution 6.

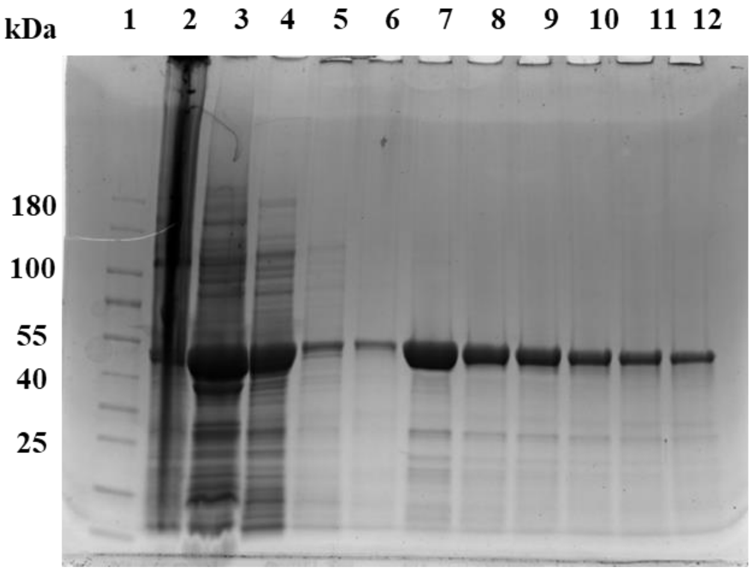

Figure. S12 SDS–polyacrylamide gel electrophoresis (SDA-PAGE) of EuryAnc MAT protein purification. 12.5 % SDS–PAGE, Coomassie blue-stained. Lane 1-prestained molecular marker, lane 2- bacterial pellet, lane 3-supernatant, lane 4- flow through, lane 5-wash 1, lane 6- wash 2, lane 7- elution 1, lane 8- elution 2, lane 9- elution 3, lane 10-elution 4, lane 11- elution 5, lane 12-elution 6.

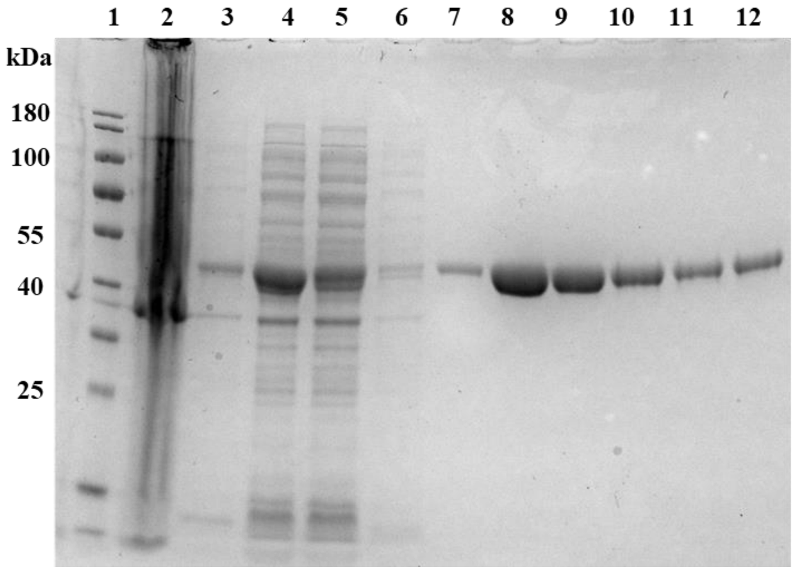

Figure. S13 SDS–PAGE of hMAT1A purification. 12.5% SDS–PAGE, Coomassie blue-stained. Lane 1-prestained molecular marker, lane 2- bacterial pellet, lane 3- flow through, lane 4- supernatant, lane 5-wash 1, lane 6- wash 2, lane 7- elution 1, lane 8- elution 2, lane 9- elution 3, lane 10- elution 4, lane 11- elution 5, lane 12-elution 6.

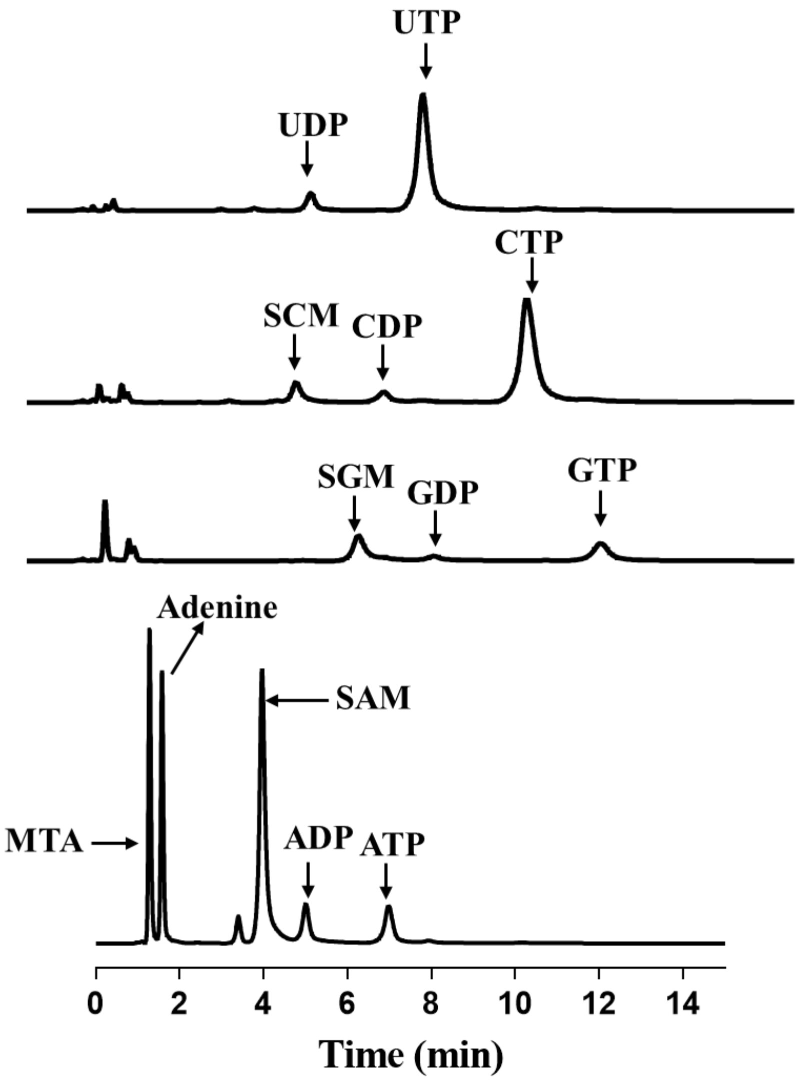

Figure. S14 UPLC chromatogram of the reaction between NTP, methionine, and ArchaeaAnc. Reaction details are in methods sections and UPLC method as mentioned above.

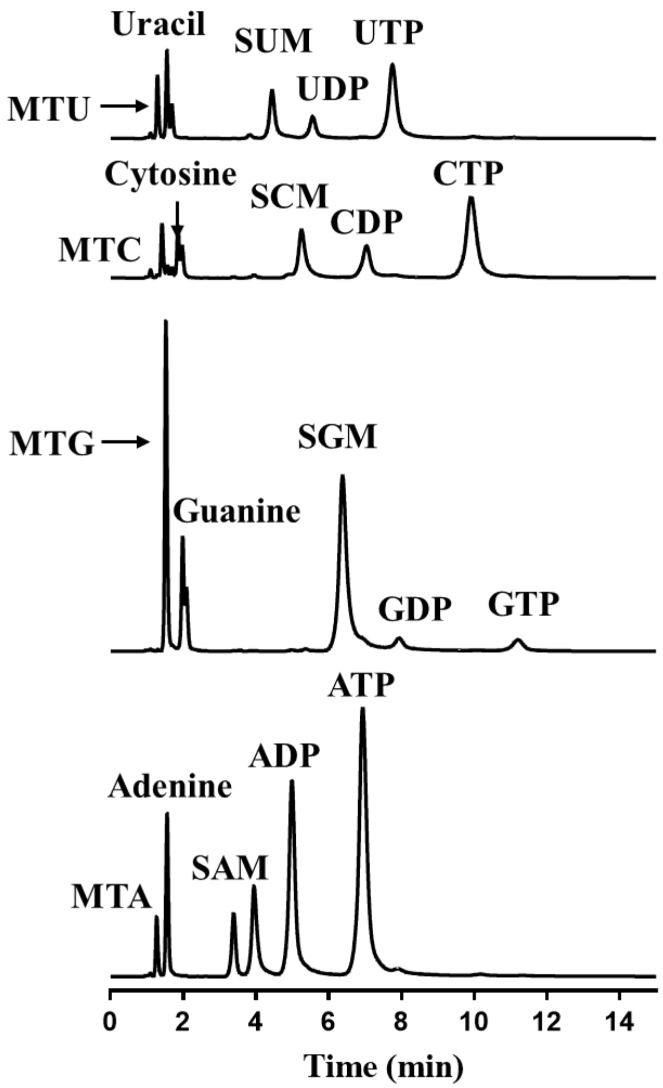

Figure. S15 UPLC chromatogram of the reaction between NTP, methionine, and CrenAnc. Reaction details are in methods sections and UPLC method as mentioned above.

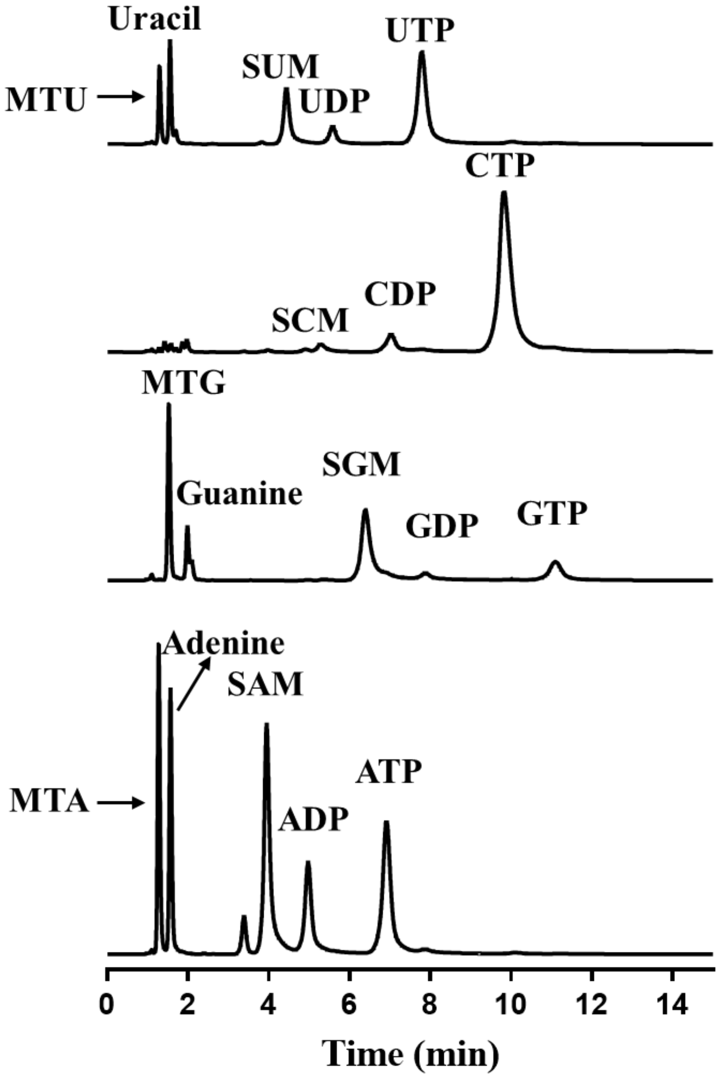

Figure. S16 UPLC chromatogram of the reaction between NTP, methionine, and EuryAnc. Reaction details are in methods sections and UPLC method as mentioned above.

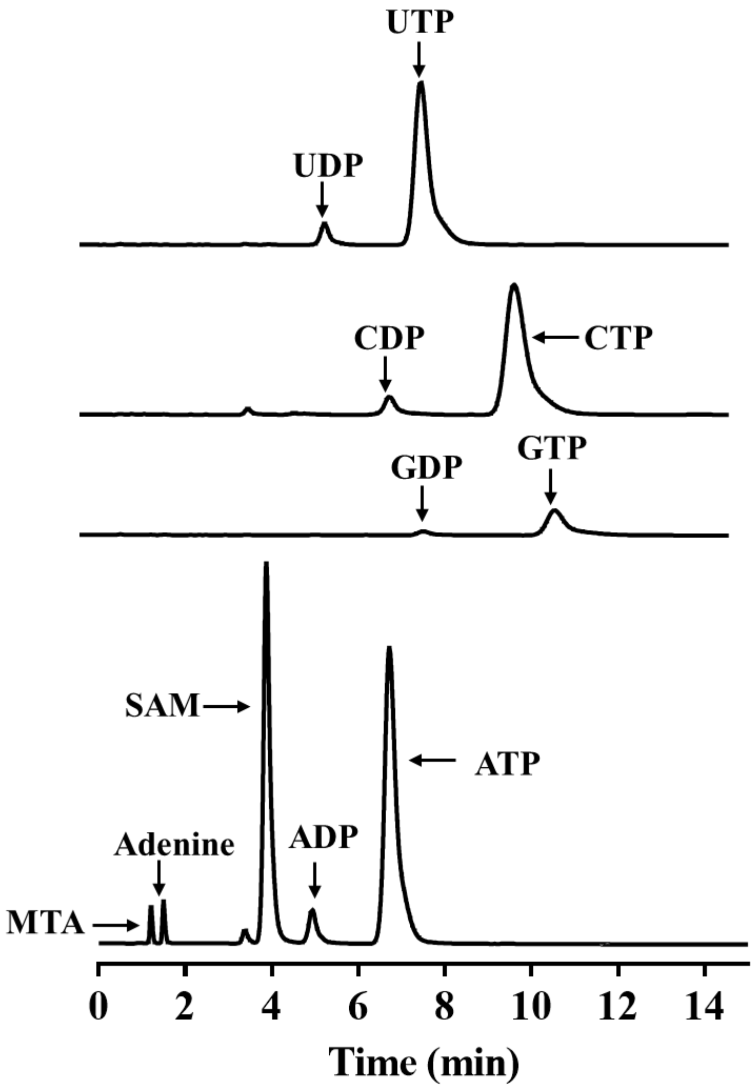
UTP
UDP
CDP
CTP
GDP
GTP
SAM
ATP
Adenine
ADP
MTA
0
2
4
6
8
10
12
14
Time (min)

Figure. S17 UPLC chromatogram of the reaction between NTP, methionine, and hMAT1A. Reaction details are in methods sections and UPLC method as mentioned above.

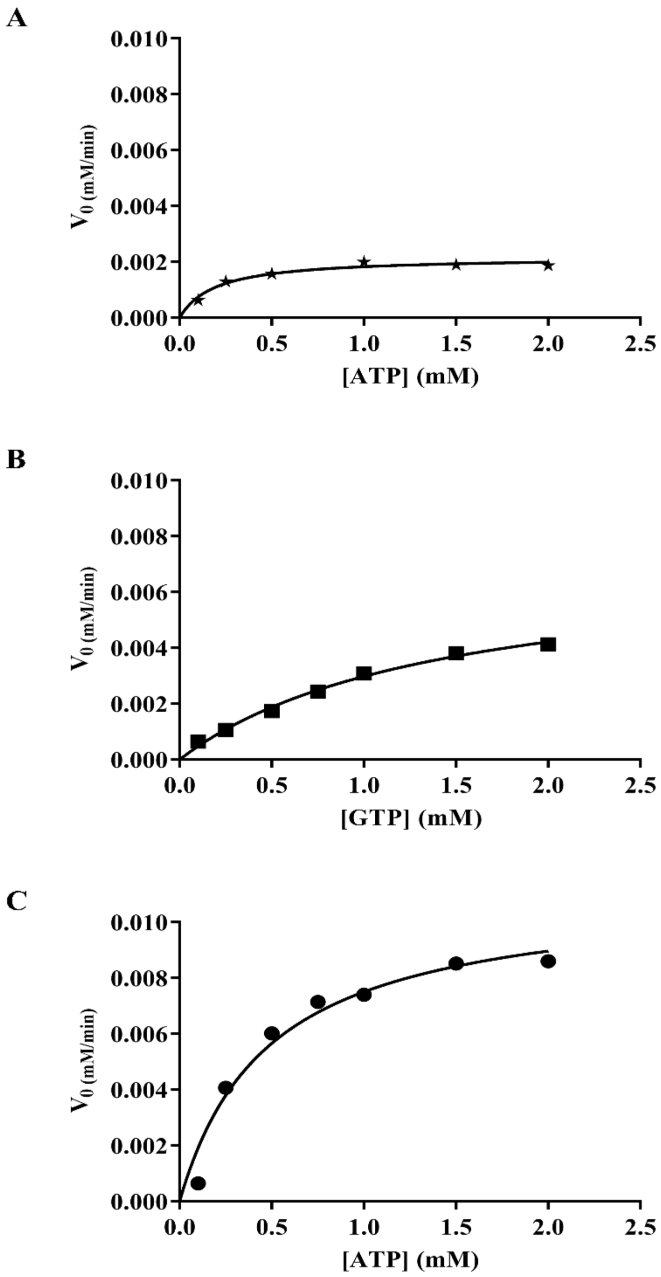

Figure. S18 Kinetic parameters for the SAM and SGM formation by ArchaeaAnc and hMAT1A.

Kinetic parameters for the SAM and SGM analog formation by ArchaeaAnc (0.5 µM) with saturating methionine (10 mM) concentration and ATP (A), GTP (B), in the range of 0.1 to 2 mM in buffer [HEPES (100 mM), KCl (50 mM), $MgCl_2$ (10 mM)], pH 8 at 55℃. Same parameters as of ArchaeaAnc was used for hMAT1A ATP but temperature 37 °C (C). SAM and SGM production were analyzed by UPLC, and data fitted to the Michaelis-Menten equation using GraphPad Prism 7.02. Experiments were performed in duplicates.
