# Supplementary material for: Implications of divergence of methionine adenosyltransferase in archaea
Source: FEBS Open Bio. 2021 Nov 5;12(1):130–45. doi: 10.1002/2211-5463.13312 (PMC8727953; doi:10.1002/2211-5463.13312)
Supplement: Supplementary file 2 — Appendix S1. Bacteria. [file FEB4-12-130-s001.docx]

>Acidobacteria_Geothrix_fermentans

-----M---A-TQ------------GR-H-LFTSESVTEGHPDKMADQISDAVLDAALKDDP-----RSRVACETLLTTGLVLVAGEITTET-YI--------PVAALVRDVVKDIGYDH--HI-KGFDYATCAVMVTIDQQSPDIAMGV-DT-G-------------------------GAGDQGLMFGYACRETPELMPAPVHYAHLLTRKLAEVRKNG--Q-LPWLRPDGKSQVTVEFD-G-D-----Q-VKRIHTV-VISTQH-DEHVT---------QN----SIRD-AVIQDVIKAS-----L-P-Q-------D-L-LDS--KT--IFHVNPTGRFVVGGPMGDTGLTGRKIIVDTYGGSGHHGGGAFSGKDPSKVDRSAAYMGRYIAKNIVAAGLADRCEIQLAYAIGVAEPVSIAVDTFGTGK--VSDE----AIVRAVREIFSCTPKAMIEALDLRR-P-------IYRATSAYGHFGR-P----------------------E-----F--S-WEKT-DK-V-----EALR----A----AAK-----------------

>Acidobacteria_Holophaga_foetida

-----M---T-QV------------GR-H-LFTSESVTEGHPDKIADQISDAVLDAALTGDP-----ASRVACETLVTTGLILVAGEITTDC-YI--------DIPRIARETVREIGYDH--SV-KGFDCDTCGVMVTLDQQSADIAMGV-DT-G-------------------------GAGDQGLMFGYACDETPELMPAPIHFAHALTQRLSEVRKSG--Q-LPWLRPDGKSQVTVEYD-G-D-----G-VARIHTV-VISTQH-DDHIS---------ND----HIRE-SVIREVIKAS-----L-P-E-------E-L-LDH--DT--IYHVNPTGRFVIGGPMGDTGLTGRKIIVDTYGGSGHHGGGAFSGKDPSKVDRSAAYFGRYIAKNIVAAGLARKAEIQLAYAIGVAEPVSIAVDSFGTGA--VSDE----AIAKAVREVFSCTPKAMIEALDLRR-P-------IYRPTAAYGHFGR-D----------------------S-----F--S-WERT-DR-A-----EALK----A----AAK-----------------

>Acidobacteria_Chloracidobacterium_thermophilum

---------M-NN------------GN-F-LFSSESVTEGHPDKMADQISDAILDEVLRRDP-----QGRVACETLLATGLVVIAGEITTTA-HV--------DFPTVAREVVRRIGYDN--AE-YGFDAKTCSVISTINAQSPDIAMGV-DT-G-------------------------GAGDQGLMFGFACTETPELMPLPIQLAHRLTKRLAQVRRDG--T-LEYLRPDGKAQVSVEY--R-D--G--K-PHRVAAV-VVSTQH-AESVK---------SD----ELRR-DIERHVIRHV-----I-T-N-------G-M-LDA--DT--KLHINPTGRFVIGGPMGDTGLTGRKIIVDTYGGYAPHGGGAFSGKDPTKVDRSAAYMARYIAKNVVAAGLADRCQVQLAYAIGVAEPVSIYVETFGTGK--IRPE----TIVELIRAHFSLTPRGIIETLDLRR-P-------IYSPTAAYGHFGREN----------------------EES---F--T-WERT-DK-A-----EVLR----K----EAGL----------------

>Acidobacteria_Pyrinomonas_methylaliphatogenes

-----------MR------------AR-R-LFTSESVTEGHPDKIADQISDAVLDAVLSEDP-----EGRVACETLVTTGLVVIAGEITTQA-RI--------DFARIARETIREIGYTR--AK-YGFDCDTCSVITTIDRQSPDIAMGV-DT-G-------------------------GAGDQGLMFGFACDETPELMPLPIQLAHKLAARLAEARKTG--E-LAFLRPDGKSQVTVEY--R-D--G--R-PHRIETV-VISTQH-DPQIP---------QE----QLRA-EIIEKVIRRT-----V-P-P-------E-L-LDR--ET--KYYVNPTGRFVIGGPQGDAGLTGRKIIVDTYGGYAPHGGGAFSGKDPTKVDRSAAYMARYIAKNIVAAGLASRCLIQLAYAIGVADPVSVLVDTQGTGL--ISDE----RLSEIVREHFRLTPREIIEELDLRR-P-------IYRRTASYGHFGR-N----------------------E-----F--S-WERT-DR-A-----TALR----S----AAGI----------------

>Acidobacteria_Bryobacter_aggregatus

-----M---A-EA------------IK-R-IFTSESVTEGHPDKMADQISDAVLDAVLAEDP-----TGRVACEVLVTTGICVVAGEITTTT-YV--------DVPDLARKVIKEIGFDD--AA-FGYDGNTCGVLNVIQRQSPHIAMGV-DT-G-------------------------GAGDQGLMFGYACNETPELMPLPIILSHKLVRKLSEVRRAG--T-LSYLRPDGKSQVSVEYN-G-D-----Q-PVRIDAV-VISTQH-GDDVS---------TE----QLRA-DVKKLIIDAV-----L-P-A-------D-M-VDG--DT--KIHINPTGRFVIGGPNGDTGLTGRKIIVDTYGGMGRHGGGAFSGKDPTKVDRSACYMARYIAKNIVAAGLADRCEVQLAYAIGVAEPVSVLVDTFGTGT--VSSE----KLTEIVRANFKLTPKGIIESLDLRR-P-------IYRDTAAFGHFGR-T----------------------EDT---F--T-WEKT-DK-A-----AALK----A----AAV-----------------

>Acidobacteria_Sulfotelmatomonas_gaucii

-----M---K-VS------------DR-F-LFTSESVTEGHPDKIADQVSDAILDACLDQDP-----YSRVAAETLTATGLVVIAGEITTKA-YV--------DFQSLVRGVVASIGYDN--AL-YGFDSNTCAVISSINKQSGDIAQGV-DT-G-------------------------GAGDQGMMFGYASNETPELMPAPISLAHKLCRQLSHVRKSG--K-LPYLRPDGKSQVTVEYG-E-D--G--K-PARIDAV-VISSQH-SEAVS---------IE----DLHA-DVLKHVIQAV-----L-P-A-------A-W-LDE--HT--KYHINPTGRFVIGGPMGDTGLTGRKIIVDTYGGMGRHGGGAFSGKDPTKVDRSAAYMARHIAKNIVAAGLAEKAEVQLAYAIGVAEPVSVLVETFGTGK--LSEA----KLTELIRKNFSLTPKSIIEYLNLRR-P-------IFQKTAAYGHFGR-N----------------------EPE---F--T-WEST-DK-A-----AKLA----E----QAGVSVAVK-AR--------

>Acidobacteria_Koribacter_versatilis

-----M---S-TR------------NR-F-LFTSESVTEGHPDKIADQISDAILDACLKDDP-----TSRVACETLTATGLVVIAGEITTKA-YV--------DFQTLVRGVVQTIGYDN--AL-YGFDSNTCAVISSINKQSGDIAMGV-DT-G-------------------------GAGDQGMMFGYATNETPELMPAPISLAHKLTLKLTEVRKNG--T-LPYLRPDGKSQVTVEYD-Q-H--S--K-PVRVDAV-VVSTQH-AETVS---------TE----DLRA-DILKHVIQAA-----V-P-A-------N-L-LDA--DT--KYHINPTGRFVIGGPMGDTGLTGRKIIVDTYGGMGRHGGGAFSGKDPTKVDRSAAYMARYIAKNIVAAGLADRCEVELAYAIGVAEPVSVLVETFGTGK--VEEA----KIEELVRKNFKLTPKGIMESLNLRR-P-------IYRKTAAYGHFGR-N----------------------D-K---F--T-WEAT-DK-A-----EALR----E----QAGLKSAAH-AGKN------

>Acidobacteria_Sulfotelmatobacter_kueseliae

-----M---A-AK------------NR-Y-LFTSESVTEGHPDKMADQISDAILDACLTDDA-----SSRVACETLTATGLVVIAGEITTKA-YV--------DFQTVVRGTIASIGYDN--AL-YGFDANTCAVISSINKQSGDIAMGV-DI-G-------------------------GAGDQGMMFGYAVNENVDLMPTPISLAHKLTQQLSLVRKNG--T-LPFLRPDGKSQVTVEYD-A-N--G--K-VQRVDAV-VISSQH-SETIS---------ND----ELRA-GILKHVIQAV-----I-P-A-------H-L-LDE--YT--HYHINPTGRFVIGGPMGDTGLTGRKIIVDTYGGMGRHGGGAFSGKDPTKVDRSAAYMARHIAKNVVAGGLADRCEVQLAYAIGVADPVSVLVETFGTEK--VDPA----VIPELIRAHFKLTPRGIIESLNLRR-P-------IYRKTAAYGHFGR-S----------------------D-P---F--T-WEAT-DK-A-----AKLA----S----DAGLREPAT-QVVRK-----

>Actinobacteria_Micrococcus_lylae

---------M-T-------------DR-K-LFTSESVTEGHPDKIADQISDAILDEILKGDP-----KARVACETSVTTGMALIAGEITTST-YV--------DIPKVVRETVKEIGYTR--AK-FGYDFQTMAVLTAIDEQSPDIAQGV-DK-ALEDR----D-TLT-D--SDIE-SI-GAGDQGLMFGFACNETESLMPLPISLSHELARRLTEVRKDG--T-LDYLRPDGKTQVTVEYD-E-A--G--K-PKRIDTI-VISSQH-HEKIE---------LE----QIQT-DIKEHVIKPV-----V-P-A-------N-L-ID--DET--KYFINPTGRFVIGGPQGDAGLTGRKIIVDTYGGYARHGGGAFSGKDATKVDRSGAYAARYVAKNIVAAGLADKCEVQLAYAIGVAQPVSISVETYGTSD--YTEQ----QLVEAVRKTFDLRPAGIIQMLDLRR-P-------IYKQTAVYGHFGR-T----------------------DVD---L--P-WEAT-DR-V-----DTLK----A----NLS-----------------

>Actinobacteria_Kitasatospora_mediocidica

---------M-S--------------R-R-LFTSESVTEGHPDKIADQISDTILDALLSEDP-----TSRVAVETLITTGLVHIAGEVTTKA-YA--------PIAQLVRQKILEIGYDS--SK-KGFDGASCGVSVSIGAQSPDIAQGV-DT-AYESR-V----EGDD---DLLD-RQ-GAGDQGLMFGYASDETPELMPLPITLAHRLSRRLSEVRKNG--T-IPYLRPDGKTQVTIEY----D--G-DK-AVRLDTV-VVSSQH-ASDID---------LD---SLLTP-DIRQFVVEPE-----L-A-D------LA---IDT-SDY--RLLVNPTGRFEVGGPMGDAGLTGRKIIIDTYGGMARHGGGAFSGKDPSKVDRSAAYAMRWVAKNIVAAGLAHRAEVQVAYAIGKAEPVGLFVETFGTES--VPVL----DIQKAVTEVFDLRPAAIIRDLDLLR-P-------IYAKTAAYGHFGR-E----------------------DAD---F--T-WERT-DR-A-----EQLK----K----AAGL----------------

>Actinobacteria_Jatrophihabitans_endophyticus

---------M-S--------------R-R-LFTSESVTEGHPDKIADQISDSILDALLAEDP-----ASRVAVETLIMTGQVHVAGEVTTSA-WA--------DIPKLVRDRIIEIGYDS--SR-KGFDGESCGVSISIGAQSADIAQGV-DT-AYEKR-V----EASE---DEIA-SQ-GAGDQGLMFGYASDETPELMPLPIWLAHRLSERLTEVRKNG--T-VPYLRPDGKTQVTIEY----I--D-GK-PARLDTV-VLSTQH-ADDID---------LD---TLLKP-DIEEHVIRPI-----V-S-A------LD---IDT-ENY--RLLVNPTGRFVIGGPMGDAGLTGRKIIVDTYGGMARHGGGAFSGKDPSKVDRSAAYAMRWVAKNVVAAKLASRCEVQVAYAIGKAEPVGLFIETFGTET--VAPE----KIAEAITQTFDLRPAAIIRDLDLKR-P-------IYAKTAAYGHFGR-E----------------------DAD---F--T-WERT-DR-A-----EQLA----K----LANG----------------

>Actinobacteria_Herbidospora_mongoliensis

---------M-S--------------R-R-LFTSESVTEGHPDKIADQISDAILDAMLKDDP-----KSRVAVETMITTGQVHVAGEVTTET-YV--------DIPGVIREKILEIGYDA--SH-KGFDGASCGVSVSIGAQSPDIAQGV-DD-AYEHR-E----ESLG---DDLD-RQ-GAGDQGLMFGYACRETPELMPLPITLAHRMARRLSEVRKNG--T-IPYLRPDGKTQVTIEY----D--G-DK-PVRLDTV-VVSTQH-APEID---------LK---EMLAP-DIREHVVEPV-----L-A-D------LD---IVV-EGY--RLLVNPTGRFEIGGPMGDAGLTGRKIIVDTYGGMARHGGGAFSGKDPSKVDRSAAYAMRWVAKNIVAAGLADRAEVQVAYAIGKAQPVGVFVETFGTET--VEIS----AIQSAVLQVFDLRPAAIIRDLDLLR-P-------IYSETAAYGHFGR-------------------------DG---F--S-WEST-DR-A-----GALR----A----AAGL----------------

>Actinobacteria_Planomonospora_sphaerica

---------M-S--------------R-R-LFTSESVTEGHPDKIADQISDAILDAMLKGDP-----KSRVAVETLITTGQVHVAGEVTTET-YV--------DIPGLIREKILEIGYDA--SH-KGFDGASCGVSVSIGAQSPDIAQGV-DA-AYEAR-E----GEAG---DEFD-RQ-GAGDQGLMFGYACRETPELMPLPIQLAHRLAERLSQVRKDG--T-VPYLRPDGKTQVTIEY----D--G-DK-PVRLDTV-VVSTQH-AAEID---------LK---EMLAP-DIKEHVVDPV-----L-A-G------LE---LET-EGY--RLLVNPTGRFEIGGPMGDAGLTGRKIIVDTYGGMARHGGGAFSGKDPSKVDRSAAYAMRWVAKNIVAAGLADRAEVQVAYAIGKAHPVGVFVETFGTEK--TDIA----KIQAAVLQVFDLRPAAIIRDLDLLR-P-------IYSETSAYGHFGR-------------------------PE---F--S-WEAT-DR-A-----DALR----A----AAGL----------------

>Actinobacteria_Murinocardiopsis_flavida

---------M-S--------------R-R-LFTSESVTEGHPDKMADQISDAVLDAMLKDDP-----KSRVAVETLITTGQVHVAGEVTTQT-YV--------DIPSLIRAKILEIGYDS--SA-KGFDGGSCGVSVSIGAQSPDIAQGV-DT-AYETR-A----EAGT---DDLD-RQ-GAGDQGLMFGYANRETPELMPLPIKLAHALSQRLSEVRRDG--T-VPYLRPDGKTQVTVEY----E--N-NT-PVRLDTV-VVSSQH-APDID---------LL---ELLTP-DVKEHVIAPV-----V-A-S------YG---LES-DNY--RLLVNPTGRFEIGGPMGDAGLTGRKIIVDTYGGYARHGGGAFSGKDPSKVDRSAAYATRWVAKNIVAAGLADRCEVQVAYAIGKAHPVGVFIETFGTEA--VAPE----NIEKAVQDVFDLRPAAIIRDLDLLR-P-------IYSLTSAFGHFGR-E----------------------IPE---F--T-WEQT-DR-A-----AALK----A----AVGA----------------

>Actinobacteria_Micromonospora_globosa

---------M-T--------------R-R-LFTSESVTEGHPDKIADQISDAVLDALLAQDP-----QSRVAVETLITTGQVHVAGEVTTRA-YA--------DIPQIVRDTILNIGYDS--SR-KGFDGLSCGVNLSIGAQSPDIARGV-DR-AIERV-A----GGSV---HALD-TQ-GAGDQGMMFGYACSETPELMPLPIALAHRLARRLAAARKDR--T-IPYLRPDGKTQVTIEY----D--G-SR-PARLHTV-VVSSQH-AADIS---------LD---SLLAP-DVREHVIEPE-----L-T-G------LG---VDV-EDY--RLLVNPTGRFEIGGPMGDAGLTGRKIIVDTYGGYARHGGGAFSGKDPSKVDRSAAYAMRWVAKNVVAAGLAERCETQVAYAIGKARPVSFLVDTFGTEN--VPTH----RIERAIDEVFDLRPAAIIRDLRLLR-P-------IYRQTAVYGHFGR-E----------------------LPD---L--L-WENT-DR-A-----QDLK----D----AAA-----------------

>Actinobacteria_Actinoplanes_italicus

---------M-A--------------R-R-LFTSESVTEGHPDKIADQISDGILDALLAQDP-----RSRVAVETLITTGQVHVAGEVTTQA-YA--------DIPKIVRDTILAIGYDS--SK-KGFDGASCGVSVSIGSQSPDIAQGV-DS-AIELR-E----GDSE---HVLD-SQ-GAGDQGMMFGFACSETPELMPLPIALAHRLARRLSAARKDG--T-IPYLRPDGKTQVTIEY----D--G-LR-PVRLDTV-VVSSQH-AADIS---------LE---SLLTP-DVREHVIAPE-----L-E-G------LG---LDA-EGY--RLLVNPTGRFEIGGPMGDAGLTGRKIIVDTYGGYARHGGGAFSGKDPSKVDRSAAYATRWVAKNVVAAGLAERCEVQVAYAIGKAHPVSLFVETFGTEN--VPVE----RIEKAIKDVFDLRPAAIIRDLDLLR-P-------IYQQTAAYGHFGR-E----------------------LPD---L--K-WEST-DR-A-----QDLK----N----AAS-----------------

>Actinobacteria_Actinoplanes_brasiliensis

---------M-A--------------R-R-LFTSESVTEGHPDKIADQISDGILDALLAQDP-----RSRVAVETLITTGQVHVAGEVTTQA-YA--------DIPSIVRDTILRIGYDS--SK-KGFDGASCGVSVSIGSQSPDIAQGV-DS-ALELR-E----GDSE---HVLD-AQ-GAGDQGMMFGFACSETPELMPLPIALAHRLARRLSAARKDG--T-IPYLRPDGKTQVTIEY----D--G-LK-PVRLDTV-VVSSQH-AADIS---------LE---SLLTP-DIREHVIAPE-----L-E-G------LG---LDT-DNY--RLLVNPTGRFEIGGPMGDAGLTGRKIIVDTYGGYARHGGGAFSGKDPSKVDRSAAYAMRWVAKNVVAAGLAERCETQVAYAIGKAHPVSLFVETFGTEN--VPVE----RIEKAINEVFDLRPAAIIRDLDLMR-P-------IYQQTAAYGHFGR-E----------------------IPE---L--L-WENT-ER-A-----QDLK----N----AAA-----------------

>Actinobacteria_Asanoa_ferruginea

---------M-A--------------R-R-LFTSESVTEGHPDKIADQISDGILDALLAQDP-----RSRVAVETLITTGQVHVAGEVTTQA-YA--------DIPTIVRETILGIGYDS--SK-KGFDGASCGVSISIGSQSPDIAQGV-DT-AIELR-D----GESAG--DALD-RQ-GAGDQGMMFGFACSETPELMPLPIALAHRLARRLSAVRKDG--T-VPYLRPDGKTQVTIEY----D--G-LR-PVRLDTV-VVSSQH-AADIS---------LE---SLLTP-DVREHVIAPE-----L-D-A------LG---LDT-EGY--RLLVNPTGRFEIGGPMGDAGLTGRKIIVDTYGGYARHGGGAFSGKDPSKVDRSAAYAMRWVAKNVVAAGLAERCEAQVAYAIGKAHPVSLFVETFGTEN--VPVD----RIEKAIGEVFDLRPAAIIRDLDLLR-P-------IYQQTAAYGHFGR-E----------------------LPD---L--T-WENT-DR-A-----ADLK----N----AAGL----------------

>Actinobacteria_Xiangella_phaseoli

---------M-T--------------R-R-LFTSESVTEGHPDKIADQISDGILDALLTQDP-----HSRVAVETLITTGQVHVAGEVTTKA-YA--------DIPTIVRETILDIGYDS--SK-KGFDGASCGVSVSIGSQSPDIAQGV-DN-AFELR-T----GASE---SALD-AQ-GAGDQGMMFGFACSETPELMPLPIALAHRLARRLSAVRKDG--T-VPYLRPDGKTQVTIEY----D--G-LR-PVRLNTV-VVSSQH-AADIS---------LD---SLLTP-DVRDHVIAPE-----L-E-S------LG---LDT-EGY--RLLVNPTGRFEIGGPMGDAGLTGRKIIVDTYGGYARHGGGAFSGKDPSKVDRSAAYAMRWVAKNVVAAGLAERCEVQVAYAIGKAHPVSLFVETFGTET--VPVA----SIEKAVTEIFDLRPAAIIRDLHLLR-P-------IYGQTAAYGHFGR-E----------------------LPD---L--T-WENT-DR-A-----ADLK----S----AAGA----------------

>Actinobacteria_Glycomyces_sambucus

---------M-A--------------R-R-LFTSESVTEGHPDKIADQISDGILDALLSEDP-----GSRVAVETLITTGQVHVAGEVTTHA-YA--------DIPRIVRETILRIGYDS--SK-KGFDGASCGVNVSIGGQSPDIAQGV-DK-AWESR-V----DAAVN--DELD-LQ-GAGDQGLMFGFACRETPELMPLPIALAHRLSQRLTEVRKDG--T-VPYLRPDGKTQVTIEY----Q--G-NR-PVRLDTV-VVSSQH-APDIS---------LD---SLLSP-DVAEHVIAPV-----V-S-G------LD---LDI-ADY--KLLVNPTGRFEIGGPMGDAGLTGRKIIVDTYGGYARHGGGAFSGKDPSKVDRSAAYAARWVAKNVVAAGLAERCEVQVAYAIGKAKPVSIAVDPMGTET--VDPA----LIEKAVLEVFDLRPAAIIRDLNLIR-P-------IYSLTAAYGHFGR-E----------------------LPE---F--T-WEAT-DR-A-----AALK----E----AVS-----------------

>Actinobacteria_Kineococcus_xinjiangensis

---MTSTSPT-S--------------E-R-LFTSESVTEGHPDKICDQISDGILDELLRQDP-----KSRVAVETMVTTGLVHVAGEVTTEA-YA--------DIPTIVRQTLLGIGYDS--SA-KGFDGRTCGVEVSIGSQSPDIAQGV-DE-AWENR-Q----DGAGS--DPLD-MQ-GAGDQGLMFGYACDDTPELMPLPIWLAHRLSQRLSEARKKG--E-IPYLRPDGKTQVTIGY----D--G-DR-PVRLDTV-VLSTQH-EPNVD---------LV---TGLTP-DVRRLVVEPV-----L-E-D------LA---IDT-SDI--RLLVNPTGRFEIGGPMGDAGLTGRKIIVDTYGGMARHGGGAFSGKDPSKVDRSAAYAMRWVAKNVVAAGLARRCEVQIAYAIGKAHPVGLYVETFGTEV--VPLA----AVGRAVREVFDLRPAAIVRDLDLLR-P-------IYAQTSAYGHFGR-S----------------------LPD---F--T-WERT-DR-V-----DALR----E----VARL----------------

>Actinobacteria_Jiangella_gansuensis

---------M-S--------------L-R-LFTSESVTEGHPDKICDQISDAVLDALLKDDP-----GSRVAVESMVTTGLVHVAGEVTTRG-YA--------DIPGIVRETILGIGYDS--ST-KGFDGYSCGVSVSIGSQSRDIAQGV-DT-AYEHR-V----EG-DL--DPLD-LQ-GAGDQGLMFGYACDETPELMPLPILLAHRLAQQLSQARRSG--A-VPYLRPDGKTQVTIEY----D--G-DR-PVRLDTV-VVSTQH-AANID---------LA---ELLTP-DIATQVVTPV-----L-E-T------LE---LEH-HDY--RLLVNPTGRFEIGGPMGDAGLTGRKIIIDTYGGMARHGGGAFSGKDPSKVDRSAAYAMRWVAKNIVAAGLARRCEVQVAYAIGKAEPVGLFLECFGTER--VPVE----RIERAVTEVFDLRPAAIIRDLDLLR-P-------IYAQTAAYGHFGR-E----------------------LPD---F--T-WERT-DR-V-----EALQ----A----AAG-----------------

>Actinobacteria_Kribbella_antibiotica

---------M-A--------------R-R-LFTSESVTEGHPDKIADQISDSILDALLAADP-----KSRVAVETLVTTGLVVVAGEVTTSA-YV--------DIPGIVRKRILEIGYDS--SL-KGFDGASCGVSIAIGSQSADIAQGV-DT-AYETR-S----DASK---DELD-LQ-GAGDQGLMFGYASNETPELMPLPITIAHRLSARLTEVRKDG--T-LAYLRPDGKTQVTVEY----D--G-DK-AVRIDTV-VVSSQH-AADIN---------LE---SMLAP-DVKKHVVDPV-----L-E-Q------FD---IDA-TDY--KLLVNPTGRFEVGGPMGDAGLTGRKIIIDTYGGMARHGGGAFSGKDPSKVDRSAAYAMRWVAKNIVAAGLADRVECQVAYAIGKAAPVGFYVDTFGTEK--IPVN----EISDAVAEVFDLRPAAIIRDLDLLR-P-------IYTQTAANGHFGR-T----------------------GED---F--T-WERT-DR-V-----EALK----A----AVNK----------------

>Actinobacteria_Friedmanniella_sagamiharensis

---------M-T--------------G-R-LFTSESVTEGHPDKIADQISDSVLDALLAEDP-----HSRVAVETLVTTGLVVVAGEVTTDA-YV--------EIPSIVRSRILEIGYDS--ST-KGFDGASCGVQVAIGQQSPDIAQGV-DT-AYERR-T----GESV---DDLD-AQ-GAGDQGLMFGYASDETANLMPLPIDLAHRLAEQLSEVRKDG--R-VPYLRPDGKTQVTIEY----D--G-DR-PVRLDTV-VLSSQH-AADID---------LD---ALLTP-DVRKHVVEPV-----L-E-R------FE---IDT-RGY--RLLVNPTGRFEVGGPMGDAGLTGRKIIVDTYGGMARHGGGAFSGKDPSKVDRSAAYAMRWVAKNVVAAGLAHRCEVQVAYAIGRAHPVGFYVETFGTEQ--VPVS----RIQEAVLAVFDLRPAAIIRDLDLLR-P-------VYAPTARYGHFGR-E----------------------LPG---S--T-WERT-DR-A-----DALA----A----AVRG----------------

>Actinobacteria_Planktophila_lacus

---------M-S--------------K-R-LFTSESVTEGHPDKIADQISDAILDSLLSQDP-----SSRVAVETLITTGQVHVAGEVTTNG-YA--------DVMGIVRDTVIGIGYDS--SV-KGFDGNSCGVSISIGQQSQDIAQGV-DD-AYENR-V----ASSA---DPLD-LQ-GAGDQGLMFGYACDDTKELMPLPIWLAHELAQQLSKVRKSG--A-LGYLRPDGKTQVTIEY----D--G-DK-AVALDTV-VISSQH-AEEVD---------VA---KKLTP-EIIEHVIEPV-----L-S-K------ID---LPR-KDM--RTLINPTGRFVIGGPMGDAGLTGRKIIVDTYGGMARHGGGAFSGKDPSKVDRSAAYAMRWVAKNVVAAGLARRCEVQVAYAIGKAQPVGVFVETFGTET--VPVQ----KIQDAVTTVFDLRPAAIIRDLNLLR-P-------IYSQTAAYGHFGR-E----------------------LPN---F--T-WEST-SR-V-----DALR----A----AVN-----------------

>Actinobacteria_Gordonia_bronchialis

---MTT---S-A--------------S-R-LFTSESVTEGHPDKICDAISDSVLDAILTEDP-----KARVAVETLVTTGQVHVAGEVTTTA-YV--------DIPGIVRQKILEIGYDS--ST-KGFDGASCGVNVAIGAQSPDIAGGV-FN-SHESR-S----GISE---DEID-SQ-GAGDQGLMFGFATNETPELMPLPIALAHRLSRKLTEVRKSG--V-LPYLRPDGKTQVTIEY----D--G-DK-PVRLDTV-VLSTQH-AADID---------LE---NMLTP-DIRTEVVEAV-----L-A-D------LEVATLDT-SGY--RLLVNPTGKFVLGGPMGDAGLTGRKIIVDTYGGMARHGGGAFSGKDPSKVDRSAAYAMRWVAKNAVAAGLADRIEVQVAYAIGKAAPVGLFVETFGTEK--VDPA----VIERVISENFDLRPLAIIRDLDLLR-P-------IYAPTAAYGHFGR-T----------------------DID---L--P-WERT-DR-A-----EKLR----A----AAGL----------------

>Actinobacteria_Williamsia_limnetica

-----M---T-D--------------S-R-LFTSESVTEGHPDKICDAISDSILDALLTDDP-----RARVAVETLVTTGQVHVAGEVNTTA-WA--------DIPKIVREKVLEIGYDS--SA-KGFDGNSCGVNIAIGAQSPEIAQGV-DN-AYESR-V--E-GITE---DEIA-RQ-GAGDQGLMFGYATNETPEYMPVPIALAHRLARRLTEVRKTG--V-LPYLRPDGKTQVTIEY----I--D-DK-PVRLDTV-VVSTQH-AADID---------LE---NLLTP-DIRNQVLESV-----F-N-D------LNLPTLDT-NDV--RLLVNPTGKFVLGGPMGDAGLTGRKIIVDTYGGMARHGGGAFSGKDPSKVDRSAAYAMRWVAKTAIAAGLADRIEVQVAYAIGKAAPVGLFVETFGTEH--VDRA----KIQSAITEVFDLRPGAIIRDLDLLR-P-------IYAPTAAYGHFGR-T----------------------DID---L--P-WEHT-DR-A-----EKLK----A----AAGL----------------

>Actinobacteria_Rhodococcus_jostii

---MSQ---S-G--------------S-R-LFTSESVTEGHPDKICDAISDSILDALLTDDP-----RARVAVETLVTTGQVHVAGEVTTSA-YA--------DIPKIVRDTVLEIGYDS--SA-KGFDGNSCGVNVAIGAQSPEIAQGV-DH-SHEVR-T--G-ELSD---DEID-RQ-GAGDQGLMFGFATTDTPELMPLPIALAHRLSRRLTEVRKSG--V-LPYLRPDGKTQVTIEY----D--G-DK-AVRLDTV-VISTQH-AADID---------LD---NLLTP-DLREKVLGSV-----L-A-E------IDMPELDV-SDI--RLLVNPTGKFVLGGPMGDAGLTGRKIIVDTYGGMARHGGGAFSGKDPSKVDRSAAYAMRWVAKNAVAAGLADRIEVQVAYAIGKAAPVGLFVETFGTEK--TDPA----RIQQAITETFDLRPGAIIRDLDLLR-P-------IYAQTAAYGHFGR-T----------------------DID---L--P-WESI-DR-A-----EKLR----A----AAGL----------------

>Actinobacteria_Saccharopolyspora_shandongensis

---MSE---I-N--------------R-R-LFTSESVTEGHPDKICDAISDAVLDALLSQDP-----RSRVAVETLVTTGQVHVAGEVTTDA-YA--------DIPTIVREKILEIGYDS--SA-KGFDGASCGVNVAIGSQSPDIAQGV-DV-AHESR-V----EGVI---DEIA-KQ-GAGDQGLMFGYACTDTDELMPLPIALAHRLSRRLTKVRKDG--V-LPYLRPDGKTQVTIEY----A--G-DQ-PVRLDTV-VLSTQH-AADID---------LE---GMLAG-DIKEKVIDPE-----I-E-R------VG---LDS-ADT--RLLVNPTGRFVVGGPMGDAGLTGRKIIVDTYGGMARHGGGAFSGKDPSKVDRSAAYAMRWVAKNAIAAGLAGRIEVQVAYAIGKAAPVGLFVETFGTEN--VDPV----KIQAAINEVFDLRPAAIVRDLDLLR-P-------IYAQTAAYGHFGR-S----------------------DVD---L--P-WERT-DR-A-----EALK----S----AAGI----------------

>Actinobacteria_Lechevalieria_xinjiangensis

------------------------------MFTSESVTEGHPDKICDAISDSILDALLAKDP-----RSRVAVETLITTGQVHVAGEVTTEA-YA--------DIPTIVREKILEIGYDA--SQ-KGFDGNSCGVNVAIGAQSPDIAQGV-DT-ALEER-V----EGAA---DEID-KQ-GAGDQGLMFGYACTDTPELMPLPIALAHRLSRRLAAVRKDG--V-LPYLRPDGKTQVTIEY----A--G-DQ-PVRLDTI-VVSTQH-ADGID---------LE---KMLGV-DIRRHVVQPE-----I-D-A------LN---LDT-SDV--RLLTNPTGRFVIGGPMGDAGLTGRKIIVDTYGGMARHGGGAFSGKDPSKVDRSAAYAMRWVAKNTVAAGLASRVEVQVAYAIGKAAPVGLFVETFGTET--VDPQ----KIQQAITEVFDLRPAAIIRDLDLLR-P-------IYAPTAAYGHFGR-T----------------------DVD---L--P-WEST-DR-A-----DALR----S----AAGA----------------

>Actinobacteria_Lentzea_waywayandensis

------------------------------MFTSESVTEGHPDKICDAISDSILDALLAKDP-----RSRVAVETLVTTGQVHVAGEVTTEA-YA--------DIPTIVREKILEIGYDS--SQ-KGFDGNSCGVNVAIGAQSPDIAQGV-DT-AFENR-V----EGAD---DEID-KQ-GAGDQGLMFGYACTDTPELMPLPIALAHRLSRRLTAVRKDG--T-VPYLRPDGKTQVTIEY----A--G-DQ-PVRLDTV-VISTQH-ADGID---------LE---KLLGV-DLREHVVAPE-----I-A-E------LG---LDT-SSV--RLLTNPTGRFVIGGPMGDAGLTGRKIIVDTYGGMARHGGGAFSGKDPSKVDRSAAYAMRWVAKNTVAAGLATRVEVQVAYAIGKAAPVGLFVETFGTET--VDPS----KIQQAISEVFDLRPAAIIRDLDLLR-P-------IYAPTAAYGHFGR-T----------------------DVD---L--P-WEST-DR-A-----DALR----N----AAGA----------------

>Actinobacteria_Kutzneria_buriramensis

---MSQ---H-S--------------R-R-LFTSESVTEGHPDKICDAISDSILDGLLSKDP-----RSRVAVETLITTGQVHVAGEVTTEA-YA--------DIPTIVREKILEIGYDS--SA-KGFDGNSCGVNVAIGSQSPDIAQGV-DT-AYESR-V----ESAE---DEIA-RQ-GAGDQGLMFGYATTDTPELMPLPIALAHRLSRRLTAVRKNG--A-LPYLRPDGKTQVTIEY----A--G-DQ-PVRLDTV-VVSSQH-ADGID---------LE---KMLAV-DVREQVVLPE-----L-A-E------LN---LDT-ESV--RLFVNPTGRFVIGGPMGDAGLTGRKIIVDTYGGMARHGGGAFSGKDPSKVDRSAAYAMRWVAKNVVAAGLATRTEVQVAYAIGKAAPVGLFVETFGTET--VDPA----KIQQAISEVFDLRPAAIIRDLDLLR-P-------IYAPTAAYGHFGR-P----------------------DLD---L--P-WERT-DR-A-----EALK----S----ASGA----------------

>Actinobacteria_Yuhushiella_deserti

------------------------------MFTSESVTEGHPDKICDAISDSILDALLAKDP-----RSRVAVETLITTGQVHVAGEVTTEA-YA--------DIPTIVRDVILRIGYDS--SA-KGFDGNSCGVNVAIGSQSPDIAQGV-DT-AYESR-L----ENAL---DEID-RQ-GAGDQGLMFGYACSDTPELMPLPIALAHRLSQRLTAVRKEG--V-LPYLRPDGKTQVTIEY----A--G-EQ-AVRLDTV-VVSTQH-ADGID---------LD---SMLGV-DVREHVVAPV-----L-G-D------IE---LDD-SDV--RLLVNPTGRFVIGGPMGDAGLTGRKIIVDTYGGMARHGGGAFSGKDPSKVDRSAAYAMRWVAKNVVAAGLATRVEVQVAYAIGKASPVGLFVETFGTET--VDPV----KIQAAITEVFDLRPAAIIRDLDLLR-P-------IYGQTAAYGHFGR-P----------------------ELD---L--P-WERT-DR-V-----DALR----A----AVGA----------------

>Actinobacteria_Prauserella_coralliicola

---MTA---S-N--------------R-R-LFTSESVTEGHPDKICDAISDSILDALLAKDP-----RSRVAVETLITTGQVHVAGEVTTEA-YA--------DIPTIVRERILDIGYDS--SA-KGFDGNSCGVNVAIGSQSPDIAQGV-DT-AYESR-L----ENAL---DDLD-KQ-GAGDQGLMFGYACSDTPELMPLPIALAHRLSQRLAAVRKEG--V-LPYLRPDGKTQVTIEY----A--G-EQ-AVKLDTV-VVSSQH-ADGID---------LD---KMLGV-DVREHVVAPV-----L-A-G------VE---LDS-SDL--RLLVNPTGRFVVGGPMGDAGLTGRKIIVDTYGGMARHGGGAFSGKDPSKVDRSAAYAMRWVAKNVVAAGLAGRVEVQVAYAIGKAAPVGLFVETFGTEH--VDPL----KIQAAITEVFDLRPAAIIRDLDLLR-P-------IYAQTSAYGHFGR-P----------------------ELD---L--P-WERT-DR-A-----DALR----S----AVGA----------------

>Actinobacteria_Thermocrispum_municipale

---MTG---S-Q--------------N-R-LFTSESVTEGHPDKICDAISDAILDALLAKDP-----RSRVAVETLITTGQVHVAGEVTTEA-YA--------DIPTIVRDTILNIGYDS--SA-KGFDGNSCGVNVAIGSQSPDIAQGV-DE-AYEAR-V--GAEIRG---DELD-RQ-GAGDQGLMFGYACSDTPELMPLPIALAHRLARRLATVRKTG--V-LPYLRPDGKTQVTIEY----A--G-DQ-AVRLDTV-VVSTQH-AEGID---------LD---KMLGV-DVREHVIMPE-----V-V-D------LP---IDT-TDF--RLLVNPTGRFVIGGPMGDAGLTGRKIIVDTYGGMARHGGGAFSGKDPSKVDRSAAYAMRWVAKNVVAAGLAGRIEVQVAYAIGKAAPVGLFVETFGTET--VDPA----KIQQAITEVFDLRPGAIIRDLNLLR-P-------MYAPTAAYGHFGR-T----------------------DLD---L--P-WERT-DR-A-----EALK----S----AAGA----------------

>Actinobacteria_Kibdelosporangium_phytohabitans

---------M-H--------------K-H-LFTSESVTEGHPDKICDAVSDEILDALLASDP-----HSRVAVETLVTTGQVHVAGEVTTTA-YV--------DIPAIVRDTILKIGYDS--SA-KGFDGNSCGVNVAIGSQSPDIAQGV-DA-AYETR-M----ENAS---DEID-LQ-GAGDQGLMFGYACTDTPELMPLPIALAHRLAHRLTKVRKDG--V-MPYLRPDGKTQVTIEY----A--R-HK-AVRLDTV-VVSSQH-AEGVD---------LE---RLLGA-DMRGHVLAPE-----I-A-G------LE---LAT-DDV--RLLVNPTGRFVIGGPMGDAGLTGRKIIVDTYGGMARHGGGAFSGKDPSKVDRSAAYAMRWVAKNVVAAGLASRIEVQVAYAIGKAAPVGLFVETFGTEN--VSPE----RIWSAIRDVFDLRPAAIIRDLGLLR-P-------IYAQTAAYGHFGR-P----------------------ELD---L--P-WERT-DR-A-----DDLK----Q----AATN----------------

>Actinobacteria_Actinomycetospora_cinnamomea

------------------------------MFTSESVTEGHPDKICDAVSDGILDALLAQDP-----KSRVAVETMVTTGQVHVAGEVTTNA-QV--------NYVDVVRKTILDIGYDS--SA-KGFDGESCGVSISIGAQSKDIAQGV-DQ-GYESR-V----EGNE---DEIA-KQ-GAGDQGLMFGYADADTPELMPLPIGLAHRLARKLTAVRRDG--T-LPYLRPDGKTQVTIAY----D--G-DK-AVGVETV-VLSTQH-AADID---------LD---ELLTP-DIKSKVIDPV-----L-E-G------ID---LDS-SNT--RVLVNPTGRFVTGGPMGDAGLTGRKIIVDTYGGYARHGGGAFSGKDPSKVDRSAAYAMRWVAKNVVAAGLAKRAEVQIAYAIGKAEPVGVFVETFGTEN--VDPA----KIEKAIHDVFDLRPGAIVRDLQLLR-P-------IYQPTAAYGHFGR-D----------------------DLD---L--P-WERT-DR-A-----DALK----S----IAG-----------------

>Actinobacteria_Ponticoccus_gilvus

---------M-A--------------K-R-LFTSESVTEGHPDKIADQISDTVLDELLRQDP-----QSRVAVETLVTTGLVVVAGEVTTEA-YA--------DIPGLVRNKILDIGYDS--SH-KSFDGGSCGVQVSLGSQSPDIAQGV-DT-AWEKR-L----ESSA---EAYD-LQ-GAGDQGLMFGYACDDTDSLMPLPIDLAHRLAERLSAVRKDG--T-MPYLRPDGKTQVTIEY----D--G-IT-PVRLDTV-VVSCQH-AEDVD---------PE---KMLSE-DIRRHVIQPV-----L-E-A------YD---LAH-DDY--KAFVNPTGKFVIGGPMGDAGLTGRKIIVDTYGGMARHGGGCFSGKDPSKVDRSAAYAMRWVAKNVVAAGLAKRCEVQVAYAIGRAHPVGFYLECFGTET--VPVD----QIADAVLATFDLRPAALVDALDLKR-P-------IYAQTAAYGHFGR-E----------------------LDD---F--T-WERT-DR-A-----DALA----R----AVRG----------------

>Actinobacteria_Naumannella_halotolerans

---------M-S--------------R-R-LFTSESVTEGHPDKIADQISDSVLDELLTHDP-----NSRVAVETLVTTGLVVVAGEVTTEA-YA--------EIPRIVRERILEIGYDS--SH-KSFDGASCGVQVSIGAQSPDIAQGV-DN-AEEAR------DGSV---ESYD-LL-GAGDQGLMFGYACSETPELMPLPITLAHRLSERLAAVRKNG--E-VPYLRPDGKTQVTVEY----D--G-DR-PVRLDTV-VISSQH-AADID---------LA---NLLTP-DLREQVVKPV-----L-A-A------FD---LEH-DDY--TLHVNPTGRFEIGGPMGDAGLTGRKIIVDTYGGMARHGGGAFSGKDPSKVDRSAAYAMRWVAKNVVAAGLAERCEVQIAYAIGRAHPVGFYLDCFGTEK--VPTE----QIEAAVLATFDLRPAAIIEALDLKR-P-------IYAQTAAYGHFGR-E----------------------LPD---F--T-WEQA-SR-A-----EALA----A----AVRG----------------

>Actinobacteria_Dietzia_psychralcaliphila

---MT----Q-A--------------R-R-LFTSESVTEGHPDKICDAISDSILDAMLTQDP-----EARVAVETMVTTGMVHVAGEVKTSG-YV--------EIPQLIREKIVEIGYDS--SE-KGFDGVSCGVSISIGQQSPEIAQGV-DT-SHEAR-T--STDPVE---DAIA-RQ-GAGDQGLMFGYACSDTPELMPLPIALAHRLSRRLTEVRKNG--T-LAYLRPDGKTQVTIEY----D--G-DR-AVRLDTI-VISTQH-APEID---------LE---TTLRD-DLTEHVIGPV-----L-A-E------AATG-IET-AGL--RLLVNPTGRFVLGGPMGDAGLTGRKIIVDTYGGMARHGGGAFSGKDPSKVDRSAAYAMRWVAKNIVAAGLAERVEVQVAYAIGKAAPVGLFVEAFGTEK--VDIA----VLQEAVTTVFDLRPGAIIRDLDLKR-P-------IYAETAAYGHFGR-T----------------------DLD---L--P-WEKT-DR-V-----DELK----R----AVGH----------------

>Actinobacteria_Geodermatophilus_amargosae

---------M-P--------------R-R-LFTSESVTEGHPDKIADQISDAILDEMLRQDP-----RSRVAVETLITTGQVHIAGEVTTAG-YV--------DIAEIVRRTVLRIGYDS--SR-KGFDGASCGVSVSIGAQSPDIAQGV-DT-AYEAR-T--T-GAADD--DEIE-RQ-GAGDQGLMFGYATDETPELMPLPIALAHRLSRRLSAVRKDG--S-VPYLRPDGKTQVTVVY----E--D-DR-PVGVDTV-VVSSQH-AEDIS---------IE---QLLTP-DVQELVVEPE-----L-Q-A------LG---LPT-DGY--RLLVNPTGKFVIGGPMGDAGLTGRKIIVDTYGGMARHGGGAFSGKDPSKVDRSGAYAMRWVAKNVVAAGLARRCEVQVAYAIGAAHPVGLFVDTHGTGT--VADD----VLEKAVTTVFDLRPGAIVRDLDLLR-P-------IYGPTAAYGHFGR-T----------------------DVE---L--P-WERT-DR-V-----EALR----S----AVV-----------------

>Actinobacteria_Sphaerisporangium_cinnabarinum

---------M-AE-------------L-R-LFTSESVTEGHPDKVCDQISDAILDALLTQDP-----TSRVAVETMVTTGLVHVAGEVTTDA-YV--------EIPQIVRDVVRRIGYTS--SV-IGFDADSCGISVSIGQQSPDIAQGV-DK-SLEER-D----DQGDH--DPLD-AQ-GAGDQGLMFGYASDDTPSLMPLPVWLAHRLAERLAAVRRDG--T-VPGLRPDGKTQVTVGY----D--G-DR-AVTLDTV-VLSTQH-DPDVR---------QD---E-LHR-QVAAQVVAPV-----L-D-AA----GLD---LDV-RAT--RLFVNPTGTFVVGGPQGDAGLTGRKIIVDTYGGMARHGGGAFSGKDPSKVDRSAAYATRWVAKNVVAAGLARRCEVQVAYAIGKAHPVGLYVETFGTEK--VPVE----RITAAIREVFDLRPAAIIRDLDLLR-P-------IYAPTAAYGHFGR-E----------------------LEQ---F--T-WERT-DR-V-----ADLQ----S----QV------------------

>Actinobacteria_Isoptericola_variabilis

---------M-TDA------------L-R-LFTSESVTEGHPDKVCDQISDAVLDALLEQDP-----TSRVAVETMVTTGLVHVAGEVTTNA-YV--------EIPQIVRDVVRRIGYTS--SA-MGFDGDSCGVSVSIGQQSPDIAQGV-DK-SQEQR-D----DAADS--DPLD-AQ-GAGDQGLMFGYASDETPQLMPLPIWLAHRLAERLAQVRRSG--E-VPGLRPDGKTQVTIGY----D--G-DR-AVRLDAV-VVSTQH-DPDVT---------QD---K-LAR-AVAESVVAPV-----L-E-T------VE---LDT-GGH--SLYVNPTGKFVVGGPQGDAGLTGRKIIVDTYGGMARHGGGAFSGKDPSKVDRSAAYAMRWVAKNVVAAGLARRCEVQVAYAIGRAHPVGLYVETFGTET--VPVE----RITAAIREVFDLRPAAIIRDLDLLR-P-------IYSQTAAYGHFGR-E----------------------LPD---L--T-WERT-DR-V-----DALR----A----AV------------------

>Actinobacteria_Sanguibacter_suarezii

---------M-TEA------------L-R-LFTSESVTEGHPDKVCDQISDAILDALLEQDP-----ASRVAVETLVTTGLVHVAGEVTTSA-YV--------EIPQIVRQVVKEIGYTS--SS-IGFDGDSCGVSVSIGQQSPDIAQGV-DK-ALEMR-D----DAGDL--DPLD-AQ-GAGDQGLMFGYASDETPTLMPLPIYTAHRLAERLAQVRKEN--S-LPGLRPDGKTQVTIAY----D--G-DR-AVSVDTI-VLSTQH-DPDIR---------QD---E-LAV-AVRELVVAPV-----L-G-E------ID---LPS-DGA--RLIVNPTGTFVIGGPKGDAGLTGRKIIVDTYGGMARHGGGAFSGKDPSKVDRSAAYATRWVAKNVVAAGLARRCEVQVAYAIGKAHPVGLYVETFGTET--VPLT----AITRAIRTVFDLRPGAIVRDLDLLR-P-------IYAQTAAYGHFGR-E----------------------LDD---F--T-WERT-DR-V-----AELQ----A----AV------------------

>Actinobacteria_Cellulomonas_flavigena

---------M-TAA-----------PL-R-LFTSESVTEGHPDKICDQISDAILDAILEQDT-----TARVAVETMVTTGLVHVAGEVTTSA-YV--------EIPQIVREVVRGIGYTS--SH-IGFDGDSCGVSVSIGQQSPDIAAGV-DK-AIEVR-Q----DDRDL--DPLD-LQ-GAGDQGLMFGYASDETPSLLPLPIWLAHRLSERLAQVRKDG--T-LEGLRPDGKTQVTVGY----D--G-DV-PVSLDTV-VLSTQH-EPNVS---------EA---A-LSA-QVAELVVAPV-----L-A-D------VG---IDT-SGH--RLLVNPTGQFVIGGPQGDAGLTGRKIIVDTYGGMARHGGGAFSGKDPSKVDRSAAYAMRWVAKNVVAAGLARRCEVQVAYAIGKAHPVGLYVETFGTGT--VPDV----VLTDAIRQVFDLRPAAIIRDLDLLR-P-------VYRRTAAYGHFGR-E----------------------LPE---F--T-WERT-DR-T-----ADLL----S----AVG-----------------

>Actinobacteria_Bogoriella_caseilytica

---------M-T--------------T-N-QFTSESVTEGHPDKICDRISDTILDAMLEQDP-----AARVAVETMVTTGLVHVAGEVTTEA-YV--------EIPELVRDVVRSIGYTS--SE-IGFDGSSCGVSVSIGQQSAEIATGV-NS-SEEKR-T----GTDYG--DPLD-DQ-GAGDQGLMFGYAAAETPTLMPVPIYLAHRLAERLAHVRRAG--I-VGGLRPDGKTQVTIGY----E--G-DR-AVSLDTV-VLSTQH-VADKD---------QG---D-LAE-QVRAEILTPV-----L-E-SA----ELD---LDV-SDV--RLLVNPTGTFVIGGPMGDAGLTGRKIIVDTYGGMARHGGGAFSGKDPSKVDRSAAYAMRWVAKNVVAAGLAKRCEVQVAYAIGRAHPVGLHVDTFGTHT--VPVD----RISHAIREVFDLRPAALVRDLDLLR-P-------IYAQTSAYGHFGR-E----------------------LPD---F--T-WERT-DR-A-----EALR----A----AAGMA---------------

>Actinobacteria_Leucobacter_chironomi

---------M-S--------------L-R-QFTSESVTEGHPDKICDRISDSILDAMLEQDP-----GARVAVETLVTTGLVHVAGEVSTSG-YV--------EIPQIVRDTVREIGYTS--SE-MGFDAASCGVSVSIGQQSPDIAGGV-DA-SLEVR-S--GEIAGEG--DELS-RQ-GAGDQGIMFGFATDETPELHPLPSWIAHRMAERLTEVRKNG--E-LPELRPDGKTQVTIGY----D--G-DR-AASVEAV-VVSTQH-HAEIS---------QE---A-LRD-AVERLVIRPV-----L-E-R------VD---LAS-GDA--QLFINPAGPFVIGGPMGDAGLTGRKIIIDTYGGASRHGGGAFSGKDPSKVDRSAAYAMRWVAKHVVRAGLAKRAELQVAYAIGRAHPVGLYVETFGTET--VPRE----RIEAAVREVFDLRPLAIIRDLALLR-P-------IYARTSAYGHFGR-E----------------------LPE---F--T-WEAT-PR-V-----AELR----A----AAGL----------------

>Actinobacteria_Gryllotalpicola_ginsengisoli

---------M-TE-------------L-R-LFTSESVTEGHPDKICDQISDGILDALLTADP-----ASRVAVETMVTTGLVHVAGEVTTSG-YV--------EIPQIVRDTITGIGYTS--SD-VWFDGRSCGVSISIGSQSPDIAQGV-DD-AYETR-E----EASV---DALD-RQ-GAGDQGIMFGFATTETPQLMPLPIWLSHRLSERLAEVRKTG--V-LDFLRPDGKTQVTVGY----D--G-FT-PKTIDTV-VLSTQH-SPEIS---------MP---A-LRE-AVAETVIRPV-----L-E-T------VD---LDT-SNV--RMLINPTGKFEIGGPHGDAGLTGRKIIVDTYGGASRHGGGAFSGKDPSKVDRSAAYAMRWVAKNAVAAGFADRLEVQVAYAIGSAHPVGLYVETFGTAK--LPEE----QIIRAIREVFDLRPAAIIRDLDLLR-P-------IYAQTASYGHFGR-E----------------------LPD---F--G-WEKL-DR-V-----DALR----S----AVGF----------------

>Actinobacteria_Agromyces_aureus

---------M-PQ-------------L-R-LFTSESVTEGHPDKICDQVSDSILDALLTVDP-----HSRVAVETLVTTGLVHVAGEVTTSG-YV--------EIPAIVRERVTSIGYNS--SD-VWFDGRSCGVSVSIGGQSPDIAQGV-DD-AFESR-E----RSSE---DLLD-KQ-GAGDQGIMFGYATRETPQLMPVPIWIAHRLAERLTEVRKRG--E-LDYLRPDGKTQVTVGY----D--G-QV-PRTIETV-VLSTQH-SPKVS---------TE---Q-LRA-EVEELVITPV-----L-D-T------VE---LTR-PEL--NVLINPTGRFEIGGPQGDAGLTGRKIIIDTYGGASRHGGGAFSGKDPSKVDRSAAYAMRWVAKNAVAAGLADRLEVQIAYAIGKAAPVGLYVETFGTAH--VPEE----RIIGAIREVFDLRPAAIIRDLDLLR-P-------IYAQTAAYGHFGR-E----------------------LPD---F--T-WERL-DR-V-----DDLR----A----IAGL----------------

>Actinobacteria_Agromyces_italicus

---------M-SA-------------L-R-LFTSESVTEGHPDKICDQISDSILDALLAVDP-----NSRVAVETLVTTGLVHVAGEVTTSG-YV--------EIPAIVRERVTSIGYDS--SD-VWFDGRSCGVSVSIGGQSPDIAQGV-DH-AFEAR-E----RASD---DALD-RQ-GAGDQGIMFGYATRETPELMPVPIWLAHRLAERLAHVRRAG--E-LDYLRPDGKTQVTVGY----D--G-PT-PKTIETV-VLSTQH-SPAVS---------TD---Q-LRA-EVEELVIRPV-----L-D-Q------VE---LAR-PEL--AVLINPTGRFEIGGPQGDAGLTGRKIIIDTYGGASRHGGGAFSGKDPSKVDRSAAYAMRWVAKNAVAAGLADRLELQVAYAIGKAAPVGLYVETFGTGV--LPDD----EIIGAIREVFDLRPAAIIRDLDLLR-P-------IYAQTASYGHFGR-E----------------------LPD---F--T-WERL-DR-V-----ADLR----S----AAGL----------------

>Actinobacteria_Lumbricidophila_eiseniae

---------M-SD-------------L-R-LFTSESVTEGHPDKICDQISDAILDAMLAEDP-----YSRVAVETLVTTGLVHVAGEVTTSG-YV--------EIPSIVRETVTAIGYNS--SD-VWFDGRSCGVSVSIGGQSPDIAQGV-DD-AFETR-E----GASR---DVLD-RQ-GAGDQGIMFGFATRETPELMPLPVWVAHRLAERLAEVRKQG--E-LDYLRPDGKTQVTVGY----Q--G-HT-PRTIEAV-VLSTQH-SPEVS---------TE---R-LRA-EITRLVIAPV-----L-D-R------VE---LTR-SNM--QLFINPTGRFEIGGPQGDAGLTGRKIIIDTYGGASRHGGGAFSGKDPSKVDRSAAYALRWVAKNVVAAGLADRLEIQVAYAIGKAAPVGLYVESFGTGT--VSDE----TIIRAIREVFDLRPAAIIRDLDLLR-P-------IYRQTAAYGHFGR-E----------------------LPD---L--T-WERL-DR-L-----DDLR----A----AARV----------------

>Actinobacteria_Mycetocola_miduiensis

---------M-TH-------------L-R-LFTSESVTEGHPDKVCDQISDSILDAMLDQDP-----DSRVAVETLVTTGLVHVAGEVTTEA-YV--------EIPALIRELVTNIGYNH--SD-VGFDGASCGVSVSIGQQSPDIAQGV-DD-AFESR-E----QASS---DALD-HQ-GAGDQGIMFGFATTETKQLMPVPGWIAHRLAERLTAVRKSG--E-LDYLRPDGKTQVTIGY----D--G-IT-PKSVETV-VLSTQH-EDGIA---------LD---R-LRA-DVEEHVIRPV-----L-D-G------VE---LDT-SGV--NFLINPTGRFVIGGPAGDAGLTGRKIIIDTYGGAARHGGGAFSGKDPSKVDRSAAYAMRWVAKNAVAAGFADKLEVQVAYAIGKAAPVGLYVETFGTAK--ISED----TIVDAIREVFDLRPAAIVRDLDLKR-P-------IYAATAAYGHFGR-E----------------------LPD---F--T-WERL-DK-V-----ADLR----S----AARL----------------

>Actinobacteria_Nocardioides_luteus

---------M-T--------------G-R-LFTSESVTEGHPDKIADQISDTVLDYLLEHDP-----KSRVAVETLLTTGLVVVAGEVTTDA-YA--------PVAQLVRQKILDIGYDS--SL-KGFDGHSCGVQVAIGAQSQDIAQGV-DT-AEEHR-L----ASSV---DELD-LQ-GAGDQGLMFGYACDDTPELFPLPIKMAQTLAEKLTAVRKEG--T-LDYLRPDGKTQVTIEY----D-DE-NK-AVRVDTV-VLSTQH-SEDTT---------QE---Q-LHA-DIKKYVIEPV-----L-E-Q------FKSS-VPF-DGY--KLHINPTGRFVVGGPMGDAGLTGRKIIVDTYGGMARHGGGAFSGKDPSKVDRSAAYAMRWVAKNIVAAGLARRAEVQVAYAIGKAQPVGVFVETFGTHT--VPEA----NIQKAVLEVFDLRPAAIVRDLDLLR-P-------IYAKTAAYGHFGR-E----------------------LPE---F--T-WERT-DR-A-----DALK----A----AAGA----------------

>Actinobacteria_Pimelobacter_simplex

---------M-T--------------G-R-LFTSESVTEGHPDKIADQISDTVLDYLLEHDP-----KSRVAVETLLTTGLVVVAGEVTTEA-YA--------PVAQLVRDKILEIGYDS--SA-KGFDGNSCGVQVAIGAQSPDIAQGV-DT-AEDVR-L----GGSS---DELD-KQ-GAGDQGLMFGYACDDTPELFPLPIKIAQTLAERLTEVRKNG--T-LAYLRPDGKTQVTIEY----D-ED-DR-AVRVDTV-VLSTQH-AEDVS---------QE---Q-IAA-DITTHVIEPV-----L-E-Q------FKTS-VPF-DGY--KLHINPTGKFVVGGPMGDAGLTGRKIIVDTYGGMARHGGGAFSGKDPSKVDRSAAYAMRWVAKNIVAAGLARRAEVQVAYAIGVAKPVGVFVETFGTGV--VPDE----KIQAAVLEVFDLRPAAILRDLDLRR-P-------IYAKTAAYGHFGR-E----------------------LPE---F--T-WERT-DR-A-----DALK----A----AVGA----------------

>Actinobacteria_Rhodoluna_planktonica

---------M-SN-------------L-R-LFTSESVTEGHPDKICDQISDSILDAMLEQDS-----KSRVAVETMVTTGLVHVAGEVTTEG-YV--------EIPQLVRNVVRNIGYTS--SE-IGFDGGSCGVSISIGQQSPDIAQGV-DA-GFELR-A----NSST---ERFD-SQ-GAGDQGIMFGYATNETPNYMPVAIDTAHKLSRRLADARKNG--E-ISFLRPDGKTQVTVGF----E--G-ST-PKTIDTV-VLSTQH-NPGVD---------QA---E-IER-EVLERVIKPI-----L-S-Q------TK---LDL-GAT--KFLINPTGKFEIGGPMGDAGLTGRKIIVDTYGGAARHGGGAFSGKDPSKVDRSAAYAMRWVAKNVVAAGLADRAEVQVAYAIGVARPVGLYVETFGTEK--VSVE----KITAAIQKTFDLRPRAIIEELDLLR-P-------IYAQTATYGHFGR-E----------------------EKD---F--T-WEST-EK-A-----SALS----A----AI------------------

>Actinobacteria_Arcanobacterium_urinimassiliense

---------M----------------I-Q-PFTSESVTEGHPDKVCDRISDAILDAMLEVDK-----NARVALETIVTTGTVHVIGEVTTSA-YV--------EIPQIVRNTLRSIGYTS--SQ-IGFDADSCGVAVSIGAQSPDIAEGV-DN-SLEER-S----ADAA---DKYD-QQ-GAGDQGLIFGYACNETKELMPLPIHLSHRLAEKLAFVRKSG--I-VGGLRPDGKTQVTVNY----D-EN-EK-PVGIASI-VISTQH-DPEVE---------LS---Y-LQK-VIKEQVIVPV-----F-R-A-----EVPQ--FSL-ADT--QILINPSGRFVVGGPLGDSGLTGRKLIVDTYGGMARHGGGAFSGKDPSKVDRSAAYAARWIAKNIVAAELAERCEIQISYAIGKAHPVSLRIETFGTEK--VPVE----NIKSAVEKVFDLRPAAIIEDLQLLR-P-------IYAKTSAYGHFGR-E----------------------LPE---F--T-WEQT-DR-A-----AALK----E----AVTQL---------------

>Actinobacteria_Brevibacterium_mcbrellneri

---------M-KS-------------L-R-YFTSESVTEGHPDKICDQISDAVLDNLLAQDQ-----RSRVAVETLVTTGLVHVAGEVTTDG-YA--------DVATIARDVITGIGYTN--SD-MGFDGHSCGVTVSIGSQSTDISDGV-TT-SIEAR-A----GGN----DPLE-RL-GAGDQGLMFGYADNATPELMPMPIHLAHRMSQRLSEVRKNG--T-VDYLRPDGKTQVTIGY----D--G-DK-AVSVDCV-VLSSQH-SEGTD---------HR---Q-LEG-DIREHVINPI-----L-N-DSG---------LDT-SGT--EFWINPAGPFIVGGPMGDAGLTGRKIIVDTYGGFARHGGGAFSGKDPSKVDRSGAYAMRWVAKNVVAAGLADRAEVQVAYAIGKSHPVGLYVETFGTEK--EDPA----RIEKAIEEVFDLRPAAIIRELALNR-P-------IYNLTSTYGHFGR-E----------------------LPE---F--T-WENT-DR-A-----EALT----R----AI------------------

>Actinobacteria_Actinomyces_odontolyticus

---------M-S--------------Q-Q-LFSSESVTEGHPDKVCDRVSDAILDALLAADP-----RSRVAVETMAATGLIHIAGEVTTEA-YV--------EIPEIVRREILSIGYDS--SR-VGFDGASCGVSVSLDGQSPDIAGGV-DE-ALEVR-G----TQGG---DPRD-RL-GAGDQGIMFGYASSDTPDLMPAPIWLAHRLAKRLTDVRKQG--I-VEGLRPDGKTQVTLAY----A--D-GR-PVGIDTV-VVSTQH-DEALS---------QS---A-ITD-AVRDYVINPV-----I-E-ESG----LA---LAT-GSM--TTLINPSGRFVLGGPAADAGLTGRKIIVDTYGGMARHGGGAFSGKDPSKVDRSATYAARWVAKNVVAAGIAQRCEIQLAYAIGRARPIGLYVDTFGTTS--VPED----RIADAIREVFDLRPLGMIEDLDLLR-P-------IYRETAAYGHFGR-D------------------------Q---F--P-WEAT-NR-V-----EQLK----A----ALA-----------------

>Actinobacteria_Brachybacterium_ginsengisoli

---------M-TSS-----------EL-R-TFTSESVTEGHPDKICDQISDAILDDLLAQDR-----DARVAVETMVTTGLVHVAGEVRTTG-YT--------DVATIVRDVIREIGYDS--SD-KGFDADSCGIEVSIGAQSPDIAQGV-DT-SFEAR-G----DLAA---GAFS-KL-GAGDQGLMFGYACRDTPELMPLPIHAAHRLTANLSAARRDG--V-LPYLRPDGKTQVSIGY----D-ED-GV-ARSVDAV-VVSTQH-SDEVD---------LL---SQLAP-AISKVVIAPV-----L-E-G------LENLGLDT-SSV--TTHINPSGRFVLGGPKGDAGLTGRKIIVDTYGGMARHGGGAFSGKDPSKVDRSGAYAMRWVAKNIVAAGLADRCEVQVAYAIGVAEPVGLYVETFGTGR--VPSH----QIAAAVRKVFDLRPAALIDALDLLR-P-------IYALTSVHGHFGR-E----------------------LPE---F--T-WERT-DR-A-----GELE----R----AL------------------

>Actinobacteria_Egicoccus_halophilus

---------M-SR-------------R-S-LFTSESVTEGHPDKVADQISDAILDAILTEDP-----AGRVACETLVTTGQVMIAGEISTRT-YV--------DIPKVVRDTILRIGYDS--AD-VGFDGNTCGVSVAIDEQSPDIAMGV-DT-AYESR-T----GSDT---DPYA-IQ-GAGDQGMMFGYATDETPSYMPMPIHLAHRMAQRLAAVRKSG--D-VSYLRPDGKTQVTVDY----E--D-GV-PKAVTAV-VVSTQH-RAEID---------LE---TLLRP-DIEEQVIQPL-----L-P-D---D-------IDT-SNL--QVYVNPTGRFELGGPVADAGLTGRKIIVDTYGGMARHGGGAFSGKDPSKVDRSAAYAVRWVAKNIVAAGLARRAELQVAYAIGVASPVSLNLETFGTEQ--ADPD----AILRAVREVFDLRPAAIADALELRR-P-------GYLDTAAYGHFGR-E----------------------GER---F--T-WERT-DR-A-----DALR----D----AVS-----------------

>Actinobacteria_Microthrix_parvicella

---------M-A--------------K-W-TFTSESVTEGHPDKMADQISDSVLDALLTQDP-----DSRVACETMVTTGLAIVAGEISTKA-YV--------DIQSIVRQTIRDIGYDR--ED-VGYDGSTCGVLVAIDEQSSDIAQGV-DD-SEEVR-S--G-TAGEE--DQLD-QQ-GAGDQGMMFGYACDETPELMPLPINTAHRLAERMAEVRKAG--T-IPYLRPDAKTQVTFEY----E--D-GQ-AVALKTV-LISTQH-NEGVD---------RD---ADIRP-DLIESVINPT-----I-P-E---Q-------FRG-DDF--EVFVNPTGRFVIGGPVGDAGLTGRKIIVDTYGGSARHGGGAFSGKDPSKVDRSAAYATRWVAKNVVASGAATRCEVQVAYAIGMAHPISILVETFGTET--VENA----KIEAAVRDIFDLRPGAIVRDLDLRR-P-------IYRETAAYGHFGR-T----------------------GDG---F--T-WELT-NR-A-----DDLR----S----ALGL----------------

>Actinobacteria_Solirubrobacter_soli

---------M-TQ---------LSDNE-Y-LFTSESVTEGHPDKVADQISDGVLDAVLREDP-----TGRVACETLVNTGLVVVSGEISTST-YV--------DIQEIARETIRKIGYVD--AD-LGFSADSCAVINAIDKQSPDIAQGV-DQ-ALEKR-T----DASDD--DEL--DIAGAGDQGMMFGYASNETEELMPLPISLAHKLAERLAAVRKDG--T-LDYLRPDGKTQVSVRY--R-D--G--K-PVEIEKL-LISTQH-REGAE--------------ELIPN-DLWEHVVLPI-----L-P-T-------E-L-YDA-EKLRKNFLVNPTGRFVIGGPVGDAGLTGRKIIVDTYGGMARHGGGAFSGKDPSKVDRSAAYAARYVAKNLVAAGLADRVEVQVAYAIGVAHPVSVMVETFGTEK--IGRG----RIAALVDEFFDLRPGAFREYLKLHR-P-------IYQKTAAYGHFGR-D----------------------DED---F--T-WEKT-DK-A-----EALR----E----AAGLGAADPVA---------

>Actinobacteria_Fortiea_contorta

-----------MS------------RR-Y-LFTSESVTEGHPDKICDQISDTIIDALLTQDP-----TSRVAAEVVVNTGLVLITGEITTKA-NV--------NYVHLARKKIAEIGYTD--AD-NGFSANSTSVLIALDEQSPDIAQGV-NT-AQETR-E----QDS-D--ELFD-KI-GAGDQGIMFGFASNETPELMPLPISLAHRIARRLAAVRKTG--D-LSYLRPDGKTQVTVVY--E-D--G--R-PVGIDTI-LISTQH-TASIGEITDEAAV-QA----KIKA-DLWSAVVEPVFGDIDVKP-D---------------AET--RFLVNPTGKFVVGGPQGDSGLTGRKIIVDTYGGYSRHGGGAFSGKDPTKVDRSAAYAARYVAKNVVAAGLAEKCEVQLSYAIGVARPTSILVETFGTGK--VDEE----TLLELVKKHFELRPAGIIHSFNLRNLPNERGGR-FYQDVAAYGHFGR-S----------------------DLD---L--P-WERT-DK-A-----ELLK----Q----ALNESLSAAIAQTLQ-----

>Actinobacteria_Cyanosarcina_burmensis

---MEA---C-NL------------SR-Y-LFTSESVTEGHPDKICDQISDAILDAHLSQDP-----TSRVAAEVVVNTGLVLVTGEISSKA-KV--------DYVKLVRQKIAEIGYTD--AN-NGFSANSCSILVALDEQSPDIAQGV-NK-AQEYE-Q----GDS-E--DDWS-AI-GAGDQGIMFGYACKETPELMPLPISLAHRLARQLAKVRKIG--K-LSYLRPDGKTQVTVVY--E-N--G--R-PVGIDTI-LISTQH-SEATT---------LG----EIYQ-GLLLEVVEPVFADLDIQI-D---------------NST--RFLVNPTGKFVIGGPQGDSGLTGRKIIVDTYGGYSRHGGGAFSGKDPTKVDRSASYACRYVAKNIVAAGLADKCEVQLSYAIGMSKPVSILIDTFGTGK--LPEE----DLLNLVNQHFDLRPAGLIHTFNMQGLPQERNGR-FYQDVAAYGHFGR-M----------------------DLD---L--P-WERL-DK-V-----PQLQ----E----AITVQMSA------------

>Actinobacteria_Trichormus_variabilis

-----------MS------------RR-Y-LFTSESVTEGHPDKICDQISDTILDALLTQDP-----SSRVAAEVVVNTGLVLITGEITTKA-NV--------NFVNLARKKIAEIGYTD--AV-NGFSANSASVLVALDEQSPDIAQGV-NT-AQETR-S----EDS-E--ELFD-KI-GAGDQGIMFGFACNETPEFMPLPISLAHRIARRLAAVRKTG--D-LPYLRPDGKTQVTVVY--E-D--G--K-PVGIDTI-LISTQH-TPNIGDITDEAGV-QA----KIKE-DLWSAVVEPVFGDIDVKP-D---------------QGT--RFLVNPTGKFVIGGPQGDSGLTGRKIIVDTYGGYSRHGGGAFSGKDPTKVDRSAAYAARYAAKNIVAAGLAEKCEVQLSYAIGVARPVSIFVDTFGTGK--VDDE----ILLELVKEYFELRPAGIIHAFNLRNLPSERGGR-FYQDIAAYGHLGR-S----------------------DLD---L--P-WERT-DK-V-----DLLK----Q----AANKLLSEVVAQALT-----

>Actinobacteria_Tychonema_bourrellyi

-----------MS------------RH-Y-LFTSESVTEGHPDKICDQISDTILDALLTEDP-----KSRVAAEVVVNTGLMLLTGEITTKA-QV--------NYIELARKKIADIGYIH--AD-NGFSANSCSVLVALDTQSPDIAQGV-NT-AHESR-E----QSS-E--EELD-AI-GAGDQGLMFGFACNETPELMPLPISLAHRICRQLAAVRKTG--Q-LPYLRPDGKSQVTVVY--E-D--G--K-PVGIDTI-LISTQH-TEAIGDIVDTAAV-QA----KIKE-DLWTAVVEPVFADIAIKP-D---------------AET--RFLVNPTGKFVIGGPQGDSGLTGRKIIVDTYGGYSRHGGGAFSGKDPTKVDRSAAYACRYVAKNIVAAGLAEKCEVQLSYAIGVARPVSMMIETFGTGK--VDDD----RLLEVVKQLFELRPAGIIQAFNLQNLPAERGGR-FYQDVAAYGHFGR-T----------------------DLE---L--P-WEQT-DK-V-----ELLK----S----ALSQQLSAVAG---------

>Actinobacteria_Microcoleus_vaginatus

-----------MS------------RR-Y-LFTSESVTEGHPDKICDQISDTILDALLTEDP-----KSRVAAEVVVNTGLMLLTGEITTKA-QV--------NYIELARKKIADIGYIH--AD-NGFSANSCSVLVALDSQSPDIAQGV-NT-AQESR-E----QSS-E--EELD-AI-GAGDQGLMFGFACNETPEMMPLPISLAHRICRQLAAVRKTG--Q-LSYLRPDGKSQVTVIY--E-D--G--K-PVGIDTI-LISTQH-TETIGDITDTAAV-QA----KIKE-DLWTAIVQPVFADISVKP-D---------------AET--RFLVNPTGKFVVGGPQGDSGLTGRKIIVDTYGGYSRHGGGAFSGKDPTKVDRSAAYACRYVAKNIVAAGLAQKCEVQLSYAIGVARPVSMMIETFGTGK--VDDD----RLLEVVKQHFELRPAGIIQAFNLQNLPAERGGR-FYQDVAAYGHFGR-T----------------------DLD---L--P-WEET-DK-A-----ELLK----E----ALSQKLSGVAG---------

>Actinobacteria_Aliterella_atlantica

-----------MS------------RR-Y-LFTSESVTEGHPDKICDQISDTILDALLTQDP-----TSRVAAEVVVNTGLVLITGEITTKA-QV--------NYVDLARKKIADIGYTE--AE-NGFCANSCSVLVALDEQSPDIAQGV-NT-AQESR-E----QDS-D--EQFD-AI-GAGDQGLMFGFACNETPELMPLPISLAQRIARRLAAVRKTG--Q-LPYLRPDGKTQVTVTY--E-D--G--K-PVGIDTI-LVSTQH-TANIGDISEQEAV-QA----KIKE-DLWAAVVEPVFADCDIKP-D---------------EQT--RFLVNPTGKFVVGGPQGDSGLTGRKIIVDTYGGYSRHGGGAFSGKDPTKVDRSAAYACRYVAKNIVAAGLAEKCEVQLSYAIGVARPVSILVETFGTGK--IDDD----RLLELVKQHFELRPAGIIHAFNLRQIPGERGGR-FYQDVAAYGHFGR-T----------------------DLE---L--P-WEQT-DK-A-----AILS----E----ALTQSVTV------------

>Actinobacteria_Neosynechococcus_sphagnicola

-----------MS------------RR-Y-LFTSESVTEGHPDKICDQISDTILDALLAQDP-----SSRVAAEVVVNTGLVLITGEITTQA-TV--------NYIDLARKKITEIGYMG--VD-SGFSANSCAVLIALDSQSPDIAQGV-NT-AHEHR-E----QAS-D--EELD-AI-GAGDQGLMFGFACNETPELMPLPISLSHRIARKLAAVRKTG--Q-LPYLRPDGKTQVTVAY--E-D--G--C-PVGIDTI-LISTQH-SPTIGEISDDAAV-QA----KIRE-DLWTLVVQPVFGDIEIKP-D---------------ATT--RFLMNPTGKFVIGGPQGDSGLTGRKIIVDTYGGYSRHGGGAFSGKDPTKVDRSAAYACRYVAKNIVAAGLADKCEVQLSYAIGVARPVSIMIDTFGTGK--VEDD----LLLELVKQNFELRPAGIIQAFNLRYLPSERGGR-FYQDVAAYGHFGR-T----------------------DLE---L--P-WEQT-DK-A-----ALLQ----E----AAKQPLSITGSRT-------

>Actinobacteria_Acaryochloris_marina

-----------MP------------KR-Y-LFTSESVTEGHPDKVCDQISDTILDALLSQDP-----ASRVAAEVVVNTGLVLLTGEITTKA-QV--------NFVDLVRQKITEIGYTD--SD-NGFAANSCSVLVALDEQSPDIAQGV-DS-AQETR-E----EKS-D--QELD-AI-GAGDQGLMFGFACNETPELMPLPISLAHRVTRRLAAVRKT---Q-LGYLRPDGKSQVTVTY--E-D--G--R-PVGIDTI-LVSTQH-DATIGDITDPAAV-QA----KIKE-DLWNAVVLPVFADIDIKP-D---------------DNT--RFLVNPTGQFVVGGPQGDAGLTGRKIIVDTYGGYSRHGGGAFSGKDPTKVDRSAAYACRYVAKNIVAAGLAEKCEVQLSYAIGVARPVSILIETFGTGK--VDED----RLLQVVQENFELRPAGLIQTFGLTKLPGERGGR-FYQDVAAYGHFGR-T----------------------DLD---L--P-WEAT-DK-A-----DLLK----Q----ALSPALSGNA----------

>Actinobacteria_Nostoc_piscinale

---------M---------------KKDF-MFTSESVTEGHPDKLCDQISDAIVDRFLQQDP-----YARVITECAASTGILFIAARFEPNA-NV--------DFTNIARQIIEQIGYEQ-----KQFNSKTCSILTSLRELPASQTHLF-DE-------K----NLS-D--EEIE-KI-TVTNQVTVFGFACNQTYTLMPLPIWLAHKLARQLSEVRHKN--I-LPYLTPDGKTQVGVEY--R-D----RR-PYRIHSITVIASQNKAGKPD---------YQ----QLQN-DIQETVINPVFENEEIRP---------D-------AKT--RIFINPDGPFIKGGPAVHSGLTGRKNAIDTYGEYSKHSGSALSGKDPIRIDRIGAYIARYAAKNIVAAKLADECEVQLSYSIGLSRPVSIQVETFGTGK--ISDE----EITNLLEKHFDFRLAGIIKQFNLRHLPTINPGG-FYRQLAVYGHVGR------------------M-----DID---L--P-WEKT-DK-V-----GVF------------------------------

>Aquificae_Thermocrinis_ruber

-----------MH-----------------IRMAESPTEGHPDKVADLIADALLDEFLRKDP-----YSRVSLEVMVISGMTFVAGHVSTEG-YV--------DIPGIVRSTIKEVGYNR--PE-LGFDADASAVIISIEEQSPEIVLGL----------S--Q-----E----------GAGDTAVVIGYACNETESYMPLPISLAHFLSKKLADLRKTG--R-APFLRPDGKALVTVAY--E-G--T--K-PLFVKDI-ILFAQH-DPDVS---------LE----KLKE-YLVEEVVKKV-----V-P-S-------R-Y-IS--KET--RILVNPSGRFVLGGPVADVGQTGRKIVSDAYGDTAYSGGSAFSGKDPTKTDRSASYLARMMAKHVVAGGFAERCLVQLAYAFGVSEPIAFDIETYGTEK--VEKE----RIKEALREIFPLNPRRMIEFLDLRK-P-------IYKKTACYGHFGK-E----------------------------L--P-WEKL-TK-L-----EELK----ERL--GGGIQ---------------

>Aquificae_Thermosulfidibacter_takaii

----MP---Y-RG------------GK-R-LFTSESVTEGHPDKVADQVSDAILDAILEKDP-----YGRVACETLVTTGLVMVAGEISTTC-YV--------DIPTIARETIRDIGYTR--AK-FGFDYETCAVVTSIHEQSPDIAMGV-DP-G-------------------------GAGDQGLMFGFAVNETPELMPLPIMLAHKLCMRLAEVRKKD--I-LDYLRPDGKSQVTVEY--E-N--G--K-PVRIHSI-VLSAQH-APDIT---------LR----KLRE-DLIEYVIKPV-----I-P-S-------E-Y-LDE-EYV--KYYINPTGRFVLGGPMADTGLTGRKIIVDTYGGAARHGGGCFSGKDPTKVDRSASYMARYVAKNIVASGIADICELQLAYAIGVPEPIAIYVDTKGTGK--IPDE----KIAEIVGKVFDLTPKGMIEALDLRR-P-------IYKKTAAYGHFGR-E----------------------LPE---F--T-WERV-DK-A-----EEIR----R----EAELIGG-------------

>Aquificae_Thermocrinis_albus

---------MGIS-----------------VRMAESPTEGHPDKLADLIADALLDEFLKKDP-----YSRVSLEVVVISGMTFVAGHVSTEG-YV--------DIPTVVRNTIKEVGYTR--PE-YGFDADASAVITSIEEQSPDIALGI----------S--G-----E----------GAGDTATVVGYACNETDSYMPLPITLAHSISKRLADIRKSG--K-APFLRPDGKVLITVLY--E-N--H--K-PVSAKDI-IVYTQH-DPDVS---------PE----KLRE-FIYEEVIKKV-----L-P-E-------K-Y-MT--SHT--RVLVNPSGRFVLGGPVADVGQTGRKIVSDAYGDTAYSGGSAFSGKDPTKTDRSASYLARMMAKHVVASGLADRCLVQMAYAFGVSEMIAFDVETYGTEK--LEKE----KIVKALLEIFPTSPRQIIEFLDLRK-P-------IYKKTACYGHFGK-E----------------G-----------L--P-WEEL-RY-L-----EKLR----D-I--ATAL----------------

>Aquificae_Hydrogenivirg_sp

---------M-YS-----------------LRMAESVTEGHPDKIADQIADALLDEFLRKDP-----YSKVSMEIFVTTGLVMVGGELSTES-FV--------DIPKVVRGVVKEIGYTK--PE-LGFDADTCAVVTSIDEQSPEIALGV----------S--G-----E----------GAGDTAIVVGYAVKEAPNLMPWSITLAHRITRRVSDLRKTG--R-FPFLRPDGKVLVSMVY--E-D--G--K-PRYVKDI-VAYVHH-DPDIS---------LQ----NLRE-LVIEEVLKKE-----V-P-E-------E-F-LK--SET--NVKVNPTGRFVIGGPVADTGLTGRKIVSDAYGDIGFSGGSAFSGKDPSKTDRSGSYMARMIAKHVVAAGLADKCVVQIGYAFGLTEPIAFDVETFGTEK--LEKE----KIEEIVQKVFPLKPCDIIEFLDLRK-P-------IYRDTACYGHFGR-E----------------R-----------F--P-WERL-DY-L-----DKLK----ELL----------------------

>Bacteroidetes_Zunongwangia_profunda

-----------MA---------------Y-LFTSESVSEGHPDKIADQISDTLLDNFLAFDE-----ESKVACETLVTTGQVVLAGEVRSNT-YL--------DVQQIARDVINDIGYTK--GA-YQFSGDSCGVISLIHEQSPDINQGV-DR-ATK------------------E-EQ-GAGDQGMMFGYATTETANFMPLALDISHKMVYELAELRREG-NE-IPYLRPDAKSQVTIEYS-D-D--N--I-PQRIVAI-VLSTQH-DQF---MENDEEM-LA----KIKK-DIIEILMPRVIAQ--L-P-E-----YVQEL-FN--NDI--KYHINPTGKFVIGGPHGDAGLTGRKIIVDTYGGKGAHGGGAFSGKDPSKVDRSAAYASRHIAKNLVAAGVADEVLVQVSYAIGVVEPTSISVDTYGTSKVAMSNG----EIAKKVGEIFDMRPFAIEERLNLRN-P-------IYRETAAYGHMGK-EPKKVTKIFKSPYNGE----IKKEVE--LF--T-WEKL-DY-V-----DKVK----D----AFGL----------------

>Bacteroidetes_Muricauda_zhangzhouensis

-----------MS---------------Y-LFTSESVSEGHPDKIADQISDALLDNFLAFDP-----ESKVACETLVTTGQVVLAGEVKSHT-YV--------DLQSIAREVINKIGYTK--GE-YQFSGDSCGVISLIHEQSQDINQGV-DR-GSK------------------E-EQ-GAGDQGMMFGYATKETENYMPLALDISHKILQVLAELRREG-TR-IPYLRPDAKAQVTIEYS-D-D--N--V-PQRIDTI-VVSTQH-DEF----DADEVM-LA----KIKE-DIIQILIPEVQKL--L-P-E-----AIQKL-FT--DSI--KYHINPTGKFVIGGPHGDAGLTGRKIIVDTYGGKGAHGGGAFSGKDPSKVDRSAAYAARHVAKNLVAAGVADEILVQVSYAIGVVEPTSIFVETYGTSKLGLSDG----EIAKKASELFDMRPFAIEERLKLRN-P-------IYLETAAYGHMGK-QPQTLTKVYESPYNGR----IEKEVE--LF--T-WEKL-DM-V-----DQVK----E----AFDL----------------

>Bacteroidetes_Psychroflexus_gondwanensis

-----------MS---------------Y-LFTSESVSEGHPDKIADQISDALLDSFLAFDD-----TSKVACETLVTTGQVVLAGEVRSNT-YL--------DVQQIARDVINDIGYTK--GE-YQFSGDSCGVISLIHEQSKDIYQGI-DK-TSK------------------E-DQ-GAGDQGMMFGYATNETENYMPLALDISHKLLIELAKLRREG-KD-ITYLRPDAKAQVTIEYS-D-D--N--V-PQRIDTI-VISTQH-DNF----DEDEPM-LK----KIEK-DIHSILIPRVKAL--F-D-E-----SIQAL-FN--DDI--TYHINPTGKFVIGGPHGDAGLTGRKIIVDTYGGKGGHGGGAFSGKDPSKVDRSAAYAARHVAKNMVAAGIADEVLVQVSYAIGVSEPTSILVNT-TNKKIDMSDG----DIAKKIRNIFDMRPGMIEERLKLRN-P-------IYRETAAYGHMGK-EPRFETKIFRSDYSGE----IRREVE--LF--T-WEKL-DY-V-----DKLK----D----AFGL----------------

>Bacteroidetes_Seonamhaeicola_aphaedonensis

-----------MS---------------Y-LFTSESVSEGHPDKVADQISDALIDNFLAFDI-----DSKVACETLVTTGQVVLAGEVKSTT-YL--------DVQKIARDTINKIGYTK--GE-YMFDGNSCGVFSAIHPQSPDINQGV-DR-GTK------------------Q-EQ-GAGDQGMMFGYATNETENYMPIALDLSHRILIELAALRREN-KD-ITYLRPDSKSQVTIEYS-D-D--N--V-PQRIDAI-VISTQH-DDF----DDDETM-LA----QIRK-DIVEILIPRVVAK--L-P-E-----ATQNL-FN--DNI--KYHINPTGKFVIGGPHGDTGLTGRKIIVDTYGGKGAHGGGAFSGKDPSKVDRSAAYATRHIAKNLVAAGVSDEVLVQVSYAIGVVEPMGIFIDTYGTSKVNLSDG----DIAKKVSKIFDMRPYAIEERLKLRS-P-------MYSETAAYGHMGR-TNRTVTKTFSQPSGES----ITLDVE--LF--T-WEKL-DY-V-----DKVK----Q----AFGL----------------

>Bacteroidetes_Belliella_buryatensis

-----------MP---------------Y-LFTSESVSEGHPDKIADQISDALIDNFLAFDP-----RSKVACETLVTTGQVILAGEVKSDT-YL--------DVQKIARDVINRIGYTK--GE-YMFDGNSCGVLSAIHEQSPDINQGV-DR-NSP------------------E-EQ-GAGDQGMMFGYATKETEDYMPLALDLSHRILRELAALRREN-DQ-IKYLRPDSKSQVTIEYS-D-D--N--V-PQRIDAI-VISTQH-DDF----ADEPTM-LA----KIKE-DLIAILIPRVKAQ--L-K-P-----EIQAL-FT--EDI--TYHINPTGKFVIGGPHGDTGLTGRKIIVDTYGGKGAHGGGAFSGKDPSKVDRSAAYATRHIAKNMVAAGIADEILVQVSYAIGVAKPMGIYVNTYGTSKLGLSDG----AIAKKVEEIFDMRPYAIEQRLKLRY-P-------IYEETAAYGHMGR-KNEVVSKTFFTPDGKS----IDLDVE--LF--T-WEKL-DY-V-----DKIK----K----AFGL----------------

>Bacteroidetes_Pelobium_manganitolerans

-----------MS---------------Y-LFTSESVSEGHPDKVADQISDALIDNFLAFDK-----DSKVACETLVTTGQVVLAGEVKSQS-YL--------DVQKIARDVIRKIGYTK--SE-YMFEANSCGILSAIHEQSQDINQGV-DR-AKP------------------E-EQ-GAGDQGMMFGYASNETDNYMPLALDLAHKLLIELAAIRREN-SE-IKYLRPDAKSQVTIEYD-D-N--H--Q-PIRIDTI-VISTQH-DEF----DEEGKM-LE----KIKS-DMISILIPRVKAS--L-K-P-----ELRQL-FT--DEI--QYHINPTGKFVIGGPHGDTGLTGRKIIVDTYGGKGAHGGGAFSGKDPSKVDRSAAYATRHIAKNLVAAGVADEVLVQVSYAIGVAEPCGIFIDTNGTSKVNLTDG----EIAKKVQEIFDMRPYFIEQRLKLRN-P-------IYSETAAYGHMGR-KNEVVTKTFYGSNGEE----KKVEVE--LF--T-WEKL-DF-V-----DKVK----V----AFGL----------------

>Bacteroidetes_Chitinophaga_barathri

-----------MP---------------Y-LFTSESVSEGHPDKVADQISDALIDNFLAYDL-----TSKVACETLVTTGQVVLAGEVKSDA-YL--------DVQDIAREVIRRIGYTK--SE-YMFEANSCGIFSAIHEQSPDINQGV-ER-KSP------------------E-EQ-GAGDQGMMFGYATKETENFMPLALDLAHKLLIELAALRREN-KD-IQYLRPDAKSQVTIEYS-D-D--N--Q-PIRIDTI-VISTQH-DDF----ESEQAM-LA----KIKQ-DMIDILIPRVKKG--L-K-P-----ELQSL-FT--DKI--TYHINPTGKFVIGGPHGDTGLTGRKIIVDTYGGKGAHGGGAFSGKDPSKVDRSAAYATRHIAKNLVAAGVADEVLVQVSYAIGVAKPCGVFVDTYGTAKVGLKDG----EIARKVEEIFDLRPYAIEQRLKLRN-P-------MYGETAAYGHMGR-ESKTVTKVFNAGKKNE----KKVDVE--LF--T-WEKL-DY-V-----DKVK----A----AFGL----------------

>Bacteroidetes_Solitalea_canadensis

-----------MA---------------Y-LFTSESVSEGHPDKVADQISDALIDNFLAFDP-----NSKVACETLVTTGQVVLAGEVKSKT-YL--------DVQQIARDVIRKIGYTK--SE-YMFEANSCGVLSAIHEQSSDINQGV-ER-TKP------------------E-DQ-GAGDQGMMFGYATNETDNYMPLALDIAHQLLIELAALRREN-KD-ITYLRPDAKSQVTIEYS-D-D--H--K-PVRIDAI-VVSTQH-DEF----DTEDVM-AK----KITD-DIINILIPRVKAK--Q-K-P-----EIQKL-FT--DNI--NYHINPTGKFVIGGPHGDTGLTGRKIIVDTYGGKGAHGGGAFSGKDPSKVDRSAAYATRHIAKNLVASGVCSEALVQVSYAIGVAKPMGLYVNTYGSAKVGLTDG----EIANKLSEIFDMRPYFIEERLKLRN-P-------IYQETAAYGHMGR-KNEVVSKSFSLPDGRS----ISKEVE--LF--T-WEKL-DY-V-----DKIK----Q----AFGL----------------

>Bacteroidetes_Microscilla_marina

-----------MA---------------Y-LFTSESVSEGHPDKVSDQISDALIDYFLAYDP-----SSKVACETLVTTGQVVLAGEVKSDA-YL--------DVQEIAREVIKKIGYTK--SE-YMFEATSCGVLSAIHEQSPDINQGV-DR-QKP------------------E-EQ-GAGDQGMMFGYATNETANYMPLALDLSHKILKELADMRREA-TD-IKYLRPDAKSQVTIEYS-D-D--N--K-PVRIDTI-VVSTQH-DDF----DTEEAM-QA----KIRE-DIISFLIPRVKAQ--L-P-E-----HIQAL-FN--DDI--TYHINPTGKFVIGGPHGDTGLTGRKIIVDTYGGKGAHGGGAFSGKDPSKVDRSAAYASRHIAKNLVAAGVADEILVQVSYAIGVAQPTSIMVDTYGSAKVDMTDS----VIGQKVAEVFDMRPYYIEQRLKLRN-P-------MYQETAAYGHMGR-ESETITKTFNAGKSNE----KTVEVD--LF--T-WEKL-DY-V-----DKVK----A----AFSL----------------

>Bacteroidetes_Lutibacter_agarilyticus

-----------MS---------------Y-LFTSESVSEGHPDKVADQISDALVDNFLAFDE-----NSKVACETLVTTGQVVLAGEVKSKT-YL--------DVQKIARDVINKIGYTK--GE-YMFDGNSCGVLSAIHEQSQDINQGV-DR-ESK------------------E-QQ-GAGDQGMMFGYATNETENFMPLALDLSHLILVELAELRREN-KD-ITYLRPDSKSQVTIEYT-D-D--N--V-PFRIKDI-VVSTQH-DDF---MENDEEM-LA----KIKS-DIISILIPRVIAK--V-P-T-----EIKAL-FN--DHI--VYHVNPTGKFVIGGPHGDTGLTGRKIIVDTYGGKGAHGGGAFSGKDPSKVDRSAAYATRHIAKNLVAAGVADEILIQVSYAIGVVEPMGIYVNTNGSANVDLADG----EIAEIVSTIFDMRPASIESRLKLRQ-P-------MYAETAAYGHMGR-KNETVNKSFKNVDGTE----ISVDVE--LF--T-WEKL-DY-L-----DKVK----E----AFNL----------------

>Bacteroidetes_Lutibacter_sp

-----------MS---------------Y-LFTSESVSEGHPDKVADQISDALVDNFLAFDK-----DSKVACETLVTTGQVILAGEVKSKT-YL--------DVQKIARDVINKIGYTK--SE-YMFDGNSCGVFSSIHEQSKDINQGV-DR-DTK------------------E-DQ-GAGDQGMMFGYATNETENFMPLALDLSHEILYEFAELRREN-KE-ITYLRPDSKSQVTIEYT-D-D--N--V-PIRIKDI-VVSTQH-DDF---DEDDKAM-LA----KIKS-DVINILIPRVIKN--Q-P-T-----AIKAL-FD--DKI--VYHVNPTGKFVIGGPHGDTGLTGRKIIVDTYGGKGAHGGGAFSGKDPSKVDRSAAYATRHIAKNIVAAGVANEILIQVSYAIGEVEPMGIYVNTYGTSNVDKSDG----EIAKIINQLFDLRPAAIEKRLKLRQ-P-------MYLETAAYGHMGR-ENRTITKTFKNVDGTE----KVIDVE--LF--T-WEKL-DY-V-----TKVK----E----AFGL----------------

>Bacteroidetes_Hydrotalea_flava

-----------MS---------------Y-LFTSESVSEGHPDKVADQISDALIDNFLAFDP-----QSKVACETLVTTGQVVLAGEVRSKA-YL--------DVQEIARGVIRKIGYTK--SE-YMFEAHSCGVLSAIHEQSPDINQGV-DR-KKK------------------E-EQ-GAGDQGMMFGYATNETDDYMPLTLDLAQKILIELAALRREN-KQ-IKYLRPDAKSQVTLEYD-D-N--N--K-PVRIDTI-VVSTQH-DDF----DTEEKM-QA----KIRK-DIIEILIPRVKSK--Y-K-------KYAKL-FN--NQV--TYHINPTGKFVIGGPHGDTGLTGRKIIVDTYGGKGAHGGGAFSGKDPSKVDRSAAYATRHIAKNLVAAGVADEILVQVSYAIGVAQPTSIYVNTYGTAKVNMSDA----EISKIVTEIFDMRPYFIEQRLKLRN-P-------IYSETAAYGHMGR-KNEVVTKEFKTPDGKV----KKVKVE--LF--T-WEKL-DY-V-----PKVK----K----AFGIK---------------

>Bacteroidetes_Cytophaga_hutchinsonii

-----------MP---------------Y-LFTSESVSEGHPDKVADQISDALIDHFLAFDP-----NAKVACETLVTTGQVVLAGEVKSKA-YL--------DVQEIAREVIRKIGYTK--SE-YMFEANSCGIFSAIHEQSADINQGV-DR-KKK------------------E-EQ-GAGDQGMMFGYATNETNDYMPLALDLAHKLLIELAALRREG-KQ-IKYLRPDAKSQVTLEYD-D-N--N--R-PVRIDAI-VLSTQH-DDF----DTEEKM-HK----KIEK-DIVSILIPRIVSK--Y-P-------KYKKF-FDG-TKI--KYHINPTGKFVIGGPHGDTGLTGRKIIVDTYGGKGAHGGGAFSGKDPSKVDRSAAYATRHIAKNLVAAGLCDEVLVQVSYAIGVAKPMGIYVNTYGTAKVKKNDG----EIAKIVEKLFDMRPYAIEQRLQLRN-P-------IYSETAAYGHMGR-KPETVTKSFKSNDGKI----IKKKVE--LF--T-WEKL-DY-V-----DKVR----K----AFGIK---------------

>Bacteroidetes_Roseivirga_echinicomitans

-----------MS---------------Y-LFTSESVSEGHPDKVSDQISDALIDNFLAFDP-----DSKVACETLVTTGQVILAGEVKSKT-YL--------DVQRIARDVIERIGYTK--AE-YQFDSKSCGVLSAIHEQSQDINQGV-DR-ANK------------------E-EQ-GAGDQGMMFGYATKETENYMPLALDLSHKILQELADLRREC-KE-IPYLRPDAKSQVTIEYS-D-D--N--V-PLRIDSI-VVSTQH-DDF----AGDEEM-LA----TIRK-DIINILIPRVMAK--L-P-A-----NLQAL-FN--GQI--KYHINPTGKFVIGGPHGDTGLTGRKIIVDTYGGKGAHGGGAFSGKDPSKVDRSAAYATRHIAKNLVAAGVADEVLVQVSYAIGVVEPMGIFISTYGTAHVDMTDG----EIAKKVSEVFDMRPYAIETRLKLRN-P-------MYLETAAYGHMGR-KNEIVKKSFIGADGMS----HEREVE--LF--T-WEKL-DY-Q-----DQVK----A----AFGL----------------

>Bacteroidetes_Parapedobacter_indicus

-----------MP---------------Y-LFTSESVSEGHPDKIADQISDALIDNFLAWDA-----DSRVAIETLVTTGQVVLAGEVKSKI-YL--------DVPKIARAVIERIGYTK--SE-YMFEANSCGILSAIHEQSPDINQGV-DR-KEK------------------Q-EQ-GAGDQGIMFGYASNETENLMPLALDLAHRLLYELAALRREN-KE-ITYLRPDSKAQVTLEYD-D-N--H--K-PIRIDAI-VVSTQH-DDF----DAEEAM-LA----KIKS-DVINILVPRVKSQ--L-K-S-----ELQHL-FT--DDI--KYHINPTGKFVIGGPHGDTGLTGRKIIVDTYGGKGGHGGGAFSGKDPSKVDRSAAYATRHIAKNLVAAGVADELLVQISYAIGVKDPIGIYVNTYGTSKVALSDG----EIARKIHELFDMTPYGIETRLKLRN-P-------IYSETAAYGHMGR-EPRTVTKTFENSNGET----TSKEVE--LF--T-WEKL-DY-V-----DQVK----A----AFGL----------------

>Bacteroidetes_Wenyingzhuangia_marina

-----------MS---------------Y-LFTSESVSEGHPDKIADQISDALIDHFLAYDA-----DSKVACETLVTTGQVVLAGEVKSKA-YL--------DVQQIARDTINKIGYTK--GE-YQFDGNSCGVLSAIHEQSQDINQGV-DR-ASK------------------E-EQ-GAGDQGMMFGYATNETENFMPLALEISHRLLKELAALRREM-KD-ITYLRPDAKAQVTLQYS-D-D--N--K-PERIDAI-VVSTQH-DDF----DEDETM-LA----KIKS-DIINILIPRVKAT--F-N-Q-----EVQDL-FG--DDI--KFHINPTGKFVIGGPHGDAGLTGRKIIVDTYGGKGAHGGGAFSGKDPSKVDRSAAYATRHIAKNLVAAGVASEILVQVSYAIGVVEPMGIFVNTYGTSKVDLTDG----QIAAIVTSIFDMRPFAIETRLKLRN-P-------IYTETAAYGHMGR-TPEVKTVVFESPYEGR----KEIEVE--TF--T-WEKL-DY-V-----NQVK----E----AFNL----------------

>Bacteroidetes_Capnocytophaga_haemolytica

-----------MG---------------Y-LFTSESVSEGHPDKVADQISDALVDHFLAFDP-----QSKVACETLVTTGQVVLAGEVKSNT-YI--------DVQQIARNVIEKIGYTK--SE-YMFEANSCGVLSAIHEQSPDINQGV-DR-AKP------------------E-EQ-GAGDQGMMFGYAVNETENYMPLALDLSHALLRELAELRREN-KE-ITYLRPDAKSQVTLEYS-D-D--N--K-PVRINTI-VISTQH-DEF----DTEANM-AK----KIKA-DMINILVPRVIKK--Y-P-------QYAPF-FD--KDI--LYHVNPTGLFIIGGPHGDTGLTGRKIIVDTYGGKGAHGGGAFSGKDPSKVDRSAAYAMRHVAKNMVAAGVADEILVQVSYAIGVAQPTGVYVNTYGTAKVNLTDG----QIAEKVQQIFSLRPYDIEQRLKLRH-P-------IYSETAAYGHMGR-TPKVVTKVFNSPYHKK----KSVEVE--LF--T-WEKL-DY-V-----DKIK----A----AFGL----------------

>Bacteroidetes_Chryseolinea_flava

-----------MP---------------Y-LFTSESVSEGHPDKVADQISDALIDYFLAYDP-----SSKVACETLVTTGQVVLAGEVKSQA-YL--------DVQDIAREVIRKIGYTK--SE-YMFEANSCGIFSAIHEQSADINQGV-ER-KKK------------------E-DQ-GAGDQGMMFGYATNETDNYMPLPLELAHLLLRELSAIRREG-KV-MTYLRPDAKSQVTIEYG-D-D--N--K-PSRIDTI-VISTQH-DEF----GKDAAM-LK----QIKE-DVINIVVPRIKKK--L-P-K-----RVQNL-FG--DDI--KYHVNPTGKFVIGGPHGDTGLTGRKIIVDTYGGKGAHGGGAFSGKDPSKVDRSAAYATRHIAKNLVAAGLCDEVLVQVAYAIGVAKPVGLYVNTYGTAKTSLSDG----EIAEKVAKIFDMRPYFIEERFGLRT-P-------IYAETAAYGHMGR-EPKTVEKIFNKGKNNE----KKVKVE--LF--P-WEKL-DF-V-----DQVK----K----TFKLN---------------

>Bacteroidetes_Lewinella_agarilytica

-----------MP---------------Y-LFTSESVSEGHPDKVADQISDNLVDHFLAFDP-----SSKVACETLVTTGQVVLAGEVKSKT-YL--------DVQQLARDVIKRIGYTK--SE-YMFEANSCGVLSAIHEQSPDINQGV-ER-TKA------------------E-DQ-GAGDQGMMFGYATNETENYMPLALDLSHRILQELAALRKAG-KK-MKYLRPDSKSQVTIEYD-D-N--H--K-PIRIDSI-VVSTQH-DDF----DVDKKM-LA----QIKE-DVISIVIPKVKKG--L-K-K-----NIQKL-FT--KDI--TYHVNPTGKFVIGGPHGDTGLTGRKIIVDTYGGKGAHGGGAFSGKDPSKVDRSAAYATRHIAKNLVAAGVADEILVQVSYAIGVAKPTNIYVNTNGSAKVKMTDA----QIAKKVAQVFDMRPYAIEKRLKLRK-P-------IYSDTAAYGHMGR-TPEKKKLTFVDGSGKS----KTMNVE--TF--T-WEKL-DY-V-----SKVK----K----AFKL----------------

>Bacteroidetes_Polaribacter_irgensii

-----------MS---------------Y-LFTSESVSEGHPDKVADQISDALIDNFLAFDK-----NSKVACETLVTTGQVFLAGEVKSKT-YL--------DVQQIARDVINKIGYTK--SA-YMFDGNSCGVLSAIHEQSPDINRGV-DR-SSP------------------E-EQ-GAGDQGMMFGYATDETDNYMPLALELSHRLLIELALLRREN-SD-IDYLRPDAKSQVTIEYS-D-E--N--V-PQRIDAI-VISTQH-DDF---DPSDAVM-LA----RIKK-DLIGILIPRVVAK--L-P-A-----DIQKL-FT--DNI--TYHINPTGIFVIGGPHGDTGLTGRKIIVDTYGGKGGHGGGAFSGKDPSKVDRSGAYATRHIAKNLVAAGLCKEVLVQVSYAIGVATPTSINVETYGTATVALTDG----EISKKVATLFDMRPYFIEQRLKLRT-P-------IYSETAAYGHMGR-TPEIKIVTFSNPMGET----VSEEVE--TF--T-WEKL-DY-V-----DKVK----A----AFKL----------------

>Bacteroidetes_Ekhidna_lutea

-----------MP---------------Y-LFTSESVSEGHPDKIADQISDSLLDHFLAFDP-----DSKVACETLVTTGLVTLAGEVKTTA-YV--------DVQNIARDVINRIGYTK--GE-YMFDGNSCGVISAIHEQSPDINQGV-DR-DSE------------------D-EQ-GAGDQGMMFGYATTETDNYMPLALNISHSILMELAAIRKEG-KE-MTYLRPDAKSQVTIEYD-D-N--N--H-PVRIDAV-VVSTQH-DEF----DNEDNM-LK----KIKQ-DVINILIPRVIAK--M-K-P-----ENQEL-FN--DQI--KYHINPTGKFVIGGPHGDAGLTGRKIIVDTYGGKGAHGGGAFSGKDPSKVDRSAAYATRHIAKNLVAAGVANEVLVQVSYAIGVAEPMGIYVNTYGTANVDMNDG----EIAKIVEKIFDMRPAYIIKRLKLKE-P-------IYSESAAYGHMGR-APEVKQVIFKGNGGDE----VKKVVE--TF--T-WEKL-DY-V-----DQVK----E----AFKL----------------

>Bacteroidetes_Empedobacter_brevis

-----------MS---------------Y-LFTSESVSEGHPDKIADQISDSILDHFLAFDA-----KSKVACETLVTTGQVVLAGEVKSNS-YI--------DLQRVARETISEIGYTK--SE-YMFDSNSCGILSAIHEQSPDINQGV-DR-SSE------------------E-EQ-GAGDQGMMFGYATSETENYMPLALDLSHRLLKELAHIRKYE-KDLLPYLRPDAKSQVTIEYD-N-D--N--K-PVRIDTI-VISTQH-DDF----ANEEAM-RD----KITN-DLISILIPRVKAQ--L-P-V-----HLRDL-FT--ENI--TYHINPTGKFVIGGPHGDTGLTGRKIIVDTYGGKGAHGGGAFSGKDPSKVDRSAAYAARHIAKNLVAAGVAEEILVQVSYAIGVAAPVSLLVNTFGTQKVNLTEG----EISKKVSEIFDMKPFAIEQRLKLRN-P-------IYKETASYGHLGR-EPKTVSKRFESANGQD---VVEVEVE--LF--T-WEKL-DF-V-----DQIK----E----AFGL----------------

>Bacteroidetes_Thermoflavifilum_aggregans

-----------MP---------------Y-LFTSESVSEGHPDKLADQISDALVDHFLAFDP-----DAKVACETLVTTGQVVLAGEVKSSV-YL--------DVQKIAREVIRKVGYTK--SD-YMFDANSCGILSAIHEQSPDINRGV-ER-QDK------------------E-EQ-GAGDQGLMFGYAVNETEDYMPLPIYLAHLLLKELSAIRREG-EV-MKYLRPDAKSQVTIAY--D-N--R--K-PVRIDTI-VVSTQH-DEF----DTDERM-AA----RIRK-DVLEILIPRVKGK--L-S-P-----DVQQL-FT--DHI--TYHINPTGKFVIGGPHGDTGLTGRKIIVDTYGGKGAHGGGAFSGKDPSKVDRSAAYATRHIAKNMVAAGLCDEVLVQVSYAIGVARPCGFFVNTFGTAKVPLSDE----EIAARIIELFDMRPYAIEQRLKLRN-P-------IYQETASYGHMGR-KPQTVVKKFYSPDRGI----LEREVE--LF--T-WEKL-DY-V-----DLIK----E----AFKLPK--------------

>Bacteroidetes_Apibacter_mensalis

-----------MP---------------Y-LFTSESVSEGHPDKVSDQISDSILDNFLAFDP-----DSKVACETLVTTGQVILAGEIKSNT-YI--------DVQQVTRNIIKKIGYTK--SE-YKFEAESCGILSALHEQSQDINRGV-DK-QNK------------------E-EQ-GAGDQGMMFGYATNETDNYMPLALDISHSILRVLARIRKEG-KI-MTYLRPDAKSQVTIEYD-D-N--N--K-PIRIDTI-VLSTQH-DMF---DEDEQVV-LK----KIAK-DIITIVIPEVKAE--S-K-D-----EIKAL-FD--DNI--TYHINPTGKFEIGGPHGDTGLTGRKIIVDTYGGKGAHGGGAFSGKDPSKVDRSAAYATRHIAKNLVAAGVADEVLVQVSYAIGITEPCGIYINTFGSSKVNLSDG----DIALKVQKLFDLRPYAIEKRLKLRN-P-------IYTETAAYGHMGR-KNETVIKKFERNSENGKTEIKEIEVE--LF--T-WEKL-DY-V-----DKIK----E----AFNL----------------

>Bacteroidetes_Brumimicrobium_aurantiacum

-----------MS---------------Y-LFTSESVSEGHPDKVADQISDSLIDHFLAFDK-----DSKVACETLVTTGLVTLAGEVKSNI-YL--------DVQKIARDTIEKIGYTK--SE-YQFDAKSCGVISAIHEQSEDINRGV-DK-ENP------------------E-DQ-GAGDQGMMFGYATNETEDYMPLALDLSHRLLIELAELRREN-DE-ITYLRPDAKAQVTIEYS-D-D--N--K-PQRIDAI-VLSTQH-DDF---DADEKVM-LD----KINK-DVKEILIPRLIKK--L-NNP-----LVEEL-FK--GNI--TYHINPTGKFVIGGPHGDTGLTGRKIIVDTYGGKGAHGGGAFSGKDPSKVDRSGAYATRHIAKNMVAAGLCEEVLVQVSYAIGVAEPMGLYVNTYGTSKVDMTDG----DIAREISKMFDMRPAAIEKRLKLRN-P-------IYSPAAAYGHMGR-KPETKTVTFENGGRTE-----TLEVE--FF--T-WEKL-DF-V-----DQIK----S----TFGL----------------

>Bacteroidetes_Flavobacterium_aurantiibacter

-----------MA---------------Y-FFTSESVSEGHPDKIADQISDALIDHFLALDP-----QSKVACETLVTTGQVVLAGEIKSDI-YL--------DVQKITREVIRKIGYTK--SE-YKFDAESCGILSALHEQSADINQGV-ER-KDP------------------K-EQ-GAGDQGMMFGYATNETADYMPLALDLSHKLLQELAELRREN-NE-IKYLRPDAKSQVTLEYD-D-N--N--K-PSRIHTI-VLSTQH-DDF---NADETVM-QK----QIEA-DVKNILVPRILNK--Y-P-------EYAHL-FN--NEI--TYHVNPTGKFVIGGPHGDTGLTGRKIIVDTYGGKGAHGGGAFSGKDPSKVDRSAAYATRHIAKNLVAAGVASEVLVQVSYAIGVARPMAVYVNTYGTATVSLNDA----QIAEKVQELFDLTPYGIETSLELRN-P-------IYQETAAYGHMGR-RPETKEKTFLGPDGKL----LTKTVN--LF--T-WEKL-DR-V-----NDIK----Q----AFGL----------------

>Bacteroidetes_Labilibaculum_manganireducens

-----------MG---------------Y-LFTSESVSEGHPDKVADQISDALLDKFLAFDP-----NSKVACETLVTTGQVVLAGEVKTKT-YI--------DAQEVAREVINRIGYTK--SA-YQFDGTSCGVLSAIHEQSADINRGV-DR-EDP------------------M-TQ-GAGDQGMMFGYACKETDDYMPLALELSHRLLIELAAIRREG-RE-MTYLRPDSKSQVTIQYA-D-D--D--T-IERVHTI-VVSTQH-DDF---GPNDELM-LA----KIKK-DVRNILIPRVIKQ--L-P-K-----RIQAM-FD--ENF--ILHVNPTGKFVIGGPHGDTGLTGRKIIVDTYGGKGAHGGGAFSGKDPSKVDRSAAYAARHIAKNLVAAGVADELLVQLSYAIGVAEPVGIYVNTYGKSNLSLTDG----QIADKVTELFDLRPAAIEQRLKLRN-P-------IYEETAAYGHMGR-TPRMVVKKFESPYFDG----VELEVE--LF--T-WEKL-DY-I-----EKVK----E----AFNL----------------

>Bacteroidetes_Ichthyobacterium_seriolicida

-----------MS---------------Y-LFTSESVSEGHPDKIADQISDAILDRFLAFDK-----DSKVACETLVTTGQVFLAGEVKSNI-YV--------DVQSTARDVINKIGYTK--GE-YQFDGNSCGVISSIHEQSQDINRGV-DK-TIR------------------E-DQ-GAGDQGIMFGYATDETETYIPLPLFISHLILKELASIRREN-KE-ITYLRPDSKAQVTIEYS-D-D--K--E-PIRIDSI-VISTQH-DEF---DSDDVKM-LE----IIKN-DMIGILIPRVKKT--L-N-K-----KIQLL-FD--DNI--KYYINPTGKFVIGGPHGDTGLTGRKIIVDTYGGKGAHGGGAFSGKDSSKVDRSATYAARHIAKNLVASGVAREVLVQLSYAIGIKEPMGVYVNTYGTSKLDISDS----EIANKVKEIFSLTPFSIEERLKLRN-P-------IYLETASYGHMGR-DPQIVEKIFESPYEGK----IQLKVE--LF--T-WEKL-DY-V-----EKIK----R----AFSI----------------

>Bacteroidetes_Ornithobacterium_rhinotracheale

-----------MS---------------Y-FFTSESVSEGHPDKVADQISDAILDHFLAFDS-----QSKVACETLVTTGQVILAGEVNSKA-YV--------DLQEITRKVIGRIGYND--SQ-MNFTADSCGVLSAIHEQSPDIRQGV-VQ-SEK------------------E-NQ-GAGDQGMMFGYATNETENFMPLALDLSHRILLELADIRKNG-TE-MPYLRPDAKAQVTLEYS-D-D--N--K-PTSIHTI-VVSTQH-DEF----GDERAM-QE----RIRR-DVIQIVIPRVIQK--L-P-T-----HIKEL-FT--SDI--TYHINPTGKFVIGGPHGDTGLTGRKIIVDTYGGKGAHGGGAFSGKDPSKVDRSAAYAMRHLAKNLVAAGVCEEALVQVSYAIGVAGPMSLCIETYGTSKVDLSDA----EIAQKLLEKYDFRPYAIEEKFKLRN-P-------IYEETAAYGHMGR-EPRTVKKVFEGNGRDK----VECEVE--LF--T-WEKL-DI-V-----DELK----A----LFELK---------------

>Bacteroidetes_Thermophagus_xiamenensis

-----------MG---------------Y-LFTSESVSEGHPDKVADQISDAVLDELLRQDP-----DSKVACETLVTTGLVVVSGEINTKG-YV--------AVQQVARETIERIGYTK--AD-YMFDSGSCGVISALHEQSDDIYRGV-ER-DGE------------------D-KQ-GAGDQGMMFGYACRDTDNFMPLPLYLSHFLLQELAAIRREG-KE-MTYLRPDSKSQVTIEYD-D-N--N--N-PIHVDTI-VISTQH-DPF----DPDDQV-MH----DVIERDVQNILIPRVKDK--L-K-P-----ELQKL-F---NGY--KLHVNPTGRFVIGGPHGDTGLTGRKIIVDTYGGKGAHGGGAFSGKDCSKVDRSAAYATRHIAKNMVAAGVADEVLVQVAYAIGVAEPVGLYVNTYGTSKVKMSDG----EIAQKIQEIFDLRPGKIIKRLGLKN-P-------IFTPTAAYGHMGR-EPYTAKVRFKENGQE-----VEREVE--FF--T-WEKL-DY-V-----DTIR----K----TFGL----------------

>Bacteroidetes_Geofilum_rubicundum

-----------MG---------------Y-LFTSESVSEGHPDKVADQISDAVLDELLRQDA-----HSRVACETLVSTGLVVVSGEIKTNG-YA--------AVQQVARRVIERIGYTK--AE-YMFDSGSCGVLSALHEQSEDINRGV-DR-ESE------------------E-TQ-GAGDQGMMFGYACRDTEEFMPLALQLSHLLLIELAAIRREG-KE-MTYLRPDSKSQVTLEYS-D-D--N--Q-PVRVHTI-VVSTQH-DPF----DADAPM-LA----KIKE-DVQNILIPRVKKK--L-N-T-----SIAGL-F---ADY--ILHVNPTGKFVIGGPHGDTGLTGRKIIVDTYGGKGAHGGGAFSGKDSSKVDRSAAYAARHMAKNMVAAGLADEVLVQLAYAIGVAQPVGVYVNTYGTAKVGLHDG----EIAKRIEKLFDLRPGMIIRRLGLKN-P-------IFEPTAAYGHMGR-APYTDKVVFIENGQE-----VEKEVE--FF--T-WEKL-DY-V-----EAIQ----K----AFDL----------------

>Bacteroidetes_Williamwhitmania_taraxaci

-----------MG---------------Y-LFTSESVSEGHPDKVADQISDGILDEFLRRDP-----ECKVACETLTTTGLIVISGEVRAEA-YV--------DVQDVARRVVNRIGYTK--AE-YKFDGNSCGIISAIHEQSVDINRGV-VK-ASP------------------E-EQ-GAGDQGMMFGYACKETDDYMPLSVVLSHVLLMELAKIRREG-KE-MTYLRPDAKSQVTIEYG-D-D--N--K-IKRVHTI-VISTQH-DEF----DTDEKM-QA----QIKK-DVQNILIPRVKAH--M-Q-T-----SVQKY-FD--NNI--ILHVNPTGKFVIGGPHGDTGLTGRKIIVDTYGGKGAHGGGAFSGKDPSKVDRSAAYAVRHIAKNMVAAGVADEMLVQVAYAIGVAQPVSIFVNCYGTAHVKFADE----VIAEKISQLFDLRPGMLIDRLMLKN-P-------IYEETAAYGHFGR-DHKVAPVKFMVNGRE-----VVRNVE--FF--T-WEKL-DY-V-----DKVK----A----EFGL----------------

>Bacteroidetes_Balneicella_halophila

-----------MS---------------Y-LFTSESVSEGHPDKIADQISDALLDEFLRRDP-----EAKVACETLVTTGLVVLSGEVNTKD-YV--------AVEKFVRETIGEIGYTN--SD-YQFEANSCAVISAIHPQSPDINRGV-ER-QYP------------------E-EQ-GAGDQGMMFGYASKDTDDYLPLSLDLSHILLQELAKIRREG-EV-MTYLRPDAKAQVTIEYS-D-D--N--V-PQRIDTI-VVSTQH-DEF---DNDDERM-LE----KIRE-DVKKYLIRRAKKR--L-P-E-----RVQKL-FK--EDY--KLFVNPTGKFVIGGPHGDTGLTGRKIIVDTYGGKGAHGGGAFSGKDASKVDRSAAYAARHIAKNMVAAGVAEEVLVQVAYAIGVAEPVGFYVNTYGKANVDLTDG----EIAQKVQELFDMRPYSIIERFGLKY-P-------IFKETAAYGHVGR-KYR---KDFVKLKYDGVD--SEKEVE--FF--A-WEKL-DY-V-----SKLK----E----SFNL----------------

>Bacteroidetes_Longibacter_salinarum

-----------MA---------------Y-LFTSESVSEGHPDKVADQISDAILDALLSDDP-----KSRVAVETMVTTGLVVLSGEVSTDA-YA--------DVREIAREVIRDIGYTD--PR-LRFDADSCGVLSSIHEQSPDISQGV-DG-TS---------------------EQ-GAGDQGLMFGYANRETDVLMPMAIMYAHRLVRELAHIRKET-DL-MPYLRPDSKSQVTVEYD-D-D--R--RTPRRVHTI-VVSTQH-DEG--------VS-QS----EIKR-DIREHLLPRVIA---------------DDM-LD--DDV--ILHVNPTGRFVIGGPHGDTGLTGRKIIVDTYGGKGAHGGGAFSGKDPSKVDRSATYAARHVAKNLVAADLCDEALVQVAYAIGVAEPVSIDVNTNGTGK--LTDD----QLTNLVRDHFELSPTAIIDRLDLMR-P-------MYRKTAAYGHFGR--------------------------D--TF--P-WEKT-NY-V-----DALK----H----AAKQLA--------------

>Bacteroidetes_Chloroherpeton_thalassium

---------M-SR---------------F-LFTSESVSEGHPDKVSDQISDAILDEFLKQDP-----NSRVACETFVTTGLVVVGGEITSTA-NV--------DVQSLVRKVVKQIGYTK--AE-YMFEADSCGVLSALHAQSPDINRGV-DR-KEEIA-------------DPFD-RI-GAGDQGIMFGFACNETPELMPAAIQYAHKLVKMLADIRKEC-KE-MTYLRPDSKSQVTLEY--V-D--G--K-IARVDTA-LISTQH-DPEPA-GVSEADW-QK----KIAE-DVINKVMKVVIP---------------AEY-ID--EKT--KFIVNPTGRFEIGGPHGDTGLTGRKIIVDTYGGAAPHGGGAFSGKDPSKVDRSAAYAARYIAKNVVAAGLADRCTIQVSYAIGIARPVSIFVDTHGTAANGLQDS----EIQEKVEKVFDLRPAALMKNFGLNK-P---EGWPCFQDTAAYGHFGR--------------------------D--IF--P-WEKL-DK-V-----EELK----S----AFK-----------------

>Bacteroidetes_Chlorobium_ferrooxidans

-----MS--Q-TR---------------Y-FFTSESVSEGHPDKVADQISDAVLDDFLRQDA-----NSRVACETFVTTGQVIVGGEVTTKG-IV--------DIQKIARQVVSEIGYTK--GE-YMFEANSCGVLSALHSQSPDINRGV-DR-KEEIA-------------DEFD-RV-GAGDQGMMFGYACTETPELMPAAIQFAQQLVKKLAEIRKEG-KI-MTYLRPDAKSQVTLEY--E-N--E--K-VKRVDAV-VVSTQH-DPEPA-GVSEAEW-QA----VIKK-DIIENVINKVIP---------------ANL-LD--ANT--KLHINPTGRFEIGGPHGDTGLTGRKIIVDTYGGAAPHGGGAFSGKDPSKVDRSAAYASRHVAKNIVAAGLADKCTVQVSYAIGVARPVSIYINTHDTAKQGLNDA----QIQEKAELIFDLRPAAIIKHFGLNK-P---QGW-SYQQTAAYGHFGR--------------------------D--IF--P-WEKT-DK-V-----AELK----K----AFNLA---------------

>Chlamydiae_Waddlia_chondrophila

------------M------------SH-F-LFTSESVSIGHPDKIADQISDAILDACLKADP-----DSKVACETLVSTGLVVLAGEITTKT-QL--------DYQEIVRKTIKRIGYNS--SE-LGFDYRSCGVILSINKQSPDISQGV-EE-GKGL----FK-------------EQ-GAGDQGIMFGFACDETPELMPLPIMLSHKIVLELQRLREEN--A-LSYLRPDAKAQVTLEY--D-E--G-FI-PRRIHTV-VVSTQH-DPGVD---------LD----FLKK-DMIGMVNR-------LAP-E-------G-F-ID--SET--KYFINPTGRFVLGGPVADCGLTGRKIVVDTYGGMARSGGGALSGKDPSKVDRSASYAARYVAKNIVAAGFAKRCEVQVSYAIGVAEPVSIKVDTFGTGV--VSEQ----MLAEAIFKTFDLSPKGIIEMLDLKR-P-------IYSETAYGGHFGR------------------------KGD--AF--T-WEKT-DR-V-----EKLR----E----AVELQATNA-----------

>Chlamydiae_Criblamydia_sequanensis

------------M------------TH-Y-LFTSESVSVGHPDKIADQISDAILDACLSQDK-----DSRVACEALVGLGLVVLAGEVTTKA-IV--------DFQAVARETIREIGYDD--PS-LGFDYKSCAVLTSIHQQSQDIAVGV-DK-TSS------K-------------EL-GAGDQGIMFGYACDETPELMPMPIMLAHAIVRKLKSLRESS--E-LNYLKPDAKSQVTIEY--D-N--A-HK-PKRLHSV-VISTQH-SPDVK---------QE----ILTR-DLKRLTEE-------IAP-S-------G-F-ID--SNT--QFFINPTGRFVIGGPVGDTGLTGRKIIVDTYGGMGRHGGGAFSGKDPTKVDRSASYAARYVAKHIIAAKLAKRCEVQLSYAIGVSHPISIKVDTFGTGK--VDEN----LIAEAIPLVFNLSPQGIIDMLDLKR-P-------IYKKTAFGGHFGR------------------------S-E--KF--S-WEQV-DK-I-----ENLK----E----AIKHRSFTH-----------

>Chlamydiae_Estrella_lausannensis

------------M------------AH-Y-LFTSESVAAGHPDKVADTISDSILDAYLKEDP-----NSRVACETLVTTGLVVLAGEISSKA-HI--------DHRKVVREAIRDIGYDD--PS-LGFDYQSCGILTTISEQSTDIAAGV-DE-AEG------K-------------EM-GAGDQGIMFGYACDETKELMPLPIMLAHSIVRELRALRESK--D-LDYLRPDAKSQITVEY--D-R--N-DR-PVRLNSV-VISTQH-SPKVK---------QE----TLVH-DMTALVHQ-------LAP-H-------G-F-ID--ENT--LFYINPTGRFVIGGPAGDTGLTGRKIIVDTYGGMGRHGGGAFSGKDPTKVDRSASYAARWVAKNIVGSGLARRCEVQISYAIGVAYPLSIKVNTFGTGQ--VPES----LLAHVIPLVFDLSPKGIIESLNLRR-P-------VYKATASGGHFGR------------------------E-E--GF--T-WERL-DK-V-----EKIK----E----AIRQAAYTTEG---------

>Chlamydiae_Chlamydia_trachomatis

--------------------------M-K-LFTSESVTEGHPDKVCDQISDSILDAMLAHDP-----HARVAVETMVTTGLVHVAGEVTTNT-YV--------EIPELIRRVLKEIGYVS--SE-IGFDADSCGISLSIGQQSHDIAQSV-DD-SLEVR-Q----GHIA---DRFD-KQ-GAGDQGMMFGYASNETPTFMPMPIFLAHKLAQRLAQVRKEL--V-VPGLRPDGKTQVTVAY----D-SN-DV-PVGIDTV-VVSSQH-APNIE---------LE---A-LQH-EITANVIDPV-----M-A-QYAPNFAYEHS------------FVNPSGRFVIGGPMGDAGLTGRKIIVDTYGGFARHGGGAFSGKDPSKVDRSAAYAARWVAKNVVAAGLADRCEVQVAYAIGRAHPVSLRLDTFGTEH--TALC----RIERAVNTVFDLRPAAIIESLDLLR-P-------IYAQTATYGHFGR-E----------------------STD---F--T-WERT-NR-I-----EDLL----A----AA------------------

>Chlorobi_Chloroherpeton_thalassium

---------M-SR---------------F-LFTSESVSEGHPDKVSDQISDAILDEFLKQDP-----NSRVACETFVTTGLVVVGGEITSTA-NV--------DVQSLVRKVVKQIGYTK--AE-YMFEADSCGVLSALHAQSPDINRGV-DR-KEEIA-------------DPFD-RI-GAGDQGIMFGFACNETPELMPAAIQYAHKLVKMLADIRKEC-KE-MTYLRPDSKSQVTLEY--V-D--G--K-IARVDTA-LISTQH-DPEPA-GVSEADW-QK----KIAE-DVINKVMKVVIP---------------AEY-ID--EKT--KFIVNPTGRFEIGGPHGDTGLTGRKIIVDTYGGAAPHGGGAFSGKDPSKVDRSAAYAARYIAKNVVAAGLADRCTIQVSYAIGIARPVSIFVDTHGTAANGLQDS----EIQEKVEKVFDLRPAALMKNFGLNK-P---EGW-CFQDTAAYGHFGR--------------------------D--IF--P-WEKL-DK-V-----EELK----S----AFK-----------------

>Chlorobi_Chlorobium_phaeovibrioides

-----MS--Q-SR---------------Y-FFTSESVSEGHPDKVSDQISDAVLDSFISQDP-----NSRVACETFVTTGQVIVGGEVTTTG-IV--------DIQKIARRVITEIGYTK--GE-YMFDANSCGVLSALHSQSADINRGV-DR-KEEIA-------------DEFD-RV-GAGDQGMMFGYACTETPELMPAAIQYAQRLVKLLAEIRKAG-KI-MTYLRPDSKSQVTLEY--I-D--D--K-VARVDAV-VVSTQH-DPEPA-GMSEAEF-QA----VIRK-DIIENVINKVIP---------------AEL-LD--ADT--KFHINPTGRFEIGGPHGDTGLTGRKIIVDTYGGAAPHGGGAFSGKDPSKVDRSAAYASRHVAKNIVAAGLADKCTVQVSYAIGVARPVSIYINTHDTAKHGLTDE----QIQEKAEKIFDLRPLAIIRRFNLDK-P---EGW-SYQETAAYGHFGR--------------------------E--NF--P-WEKT-EK-V-----EELK----K----AFNLA---------------

>Chlorobi_Pelodictyon_luteolum

-----MS--Q-SR---------------Y-FFTSESVSEGHPDKVADQISDAVLDDFIRQDP-----NSRVACETFVTTGQVIVGGEVTTTG-IV--------DVQKIARKVITEIGYTK--GE-YMFEANSCGVLSALHSQSADINRGV-DR-KEAIA-------------DEFD-RV-GAGDQGMMFGYACTETPELMPAAIQFAQQLVKVLADIRKAG-KI-MTYLRPDAKSQVTLEY--V-D--E--K-VARVDAV-VVSTQH-DPEPA-GMSEAEF-QE----VIKK-DIIENVIKKVIP---------------ANL-LD--LNT--KFHINPTGRFEIGGPHGDTGLTGRKIIVDTYGGAAPHGGGAFSGKDPSKVDRSAAYASRHVAKNIVAAGLAEKCTVQVSYAIGVARPVSIYINTHGTAMHGLSDE----QIQEKAEKIFDLRPLAIIRRFSLDN-P---QGW-CYQETAAYGHFGR--------------------------D--IF--P-WEKT-EK-V-----AELK----T----AFSLA---------------

>Chlorobi_Chlorobaculum_parvum

-----MS--H-SR---------------Y-FFTSESVSEGHPDKVSDQISDAVLDEFIKQDP-----NSRVACETFVTTGQVIVGGEVTTKG-IV--------DIQTIARKTITEIGYTK--GE-YMFDANSCGVLSALHSQSPDINRGV-DR-KEEIE-------------DEFD-RV-GAGDQGMMFGYACTDTPELMPAAIQYAQELVRLLAEIRKEG-KI-MTYLRPDSKSQVTLEYD-E-N--D--K-VLRVDAV-VVSTQH-DPEPA-GMSEAEF-QE----VIKN-DIIENVIRKVIP---------------AEL-ID--ENT--KFHINPTGRFEIGGPHGDTGLTGRKIIVDTYGGAAPHGGGAFSGKDPSKVDRSAAYAARHVAKNIVAAGLADKCTVQVSYAIGVARPVSIYINTHGTGKHGLSDS----QIQEKAEAIFDLRPLAIIRRFNLDR-P---HGW-CYRDTAAYGHFGR--------------------------E--QF--P-WEKT-EK-V-----AELK----A----AFGL----------------

>Chloroflexi_Herpetosiphon_aurantiacus

MDATTF---M-RA------------PQ-F-FFTSESVTEGHPDKICDQVSDAILDELLAQDP-----MSRVACETATTTGLIVVLGEVTTKG-YV--------EVQDIVRKVVSDIGYTR--GK-FGFDAETCGIIVALHGQSPDIAQGV-DV-ALEVR-D--D-QASRE--AEIE-KV-GAGDQGMVFGFACNETDEFMPLTISLAHQLTRRLAKVRKTG--E-VGYLRPDGKSQVTVEY--S-H--G--K-PVRVDTV-LISTQH-DPNVD---------NE----RIHK-DVIELVIKPV-----I-P-E-------G-M-LD--ENT--KIFINPTGRFVTGGPMGDSGLTGRKIIVDTYGGVARHGGGAFSGKDSTKVDRSAAYAARYVAKNIVAAGLAERFELQVSYAIGVSKPLSISFETFGTAK--VSDE----KLLELINKHFDLRPGAIIRDLDLRR-P-------IYRQTAAYGHFGR-A----------------------DID---L--P-WERT-DK-A-----ELLR----A----ESGL----------------

>Chloroflexi_Kouleothrix_aurantiaca

-MPTSF---M-QS------------PQ-L-FFTSESVTEGHPDKLCDQVSDAILDAFLEKDP-----RSRVACETSATTGLVLVMGEVTTEA-YV--------EIQEVVRRTIRDFGYTG--TG-VGFDADTCGVIVALHGQSADIAMGV-DK-ALEAK-Q--G-AMT-E--AEIE-AV-GAGDQGMMFGFACNETPELMPLTISLAHKLTRELAVARKSG--Q-IAYLGPDAKSQVTVEY--S-H--G--Q-PVRVDTL-LISTQH-AADVS---------QE----QIRE-DVIEDIFHKV-----V-P-A-------E-L-ID--GST--KYYVNPTGRFVTGGPHGDAGLTGRKIIVDTYGGVARHGGGAFSGKDPTKVDRSAAYAARWVAKNVVAAGLADRFELQLAYAIGVARPLSVSVETFGTNK--VAEE----TIVKLINEHFDLRPGAIIRDLDLRR-P-------IYRQTAAYGHFGR-T----------------------DVD---L--P-WERT-DR-A-----DALR----R----AAGL----------------

>Chloroflexi_Ardenticatena_maritima

-MSTTF---M-SS------------PK-L-YFTSESVTEGHPDKICDQISDAILDAILAQDP-----DARVACETATTTGLIAIIGEITTSA-NV--------DYTEIARETLRQIGYTS--SE-YGFDADTCAVIVSIKKQSADIAMGV-DK-ALEAK-T--G-EMS-D--AEIE-AI-GAGDQGMMVGFACNETPELMPLSISLAHRLTRRLTEVRKSG--L-LGYLRPDGKSQVTVEY--A-Y--G--K-PVRVDTV-LISTQH-APDVT---------QE----QIRA-DIIEHVIRPV-----V-G-E-------K-L-LD--ENT--KIYVNPTGRFVVGGPMGDAGLTGRKIIVDTYGGVARHGGGAFSGKDPTKVDRSGAYMARYVAKNIVAAGIADRFEVQISYAIGVARPLSISVETFGTGR--IPDD----KIVDLINQFFDLRPAAIIRDLDLRR-P-------IYKQTAAYGHFGR-E----------------------DVD---L--P-WERT-DK-A-----DALR----A----AAGLDTVPS-QA--------

>Chloroflexi_Bellilinea_caldifistulae

-MNTTF---M-NS------------PR-Y-LFTSESVTEGHPDKLCDQVSDAVLDACLEQDP-----YSRVACEAATKTGFVLVFGEITTRA-QI--------NFDELVRRVVNEIGYDS--SE-KGFDGTTCAVQVAISKQSGDIAMGV-DK-ALEAK-T--G-SMS-E--REIE-AV-GAGDQGMMFGYACNETPTLMPMPIYLAHKLTRRLAEVRKNG--T-LPWLRPDGKSQVTIEY--R-F--G--R-PHRVDTV-LISTQH-APEIS---------QE----EIYQ-AITRHVIYPV-----L-P-P-------E-M-VD--DQL--KIYVNPTGRFVIGGPMGDSGLTGRKIIVDTYGGMGRHGGGAFSGKDPTKVDRSAAYAARWVAKNIVAAGLAERVEIQVAYAIGVARPLSINVETFGTGT--IPDE----EIAQLINEHFDLRPGAILRDLDLRR-P-------IYRQLAAYGHFGR-D----------------------DLD---L--T-WERT-NK-A-----EALK----Q----AVAAKLATA-AD--------

>Chloroflexi_Leptolinea_tardivitalis

-MQSTF---L-NS------------EK-Y-LFTSESVTEGHPDKICDQVSDAVLDACLAQDP-----YSRVACETATKTGFVMLLGEITTNA-HI--------DFDRLVRKVVTDIGYDD--SS-KGFDGNSCGVQVAIAKQSGDIDMGV-SK-ADEVK-K--G-QAS-E--DEIA-KI-GAGDQGMMFGFACNETPALMPMPIYLAHKLTRKLTEVRKND--T-LPWLRPDGKSQVTIEY--R-K--G--K-PYRVDTV-LISTQH-DPDVT---------QK----EIFD-GVKKYVIDPV-----L-K-E-------SGI-ID--GDL--KIYVNPTGRFVIGGPMGDAGVTGRKIIVDTYGGTGRHGGGAFSGKDPTKVDRSAAYAARWVAKNVVAAGLADRCEIQVAYAIGVAHPLSINVETFGTGK--IADE----DIAEIISKTFDLRPGAIIRDLDLRK-P-------IYQQVAAYGHFGR-D----------------------DLD---L--S-WEKT-NK-V-----EALK----A----AAAAKASK------------

>Chloroflexi_Litorilinea_aerophila

--MTTF---M-TA------------PK-L-LFTSESVTEGHPDKMCDQISDAVLDAVYAQDP-----YGRVACETAIKTGFVMLLGEITTTA-EL--------NYDQLVRQVIRDIGFDC--SK-KGFDGNTCGVQVAIAAQSPDIAMGV-DK-ALEAK-E--G-QME-E--DELE-TI-GAGDQGMMFGFACDETPTLMPMPIYLAHKLARRLSQVRKEG--I-LPWLRPDGKTQVTVEY--A-F--G--K-PKRIHTV-LISTQH-DPDVT---------QS----EIRQ-ALIEHVIDPV-----L-P-Q-------E-L-VD--KDL--RIFTNPTGRFVVGGPMGDAGLTGRKIIVDTYGGMGRHGGGAFSGKDCTKVDRSAAYAARWVAKNIVAAGLARRCEIQLAYAIGVARPLSINVETFGTAQ--ISDE----QIVRLIEEHFDLRPGAIIRDLGLRR-P-------IYRQTAAYGHFGR-D----------------------DIE---L--P-WEDT-SR-A-----DALR----R----AAGLEEMAH-A---------

>Chloroflexi_Flexilinea_flocculi

--MSTF---M-NS------------PK-F-LFTSESVTEGHPDKMCDQISDAVLDACLAQDP-----FSRVACETATKTGYVMVFGEITTKA-FV--------NFDELVRKIVLSIGYDS--SE-KCFDGHTCGIISAISTQSPDIALGV-DK-SLEAK-K--G-ELN-G--SGTE-DV-GAGDQGMMFGYACNETDVLLPLPIFLAHRLAEKLTEVRKSG--V-LPWVYPDGKSQVTVEY--A-Y--G--K-PRRIDTV-VISTQH-SDSMR---------NKH--DEIEA-AIKKYVIDPI-----L-P-K-------E-L-VD--SDM--KVYVNPTGEFIVGGPQGDSGLTGRKIIVDTYGGMGRHGGGCFSGKDCTKVDRSAAYAARWVAKNIVAAGIADRCEIQLSYAIGVSKPISINVETFGTGK--ISDE----KISEIIHANFDLRPGAIIRDLNLRR-P-------IYQKTASYGHFGR-E----------------------DDS---F--T-WEKT-NK-A-----ETLR----K----AAGL----------------

>Chloroflexi_Anaerolinea_thermophila

--MDSL---M-ES------------PK-Y-LFTSESVTEGHPDKLCDQISDAVVDACLEQDP-----TSRVACETAAKTGFVMLLGEVTTKA-II--------NYEELARDVIKDIGYDH--TD-KGFDYNTCGVLVSICKQSPDIDMGV-SK-AKEAK-E--G-KMD-----DIE-AI-GAGDQGMMFGYACNETDVLMPMPIYLAHKLTRRLAEARKDG--T-IDWLRPDGKSQVTVEY--A-Y--G--K-PKRIDTV-VISTQH-APDVS---------HD----EIEK-QVIEKIIRPI-----I-P-A-------N-M-LD--ENT--IFHINPTGRFVTGGPKGDSGLTGRKIIVDTYGGMGRHGGGCFSGKDATKVDRSATYAARWAAKNVVAAGLADRCEIQVAYAIGVAHPVSINVETFGTGK--IPGD----KIADLIYDTFDLRPGAILRDLDMRK-P-------QFRKFSNYGHFGR-D----------------------DLD---L--A-WERT-DR-A-----DALR----K----AAGL----------------

>Chloroflexi_Thermorudis_peleae

---------M-RA------------PR-T-FFTSESVTEGHPDKICDQVSDAVLDAVLAQDP-----AGRVACEVATTTGLLFIFGEITTSA-RI--------DYEKVARQVVTEIGYTR--SD-YGLDGRTMAVITSIKEQAPEISAGV-SR-AYEVR-E--R-DTG----DPFD-AI-GAGDQGMMIGFACTETPELMPLPIALAHRLTRRLAEVRKTG--Q-LPYLRPDGKSQVTVEY--R---AG--K-PVRVDTV-VLSAQH-DPDVD---------PA----RMRA-ELIEAVIAEA-----I-P-T-------E-L-MD--SQT--RCFINPSGSFSVGGPMADAGLTGRKIMVDTYGGMARHGGGAFSGKDPTKVDRSAAYAARYVAKNIVAAGLADRFEIQLSYAIGVARPLAIHIETFGTAR--VDES----VIRALIERHFDLRPAAIIHHLGLNR-P-------IYRQVAAYGHFGR-P----------------------DLD---L--P-WERL-DK-V-----EVLR----E----EAKLSSVVS-SR--------

>Chloroflexi_Dehalogenimonas_formicexedens

--MLSC---Q-DS------------DK-Y-MFTSESVTEGHPDKICDQISDAVLDAILAHDP-----YGRVACETAVTNGLVFVLGEITTKT-YV--------EIPEVVRRTIKEIGYTK--PE-YGFDAQSCGVLVSINKQSPDIAFGV-GQ-SMEAK-A--G-ST-----DPMD-AV-GAGDQGMMVGYACNDTPELMPLPIALSHRLCRQLATVRKQG--V-LPYLRPDGKSQVTVEY--H---LG--K-PKRIETV-VIAAQH-D-DVP---------QS----KIHD-DIVREVIKPI-----I-P-A-------K-L-LD--DNT--NFFVNTTGRFIIGGPASDTGFTGRKILVDTYGGIARHGGGAFSGKDPTKVDRSAAYMARYVAKNIVGAGIADQVEMQVSYTIGRAQPLSVSLETYGTCR--ISQD----ELMAVVNKTFDLRPEAIIENLELRR-P-------IYRQTAAYGHFGR-T----------------------DID---L--P-WERL-NK-V-----EALK----------AALAANAK-K---------

>Chloroflexi_Dehalogenimonas_alkenigignens

---------M-DS------------DK-Y-MFTSESVTEGHPDKICDQVSDAVLDAILTHDP-----YGRVACETAVTNGLVFVLGEITTKT-YV--------EIPDIVRRTVREIGYTK--PE-YGFDYQSCGVMVSINKQSPDIAFGV-GK-SMEAK-A--G-ST-----DPMD-AV-GAGDQGMMVGYACTDTPELMPLPIALSHRLTRQLANVRKQG--V-LPYLRPDGKSQVTVEY--H---LG--K-PKRIETV-VIAAQH-D-DVP---------QA----KIYE-DVVREVIKPT-----I-P-A-------E-L-LD--DKT--NFYVNTTGRFIIGGPASDTGFTGRKILVDTYGGIARHGGGAFSGKDPTKVDRSAAYMARYVTKNIVAAGIADQVEMQVSYTIGRAQPLSVSLETYGTCR--VSQE----QLMQFVHDHFDLRPEAIIENLGLRR-P-------IYRQTAAYGHFGR-T----------------------DID---L--P-WERL-DK-V-----AALK----------AALAVCAQ-A---------

>Chrysiogenetes_Desulfurispirillum_indicum

---------M-SI------------K----LFTSESVTEGHPDKISDQISDAILDGILSQDS-----HGRVACETMVSTGLVIVAGEITTSC-YV--------DVPEVVRQTIREIGYTR--AK-YGFDYETCGVISAINKQSPDIAMGV-DV-G-------------------------GAGDQGMMFGFACRETEELMPLPITLAHKLTMRLAAVRKNG--T-LSYLRPDGKAQVTIAY--E-G--N--R-PVYVDTV-VVSTQH-SESVT---------QD----IIHH-DIIEQVIKPI-----I-P-A-------E-L-FDE-SRI--TYHINPTGKFVIGGPMGDCGLTGRKIIVDTYGGMGRHGGGAFSGKDPSKVDRSAAYAARWVAKNVVAAGYADRCEVQLAYAIGVVKPVSINVNTYGTAT--VDED----RIARAIQEVFDLTPKGIITSLDLLR-P-------IYRPTAAYGHFGR-G------------------------E---F--S-WEQT-NR-V-----DMLR----T----QLG-----------------

>Chrysiogenetes_Chrysiogenes_arsenatis

---------M-TR------------L--T-TFTSESVSEGHPDKMADQISDAILDAILAEDP-----KARVACETLVKTGLVMLAGEITTSA-WI--------DAEAIVRDVVTKIGYDN--SN-LGFDGHTCSVLTAIGKQSPDIAMGV-NE-AEN---H----------------EQ-GAGDQGLMFGYATNETDVLMPAPITLAHRLVKRQSDMRKKK--I-LPWLRPDAKSQVTLRY--E-N--D--R-PIAIDAI-VLSTQH-DPHIA---------TQ----DLWE-AVMEEIIKPV-----L-P-T-------E-W-LH--KGT--KYHINPTGNFVIGGPVGDCGLTGRKIIVDTYGGYARHGGGAFSGKDASKVDRSAAYAGRYVAKNIVAAGLAEKCEIQISYAIGVAEPTNIAVNTFGTGT--ISDD----KIEALIARHFDLRPKGLINMLGLLK-P-------IYQQTAAYGHFGR-N----------------------DID---L--P-WEKT-DK-A-----EMLK----N----DAK-----------------

>Cyanobacteria_Phormidium_tenue

---------M-SE------------Y--S-IFTSESVSEGHPDKMADQISDAVLDAILKEDL-----HARVAVETLVKTGMAVIAGEVRTNT-YV--------DLEDIVRNVILAIGYDS--SD-VGFDGASCAVLNAIGKQSSDIAMGV-DV-ATD---K----------------DL-GAGDQGLMFGYATNETDTLMPAPIYYAHRLVERQAHLRKHG--V-LPWLRPDAKSQVTLRY--E-N--L--K-PVAVEAV-VLSTQH-DPDIS---------QA----DIRE-AVMEEIIKPV-----L-P-N-------G-W-LH--ADT--QYHINPTGQFIIGGPVGDCGLTGRKIIVDTYGGMARHGGGAFSGKDPTKVDRSAAYAGRYVAKNIVAAGLADRCEIQVSYAIGVAQPTSISINTFGTGK--IADG----AIAELVRKHFDLRPQGLIDMLDLRR-P-------IYQQTAAYGHFGR-E----------------------LPD---F--T-WEKT-DK-A-----DLLK----T----AL------------------

>Cyanobacteria_Nodosilinea_nodulosa

---------M-SE------------Y--S-IFTSESVSEGHPDKMADQISDAVLDAILKEDL-----HARVAVETLVKTGMAVIAGEVRTNT-YV--------DLEDIVRNVILGIGYDS--SD-VGFDGASCAVLNAIGKQSSDIAIGV-DV-AVD---K----------------DL-GAGDQGLMFGYATSETETLMPAPIYYAHRLVERQAHLRKHG--V-LPWLRPDAKSQVTLRY--E-H--L--K-PVAVEAV-VLSTQH-DPDIS---------QA----DIRE-AVMEEIIKPV-----L-P-S-------G-W-LH--ANT--QYHINPTGQFIIGGPVGDCGLTGRKIIVDTYGGMARHGGGAFSGKDPTKVDRSAAYAGRYVAKNIVAAGLADRCEIQVSYAIGVAKPTSISINTFGTGK--IADG----AIADLVSKHFDLRPQGLIDMLDLRR-P-------IYQQTAAYGHFGR-E----------------------LPD---F--T-WENT-DK-A-----DLLK----G----AL------------------

>Cyanobacteria_Nostoc_phaeroides

-----------MS------------RR-Y-LFTSESVTEGHPDKICDQISDTILDALLTQDP-----SSRVAAEVVVNTGLVLITGEITTKA-NV--------NFVNLARKKIAEIGYTN--AD-NGFSANSTSVLLALDEQSPDIAQGV-NT-AQETR-E----QDS-D--EQFD-KI-GAGDQGIMFGFASNETPELMPLPISLAHRIARRLAAVRKTG--E-LPYLRPDGKTQVTVIY--E-D--G--R-PVGIDTI-LISTQH-TASIGEITDEAAV-QA----KIKQ-DLWLAVVEPVFGDIDVKP-N---------------EET--RFLVNPTGKFVVGGPQGDSGLTGRKIIVDTYGGYSRHGGGAFSGKDPTKVDRSAAYAARYVAKNIVAAGLAEKVEIQLSYAIGVARPTSILVDTFGTGK--VDEE----TLLELINKHFELRPAGIIHTFNLRNLPSERGGR-FYQDVAAYGHFGR-A----------------------DLD---L--P-WEQT-DK-A-----ELLK----Q----AANESLSAVVAQALT-----

>Cyanobacteria_Trichormus_variabilis

-----------MS------------RR-Y-LFTSESVTEGHPDKICDQISDTILDTLLTQDP-----TSRVAAEVVVNTGLVLITGEITTKA-NV--------NYANIARQKIAEIGYTN--AD-NGFSASSTSVIVALDEQSPDIAQGV-NT-AQETR-E----QDS-E--ELFD-KI-GAGDQGIMFGFACNETPELMPLPICLAHRIARRLAAVRKTG--E-LSYLRPDGKTQVTVVY--E-D--G--R-PVGIDTV-LISTQH-TATIGDITDEAAV-QA----KIKQ-DLWTAVVEPVFGDLEIKP-D---------------QET--RFLVNPTGKFVIGGPQGDSGLTGRKIIVDTYGGYSRHGGGAFSGKDPTKVDRSAAYAARYVAKNIVAAGLAEKCEVQLSYAIGVARPVSIFLDTFGTGK--VDDE----ILLGLVKDNFELRPAGIIHSFNLRNLPSERGGR-FYQDVAAYGHLGR-N----------------------DLD---L--P-WERT-DK-A-----DFLK----Q----AVTHSLSAAIA---------

>Cyanobacteria_Nodularia_spumigena

-----------MS------------KR-Y-LFTSESVTEGHPDKICDQISDTILDTLLTEDP-----SSRVAAEVVVNTGLVLITGEITSKT-HA--------NYVNIARQKIAEIGYID--AV-NGFSAGSASVLVALDEQSPDIAQGV-NK-AQETR-Q----QNS-E--ELFD-AV-GAGDQGIMFGFACNETPELMPLPISLAHRLARRLAAVRKTG--K-LPYLRPDGKTQVTVAY--E-D--G--Y-PVGIDTI-LISTQH-TESIGEITEEAAI-QA----KIKE-DLWSTVVEPAFGDIDIKP-S---------------QET--RFLVNPTGKFVIGGPQGDSGLTGRKIIVDTYGGYSRHGGGAFSGKDPTKVDRSAAYAARYMAKNIVAAGLAQKCEVQLSYAIGVARPVSIFLETFGTGK--LDDE----TLLELVKDQFELRPAGIIHAFNLRNLPSERGGR-FYQDIAAYGHFGR-N----------------------DLD---L--P-WERT-DK-V-----ELLQ----Q----LVTQSLAAAIA---------

>Cyanobacteria_Tychonema_bourrellyi

-----------MS------------RH-Y-LFTSESVTEGHPDKICDQISDTILDALLTEDP-----KSRVAAEVVVNTGLMLLTGEITTKA-QV--------NYIELARKKIADIGYIH--AD-NGFSANSCSVLVALDTQSPDIAQGV-NT-AHESR-E----QSS-E--EELD-AI-GAGDQGLMFGFACNETPELMPLPISLAHRICRQLAAVRKTG--Q-LPYLRPDGKSQVTVVY--E-D--G--K-PVGIDTI-LISTQH-TEAIGDIVDTAAV-QA----KIKE-DLWTAVVEPVFADIAIKP-D---------------AET--RFLVNPTGKFVIGGPQGDSGLTGRKIIVDTYGGYSRHGGGAFSGKDPTKVDRSAAYACRYVAKNIVAAGLAEKCEVQLSYAIGVARPVSMMIETFGTGK--VDDD----RLLEVVKQLFELRPAGIIQAFNLQNLPAERGGR-FYQDVAAYGHFGR-T----------------------DLE---L--P-WEQT-DK-V-----ELLK----S----ALSQQLSAVAG---------

>Cyanobacteria_Trichodesmium_erythraeum

-----------MS------------SR-Y-LFTSESVTEGHPDKICDQISDTIIDAILAQDP-----QSRVAAEVVVNTGLVLITGEITTKA-QV--------NYIELARKKIADIGYVH--AE-NGFSADSCSVLVALDEQSADIAQGV-DK-AQETR-E----LLS-E--EELD-AV-GAGDQGLMFGFACNETPELMPLPISLAHRVCRQLTAVRKTG--Q-LPYLRPDGKSQVTITY--E-D--G--R-PVGIDTI-LISTQH-TAKIGEITELSDI-QA----KIKE-DLWKYVVEPIFKDAEIKP-N---------------SQT--RFLVNPTGKFVIGGPQGDCGLTGRKIIIDTYGGYSRHGGGAFSGKDPTKVDRSAAYACRYIAKNIVAADLAEKCEVQISYAIGVAKPVSMMIETFGTSK--VDED----KLLEVVKENFELRPAGIIQNFNLRNLPGERGGR-FYQDVAAYGHLGR-T----------------------DLD---L--P-WEQT-DK-V-----ELLK----Q----AFSPQLLATAS---------

>Cyanobacteria_Acaryochloris_marina

-----------MP------------KR-Y-LFTSESVTEGHPDKVCDQISDTILDALLSQDP-----ASRVAAEVVVNTGLVLLTGEITTKA-QV--------NFVDLVRQKITEIGYTD--SD-NGFAANSCSVLVALDEQSPDIAQGV-DS-AQETR-E----EKS-D--QELD-AI-GAGDQGLMFGFACNETPELMPLPISLAHRVTRRLAAVRKT---Q-LGYLRPDGKSQVTVTY--E-D--G--R-PVGIDTI-LVSTQH-DATIGDITDPAAV-QA----KIKE-DLWNAVVLPVFADIDIKP-D---------------DNT--RFLVNPTGQFVVGGPQGDAGLTGRKIIVDTYGGYSRHGGGAFSGKDPTKVDRSAAYACRYVAKNIVAAGLAEKCEVQLSYAIGVARPVSILIETFGTGK--VDED----RLLQVVQENFELRPAGLIQTFGLTKLPGERGGR-FYQDVAAYGHFGR-T----------------------DLD---L--P-WEAT-DK-A-----DLLK----Q----ALSPALSGNA----------

>Cyanobacteria_Acaryochloris_sp

-----------MS------------KR-Y-LFTSESVTEGHPDKVCDQISDTILDALLAQDP-----MSRVAAETVVNTGLVLVTGEVTTKA-DV--------NIVKLVREKITEIGYTD--SD-NGFAANSCSVLIALDEQSPDIAQGV-DA-AQETR-E----QKS-D--EELD-SI-GAGDQGLMFGYACNETPELMPLPISLAHRITRQLAAVRKQG--K-LPYLRPDGKSQVSVAY--E-D--G--Q-PVGIDTI-LISTQH-DATIEEIREPSAV-RD----RIQA-DLWEHVVLPIFADLDLKP-D---------------DDT--RYLVNPTGQFVIGGPQGDAGLTGRKIIVDTYGGYSRHGGGAFSGKDPTKVDRSAAYASRYVAKNIVAAGLADKCEVQVSYAIGVARPTSILIETFGTGK--VDEE----RLLEVVQTHFDLRPAGLIQAFDLTHLPQERGGR-FYQDVAAYGHFGR-A----------------------DLD---L--P-WEKT-DK-A-----DALK----Q----ALSPAMAGAAG---------

>Cyanobacteria_Calothrix_sp

-----------MS------------RR-N-LFTSESVTEGHPDKVCDQISDTILDALLTQDP-----KSRVAAEVVVNTGMVVITGEITTQA-NV--------NYVKLVRKKITEIGYTD--AD-NGFSANSCSVLIALDEQSPDIAQGV-NS-AQETR-G----ASS-D--EQFD-KI-GAGDQGIMFGFACNETPELMPLPISLAHRLARRLSVVRKKG--L-MPYLRPDGKTQVTVVY--E-D--G--R-PVGIDTI-LISTQH-DATIGEITDEDAV-QA----KIKS-DLWTAVVEPIFGDLTIKP-D---------------AQT--RFLVNPTGKFVVGGPQGDAGLTGRKIIVDTYGGYSRHGGGAFSGKDPTKVDRSAAYACRYVAKNIVAAGLAEKCEVQLSYAIGVARPTSIFVDTFGTGT--VDDE----VILKLIQDNFELRPAGIIQAFDLQNLPKERGGR-FYQDVAAYGHFGR-D----------------------DLD---L--P-WERT-DK-A-----ELLR----A----AASELRTPAIA---------

>Cyanobacteria_Merismopedia_glauca

-----------MS------------RR-Y-LFTSESVTEGHPDKICDQLSDTILDALLTEDP-----HSRVAAEVVVNTGLVVITGEITSKA-HV--------NFARLVRQKIADIGYTN--AD-NGFCATSCNVMVALDEQSPDIAQGV-NQ-AHETR-E----DDS-E--DALD-AV-GAGDQGIMFGFACNETPELMPLPISLAHRISRRLAAVRKTG--D-LPYLRPDGKTQVTVAY--E-D--G--R-PVGIDTI-LVSTQH-TASIDGITEEAAV-QA----RIKA-DLWEKVVQPIFGDDEIKP-D---------------SET--RFLVNPTGKFVVGGPQGDAGLTGRKIIVDTYGGYSRHGGGAFSGKDPTKVDRSAAYAARYVAKNIVAAGLADKCEIQLSYAIGVAKPVSFLVETFGTGK--IEEE----RLLELVQKHFELRPAGIIQTFNLRNLPAERNGR-FYQDVAAYGHFGR-T----------------------DIE---L--P-WEQT-DK-A-----AILK----D----AAKELQTIS-----------

>Cyanobacteria_Hydrococcus_rivularis

-----------MS------------RR-Y-LFTSESVTEGHPDKICDQISDTILDALLAQDD-----HSRVAAEVVVNTGLVLITGEITTNA-HV--------NYVDLARKKIAEIGYTD--AN-NGFSANSCAVLVALDEQSPDIAQGV-TA-AHEQR-E----ELS-D--DELD-RI-GAGDQGLMFGYACNETPELMPMPISLAHRISRRLAAVRKTG--D-LSYLRPDGKTQVSVVY--E-D--G--R-PIGIDTI-LISTQH-TETIGEICDNAAV-QA----KIKA-DLWEAVVEPVFADIDVKP-D---------------RDT--RYLVNPTGKFVVGGPQGDAGLTGRKIIVDTYGGYSRHGGGAFSGKDPTKVDRSAAYACRYVAKNIVAAGLADKCEVQVSYAIGVAKPVSIMVETFGTAK--VDEE----KLLEAVKQHFELRPAGIIQTFNLRRLPAER-GR-FYQDVAAYGHFGR-T----------------------DLE---L--P-WERT-DK-A-----ALLK----E----ALSYSLSNATVAI-------

>Cyanobacteria_Stanieria_cyanosphaera

-----------MS------------RR-Y-LFTSESVTEGHPDKICDQISDTIVDALLSEDH-----HSRIAAEVVVNTGLVLITGEISTQA-HV--------NYVDLARQKIAEIGYVN--AE-NGFSANSCSVLVALDEQSPDIAQGV-TA-AQEQR-E----ELS-E--DELD-QI-GAGDQGIMFGFACNETPELMPMPISLAHRMSRRLAAVRKTG--E-LAYLRPDGKTQVSVIY--E-D--G--Q-PIGIDTI-LISTQH-TETIGEITEGSAV-QN----KIKT-DLWEAVVQPVFGDLSVKP-D---------------QNT--RFLVNPTGKFVIGGPQGDSGLTGRKIIVDTYGGYSRHGGGAFSGKDPTKVDRSASYACRYVAKNIVAAGLADKCEVQVSYAIGVAKPVSIFIETFGTGK--VSEE----KLLTLVKEYFEFRPAGIIQTLNMRRLPSERGGR-FYQDVAAYGHFGR-N----------------------DLD---L--P-WEKT-DK-A-----TLLK----EQ---SLSGTLA-------------

>Cyanobacteria_Pleurocapsa_minor

-----------MS------------RR-Y-LFTSESVTEGHPDKICDQISDTILDALLASDD-----RSRVAAEVVVNTGLVLITGEITTKA-QI--------NYVDLARRKIAEIGYTN--AE-NGFAANSCSVLVALDEQSPDIAQGV-TA-AQEQR-E----ELS-D--DELD-RI-GAGDQGIVFGYACRETPELMPMPISLAHRISRRLAAVRKTG--D-LPYLRPDGKTQVSVIY--E-D--G--Q-PIGIDTI-LISTQH-AETIGEIADNATVDQA----KIKS-DLWDAVVKPVFADIEIKP-T---------------QET--RFLVNPTGKFVIGGPQGDAGLTGRKIIVDTYGGYSRHGGGAFSGKDPTKVDRSAAYACRYVAKNIVAAGLADKCEVQASYAIGVARPVSIMVETFGTGK--VDEE----KLLAFVREYFELRPAGIIQNFNLRRLPAEQGGR-FYQDVAAYGHFGR------------------------DLD--LL--P-WERT-DK-V-----VLLQ----D----AFAPSLSEVTVGNSRGRF--

>Cyanobacteria_Crocosphaera_watsonii

-----------MS------------KR-Y-LFTSESVTEGHPDKVCDQISDTIIDTLLNEDD-----QSRVAAEVVVNTGLVLITGEVTSKA-HV--------DFAKLARNKIAEIGYTD--AD-NGFSANSCSVLVALDEQSPDIAQGV-SQ-AREQR-K----ELS-D--DELD-QI-GAGDQGLMFGYACNETPELMPLPISLAHRCARRLAAVRKTG--D-LSYLRPDGKTQVSIAY--E-D--G--I-PVGIDTI-LISTQH-DETIGSITDNDAV-QA----KIKA-DLWEAVILPVFADIDIKP-S---------------QDT--RYLVNPTGKFVIGGPQGDAGLTGRKIIVDTYGGYSRHGGGAFSGKDPTKVDRSAAYASRYVAKNIVAAGLADKCEVQLSYAIGVARPVSILVETFGTGK--VDET----KLLEVVNELFELRPAGIIQSLKLRELPSQRGGR-FYQDVAAYGHFGR-N----------------------DLD---L--P-WEAT-DK-A-----ALLK----D----AL------LKVGV-------

>Cyanobacteria_Gloeocapsa_sp

-----------MS------------NR-Y-LFSSESVTEGHPDKICDQISDTIVDALLSEDN-----HSRIAAEVVVNTGLVLITGEITSKA-HV--------NFVELARKKIAEIGYTD--AK-NGFCANSCSILLAIDEQSPDISQGV-TQ-AKEQR-D----KLS-E--DELD-KI-GAGDQGLMFGYACNETPELMPLPISLAHRISRQLAIARKTG--E-LPYLRPDGKTQVTIIY--E-D--G--K-PVGIDTI-LISTQH-TETIGEITAGEAV-QA----KIKA-DLWEVVVGPVFADIELKP-D---------------DQT--RFLVNPTGKFVIGGPQGDSGLTGRKIIVDTYGGYSRHGGGAFSGKDPTKVDRSASYACRYVAKNIVAAGLAEKCEVQVSYAIGVARPVSIMIETFGTSK--IDSE----KLLEVVQQNFDLRPAGIIQTFNLRDLPKQRNGR-FYQDVAAYGHFGR-N----------------------DLD---L--P-WEKT-DK-A-----AALR----E----AVFTSVSPVTV---------

>Cyanobacteria_Microcystis_aeruginosa

-----------MS------------KR-Y-LFTSESVTEGHPDKICDQISDTILDALLSQDD-----HSRVAAEVVVNTGLVLITGEITSKA-QV--------HFVDLVRKKIAEIGYIN--AD-NGFSSNSCTVLVALDEQSPDISQGV-TA-AQENR-E----LLS-N--DELD-KI-GAGDQGIVFGFACNETPEFMPLPISLAHRIARRLAAVRKTG--E-LSYLRPDGKTQVSVIY--E-N--G--R-PVGIDTI-LVSTQH-DATIGDITDNDLV-QQ----KIKS-DLWKAVVEPVFSDLEIKP-D---------------ANT--RYLVNPTGKFVIGGPQGDAGLTGRKIIVDTYGGYSRHGGGAFSGKDPTKVDRSAAYACRYVAKNIVAAGLADKCEIQVGYAIGVARPVSLFVETFGTGK--VADD----VILDLINKYFELRPAGIIQTFNLRGLPSERGERLFYQDVAAYGHFGR-N----------------------DLD---L--P-WEKT-DK-A-----AILK----E----ALTSVVV-------------

>Cyanobacteria_Atelocyanobacterium_thalassa

-----------MS------------RS-Y-LFTSESVTEGHPDKVCDQISDTIIDTLLHHDD-----QSRVAAEVVVNTGLVLITGEITSQA-NV--------NFVDLARKKIAEIGYTD--AD-NGFSADSCSVLIALDQQSADIAQGV-TQ-AHEQR-K----SSS-D--DDLD-KI-GAGDQGIMFGFACDETPELMPLPISLAHRFSHRLATVRKTG--D-LSYLRPDGKTQVSIVY--E-D--G--I-PVGIDTI-LISTQH-DETIGSLTSNNDVDQN----KIKS-DLWDAVILPVLKDFNIES-S---------------ENT--RFLVNPTGKFVIGGPQGDAGLTGRKIIVDTYGGYSRHGGGAFSGKDPTKVDRSAAYACRYVAKNIVAAGLAKKCEVQVSYAIGVARPVSIFIDTFGTSK--VDEI----KLLEIIKELFELRPAGIIQNFRLRQLPAERNGR-FYQDTAVYGHFGR-D----------------------DLD---L--P-WEST-DK-A-----MVLN----Q----AFSINV--------------

>Cyanobacteria_Halomicronema_hongdechloris

-----------MS------------RR-Y-LFTSESVTEGHPDKICDQISDTILDALLTEDP-----SSRVATEVVVNTGLVLITGEITSNT-QA--------DYAHIARQKIAEIGYTN--AD-NGFSADSCSVLIALDKQSSDIALGV-DN-AHEQR-E----QLS-D--EALD-AI-GAGDQGLMFGFACNETPELMPLPISLAHRISRRLAAVRKAG--E-LSYLRPDGKTQVSVVY--E-D--G--R-PVGIDTI-LISTQH-DPIIDDMQDATAI-QT----KIKE-DLWTYVVQPAFANIEIKP-D---------------DAT--RFLVNPTGKFVIGGPQGDSGLTGRKIIVDTYGGYSRHGGGAFSGKDPTKVDRSAAYACRYAAKNIVAAGLADKCEVQLSYAIGVARPVSVLVETFGTGR--IDED----ILTDLVRQQFELRPAGIIQSFNLKNLPAERGGR-FYQDIASYGHFGR-N----------------------DLE---L--P-WEAT-DK-A-----DALR----D----AAKRYLVSPAGV--------

>Cyanobacteria_Rubidibacter_lacunae

-----------MS------------RR-Y-LFSSESVTEGHPDKICDQISDTILDALLAGDP-----SSRVAAEVVVNTGLVLVTGEITSQA-AI--------HYANLVREKIAEIGYTD--AD-NGFSADSCSVMIAIDEQSPDISQGV-SA-ARESR-L----SKS-D--DELD-RI-GAGDQGLMFGFACNETPELMPLPISLAHRITRRLALVRKSG--E-LGYLRPDGKSQVTVVY--E-D--G--K-PVGIDTI-LISTQH-DAEVGGTTDEAAV-HA----QIKR-DLWPTVIEPVFSDLAIKP-G---------------DTT--RYLVNPTGKFVVGGPQGDSGLTGRKIIVDTYGGYSRHGGGAFSGKDPTKVDRSAAYAARYAAKNIVAAGLADKCEVQLSYAIGVARPVSILVETFGTGK--VDEE----RLLELVRDHFELRPAGIIQAFNLRGLPGERGGR-FFQDTAAYGHFGR-P----------------------DLD---L--P-WERT-DK-A-----ALLK----E----SLAVNA--------------

>Cyanobacteria_Dactylococcopsis_salina

-----------MS------------SK-Y-IFSSESVTEGHPDKVCDQISDTILDALLAEDD-----KSRVAAEVVVNTGLVLVTGEITSKT-QV--------NYINLVRDKIKEIGYTD--AD-NGFSADSCAVLVALDEQSPDISQGV-TS-AQESR------DLS-D--DELD-QI-GAGDQGLMFGFACNETPELMPLPISLSHRLTRQLAKVRKDG--T-LNYLRPDGKSQVSVAY--E-N--G--K-PVRIDTI-LLSTQH-DAEVGGSTEEKAV-HD----KIKA-DLWEKVVLPIFEDIDIKP-D---------------ADT--KYLMNPTGKFVVGGPQGDAGLTGRKIIVDTYGGYSRHGGGAFSGKDPTKVDRSAAYATRYVAKNIVAAGLAEKCEVQLSYAIGVARPTSVFVDTFGTGK--VDED----KLLEVIRENFELRPAGIIQAFDLQSLPKQRGGR-FYQDTAAYGHFGR-P----------------------DLD---L--P-WERT-DK-V-----ETLK----E----ALKAAAAV------------

>Cyanobacteria_Xenococcus_sp

-----------MS------------SR-Y-LFTSESVTEGHPDKICDQISDTILDALLAQDP-----LSRVAAEVVVNTGLVLVTGEITSQA-EV--------NYVKLVRNKIAEIGYTH--AE-NGFSADSCSVLIALDEQSPDISQGV-SS-AQEQR-S----ELS-D--DELD-KI-GAGDQGLMFGYACNETPELMPMPISLAHRISRSLTAARKVG--G-LSYLRPDGKTQVSVVY--E-D--G--K-PVGIDTI-LISTQH-TATIGDLTENDDVDQQ----KIKE-DLWVKVVKPVFEDIDLQP-D---------------DNT--RFLVNPTGKFVVGGPQGDSGLTGRKIIVDTYGGYSRHGGGAFSGKDPTKVDRSACYACRYVAKNIVAAGLADKCEVQVSYAIGVAKPVSVFLETFGTAK--IAED----KLLEIVLKHFELRPAGILQTLKLRELPKERGGR-FYQEVAAYGHFGR-T----------------------DLD---L--P-WEQL-DK-V-----EALK----EY---RLVEATV-------------

>Cyanobacteria_Limnothrix_sp

-----------MS------------RR-S-LFTSESVTEGHPDKICDQISDAILDALLTVDP-----KARVAAEVVVNTGLVLITGEISSKA-HV--------NFVEIARRKIAEIGYTD--SS-NGFAAESCSVIVALDEQSPDIAQGV-NA-AQETR-E----AMS-D--EALD-AI-GAGDQGLMFGYACNETPELMPLPISIAHRLARRLAAVRKTG--Q-LAYLRPDGKTQVSVVY--E-D--G--R-PVAIDTI-LISTQH-TASIGDISEEAAVEQA----KIRE-DLWSAVVEPVFTDLSVKP-S---------------SET--RFLVNPTGKFVIGGPQGDSGLTGRKIIVDTYGGYARHGGGAFSGKDPTKVDRSAAYACRHVAKNVVAAGLADKCEVQVSYAIGVARPVSISIETFGTAK--VDED----QLLTAIRNCFELRPAGIIQTYDLQRLPGDRGGR-FYQEVAAYGHLGR-S----------------------DLD---L--P-WEAL-DK-V-----EALK----A----NLAQLSSVS-----------

>Cyanobacteria_Thermosynechococcus_elongatus

-------------------------MR-Y-LFSSESVTEGHPDKICDQIADAILDALLTQDP-----QSRVAAEVVVNTGLVLITGEITTKA-QV--------NYVNLARQKIHEIGYTD--AN-NGFAANSCAVLVALDEQSPDIARGV-DT-AQEAR-E----QLS-D--AELD-RI-GAGDQGIMFGYACNETPEYMPLPISLAHRMARRLAAVRKTG--Q-LPYLRPDGKTQVTVIY--E-D--G--K-PVGIDTI-LISTQH-TATIDDISEESAV-QA----KIKA-DLWEAVVKPVFADLTLQP-D---------------GNT--RFLVNPTGKFVIGGPQGDSGLTGRKLVVDTYGGYARHGGGAFSGKDPTKVDRSAAYMARYIAKNIVAAGLADKCELQISYAIGVARPMSLFVDTFGTGK--LSPE----QLLELIKTHFDLRPAAIIQTLNLRHLPQERGGR-FYQDVAAYGHFGR-H----------------------DLD---L--P-WEKL-DK-V-----ADLQ----A----AAAQFLSAV-----------

>Cyanobacteria_Prochlorothrix_hollandica

-----------MA------------RR-Y-LFTSESVTEGHPDKICDQISDTILDTLLAADP-----ASRVAAEVVVNTGLVLITGEITSKA-QV--------NYIDLARQKIAEIGYLG--ED-SGFSAHSCSVLVALDAQSPDIAQGV-DS-AQEQR-E----ALS-D--EELD-KI-GAGDQGLMFGFACNETPELMPMPISLAHRLSLRMATVRKDG--T-LPYLRPDGKSQVTVAY--E-D--G--K-PVSIDTI-LLSTQH-APTIGDITGDGPV-RD----KIQA-DLWEQVVLLVFADIAVKP-S---------------DAT--KFLVNPTGKFVVGGPQGDAGLTGRKIIVDTYGGYSRHGGGAFSGKDPTKVDRSAAYACRHVAKNIVAAGLADKCEVQLSYAIGVARPVSFMVETFGTGK--VDED----QLTDAVSKLFDLRPAGIIQAFNLRGLPGERGGR-FYQDVAAYGHFGR-N----------------------DLD---L--P-WEKL-DK-V-----DALK----A----ALA-----------------

>Cyanobacteriua_Synechococcus_elongatus

-----------MT------------RR-Y-LFTSESVTEGHPDKICDQISDTILDALLTEDP-----SSRVAAEVVVNTGLVLITGEVSTQA-QT--------NLIDLARRKIAEIGYTG--ED-SGFGANNCTVLIALDKQSPDIAQGV-DT-AQEQR-Q----ASS-D--ERFD-SI-GAGDQGIMFGYACNEAPELMPLPISLSHRLARQLAVVRHNG--Q-LDYLRPDGKTQVTIAY--E-D--G--K-PVAIDTI-LISTQH-KAAIGDISDDNAV-QE----RIKS-DLWEQVVLPVFSDLVIQP-D---------------SAT--RFLVNPTGKFVIGGPQGDAGLTGRKIIVDTYGGYSRHGGGAFSGKDPTKVDRSAAYACRYVAKNIVAAGLAEKCEVQLSYAIGVARPVSVLVETFGTGK--VADE----VLLDLVRKHFELRPAGIIEHFNLQRLPGERGGR-FYQEVAAYGHFGR-N----------------------DLD---L--P-WEQT-DK-A-----DTLR----QE---ALATTQA-------------

>Cyanobacteria_Roseofilum_reptotaenium

-----------MV------------RR-Y-LFTSESVTEGHPDKICDQISDTIIDALLAEDP-----ASRVAAEVAVNTGLVLITGEITSQA-QV--------NYVDLARKKIAEIGYVH--SE-GGFTSNSCSVLVALDAQSPDIAQGV-DA-AAEQR-E----MSS-E--ERFD-ST-GAGDQGLMFGFACNETPELMPLPISLAHRISRRLAAVRKTG--Q-LSYLRPDGKTQVTVAY--E-D--G--K-PVAIDTV-LISTQH-AATID---------QK----KIKA-DLWDAVVLPSFSDLEIKP-D---------------SAT--KYFVNPTGKFVVGGPQGDAGLTGRKIIVDTYGGYSRHGGGAFSGKDPTKVDRSAAYACRYAAKNIVAAGLADKCEVQLGYAIGVARPVSIMIETFGTGK--VNDD----QLLEAAQKVFEFRPAGIIETFGLRT-PQERGGRLFYQDVAAYGHFGR-N----------------------DLD---L--P-WEKT-DK-V-----ELLK----E----AVSAATTAG-----------

>Cyanobacteria_Chamaesiphon_minutus

-----------MS------------RR-Y-LFTSESVTEGHPDKICDQISDTILDALLAQDP-----YSRVAAEVVVNTGLVLITGEITTKA-TV--------NYIDLARNKIAEIGYTD--AD-NGFSATSASVLIALDQQSPDIAQGV-NT-AKENR-D----D-S-T--DIFD-QT-GAGDQGIMFGYACNETPELMPLPISLSHRVARRLAAVRKTG--D-LPYLRPDGKTQITVEY--K-N--G--I-PIAIDTI-LISTQH-DATIGDIKDDIGV-QA----KIKA-DLWNYVVLPVFADLDIKP-D---------------DKT--HFLVNPTGKFVIGGPQGDAGLTGRKIIVDTYGGYSRHGGGAFSGKDPTKVDRSAAYACRHVAKNIVAAGLATKCEVQLSYAIGVAAPVSILVETFGTGI--ISDD----QLFELIEEHFELRPEGILATFNLRNLPAERNGR-FYQDVAAYGHFGR-A----------------------DLD---L--P-WEQT-EK-A-----ATLK----A----AATKFETTTVG---------

>Cyanobacteria_Oscillatoriales_cyanobacterium

-----------MA------------SR-F-LFTSESVTEGHPDKICDQISDTILDTLLAQDP-----RSRVAAEVVVNTGLVLITGEITTKA-HV--------NYVDIARKKIAEIGYTN--AD-NGFSANSCAVMVALDEQSPDIAQGV-NV-AHEVR-T--G-AEE-E--AALE-AV-GAGDQGIMFGFACDETPEYMPMPIALAHRLARQLSVVRKNG--T-LPYLRPDGKTQVTVVY--E-G--D--K-PVEIDTI-LISTQH-DAAIDGITDEAKV-QA----RIKA-DLWTHVVLPSFADTSVKP-S---------------EQT--RYLVNPTGKFVIGGPQGDSGLTGRKIIVDTYGGYARHGGGAFSGKDPTKVDRSAAYAARHAAKNIVAAGLASKCELQISYAIGVARPTSMHLDTFGTGK--VDEE----TLLRLVKDNFDLRPAAIIKDFDLQKLPSDRAGR-FYQDVAAYGHMGR-N----------------------DLD---L--P-WEKL-DK-V-----EALK----K----YL------------------

>Cyanobacteria_Pseudanabaena_sp

-----------MV------------SR-N-LFTSESVTEGHPDKICDQISDTIVDTLLANDP-----ASRIAAEVVVNTGLVLITGEITSKA-HV--------NFVDLARRKIAEIGYID--AD-NGFSANSCAVLLAIDEQSPDIAQGV-DQ-ALEAR-Q--G-EQS-D--EELE-AV-GAGDQGIMFGFACDETPELMPMPIALAHRISRQLAAVRKDD--T-LAYLRPDGKTQVTVIY--E-G--G--K-PIGIDTV-LVSTQH-DPEISGTSDTAKM-QE----IIKA-DLWQEVVKPAFEGTFVLP-D---------------PNT--RFLVNPTGKFVIGGPQGDSGLTGRKIIVDTYGGYSRHGGGAFSGKDPTKVDRSAAYACRYVAKNIVAAGLAAKCELQISYAIGVARPTSLTIETFGTSK--VDED----VLLKLVQDNFDLRPAAIIQNFNLRKLPSMRNGR-FYQDVAAYGHLGR-T----------------------DLD---L--P-WEKT-DK-V-----DVLK----Q----AL------------------

>Cyanobacteria_Leptolyngbya_foveolarum

-----------MS------------RR-YLLFTSESVTEGHPDKICDQISDTILDTMLALDP-----DSRVAAEVVVNTGLVLVTGEISSKA-KV--------NFVEVVRKKIAEIGYTN--PK-NGFSADSCSVLIAIDEQSADIAQGV-DQ-AQESR-A----KTS-D--ERWD-AVAGAGDQGLMFGFACNETPELMPLPICLAHRISLELARVRKSG--R-LDYLGPDGKTQVTVEY--E-D--G--K-PVGIDTV-LVSTQH-TETIDGLSSNEDV-QA----RIKK-DLWQAVVLPAFAGLDIQP-G---------------EST--RFLVNPTGKFVIGGPQGDSGLTGRKIIVDTYGGYSRHGGGAFSGKDPTKVDRSAAYAGRYVAKNIVAAGLADKCEVQLSYAIGVARPVSIFVETFGTGS--IPDD----KLLDLVTEHFELRPAGIIETFNLRGLPQERGGR-FYQDIAAYGHFGR-E----------------------DLD---L--P-WEKT-DK-V-----EALK----A----ATEKLAATV-----------

>Cyanobacteria_Cyanosarcina_burmensis

---MEA---C-NL------------SR-Y-LFTSESVTEGHPDKICDQISDAILDAHLSQDP-----TSRVAAEVVVNTGLVLVTGEISSKA-KV--------DYVKLVRQKIAEIGYTD--AN-NGFSANSCSILVALDEQSPDIAQGV-NK-AQEYE-Q----GDS-E--DDWS-AI-GAGDQGIMFGYACKETPELMPLPISLAHRLARQLAKVRKIG--K-LSYLRPDGKTQVTVVY--E-N--G--R-PVGIDTI-LISTQH-SEATT---------LG----EIYQ-GLLLEVVEPVFADLDIQI-D---------------NST--RFLVNPTGKFVIGGPQGDSGLTGRKIIVDTYGGYSRHGGGAFSGKDPTKVDRSASYACRYVAKNIVAAGLADKCEVQLSYAIGMSKPVSILIDTFGTGK--LPEE----DLLNLVNQHFDLRPAGLIHTFNMQGLPQERNGR-FYQDVAAYGHFGR-M----------------------DLD---L--P-WERL-DK-V-----PQLQ----E----AITVQMSA------------

>Cyanobacteria_Gloeobacter_violaceus

------------M------------SR-Y-LFSSESVTEGHPDKICDQISDTILDALLTQDP-----RSRVAAEVVVNTGMVVVTGEITTTA-NV--------NFTKLVRDKIREIGYTE--AD-NGFSADSCAVFLALDEQSPEIAQGV-SC-ALEVR------TSE-E--DALD-RI-GAGDQGLMFGFACTETPELMPLPISVAHRLTRRLAQVRKDG--T-LAYLKPDGKAQVTVEYR-K-D--GVDR-PGRIDTI-LISTQH-AAVIDDLSDNDAV-QA----RIKA-DLQTHVIGPVFADLDIRP-D---------------AQT--RLLVNPSGRFVIGGPQGDSGLTGRKIIVDTYGGYARHGGGAFSGKDPTKVDRSAAYAARYVAKNIVAAELADRCEVQVAYAIGVARPVSIFVETFGTGR--VSDE----ALMLLVREHFDLRPAAILRDFDLCRLPAQRGGR-FYQDVAAYGHLGR-P----------------------DLD---L--P-WEHT-DK-A-----ATLK----Q----AIQTAAAV------------

>Cyanobacteria_Aphanothece_minutissima

------------M------------SR-F-VFTSESVTEGHPDKICDQVSDAVLDALLAQDP-----SSRVACETVVNTGLCLITGEVTTNA-RV--------DFNTLVRGVIEQIGYSG--ARAGGFDARSCAVLIALDQQSPDIAQGV-DE-ADDHD-G-----------DPLD-KV-GAGDQGIMFGYACDETPELMPLPISLAHRLALRLAQVRHDG--S-LGYLLPDGKTQVSVVY--E-N--D--R-PVAIDTI-LISTQH-TAEVEGISDEKAV-QQ----RIKD-DLWTHVVLPATADLPLKP-S-------K-------EST--RFYVNPTGKFVVGGPQGDAGLTGRKIIVDTYGGYARHGGGAFSGKDPTKVDRSAAYAARYVAKALVASGLAHKAEVQLSYAIGVARPVSILVESFGTGA--LSNA----DLTALVQEHFDLRPGAIIEAFGLRELPQQRGGR-FYQDVAAYGHFGR-K----------------------DLD---L--P-WEDV-TAIA-----ATLK----Q----ATASRVSAVSGG--------

>Cyanobacteria_Cyanobium_gracile

------------M------------SR-F-VFTSESVTEGHPDKICDQVSDAVLDALLAQDP-----SSRVACETVVNTGLCLITGEVTTNA-RV--------DFNTLVRGVIEQIGYSG--ARAGGFDARSCAVLIALDQQSPDIAQGV-DE-ADDHD-G-----------DPLD-KV-GAGDQGIMFGYACDETPELMPLPISLAHRLALRLAQVRHDG--S-LGYLLPDGKTQVSVVY--E-D--D--R-PVAIDTI-LISTQH-TAEVEGISDEKAV-QQ----RIRE-DLWSHVVLPATADLPLKP-S-------K-------EGT--RFYVNPTGKFVVGGPQGDAGLTGRKIIVDTYGGYARHGGGAFSGKDPTKVDRSAAYAARYVAKALVASGLAHKAEVQLSYAIGVARPVSILVESFGTGA--LSNA----DLTALVQEHFDLRPGAIIAAFGLRELPQQRGGR-FYQDVAAYGHFGR-K----------------------DLD---L--P-WEDV-TAIA-----ATLK----Q----ATANRVSAVSGG--------

>Cyanobacteria_Vulcanococcus_limneticus

-----------MG------------SR-Y-VFTSESVTEGHPDKICDQVSDAVLDALLAQDP-----ASRVACETVVNTGLCLITGEVTTNA-RV--------DFNTLVRGVIERIGYSG--ARAGGFDARSAAVLIALDQQSPDIAQGV-NE-ADDHD-G-----------DPLD-LI-GAGDQGIMFGFAVDETPELMPLPISLAHRLARRLAEVRHNG--T-LGYLLPDGKTQVSVVY--E-D--D--V-PVAIDTI-LISTQH-TAEIDGISEEKAL-RD----RIAA-DLWVHVVEPATADLPLKP-S-------K-------DTT--RFLVNPTGKFVVGGPQGDAGLTGRKIIVDTYGGYARHGGGAFSGKDPTKVDRSAAYAARYVAKALVSAGLAKKVEVQLSYAIGVAKPVSILVESFGTGA--ISNA----DLTALVQEHFDLRPGAIIEAFGLRQLPQQRGGR-FYQDVAAYGHFGR-T----------------------DLN---L--P-WENV-EAIA-----ETLK----Q----ATAGRVAAGSAS--------

>Cyanobacteria_Nostoc_piscinale

---------M---------------KKDF-MFTSESVTEGHPDKLCDQISDAIVDRFLQQDP-----YARVITECAASTGILFIAARFEPNA-NV--------DFTNIARQIIEQIGYEQ-----KQFNSKTCSILTSLRELPASQTHLF-DE-------K----NLS-D--EEIE-KI-TVTNQVTVFGFACNQTYTLMPLPIWLAHKLARQLSEVRHKN--I-LPYLTPDGKTQVGVEY--R-D----RR-PYRIHSITVIASQNKAGKPD---------YQ----QLQN-DIQETVINPVFENEEIRP---------D-------AKT--RIFINPDGPFIKGGPAVHSGLTGRKNAIDTYGEYSKHSGSALSGKDPIRIDRIGAYIARYAAKNIVAAKLADECEVQLSYSIGLSRPVSIQVETFGTGK--ISDE----EITNLLEKHFDFRLAGIIKQFNLRHLPTINPGG-FYRQLAVYGHVGR------------------M-----DID---L--P-WEKT-DK-V-----GVF------------------------------

>Deferibacteres_Geovibrio_thiophilus

---------M-DQ------------KK-Y-VFTSESVTEGHPDKIADQISDAILDAMLTDDP-----MSRVACETMVTTGLVIVAGEVTTRT-YV--------DIPGVVRETIKDIGYTR--AK-YGFDYETCGVISTIDKQSPDIAMGV-DT-G-------------------------GAGDQGLMFGFACDETEELMPLPIMLAHKLTMQLSKVRKNE--V-LPYLRPDGKSQVTIEY--D-G--Y--K-PVRVDTV-VISSQH-SNEIK---------LK----DLRE-DIIEKVIKPV-----I-P-A-------D-L-LDE-EHI--KYHINPTGRFVIGGPMGDCGLTGRKIIVDTYGGAGRHGGGAFSGKDPSKVDRSAAYAARWVAKNIVAGGFAKRCEIQVAYAIGVAEPVSVNVNTFGTST--VPES----EIERAVGKVFDLTPKGIMEALDLRK-P-------IFRKTAAYGHFGR-N------------------------E---F--S-WEKT-NK-V-----DELK----K----IIG-----------------

>Deferribacteres_Flexistipes_sinusarabici

---------M-DK------------KK-Y-VFTSESVTEGHPDKIADQISDAILDSMLSEDP-----FSRVACETMVTTGLAIVAGEITTRT-YV--------DIPGVVRDTVKEIGYTR--AK-YGFDYETCGVMTSIDKQSPDIAQGV-DT-G-------------------------GAGDQGLMFGFACDETEELMPLPIMLAHKICMQLSDVRKSE--I-LPYLRPDGKSQVTIEY--D-G--F--K-PVKVDTV-VVSTQH-ADDIK---------LT----DLKE-DIVEKVIKPV-----I-P-K-------H-L-LDD-EDI--TYHINPTGRFVVGGPMGDCGLTGRKIIVDTYGGSGRHGGGCFSGKDPSKVDRSAAYAARWVAKNIVASGVASRCELQLAYAIGVVEPVSVSVNTYGTGA--VPDS----ELEKIVRKTFDLTPKGIMEALDLRK-P-------IYKSTAAYGHFGR-D------------------------G---F--S-WEDT-AR-A-----QDIK----D----LAGKLGS-------------

>Deferribacteres_Deferribacter_desulfuricans

---------M-ER------------KK-Y-VFTSESVTEGHPDKVADQISDAILDAMLKDDP-----YSRVACETMVTTGLAVVAGEITTRT-YV--------DIPGLVRETIKEIGYTR--AK-YGFDYETCAVITTIDKQSPDIAMGV-DT-G-------------------------GAGDQGLMFGFACDETEELMPLPIMLAHKICKQLSVVRKQG--I-LDYLRPDGKSQVTIQY--E-G--F--K-PVKIDTI-VVSTQH-SPDVK---------LK----DLKE-DVIEKVIKPV-----V-P-Q-------E-L-LDE-EHV--TYHINPTGRFVIGGPMGDCGLTGRKIIVDTYGGVGRHGGGCFSGKDPSKVDRSAAYAARWVAKNIVAAGLARRCEIQLAYAIGVAYPVSIMVNTFGTGI--IPDE----EIEKIVKQVFDLTPKGIIEALDLRK-P-------IYKQTAAYGHFGR-K------------------------E---F--S-WEQT-NK-A-----DELK----E----LAGKLGA-------------

>Deferribacteres_Calditerrivibrio_nitroreducens

---------M-KN------------RK-Y-VFTSESVTEGHPDKVADQISDAILDAMIKDDP-----NSRVAVETLVTTGLCIVAGEVTTST-YV--------DLPEVIRNTIKDIGYTR--AK-YGFDYETCAVISTIDKQSPDIAMGV-DT-G-------------------------GAGDQGLMFGFACDETEELMPLPIMLAHKICMKLAEVRKKE--I-LSYLRPDGKSQVTIEY--N-G--Y--T-PLRVDTV-VVSAQH-SPDVK---------HK----DLKE-DIIEKVIKEV-----I-P-S-------N-L-LDE-ENI--TYHINPTGRFVVGGPMGDCGLTGRKIIVDTYGGSGRHGGGAFSGKDPSKVDRSGAYAARWVAKNIVAAGLANRCEVQIAYAIGVAEPVSLMVNTFDTGI--IPDE----EISNIVKKIFDLTPKGIITALDLKK-P-------IYKKTAAYGHFGR-K------------------------G---F--S-WEET-NY-A-----DEIK----K----MAGKLGA-------------

>Deferibacteres_Seleniivibrio_woodruffii

---------M-NN------------KK-Y-LFTSESVSEGHPDKIADQISDAILDAMLADDP-----NSRVACETLVTTGLAVISGEVTTNT-YV--------DIQSTVRETIRQIGYTR--AK-FGFDADTCGIIITLNKQSPDIAMGV-DT-G-------------------------GAGDQGLMFGYACDETPELMPMPIMLAHKITKKLSEVRKSG--E-LSYLRPDSKSQVTIEY--D-G--F--T-PLRVDTV-VVSSQH-SEDIK---------MD----TLKR-DIIEKVIKPV-----I-P-S-------D-M-LDD--NC--KYHINPTGRFVIGGPMGDCGLTGRKIIVDTYGGMGRHGGGAFSGKDPSKVDRSAAYAARWVAKNIVAAGLAKRCEIQVAYAIGVAEPVSVMVNTFGTSE--VDEK----LIEKAVKEVFDLTPKGIMSALDLRK-P-------IYRKTAAYGHFGR-N------------------------E---F--S-WEQT-DK-V-----DALL----A----AIR-----------------

>Deferribacteres_Denitrovibrio_acetiphilus

---------M-NN------------KN-Y-LFTSESVSEGHPDKMADQISDAVLDAMIKDDP-----YSRVACETLVTTGLVVVSGEVTTNT-YV--------EIPEIVRETVRNIGYRN--SA-HGFDCDTCGVMVTLDKQSPDIAMGV-DV-G-------------------------GAGDQGLMFGFACDETPELMPLPITLSHKLTKKLSELRKSG--E-LNYLRPDSKSQVTIEY--D-G--F--N-PVRVDTV-VISTQH-SEDIH---------LD----DLKK-DIIQ-VIKGV-----I-P-Q-------E-L-NAG--DI--KYHINPTGRFVIGGPMGDCGLTGRKIIVDTYGGMGRHGGGAFSGKDPSKVDRSAAYSARWVAKNIVAAGLAKRCELQVAYAIGVVDPVSIMVNTFGTSV--VPEE----KIEKAVREVFDLTPKGIMDALDLRR-P-------IYKSTAAYGHFGR-S------------------------E---F--P-WENT-DK-I-----ADLK----T----AIG-----------------

>Deferribacteres_Mucispirillum_schaedleri

---------M-DN------------RN-Y-LFTSESVTEGHPDKMADQISDAILDAMLKDDP-----FSRVACETMLTTGLVIVSGEVTTKT-YV--------DIPAVVRNTVAGIGYTR--AK-FGFDHETCGVISTINHQSPDIAMGV-DT-G-------------------------GAGDQGLMFGFACNETPELMPLPIMLAHKITMKLSEVRKSG--E-LDYLRPDGKSQVTIEY--N-G--F--E-PVKVHTV-VISSQH-SDSVS---------ME----QLKH-DIIEKVIKPV-----M-P-A-------K-L-FNE-NEC--IFHINPTGRFVIGGPMGDCGLTGRKIIVDTYGGMGHHGGGAFSGKDATKVDRSAAYGARWVAKNIVAAGLAKRCEVQLAYAIGVSEPVSIMVNTFYTGI--IPDS----EIEKIVSKVFDLTPKGLISALDLRK-P-------IFQKTASYGHFGR-G------------------------E---F--A-WEDT-NK-V-----ADIK----D----LAGKLGS-------------

>Deinococcus_Thermus_thermophilus

---------M-RA-------------L-R-LVTSESVTEGHPDKLADRISDAILDALIAQDK-----KARVAAETLVTTGLVFVAGEITTEG-YV--------DIPNLVRKTVREVGYTR--AK-YGFDADTCAVLTAIDEQSPDIAGGV-NL-SYEWR-V----LKST---DPLD-RV-GAGDQGLMFGYATDETPELMPLPITLAHRLTMRLAEVRKTG--L-LPYLRPDGKAQVTVVY----E--G-DK-PLYVKTV-VVSAQH-SPEVE---------QE---Q-LRE-DLIREVVRQA-----I-P-PEY---------LKD-GET--EYLINPSGRFILGGPHADTGLTGRKIIVDTYGGAVPHGGGAFSGKDPTKVDRSASYYARYMAKNIVAAGLARRALVELAYAIGKARPVSLRVETFGTGV--LPDE----KLTEIAKKVFDPRPLAIIEELDLLR-P-------IYTPTSAYGHFGR-P------------------------G---F--P-WEET-DR-VEALRREALR----R----EAGL----------------

>Deinococcus_Marinithermus_hydrothermalis

--------------------------M-R-LVTSESVTEGHPDKLADRISDAILDALIAQDP-----KARVACETLVTTGLVFVAGEITTEG-YV--------DIPGLVRKTIKDVGYTR--AK-FGFDGDTCAVLTAIDEQSPDIAGGV-NE-SYEVR-V----LKST---DPLD-RV-GAGDQGLMFGYATDETPELMPLPIMLAHKLTMRLAEARKTG--E-LEYLRPDGKSQVTVVY----D--G-EK-PLYVKTV-VISTQH-SPEME---------QD---Q-IRE-DVIQRVVRRA-----I-P-EEY---------LSE--ET--EYLINPSGKFVIGGPHGDTGLTGRKVIVDTYGGAVPHGGGAFSGKDPTKVDRSASYYARYMAKNIVAAGLAKRALVELAYAIGKARPVGLRVETFGTGV--LPDE----KIAEIARKVFDPRPQAIIEQLDLLR-P-------IYTPTSAYGHFGR-T------------------------G---F--P-WEAT-DR-V-----EALR----Q----EAGL----------------

>Deinococcus_Meiothermus_taiwanensis

---------M-R----------------R-LVTSESVTEGHPDKLADRISDAVLDAILAQDP-----KARVACETLVTTGLVMVAGEITTDS-YV--------DIPRLVRQTVLEVGYTR--AK-YGFDGDTCAVLTAIDEQSPDIAGGV-NE-SWEWR-V----LGSR---DEFD-RV-GAGDQGLMFGYATDETPELMPLPISLAHRLTRRLAEARKTG--E-IPYLRPDGKAQVTVVY----E--G-QK-PLYVGTA-LVSAQH-SEEVE---------AD---Q-IHH-DIRTKVIEKA-----I-P-EEY---------LSK--ET--LYLVNPSGKFVIGGPHGDTGLTGRKIIVDTYGGAVPHGGGAFSGKDPTKVDRSAAYYARYIAKNIVAAGLAKRALIELAYAIGKARPVGMRVETFGTGI--VPDE----KITEAAQKVFDARPRAIIENLNLQR-P-------IYTPTSAYGHFGR-E------------------------G---F--P-WENT-DK-V-----EELR----K----LLP-----------------

>Deinococcus_Oceanithermus_profundus

---------M----------------L-R-LVTSESVTEGHPDKLADRISDAILDAILAKDT-----AARVAAETLVKTGMVLVAGEITTKA-YV--------HLPDLVRRTVKEVGYTR--AK-FGFDGDTCAVLSAIDEQSPDIAGGV-NT-AYEVR-V----LGST---DPYD-QV-GAGDQGLMFGYATRETEELMPLPITLAHRLTRRLAEVRKSG--E-LPYLRPDGKAQVTVAY----D--G-DR-PLYVETV-VLSAQH-APDID---------PD---E-LRD-DLIEKVVRQA-----I-P-AGL---------LRD--DT--HFLINPSGRFVLGGPHGDAGLTGRKIIVDTYGGAVPHGGGAFSGKDPTKVDRSGAYYARYMAKNLVAAGLAERALVEVAYAIGKARPVGLRVETFGTGT--LTDS----QITEVARKVFDPRPLAIIEELELLR-P-------IYTETSAYGHFGR-G------------------------F---F--P-WEET-RR-V-----ADLL----Q----AAGG----------------

>Elusimicrobia_Elusimicrobium_minutum

---------M-LKE-----------GR-Y-YFTSESVGEGHPDKMCDQISDAVLDAILAKDS-----TARVACETYVTRGLVVIGGEITTKT-WV--------DVDSIAREEIKRIGYTD--PK-YGFNYETCAILNVIGKQSPDISQGV-DV-G-------------------------GAGDQGFMVGYAVNETKEYMPITIVLANEIVKNLAVCRKNG--K-LKFTGPDCKSQITFEYI---N--G--K-PARIDTV-VVSTQH-TSDVLDKSGQFI--TE----AAQK-KIIDAAVMPV-----I-------K---K-W-CD--KNT--KILINPTGKFVIGGPQSDTGMTGRKIIVDTYGGRCPHGGGAFSGKDPTKVDRSAAYMARYIAKNIVAAKLAEECTIQIAYAIGKAEPVGVYVYTHGTGK--VSDE----KLTLAAKKVFPLTPKGIIEHFKLRR-P-------VYKQTAAFGHFGR-A----------------------G-----F--P-WEKT-DK-T-----KALI----D----AVR-----------------

>Elusimicrobia_Endomicrobium_proavitum

---------M-AIK-----------NG-Y-FFTSESVTEGHPDKVCDIVSDSILDAILKQDS-----KARVACETFVSPGLLVVGGEITTSA-KI--------NTKKVIRNAIKNIGYDR--PE-FGFDYRHCAIADIINAQSPDIAQGV-DI-G-------------------------GAGDQGLMIGFACKETPELMPLPVILAHKLARKLTEVRKKN--I-LPYLGPDGKTQVTVEYV---D--W--K-PKRIDAV-VLSTQH-TEDILDKTGNRI--TE----KSKK-EIFEKIIKPV-----L-------G---K-Y-ID--KET--KIYINPTGKFVIGGPQGDTGLTGRKIIVDTYGGMAAHGGGAFSGKDSTKVDRSASYMARHIAKNIVAAGFAEKCTIQLAYAIGVAEPVSVMVDTHHSGK--VSGT----VLEPIVRKVFPLTPKGITDYLQLRK-P-------IFAQTAAYGHFGR-S----------------------EKE---F--T-WEKV-NK-V-----AELK----K----YVKI----------------

>Elusimicrobia_Endomicrobium_trichonymphae

---------M-SNK-----------QS-Y-FFTSESVTEGHPDKVCDIISDSVLDAILKQDS-----KARVACETFISKGLFIVGGEITTSA-RI--------NTRDIIKEAIKKIGYDK--LS-FGFDCNTCVIIDAINTQSHDVAVGV-DT-G-------------------------GAGDQGLMIGFACRETPEFMPLPIILAHKLAMRLTEVRKKG--I-LPYLGPDGKTQVTVEYV---D--W--K-PKRIDTV-VLSTQH-TEKVLDRTGNRM--TK----KSKE-EIVNKVIKYV-----L-------G---K-L-ID--KNT--KIYINPAGRFLSGGPSADTGLTGRKIIVDTYGGMTAHGGGAFSGKDSTKVDRSACYMARHIAKNVVAAGFADKCTVQLSYAIGMADPVSVMVDTHHSRK--ISGS----VIESAVRKIFPLTPEGITDYLQLRR-P-------IFAQTAAYGHFGR-E----------------------GKD---F--T-WEKL-NK-V-----SELK----RECGGYVAE----------------

>Fibrobacteres_Chitinispirillum_alkaliphilum

-------------------------MR-R-FFTSESVSQGHPDKVCDQISDAILDAMLAQDS-----ESRVACETLVTTGLVVVAGEITSKA-VV--------DVQEIVRKTIREIGYNS--TE-LGFDADSCGILLSLHQQSPDISQGV-TQ-GEGLY-K----------------EQ-GAGDQGIMFGYACNETETYMPMGIYYAHRLMEELVVQRESG--K-IPYLRPDSKSQVSVEY--D-D--L--T-PKRIDTV-VISTQH-DPDVD---------HA----TIQK-DMAE-VVKKV-----I-P-A-------E-L-LD--SET--VYHINPTGRFVVGGPHGDSGVTGRKIIVDTYGGYGAHGGGAFSGKDPSKVDRSAAYAARWVAKNIVAAGLATHCEIQLSYAIGVAKPLSIHVDTYGTGK--LSEL----QICEIICELFDLTPSGIIERLNLKR-P-------IYRETARNGHFGR-E----------------------LPD---F--T-WEKT-DM-A-----EAIK----K----AAKL----------------

>Fibrobacteres_Chitinivibrio_alkaliphilus

-------------------------MG-I-LFTSESVSQGHPDKVADQISDAILDAMLAQDP-----HSRVACETMVTTGLVIIAGEVTTTA-IV--------DIQDVVRNTIKEIGYTD--PA-IGFDYENCAVMVTLDKQSPDISQGV-TK-GEGLF-A----------------EQ-GAGDQGMMFGYACKETPELMPLAIHMSHRLVEKVASLRETG--V-LPYLRPDTKSQVTVEY--D-DDGL--T-PRRVDAV-VISTQH-TEDVS---------HE----QIEK-DMIEKVISQV-----I-P-A-------S-Y-LD--ENT--KYFINPTGRFVIGGPKGDCGLTGRKIIVDTYGGYGAHGGGAFSGKDPSKVDRSAAYATRWVAKNIVAAGLATHCEVQVAYAIGVAEPMSLTVNTYGTGT--VSDA----EIIAAVQRVFDLRPAAIISALNLQR-P-------IFRETARNGHFGR-E----------------------DAH---F--T-WEKT-DK-A-----EALK----Q----ALA-----------------

>Firmicutes_Macrococcus_epidermidis

---------M-T-------------DR-K-LFTSESVTEGHPDKIADQISDAILDAILTGDP-----KARVACETSVTTGMALIAGEITTST-YV--------DIPKVVRDTVKEIGYTR--AK-FGYDFQTMAVLTAIDEQSPDIAQGV-DR-ALEDR----G-NLS-D--SDIE-SI-GAGDQGLMFGFACNETESLMPLPISLSHELARRLTAVRKDG--T-LEYLRPDGKTQVTVEYD-E-A--G--K-PKRIDTI-VISSQH-HEKIE---------LE----QIQA-DIKEHVIKPV-----V-P-A-------E-L-ID--EET--KYFINPTGRFVIGGPQGDAGLTGRKIIVDTYGGYARHGGGAFSGKDATKVDRSGAYAARYVAKNIVAAGLADKCEVQLAYAIGVAQPVSISVETYGTSE--YTEQ----QLVDAVRKTFDLRPAGIIQMLDLRR-P-------IYKQTAAYGHFGR-T----------------------DVD---L--P-WEAT-DK-I-----EDLK----A----NLNK----------------

>Firmicutes_Staphylococcus_fleurettii

---------M-TY------------NR-R-LFTSESVTEGHPDKIADQISDAILDEILKGDK-----NARVACETTVTTGMALIAGEISTST-YV--------DIPKVVRETVKEIGYTR--AK-YGYDYKTMSVLTAIDEQSPDIAQGV-DR-ALEYR----D-EMN-D--EELN--T-GAGDQGLMFGFATNETDTLMPLPIDLSHKLSKRLSEVRKNG--T-LDYLRPDGKVQVTVEYD-E-Q--N--Q-PKRIDTI-VVSSQH-HEEIE---------LE----KIQN-DIKEYVIYPT-----V-D-P-------S-L-LD--EET--KFFINPTGRFVIGGPQGDAGLTGRKIIVDTYGGYARHGGGAFSGKDPTKVDRSAAYAARYVAKNIVAAQLADKCEVQLAYAIGVAQPVSIAIDTFGTGK--VSED----TLIQAVRDHFDLRPAGIIKMLDLQR-P-------IYKQTAAYGHFGR-N----------------------DVQ---L--P-WEKT-DK-I-----ELLK----E----AVSALQ--------------

>Firmicutes_Staphylococcus_epidermidis

-----M---M-TY------------NK-R-LFTSESVTEGHPDKIADQVSDAILDEILKDDP-----NARVACETTVTTGMALISGEISTTT-YV--------DIPKVVRETIKDIGYTR--AK-YGYDSQTMAVLTAIDEQSPDIAQGV-DK-ALEYR----N-DIS-E--EEIE-AT-GAGDQGLMFGYATDETDTYMPLPIFLSHQLAKRLADVRKDE--I-LDYLRPDGKVQVTVEYG-D----D--K-PRRIDTI-VVSTQH-AEDVE---------LA----QIEK-DIKTHVIYPT-----V-D-K-------A-L-LD--DET--KFYINPTGRFVIGGPQGDAGLTGRKIIVDTYGGYARHGGGCFSGKDPTKVDRSAAYAARYVAKNIVAAGLAKQCEVQLAYAIGVAEPVSISINTFDTGK--VSEA----RLVEAVRKHFDLRPAGIIKMLDLKQ-P-------IYRQTAAYGHFGR-T----------------------DVL---L--P-WEKL-DK-V-----NVLK----D----AVE-IQ--------------

>Firmicutes_Pediococcus_ethanolidurans

-----------MQ------------ER-H-LFTSESVSEGHPDKIADQISDAILDAMLAQDP-----QARVACETTVTTGLVVVVGEISTTA-YV--------DIQKTVRQTIKDIGYKD--GE-YGFDGDNCAVMVAIDEQSPDIAQGV-DD-SLENR-E--G-DE-----DPLD-KI-GAGDQGLMFGFAIDETPELMPLPIMLSHHLMQKTASVRKSG--E-LQYLRPDAKAEVTVEYD-D-N--E--K-PVRVDTV-VLSTQH-DPNVL---------LE----TIRK-DVVEHIIKPV-----I-P-A-------N-L-LD--DKT--KYFINPTGRFVIGGPQGDAGLTGRKIIVDTYGGFARHGGGAFSGKDATKVDRSASYAARYIAKNIVAAGFAHKVEVQLAYAIGVAQPVSVSVDTFGTGS--VSEE----KLAAAIRKVFDLRPAGIIKMLDLQR-P-------IYKKTAAYGHFGR-T----------------------DID---L--P-WEHT-DK-V-----DELK----A----LLK-----------------

>Firmicutes_Lactobacillus_salivarius

-----------MT------------EK-H-LFTSESVSEGHPDKIADQISDSILDAMLEQDP-----EARVAVETTVTTGLVVVVGEVSTTA-HI--------DIQKVVRETIKEIGYKN--GK-YGFDGDNCAVITAIDEQSPDIALGV-DN-SLEVK-N--G-DV-----DPLD-QI-GAGDQGMMFGYAINETPELMPLPISLSHHLMKKVAEVRKNG--T-LAYLGPDAKAQVTVEYD-D-N--N--Q-PLRIDTV-VLSTQH-DENIS---------LD----QIRK-DVMDQVITKV-----I-P-D-------K-Y-LD--SNT--KYFINPTGRFVIGGPQGDAGLTGRKIIVDTYGGYAHHGGGAFSGKDATKVDRSASYAARYIAKNIVAAGLASQVEVQISYAIGVAQPVSVSVNTFGSGK--VPNQ----KLVDAIRKVFDLRPAGIIKMLDLRR-P-------IYKKTAAYGHFGR-T----------------------DID---L--P-WEKT-DK-I-----NELK----K----ELDF----------------

>Firmicutes_Vagococcus_penaei

-----------MK------------ER-R-LFTSESVSEGHPDKVADQISDAILDAILEKDP-----YARVACETSVTTGLVLVFGEITTTA-YV--------DIQKVVRDTIKGIGYTR--AK-YGFDGDTCAVMVAIDEQSPDIAQGV-NE-SLEKK-E--G-LH-----DADE-AL-GAGDQGLMFGFAINETPELMPLPIALSHRLTKRLADLRKSE--T-LSYLRPDAKSQVTVEYD-E-N--G--H-PLRVDTV-VISTQH-DEDVS---------LE----TIKK-DLIEHVVKFE-----I-P-N-------E-L-LD--EQT--KYYINPTGRFVIGGPQGDSGLTGRKIIVDTYGGYARHGGGAFSGKDATKVDRSASYAARYIAKNIVAAGLAEKCEVQLAYAIGVAEPVSISVETFGTSV--VPES----KLIEAIRALFDLSPVGIINMLDLRR-P-------IYQKTAAYGHFGR-D----------------------EVE---F--T-WERT-DK-V-----DQLK----A----FVGK----------------

>Firmicutes_Alkalibacterium_pelagium

------------M------------EK-R-LFTSESVTEGHPDKIADQISDAILDAILSKDP-----EARVACETLVTTGLVLIAGEIRTET-YV--------DMQKIARDTIRDIGYTR--AK-YGFDADTCAVVVSIDDQSPDIAVGV-DD-SVEYK-S--E-AQ-----DELD-KI-GAGDQGLMFGYATKETPELMPLPISLSHKLTRQLAKVRKNK--S-LDYLRPDGKAQVTVEYD-E-N--D--N-PRRIDAV-VISTQH-RPDIT---------AD----QLEK-DIMEKVITQV-----I-P-S-------K-W-LD--NDT--KYYINPTGRFVVGGPQGDAGLTGRKIIVDTYGGYARHGGGAFSGKDGTKVDRSASYVARYIAKNIVAADLADKVEVQLAYAIGVSQPVSISIDTFGTGK--ISNE----RLIKAVRELFDLRPAGIIKMLDLKR-P-------IFKQTAAYGHFGR-T----------------------DVD---L--P-WEKT-DK-A-----DELR----K----LM------------------

>Firmicutes_Lacticigenium_naphtae

------------M------------EK-R-LFTSESVSEGHPDKIADQISDAILDAILTDDP-----DARVACETLVTTGLVLIAGEIRTNT-YV--------DMQKIARETIREIGYTR--AK-YGFDADTCAVLVSIDEQSSDIALGV-DD-STEFK-S--S-NV-----DELD-KI-GAGDQGLMFGFAVNETPELMPLPIALSHLLTHRIAEVRKKR--I-VPYLRPDAKAQVTVEYD-E-N--D--K-PKRIDAI-VVSTQH-HPDIE---------YS----KLKE-DVMKHVVRYV-----I-P-A-------K-F-LD--KDT--KYYINPTGRFVVGGPQGDAGLTGRKIIVDTYGGYARHGGGAFSGKDPTKVDRSASYAARYIAKNIVAAELAEKCEVQLAYAIGVAQPVSISIDTFKTGK--VSED----DLIQAVRKHFDLRPAGIIQMLDLQR-P-------IYRQTAAYGHFGR-T----------------------DIE---L--P-WEKL-DK-V-----DVLK----Q----SLNQ----------------

>Firmicutes_Carnobacterium_divergens

-----------MT------------ER-R-LFTSESVSEGHPDKIADQISDGILDAILAKDP-----LARVACETTVTTGLVLVVGEISTTT-YV--------DIQKVVRETIRKIGYTR--AK-FGFDADTCAVMVAIDEQSSDIAQGV-DD-SLEFK-S--K-DE-----DALD-QI-GAGDQGLMFGFAINETPELMPLPIALSHRLTKRLADVRKEG--L-LNYLRPDAKSQVTIEYD-E-K--G--L-PLRVDTI-VISTQH-HPDTT---------LE----ELQS-DIKEHVIKAV-----I-P-P-------E-L-LD--NET--KYFINPTGRFVIGGPQGDSGLTGRKIIVDTYGGYARHGGGAFSGKDPTKVDRSASYAARYIAKNIVAAKLADKCEVQLAYAIGVAQPVSIAIDTFGTGK--VLES----KLIEAVRKNFDLRPAGIIEMLDLRR-P-------IYQQTAAYGHFGR-T----------------------DIE---L--P-WEQL-DK-I-----EALK----A----SATE----------------

>Firmicutes_Enterococcus_faecalis

-----------MT------------ER-H-LFTSESVSEGHPDKVADQVSDAILDAILEKDP-----MARVACETSVTTGLVLVFGEISTTA-YV--------DIQKIVRQTVKDIGYTR--AK-YGFDGETVAVLVAIDEQSPDIAQGV-DA-AIEVR-D--Q-DE-----K--D-DI-GAGDQGLMFGFAVDETPELMPLPIALSHRLVRRLAELRKEK--V-LPYLRPDAKSQVTVEYD-D-Q--G--Q-PQRVDTI-VISTQH-DDETT---------LE----QIEK-DIKEQVINEV-----I-P-H-------E-L-LD--DET--KYFINPTGRFVIGGPQGDAGLTGRKIIVDTYGGYARHGGGAFSGKDATKVDRSASYAARYIAKNIVAAGLAKKVEVQLAYAIGVAQPVSISINTFGTSD--LPES----KLIEAVRKNFDLRPAGIIEMLDLRR-P-------IYKQTAAYGHFGR-T----------------------DID---L--P-WEQT-DK-V-----EALK----A----SLAE----------------

>Firmicutes_Catellicoccus_marimammalium

---------M-EK------------EK-K-LFTSESVTEGHPDKLADQISDAILDAILEKDP-----MARVACETAVTTGLVLVFGEITTST-YV--------DIQKVVRQAIKDAGYTR--AK-YGFDGDTCAVQVAIDEQSPDIAQGV-DQ-ALD-Q-S--T-DE-----N--S-DT-GAGDQGLMFGFAIDETEELMPLPIALSHRLTKRLAEVRKEG--I-LPYLRPDGKAQVTVEYD-E-N--D--K-AKRIDTI-VLSTQH-DPDVS---------LE----QIQK-DMRTYVIDEV-----M-P-K-------E-L-LD--EDT--KYYINPTGRFVIGGPCGDAGLTGRKIIVDTYGGYARHGGGAFSGKDATKVDRSASYAARYIAKNIVAAGLAHKCEVQLAYAIGVAEPVSIAIDTFGTSE--YTEE----QLIAAVRKVFDLRPAGIIKMLDLRR-P-------IYKQTAAYGHFGR-T----------------------DVD---L--P-WERT-DK-V-----DALK----A----ALTK----------------

>Firmicutes_Tetragenococcus_koreensis

-----------MK------------ER-R-LFTSESVSEGHPDKIADQISDAILDALLEQDP-----MARVACETSVTTGLVLVFGEVSTSA-YV--------DMQKVVRQTIKDIGYDR--PE-YGFDGDNVAVLVAIDEQSQDIAQGV-DT-SLEAR-E--D-KE-----ERIN-ST-GAGDQGLMFGYATDETPELMPLPISLSHKIVARLAQLRKSE--E-ISYLRPDAKSQVTVEYD-E-N--D--Q-PKRIDTV-IISTQH-NDDVA---------NE----TIRR-DMIEKVIKVV-----I-P-N-------E-L-LD--EQT--KYFINPTGRFVIGGPQGDAGLTGRKIIVDTYGGYARHGGGAFSGKDATKVDRSASYAARYIAKNIVAAKLADKVEVQLAYAIGVAEPVSIAIDTFGTGK--VSQE----KLIEAIRQNFDLRPAGIIEMLDLRR-P-------IYKDTAAYGHFGR-T----------------------DIN---L--P-WEKT-DK-V-----EILQ----K----SVK-----------------

>Firmicutes_Globicatella_sanguinis

-----------MT------------EK-R-LFTSESVTEGHPDKIADQISDAILDAILEQDP-----NARVACETAVNTGLVLVFGEITTSA-YV--------DIQQIVRQKIDEIGYNK--QQ-YGFDAKTVAVLVSLDEQSPDIAQGV-NN-AIETR------DQDSE-----I-EI-GAGDQGLMFGYATDETPELMPLPISLSHQLALKLAEVRKSG--E-LNYLGPDGKTQVTIEYD-E-T--G--V-AQRVDTI-VISTQH-TEDVT---------LQ----QIHD-DVIAHVVKPI-----I-P-S-------E-L-LD--DET--KYFINPTGKFIIGGPVGDSGLTGRKIIVDTYGGYARHGGGAFSGKDATKVDRSASYAARYIAKNIVAAGLAKRCEVQLAYAIGVAEPVSISIDTFNTSE--ITES----KLIAAVRSTFRLTPKGIIEMLDLLR-P-------IYAQTAAYGHFGR-R----------------------DIE---L--P-WEQT-DK-V-----EALK----A----AINA----------------

>Firmicutes_Salinicoccus_luteus

-----------MA------------ER-R-LFTSESVTEGHPDKIADQISDAILDEILKGDP-----HARVACETVVNTGMALIVGEISTNT-YV--------DIPKIVRETVKDIGYVL--AK-YGYDYKTMSVLTGIDEQSSDIAQGV-NN-AVETR-E--T-DAV---------ST-GAGDQGLMFGFACDETEELMPLPIALAHKLSRRLTEARKEN--I-IDYLRPDGKTQVTVEYD-E-N--D--Q-VKRVDTI-VISTQH-HEEVE---------KE----TIQR-DLLTHVIREV-----V-P-A-------E-L-LD--EET--KYIINPTGRFVIGGPQGDAGLTGRKIIVDTYGGYARHGGGAFSGKDPTKVDRSAAYAARYIAKNIVAAGIASKCEIQLAYAIGVSEPVSISVDTFGTSS--YSEA----DIVAVIRKIFNLSPDGIIDMLDLRR-P-------IYKNTASYGHFGR-E----------------------DQD---F--P-WEKL-DK-A-----AQLK----E----ELSSLKVN------------

>Firmicutes_Trichococcus_alkaliphilus

-----------MV------------EK-R-LFTSESVTEGHPDKIADQISDAILDTLLAADP-----NARVACETIVTTGLVVVFGEITTSA-YV--------NIQQIVRDTVTEIGYTR--GK-FGFDADNLAVMVSLDEQSDDIAIGV-NE-SLERR-E--S-VT-----TDYN-EI-GAGDQGLMFGFAINETEELMPLPISLSHKLAKRLSEVRKDG--T-LEYLRPDGKTQVTVEYD-E-N--G--K-PKRVDTI-VLSTQH-DENVT---------LE----QLKK-DILEHVIHAV-----I-D-D-------A-L-LD--ADT--KYFINPTGRFVIGGPKGDSGLTGRKIIVDTYGGYARHGGGAFSGKDATKVDRSASYAARYIAKNIVAAGLATKCEIQLAYAIGVAEPVSIAIDTFGTSE--YPEA----TLIEAVRKNFNLTPAGIIQMLDLRR-P-------IFKKTAAYGHFGR-E----------------------DAD---F--T-WEKT-DK-T-----EALL----S----YVQAGK--------------

>Firmicutes_Abiotrophia_defectiva

------------M------------ER-K-LFTSESVTEGHPDKVADQISDAILDAILTQDP-----LARVACETAVNTGLVLVFGEVSTTA-YV--------DIQQIVRETIKGIGYNS--SD-YGFDGDNVAVLVALDEQSPDIAQGV-DA-AIETR------DEATD-----Q-LI-GAGDQGLMFGYACDETEEFMPLPIALSHRLAKRLSEVRKSS--Q-LAYLRPDGKSQVTVEYG-E-D--G--Q-ALRVDTV-VISTQH-APEAT---------LE----TVRQ-DVIEQVVKPV-----I-P-A-------H-L-LD--DQT--RFLINPTGRFVIGGPKGDSGLTGRKIIVDTYGGYARHGGGAFSGKDATKVDRSASYMARYIAKNIVAAGLAKRCEVQLAYAIGVAQPVSIRIETFGTGV--VSES----RLVQAVRQLFDLTPDGIIRTLALRR-P-------IYKQTAAYGHFGR-Q----------------------DVV---L--P-WEQV-NK-V-----DELK----A----AIAE----------------

>Firmicutes_Streptococcus_downei

-----------MS------------ER-K-LFTSESVSEGHPDKIADQISDAILDAILEQDP-----DAHVAAETAVYTGSVHVFGEISTTA-YV--------DINRVVRDTIADIGYTN--AD-YGFAADSVGVHPSLIEQSPDIAQGV-NQ-ALEAR-E--G-QE-----DELS-LI-GAGDQGLMFGFAINETPELMPLPISLSHKLVHKLAELRKSG--A-ISYLRPDAKSQVTVEYD-E-M--D--Q-PVRVDTV-VISTQH-DPDVS---------QE----QIRQ-DVIEQVIKAV-----I-P-A-------E-Y-LD--DKT--KYYVNPTGRFVIGGPQGDSGLTGRKIIVDTYGGYARHGGGAFSGKDATKVDRSASYAARYIAKNIVAAGLADKAEVQLAYAIGVAHPVSVRVDTFGTGK--VSES----KLEAAVRENFDLRPAGIIKMLDLKR-P-------IYRQTAAYGHMGR-T----------------------DID---L--P-WEKL-DK-V-----DALK----A----AVQ-----------------

>Firmicutes_Streptococcus_equi

-----------MS------------ER-K-LFTSESVSEGHPDKIADQISDAILDAILAKDP-----EAHVAAETCVYTGSVHVFGEISTTA-YV--------DINRVVRDTIAEIGYTE--AE-YGFSAESVGVHPSLVEQS-DIAQGV-NE-ALEAR-E--G-QS-----DDFN-AI-GAGDQGLMFGFAIDETPELMPLPISLSHQLVRRLATLRKSG--E-ISYLRPDAKSQVTVEYD-E-H--D--K-PVRVDTV-VISTQH-DPEVS---------ND----QIRQ-DMIEQVIKAV-----I-P-A-------H-Y-LD--EKT--RFLINPTGRFVIGGPQGDSGLTGRKIIVDTYGGYARHGGGAFSGKDATKVDRSASYAARYIAKNLVAAGLASKAEVQLAYAIGVAQPVSVRVDTFGTST--VSES----ILEAAVRQVFDLRPAGIIKMLDLKR-P-------IYRQTAAYGHMGR-T----------------------DID---L--P-WEQL-DK-V-----SPLT----E----AVAALSEGA-----------

>Firmicutes_Pilibacter_termitis

-----------MT------------EK-R-LFTSESVSEGHPDKIADQISDAILDEILKQDP-----NARVAAETAVYTGSVHVFGEITTSA-YV--------DINKIVRDTIREIGYTR--AK-FGFDADTVGVHPSIVEQSPDIAQGV-NL-AQEAREN--N-DH-----DPLD-EI-GAGDQGLMFGFAVDETPELMPLPISLSHKLVWKLTELRKTN--R-LPYLRPDAKSQVTVEYD-E-F--D--H-PKRVDTI-VISTQH-DPEVT---------LE----QIKK-DVIEQVIHAV-----I-A-S-------E-L-LD--DET--KIYINPTGRFVIGGPQGDSGLTGRKIIVDTYGGYAHHGGGAFSGKDPTKVDRSASYAARYIAKNIVAAGIAKKLEVQLAYAIGVAHPVSISVNTFGTGE--VSDE----KIIQAIRQFFDLRPAGIIEMLDLKR-P-------IYKQTAAYGHFGR-T----------------------DMA---L--P-WEKL-DK-V-----EILR----D----FFAKS---------------

>Firmicutes_Lactococcus_lactis

-----------MS------------EK-H-LFTSESVSEGHPDKVADQISDAILDAILAQDP-----HAHVACETVVYTGTVNVFGEISTSA-YV--------DIAHVVRETIKKIGYTD--SE-NGFDYKSVGVHVSLVEQSSDIAQGV-NE-AEEVR------DKNGQLVDPLD-LI-GAGDQGMMFGFATNETAEYMPLAISLSHKLVKKLADLRKSG--E-ISYLRPDAKSQVTVEYD-D-N--C--K-AKRIDTV-VISTQH-AASAT---------NE----EIHD-DVINKVIKAV-----I-P-A-------E-L-LD--DET--KYFINPTGRFVIGGPQGDSGLTGRKIIVDTYGGYAPHGGGAFSGKDATKVDRSASYAARYVAKNIVAAGLAEKAQIQLSYAIGVATPTSINVDTFGTGK--VSDD----ELLAAIRKVFDLRPAGIIQMLDLLR-P-------IYGQTAAYGHFGR-T----------------------DVE---L--P-WEQT-DK-V-----EELK----S----VLGK----------------

>Firmicutes_Listeria_newyorkensis

---------M-AR------------NR-H-LFTSESVSDGHPDKIADQISDAILDAILTKDA-----DARVACETTVTTGLVLVAGEITTST-YV--------DIPKIVRDTIKEIGYTR--AK-YGFDAETCAVLTAIDEQSPDIAQGV-DV-ALESR-G--G-SAE-D--AAID-AI-GAGDQGLMFGFATDETEELMPLPISLAHALSRKIAELRKNN--V-LTYLRPDAKTQVTIEYD-N-N--N--Q-PIRVDTI-VISTQH-HPDIS---------QE----QIAT-DLHKLVIEEV-----I-D-A-------R-L-LD--AET--KYFINPTGRFVIGGPQGDAGLTGRKIIVDTYGGYARHGGGAFSGKDPTKVDRSGAYAARYVAKNIVAAGLAKKVEVQLAYAIGVAHPVSISIDTYGTSD--YSEQ----DLINAVNKFFDLRPAGIIKMLDLRR-P-------IYRQTAAFGHFGR-L----------------------DLD---L--P-WEKV-DK-A-----SQMKEFLSQ----TANIN---------------

>Firmicutes_Dolosicoccus_paucivorans

------------M------------EK-R-LFTSESVTEGHPDKIADQISDAVLDEILTQDP-----KARVACEVAVNTGLVLVFGEITTTA-YV--------DIQQIVRQTLKDIGYTK--RE-YGFMADEVAVLVSIDEQSPDIAQGV-DE-SLESR------QTDHT-----P-SL-GAGDQGIMFGYASDQTKEWMPMPIALSHRLAKRLAKVRKDG--T-LAYLGPDGKTQVTVEYD-E-N--N--Q-PYRVDTI-VVSSQH-TEDIT---------LE----ELEQ-DIKQHVINEV-----I-P-N-------E-W-LD--EST--NIYINPTGKFVIGGPKGDSGLTGRKIIVDTYGGYARHGGGAFSGKDATKVDRSASYMARYIAKNIVAAGIAKECEIQLSYAIGKAEPTSIMIETFGTST--TPQE----KLVKAVRELFDLTPSGIIRTLELDR-P-------IFKQTAAYGHFGR-D----------------------DIQ---V--P-WEQL-DQ-V-----DALK----D----KLLS----------------

>Firmicutes_Nosocomiicoccus_massiliensis

------------M------------ER-R-LFTSESVTEGHPDKVADQISDAILDEIIKDDK-----DARVACETVVNTGLAMIVGEISTST-YV--------DIPKIVRNTVQEIGYTN--AE-YGYDYKTMSVLTGIDEQSVDIAQGV-DG-EGDNE-S--K-ESG---------TT-GAGDQGLMFGYATDETKEFMPLPITLAHKLARRLTEVRKTH--V-LDYLRPDGKTQVTVEYD-E-N--D--Q-PLRVDTI-VLSTQH-DEKVA---------HS----EIKE-DIKREVIDYV-----I-D-E-------N-L-ID--EDT--RIIINPTGRFVIGGPHGDAGLTGRKIIVDTYGGYARHGGGAFSGKDPTKVDRSAAYAARYIAKNIVASGLSKQCEIQLSYAIGVAEPVSISVNTFGTGK--IEDS----KIAAAVRNEFTLTPDGIIEMLDLQR-P-------IYKQTASYGHFGR-H----------------------DID---V--P-WEAL-DK-V-----DILK----S----YLN-----------------

>Firmicutes_Facklamia_hominis

-----------MV------------EK-N-LFTSESVTEGHPDKIADQISDAILDAILEQDP-----LARVACETAVNTGLVVVFGEITTSA-YV--------DIQKIVRDKVKSIGYDR--GK-YGFDAENLAVLVSIDEQSPDIALGV-DH-SLESK-E----GFADQ-----A-DN-GAGDQGLMFGFACDETPELMPLPIALAHRLAKRLTQVRKEG--L-LDYLRPDGKTQVTIEYN-D-A--G--I-PERVDTV-VVSTQH-SEEIS---------LD----QLRQ-DIHQQVILQT-----I-P-Q-------Q-L-MD--EQT--KIYINPTGRFTIGGPKGDSGLTGRKIIVDTYGGYSRHGGGAFSGKDATKVDRSASYAARYIAKNLVAAGIAKKIEIQLAYAIGVAQPVSVSVNTFGTSE--YSNH----QIEAAIRELFQLTPSGIIKMLELRQ-P-------IYSQTAAYGHFGR-Q----------------------EPG---F--T-WERL-DK-V-----EAIQ----T----YFKD----------------

>Firmicutes_Facklamia_miroungae

-----M---K-VL------------EK-R-LFTSESVTEGHPDKIADQISDSILDAILEQDP-----MARVACETAVNTGLVMVLGEVTTSA-YV--------DIQKIVRQKVDEIGYNR--GK-FGFDAENLAIIVSLDEQSADIALGV-DS-AIETR-S----DQASE-----Q-EI-GAGDQGLMFGYACDETPELMPLPISLAHKLTRRLTEVRKSG--Q-LDYLRPDGKAQVTVEYD-E-N--D--Q-PVRLEAV-VVSTQH-SEEVS---------LG----QLRE-DILNQVIKPT-----L-S-E-------N-L-LD--DQT--KIYINPTGRFTIGGPKGDAGLTGRKIIVDTYGGYARHGGGAFSGKDATKVDRSACYAARYIAKNIVAAGIAKKIEIQLAYAIGVAQPVSVSVNTFESSN--YSDA----AIVKAIRQVFNLTPKGIIDMLDLRQ-P-------IFAQTAAYGHFGR-E----------------------EAG---F--T-WERT-DK-A-----EELK----S----LMM-----------------

>Firmicutes_Granulicatella_adiacens

------------M------------EK-I-LFTSESVTEGHPDKVADQISDGILDAILAQDP-----TARVACETVVNTGLVVLVGEITTSA-VV--------DYQQVARDTIRKIGYTD--RK-YGYDADAVAVMVSLDKQSPDIAMGV-DD-ALETR------GQSEE--------F-GAGDQGLMFGYATNETKSYMPLPIDLSHKLAYQLAKVRKEK--Q-LDYLGPDGKVQVTIEYT-P-E--H--K-VKRIDTI-VVSTQH-DEEIS---------QE----QIRK-DVLEYVVKPV-----V-P-S-------E-L-LD--EET--RYFINPTGKFVIGGPVGDSGLTGRKIIVDTYGGYARHGGGAFSGKDSTKVDRSASYAARYIAKNVVAAGLADKCEIQLAYAIGVAQPVSIAVDTFGTSH--VPED----KIIEVIRKEFRLTPKGIIETLNLRR-P-------IYQQTAAYGHFGR-T----------------------DIE---L--P-WEQL-NK-V-----EELK----A----YFA-----------------

>Firmicutes_Atopobacter_phocae

------------M------------EK-K-LFTSESVTEGHPDKVADQISDAILDAILAEDP-----DARVACETVVNTGLVVVVGEVSTSA-YV--------NIQEVVRETINTIGYSN--PI-YGFDGNSVAVLVSLDEQSADIAQGV-DA-AIETR------DQAGP-----N-EI-GAGDQGLMFGYASNETPELMPLPISLSHQLARQLAKVRKEQ--T-IPYLGPDGKVQVTVEYD-E-T--D--Q-VKRIDTI-VISTQH-AESAT---------LE----QIKK-DVKEFVIQPI-----V-P-Q-------S-F-ID--DET--KYFINPTGKFVIGGPKGDSGLTGRKIIVDTYGGIGRHGGGAFSGKDSTKVDRSASYAARYIAKNIVKAELADRCEVQLAYAIGVAEPVSLRIDCFGTEK--YPED----QLIQAIRKNFNLTPRGIIEMLDLRR-P-------IFKATAAYGHFGR-E----------------------EEN---F--T-WEKT-DK-V-----DALK----Q----YFQSV---------------

>Firmicutes_Bacillus_novalis

----MS------T------------KR-R-LFTSESVTEGHPDKICDQISDSILDAILAKDA-----NARVAAETSVTTGLVLVAGEITTST-YV--------DIPKIVRETIKGIGYDR--AK-YGFDSETCAVLTSIGEQSPDIAMGV-DQ-ALEAR-E--G-LMS-D--EEIE-AI-GAGDQGLMFGYACNETKELMPLPISLAHKLARKLTEVRKDK--V-LEYLRPDGKTQVTVEYD-E-N--D--N-PVRIDTI-VISTQH-NPEVT---------LE----QIQR-NLKEYVINPV-----V-P-Q-------E-L-ID--DNT--KYFINPTGRFVIGGPQGDAGLTGRKIIVDTYGGYARHGGGAFSGKDPTKVDRSAAYAARYVAKNIVAAGLADKVEVQLAYAIGVARPVSISIDTFGTGK--VSEE----VLVDVVSKNFDLRPAGIIKMLDLRR-P-------IYQQTAAYGHFGR-T----------------------DVE---L--P-WELT-DK-A-----DTLR----Q----EANL----------------

>Firmicutes_Tumebacillus_avium

-----M------A------------KR-Y-LFTSESVTEGHPDKICDQISDSVLDAIFAKDP-----NARVACETSVTTGLVLVAGEITTST-YV--------DIPKIVRETIRDIGYTR--AK-YGFDAETCAVLTSIDEQSADIALGV-DQ-ALEAR-E--G-QMS-D--AEIE-AI-GAGDQGLMFGFAVNETEELMPLPISLAHKLARRLSEVRKNG--T-LPYLRPDGKTQVTVEY--E-G--D--K-PVRIDTI-VISTQH-AEEVT---------LE----QIKK-DLHEHVIVPV-----M-P-T-------E-Y-VN--DAT--KYFINPTGRFVIGGPQGDAGLTGRKIIVDTYGGYARHGGGAFSGKDPTKVDRSAAYAARYVAKNIVAAGLADKAEVQLAYAIGVARPVSVMVDTFGTGK--VADE----KIVELVEANFDLRPAGIIRELDLRR-P-------IYRQTAAYGHFGR-T----------------------DID---L--P-WERT-DK-A-----DTLR----Q----GAGL----------------

>Firmicutes_Planifilum_fulgidum

----MS-------------------SR-S-LFTSESVTAGHPDKICDQISDAVLDAILAKDP-----NARVACETAVTTGLVLVSGEISTTC-YV--------DIPKIVRETIREIGYTR--AK-YGFDADTCAVLTSIDEQSPDIAMGV-DA-ALEKR-E--G-RMT-E--EEIE-AI-GAGDQGLMFGFACNETPELMPLPISLAHRLARRLHQVRTDG--I-LPYLRPDGKTQVTVEY--E-G--D--R-PVRIDTV-VVSAQH-AEDVT---------LA----RIKE-DILEHVIRPV-----V-P-E-------A-L-LD--AGT--KYFINPTGRFVIGGPQGDAGLTGRKIIVDTYGGYARHGGGAFSGKDPTKVDRSGAYAARYVAKNIVAAGLADKCEVQVAYAIGVAQPVSIRVDTFGTGK--VDES----RLTELVRAHFDLRPAGIIRSLDLRR-P-------IYRQTAAYGHFGR-E----------------------DLD---L--P-WERT-DK-A-----EVLR----K----EAGL----------------

>Firmicutes_Seinonella_peptonophila

----MS---E-EK------------QR-S-LFSSESVTAGHPDKICDQISDAILDAILEKDP-----NARVACETAVTTGLVLVSGEISTQC-YV--------DIPKIVRNTISEIGYTR--AK-YGFDATTCAVLTSIDEQSSDIAQGV-DQ-ALEKR-E--G-SMD-E--QEIE-AI-GAGDQGLMFGYACNETPELMPLPISLAHRLARRLHEVRIDG--T-LPYLRPDGKTQVTVEY--E-E--N--R-PVRIDTI-VVSTQH-AEEIE---------HD----QLKQ-EIIRHVIEPI-----V-P-A-------E-M-LD--SQT--HYFINPTGRFVIGGPMGDAGLTGRKIIVDTYGGYARHGGGAFSGKDPTKVDRSAAYAARYVAKNIVAAGLADKCEVQLAYAIGVARPVSIRIDTFGTGR--IDEK----SLIRAVRKHFDLRPAGIIRQLDLRR-P-------LYRQTSAYGHFGR-T----------------------DID---L--P-WEQT-DQ-V-----EALQ----I----ELGTQVKD------------

>Firmicutes_Sediminibacillus_massiliensis

----MA------A------------NR-R-LFTSESVTEGHPDKICDQISDAILDEILEKDP-----NARVACETTVTTGLVLVSGEITTTT-YV--------DIPSIVRKTVKEIGYTR--AK-YGFDAETCAVLTAIDEQSPDIAEGV-NQ-ALEAR-E--G-KTA-D--EAIE-AI-GAGDQGLMFGYANNETPELMPLPISLAHKLSKRLSDVRKDN--T-LSYLRPDGKTQVTVEYD-E-N--D--K-PVRIDTI-VISTQH-HPEIE---------LT----QIQQ-DLKEFVINPI-----V-P-K-------E-L-LD--EQT--KYFINPTGRFVIGGPQGDAGLTGRKIIVDTYGGYARHGGGAFSGKDATKVDRSAAYAARYVAKNIVAAKLADSCEVQLAYAIGVAEPVSISINTFGTGK--VTEE----VLVKAVRELFDLRPAGIIKMLDLRR-P-------IYKDTAAYGHFGR-T----------------------DIE---F--P-WEQT-DK-V-----ESLK----E----YTNA----------------

>Firmicutes_Sporosarcina_psychrophila

-----M------T------------NR-R-LFTSESVTEGHPDKMCDQISDAILDAILAEDP-----NARVACETTITTGLVLVAGEITTTT-YV--------DIPKVVRETVKEIGYVR--AK-YGFDWETSAVLTAIDEQSADIAAGV-DQ-ALEAR-E--G-SMT-D--DELE-AI-GAGDQGLMFGYACNETPELMPLPISLAHKLSFRLAQVRKDE--T-LTYLRPDGKTQVTVEYD-E-N--N--K-PVRIDTI-VISAQH-HPEIT---------LE----QIQR-DIKEFVINPV-----V-P-T-------D-L-ID--EDT--KYFINPTGRFVIGGPQGDAGLTGRKIIVDTYGGYARHGGGAFSGKDATKVDRSASYAARYVAKNIVAAGLADRCEVQLAYAIGVAQPVSISIDTFGTGT--VEES----KLVELTRGLFDLRPAGIIKMLDLRR-P-------IYKQTAAYGHFGR-T----------------------DID---L--P-WEQT-DK-A-----DALK----E----LAGK----------------

>Firmicutes_Virgibacillus_proomii

----MA------T------------NR-R-LFTSESVTEGHPDKIADQISDAILDEILKSDP-----YARVACETAVTTGLVLVAGEISTTT-YV--------DIPAIVRKTIKEIGYTR--AK-FGFDAETCAVLTAIDEQSPDIAGGV-NT-ALEAR-Q--G-KMS-E--EEID-AI-GAGDQGLMFGFACDETEELMPLPISLAHKLAKRLADVRKDK--M-LAYLRPDGKTQVTIEYG-E-N--D--E-PVRIDTI-VISAQH-HQDIT---------PE----QIEK-DLIEHVIRPV-----V-P-T-------H-L-LD--EKT--KYFINPTGRFVIGGPQGDAGLTGRKIIVDTYGGYARHGGGAFSGKDATKVDRSAAYAARYVAKNIVAADIAKTCEVQLAYAIGVAEPVSISINTFGTGK--VSEE----KLVNAVRKLFDLRPAGIIRMLDLQK-P-------IFKDTAAYGHFGR-T----------------------DHS---F--P-WEKT-DK-V-----DELR----S----LTKK----------------

>Firmicutes_Oceanobacillus_arenosus

----MA------A------------NR-R-LFTSESVTEGHPDKMSDQISDAILDAILEKDP-----YARVACETTVTTGLVLVAGEISTST-YV--------DIPAIVRQTVKEIGYTR--AK-YGFDAETCAVMTAIDEQSADIAGGV-DK-ALEAR-Q--G-KMS-E--AEID-AI-GAGDQGLMFGFACDETEELMPLPISLAHKLAKRLTDVRKDK--T-LDYLRPDGKTQVTIEYD-E-N--D--T-PIRVDTI-VISTQH-HQDIT---------TE----QIEK-DMIEQVIRPI-----V-P-E-------A-L-LD--DET--KYFINPTGRFVIGGPQGDVGLTGRKIIVDTYGGYARHGGGAFSGKDATKVDRSAAYAARYVAKNIVAADLAKSCEVQLAYAIGVAEPVSIAINTFGTGI--VAED----VLVEAVRQLFDLRPAGIINMLDLRK-P-------IFRNTAAYGHFGR-T----------------------DIK---F--P-WEKT-DK-V-----NELK----A----L-------------------

>Firmicutes_Caryophanon_latum

-----M------T------------QR-R-LFTSESVTEGHPDKICDQISDAILDAILAADP-----NARVACETTVTTGLVLVAGEITTST-YV--------DMKGIIRDTVAEIGYTR--GK-YGFDAENLAVLVAVGEQSPDIAQGV-DR-ALEAR-E--G-SMT-D--EELE-AI-GAGDQGLMFGYACNETPELMPMPISLAHKLARRLAEVRKNG--T-LDYLRPDGKTQVTVEYD-E-N--N--T-PVRVDTV-VISTQH-DEEAT---------LE----QIQE-DMKKHVIAAV-----V-P-A-------E-L-LD--EHT--KYFINPTGRFVIGGPKGDAGLTGRKIIVDTYGGYARHGGGAFSGKDATKVDRSAAYAARYVAKNIVAAGLADRAEVQLAYAIGVARPVSIAVDTFGTGK--VDSE----QIVEWIREEFDLRPAGIIKMLDLRR-P-------IYKQTAAYGHFGR-T----------------------DLN---V--P-WEAT-DK-A-----DILR----A----KAGL----------------

>Firmicutes_Bacillus_clausi

----MS---V-KR------------GR-H-LFTSESVTEGHPDKICDQISDAILDEILKKDP-----NARVACETSVTTGLVLVAGEITTNT-YV--------DIPSVVRQTIKGIGYTR--AK-YGFDAETCAVLTSIDEQSADIAQGV-DK-ALEAR-E--G-QMT-D--AEIA--I-GAGDQGLMFGFATNETEELMPLPISLSHKLSRRLTEVRKNG--T-LGYLRPDGKTQVTIEYD-E-N--D--T-PVRVDTI-VLSTQH-APEVT---------LE----QIQA-DLKEHVIEAV-----V-P-S-------A-L-ID--EET--KYFINPTGRFVIGGPQGDAGLTGRKIIVDTYGGYARHGGGAFSGKDATKVDRSGAYAARYVAKNIVAAGLADKCEVQLAYAIGVAQPVSIAVNTFGTGK--AAED----VLVELVRKHFDLRPAGIIRMLDLRR-P-------IYKQTAAYGHFGR-T----------------------DVP---L--P-WEAT-DK-A-----AALK----E----DVLKLEAN------------

>Firmicutes_Amphibacillus_xylanus

---------M-TA------------NR-R-LFTSESVTEGHPDKICDQISDAVLDAILKEDP-----LARVACETTITTGLVLVSGEITTST-YV--------DIPKIVRQTVKEIGYVD--SA-YGFDYETCAVLTAIDEQSRDIADSV-DL-ALEAR-E--G-QMT-D--EEIE-AI-GAGDQGLMFGYATNQTPEYMPLPISLAHKLAKQLTDVRKNG--Q-LDYLRPDGKTQVTVEYD-E-Q--N--Q-PIRIDTI-VISTQH-DADVS---------LE----QIQS-DMKEYVIKAI-----V-P-N-------E-L-LD--DQT--KYIINPSGRFVIGGPQGDVGLTGRKIIVDTYGGYARHGGGAFSGKDATKVDRSASYAARYVAKNIVAAGLADNCEVQLAYAIGVAEPVSISIDTFGTGK--VSEE----KLIEAVRDLFDLRPAGIINMLQLRK-P-------IFKPTATYGHFGR-T----------------------DVE---F--P-WERL-DK-A-----EAIK----A----FVNK----------------

>Firmicutes_Hydrogenibacillus_schlegelii

----MR---H-LK------------GR-H-LFTSESVTEGHPDKMCDQISDAILDAVLAQDP-----NARVAIETTVTTGLVLVVGEMTTSG-YV--------DIPKVVRDTVREIGYTR--AK-FGFDADTCAVLTSINEQSPDIAMGV-DR-ALEAR-E--G-QMS-E--DEIE-AI-GAGDQGLVFGFAVNETPELMPLPIALAHRLARRLAEVRKDK--T-LPYLRPDGKTQVTVEY--D-G--P--K-PVRVDTI-VVSAQH-DPDVD---------QA----TIRR-DILEHVIRPV-----V-P-A-------A-Y-LD--AET--KYFINPTGRFVLGGPQADAGLTGRKIIVDTYGGYARHGGGAFSGKDPTKVDRSAAYAARHVAKNIVAAGLADMCEVQIAYAIGVARPVSIRVDTFGTGK--VPEG----VLIRLIERHFDLRPAGIIRRFDLRR-P-------IYRQTAAYGHFGR-T----------------------DLD---L--P-WERT-DV-A-----EALR----R----DAEAYVSGA-Q--AGNL---

>Firmicutes_Caldicellulosiruptor_danielii

-------------------------MR-K-LFTSESVTEGHPDKICDQISDAVLDAILEKDP-----YARVACEVAVTTGLVLVMGEITTKC-YV--------DIPKIARDTIREIGYTR--AK-YGFDADTCAVITSIDEQSPDIAMGV-DK-ALEAK-L--G-EMT-D--DEIE-AI-GAGDQGMMFGFACDETPVLMPMPIYLAHKLARRLAYVRKEG--I-LPYLRPDGKTQVTVEY--E-D--D--R-PVRVDTI-VVSTQH-SPEVT---------HA----QIEA-DVIEHVIKPI-----I-P-E-------G-M-LD--KNT--KIYINPTGRFVIGGPQGDSGLTGRKIIVDTYGGYARHGGGAFSGKDPTKVDRSATYAARYVAKNIVAAGLAKKCEVQVSYAIGVARPLSIRVDTFGTGK--ISDE----KIAEIVKRVFDLRPAAIIRDLDLRR-P-------IYKQIAAYGHFGR-E----------------------DLD---L--P-WERT-DK-V-----DIIL----K----EAQSI---------------

>Firmicutes_Ruminiclostridium_hungatei

------------M------------TR-R-LFTSESVTEGHPDKMCDQISDAVLDAIFQQDP-----QARVACETAVTTGMVLVMGEISTKC-YV--------DIPKVVRNTIREIGYDR--AK-YGFDSETCAVLTSIDEQSADIAMGV-DK-ALEAK-T--G-EMS-E--AQIQ-AI-GAGDQGMMFGFACDETPELMPMPITLAHKLSKKLSEVRKDG--T-LDYLRPDGKSQVTVEY--D-G--D--K-PVRVDTV-VISTQH-GPDVS---------HE----IIER-DMMEFVIKPV-----I-P-A-------E-L-LD--QNT--RYFINPTGRFVVGGPQGDSGLTGRKIIVDTYGGYARHGGGAFSGKDPTKVDRSAAYAARYVAKNIVAAGIAKKCEVQLAYAIGVAKPVSVLVDTFGTAV--IPEE----KISELVNKHFDLRPAGIIKTLDLRR-P-------IFKKTAAYGHFGR-N----------------------DND---F--T-WEKT-DV-A-----ELLK----K----EA-GL---------------

>Firmicutes_Hungateiclostridium_thermocellum

------------M------------AR-K-LFTSESVTEGHPDKICDQISDAVLDAIFSQDP-----MARVACETAVTTGLVLVTGEITTNC-YV--------DIPKIARSTIREIGYDR--AK-YGFDCDTCAVLTSIDEQSPDIAMGV-NK-ALEAK-T--G-EMS-D--EEIE-AI-GAGDQGMMFGFACDETPELMPMPISLAHKLAMRLSQVRKNG--T-LNYLRPDGKSQVTVEY--D-G--D--K-PVRVDTV-LISTQH-GPEVD---------YD----TIKR-DVIEHVIKPV-----I-P-A-------E-L-LD--SKT--KYLVNPTGRFVIGGPQGDSGLTGRKIIVDTYGGYARHGGGAFSGKDPTKVDRSAAYAARYVAKNIVAAGLARKCEVQLAYAIGVARPVSVRVDTFGTGI--IPEE----KIESLVNKYFDLRPAGIIKTLDLRR-P-------IYKQTAAYGHFGR-T----------------------DID---L--P-WERT-DK-A-----EELR----K----EAQSL---------------

>Firmicutes_Petroclostridium_xylanilyticum

------------M------------SR-K-LFTSESVTEGHPDKICDQISDAVLDAIFEKDP-----MARVACETSVTTGLVLVAGEITTSC-YV--------DIPKIVRETIRGIGYDR--AK-YGFDCDTCAVLTAIDEQSPDIAMGV-DR-ALEAK-K--G-EMS-E--EEIA-AI-GAGDQGMMFGFACDETPELMPMPIALAHRMARKLAEVRKNG--T-LEYLRPDGKTQVTVEY--E-D--D--R-PVRIDTI-VVSTQH-SPEVD---------HD----TIEK-DIMEHVVKPS-----V-P-A-------E-L-LD--AQT--KYFINPTGRFVVGGPQGDAGLTGRKIIVDTYGGYARHGGGAFSGKDPTKVDRSAAYAARYVAKNVVAAGLARKCEVQLAYAIGVAKPVSVLVETFGTAK--IPEE----KIQELIVKHFDLRPAGIIKTLDLRR-P-------IYKQTAAYGHFGR-T----------------------DVD---L--P-WERT-DK-A-----EILK----K----EA-SL---------------

>Firmicutes_Caldicoprobacter_oshimai

------------M------------RR-R-LFTSESVTEGHPDKICDQISDAVLDAILAKDP-----YARVACETAVTTGLVLVMGEITTDC-YV--------DIPSIVRDTIREIGYTR--AK-YGFDADTCAVITSIDEQSPDIAMGV-NK-ALEAK-M--H-EM--E--DDIE-AI-GAGDQGMMFGFACNETPELMPLPISLAHKITRRLAQVRKDG--T-LEYLRPDGKSQVTVEY--E-G--D--K-PVRVHTV-VVSTQH-SPDVD---------HS----TIER-DVIEKVIKAV-----I-P-S-------E-L-LD--DKT--RYLVNPTGRFVVGGPQGDSGLTGRKIIVDTYGGYARHGGGAFSGKDPTKVDRSASYAARYVAKNIVAAGLADKCEVAVAYAIGVARPVSISVDTFGTAK--VPEE----LILELIHKNFDLRLGAIIRDLNLRR-P-------IYKQTAAYGHFGR-T----------------------DLD---L--P-WEKT-DK-A-----QLLR----E----QAFGK---------------

>Firmicutes_Caloramator_australicus

------------M------------VK-R-LFTSESVTEGHPDKVCDQISDAILDAILAQDP-----EARVACETAVTTGMVLVMGEITTKC-YV--------DIPKIVRKTVEEIGYTR--AK-YGFDADTCAVLTSIDEQSPDIAMGV-DK-ALEAK-K--G-EM-----DAIE-AI-GAGDQGMMFGFACNETPEYMPLPIALAHKLARRLAEVRKNG--T-LEYLRPDGKTQVTVEY--H-D--D--K-PVRVDTI-VVSTQH-SPEVT---------RE----QIER-DIIEHVIRKV-----V-P-A-------N-L-LD--ENT--KYLINPTGRFVIGGPHGDSGLTGRKIIVDTYGGYGRHGGGAFSGKDPTKVDRSAAYAARWVAKNLVAAGLADKLEIEIAYAIGVARPVSIEVETFGTGK--LPDD----KIVEIVNRVFDLRPAAIIKNLDLRR-P-------IYRQVAAYGHFGR-T----------------------DLD---L--P-WERL-DK-V-----EEIK----K----IVAEMM--------------

>Firmicutes_Clostridium_acetobutylicum

-------------------------MR-K-LFTSESVTEGHPDKICDQISDAILDAILEKDP-----NGRVACETTVTTGIVNVMGEISTNC-YV--------DIPKIVRKTVREIGYTR--AK-YGFDCDTCAVVTSIDEQSADIAMGV-DE-ALESK-K--G-EM-----DKID-AV-GAGDQGMMFGYATNETKEFMPMPIALAHRLSRRLAEVRKDG--T-LDYLRPDGKTQVTIEY--E-D--D--K-PVRVDAI-VISTQH-GPEIG---------HE----QIEK-DLIEKVVKYV-----I-S-P-------E-L-LD--ENT--KYYINPTGRFVVGGPQGDSGLTGRKIIVDTYGGYGRHGGGAFSGKDPTKVDRSAAYAARWVAKNLVAAGIADKLEIQLAYAIGVAKPVSISVDTFGTGK--IEES----KIVEIVEKVFDLRPGAIIRDLNLKR-P-------IYRQVAAYGHFGR-L----------------------DVE---L--P-WEQL-DR-V-----EAIK----K----YL------------------

>Firmicutes_Paramaledivibacter_caminithermalis

-----------ME------------LR-R-LFTSESVTEGHPDKICDQVSDAILDAIFVNDP-----NARVACETTVTTGLVLVTGEISTKC-YV--------DIPKIVRKTIEEIGYTR--AK-YGFDCDTCAVLTSIDEQSPDIAMGV-DE-ALERK-K--G-DM--G--DEIE-AI-GAGDQGIMFGFACNETEELMPLPISLAHKLARRLSEVRKNG--T-LEYLRPDGKTQVTVEY--E-G--D--K-PVRIDTI-VISTQH-GPEVS---------RD----QIEK-DMIEHVVNKI-----V-P-P-------D-L-LD--ENT--RYLINPTGRFVIGGPQGDAGLTGRKIIVDTYGGYSRHGGGAFSGKDPTKVDRSAAYAARYVAKNIVAAGLAEKCEIELAYAIGVAQPVSILVETFGTGK--VSEE----KLVQLVKKHFDLRPAAIIRDLDLRR-P-------IFRQTAAYGHFGR-T----------------------DID---L--S-WERT-DK-A-----EILR----K----E-GFGE--------------

>Firmicutes_Alkaliphilus_oremlandii

------------M------------FK-K-LFTSESVTEGHPDKMCDQISDAILDAILEKDP-----AARVACETSVSTGLVLVAGEITTKC-YV--------DIPKIVRKTIEEIGYTR--AK-YGFDSDTCAVLTAINEQSADIALGV-DE-ALESK-K--G-EH--R--DELE-AI-GAGDQGIMFGFACNETPELMPLPISLAHKLAKRLSDVRKDG--T-IGYLRPDGKTQVTVEY--D-G--D--K-PVRVDTI-VISTQH-GPEVD---------AA----TIEK-DMIEHVVQKI-----V-P-A-------E-L-LD--ENT--RYFINPTGRFVIGGPQGDAGLTGRKIIVDTYGGYARHGGGAFSGKDATKVDRSAAYAARYVAKNIVAAGLADKCEIELAYAIGVSRPISILVETFGTGK--IAEA----DIAELIGKHFDLRPAAIIRDLGLRN-P-------GYRNVAAYGHFGR-A----------------------DLD---L--T-WERT-DK-A-----ELLR----K----EAGL----------------

>Firmicutes_Garciella_nitratireducens

------------M------------VK-K-LFTSESVTEGHPDKICDQISDAILDAILAEDP-----NARVACETTVTTGLVMVMGEISTNT-YV--------DIPKVVRNTVKEIGYTR--AK-FGFDGDTCSVITSIDEQSPDIAMGV-NQ-ALEAR-E--G-SMT-D--EEIE-AI-GAGDQGMMFGFATNETPELMPLPISLAHKLARRLAQVRKEG--I-VEYLRPDGKTQVTIEY--E-E--D--K-PIRVEAI-VVSTQH-NSEVD---------RK----TIEK-DILKQVIQPV-----V-P-A-------E-L-LD--ENT--KYFINPTGRFVIGGPQGDSGLTGRKIIVDTYGGYGRHGGGAFSGKDPTKVDRSASYAARYVAKNIVAAGLADKCEVELAYAIGVAKPVSIFIETFGTGK--IEDE----KIVQLVRKHFDLRPAGIIKELNLRR-P-------IYRQTAAYGHFGR-T----------------------DID---L--P-WERT-DK-A-----EILR----K----EVLEN---------------

>Firmicutes_Anoxybacter_fermentans

-----------MT------------KR-Y-LFTSESVTEGHPDKMADQISDAVLDAIFANDP-----NARVACECFLTTGLVLVGGEITTKC-YV--------DIPKLVRDTIRDIGYTR--AK-YGFDADTCAVITSIDEQSPDIAMGV-DK-ALEAK-K--G-EM-----EAEE-AI-GAGDQGLMFGFATNETEEYMPTPIMLAHKLARRLAEVRKNK--T-LPYLRPDGKTQVTIEY--E-D--D--K-PIRVDTI-VVSAQH-NPEVT---------LE----QIEK-DLIEHVIKPI-----V-P-E-------N-L-LD--NKT--RYLINPTGRFVIGGPQGDAGLTGRKIIVDTYGGYARHGGGAFSGKDPTKVDRSAAYAARYVAKNVVAAGLADKCEVQLAYAIGVAEPVSIMVDTFGTNR--IDEK----KIVELIKENFDLRPACIIRDLDLRR-P-------IYRQVAAYGHFGR-N----------------------DLD---L--P-WERL-DK-V-----EVLK----K----ALK-----------------

>Firmicutes_Desulfosporosinus_acidiphilus

------------M------------AK-R-LFTSESVTEGHPDKICDQISDAILDAIFTQDP-----NARVACEAMVTTGLVLVSGEITTSC-YV--------DIPHVVRETVREVGYTR--AK-YGFDADTCAVLTSIGEQSADIALGV-NQ-ALESK-T--G-EMS-D--LDIE-AI-GAGDQGMMFGYATNETESYMPVPIDLAHRLARRLTEVRKSE--F-LSYLRPDGKTQVTVEY--D-G--D--K-PVRVDTI-VISTQH-HPDVT---------QE----QIRR-DLLQHVIYPT-----V-P-K-------E-L-LN--EDT--KYFINPTGRFVIGGPQGDCGLTGRKIIVDTYGGMARHGGGAFSGKDPTKVDRSAAYAARYVAKNVVAAGFADRCEIQIAYAIGVARPVSVSVETFGTGK--LPDE----KIVELVMKTFDLRPAGIIKNLDLRR-P-------IYRQTAAYGHFGR-T----------------------DLD---L--P-WERT-DK-V-----EALK----K----LAGL----------------

>Firmicutes_Heliobacterium_modesticaldum

-----------MS------------RK-R-LFTSESVTEGHPDKVADQISDSVLDAILAQDP-----MARVACETSVTTGLVLVAGEITTKC-YV--------DIPKVVRQTIREIGYTR--AK-FGFDCETCAVLTSIDEQSPDIAMGV-DK-ALEAR-T--G-EMS-E--SEIE-AT-GAGDQGMMFGYATNETEEYMPMPISLAHKLARRLSEVRKTA--E-LEYLRPDGKTQVTVEY--D-G--D--K-PVRVDTV-VISAQH-SPEVS---------LD----TIKQ-DLIERVILPI-----I-P-P-------G-L-LD--DKT--RYFINPTGRFVIGGPQGDAGLTGRKIIVDTYGGMARHGGGAFSGKDPTKVDRSAAYAARHVAKNVVAAGLADRCEIQLAYAIGVAHPVSVLVETFGTAK--VDEA----SIEKWVKEVFDLRPAGIIKELQLRR-P-------IYRQTAAYGHFGR-V----------------------DLD---L--P-WERL-DK-V-----EALK----K----LAGL----------------

>Firmicutes_Anaerobranca_californiensis

-----------MS------------RK-F-LFTSESVTEGHPDKIADQISDAILDAILEKDP-----NARVAAETMVSTGLVLVSGEITTEC-YV--------DIPKIVRETVKEIGYTR--AK-FGFDSETCAVLTAIDEQSPDIAMGV-DK-ALEAK-E--G-EM-----EDID-AV-GAGDQGMMFGFACNETPELMPLPISLAHKMSRRLSEVRKSE--V-LPYLRPDGKTQVTVEY--V-D--G--K-PTRVTTV-VVSTQH-SPEIS---------LE----TLKK-DIIEHVVKPV-----I-P-T-------E-F-LD--ENT--KFLINPTGRFVIGGPQGDAGLTGRKIIVDTYGGYARHGGGAFSGKDPTKVDRSAAYAARYVAKNVVAAGLAEKCEVQLAYAIGVAKPVSIMVETFGTEK--VPVE----KIEKAIKETFDLRPGAIIRDLGLRR-P-------IYKQVAAYGHFGR-T----------------------DIQ---L--P-WEQT-DK-A-----EILA----Q----KVK-----------------

>Firmicutes_Desulfurispora_thermophila

------------M------------AK-R-LFTSESVTEGHPDKVADQISDAILDAILAQDP-----MARVACETLVTTGLICVAGEISTRC-YV--------DIPKIARETVREIGYTR--AK-YGFDCDTCAVITSIDEQSPDIAMGV-DK-ALEAR-E--G-QMT-D--SEIE-AI-GAGDQGMMFGYATDETPEMMPLPIALAHRLARQLALMRKTH--E-VPYLRPDGKVQVTVQF--E-D--D--R-PVRLDTV-VISTQH-HPKVG---------LE----TIRE-DMIEKVIKPI-----V-P-A-------E-L-LD--KNT--RYFVNPTGRFVIGGPQGDTGLTGRKIIVDTYGGMARHGGGAFSGKDPTKVDRSASYAARYVAKNIVAAGLARRCEVQLAYAIGVARPVSIMVETFGTGL--VAEE----VLVRLVQEYFDLRPAGIIQALDLRR-P-------IYRQVAAYGHFGR-N----------------------DLD---L--P-WERT-DK-A-----EYLR----R----EAGI----------------

>Firmicutes_Pelotomaculum_propionicicum

------------M------------SR-R-LFTSESVTEGHPDKVADQISDAVLDSIFESDP-----MARIACETLVTTGLVLVAGEITTNC-YI--------DIPRVARDTIREIGYTR--AK-YGFDCDTCAVITSIDEQSCDIARGV-NR-ALETR-E--C-EEG-V--DEFD-LT-GAGDQGMMFGYATDETPELMPLPIALAHRLARRLAEVRKGR--I-VPYLRPDGKTQVTVEY--E-N--G--R-PLRVDNI-VVSTQH-HPRVS---------LE----EIRR-DVLKEVIKPI-----V-P-D-------E-F-LD--AGT--RFFVNPTGRFVVGGPQGDTGLTGRKIIVDTYGGMARHGGGAFSGKDPTKVDRSASYAARYVAKNIVAAGLADKCEFQVAYAIGVAHPVSVMVETFGTGK--IDEQ----LLVNLVRQHFDLRPAAIIKELDLRR-P-------IYKKVAAYGHFGR-N----------------------DLD---L--P-WERT-DK-A-----EALR----R----SAGL----------------

>Firmicutes_Sporolactobacillus_vineae

----MS---K-KF------------SR-R-LFTSESVTEGHPDKICDQISDAILDELLKGDS-----NARVACETAVNTGLVLVTGEISTST-YV--------DIPKIVRRTIREIGYTD--AK-YGFDASNCAILTSIEEQSPDIAQGV-DQ-ALEAR-E--G-KMS-D--DEIE-AI-GAGDQGMMFGFAVNETPELMPLPISLAHKLAKKLADVRKSQ--E-LTYLRPDGKTQVTVEYD-E-E--G--H-PARIDTI-VVSTQH-DPDVS---------LA----QITR-DIREHVIAPS-----V-P-E-------G-L-ID--DET--KFFINPTGRFVIGGPQGDAGLTGRKIIVDTYGGYARHGGGAFSGKDATKVDRSASYAARYVAKNIVAAGLADKCEVQFAYAIGVARPVSISVDTFGTGK--ASEE----QLVQLIRHNFDLRPAGIIKMLDLRR-P-------IYRQTAAYGHFGR-T----------------------DID---L--P-WEKT-DK-A-----ELLK----Q----QIS----NG-SFAATHLD--

>Firmicutes_Fervidicola_ferrireducens

-----------MA------------RK-Y-LFTSESVTEGHPDKICDQISDAILDAILEKDP-----MARVACETAVTTGLVLVMGEITCDC-YV--------DIPSIARETIREIGYTR--AK-YGFDCDTCAVITSIDEQSPDIAIGV-DS-AYEVR-E----EGQ-G--DELD-RI-GAGDQGMMFGFAVDETPELMPMPIMLAHKLARRLAEVRKTK--I-LPYLRPDGKTQVTVEY--E-D--G--R-PVRVDKV-VVSAQH-KSTVD---------LA----TLRS-DIVETVIKKV-----I-P-E-------E-L-MD--KNT--KFYINPTGRFVIGGPHGDSGLTGRKIIVDTYGGYARHGGGAFSGKDPTKVDRSASYAARYVAKNIVAAGLAKKCEVQVAYAIGVAHPVSIMVDTFGTGV--LPDE----KIEKLIVENFDLRPAAIIRDLDLRR-P-------IYRQVAAYGHFGR-T----------------------DLD---V--P-WEKT-DK-A-----EILK----K----QASSL---------------

>Firmicutes_Moorella_humiferrea

------------M------------TR-K-LFTSESVTEGHPDKIADRIADAVLDAIIEKDP-----QARVACECLVSTGLVLVAGQITTSC-YV--------DIPRVARETIREVGYTR--AK-FGFDCDTCAVITSIDEQSPDIAMGV-NE-AWEKK-E--G--LA-A--DEVE-TL-GAGDQGMMFGYATRETPEYMPMPIALAHRLTRRLAEVRKER--I-LPYLRPDGKSQVTVEY--E-D--G--R-PVRVDTV-VISTQH-HPDIN---------MA----TLRN-EVLETVIKPV-----I-P-A-------E-M-LD--NRT--RYFINPTGRFVIGGPQGDTGLTGRKIIVDTYGGMARHGGGALSGKDPTKVDRSAAYAARYVAKNVVAAGLADRCEVQVAYAIGVARPVSISVETYGTGK--ISDE----RLVELIKAHFDLRPGAIIRDLDLRR-P-------IYKQVAVYGHFGR-T----------------------DLD---L--P-WERL-DK-V-----EALQ----E----AAGI----------------

>Firmicutes_Orenia_metallireducens

-----------MG------------KR-Y-LFTSESVTEGHPDKVADQISDSVLDAILSQDP-----QARVACETTVTTGMILVSGEISTNC-YV--------DIPKIARNVVREIGYTR--AK-YGFDSETCAVLTSIDEQSPDIAQGV-DE-ALEVK-E--G-KE--S--AE----I-GAGDQGLMFGYASNETEELMPLPITLAHKLARRLAKVRKEE--I-LDYLRPDGKTQVTIEY--E-D--D--K-PVRIDTI-VISTQH-HPEIS---------LE----QIRE-DMIEYVVKPV-----I-D-S-------D-L-LD--EKT--RYFINPTGRFVIGGPQGDVGLTGRKIIVDTYGGMARHGGGAFSGKDATKVDRSAAYAARYVAKNIVAAGLADKCEVQLSYAIGVAYPVSIMVDTFGTAK--IEES----KIVELVNKHFDLRPGKIIETLNLRK-P-------HYRQVAAYGHFGR-N----------------------DLD---L--P-WERT-DK-V-----DLLK----A----DAGL----------------

>Firmicutes_Acetohalobium_arabaticum

---------M-AN------------KN-Y-LFTSESVTEGHPDKVADQISDAVLDAILSQDP-----QGRVACETLVTTGMVLVSGEISTDC-YV--------DISQIARETVKEIGYTR--AK-FGFDGETCAVLTSIDEQSPDIAMGV-DE-ALEAK-E--G-STG-D--QE----L-GAGDQGLMFGYATNETEELMPLPIVLSHKLARRLAKVRKDN--L-LSYLRPDGKTQVTVEY--E-E--D--K-PVRVDTV-VVSTQH-GPDVS---------LD----QIKE-DIISEVIKPV-----I-D-E-------E-M-LD--DKT--EYLVNPTGRFVIGGPHGDAGLTGRKIIVDTYGGTARHGGGAFSGKDPTKVDRSATYAARYVAKNVVAAGLADRCEVQLSYAIGVASPVSIMVDTFGTAN--IEEE----ALEELITEYFDLRPSRIIEELNLRR-P-------IYNQVAAYGHLGR-N----------------------DLE---L--P-WERT-DK-A-----DLLR----D----EAGL----------------

>Firmicutes_Desulfitibacter_alkalitolerans

------------M------------VK-K-LFTSESVTEGHPDKIADQVSDSILDAIISEDE-----NARVACETTVTTGLVLVAGEITTEC-YV--------DIPKIVRETIRDIGYTR--AK-FGFDCDTCAVLTAIDEQSPDIALGV-NK-ALEAK-K--G-EMS-E--VEIQ-AI-GAGDQGMMFGYACDETPELMPLPISLAHKLTKRLSEVRKQR--I-LPYLRPDGKSQVTVEY--E-D--D--K-PIRVDTV-VISTQH-RPDIE---------QE----IIHE-DVLRCVVKEV-----I-P-A-------E-L-LD--DKT--RYFINPTGRFVVGGPQGDAGLTGRKIIVDTYGGMARHGGGAFSGKDPTKVDRSAAYAARYVAKNIVAAGLANRCEIQLAYAIGVAQPVSIMIETFGTGK--IADE----KIAQLVKENFDLRPAAIIRDLKLRK-P-------IYRNLAAYGHMGR-V----------------------DLN---V--K-WEDT-DK-A-----EALK----Q----QAGILK--------------

>Firmicutes_Alicyclobacillus_montanus

-----------MT------------IR-R-LFTSESVTEGHPDKICDQISDAVLDEILTHDP-----HARVACETSVTTGLVLVAGEITTSC-YV--------DIPKIVRRTLADIGYTR--AK-FGFDAETCAVITSIDEQSPDIAQGV-NV-ALEAR-N-----LT-D--EQVE-EI-GAGDQGLMFGFACDETPELMPLPISLAHKLSRRLSEVRKNG--T-LPYLRPDGKTQVTIEY--D-G--D--K-PVRVDTI-VISTQH-DDNTT---------LE----TISE-DMHTHVISAV-----V-P-K-------E-Y-LD--AET--KYFINPTGRFVIGGPQGDAGLTGRKIIVDTYGGYARHGGGAFSGKDPTKVDRSAAYAARYVAKNLVASGLARKCEVQVAYAIGVARPVSIMVDTFGTGK--LSED----ALVELVKRNFDLRPAAIIRDLDLRR-P-------IFRQTAAYGHFGR-S----------------------DLQ---L--P-WERT-DK-A-----SLLA----E----QARAISQ-------------

>Firmicutes_Paeniclostridium_sordellii

------------M------------AR-H-LFTSESVTEGHPDKICDQISDAILDALLEKDP-----LARVACETTVTTGLVLVAGEISTNT-YV--------DIPKLVRETVKGIGYTR--AK-FGFDGDTCAVITSIDEQSGDIAMGV-DE-ALENR-S--G-ELS-E--DEIE-KI-GAGDQGIMFGFACNETEELMPLPISLAHKLARRLTEVRKNG--E-LSYLRPDGKTQVTVEY--D-K--D--K-AIRVHTV-LISTQH-GEDVD---------ND----TIRR-DLIEKVIKEV-----I-P-S-------E-L-LD--EET--KIYINPTGRFVIGGPQGDTGLTGRKIIIDTYGGYSRHGGGAFSGKDPTKVDRSAAYAARYVAKNIVAAGLADKCEIELAYAIGVARPLSIFIDTFGTGK--VSEE----ILVDLVNKNFDLRPGAIIRDLDLRK-P-------MYKNVAAYGHFGR-C----------------------DLD---L--P-WERT-DK-A-----KTLR----E----QAEL----------------

>Firmicutes_Terrisporobacter_glycolicus

------------M------------SR-H-LFTSESVTEGHPDKMCDQISDAILDALLEKDP-----LSRVACETITTTGLVLVAGEISTNA-YV--------DIQKVVRDTVKEIGYTR--AK-YGFDSQTCAVITAIDEQSSDIAMGV-DE-ALESR-S--G-EQT-E--EEIE-AI-GAGDQGIMFGFACNETPELMPLPISLAHKLSRRLTEIRKNK--T-VDYLRPDGKTQVTVEY--D-G--N--K-PVRVHTI-LISTQH-SEHVD---------ND----TIRK-DLIELVIKEV-----I-P-A-------D-L-LD--DET--KIYINPTGRFVIGGPQGDTGLTGRKIIIDTYGGYSRHGGGAFSGKDATKVDRSAAYAARYVAKNIVAAGLADKCEIELAYAIGVARPLSIFVDTFGTGK--VSEA----ELVELINKNFDLRPGAIIRDLDLRK-P-------IYRQTAAYGHFGR-T----------------------DVD---L--P-WERT-DK-A-----EALR----E----QSSIK---------------

>Firmicutes_Peptostreptococcus_stomatis

------------M------------AR-H-LFTSESVTEGHPDKLCDQVSDAILDALIEKDP-----KSRVACETTATTGLILVAGEISTNA-YV--------DIPKIVRETVEGIGYVR--GK-FGFDAENCAVITAIDEQSNDIAMGV-DE-GLESK-E--G-EKV-E--DEIE-KV-GAGDQGIMFGFACNETPELMPLPISLAHKLSRKLTDVRKSG--Q-IAYLRPDGKTQVTVEY--E-E--D--K-PVRVHTV-LISAQH-DPDTT---------ND----QIRK-DLIEKVIKTT-----I-P-A-------E-L-LD--DET--LYYINPTGRFVVGGPHGDTGLTGRKIIIDTYGGYSRHGGGAFSGKDPTKVDRSACYAARYVAKNIVAAGLADKCEIELAYAIGVARPLSIFVDTFGTGK--LSNE----KLVEIVKENFDLRPGAIIKNLDLLR-P-------IYKQVAAYGHFGR-T----------------------DID---L--P-WERT-DK-V-----ETLK----A----YL------------------

>Firmicutes_Ammonifex_thiophilus

------------M------------AK-R-LFTSESVTEGHPDKMADQISDAILDAILAQDP-----EARVACETLVTTGLAFVAGEITTRC-YV--------DIPAVVRETIREIGYTR--AK-FGFDYETCAVITSIQEQSPDIAMGV-SQ-ALEYR-T--G--VE-K--DPYS-AI-GAGDQGIMFGYATNETPELMPLPIMLAHKLARQLAMVRKSG--E-LPYLRPDGKTQVTVEY--E-G--D--R-PVRVHTV-LVSAQH-HPDID---------LP----TLRE-DLLMKVIRPV-----I-P-P-------E-L-MD--GET--RFLVNPTGRFVIGGPQADTGLTGRKIIVDTYGGMARHGGGCFSGKDPTKVDRSGSYAARYVAKNIVAAGLADRCEVQVAYAIGVARPVSISVDTFGTGK--VPEE----RLVELIYKHFDLRPAAIIAHLDLRR-P-------IYRQTAAYGHFGR-T----------------------DID---L--P-WERT-DK-A-----EDLR----R----DA------------------

>Firmicutes_Syntrophomonas_wolfei

-----M---I-SL------------AR-T-LFTSESVTEGHPDKVADQISDAILDAIMELDP-----YGRVAAETIVTTGLVLVAGEITTNC-YV--------DIPRLVRSTIEDIGYTR--AK-YGFDAETCAVLTSLDEQSGDIALGV-DR-AFEAK-K--G-EMD-D--KELD-AI-GAGDQGMMFGYASNETAVLMPMPIYLASKMAERLGEVRKEG--I-IPYLRPDGKTQVTVEY--E-D--Y--K-PLRVTTV-VVSAQH-HPEVS---------SA----TIEK-DIIEHVIKAV-----I-P-P-------E-M-ID--AHT--IFYVNPTGRFVIGGPQGDSGLTGRKIIVDTYGGMARHGGGAFSGKDPTKVDRSASYMLRYVAKNIVAAGLAERCEIQVAYAIGVANPLSINVNTFGTGK--ISDD----KIIELIKKNFDLRPAAIIRDLDLRR-P-------IYRKTAAYGHFGR-E----------------------DAE---F--T-WERT-DK-A-----DTLR----E----QAGL----------------

>Firmicutes_Syntrophomonas_zehnderi

-----------MT------------GK-R-LFTSESVTEGHPDKMADQISDAILDAILEQDP-----FARVAAETIVTTGLVLVSGEITTEC-YV--------DIPRVVRSTISEIGYTR--AK-YGFDSETCAVMTSLDEQSGDIAMGV-NK-ALEAK-K--G-TMN-D--ADFE--I-GAGDQGMMFGYASNETEELMPMPILLASKMAKRMTDVRKAG--I-IPYLRPDGKTQVTVEY--E-D--G--R-PVRIDTV-VVSTQH-HPEVS---------VQ----TIEQ-DIIEHVIKDV-----V-P-A-------D-M-LD--SET--KYYINPTGRFVIGGPQGDCGLTGRKIIVDTYGGMARHGGGAFSGKDATKVDRSATYAARYVAKNMVAAGVADRLEIQLAYAIGVAQPVSISVDTFGTSK--IPED----KIVELIKQHFDLRPAAIIKDLDLRR-P-------IYKKTAAYGHFGR-N----------------------EPE---F--T-WEKT-DK-A-----SAIR----K----SAGL----------------

>Firmicutes_Syntrophothermus_lipocalidus

-------------------------MK-R-LFTSESVTEGHPDKIADQISDAVLDAILTEDP-----MGRVACETLVTTGLVMVAGEITTSC-YV--------DIPRLVRETIREIGYTR--AK-FGFDCDTCAVITSIDEQSPDIAMGV-DR-ALEAK-M--G-EIT-E--EEL--AT-GAGDQGMMFGYATNETEELMPLPIMLAHKLAMRLAEVRKSG--I-IPYLRPDGKTQVTVEY--D-G--D--T-PVRVETV-VVSTQH-HPDIS---------LA----TIRE-DIIENVIETV-----I-P-P-------H-M-LD--RNT--RYLINPTGRFVIGGPQGDTGLTGRKIIADTYGGMARHGGGAFSGKDPTKVDRSATYAARYVAKNLVAAGIADRCELQLAYAIGVARPISVSVDTFSTGK--IAES----KIVDIINEVFDLRPAAIIRDLNLRR-P-------IYKKTAAYGHFGR-N----------------------DED---F--T-WEKT-DK-A-----EELR----R----LAGL----------------

>Firmicutes_Thermohydrogenium_kirishiense

-------------------------MK-K-YFTSESVTEGHPDKICDQISDAIVDEILKNDP-----YARIACETAVTTGMVLVMGEISTKC-YV--------DIPKIARKVIEEIGYTR--AK-YGFDADTCSVLTSIDEQSPDIALGV-DK-ALEVK-K--G--IS-H--NDLE-EQ-GAGDQGMMFGYACTETPELMPLPISLAHRLARRLAEVRKNG--I-LDYLRPDGKTQVTIEY--I-D--D--V-PKRVDSI-VVSTQH-SPDVD---------HD----KIEN-DVKEYVIKPI-----V-P-P-------E-L-ID--EDT--KIYVNPTGRFVVGGPQGDSGLTGRKIIVDTYGGYARHGGGAFSGKDPSKVDRSAAYAARYVAKNIVAAGLADRCEVQLAYAIGVATPLEVNIDTFGTGK--IPDD----EISDIVKKVFDLRPAAIIRDLDLRR-P-------IYRQVAAYGHFGR-T----------------------DLD---L--T-WERT-DR-V-----EILK----K----LAFNK---------------

>Firmicutes_Thermoanaerobacterium_aotearoense

-------------------------MK-K-YFTSESVTEGHPDKICDQISDAIVDEILKNDP-----YARIACETAVTTGMVLVMGEISTKC-YV--------DIPKIARKVIEEIGYTR--AK-YGFDADTCSVLTSIDEQSPDIALGV-DK-ALEVK-K--G--IS-H--DELE-EQ-GAGDQGMMFGYACTETPELMPLPISLAHRLARRLAEVRKNG--T-LDYLRPDGKTQVTIEY--V-D--D--V-PKRVDSI-VVSTQH-SPDVD---------HD----KIEN-DVKEYVIKPI-----V-P-P-------E-L-ID--DDT--KIYVNPTGRFVVGGPQGDSGLTGRKIIVDTYGGYARHGGGAFSGKDPSKVDRSAAYAARYVAKNIVAAGLAEKCEVQLAYAIGVATPLEVNVDTFGTGK--IPDE----EISEIVKKVFDLRPAAIIRDLDLRR-P-------IYRQVAAYGHFGR-T----------------------DLD---L--T-WERT-DR-A-----EILK----K----LAFNKQ--------------

>Firmicutes_Caldanaerobius_fijiensis

------------M------------AK-S-FFTSEAVTEGHPDKICDQISDAILDAIISKDP-----NAKVACETVVTTGMVLVMGEITTSC-YV--------DIPKIVREVVADIGYTR--AK-FGFDADTCAVITSIKEQSPDIAMGV-NK-SLELK-K--G-IDH-D--EDLS-LI-GAGDQGMMFGYACNETPELMPMPIMLAQKLAKRLAYVRKSG--E-LDYLRPDGKTQVTVEY--N-D--G--K-PTRVDAV-VVSTQH-APEVD---------HD----TIER-DIIEHVIKAV-----I-P-N-------E-L-LD--ENT--RYFINPTGRFVIGGPYGDSGLTGRKIIVDTYGGFARHGGGAFSGKDPTKVDRSGAFAARYVAKNIVAAGLADKCEVELAYAIGVAHPISIAVDTFGTGK--VSDE----KLIELIKENFDLRPAAIIKAFDLRR-P-------IYRQISVYGPFGR-T----------------------DLD---L--P-WEKT-DK-V-----DILR----K----QVF-----------------

>Firmicutes_Niameybacter_massiliensis

----MA--------------------R-R-LFTSESVTEGHPDKIADQISDSVLDAIYAQDP-----QARVACETAVTTGLVLVMGEVTTNC-YV--------DINKIARDTINEIGYNN--PA-FGFDGNSCGVMVALDEQSPDIAMGV-DT-ALEKR-N--N-EMS-D--ESIE-AT-GAGDQGMMFGFACDETPELMPMPISLAHKLTRRLTEVRKNG--T-LSYLRPDGKSQVTVEY--D-G--D--K-VVRVDAV-VISTQH-AEEVS---------QA----QIHE-DLMEHVIKHV-----I-P-A-------E-L-LD--DET--KYFINPTGRFVIGGPVGDSGLTGRKIIVDTYGGYASHGGGAFSGKDPTKVDRSAAYAARYVAKNIVAAGLANRCEIELAYAIGVAQPVSILINTHGTGV--VSDE----RLVEIVRETFDLRPAGIIKMLDLRR-P-------IYKQTAAYGHFGR-T----------------------DVS---L--P-WEQT-DK-V-----EALK----K----LAAH----------------

>Firmicutes_Cellulosilyticum_lentocellum

----MA--------------------K-R-LFTSESVTEGHPDKIADQISDAVLDAIFAQDP-----NARVACETALTTGLVLVMGEITTNC-YV--------DINKTVRDTINEIGYNA--PE-YGFDGHSCGVIVALDEQSADIAMGV-DK-ALEAK-K--G-EMT-D--DAIE-AI-GAGDQGMMFGFACDETPELMPMPISLAHRLSKRLSEVRKDG--T-LNYLRPDGKTQVTVEY--D-G--D--T-PIRVDAI-VVSTQH-AAEVT---------QE----QIHA-DIMEYVIKPI-----V-P-E-------N-L-LD--ADT--KYFINPTGRFVIGGPVGDSGLTGRKIIVDTYGGYASHGGGAFSGKDPTKVDRSAAYAARYVAKNIVAAGLAKKCEIELAYAIGVAQPVSVLVNTHGTGV--VSDE----ALVELVRNNFDLRPAGIIKMLDLRR-P-------IYKQTAAYGHFGR-I----------------------DVD---L--P-WERT-DK-A-----EILK----A----AAEK----------------

>Firmicutes_Desulfuribacillus_stibiiarsenatis

----MA---E-EK------------RR-Y-LFTSESVTEGHPDKICDQISDSVLDAIFAEDP-----NARVACETSATTGLILVAGEITTAC-YV--------DIPKIVRKTINEIGYNS---D-FYFDGNTCAVLTAIDEQSPDIAMGV-DR-ALEAK-E--G-LMS-D--EEIE-AI-GAGDQGLVFGFAVNETEVLMPLPIYLAHNLSRRLADVRKNG--T-LNYLGPDGKTQVTVEY--D-G--D--K-PVRIDAI-VVSTQH-KAEIT---------LE----QIKE-DIRKHVIDPE-----V-P-A-------N-L-ID--ENT--KFFINPTGRFVIGGPVGDAGLTGRKIIVDTYGGYARHGGGAFSGKDPTKVDRSGAYAARYVAKNIVAAGLASKCEVQIAYAIGVARPVSVMVDTFGTGK--VSET----LLTDLVNKHFDLRPAGIIKMLDLRR-P-------IYKKTAAYGHFGR-N----------------------DLD---L--P-WEKT-DK-A-----ELLK----Q----EAGL----------------

>Firmicutes_Paenibacillus_yonginensis

-----M---S-IQ------------GR-H-LFTSESVTEGHPDKICDQISDAVLDAFLANDP-----NARVACEVSVATGLVLVIGEISTKSEYV--------DIPSIVRNTIKEIGYTR--AK-YGFDSNTCAVLTSLNEQSADIAQGV-NA-ALEDR-D--P-AKV-A--EETA-NI-GAGDQGLMFGFATNETPELMPLPIALSHRIARRLSEVRKDG--T-LEYLRPDGKTQVTIEY--V-D--G--K-PKRVDTI-VVSTQH-SEDIT---------LE----QIQK-DIKEHVILPV-----V-P-V-------E-L-LD--GET--KYFINPTGRFVIGGPQGDAGLTGRKIIVDTYGGYARHGGGAFSGKDPTKVDRSAAYAARYVAKNLVAAGLADKVEIQLAYAIGVANPVSINVDTYGTGK--VPEE----KLVELVRKNFDLRPAGIISMLDLRR-P-------IYRQTAAYGHFGR-T----------------------DLD---L--P-WERV-DK-A-----ELLK----E----QAGL----------------

>Firmicutes_Selenomonas_sputigena

-----------MS------------KT-M-LFTSESVTEGHPDKMADQISDAILDAILAKDP-----QGRVACETLVTTGQVHVVGEISTSC-YI--------DIPHIIRKTVEKIGYTR--AK-YGFDAATCGILVSLDEQSADIALGV-DK-ALEAK-E--G-ES-----DVAD-SI-GAGDQGMMFGYAANETPELMPLPIALAHRLARRLAEVRKNG--E-LGYLRPDGKTQVTVRY--E-D--G--K-PVAVDTV-VISTQH-DPEVD---------LA----TIRK-DMIEKVIKVI-----I-P-A-------E-L-LT--ADT--KYFVNPTGKFVIGGPQGDSGLTGRKIIVDTYGGMARHGGGAFSGKDPTKVDRSAAYAARYVAKNVVAAGLADRCEIQLAYAIGVARPVSIMVDTFGTNK--VDEA----LIERLVHENFDLRPASIISNLDLRR-P-------IYEQTAAYGHFGR-T----------------------DAD---L--P-WEKT-DK-A-----DALK----K----AAGI----------------

>Firmicutes_Megamonas_funiformis

-----------MS------------NR-V-LFTSESVTEGHPDKVADQISDSILDAILAKDP-----NGRVACETFAMTGQIHVAGEISTDC-YV--------DIPSIVRETIRNIGYTR--AK-FGLDCDTCGISLSIDEQSPDIAMGV-DK-SLEAK-E--G-EA-----DKTD--L-GAGDQGMMFGYATNETPEYMPLPIALAHKLARRLTEVRKSG--E-LSYLRPDGKTQVTVVY--E-D--G--K-PVAVDTI-VISTQH-DPDVT---------LE----QIKQ-DLMEKVIKPV-----V-P-A-------E-L-LT--AET--KYFINPTGKFVIGGPQGDTGLTGRKIIVDTYGGMARHGGGAFSGKDPTKVDRSAAYAARYVAKNIVAAGLADKCEIQLAYAIGVAKPVSISINTFGTNK--VTED----VITKLVNDNFDLRPAGIIKELDLRR-P-------IYAQTAAYGHFGR-T----------------------DVD---L--P-WEHT-DK-A-----ETLR----K----QAGL----------------

>Firmicutes_Pectinatus_cerevisiiphilus

---------M-AQ------------KR-I-LFTSESVTEGHPDKIADQISDSILDAILEQDP-----QGRVACETLITTGQVHIAGEISTKC-YV--------DIPKIVREKIKQIGYTR--AK-FGFDAQTCGISVSIDEQSADIALGV-DK-ALEAK-K--G-TM-----ADDD-AI-GAGDQGMMFGYATNETPEFMPFPIAIAHRLSRRLTEVRKNG--E-LSYLRPDGKTQVTVAY--E-N--G--K-PVHIEAI-VVSTQH-GPEVS---------LK----TIEK-DIKEKVIAPV-----V-P-S-------D-M-VD--DKT--KYYINPTGKFVIGGPQGDCGLTGRKIIVDTYGGMARHGGGAFSGKDPTKVDRSAAYAARYVAKNVVAAGLADKCEIQLAYAIGVAQPVSIMVDTFGTAK--IDEE----KIADLINKDFDLRPAGIIKMLDLRR-P-------IYAQTAAYGHFGR-T----------------------DID---L--P-WEHT-DK-A-----KLLK----Q----QAGL----------------

>Firmicutes_Pelosinus_fermentans

---------M-EK------------KC-V-LLTSESVTEGHPDKMADQISDGVLDAIMAKDP-----MGRVACETLLTTGQVHVVGEISTTC-YV--------DIPKIVRDTIKEIGYTR--AK-FGFDGETCGILVSIGEQSADIAMGV-NK-ALEAK-K--G-EM-----DEFE-AI-GAGDQGMMFGYATNETAEYMPLTISLAHKLAYRLTEVRKNK--E-VDYLRPDGKTQVTVEY--E-D--G--K-PVRVDTI-VIAAQH-SSEVD---------LA----TIEK-DMIEKVIVPI-----V-P-A-------E-L-LD--KNT--KYYINPTGRFVVGGPQGDAGLTGRKIIVDTYGGMARHGGGAFSGKDCTKVDRSAAYAARYVAKNIVAAGLADKCEIQLAYAIGIAYPVSIMVESFGTAK--VDEA----LIAELIKKHFDLRPAGIIKTLDLRR-P-------IYRQTAAYGHFGR-T----------------------DVD---L--P-WERI-DK-A-----EELH----K----EANL----------------

>Firmicutes_Veillonella_atypica

---------M-AE------------KH-M-FFTSESVTEGHPDKIADQISDAVLDAIIEKDP-----KARVACETLVTTGLVHVVGEISTST-YV--------DIPRVVRDTIREIGYTR--AK-YGFDCDTCGVLVSIDEQSADIAMGV-DE-ALESK-D--G-NS-----EIND-KI-GAGDQGMMFGYATNETPEYMPMPISLAHRLSRRLAEVRKNG--T-LTYLRPDGKTQVTVEY--V-D--N--Q-PKRIDTI-VISTQH-SPEVT---------QE----QIKN-DLKRYVIDAA-----L-P-A-------E-F-ID--ENT--KFFINPTGRFVIGGPHGDAGLTGRKIIVDTYGGMARHGGGAFSGKDPSKVDRSAAYAARYVAKNVVAAGLADKCEVQVAYAIGVAKPVSILVDTFGTAK--IAEE----KIQELVSKHFDLRPAGIIEMLDLLK-P-------HYRKTAAYGHFGR-T----------------------DVD---L--P-WERT-DK-A-----DILR----H----ETGL----------------

>Firmicutes_Megasphaera_massiliensis

---------M-VK------------NR-E-FFTSESVTEGHPDKMADQISDSILDAILAKDK-----NARVACETLVTTGMVHVVGEISTTC-YV--------DIPHIVRETIRSIGYTR--AK-YGFDCDTCGVLVSLDEQSPDIAMGV-DD-ALEKK-E--G-NT-----DQFD-TI-GAGDQGMMFGYATNETDDYMPLPVSLAHRLARRLASVRKDG--T-LSYLRPDGKTQVTVEY--E-D--G--K-PVRIDTI-VISAQH-SPEIT---------RE----QIEK-DLLEFVIKPE-----L-P-Q-------G-L-FD--DKT--KLFINPTGRFVIGGPQGDSGLTGRKIIVDTYGGMARHGGGAFSGKDPTKVDRSAAYAARYVAKNVVAAGLADKCEIQLAYAIGVARPVSIHVETFGTGK--VENE----KIVGLIEKNFDLRPAGIIAMLDLQR-P-------IYKQTAAYGHFGR-N----------------------DLN---L--P-WEQT-DK-A-----DTLR----K----EAGL----------------

>Firmicutes_Dialister_succinatiphilus

-----------MS------------KK-V-LFTSESVTEGHPDKIADQISDAILDAILAQDP-----NGRVACETIVTTGQVHVFGEITTTA-YV--------PIPQVIRKTINDIGYTD--AR-YGFDGESVGINVAIDEQSPDIAMGV-NQ-SLEAK-E--K-KA-----DKYD--I-GAGDQGMMFGYASNETPEYLPMPIALAHRLARRLAEVRKNG--D-VPYLRPDGKTQVTVEY--D-D--G--K-PVRVDTV-VISAQH-NPDVT---------HE----QIEE-DMINKVIKAT-----I-P-A-------R-F-LD--ENT--KYLINPTGRFVIGGPQGDAGLTGRKIIVDTYGGMARHGGGCFSGKDPTKVDRSAAYAARYVAKNIVAAGIADKCEIELAYAIGVAEPVSIFVDTFGTGK--IDDE----KIVDLIRRNFELRPAGIIDMLNLRR-P-------IYKQTAAYGHFGR-T----------------------DID---L--P-WEHT-DK-A-----DVLR----K----EAGL----------------

>Firmicutes_Succiniclasticum_ruminis

-------------------------MR-K-LFTSESVTEGHPDKIADQISDAILDAILEKDP-----EGRVACETVVATGQIHIVGEISTTC-FV--------DIPKIARDTVLGIGYDR--AK-YGFDGSTCGVLISIDEQSGDIAMGV-DK-SKEAK-E--G-AA-----AENE--M-GAGDQGMMFGYACDETPELMPLPISLAHKLARRLTEVRKNK--T-LAYLRPDGKTQVTVEY--E-D--G--K-PVRVDTI-VISTQH-DPDAT---------QD----QIRK-DMIELVIKEV-----V-P-A-------E-L-LD--ENT--KYFINPTGRFVIGGPQGDSGLTGRKIIVDTYGGMARHGGGAFSGKDPSKVDRSAAYAARYVAKNIVAAGLAKRCEIQLAYAIGVAQPVSILVDTFGTGV--IADD----KIAELVRKNFSLSPTGIIRDMDLRR-P-------IYKQTAAYGHMGR-T----------------------DID---V--P-WEHT-DK-A-----AALK----E----QAGI----------------

>Firmicutes_Acidaminococcus_timonensis

-------------------------MR-K-VFTSESVTEGHPDKIADQISDAVLDAILEKDP-----DARVACETLVATGQIHIVGEISTSC-YV--------DIAKIARQTVIDIGYDR--AK-YGFDGATCGVLISIDEQSPDIAQGV-DE-SDEAK-A--G-DH-----TDDE-KI-GAGDQGMMFGYATDETPEYMPLPISLAHKLARRLAQVRKEG--I-LPYLRPDGKTQVSVEY--E-D--D--K-PVRVNTI-VVSAQH-APEIS---------ME----QLRR-DILEYVIKPV-----V-P-A-------D-L-LD--EDT--IYHINPTGRFVIGGPQGDSGLTGRKIIVDTYGGMARHGGGAFSGKDPTKVDRSAAYAARYVAKNVVAAGLARKCEIQLAYAIGVARPVSISVNTYGTNA--IPEE----EIVKLIEKNFSLTPAGIIKSLDLRR-P-------IYKQTAAYGHFGR-T----------------------DVD---L--P-WERL-DK-V-----DALK----K----ALQ-----------------

>Firmicutes_Acetobacterium_woodii

-------------------------MK-K-LFTSESVTEGHPDKICDQISDAVLDAIFEQDP-----MARVACETMVTTGLVLVAGEITTDC-YV--------DIPKLVRNTVREIGYDR--AK-YGFDCDTCSVITAIDEQSTDIAMGV-DE-GWESK-K--G-QT-----SEIDLAT-GAGDQGMMFGYACNETPELMPLPISLAHQLAKRLTDIRKGK--V-VDYLRPDGKTQVTVEY--N-D--G--I-PTRIDTV-VISTQH-SSSVS---------SE----TIEK-DMIEKVIKPV-----I-D-P-------K-L-LD--EDT--KYFVNPTGRFVIGGPQGDCGLTGRKIIVDTYGGYGRHGGGAFSGKDPTKVDRSAAYAARHVAKNIVAAELADKCEVELAYAIGVAKPVSIYVETFGTGK--ISED----KMIALIEKCFDLRPAAIIKDLGLRA-P-------IYKQTAAYGHFGR-T----------------------DLD---L--P-WERL-DM-V-----EKLK----S----ML------------------

>Firmicutes_Marasmitruncus_massiliensis

------------M------------GK-Y-LFTSESVTEGHPDKICDQISDAVLDAIIEQDP-----TARVACETAVTTGMVMIMGEISTKC-YV--------DIPTLARSVIHEIGYDR--AK-FGFDSETCAVLTSIHEQSGDIAQGV-ND-ALEIR-E--G-QKD-E--AF---ST-GAGDQGMMFGYASDETPELMPMPISLAHKLARQLTKVRKDG--T-LGYLRPDGKTQVTVEYE-N-D-----S-PVRVDTV-VISNQH-SPEIP---------LE----QIRE-DIVQHVIRKI-----I-P-A-------E-L-MD--DRT--KIFVNPTGRFVIGGPQGDSGLTGRKIIVDTYGGFGRHGGGAFSGKDPTKVDRSAAYAARHVAKNIVAAGLAKRCEVQVAYAIGVARPVSVNVNTFGTGT--VSDE----RLAQAVDKVFDLRPAMIIKNLDLRR-P-------IYRQLAAYGHMGR-E----------------------DLG---V--S-WEKT-DK-T-----DAI-----------LAALK--------------

>Firmicutes_Phocea_massiliensis

------------M------------AK-Y-LFTSESVTEGHPDKICDQISDGILDAMLEKDP-----YARVACETTCTTGLVSIMGEISTSC-YV--------DIPKLAREIVKEIGYDR--AK-YGFDCDTCAVITTIDEQSTDIALGV-DN-ALEAK-N--A-QIQ-D--DL---VT-GAGDQGMMFGYACDETPELMPMAISLAHKLAKQLAKVRKDG--T-LSYLRPDGKTQVTVEYE-D-D-----R-PVRIDTI-VISNQH-SPEVT---------LE----QIRE-DILREVIRPI-----I-P-A-------E-L-LD--GET--KYYINPTGRFVIGGPQGDSGLTGRKIIVDTYGGYGRHGGGAFSGKDPTKVDRSAAYAARYVAKNIVAAGFAHKCEIQLAYAIGVAHPVSINVNTFGTGA--VSDE----ILEKAVAQVFDLRPAAIIKDLDLRK-P-------IYRKLAAYGHMGR-E----------------------DLG---V--A-WENR-DK-V-----DALK----E----AVAALTK-------------

>Firmicutes_Hydrogenoanaerobacterium_saccharovorans

------------M------------AR-H-LFTSESVTEGHPDKICDQISDAVLDSIIEQDP-----SARVACETTVTTGMVMLMGEITTNC-YV--------DIPKIVRGVISDIGYDR--AK-YGFDCDTCAVITTINEQSGDIALGV-DR-ALEAK-S--G-SSS-A--AED--ET-GAGDQGMMFGYACDETPELMPMPISLAHKLAKRLTEVRKNG--T-LSYLRPDGKTQVTIEYE-E-D-----K-PVRVDTV-VVSSQH-GPEVA---------LE----TIRE-DIIKHVIQPI-----V-P-A-------D-L-LD--SKT--KIFINPTGRFVIGGPQGDSGLTGRKIIVDTYGGYARHGGGAFSGKDPTKVDRSAAYAARYVAKNMVAAGFAKKCEIQLAYAIGVAAPVSVMVDTFGTGT--VNDD----LLSQAATHVFDLRPAAIIRELDLRK-P-------IYRQLAAYGHVGR-E----------------------DLD---V--Q-WEKT-DR-V-----DALK----K----YISEHK--------------

>Firmicutes_Butyricicoccus_porcorum

------------M------------GK-F-LFTSESVTEGHPDKICDQISDAVLDAILENDP-----QARVACETTVTTGLISIMGEISTHC-YV--------DIPKIARGVVREIGYDR--AK-YGFDCDTCAVVTTLDEQSSDIAMGV-DK-ALESK----A-DET-A--DL---TG-GAGDQGMMFGYACNETPELMPLPISLAHKMAKKLTEVRKAG--V-VDYLRPDGKTQVTVEYD-K-DT-N--K-PVHIDTV-VLSTQH-GPEAS---------LE----QIRS-DMIEYVIKPT-----L-P-A-------D-L-FD--EKT--RIFVNPTGRFVIGGPQGDSGLTGRKIIVDTYGGSAPHGGGAFSGKDPTKVDRSAAYAARYVAKNIVAAGLADKCQIQLAYAIGVAQPVSILVDTFGTGV--VDDE----VLETAVEKVFDLRPSAIIRDLDLRR-P-------IYRKLAAYGHMGR-T----------------------DLD---V--A-WEKT-DR-I-----DALK----A----AVAAR---------------

>Firmicutes_Intestinibacillus_massiliensis

------------M------------AK-H-LFTSESVTEGHPDKICDQISDAVLDAIIEQDP-----AARVACETTVTTGMVMVMGEISTQC-YV--------DIPHIARKVIEEIGYDR--AK-YGFDATTCAVLTSIDEQSADIALGV-DN-SLERK-E--G-ASD-A--DL---AI-GAGDQGMMFGYACDETPELMPLPISLAHAMAKRLTAVRKDG--T-LPYLRPDGKTQVTVEYD-E-N--N--Q-PVRVDTV-VLSTQH-SPDVS---------LT----QIRR-DMLEHVIRPV-----V-P-A-------Y-L-LD--DNT--RFFVNPTGRFVVGGPQGDSGLTGRKLIVDTYGGMARHGGGAFSGKDPTKVDRSAAYAARYVAKNIVAAGLATRCEVQLAYAIGVAEPVSIMVDTSGTGK--VDDA----VLAAAIPQVFDLRPAAIIETLELRR-P-------IYRQLAAYGHLGR-E----------------------DLG---V--K-WEQT-DR-T-----DALR----A----AVAAQ---------------

>Firmicutes_Agathobaculum_desmolans

------------M------------AH-H-FFTSESVTEGHPDKICDQISDAILNEILTHDP-----SARVACETTCTTGMVQVMGEISTTC-YV--------DIPKIVRDTVREIGYDR--AK-YGFDCDTCAVLTAIDEQSEDIALGV-DN-SLERK-E--G-AED-A--DL---AI-GAGDQGMMFGYACDETPEKMPLPISLAHKLAKRLAEVRKNG--M-FTYLRPDGKSQVTVEYD-E-D--N--C-PVRVDTV-VISTQH-SPEVS---------LQ----QIRH-DMIEQVVRQV-----I-P-A-------A-L-LD--ENT--KYFINPTGRFVVGGPQGDSGLTGRKIIVDTYGGIGRHGGGAFSGKDPTKVDRSGAYAARWVAKNIVAAGLARRCEIQLAYAIGVAEPVSIMVETFGTGT--VAED----RLAEAVRRVFDLRPAAIIETLELRR-P-------IYKALAAYGHMGR-E----------------------ELG---V--K-WEQT-DK-V-----EELK----K----ALA-----------------

>Firmicutes_Anaerotignum_lactatifermentans

------------M------------SR-R-LFTSESVTEGHPDKICDQISDAILDTILAKDP-----KARVACETCTTTGIIFVMGEITTSA-YV--------DVEGIVRDTLRQIGYDR--AK-YGFDCDTCSVITSIHGQSPDIALGV-DK-ALENK-T--G-ED--D--SEFD--T-GAGDQGMMFGFACDETEELMPLPISLSHRLTKRLAEVRKNG--T-LSYLRPDGKAQVTVEY--V-D--D--I-PVRVDTV-VVSTQH-DPEAT---------QE----QIRA-DIIEHVVKKV-----I-D-A-------N-L-LD--ENT--KYYINPTGRFVIGGPQGDSGLTGRKIIVDTYGGYAAHGGGAFSGKDPTKVDRSACYAARYVAKNIVASGIASKCEVQLAYAIGVAKPVSILVNTFGTGK--ISDD----KITELVQRNFDLRPAAIIKNFDLLR-P-------IYKQVAAYGHFGR-T----------------------DLD---L--P-WEKT-DK-T-----DVFQ----I------------------------

>Firmicutes_Symbiobacterium_thermophilum

----MT---E-QK------------GR-Y-LFTSESVTEGHPDKMADQISDAVLDAILALDP-----RARVACETLLTTGLVVVTGEITTHA-YV--------DIPNLVRDVVRDIGYTR--AK-YGFDGSTCGVMTAIDPQSPDIAQGV-DV-AIEVR----G-EVN-E--KELE--I-GAGDQGMMFGFACDETPELMPLPISLAHRLARRLAQVRKNG--T-LPYLRPDGKTQVTIEY--E-D--G--R-PKRIDTI-VISAQH-DPDTT---------QE----QIRK-DVIDYVILDV-----V-D-R-------N-L-ID--DRT--RYFINPTGRFVVGGPQGDTGLTGRKIIVDTYGGYARHGGGAFSGKDPTKVDRSGAYAARWVAKNIVAAGLARKCEVQVAYAIGVAHPVSILVTTFGTGK--LPDE----RLAQLVRETFDLRPGAIIRDLDLRR-P-------IYRQVAAYGHFGR-P----------------------DLD---L--P-WERT-DK-V-----DLLR----E----KAGI----------------

>Firmicutes_Proteiniclasticum_ruminis

------------M------------KR---LFTSESVTEGHPDKICDQISDAILDSILEQDP-----DARVACETAVTTGMVMVMGEITTKC-YV--------DIPKIVRKTVTEIGYVR--AK-YGFDAETCSVLTSIDEQSPDIAQGV-DS-SLETR------EGQ-E--DALL-QT-GAGDQGMMFGFATNETEDFMPMPINLAQKLARRLAEVRKNG--T-LTYLRPDGKTQVTVEYD-E-N--D--Q-PVRVDTI-VVSTQH-GADVT---------QE----EIRR-DLIEQVINAV-----I-P-E-------S-L-MD--KET--KIFINPTGKFIVGGPQGDSGLTGRKIIVDTYGGYGRHGGGAFSGKDPTKVDRSAAYAARWVAKNLVAAGVADKLEIQLSYAIGVAKPISIEVFTFGTEK--VSHE----VIIEIVKRVFDLRPGAIIRDLDLRK-P-------IYRQIAAYGHFGR-N----------------------DLD---L--T-WEKL-NK-V-----EDIKA-ALK----EITA----------------

>Firmicutes_Traorella_massiliensis

------------M------------SK-K-FFTSESVTEGHPDKICDAISDAVLDAILEKDP-----MARVACETCTTTGMVLVMGEITTNC-YV--------DIPKIARNVVLEIGYDR--AK-YGFDGNTCAVLSAIDEQSGDIAMGV-DA-ALETR-E--S-DDH---------EN-GAGDQGMMFGYACDETKELMPMPIMLAHKLARQLAKVRKDG--T-LSYLRPDGKSQVTVEYD-G-D-----E-VKRIEAV-VISTQH-SDAVE---------LS----QIRE-DIIHHVINEI-----I-P-A-------E-L-MD--ENT--KIYVNPTGRFVIGGPQGDSGLTGRKLIVDTYGGMARHGGGAFSGKDPTKVDRSAAYAARWVAKNLVAAKLAKKVEIQLAYAIGVAHPLSIHVDTFGTST--YSEE----QLIEIINKVFDLRPSAIIDTLDLRR-P-------IYRQLAAYGHFGR-D----------------------DLD---I--P-WEKT-NK-V-----EEIL----N----CLK-----------------

>Firmicutes_Halanaerobium_hydrogeniformans

-----------MT------------KK-Y-LFTSESVTEGHPDKLADQISDAVLDAILAKDP-----DARVACETFVTTGLILISGEITTSC-YV--------DLTKIARKTVNDIGYTR--AK-YGFDGETCAVLTAIDEQSGDIALGV-DH-SLEEK-E--G--------KSQS-EL-GAGDQGLMFGYASDETEELMPLPIMLAHKLARRLAEVRKDG--S-LDYLRPDGKTQVTVAY--E-G--N--T-PLYVDKV-VVSSQH-HPDIS---------QK----KIRK-DIIEHVIKAI-----I-P-A-------D-L-LK--EQS--KFLINPTGRFVIGGPNGDSGLTGRKIIVDTYGGTSRHGGGAFSGKDPTKVDRSASYAARYIAKNVVAAKLAKKCEVQISYAIGVAEPLSLMVDTFGTGI--IEDE----RLTEIVDQEFDLRPAQIIKTLNLRS-P-------IYKQVAAYGHFGR-H----------------------DLD---L--S-WEKT-DR-V-----KSLR----K----KAGLE---------------

>Firmicutes_Halothermothrix_orenii

-----------MG------------RR-Y-LFTSESVTEGHPDKIADQISDAVLDEILSKDS-----QARVACETFVTTGLVLVSGEISTDC-YV--------DVPHIARETIKKIGYDR--AK-YGFDGDTCSVLTAIDEQSPDIAIGV-DK-SLESK-V--G--------IDEN-QL-GAGDQGLMFGYACNETPELMPLPIILAHKLARRLAEVRKKG--I-LPYLRPDGKTQVTVEY--E-D--D--R-PVRIDKV-LVSAQH-HPDIS---------ST----QIKE-DITRHVINYI-----I-D-E-------N-L-RD--DRT--EILVNPTGRFVIGGPHGDTGLTGRKIIVDTYGGIARHGGGAFSGKDPTKVDRSAAYAARYVAKNIVAAGLADRCEVQLAYAIGVARPVSIMVDTFGTGK--VSHD----ILVKLINENFDLRPASIIRNLDLRK-P-------IYSKVAKYGHFGR-S----------------------DLD---L--T-WERT-DK-S-----EAL----------KAGAETLAG-SKIPED----

>Firmicutes_Catabacter_hongkongensis

------------M------------RR-L-LFTSESVTEGHPDKVCDQISDSVLDAILEQDP-----NARVACETAVNTGLVLVMGEITTNA-YV--------DIAKIARETVKQIGFDK--AD-YGFDGETCGVITSIDEQSPDIAMGV-DK-ALEVR------EDN-D--DD-A-EN-GAGDQGMMFGYACNETPEYMPMPIYLAHRLSRRLAEVRKNG--T-LEYLRPDGKSQVTIEYD-E-N--W--K-PMRVDAV-VISTQH-DPEAT---------QA----QIRR-DMIEQVINPM-----I-P-A-------D-M-MD--DDT--KIYVNPTGRFVIGGPKGDSGLTGRKIIVDTYGGMGRHGGGAFSGKDPSKVDRSAAYAARYVAKNVVAAGLADRCEIQLAYAIGVARPVSVFVETFGTGK--KHST----EIADIITKLFDLRPTAIIERLQLRK-P-------IYRQTAAYGHFGR-E----------------------DLD---L--P-WEKL-DM-V-----EAIK----K----EI------------------

>Firmicutes_Cuneatibacter_caecimuris

------------M------------EK-F-LFTSESVTEGHPDKMCDQISDAILDALLEQDP-----MSRVACETCATTGLVLVMGEVTTKG-YV--------DIQKVVRDTVREIGYTR--GK-YGFDADTCGVIVALDEQSADIALGV-DK-ALEAK-E--N-QMS-E--EEIE-AT-GAGDQGMMFGYATNETEEYMPYPIAIAHKLARQLTKVRKDG--T-LDYLRPDGKTQVTVEYD-E-K--H--R-PIRLDTV-VLSTQH-DEKVT---------QE----QIHQ-DIKKYVFDPI-----L-P-A-------D-M-VD--GET--KFFINPTGRFVIGGPHGDSGLTGRKIIVDTYGGAARHGGGAFSGKDCTKVDRSAAYAARYVAKNIVAAGLADQCEIQLAYAIGVAHPMSVMVDTAGTGK--VSDE----KLVEIIRENFDLRPAGIINMLNLRR-P-------IYKQTAAYGHFGR-N----------------------DLN---L--P-WERL-DK-V-----EELK----K----YLQ-----------------

>Firmicutes_Blautia_schinkii

------------M------------EK-I-LFTSESVTEGHPDKMCDAISDAILDALMEQDP-----MSRVACETATTTGLVMVMGEITTKA-YV--------DIQKIVRDTVREIGYTR--GK-YGFDADTCGVITAIDEQSPDIALGV-DK-ALELK-E--N-DMN-E--DELD-AI-GAGDQGIQFGYASNETEEYMPYAINMAHKLAQQLTKVRKDG--T-LTYLRPDGKTQVTVEYD-E-Q--G--K-PVRIDAV-VCSTQH-DPDVT---------QE----QIHK-DIKKFVLDAI-----I-P-A-------E-M-VD--ENT--KYFINPTGRFVIGGPHGDSGLTGRKIIVDSYGGYGRHGGGAFSGKDCTKVDRSAAYAARYVAKNIVAAGLADKCEIQLSYAIGVARPTSINVETFGTGR--LSDS----KLVEIIRENFDLRPAGIIKMLDLRR-P-------IYKQTAAYGHFGR-T----------------------DVE---L--P-WEKL-DK-A-----EELK----K----YLA-----------------

>Firmicutes_Eubacterium_plexicaudatum

------------M------------EK-L-LFTSESVTEGHPDKVCDAISDAVLDACMEQDP-----MSRVACETACCTGFVLVTGEITTKA-QL--------DIPSIVRKTVNEIGYND--AK-MGFDCNTCAVFSALDRQSADIAMGV-DK-ALEAK-E--N-KMT-D--EQLD-AI-GAGDQGMMFGYATNETESYMPYPISLAHKLALQLTKVRKDG--T-LTYLRPDGKTQVSVEYD-E-A--G--K-PKRLEAV-VLSTQH-DTEVT---------QE----QIHA-DIMKHVFDPV-----I-P-K-------D-M-VD--ADT--KYFINPTGRFVIGGPQGDAGLTGRKIIVDTYGGYARHGGGAFSGKDCTKVDRSAAYAARYVAKNIVAAGLADKCEIQLSYAIGVAQPTSVMVDTFGTGK--LSDA----QLVGLVRENFDLRPAGIIKMLDLRR-P-------IYKQTAAYGHFGR-N----------------------DLN---L--P-WEAL-DK-V-----DVLK----K----YLA-----------------

>Firmicutes_Lachnoanaerobaculum_saburreum

-----------ME------------ER-R-LFTSESVTEGHPDKMCDQISDAILDAYLGADP-----DSRVACETATTTGMVLVMGEISSKA-TV--------DIQKIVRETVREIGYDR--AK-YGFDCDTCSVLVALDEQSKDIAMGV-DK-ALESR-E--FIDKE-D--EDN-----GAGDQGLMFGYATDETKEFMPYPIYIAHNLAKRLTQVRKDG--T-LKYLRPDGKTQVTVEYD-E-N--D--K-PVRIDTV-VLSTQH-DEDVT---------QE----QIHE-DIRKYVFDEV-----I-P-K-------D-M-VD--ENT--KYFINPTGRFVIGGPHGDSGLTGRKIIVDTYGGYARHGGGAFSGKDSTKVDRSAAYAARYVAKNIVAAGLAKKCEIQLSYAIGVAHPTSISVDTFGTGK--ISDS----AIVELIRRHFDLRPTGIIKMLDLRK-P-------IFKQTAAYGHFGR-D----------------------DLN---L--P-WERL-DK-V-----EELK----K----GI------------------

>Firmicutes_Johnsonella_ignava

-----------MS------------EK-R-LFTSESVTEGHPDKMCDQISDAVLDALLELDP-----LSRVACETCATTGFVMVMGEVTTKA-YV--------DIQKIVRDTVREIGYNR--AK-YGFDADTCGVIVSLDEQSPDIAMGV-DK-SLEAK-L--GTEKD-D--LNI-----GAGDQGMMFGYATDETEEYMPYPISLSHKLAKRLSDIRKDG--T-LKYLRPDGKVQVTVEYD-E-D--N--K-PVRIDTV-VLSAQH-DENVT---------HE----ELER-DIKKFIFDKV-----L-P-K-------H-M-VD--DNT--RFYVNPTGRFVIGGPHGDSGLTGRKIIVDTYGGFARHGGGAFSGKDCTKVDRSAAYAARYAAKNIVASGLASKCEIQLSYAIGVAHPTSISVNTFGTGK--IRDD----ELCRIVREHFDLRPAGIIKMLDLRR-P-------IYKQTAAYGHFGR-T----------------------DIK---L--P-WEEL-DK-V-----EEIK----R----AAQLLS--------------

>Firmicutes_Lachnospira_pectinoschiza

------------M------------EK-R-LFTSESVTEGHPDKICDQISDAVLDALYAQDP-----YSRVACETLTNTGFVMVMGEITTKA-NV--------DIPQIVRDTVVEIGYDS--SE-KGFDGNTCAVMVALDKQSADIAMGV-DK-ALEAK-E--GVDKE-D--DDL-----GAGDQGMMFGYATNETEEYMPYSAALAQKLSKQLTKVRKDG--T-LSYLRPDGKTQVTVEYD-E-N--N--K-PIRLDAI-VVSSQH-APDVT---------QE----QIHE-DIKKYVIDAI-----V-D-P-------A-M-VD--ADT--KIFINPTGRFVIGGPNGDSGLTGRKIIVDTYGGAARHGGGAFSGKDCTKVDRSAAYAARYVAKNLVAAGIADRCEIQLSYAIGVAHPTSICVDTFGTGK--LSDE----KIVEIIRENFDLRPNGIIKMLDLRR-P-------IYKQTASYGHFGR-T----------------------DLN---L--P-WEQL-DK-V-----DALK----K----YL------------------

>Firmicutes_Eubacterium_eligens

------------M------------EK-R-LFTSESVTEGHPDKICDQISDAVLDALYEQDP-----YSRVACETLTNTGFIMVMGEVTTKA-NI--------DIPSIVRKTVTDIGYDS--SD-KGFDGNTCAVMVALDKQSADIAMGV-DK-ALEAK-D--G-EID-D--SEG-----GAGDQGMMFGYATNETPEFMPYSSALAQKLSKQLTKVRKDG--T-LSYLRPDGKTQVTVEYD-E-N--K--K-PIRLDAI-VVSSQH-APEVS---------QE----QIHE-DIKKYVIDAI-----V-D-P-------A-M-ID--ADT--KIYINPTGRFVIGGPNGDSGLTGRKIIVDTYGGAARHGGGAFSGKDCTKVDRSAAYAARYVAKNIVAAGLADRCEIQLSYAIGVARPTSIMVDTFGTGK--LDEE----KIVEIIRENFDLRPNGIIKMLDLRR-P-------IYKQTAAYGHFGR-T----------------------DLN---L--P-WEKL-DK-V-----DVLA----K----YLG-----------------

>Firmicutes_Stomatobaculum_longum

------------M------------ER-R-LFTSESVTEGHPDKICDQISDAVLDALLAEDP-----ESRVACETSTTTGLVVLMGEISTTA-RI--------DYQQLVRNTIREIGYTR--GK-YGFDCDTCAVLTALHEQSPDIAQGV-NH-ALEER-E--GEKEW-D--LNQ-----GAGDQGLMFGYACRETEELMPYPISLAHKLCRKLTELRKTK--K-LPYLRPDGKSQVTVEYD-E-N--G--K-PARLDAV-VLSTQH-DPDVS---------QE----KIHA-DIKKYIFDEV-----L-P-A-------E-L-VD--EHT--RFYINPTGRFVIGGPNGDAGLTGRKIIVDTYGGYARHGGGAFSGKDPSKVDRSAAYAARYVAKNLVAAGLADSVEIQLSYAIGVATPTSVSVNSFGTGK--LSDE----QLCDIVRKEFDLRPAGIIRMLDLKR-P-------IYRQTAAYGHFGR-E----------------------DLS---L--P-WEKT-DR-A-----EQLK----K----YLA-----------------

>Firmicutes_Natribacillus_halophilus

-----M---E-AN------------GR-R-LFTSESVTEGHPDKICDQISDAILDDILTKDP-----NARVACETVVNTGMILVTGEITTTT-YV--------DISKVARDTLKKIGYTR--AK-FGLDADTCAVMTAIDEQSPDIAQGV-DT-ALESR-E--G-RMS-E--AEME-AI-GAGDQGLMFGYADNETEEYMPLPIYLAHQLARRLSTVRKEG--I-VPFLRPDGKTQVTIEYD-K-H--D--R-PLRVDTI-VISTQH-QPDVT---------IV----EIEK-EIQAHVISAV-----V-P-E-------H-L-ID--KET--KVLINPTGRFVIGGPQGDAGLTGRKIIVDTYGGIARHGGGAFSGKDATKVDRSASYAARYVAKNIVAAGLAKKCEVQLAYAIGVARPVSISVDTFGTAK--VKES----LLLQLVEKHFDLRPAGIIRMLDLRR-P-------IFEQTAVYGHFGR-P----------------------DID---L--P-WERT-DK-A-----ALLN----E----DAFAAETK------------

>Firmicutes_Lactobacillus_panisapium

------------M------------EK-R-LFTSESVSEGHPDKVADQISDAILDAIIAQDP-----DAHVACETIVNTGIVYVFGEISTSA-YV--------DIQSIVRKTVLRIGYDK--PE-LGFDGNNCAVLVGIDEQSPDIAGGV-DH-SLETR-E----DKSDN--DQLD-QI-GAGDQGLMFGFAIDETPELMPLPISLAHRLMRQVAKLRKEG--T-LTWLRPDAKAQVTVEYD-D-N--N--K-PKRVDTV-VISTQT-DDQVS---------NE----DIRQ-AMIDLVIKKV-----I-P-A-------K-Y-LD--AET--RFLINPSGRFVIGGPKGDSGLTGRKIIVDTYGGYARHGGGAFSGKDPTKVDRSASYAARYVAKNIVAAGLAKRCEVQLAYAIGVAHPVSVMIDTANTGK--VSDE----LLTKAVRSVFDLRPAGIIKMLDLRR-P-------IYEQTAAYGHFGR-T----------------------DID---L--P-WEKT-DK-I-----QALL----D----FVKENK--------------

>Firmicutes_Ignavigranum_ruoffiae

------------M------------YK-K-LITSESVTEGHPDKIADQISDAILDSIIIQDP-----NARVAVETAVTTGLVFVFGEISTSA-YV--------DIQKIVRQKIAEIGYTD--RK-YGFDADNVAVLVSLDEQSADIAQGV-DR-ALESR-D----QVD----NSAE-TL-GAGDQGIMFGFASRETESYMPLPIDLSHRLARKLAEVRKMG--P-LTYLGPDGKTQVTIEYD-E----AM-N-VRRIDNI-VVSSQH-AEEIS---------QE----QLHK-DIFEHVIKPV-----C-P-A-------E-W-LD--ERT--KYFINPTGKFVIGGPVGDSGLTGRKIIVDTYGGIAHHGGGAFSGKDATKVDRSASYMARYVAKNIVAAGLADRCEILLSYAIGVAQPTSINVNTFDTGH--YSET----KIEAMIERVFDLTPQGIIDHLQLRR-P-------IYSQTAAYGHFGR-----------------------IDLD---L--P-WERL-DK-V-----QEIQNFM-Q----KA------------------

>Firmicutes_Mogibacterium_diversum

-------------------------MK-K-LFTSESVTEGHPDKVCDQISDAILDAILAEDP-----HAHVACETATKTGFVIVFGEISTTC-TV--------DIEGIVRGVVKSIGYDC--SE-VCFDGNTCAVLVSIEEQSPDIAMGV-NE-SFEVK-E--GKEAD-D--DAMT----GAGDQGMMFGFACDETEELMPMPISLAHQLARRLAEVRKDG--T-VSYLRPDGKTQVTVEY--D-D--D--K-VVRVDTV-VISTQH-DPDAT---------LE----QIRR-DMIEHVIKPI-----I-P-A-------E-L-ID--DET--KLFVNPTGRFVIGGPKGDSGLTGRKIIVDTYGGYASHGGGAFSGKDPTKVDRSGAYMARYIAKNIVAAGLARKCEIELAYAIGVAEPVSVYVNTYGTAS--LKDE----DIAKLVRENFDMRPAAIIKQLDLLK-P-------IYRALAAYGHFGR-D----------------------ALG---V--R-WEDT-DK-A-----EQLKAAAEK----FA------------------

>Firmicutes_Peptoniphilus_timonensis

------------M------------KK-W-QFSSESVTEGHPDKVCDQISDAILDKILAEDK-----NARVACETMASTGMIVITGEISTET-YV--------DFEKTAREVLEDIGYNR--AK-FGFDGRTCAVLSAIKEQSSDIAMGV-DR-FKE---E--G-----V--DELD-SF-GAGDQGIMFGFACNETEELMPLPISLAHKLSRRLTQVRKDG--T-LDYLRPDGKTQVTVEY--E-E--D--K-PVRLKNV-VVSSQH-APDVS---------ME----KIRE-DIIREVVEKV-----V-P-E-------D-L-ID--ADT--EFFINPTGRFVIGGPMADAGLTGRKIIVDTYGGFGRHGGGAFSGKDPTKVDRSGAYYARYIAKNLVAAGLADKVEIGLAYAIGIARPTSVYVETFGTGK--LSDE----EIEKIIMENFDMRPAAIIRDLDLLR-P-------IYRQTASYGHFGR-N----------------------DLD---L--P-WEKT-DR-A-----EELK----K----YLK-----------------

>Firmicutes_Intestinimonas_massiliensis

------------M------------SH-R-VFTSESVTEGHPDKVCDQISDAVLDDILASDP-----TAHVACETFTTTGMIVVMGEITTSH-YT--------DIPAIARKTVQRIGYTD--PA-YGFDYRSCAVMTAIDEQSPDIAMGV-NQ-SYEVQ-Q--G-GDS----DPLD-LV-GAGDQGMMFGYACDETAELMPAPISMAHQLCRRLTEARKSG--E-LTWLRPDGKSQVTVEYD-A-D--G--S-VLRCPAI-VVSTQH-DPDIA---------LK----DLRE-AVTETIIRPV-----I-P-A-------R-F-ID--KET--KIYVNPTGRFVVGGPVGDTGLTGRKIIVDTYGGSASHGGGCFSGKDPTKVDRSAAYMARYAAKNLVAAGLARKCQIELAYAIGVAHPVSVLVDTFGTGR--LEDE----ALAQIVDKHFDLRPAAIIRDLDLRR-P-------IYEQTAAYGHFGR-T----------------------DID---L--P-WERT-DR-A-----ETLK----Q----YLK-----------------

>Firmicutes_Weissella_oryzae

-----------MV------------EK-R-LFTSESVSEGHPDKVSDQISDAILDAILAKDP-----MAHTAIETSVTTGYVNVFGEISTSA-YV--------NMDEIIRKTLARIGYDD--PD-AGFLASDVHVGLTIDEQSPDIAQGV-NS-AIEQR------EEGGV--DPLD-EI-GAGDQGLMFGFATNETDEYLPLSVVLSHKLVKKQAELRRTG--E-LAYLLPDAKAQVTVELD-E-H--D--E-PKRIDAV-VLSTQH-RDNVT---------LE----QLRK-DVRAHILDEI-----L-P-A-------E-M-VD--ADT--KYYINPTGRFVIGGPKGDAGMTGRKIIVDTYGGYARHGGGAFSGKDATKVDRSAAYATRHIAKNIVAAGLADKAEIQVAYAIGVAHPVSVNVNTFGTGR--VSDD----QLTEAIRDLFELRPAGIIEDLDLRK-P-------RYEATAAYGHFGR-P----------------------ELD---L--P-WERL-DK-V-----DELK----A----YFGKLG--------------

>Firmicutes_Sporosarcina_koreensis

---------M-A-------------EQ-I-LFTSESVSEGHPDKIADQISDAVLDAALAQDP-----FSRVACEVFTTTNTVIVGGEITTNA-VL--------DIEGIARRTLLDIGYTN--DE-LGIDGSSCNVQVLVHTQSPDIAMGV-DT-S-SDT-K----------------EI-GAGDQGIMFGFATDETDSSMPLAIELAHALVRKASVLRKNG--Q-FRWARPDMKSQVTIDYT---A----EE-TPKIHTI-LMSIQH-DGDFD---------KE----AFEA-YVKEEIIGQT-----V-A---------E-FGLS--GEY--RVLINPTGRFVTGGPHGDAGLTGRKIIVDTYGGAARHGGGAFSGKDCTKVDRSAAYAARYVAKNIVAAGLANKCEIQLSYAIGVSEPISIAIDTFGTGK--APDA----ELLRAVREVFNLTPAGIISMLDLRT-P-------RYQATAAYGHFGRTE----------------------------LDLP-WERK-DK-A-----EALM----QSVFGAVGAGC--------------

>Firmicutes_Agitococcus_lubricus

---------M-SD------------Y--S-VFTSESVSEGHPDKIADQISDAVLDAIIAQDK-----YARVACETMVKTGVAIVSGEITTSA-WV--------DLEDLVRQVINDIGYNS--ST-VGYDGSTCGIINIIGKQSVDIAQGV-DR-ARS---E----------------DQ-GAGDQGLMFGYASNETDVLMPAPICYAHRLVERQAEVRKTG--L-LPWLRPDAKSQVTFRY--E-N--G--K-PVAIDAV-VLSTQH-NPDIA---------YA----DLQE-AVMELIIKQV-----L-P-A-------E-W-LH--KGT--KFHINPTGQFIIGGPVGDCGLTGRKIIVDSYGGMARHGGGAFSGKDPSKVDRSAAYAGRYVAKNIVAAGLADRCEIQVSYAIGVAEPTSISLNTFGTGK--ISDD----KIIDLVRRHFDLRPYAITKMLDLLH-P-------MYKPTAAYGHFGR-TPQTMTVG------------DDTFTT---F--T-WEKT-DR-A-----AALK----A----DAGL----------------

>Firmicutes_Kandleria_vitulina

---------M-K-------------EK-I-LFTSESVSEGHPDKVCDQISDAILDACLAEDP-----DSRVACEVFATTNFVVIGGEITTAA-KV--------DYEKVARDVIRRIGYTS--DE-IGIDADHCEIKVVMDTQSKDIALGT-N------N-E----------------VG-GAGDQGIMFGYATNETEGYMPLPVSIAHHLVRYASELRHDG--T-FKWARPDMKSQVTVDYT---D----EK-HPRIDTI-LMSVQH-DEDFD---------ED----AFKT-FIKNDIMDKV-----V-D---------K-YGLN--HDY--KVYINPTGRFVIGGPHGDTGLTGRKIIVDTYGGMARHGGGAFSGKDPTKVDRSAAYMARYIAKNIVAAGACDRCEIQLSYAIGIKEPTSIFVETYGTEK--TSID----NILHAIKNEFDLTPKGIIDTLDLQR-P-------IYSSTAAYGHFGRKE----------------------------Y--P-WEAL-DR-V-----DAIK----KYL----------------------

>Firmicutes_Virgibacillus_halodenitrificans

---------M-SE------------Y--S-LFTSESVSEGHPDKIADQISDAVLDAIIARDK-----QARVACETMVKTGVAIVAGEITTSA-WV--------DLEDLVRHVINDIGYTS--SE-VGFDGNTCGVLNLIGKQSVDIAQGV-DR-SKP---E----------------DQ-GAGDQGLMFGYATDETDSYMPAPIHYSHRLVERQAELRKHG--L-LPWLRPDAKSQLTFRYD-A-H--G--K-PCSVDAI-VLSTQH-DPDID---------QE----ELRD-MIKREIIEEV-----I-P-A-------E-W-LT--EDT--KYHINPTGNFVIGGPVGDCGLTGRKIIVDTYGGMARHGGGAFSGKDPSKVDRSAAYAGRYVAKNIVAAGLAERCEIQISYAIGVAEPTSVSVDTFGTGK--ISDA----KIVELVREHFDLRPNAITSMLNLLH-P-------MYQLTAAYGHFGR-EPFEHSYTWTDANGEQ---QTETFTA---F--P-WEKV-DR-A-----DALR----Q----AAGL----------------

>Fusobacteria_Sebaldella_termitidis

---------M-SKN---------------LFFTSEFVSPGHPDKICDQISDAVLDACLVDDP-----QSRVACEVFATTGLVMVGGEITTNT-YI--------DIQKIIREKIEEIGYRQG----MGFDSD-CGVLNTIHSQSPDIAMGV-DT-G-------------------------GAGDQGIMFGGAVNETPELMPLALTLARSIINRLTVLTRSK--E-LAWARPDAKAQVTLAYD-E-N--G-EK-VEYVDTV-VLSVQH-DEEVT---------QE----EIKR-DLKEKVIRPV-----L---E-------K-YGLDS-EKV-KKYHINPTGRFVIGGPHGDTGLTGRKIIVDTYGGFFRHGGGAFSGKDPSKVDRSAAYAARWIAKNIVSAGIADKCEVQLSYAIGVAEPTSIKVDTFGKSK--VSEE----KIAEAVAEIFDLTPRGIEKSLELRE-GKF-----RYQDLAAFGHIGR-T----------------------DID---L--P-WERV-NK-A-----EELK----K----ILL-----------------

>Fusobacteria_Hypnocyclicus_thermotrophus

-----M---I-EK------------GR-V-FFTSESVTEGHPDKMADQISDAILDAILKEDP-----KARVACETMLTTGLVVIAGEITTAT-YV--------DFQKIVRNTVKEIGYTR--AK-YGFDFETCAVINSIHEQSADIAMGV-DE-SLEIK-E--NKSKK-E--DERE-LL-GAGDQGIMFGFATNETEELMPMPIILAHKLARKLSEVRKNN--T-LDYLRPDGKTQVTVEY--V-D--G--K-PVRIDAI-VISTQH-DSDIT---------HD----KIEH-DMKKYVINSI-----I-P-E-------E-L-LD--ENT--KYYINPTGRFVIGGPHGDAGLTGRKIIVDTYGGMGRHGGGAFSGKDPTKVDRSAAYAARWVAKNIVAAGLADKCEIQLSYAIGVASPTSIAVNTFETGK--VADD----TIVKAVKKVFDLRPGAIIQDLDLRR-P-------LYRQVAAYGHFGR-I----------------------DLD---L--P-WEKT-NK-V-----EELKK-IVE----ELK-----------------

>Fusobacteria_Leptotrichia_buccalis

---------M-TKK---------------IYFTSEFVSPGHPDKICDQISDSILDACLADDE-----NSRVACETFATTGLVVVGGEITTKT-YV--------DVQKIVRDKISEIGYRPG----MGFDSD-CGVLNTIHSQSPDISMGV-DT-G-------------------------GAGDQGIMFGGAVNETEELMPLALVLSRGIIQKLTEITRNG--T-LAWARPDAKAQVTLAYD-E-N--G--K-LLNVDTV-VLSVQH-NEDVT---------NE----QIEK-DLKELVIKPV-----L---E-------K-YDLNI-ENV-RKFHINPTGRFVIGGPHGDSGLTGRKIIIDTYGGYFRHGGGAFSGKDPSKVDRSAAYAARWIAKNIVAAGFATKCEVQLSYAIGVVEPVSIRVETFGTGT--VEET----RIEEAVAKLFDLTPNGIQKSLNLRK-PSF-----RYQDLAAFGHIGR-T----------------------DID---L--P-WEKL-DK-V-----EGLK----K----ELEK----------------

>Fusobacteria_Streptobacillus_hongkongensis

---------M-SK----------------FYFTSEFVSPGHPDKICDQISDAVLDACLSEDP-----ESRVACETFATTGFVLVGGEITTKT-YV--------DIQSLVRNKIKEIGYVPG----LGFDYD-CGVINMIHSQSPDISQGV-DI-G-------------------------GAGDQGIMFGGAVKETPELMPLALVLSRSIIVKLTELTRNK--V-LPWARPDAKAQVTLEYD-D-N--N--N-IVGIDSI-VLSVQH-DEEIT---------LD----EIKK-QVKELVIAPA-----L---K-------E-YGRDI-SEV-KNFYINPTGRFVIGGPHGDTGLTGRKIIVDTYGGYFRHGGGAFSGKDPSKVDRSAAYAARWIAKNIVGSGIADKCEVQLSYAIGVIEPTSILVETFGTSS--YSNE----EIANVVKEIFDLTPRGIEKSLSLRK-PNF-----RYQDLAAFGHIGR-K----------------------DIE---L--P-WEKL-DK-I-----EEIK----K----GLIR----------------

>Fusobacteria_Sneathia_amnii

---------M-NNN--------------YSFFTSEFVSPGHPDKICDQISDAILDECLADDV-----NSRVACETFATTGLILIGGEITTNT-YV--------DVQTIARGVIKEIGYKPG----LGFDYD-CGVINTIHAQSPDISMGV-DT-G-------------------------GAGDQGIMFGGAVDETPELMPLAITLAREIIKRLTKLTRDK--T-LPWARPDAKSQVTLKY--D-N--D--K-IVGIDAI-VLSVQH-DESVT---------QD----MIKE-EVKNLVFEPV-----L---R-------K-YGYDL-QDV-KKIFINPTGRFVIGGPHGDTGLTGRKIIVDTYGGYFRHGGGAFSGKDPSKVDRSAAYMARYIAKNIVAAKLAKKCEVQLSYAIGVVDPISIKVETFNTSN--VPEQ----KIEEVIPQLFNLSPRGIEKTLNLRT-PNF-----KYKDLAAFGHIGR-T----------------------DIE---L--P-WEKI-DM-V-----DKLL----G----AIHD----------------

>Fusobacteria_Oceanivirga_salmonicida

---------M-SK----------------SFFTSEFVSPGHPDKICDQISDAILDECLLNDP-----NSRVACETFATTGLVVVGGEITTEG-YV--------DIQKIVRDIIKEIGYIPG----LGFDHD-CGVINTIHAQSPDISMGV-DT-G-------------------------GAGDQGIMFGGAVNETTEYMPLAITLAREIIFKLTELTKNN--T-LNWARPDAKTQVTLEY--E-N--D--K-IIGIDTI-VLSVQH-DEEVT---------LD----KIKK-DLKELVIKPV-----L---E-------K-YGYNI-SNV-KKFYINPTGRFVIGGPHGDTGLTGRKIIVDTYGGYFKHGGGAFSGKDSSKVDRSAAYMCRYIAKNLVAAGVAKKCEVQLSYAIGVIEPTSIKVDTFGTSN--IDEE----NIEKAVRKIFKLSPREIEKQFSLRK-PCF-----KYRDLAAFGHIGR-T----------------------DID---I--P-WEKL-DK-I-----EVIK----E----VLNV----------------

>Fusobacteria_Caviibacter_abscessus

-----M---M-SK----------------VYFTSEFVSPGHPDKICDQISDAILDECLKEDE-----NSRVACETFATTGLILVGGEITTKT-YV--------DIQKTTRDLIKEIGYIPG----LGFDYD-CGIINTIHSQSPDIAMGV-DT-G-------------------------GAGDQGIMFGGAVDETPNYMPLAITLAREIIIKLTNLTKDK--T-LLWARPDAKSQVTLEY--E-N--D--K-IIGIDSI-VLSVQH-SEDVT---------LE----KIKE-DLKTLVLAPT-----L---E-------K-YGYDL-AKV-KHIYINPTGRFVIGGPHGDTGLTGRKIIVDTYGGYFRHGGGAFSGKDSSKVDRSAAYMARYIAKNIVASKIAKKCEIQLSYAIGVSQPTSIKVDTFGTSN--INDE----KIIETIKKLFDLSPKGIEKTLKLRT-PNF-----RYRDLAALGHIGR-E----------------------DIK---L--P-WEEL-DK-V-----DELK----K----ELYV----------------

>Gemmatimonadete_Gemmatimonas_phototrophica

-----------MA------------DR-H-LFTSESVTEGHPDKIADQISDAVLDAILTVDP-----AARVACETLVTTGLAVIAGEITTTA-YV--------HLPEIVRQTIDGIGYNN--AE-FGFDAKTCAVMSTIDRQSPDIAQGV-DT-G-------------------------GAGDQGMMFGYATDETPELMPLPIQLAHALTHQLSVLRKDG--T-YPWLRPDGKAQVSVVY--E-G--D--R-PVAVDTV-VISTQH-DEKVS---------NT----KLRR-AILDDVIHAT-----I-P-E-------E-F-IA--RRM--KTHINPTGRFVIGGPQGDAGLTGRKIIVDTYGGMGRHGGGAFSGKDPSKVDRSACYAARWVAKNIVAAKLARRCEVQVAYAIGVAEPVSVYVQTFGTGA--VPDR----EIEAAVNAVFDLTPRGISKALDLRK-P-------IYQATAAYGHFGR-KSETIGRGKSA------------RTT---F--T-WERT-DR-V-----AALK----K----AL------------------

>Gemmatimonadetes_Gemmatimonas_aurantiaca

-----------MA------------DR-H-LFTSESVTEGHPDKIADQISDAVLDALLTEDQ-----KARVACETLVTTGLAVIAGEITTTA-YV--------HLPEIVRRTIDGIGYND--AS-FGFDSKTCAVMSTIDRQSPDIAQGV-DT-G-------------------------GAGDQGMMFGYATDETPELMPMPIQLAHALTYRLSELRKDG--T-LPWLRPDGKAQVSVVY--E-D--N--L-PVAIDTV-VISTQH-DERVS---------NT----KLRR-AILDDVIHAV-----L-P-E-------E-M-IG--RKL--KTHINPTGRFVIGGPQGDAGLTGRKIIVDTYGGMGRHGGGAFSGKDPSKVDRSACYAARWVAKNIVAAKLAKRCEVQVAYAIGVAEPVSVYVQTFGTGA--VPDR----AIEAAVQEVFDLTPRGISTALDLRK-P-------IYQATAAYGHFGR-KPETIGRGRSA------------RTT---F--T-WERT-DR-V-----TALK----K----AIR-----------------

>Lentisphaerae_Lentisphaera_araneosa

---------M-SKI-----------HTTH-IFTSESVSEGHPDKVSDQISDAILDACLEQDS-----ASRVACETLVTTDLVVIAGEITTNA-KI--------DYEAIARKVISDIGYVS--DG-IGFDSATCEVQVRVHEQSADIAQGV-DE-GAGLH-A----------------EQ-GAGDQGMMFGYASNETEALMPAPVYYSHKILEKLADVRKNN-PD-YAFLLPDSKSQVSIRY--E-N--G--K-PAAVTAV-VVSHQH-REEMD---------IA----QL-E-QIVKQVVSEV-----I-P-A-------D-L-LQ--EEI--VYHINPTGKFVIGGPHGDCGLTGRKIIVDTYGGVGSHGGGAFSGKDPSKVDRSAAYYSRYVAKNIVAAGLAEKCEIQVAYAIGVPYPISVNVDTYGTGT--LEDD---KDLEAIVSQVFSMKPADLISELKLKT-P---SNW-KYQQSAAYGHFGR--------------------------D--IF--P-WEQM-TK-V-----AELK----A----AAGV----------------

>Lentisphaerae_Victivallis_vadensis

---------M-SKI-----------HAEH-VFTSESVSEGHPDKVCDQISDAILDACLAQDP-----ESRVACETLTTTNLVVVSGEITTKA-KV--------NWQDVALNAIRKIGYND--PT-VGFCADEAKVMICVHRQSPDISQGV-TE-GQGRF-A----------------EQ-GAGDQGMMFGYASNETPDMMPATIYYAHKIVEELARLRKSDTPV-YRYLRPDSKSQVSIRY--R-H--G--K-PVAVETV-VVSHQH-TPDMP---------IE----WL-E-ALVKDVVKKV-----V-P-A-------E-M-LS--ENI--VYYVNPTGRFEVGGPHGDTGLTGRKIIVDTYGGVGSHGGGAFSGKDPSKVDRSAAYMCRYIAKNIVAAGLADKCEVQVAYAIGVADPLSINVDTYGTGK--LADD---RDLEQVIRKVFCLKPAEIVKHLGLKH-P--GAGW-CYQDTAAYGHFGR--------------------------S--QF--P-WEKT-DK-V-----AELK----A----AADLQK--------------

>Nitrospirae_Magnetoovum_chiemensis

---------M-SK------------GN-F-FFTSESVTEGHPDKVADQISDAILDELLSRDS-----IARVACETLVTTGLAFVAGEITTSA-YV--------DIPQIVRRTIKEIGYTR--AI-YGFDYETCAVITSIHEQSQDIAMGV-DP-G-------------------------GAGDQGLMFGYACDETQELMPLSIILAHKLAMELARARKED--I-LSYLRPDGKTQVTIEY--A-N--G--R-PVRIDTI-VVSTQH-SAEIS---------LK----ELKD-DVIEKIIRPI----AA-A-T-------G-I-LDE-ERI--KYHVNPTGRFVIGGPMGDTGLTGRKIIVDSYGGVGRHGGGCFSGKDPTKVDRSGTYMARYLAKNIVAAGLAERVEVQIAYAIGVPEPVSILTESFGTGK--IDPA----KLEKIIRNLFDLTPKGIIHHLNLRR-P-------IYKKTAAYGHFGR-N----------------------DDD---F--T-WEKT-DM-A-----DALR----K----EAGL----------------

>Nitrospirae_Magnetobacterium_bavaricum

---------M-PK------------KN-Y-FFTSESVTEGHPDKIADQISDAILDSIIVNDP-----YARVACETLVTTGLAFIAGEITTNC-YV--------DIPSIVRATIREIGYTR--AK-YGFDYETCSVITAIHEQSPDIAMGV-DP-G-------------------------GAGDQGLMFGYACNETQELMPMSIMLAHKLAMKLAEVRKTD--V-LPYLRPDGKTQVTIEY--R-E--G--K-PYRINTI-VVSSQH-SQNTK---------LK----DLKD-DVIEKVIKPV-----L-P-S-------D-L-LDE-ENI--IYHVNPTGRFVTGGPMGDTGLTGRKIIVDSYGGTGRHGGGCFSGKDPTKVDRSGAYMGRYIAKNIIAAGIADRVEVQIAYAIGVAQPLSILADTSGTGK--IPNE----RIVQLIRDHFDLTPKGIIEKLNLRR-P-------IYKKTAAYGHFGR-N----------------------EQD---F--T-WEHT-DM-A-----DILR----K----EAGL----------------

>Nitrospirae_Sulfobium_mesophilum

---------M-AR------------KD-Y-YFTSESVTEGHPDKIADQISDSILDAILAQDP-----IARVACETLVTTGLAFVAGEISTTC-YV--------HIPDIVRETIKNIGYTR--AK-YGFDYETCSVITSIDQQSSDIAMGV-DT-G-------------------------GAGDQGLMFGFACNETEELMPLPIMLAHKISIRLSEVRKND--I-LAYLRPDGKSQVTVEY--Q-D--G--K-PFRIDTV-VVSSQH-SPDVH---------LK----DLKD-DIIEKVVRPV-----L-P-K-------A-L-LDE-EKI--KYYINPTGRFVVGGPMGDTGLTGRKIIVDSYGGVGSHGGGCFSGKDPTKVDRSGAYISRYIAKNIVAAGLADRVEVQLAYAIGVPEPVSILVDTFGTGK--IPEE----KIVPLIRKNFDLTPKGIIEKLNLRR-P-------IYKKTAAYGHFGK-N----------------------DPD---F--T-WEAT-DK-A-----GTLK----R----EAGL----------------

>Nitrospirae_Thermodesulfovibrio_aggregans

----ML---G-DR------------KD-Y-IFTSESVTEGHPDKVADQISDAILDAMISKDP-----YARVACETLVTTGLVMVAGEITTEC-YV--------DIPSVIRETVKEIGYTR--AK-YGFDYETCAVITSIHEQSPDIAMGV-DP-G-------------------------GAGDQGLMFGFACDETEELMPMPIMLAHKLAMKLAEVRKKD--I-LTYLRPDGKTQVTVEY--R-D--G--R-PYQVRSV-VVSAQH-APDIT---------LR----ELRE-DIVEKVIKPV-----I-P-K-------E-L-LDE-ESV--QYFINPTGRFVIGGPMGDTGLTGRKIIVDTYGGIARHGGGCFSGKDPTKVDRSGSYMARYIAKNLVAAGLCKRIEIQIAYVIGIPEPVSVYINTFGTGK--IEDF----KLEEVVKKVFDLTPKGIIDKLKLRR-P-------IYKKTAAYGHFGR-P----------------------DPD---F--T-WEQT-DM-A-----DILR----K----EAELC---------------

>Nitrospirae_Nitrospira_lenta

-----------MR------------NN-Y-LFTSESVTEGHPDKIADQISDGILDAILAQDK-----YSRVACETILTTGIAFVAGEISTKA-YV--------EIPDIIREVIKDVGYND--AS-WGFDSHTCSVLTAIHQQSGDIAMGV-DS-G-------------------------GAGDQGLMFGYATNETTELMPMPIVLAHRLTKRLAEVRKKK--I-LPWVRPDGKSQVTVEY--K-N--G--K-PVRIDTI-VVSTQH-SPDVT---------NK----QIER-DIMEKVIKPV-----M-P-K-------G-L-YNP-TSV--KHHINPTGRFVVGGPMGDTGLTGRKIIVDTYGGHGSHGGGAFSGKDPTKVDRSASYMARYIAKNIVAAGLADKCEVQLAYAIGVADPVSVLVDTKGTEK--MAVE----NLDKLVRKHFPLTPRGIIDHLKLRR-P-------IFRKTAAYGHFGR-N----------------------EPE---F--T-WEKT-DK-A-----KALR----R----DAGL----------------

>Nitrospirae_Leptospirillum_ferrooxidans

---------M-AR------------AP-Y-LFTSESVTEGHPDKLCDQVSDGILDAILSQDP-----MARVACETLTTTGIVIVAGEITARH-NT--------QYEDIVRETVKEIGYTD--AS-FGFDYKTCSVLTAVHSQSPDISMGV-DT-G-------------------------GAGDQGLMFGMAVNESEELMPMPILLAHRLTRQLSNVRKTG--L-LPYLRPDGKAQVSLRY--D-G--F--K-PVGVETI-VVSTQH-APEVT---------QE----EIRR-DIIEKVIRPV-----I-P-K-------N-L-LDE-KTV--TFHINPTGRFVTGGPMGDAGLTGRKIIVDTYGGWGRHGGGAFSGKDPTKVDRSACYMARYIAKNIVGAGLASRCEVQLAYAIGVADPVSIHVETQGTST--IPDD----RIAEIVAEIFPLTPKGIIDHLKLRR-P-------IYKKTASYGHFGR-L----------------------EED---F--T-WEKL-DQ-V-----DNLR----R----EAARKG--------------

>Planctomycetes_Scalindua_brodae

---------M-KK------------GN-Y-LFTSESVSMGHPDKIADQISDAILDAILAQDP-----MSRVACETLVTTGMAMVAGEITTTA-VI--------DIQHIVRQTIKDIGYTD--SS-MGFDYETCAVMSTLGKQSPDIAQGV-SE-GEGLH-S----------------EH-GAGDQGMMFGFACNETAELMPLPICMAHHLVKKLSEERQTG--D-LFYLRPDGKSQVTVEY--D-G--S--K-PLRVDAV-VISSQH-APDIS---------HD----QLEN-DIIEKVVKKV-----I-P-V-------D-M-LD--DKT--KYYINPTGRFVVGGPQGDCGLTGRKIIVDTYGGRARHGGGAFSGKDPSKVDRSAAYAARYVAKNVVAAGLADICEVQLSYAIGVAKPMAVLILTENTAK--IPEA----KIEELVKKHFDLTPKGIIDHLQLRR-P-------IYQVTARHGHFGR-S----------------------EDS---F--S-WEKT-DK-A-----EILR----K----DAGL----------------

>Planctomycetes_Blastopirellula_cremea

---------M-AS------------GK-Y-LFTSESVSMGHPDKMADQISDGILDALLAEDP-----HSRVACETMVTTGVAIVAGEITTKA-RI--------DYQDVIRQVIRDIGYTS--DD-MGFNADTCAVMVTLDRQSPDIAQGV-ND-DSAKG-K----------------EI-GAGDQGLMFGYACNHTPELMPLPVALSHRILNRLTEARQNG--E-VDWLRPDSKSQVTVEF--D-G--D--R-PVGIHTV-VVSTQH-SDKVD---------NA----TIKE-FVIEKVIKPV-----L-P-E-------E-F-LD--DDI--IFHINPTGNFVVGGPMGDCGLTGRKIIVDTYGGWGRHGGGAFSGKDSTKVDRSAAYMGRYVAKNIVAAGLADRCEVQLAYAIGVTEPVSVHIDTFGTGK--IDDE----KIADLVVKNFPLSPGGIIDYLDLRR-P-------IFRATAAGGHFGR-D------------------------E---F--P-WENT-DK-A-----EELA----K----QAGITV-SA-----------

>Planctomycetes_Fimbriiglobus_ruber

---------M-SA------------DR-Y-LFTSESVSMGHPDKVADQISDAILDFCLSHDR-----MSRVACETLVTTDHCTVAGEITTKAPLTRV------NVDKLVRDAITEIGYTD--PR-IGFAAETCQVNCLIHAQSPDISVGV-DT-G-------------------------GAGDQGMMFGFACDETRTLMPLPIYLSHRLVENHAAMRRDG--R-LKWLRPDAKSQVTVEYN-A-D--G--S-PHRITTV-VLSTQH-DETVV---------LEADKKRMTD-AARKEIVEKL-----VLP-TLQAE-RAD-L-LK--GDI--TYHINPTGIFLEGGPHGDCGLTGRKIIVDTYGGRGRHGGGAFSGKDPTKVDRSAAYMARYIAKNIVAARLAKQCEVQLSYAIGYPDPLSVWVSTFGTVAPGVTEH----KLIGLIRDHFKLTPKGIIETLDLRR-P-------IYRETARHGHFGR-E----------------------RPE---F--T-WEKT-DK-A-----EALR----K----AAGV----------------

>Planctomycetes_Brocadia_caroliniensis

---------M-KK------------GR-H-LFTSESVSMGHPDKVADQISDAVLDAMLEQDP-----MSRVACETLVTTGVAFVAGEFTTKA-NV--------NIPDIVRHTIKEIGYGD--AS-IGFDYNTCAVITSIGKQSPDISQGV-SE-GEGLH-K----------------EQ-GAGDQGMMFGYACNDTPELMPLPIMLAHRIIIKHAELRQGG--K-LKYLRPDAKSQVTVEY--E-D--H--K-PIRVHTV-VVSTQH-APDVK---------YE----TIKE-DMIEKVVKQV-----V-P-A-------N-L-LD--SKT--IYHINPTGRFVIGGPQGDCGLTGRKIIVDTYGGWGRHGGGAFSGKDPTKVDRSACYAARHVAKNVVASGLADRCEAQVAYAIGVAKPLALHITTEGTGK--IPDE----KLTQICETVFDMTPRGIIDRLKLRR-P-------IYKLTARHGHFGR-S----------------------EDT---F--T-WEKT-DM-V-----DALR----K----AAGV----------------

>Planctomycetes_Roseimaritima_ulvae

-----M---S-EK------------QR-Y-LFTSESVSMGHPDKLADRISDGILDALLSQDP-----HSRVACETMVTTGVAIVAGEISSKA-QV--------NYSQIVRDVINDVGYTD--DQ-MGICGDTCAVMVSLDAQSPDIAQGV----DAGEN-K----------------DV-GAGDQGLMFGFACKDTPELMPLPIALSHRIINRITEARKNN--E-VSWLRPDSKAQVTVEY--D-G--F--T-PVRVDTV-VVSTQH-GPEVD---------NE----TIRE-FVINKVIKPS-----L-P-A-------E-L-VQ--TEP--TYHINPTGKFVIGGPHGDCGLTGRKIIVDTYGGWGRHGGGAFSGKDSTKVDRSAAYMARYIAKNIVAAGLAERCEVQLAYAIGVSEPVSVHVDTQGSGT--IDDS----RLCELVREHFPLTPAGIIDHLQLRR-P-------IFQATSAGGHFGR-S----------------------EPE---F--S-WEQT-DK-A-----DALA----E----AAGKAA-QA-V---------

>Planctomycetes_Mariniblastus_fucicola

---------M-AS------------GK-Y-LFTSESVSMGHPDKLADQISDAILDAMLEQDP-----ASRVACETMVSTEMAVIAGEITSKA-DV--------DIEKVVRDVIRDVGYTD--AS-IGIGCDSCQVQVNLDQQSPDIAMGV-DA-D-------------------------GAGDQGLMFGFACDDTEELMPLPIALSHKILDRLNKARFDK--E-VDWLRPDSKSQVTVEY--D-G--F--K-PVRIDTV-VVSTQH-DPGVD---------NE----TIQK-FVREQIVEPL-----L-P-A-------E-L-NK--GDI--KYHINPTGNFVIGGPHGDCGLTGRKIIVDTYGGWGRHGGGAFSGKDSTKVDRSAAYMARHVAKNIVASGLASRCEVQLAYAIGVADPVSVHVDTQGTGS--IPDE----KICEIVTEQYPLSTQGIIKYLELRR-P-------IFRKTAAGGHFGR-S----------------------DAD---F--S-WENT-SS-A-----KALA----D----KASALV--------------

>Planctomycetes_Isosphaera_pallida

---MSS---S-AA------------DR-F-LFTSESVSMGHPDKMADQISDGVLDAILSQDA-----TARVACETLLTTGLICLAGEITTTA-II--------DYPAVVRQIVKEIGYVS--SD-MGFDATTCAVMVALGKQSPDIALGV-DR-D-------------------------GAGDQGLMFGFACDETDELMPLPIALSHRIINHLTEVRQAH--K-VDWLRPDSKSQVTIEY--D-G--L--K-PVRIDTV-VVSTQH-APHVD---------QK----TIRE-FVIDEVIKPV-----L-P-T-------S-L-IN--GAI--TYHINPTGNFVVGGPHGDAGLTGRKIIVDTYGGYARHGGGAFSGKDPTKVDRSAAYMARYVAKNIVAAHLANRCEIQLAYAIGVAEPVSIHVNTYGTGK--LPDS----KLAEIVRRVFPLTPRGIIKHLDLRR-P-------IYRKTASGGHFGR-N----------------------DPD---F--T-WERV-DQ-V-----EALR----Q----AAGV----------------

>Planctomycetes_Singulisphaera_acidiphila

-----M---P-QQ------------DR-Y-LFTSESVSMGHPDKMADQISDGILDAIFKQDP-----YARVACETLLTTGLVVVAGEITTTA-SV--------DYASVVRQVVEEVGYTS--SE-MGFDAKTCAVMVALGKQSPDIAQGV-ND-DAEKG-K----------------DI-GAGDQGLMFGYAANETPELMPMPIALSHRIINRITELRKNG--T-IKWLRPDSKSQVTVEY--V-D--G--K-PVRIDAV-VVSTQ--TDETP---------IE----EIRK-VIKEQVILPV-----L-P-K-------D-M-VD--AET--KYYINPTGRFVVGGPCGDAGVTGRKIIVDTYGGAGRHGGGAFSGKDPTKVDRSAAYMARYVAKNVVAAGLADRCEIQFAYAIGVSEPVSVHVDTFGTGQ--IADA----QIADLVRKTFPLTPSGIIKHLDLRR-P-------IYQKTASGGHFGR-S----------------------EPE---F--T-WERT-DK-A-----DALR----Q----ATSALA-TA-----------

>Planctomycetes_Paludisphaera_borealis

-----M---S-QH------------DR-Y-LFTSESVSMGHPDKMADQISDSILDAILEQDP-----YGRVACETLLTTGLVCLAGEITTKA-MI--------DYPSIVRQVVRDIGFTS--SD-MGFDGTTCAVMVALGKQSPDIAQGV-DE-NSDKG-K----------------DI-GAGDQGMMFGFACNETPELMPLPIALAHRIINKITEVRQNG--Q-IPWLRPDAKSQVTIEY--D-G--F--T-PVRVNTV-VVSTQH-SPDVS---------HE----ELCR-RIRKEVIDPV-----I-P-A-------H-L-VS--GDI--VCHINPTGKFVIGGPHGDAGVTGRKIIVDTYGGMGRHGGGAFSGKDATKVDRSAAYMARYVAKNVVAAGLAERCEIQLAYAIGVSEPVSIHVDTFGTGQ--LPDA----AIAEIVRKGFPLTPAGIIRHLDLRR-P-------IFRKTASGGHFGR-S----------------------EPE---F--T-WEKT-DR-A-----TLLR----D----AANQIARTA-TLV-------

>Planctomycetes_Gimesia_maris

-----------MA-------------N-F-SFTSESVSMGHPDKVSDQVSDGILDALLAQDP-----YSRVACETLCTTDFVVLAGEITSNA-QV--------DYEKVARDVIRDIGYTS--KD-IGFNADTCEVLVKLHQQSADIAQGV-DA-E-------------------------GAGDQGLMFGYACNQTEEYMPVPIALSHRILNKLTEIRQNG--E-VDWLLPDSKSQVTVDY--E-D--G--K-PVGVSAV-VVSTQH-TEEVS---------QE----EIHR-FVTEKVVKEV-----I-P-A-------D-F-LS--DKT--KYHINPTGRFVIGGPHGDTGLTGRKIIVDTYGGWGRHGGGAFSGKDSTKVDRSAAYMARYIAKNIVAAGLAAECEVQLSYAIGVVEPTSIYVDTKGTSV--IPEA----KISELVGELFPLSPQGIINHLQLRR-P-------IFRKTTWGGHFGR-N----------------------DPD---F--T-WEAT-DK-A-----AELR----D----AAGLGN-EV-PETQFAMS--

>Planctomycetes_Rubinisphaera_brasiliensis

-----------MS-------------N-Y-IFTSEAVSMGHPDKVSDQVSDGVLDALLAQDP-----AARVACETLCTTDFVCLAGEITANA-DV--------DYEAITREVIADIGYTS--DD-IGFNAATCEVDVRLHSQSQDIAMGV-DR-D-------------------------GAGDQGLMFGYACNQTEEFMPVPIALSHRILNRLTEARQNG--E-VNWLRPDSKSQVSVEF--A-G--N--R-PVGISAV-VVSTQH-TPSVK---------QD----EISS-FITEKIVKDV-----V-P-H-------D-F-LT--DKT--KFHINPTGQFIIGGPHGDCGLTGRKIIVDTYGGWGRHGGGAFSGKDSTKVDRSAAYMARYVAKNIVAAGLADECEVQLAYAIGVAEPVSVHIDTNGTGK--VEGE----KIAAAVREVFPLTPKGIIETLELRR-P-------IFRKTAWGGHFGR-S----------------------DAD---F--T-WEAI-NK-A-----GDLR----K----ALGMDA-EL-PKTEFAMS--

>Planctomycetes_Planctomicrobium_piriforme

-----------MS------------RS-F-IFTSESVSSGHPDKVSDQVSDGVLDALLAQDP-----TSRVACETLCTTDFVCVAGELRTNA-KV--------DFEKVVRDAIRDIGYTS--DD-IGFNADTCEVLNKLHSQSADIAMGV-DA-D-------------------------GAGDQGLMFGFACNQTPELMPLPIALSHRILNLLTKLRHEK--D-VDWLRPDAKSQVSIKY--E-N--G--K-PVGITAI-VLSTQH-SEKVN---------QK----QIED-YIRSKVIPAT-----I-P-S-------E-L-LT--KET--KYHVNPTGQFIIGGPHGDTGLTGRKIIVDTYGGWGRHGGGAFSGKDSTKVDRSAAYMARYVAKNIVAAGLADECEVQLAYAIGVADPVSINVDSHGTAK--VAET----KIESAVRDLFPLTPKGIIEHLKLRR-P-------IFRKTASGGHFGR-D----------------------DKD---F--T-WEHT-DK-A-----EALK----K----AVK-----------------

>Planctomycetes_Schlesneria_paludicola

-----------MT-------------N-Y-LFTSESVSMGHPDKVADQVSDGVLDAILAQDP-----RSRVACETLCTTDLVVLSGEITTQA-KL--------DYVQVARDVIKDIGYIS--DD-IGFSYESCKVFVALHSQSPDIAMGV-DR-E-------------------------GAGDQGLMFGYACDQTPELMPLPIALSHRIINRLTEARQKK--E-VDWLRPDAKSQVTVEYD-S-N--N--K-PVGISAV-VVSTQH-HEKVS---------QD----DITN-FVIADVIKQS-----V-P-A-------E-L-LT--KNT--KYHINPTGRFVVGGPHGDTGLTGRKIIVDTYGGWGRHGGGAFSGKDPTKVDRSAAYMARHVAKNIVAAGLARECEVQLAYAIGVAEPVSVHVNTNGTNA--IPDE----KIVALVREHFKMTPLAIIDYLKLRR-P-------VYRKTASGGHFGR-S----------------------DAD---F--T-WEAT-NK-A-----ADLK----K----AAGL----------------

>Planctomycetes_Planctopirus_limnophila

-----------MS-------------T-F-LFTSESVSMGHPDKVSDQVSDAVLDALLAQDP-----RARVACETLCTTDFVCLAGEITANA-QV--------DYEKVAREAIRQIGYTS--DD-IGFNAATCEVLVKLHSQSADIAMGV-DR-D-------------------------GAGDQGLMFGYACDQTPELMPLPIALSHRIINRLTEARQRG--E-VNWLRPDSKSQVTVEYD-A-K--N--N-AVGISAI-VVSTQH-AEHVS---------QD----EISE-FVRSAVIGKS-----V-P-T-------S-L-LK--PST--KYHINPTGRFVIGGPHGDSGLTGRKIIVDTYGGWGRHGGGAFSGKDPTKVDRSAAYMARYIAKNIVAAGLAKECELQLAYAIGVADPVSVRINTNGTAL--IAEG----KIAELVRENFKMTPLGIIESLQLRQ-P-------IYQRTAAGGHFGR-N----------------------EPG---F--T-WEAT-DK-A-----LKLR----E----QAGLGS-TL-PPVGHIA---

>Planctomycetes_Fuerstia_marisgermanicae

-----------MA-------------N-Y-FFTSESVSMGHPDKVSDQVSDGILDALLAQDP-----MSRVACETLCTTDLVVLSGEITTSA-EV--------DYVEVARQVIREIGYTS--DD-IGFNADTCKVLNYLHSQSADIAMGV-DR-D-------------------------GAGDQGLMFGYACDQTPELMPVAIAFSHRIINLLAEKRQKG--E-VNWLRPDSKSQVTVEY--D-G--A--K-PVRIDAI-VVSTQH-SADVS---------QA----EISE-YVK-KVIKEA-----I-P-N-------D-L-LD--DKT--KYHINPTGNFVIGGPHGDTGLTGRKIIVDTYGGWGRHGGGAFSGKDSTKVDRSAAYMGRYVAKNIVAAGLATECEVQLAYAIGVADPVSVRVDTNGTAT--VPEE----KIQKAVQEIFPLNPKGIIDHLGLRK-P-------IFRHTAHGGHFGR-T----------------------EEE---F--K-WEAT-DM-V-----DALK----A----AVS-----------------

>Spirochaetes_Treponema_putidum

---------M-KN------------NI-S-YFTSESVSEGHPDKLCDQISDAVLDACLRDDP-----ESHVACETFASTALVLVGGEITTDT-YV--------DIQDIARTIAEEIGYTN--TD-FGLDCHSMAVMNMIHSQSPDISQGV--D-GTGLD-EYKG-------------QQ-GAGDQGMMFGFACKETPELMPAPIMFSHSVLRYAAKLRKEK--V-IPWLRPDSKTQITVKY--E----G-FK-PIKIDTV-VLSHQH-YPDVQ---------YD----ELKH-TLINQVIKPV-----LGP-T-------G-L-LA--DDT--KYFINPTGRFVIGGPFGDTGLTGRKIIVDTYGGMGRHGGGAFSGKDPSKVDRSAAYMARYIAKNVVAADLARRCELQLAYAIGVPFPVAVRVDTFGTGE--VPEE----KIEKAIKEVFDMSPAGIIKALDLKR-P-------IYKETAAYGHFGR--------------------------P--EF--S-WEKT-DK-T-----EALK----K----AIK-----------------

>Spirochaetes_Marispirochaeta_aestuarii

---------M-KQ------------RN-Y-LFTSESVSEGHPDKLADQVSDAVLDACLAGDP-----ESRVACETFTTTGMVLVGGEITTNT-YV--------DIQEIARGVAKSIGYDK--PE-YGLDFESMAVMSTIHSQSPDISQGV-SV-GHGLK-EYEG-------------KQ-GAGDQGMMFGFACRENDELMPAPITLAHKLLMHAAKVRKTG--K-ITWLRPDSKSQVTVEY--E----G-HT-PKRIDTV-VVSHQH-DEGIG---------YE----ELKA-EIIAKIIKPV-----LEP-T-------G-L-LA--ENT--RYFINPTGRFVIGGPHGDSGLTGRKIIVDTYGGMGRHGGGAFSGKDPSKVDRSAAYMARYAAKNIVAAGLCERCELQLSYAIGVPEPVSIMVEAFGTEN--VAIE----RIEKAAREVFDFSPSGIIRTLDLMR-P-------IYQETAAYGHFGR--------------------------D--SF--S-WEKT-DR-V-----DDLK----T----ACGV----------------

>Spirochaetes_Sediminispirochaeta_smaragdinae

-----M---D-TK------------RK-V-LFTSESVSEGHPDKVCDQISDAVLDACLREDS-----TSRVACETFTTTGMVLVGGEISTST-YV--------NLQDVVRGVVKRIGYDR--PE-YGLDWESMAVIDTIHNQSSDISMGV-TA-GE--N-KYK--------------SQ-GAGDQGMMFGFACNETEELMPAPIIFAHRLLQKAAAVRKSG--E-CSWMRPDAKSQVTVQY--E----G-GK-PVRIDTI-VISHQH-SPSVS---------IK----EIEA-SLRKRVIEPV-----LGD-T-------G-L-ID--EKT--RFFINPTGRFVIGGPHGDSGLTGRKIIVDTYGGMGRHGGGAFSGKDPSKVDRSAAYMARYVAKNIVAAGLCDRCELELSYAIGVPFPISVMVDTFGTAT--VPEE----RIEAAVEKVFDLSPEGIIRELGLLA-P-------IYEATAAYGHFGR--------------------------D--GF--S-WERV-DK-T-----EELK----K----AVG-----------------

>Spirochaetes_Salinispira_pacifica

----MA---Q-HK------------EK-Y-VFTSESVSEGHPDKLADQVSDAVLDACLSDDP-----GAHVACETYTTTGMVLIGGEITTDT-YV--------DIQQIARDVVRDIGYTH--AD-YGLDWESMSVLNVIHSQSPDIAQGV--N-GSGL---YSG-------------EQ-GAGDQGMMFGFACNETGELMPAPVSFAHRILRRAAELRKSG--D-IPWLRPDSKSQVSIKY--D----G-FT-PVGVDSV-VVSHQH-DPDVE---------RS----DLEE-CIREEIIRPV-----LEP-T-------G-L-LT--PET--DFFINPTGRFVIGGPHGDAGLTGRKIIVDTYGGMGRHGGGAFSGKDPSKVDRSAAYMARYVAKNIVAAGLADRAEVQFAYAIGVPFPVSVMVNSFGTAE--VEDY----QIAEAVKQVFDLSPKGIISELDLLK-P-------IYRSTTNYGHFGR--------------------------D--GF--S-WEKT-DR-A-----SALK----A----AL------------------

>Spirochaetes_Spirochaeta_thermophila

---------M-SL------------RR-Y-LFSSESVSEGHPDKIADQVSDAVLDACLAQDP-----ESRVACEVFTTTGMVLVGGEIASQA-RI--------DVQELVREVVKEIGYDR--AE-YGLDYRSMSVVNVLQKQSPDIAQGV-LE-GIGL---FKE-------------RI-GAGDQGMMFGFACDETPELMPMPIMCAHRLLQHAARLRKEG--V-IPWLRPDAKSQVTVEY--E----G-WT-PKRITAV-VLSHQH-DDGLE---------YE----EIRT-VLIDQVIRPV-----LEP-T-------G-L-LD--EST--VYYINPTGRFVIGGPHGDTGLTGRKLMVDTYGGMARHGGGAFSGKDPTKVDRSAAYMARYIAKNIVAAGLAKRCEIQVSYAIGVPRPVSIMVDTFGTGE--VSDR----ALEEAIDGVFDLSPEGIIRTLDLQR-P-------LYRATAVYGHFGR--------------------------E--FF--P-WERV-DK-V-----EEIR----R----AVGM----------------

>Spirochaetes_Treponema_pallidum

-------------------------ME-T-FFTSESVSEGHPDKLCDQISDAVLDACLSQDP-----HSCVACETFASTSLILIGGEISTRA-HI--------NLTQIARDVAADIGYVS--AD-VGLDAASMAVLDMTHHQSPDIAQGV--H-GAGLK-EFAG-------------SQ-GAGDQGIMFGFACRETPEFMPAPLMCAHAVVRYAATLRHER--R-VPWLRPDAKSQVTVQY--E----G-HR-PVRISAV-VFSQQH-DPSPS---------YE----TIRE-TLIEEIVRPA-----LAP-T-------G-L-LD--ENT--RFFINPTGRFVIGGPFGDTGLTGRKIIVDTYGGMGRHGGGSFSGKDASKVDRSAAYMARYIAKNIVAADLAERCEVQLAYAIGVPYPVSLRIETFGTAR--ASES----HITHAVKEIFDLTPAGIVRTLDLCA-P-------RYRSTAVYGHFGR--------------------------E--QF--P-WERT-DC-V-----CDLQ----R----AVRPFALSGQIKE-------

>Spirochaetes_Leptospira_elllisii

---------M-SL-------------KDF-IFTSESVGEGHPDKVCDQISDAVLDAYLEQDP-----KSRVACETLVTTNLVVIAGEITSKG-KV--------DAQEIARNVIREIGYND--IT-MGFDADFAVVSAHVHAQSPDISQGV-TE-GEGLF-K----------------EQ-GAGDQGLMFGFAINETPELMPMPIYYSHELVKHLAGLRHGN--K-LKFLRPDAKSQVTVEY--K-D--G--K-PVRIDTV-VISTQH-SPDVT---------HK----QIEE-SVIEECVKKV-----I-P-A-------N-L-LV---NT--KYFINPTGQFIVGGPHGDAGLTGRKIIVDTYGGYGRHGGGAFSGKDPSKVDRSAAYMGRYIAKNVVASGLADKCEVQLAYAIGVAEPVSVHVDTFGTGK--VSEE----ELVKRIRANFKLTPRGIIESLKLLE-----KGR-KYRETASYGHFGR------------------------KGS--TF--T-WEET-DK-A-----AALK----G------------------------

>Spirochaetes_Leptospira_borgpetersenii

---------M-SL-------------KDF-IFTSESVGEGHPDKVCDQISDAILDAYLEQDP-----KSRVACETLATTNLVVIAGEITSKG-KV--------DAQEIARNVIRDIGYND--IT-MGFDADFAVVSAHVHAQSPDISQGV-TE-GEGLF-K----------------EQ-GAGDQGLMFGFAINETPEFMPMPIYYSHELVKHLAGLRHSN--K-LKFLRPDAKSQVTVEY--K-D--G--K-PVRVDTV-VISTQH-SPDVA---------HK----QIEE-SLIEECIKKV-----I-P-A-------N-L-LV---NT--KYFINPTGQFIVGGPHGDAGLTGRKIIVDTYGGYGRHGGGAFSGKDPSKVDRSAAYMGRYIAKNVVASGLADKCEVQLAYAIGVAEPVSVHVDTFGTGK--ISEE----ELVKRIRANFKLTPRGIIESLKLLE-----KGR-KYRETASYGHFGR------------------------KGS--TF--T-WEKT-DK-T-----VALK----G------------------------

>Spirochaetes_Brachyspira_suanatina

---------M-AEI-----------KN-Y-YFSSESVTEGHPDKICDAVSDAVLDECLKQDP-----NSRVACETLAKTGMIMIAGEITTKA-KL--------DYQKIARDTVRRIGYTS--SD-MGFDADTCAVMESIAEQSPDIDMGV-SA-GKGLF-N--S-ESSNV-------SE-GAGDQGIMFGYAINETETFMPLTIHLAHRLAERLTKIRKDK--V-VDYLRPDGKSQVTVEY--K-D--G--K-AARIEAV-VISTQH-AAGVD---------HK----QIEA-DIKKYVINEI-----C-P-A-------N-M-LD--ENT--KYYINPTGSFVVGGPMGDCGLTGRKIIVDSYGGHGAHGGGAFSGKDPTKVDRSACYMARYVAKNIVASGIADRALVQFAYAIGVPEPLSVYVNTFGTAK--VHDE----VLANIVAKELDLTPAGIIKRLDLRR-P-------IYEKTTAYGHFGR------------------------ELP--EF--T-WEKT-D----------IKD-LFA----NAK-----------------

>Spirochaetes_Brachyspira_hyodysenteriae

---------M-AEI-----------KN-Y-YFSSESVTEGHPDKICDAVSDAVLDECLKQDP-----NSRVACETLAKTGMIMIAGEITTKA-KL--------DYQKIARDTVRRIGYTS--SD-MGFDADTCAVMESIAEQSPDIDMGV-SA-GKGLF-N--S-ESSNV-------SE-GAGDQGIMFGYAINETETFMPLTIHLAHRLAERLTKVRKDK--V-VDYLRPDGKSQVTVEY--K-D--G--K-AARIEAV-VISTQH-AAGVD---------HK----QIEA-DIKKYVINEI-----C-P-A-------N-M-LD--SNT--KYYINPTGSFVVGGPMGDCGLTGRKIIVDSYGGHGAHGGGAFSGKDPTKVDRSACYMARYVAKNIVASGIADRALVQFAYAIGVPEPLSVYVNTFGTAK--VHDE----VLANIVSKELDLTPAGIIKRLDLRR-P-------IYEKTTAYGHFGR------------------------ELP---F--T-WEKT-D----------IKD-LFA----NAK-----------------

>Spirochaetes_Brevinema_andersonii

---------M-KP----------------ILFTSEYVSPGHPDKIADQISDAVLDACLAQDK-----NARVACETFCTTGQVVVGGEITTNA-YV--------DIQSLVRNKIEEIGYTQD----MGFDKN-CGIFNAIHSQSPDIAMGV-DT-G-------------------------GAGDQGIMFGGAVRETPEFMPLALVLAREIIFTLTQLTRSK--Q-LSWARPDAKSQITLSYD-E-N--G--K-IINVDTV-VVSVQH-SPEVS---------QE----KIQQ-DLKEFVIKPV-----L---Q-------K-YNLNI-DDV-KKFHINPTGRFVIGGPHGDTGLTGRKIIVDTYGGYFRHGGGAFSGKDPSKVDRSAAYAARWVAKNIVAAGLADKCEVQISYAIGVPHPTSIKIDTFNTSN--IDER----ILAQAVLKTFDLSPQGIEKALELRE-GTF-----KYQDLAAFGHIGR-T----------------------DID---L--P-WERI-NK-I-----NKLK----E----NLSI----------------

>Spirochaetes_Borrelia_hermsii

---------M-LN------------NN-L-TSTSEAVSEGHPDKIADQISDAILDEILKRDK-----MAKVACETLVSQNLVVIAGEINSKA---------KKDID--IKQTIKNIGYTN--IE-YGLDYKSATIIDAIGSQSIDITNAV-DK-------K----DTK---------DI-GAGDQGIIFGYACNETENFLPIAYELSNLILQKASELRKSG--E-IKWLRPDAKSQVTIEY----D-SN--KNPIRINNI-VVSHQH-DPDIP---------QD----FLRQ-TIIEKIIKPT-----L-Q-S-------KSM-LD--ENI--KYYINPSGNFVIGGPTGDTGLTGRKIIADSYGGFARHGGGAYSGKDATKVDRSAAYISRYIAKNMVAAGISKEFEMQLAYAIGIPNPVSIKITT------GINDHEYEKKILSFIINNFDLTPNGIIKKLKLRE-P-------IYSKTCIYGHFGK-N----------------------ELE--------WEKL-DF-V-----DIIK----K----EFKI----------------

>Spirochaetes_Dethiosulfovibrio_salsuginis

---------M-A-------------EK-I-LISSESVTEGHPDKVADQISDGVLDAILAEDP-----MGRVACETLVTTGLIQVAGEITTST-YV--------DIPRIAREIVKEIGYTR--AK-YGFDGETCAVLTAIDEQSSDIALGV-DK-ALELK-E--G-DMS-D--EQID-RI-GAGDQGMMIGYATDETEEFLPMPFALAQRLSRRLTKIRKEG--I-LDYVRPDGKTQVTLEY--V-D--G--V-AVRVDTV-VVSTQH-GPDVS---------LR----EIRE-DITEQVIDPI-----L-P-K-------D-L-IK--GDL--RILVNPTGRFVMGGPMADSGLTGRKIIVDTYGGMVPHGGGAFSGKDPTKVDRSGAYMARYAAKNVVAAGLASRCQIQVAYAIGVARPVSIFVETFGTGK--VSDD----LLTALVRKNFDFRPAAMIRDLELRK-P-------QYRRIAAYGHMGR-I----------------------DLN--PM--PAWERL-DR-V-----EALK----S----GSGV----------------

>Spirochaetes_Alkalispirochaeta_odontotermitis

---------M-HTY-----------GS-K-LFTSESVSEGHPDKVCDIISDAVLDRALTLDS-----KSRVACECYTSTGLVLVGGEISTEG-YI--------DIIQTARDVLGEIGYTN--PA-DGIDNLSCSILSVIRPQSSDISQGV-NV-GEGLH-K----------------EL-GAGDQGFMFGYACRQTDEMMPAPIQYAHQIMKKAAEVRKSG--T-LSFLRPDAKCQVTVKYR---D----DE-PVHIDTV-VLSHQH-TETVS---------HA----TVSE-SLIESVIRPA-----L-P---------A-ELFN--KQT--AIHINPTGRFVVGGPNADTGLTGRKIIVDTYGGYSRHGGGSFSGKDPTKVDRSAAYMARYMAKNLVAANIVDEVEIQIAYAIGMADPISLFVNTFQTRQ--LD-D----IIAKKLYDIFDLTPAGIIQTLDLQR-P-------IYKKTAAYGHFGRSE----------------------------F--P-WEKV-DK-V-----EIIQ----KMMS---------------------

>Synergistetes_Dethiosulfovibrio_peptidovorans

---------M-S-------------EK-L-LISSESVTEGHPDKVADQISDGILDAILSEDP-----MGRVACETLVTTGLVQVAGEITTST-YV--------DIPRIAREIVKEIGYTR--AK-YGFDGETCAVLTAIDEQSADIAMGV-DR-ALELR-E--G-DMT-E--EQIN-GI-GAGDQGMMIGYASDETDEFLPMPFALAQRLSRRLSKVRKDG--T-IDYLRPDGKTQVTLEY--D-G--D--R-AVRVDTV-VVSAQH-SPDVS---------LR----DIRE-DLTEKVIDPI-----L-P-R-------E-L-LK--GDM--RILVNPTGRFVKGGPMADSGLTGRKIIVDTYGGMVPHGGGAFSGKDPTKVDRSGAYMARYAAKNVVAAGLASKCQIQVAYAIGVAHPVSIFVETFGTGK--VSND----ILVSLVRKYFDFRPAAMIRDLELRK-P-------QYRRLAAYGHMGR-I----------------------DLT--PV--PAWERL-DR-A-----AALK----T----DAGVQ---------------

>Synergistetes_Pyramidobacter_piscolens

---------M-A-------------EK-I-LISSESVTEGHPDKLADQISDGVLDAILAQDP-----MSRVACETLVATGAIIVAGEITTKA-YV--------DIPGIARKAVLDVGYNR--AK-FGFDGETCAVFTQIDEQSGDIAMGV-DR-AMEIR-E--S-EMD-D--DQID-RI-GAGDQGMMIGYATDETPEFLPMPFALAQRLARRLTAVRKDG--T-LNCLRPDGKTQVTLEY--R-D--G--V-AVAADTI-VVSSQH-SPMVS---------LE----ELRD-LIARNVIEPI-----M-P-K-------E-L-MK---NP--RILVNPTGRFVLGGPMADTGLTGRKIIVDTYGGMVPHGGGAFSGKDPTKVDRSGAYMARYAAKNVVAAGLARRCQIQVAYAIGVARPVSVHVETFGTSK--LNEE----QLTELIRANFDFRPAAIIRDLELRT-P-------QYRRLAAYGHMGR-I----------------------DLE--PL--PCWEKL-DR-V-----DALR----R------------------------

>Synergistetes_Synergistes_jonesii

---------M-SK------------ER-F-LISSESVTEGHPDKLADQISDAVLDAILESDP-----NGRVACETLVSTGLILVAGEISTTC-YV--------DIPKIARKTVKDVGYTR--AK-YGFDGDTCSVLTTIDEQSPDIALGV-DR-AKETK-E-----LS-N--DEVD-AI-GAGDQGLTVGYACDETEELMPMPISLAHKLTRRLAEVRRNK--T-LPYLRPDGKSQVTVEY--E-N--G--K-PLRVDTV-VISTQH-HPAID---------QK----QIEA-DVIEHVVKPV-----I-P-A-------R-L-IT--TKP--RIFVNPTGRFVMGGPQADTGLTGRKIIVDTYGGAVPHGGGAFSGKDPTKVDRSGAYMARYAAKNIVAAGIAEMCQVQAAYAIGVARPVSIMVETFGTGK--IRDE----DITCLLRENFDFRPAAIIRDLDLRK-P-------QYKRLAAYGHMGR-V----------------------DLD--PM--PSWERT-DK-A-----EILK----H----AAERFA--------------

>Synergistetes_Cloacibacillus_porcorum

---------M-SK------------ER-F-LISSESVTEGHPDKLADQISDAVLDAILEADP-----MGRVACETLVSTGLIVVAGEISTVC-YV--------DIPKIARKTVKDVGYTR--AK-YGFDGDTCSVITTIDEQSPDIALGV-DK-AKEAK-E-----LS-E--DEID--I-GAGDQGLMVGYACNETEELMPLPISLAQKLTRRLSEVRKNK--T-LPYLRPDGKSQVTVEY--V-N--G--K-PLRVDTV-VISTQH-HPAID---------QK----QIEA-DIIEHVIKPV-----I-P-S-------Q-L-IT--TKP--RILVNPTGRFVMGGPQADSGLTGRKIIVDTYGGAVPHGGGAFSGKDPTKVDRSGAYMARYAAKNVVAAGLADACQIQVAYAIGVAKPVSIMVETFGTGK--IKDE----EITQLLRENFDFRPAAIIRDLDLRK-P-------QYRRLAAYGHMGR-I----------------------DLD--PM--PAWERT-DK-A-----ETLK----R----AAERFA--------------

>Synergistetes_Acetomicrobium_thermoterrenum

---------M-PK------------ER-L-LLTSESVTEGHPDKLADQISDGILDAILAQDP-----MGRVACETLVTTGLVMVAGEITTSC-YV--------DIPRLVRSIVKDIGYTR--AK-YGFDGDTCAVITAIDEQSPDIAQGV-NR-ALEVR-E--T-DMT-D--DEIN-LI-GAGDQGMMIGYACDETEELLPMPVALAHKLARRLAYVRKEK--I-IPYLRPDGKTQVTLEY--E-D--G--R-PVRVDTV-IVSAQH-HPSVE---------QE----QIKA-DIVEHVIEPI-----M-P-P-------D-L-ME--DSP--KVLVNPTGRFVLGGPLADTGLTGRKIIVDTYGGVVPHGGGCFSGKDPTKVDRSGAYMARYAAKNVVAAGLARKCQIQVAYAIGVARPVSISVETYGTGV--ISDE----RITDLVREYFDFRPAAMIRDLDLRK-P-------QYMSLAAYGHMGR-I----------------------DLN--PI--PAWERT-DK-A-----EAMK----K----AAR-----------------

>Synergistetes_Thermanaerovibrio_velox

---------M-SK------------ER-F-LFTSESVTEGHPDKLADQISDGILDAILEKDP-----MGRVACETLVTTGLVVVAGEISTSC-YV--------DIPRLAREIVKEIGYTR--AK-YGFDGDTCAVVTSIDDQSPDIAQGV-DR-ALEIR-E--S-HMT-D--SQID-AI-GAGDQGMMFGYACDETEELMPAPISLAHKLARRLAMVRKQR--I-LPYLRPDGKTQVTLEY--V-D--G--K-PVRVDTI-VVSTQH-HPAVD---------AS----QIEA-DVVEHVIYPV-----I-P-A-------H-L-ME--RKP--RILVNPTGRFVLGGPLADTGLTGRKIIVDTYGGMVPHGGGAFSGKDPTKVDRSAAYMARYAAKNVVAAGLAKACQIQVAYAIGVAHPVSIMVDTFGSGV--ISDE----ALTQLIREHFDFRPGAIIRDLDLRK-P-------QYRRLAAYGHMGR-M----------------------DLD--PL--PAWERT-DR-A-----EVLR----K----AAERV---------------

>Synergistetes_Aminiphilus_circumscriptus

---------M-AN------------ER-F-LITSESVTEGHPDKLADQISDGVLDAILEQDP-----MGRVACETLVTTGLVVVAGEITTSC-YV--------EIPALARSIVKEIGYTR--AK-YGFDGDTCAVITAIDEQSPDIAQGV-DK-ALEIR-E--K-DMN-D--DEID-KV-GAGDQGMMIGYACNECEEYLPLPIALAHRLARRLAKMRRDC--V-LPYLRPDGKTQVTLEY--E-G--R--K-ALRADTI-VVSTQH-HPEVE---------QK----QIAA-DILEHVIRPV-----I-P-A-------A-L-LD--GKT--RLLVNPTGRFVLGGPMADTGLTGRKIIVDTYGGVAPHGGGAFSGKDPTKVDRSAAYMARYAAKNIVAAELAEVCQIQVAYAIGVAHPVSIMVETFGTGV--VDNA----TLTRFVREQFDFRPAAIIRDLELRK-P-------QYRRLAAYGHMGR-I----------------------DLD--PL--PRWECT-DR-A-----EALR----K----AAGR----------------

>Synergistetes_Thermovirga_lienii

---------M-RKANVQIWRWKRLTSK-F-LLTSESVTEGHPDKLADQISDGVLDAILAEDP-----YGRVACETLVTTGLVVIAGEITTSC-YV--------DIPRLTRQIIKDIGYTR--AK-YGFDGDTCAVITAIDEQSPDIKGGV-DE-AIEVR-E--K-GQI-T--DELD--V-GAGDQGIMVGYACDETEEFLPLPIALAHRLARRLSFVRKEK--I-LPYLRPDGKTQVTVEY--E-E--G--K-AVQIDTV-VVSTQH-HPAVE---------ND----QIEQ-DIIEEVINPI-----I-P-E-------H-L-VP--RKP--RILVNPSGRFVLGGPMADTGLTGRKIMVDTYGGVIPHGGGAFSGKDPTKVDRSAAYMARYAAKNVVAAGLAKECKIQVAYAIGMAHPVSIMVDTNGTGV--LPDP----KITQLVIEHFDFRPAAIIRDLEMRK-P-------QYRRLAAYGHMGR-V----------------------DLD--PM--PAWERT-DR-T-----EQLR----E----AAEKL---------------

>Synergistetes_Aminobacterium_colombiense

---------M-AD------------RK-F-LITSESVTEGHPDKIADQISDGVLDAILADDP-----MGRVACETLVTTGMVMVAGEISTST-YV--------DIPKLARDIVKEIGYTR--AK-YGFDGDTCAVLTAIDEQSSDIAQGV-DT-ALEVR-SH-T-EMG-E--AEVA-KV-GAGDQGMMIGYACTETEEYLPMPIALAHRLARRLALVRKNG--T-IPYLRPDGKTQVSLEY--V-D--G--Q-AIRAENL-VVSAQH-HPGAT---------EA----QIRG-DIIEHVIKPV-----V-P-E-------H-L-LR--PDT--TILVNPTGRFVKGGPMADTGLTGRKIIVDTYGGWVSHGGGAFSGKDPTKVDRSGAYMTRYAAKNIVAAGLAEKCMIQIAYAIGVAEPVSLMVDTFGTGK--ISDE----ALTDLIERHFDFRPAAIIRDLDLRK-P-------QYRRLAAYGHMGR-I----------------------DLD--PL--PQWERT-DR-A-----DALA----R----DAKRLK--------------

>Synergistetes_Fretibacterium_fastidiosum

---------M-TDK-----------DR-F-IFSSESVTEGHPDKVADQISDGVLDAILKDDP-----NGRVACETLCTTGLVMVSGEITTST-YV--------DIPKLAREIVKEIGYTR--AK-YGFDGETCAVLTAIDEQSGDIAQGV-DN-AIEVR-D----KLS-N--EDIS--I-GAGDQGMMFGYACNETKEFMPMPIALAHNLARQLTKVRRDG--T-IPYLRPDGKTQVSLRY--E-G--H--K-AVHADTI-VVSAQH-HPEAG---------LK----EIRA-DIIEHVINPI-----V-P-D-------A-L-LD--TDT--RIFVNPTGRFVKGGPMADTGLTGRKIIVDTYGGWIPHGGGAFSGKDPTKVDRSAAYMTRYAAKNVVAAGIADKCQIQVAYAIGVAEPVSLNVETFGTSR--ISHE----KIVELIRERFDFRPAAIIRDLDLRR-P-------QYRALAAYGHMGR-I----------------------DLP--HL--PCWEKT-DR-A-----EELR----K----AAGL----------------

>Synergistetes_Jonquetella_anthropi

---------M-K-------------ER-V-FITSESVTEGHPDKIADQISDAVLDAVLAEDP-----MGRVACETLVTTGLVQIAGEITTTA-RL--------DYPDIARGVVREIGYTR--GK-FGFDCDTCAVVTSIDRQSPDIAMGV-DQ-SLERK-G--G-DES-N--DEA---V-GAGDQGMMIGYATDATPEFLPEPFALAQRMARRLTAVRKVG--E-LTYLRPDGKTQVTLEY--E-G--G--K-AVRADAI-VVSTQH-NPEVS---------LE----QLRS-DVKRLVIEPV-----M-P-K-------N-L-MD--GKT--KFLINPTGRFVLGGPHADTGLTGRKIIVDTYGGMVPHGGGAFSGKDPTKVDRSAAYMARYAAKNVVASGLAHRCQIQVAYAIGVAEPVSLMVETFGTSQ--VSEE----ALTKALRQVFDFRPAAIIRTLDLRR-P-------QYRRLAAYGHMGR-I----------------------DLD--PL--PKWENT-DK-A-----DELR----R----LLS-----------------

>Tenericutes_Mycoplasma_feriruminatoris

---------M-TQN----------IEK-R-LFTSESVSEGHPDKICDQISDAILDQVLSQDP-----NGKVACEVFATTNYLLIGGQITTSA-VV--------DYEKVARDVLKKIGYID--DA-YGINANTCKIDIRIESQSLDIAQGV-EL----ND-H----------------EI-GAGDQGIMFGYATNESKTYLPLAITIAHELVYNATAQRKQG--L-FKWARPDMKSQVTIDYT---D----IN-NPKIDTI-LMSIQH-DPDYN---------EA----EFKK-YIKENIMDLV-----A-K---------E-FNLN--TDF--KVLINPTGRFVIGGPQGDTGLTGRKIIADTYGGYSRHGGGAFSGKDSTKVDRSAAYMCRYVAKNLVAAGLADKLEIQVSYAIGISQPISIFIETFNTHK--VDLN----TIYKAVYENFDFSVSSMIKTLDLRQ-P-------IFFKTSKYGHFGKKD----------------------------L--P-WEKL-DK-I-----EVLK----EYKKCS-------------------

>Tenericutes_Mesoplasma_seiffertii

-------------------------MR-N-LFTSESVSEGHPDKICDQISDAILDEVLKQDP-----NAKVACETFATTNYLLIGGQISTTA-VV--------DYETIARDILRKIGYNN--DD-YGINAETCKIELRIEEQSHDIALGI-DQ----TE-Q----------------LI-GAGDQGIMFGYATNESKTFLPLAITIAHELVYTASKLRKQK--T-FKWARPDMKSQVTIDYT---D----LE-NPKIDTI-LMSIQH-DADFN---------EK----EFKA-YIKTNIMDKV-----A-L---------E-FGLN--TDF--KVLINPTGRFVIGGPQGDTGLTGRKIIVDTYGGYARHGGGAFSGKDSTKVDRSAAYMARYAAKNLVAAGLADKLEIQVSYAIGVAEPVSIFVETFGTEK--VAKA----VILKALEENFDFSVYNLIKELDLRK-P-------VFLKTSTYGHFGKAD----------------------------F--S-WEKL-DK-V-----EELQ----KYL----------------------

>Tenericutes_Entomoplasma_somnilux

-------------------------MR-N-LFTSESVSEGHPDKICDQISDAILDEILKQDP-----NAKVACETFATTNYLLIGGQITTTA-KV--------DYEAIARDVLRKIGYNN--DD-YGINAETCKIDIKIEGQSPDIALGI-DL----NA-Q----------------TI-GAGDQGIMFGYATNESKTYLPLAITLSHELVYTATKLRKNK--E-FKWARPDMKSQVTIDYT---N----KN-KPVIDTI-LMSIQH-DENFN---------EQ----EFKT-YVKKHIMDKV-----A-L---------D-FGLN--TDF--KVLINPTGKFVIGGPQGDTGLTGRKIIVDTYGGYARHGGGAFSGKDSTKVDRSAAYMARYAAKNLVAAGLADKLEIQVSYAIGKPEPVSIFVETFKTNH--VNQK----IIEQALLENFSFSVIDMITNLKLRE-P-------VFLKTATYGHFGKKD----------------------------F--A-WEEL-DK-V-----PLLK----KYLK---------------------

>Tenericutes_Mesoplasma_florum

-------------------------MR-K-LFTSESVSEGHPDKICDQISDAILDEVLKQDP-----NAKVACETFATTNYLLIGGQITTTA-SV--------DYEKIARDVLRKIGYNN--DA-YGINADTCKIDIRVEQQSADIALGI-DL----DT-E----------------VI-GAGDQGIMFGYATNESKTFLPLAITISHELVYLASKLRKEG--K-FKWARPDMKSQVTIDYT---D----ES-NPKIDTI-LMSIQH-DDEMI---------EE----EFKK-FIKSEIMDVV-----A-K---------E-FELN--TDF--NVLINPTGRFVIGGPQGDTGLTGRKIIVDTYGGYSRHGGGAFSGKDATKVDRSAAYMARYAAKNLVASGLADKIEIQVSYAIGKPEPVSIFIETFGTEK--VSKE----VIAKALNENFDFSVNEIIKKLDLRK-P-------TFLKTATYGHFGKDE----------------------------F--T-WEQL-DK-V-----KTIK----K------------------------

>Tenericutes_Spiroplasma_taiwanense

-------------------------MK-K-FFTSESVSEGHPDKLCDQISDAILDECLKQDK-----NSKVACETFITDNFVLIGGEIKTTA-IV--------DYKEIAKWVLSRVGYKD--ES-TGINPNKAEIIVKIHEQSPDIAIGV-E------K-E----------------DM-GAGDQGIMFGYANNETSNYMPYSIQVAHDLVHIASKLRKAG--A-FKWAQPDMKSQVTIDYT---N----SK-NPRIETI-LMSVQH-DKDYN---------KE----EFEN-FIKKNIMDVI-----A-K---------R-YNLN--TDF--NVLINPTGRFVIGGPKSDTGLTGRKIIVDTYGGYSRHGGGAFSGKDASKVDRSAAYMCRYAAKNIVAARLADQLEIQVSYAIGKADPISIFIESFGTNK--VSND----VLYKALIENFNFNLSSIIKELDLNS-P-------VYFRTSKYGHFGKKE----------------------------FN-N-WEKL-DK-V-----RVLK----EFL----------------------

>Tenericutes_Phytoplasma_mali

---------M------------------Q-KFTSESVTKGHPDKVADQISDALLDAFLEQKK-----DARVAIETIVTAKTVFILGEVEPNI-FI--------DYNNIIKKIVRSIGYDR--RE-ENFSYQDLKIINKIHSQSKEISLVV-DK-------N-------------------VPGDQGLMFGYATNETLSFLPLGFDLSKKLSLRLTEVRENK--I-LPFLRPDGKTQITMVY--DFN--N--N-PLYIDSI-VISSQH-EANID---------RK----ILVD-AIKKEVIMPV-----I---D-------NNF-IN--SKT--NYYINPAGSFINGGPNADTGLTGRKIIVDTYGGYSRHGGGSFSGKDSSKVDRSASYMARYLAKNIVASGICDICEIQISYAIGIENPMGIYINTFNTNK--VSEK----LILQTIKNNFDLRLKGIIQKLDLQN-P-------IYQQTASEGHFGNYK--------------------------HNF--S-WEKI-DK---KNIFEKLF-----------------------------

>Tenericutes_Acholeplasma_brassicae

------------M------------ER-K-LFTSESVTSGHPDKICDRISDAILDAILEKDS-----NARVACETTVTTGLVLVVGEITTET-YV--------DIQTIVRNTVKNIGYDR--GK-YGFDADNLAVLTSINSQSEDIALGV-DE-TDS------H-------------EQ-GAGDQGLMFGFACDETDNLMPLPIELAHKLTRRLDFVRQEN--L-LDFLRPDGKAQVTIEY--D-E--Q-DR-PLRIDAV-VVSSQH-Q-DLP---------IE----VVRK-GIKELVIDAV-----L-P-S-------E-L-ID--ENT--KYFINPTGKFVIGGPKGDSGLTGRKIIVDTYGGYARHGGGAFSGKDPSKVDRSASYMARYIAKNIVASGISKKAEVQLAYAIGVAQPVSVRVDTFNTSK--VSES----LIEKAVRKHFDLTPKGIINTLDLKR-P-------IYEQTSNYGHMGR------------------------EDI--DL--P-WEKL-NK-I-----DIFK----D----LL------------------

>Tenericutes_Anaeroplasma_bactoclasticum

-------------------------MK-R-FFTSESVTEGHPDKIADQISDAILDDILAHDP-----NGRVACETICTTGMVMVFGEITTNH-YV--------DVQKIVRDTVTEIGYVR--GK-YGFDADNLAVLTAIDPQSPDIAMGV-DE-SSDH-------------------AQ-GAGDQGIMFGYACRETEEYMPLAITLAHKLCYKLTEVRKNG--T-LSYLRPDGKSQVTVEYD-D-N--G--K-IYRIDTV-LISTQH-SPDVS---------LE----QLAA-DVKKYVVDEV-----I-P-S-------S-L-VD--KDT--KFLFNPTGRFVIGGPAGDSGLTGRKIIVDTYGGAACHGGGAFSGKDPSKVDRSASYMARYIAKNVVAAGLCDKCQIQLSYAIGVAEPTSVLVDTFGTAK--VSEE----KIQDKILEVFNLTPKGIIRTLDLKR-P-------IYKKTAAYGHFGR-T----------------------DID---L--P-WEHL-DK-V-----SELKT-LLK------------------------

>Thermodesulfobacteria_Thermodesulfobacterium_thermophilum

-----M---L-VQ------------N--F-LFTSESVTEGHPDKVADQISDAILDAILEKDP-----YARVACETLVNTGMILIAGEITTEA-RI--------DYPTIARGVVKEIGYNH--SD-LGFDYQTCAVLISIDRQSPDIAMGV-DR-D-----G----------------EI-GAGDQGLMFGYACDETPDFMPMPIWYAHKLAMRLAEVRKKG--I-LPFLRPDGKTQVTIRY--E-M--R--R-PVDVHTI-VIAAQH-DPTVT---------LK----ELRE-AIMEEVIKKV-----I-A-P-------E-H-LK--SDT--KIIINGTGRFVIGGPLADCGMTGRKIIVDTYGGRGHHGGGAFSGKDPTKVDRTPSYYGRYVAKNLVAAGVAKELEVQVAYAIGVPEPLAININTYGTEN--IPVE----KILDIIHKLFDFRPKHMIEYLNLRR-P-------IFRKTACYGHFGR-N----------------------EPE---F--T-WEKL-DM-V-----EKIK----E----LAGFDK--------------

>Thermodesulfobacteria_Caldimicrobium_thiodismutans

-----M---L-VK------------N--F-LFTSESVTEGHPDKVADQISDAILDAILEKDP-----YARVACETLVNTGMILIAGEITTEA-RI--------DYPTIARGVVKEIGYNH--SD-LGFDYQTCAVLISIDRQSPDIAMGI-DR-D-----G----------------EI-GAGDQGLMFGYACDETPDFMPMPIWYAHKLAMRLAEVRKKG--I-LPFLRPDGKTQVTIRY--E-D--R--R-PVDVHTI-VIAAQH-DPTVT---------LK----ELRE-AIYEEVIKKV-----I-A-P-------E-H-LR--PDT--KIIINGTGRFVIGGPLADCGMTGRKIIVDTYGGRGHHGGGAFSGKDPTKVDRTPSYYARYVAKNLVAAGVARELEVQVAYAIGVPEPLAINVNTYGTET--IPVE----KILDIINQLFCFRPKHMIEYLNLRR-P-------IFRKTACYGHFGR-N----------------------EPE---F--T-WEKL-DM-V-----EKIK----E----LAGFDK--------------

>Thermodesulfobacteria_Thermosulfurimonas_dismutans

-----M---G-LS------------N--F-LFTSESVTEGHPDKVADQISDAILDAILEKDP-----YARVACETLVNTGMILIAGEITTEA-RV--------DYATIARGVVKEIGYNH--SD-LGFDWQTCAVLTSIDRQSPDIAMGV-DR-G-----E----------------EI-GAGDQGLMFGYACDETPDYMPMPIWYAHRLAMRLAEVRKKG--I-LPFLRPDGKTQVTIQY--E-D--R--R-PVAVHTV-VVAAQH-EPWVE---------YK----ELKE-AIVEEVIKKV-----V-A-P-------E-H-LT--SET--KFLVNTTGRFVIGGPLADCGMTGRKIIVDTYGGRGHHGGGAFSGKDPTKVDRTPSYYARYVAKNMVAAGVARELEVQVAYAIGVPEPLAINVQTYGTNT--IPIE----RIVEIIKELFDFRPREMIEYLNMRR-P-------IFRKTACYGHFGR-P----------------------DPD---F--T-WERL-DM-V-----EKIK----E----LAGFEK--------------

>Thermotogae_Petrotoga_sibirica

----------------------------M-LFTSESVTEGHPDKICDQISDTILDAILEKEPEENKINARCAVETLVTRGLVIVTGEVRTSA-YI--------DVPTLVRNTILDIGYNR--AK-FGFDGETCAVITSIEEQSADIALGV-DR-SLEVK-S--K-Q-EEE--DPFE-KI-GAGDQGIMFGYATNETDAYMPLPILLAHRLAKRLADVRKSD--T-LDFLRPDGKTQVTVEYD-E-N--N--K-PVGIETI-LISTQH-SPDIK---------RQ----ELEE-AIKEHVIIPV-----I-P-E-------N-L-FT--KNT--KILINPTGRFVIGGPQADTGLTGRKIIVDTYGGWAPHGGGAFSGKDPTKVDRSATYMARYVAKNLVASGAADEVLIQLSYAIGVAEPVSINIDTKGTAK--VEEE----KIYKVVKEIFDFRPAAIINNLDLLQ-P-------IYKKTAAYGHFGR-K----------------------DVE---F--P-WERL-DK-V-----KELK----A----ALGL----------------

>Thermotogae_Defluviitoga_tunisiensis

------------M------------KK-M-LFTSESVTEGHPDKICDQISDAILDTLLEKEPKDNRINVRSAVETLVTRGLVVVTGEIRTSA-YV--------DVPTVARNTVLEIGYNR--AK-FGFDGETCAVVTAIEEQSPDIAMGV-DK-SFESK-T--K-K-DKI--DPYD-RI-GAGDQGIMFGYATNETDVYMPMPIVLAHRLARRLSEVRKSK--V-VEFLRPDGKTQVTVAYD-E-N--F--K-PVAIDTI-LISAQH-APDVT---------RQ----ELEE-GIKKYVIEPV-----I-P-Q-------E-L-IT--KDT--KILINPTGRFVIGGPQADTGLTGRKIIVDTYGGWAPHGGGAFSGKDPTKVDRSATYMARYVAKNLVASGAAEEVLIQLSYAIGVAQPVSINIDTRGTAK--VDEE----KIYKVVREIFDFRPAAIIENLNLLQ-P-------FYKKTAAYGHFGR-D----------------------DFE---F--P-WEKL-DK-V-----KELR----S----ALGL----------------

>Thermotogae_Geotoga_petraea

------------M------------NK-R-LFTSESVTEGHPDKVADQISDAILDEMLSKESEQNRINARSAVETMVTTGVAIVAGEMRTDA-YV--------DIPKIVRQTILDIGYDN--PK-YGFDGETCAVMVSIDEQSADIAMGV-DE-NLETK-E--G-S-ATK--EIFD-KI-GAGDQGIMFGYATNENNDYMPTPISLSHKLARQLSVVRKDK--T-LDYLRPDGKTQVTVEYD-E-N--N--K-PIAIDTV-LISTQH-EEGVT---------RD----QMSK-DLIEKVITPV-----I-P-E-------G-L-LN--KHT--KYLINPTGRFVLGGPQADAGLTGRKIIVDTYGGWIAHGGGAFSGKDPTKVDRSAHYMSRYVAKNLVAAGVADEVMIQLGYAIGVAHPVSLLIDTKGTAK--VDEE----KIVKVVKELFDFRPGAIIHNLNLLQ-P-------FYKKSAAYGHFGR-T----------------------DVE---F--P-WERL-DM-V-----QEIK----A----ALGL----------------

>Thermotogae_Thermotoga_caldifontis

------------M------------KR---LFTSESVTEGHPDKLADQISDAILDAILEQDP-----NARVAVETLVTTGLAVVAGEVTTEA-YV--------DIQDIVRRTILDIGYTR--AK-YGFDGETCGVLTSIHNQSPDIAIGV-NR-ALEAR-E--K-N-HLD--DEYD-KI-GAGDQGMMFGYATNETPEYMPLPIMLAHKLAMRLAEVRKKK--I-VPFLRPDGKTQVTIEYD-E-N--W--K-PVRVDTI-LISAQH-EPDVT---------IQ----ELRE-ALIEHVIKPT-----I-P-E-------Q-Y-WS--NDV--KILVNPTGRFVLGGPSADTGLTGRKIIVDTYGGWVPHGGGAFSGKDPTKVDRSAHYMARYVAKNVVAAGLADRFMIQVAYAIGKARPVSLMIETFGTAK--VDEE----KLKKAIIELFDFRPLAIIERLNLRR-P-------IYRKTAAYGHFGR-N----------------------DPD---F--T-WERL-DA-V-----DELR----R----YFNM----------------

>Thermotogae_Pseudothermotoga_thermarum

------------M------------KK---LFTSESVTEGHPDKMADQISDAILDAILEQDP-----NARVAVETLVTTGLAIVAGEVTTEA-YV--------DIQDIVRKTILEIGYTR--AK-YGFDGETCGVLTSIHNQSPDIAIGV-NK-ALEAR-E--K-N-NDV--DEYD-KI-GAGDQGMMFGYATNETPEYMPLPIMLAHKLAMRLAQVRKEK--I-VPFLRPDGKTQVTVEYD-E-N--G--K-PVAVDTI-LISAQH-EPDVS---------LQ----DLRD-ALIEYVIKPV-----I-P-E-------Q-Y-MK--KDT--KILINPTGRFVLGGPSADTGLTGRKIIVDTYGGWIPHGGGAFSGKDPTKVDRSAHYMARYVAKNVVAAGLADKFMIQVAYAIGKARPVSMMIETFGTCK--VDEE----KLKKVINEIFDFRPKAIIDRLKLLR-P-------IYKKTAAYGHFGR-N----------------------DPD---F--T-WEAL-DA-V-----PELK----R----AFNL----------------

>Thermotogae_Thermosipho_globiformans

------------M------------KR---LFTSESVTEGHPDKVADQISDAILDAMLEQDP-----KSRVAVETLVTTGIAIVSGEVTTRA-YV--------DIQDIVRKTILDIGYTR--AK-YGFDGETCAVLSSIHSQSPDIALGV-DK-ALEAK-E--G-ELKAE--DELE-QV-GAGDQGMMFGYATNETKEYMPLPIMLSHKLAMKLSEVRKNG--T-LPFLRPDGKTQVTIEYD-E-N--D--K-PVRVDTV-LISTQH-EPDVT---------IP----EIKE-ALIKHVIDPI-----I-P-E-------E-L-RD--DNM--KILVNPTGRFVLGGPSADTGLTGRKIIVDTYGGAVPHGGGAFSGKDPTKVDRSAHYFARYVAKNVVAAGLADKFMIQVAYAIGKAHPVSVMINTFGTAK--TDED----KILKAILEIFDFRPGAIIKKLNLLR-P-------IYKKTAAYGHFGR-E----------------------LEE---F--T-WEKL-DM-V-----NELK----K----I-------------------

>Thermotogae_Fervidobacterium_nodosum

------------M------------RR---LFTSESVTEGHPDKMADQISDAILDAIIEQDP-----KARVAVETLLTTGVAIVAGEVTTKA-YV--------DVQDIVRKTVLDIGYTR--AK-YGFDGETCAVLSSIHSQSPDIALGV-NE-AAEFK-Q--G-D-KAE--DEIE-KV-GAGDQGMMFGYATNETEELMPLPIMLAHKLAMKLAEVRKNG--T-VPFLRPDGKTQVTIEYD-E-N--E--K-PVRVDTV-LISTQH-EPDVT---------IP----EIRE-ALVKYVINPI-----I-P-E-------N-L-RD--DNM--KILVNPTGRFVLGGPSADTGLTGRKIIVDTYGGAIPHGGGAFSGKDPTKVDRSAHYFARYVAKNVVAAGLADKFMIQVAYAIGVARPVSIMINTFGTAK--VDEE----KLRKVVEEIFDFRPGAIIKKLDLLR-P-------IYRKTAAYGHFGR-N----------------------DPD---F--T-WERT-DM-V-----RELK----A----AFNL----------------

>Thermotogae_Mesotoga_prima

------------M------------KT-W-LFTSESVTEGHPDKMADQISDAILDAMLTQDE-----NSRVAVETLLATGVAVVAGEVSTKA-YV--------DIPRVVRDTILDIGYNR--AK-FGFDGETCAVLTSIDEQSPDIALGV-DR-SYEAK-K--N-D---V--DRYA-LI-GAGDQGMMFGYATNETPEMMPLPIVLAHRLARRLSQVRRDG--T-VQGFRPDGKTQVTVKY--Q-D--G--K-PVGVTAI-VVSTQH-DPDLT---------SH----EIER-LVVDNVVAPV-----I-E-H-------D-L-LL--EGV--EIFVNPTGRFVKGGPSADTGLTGRKIIVDTYGGWIPHGGGAFSGKDPTKVDRSAHYMARYTAKNIVAAGLAERVTLQLAYAIGVAKPVSFMIDAHDTEK--VDLE----KLRKAVLKVFDFRPAAIIDRLNLRR-P-------IYRQIAAFGHFGR-I----------------------DVD---L--P-WEET-DA-V-----DQLK----K----ALD-----------------

>Thermotogae_Kosmotoga_olearia

------------M------------KK-W-LFTSESVTEGHPDKIADQVSDAILDAMLEQDP-----ESRVAVETLVATGVAVVAGEVTTKA-YV--------DIPRIVRDTILEIGYTR--AK-FGFDGETCAVLTSIDEQSPDIALGV-NK-SLEAK-N--D-G---K--DKYD-II-GAGDQGMMFGYATNETEEMMPLPIVLAHNLARRLATVRKER--I-VEGFRPDGKTQVTVLY--E-D--G--K-PIGVKTI-VVSTQH-DPAMT---------QE----EIEQ-LVKEHVIKAV-----V-P-E-------E-M-ML--DGI--ETFVNPTGRFVKGGPAADTGLTGRKIIVDTYGGWIPHGGGAFSGKDPTKVDRSAHYMARYVAKNIVAAGLADRVTLQIAYAIGVAHPVSFMIDAHGTEK--VALE----KLEKVVKEVFDFRPAAIIDKLNLRR-P-------IYKQVAAYGHFGR-I----------------------DVE---V--P-WEKL-DA-V-----DELK----R----AFNM----------------

>Thermotogae_Mesoaciditoga_lauensis

------------M------------KK---LFTSESVTEGHPDKIADQISDAILDEALAQDP-----QSRVAVETFVIRGEVMVGGQMTTKA-YI--------DIPTIVRQTVLDIGYTR--AK-YGFDGETCAVITAIDEQSPDIALGV-DT-ALEFK-E--G-E-KTE--DPLD--I-GAGDQGIMFGYASNETEEYMPLSISLAHKLARKLTEVRKNK--V-LDFLRPDGKTQVTVEFD-G-D--G--K-PIAVDAI-VVSTQH-DPTVS---------EI----DLKN-GIREEVINKI-----V-P-Q-------N-Y-MT--KNT--KIYINPTGRFVIGGPMADTGLTGRKIIVDTYGGVAPHGGGAFSGKDPTKVDRSASYMARYVAKNVVAAGLASKCLVQLAYAIGVARPVSIFVDTFGTGV--VDDI----ELTKIINELFDFRPGAIIKNLDLLR-P-------MYKKVAAYGHFGR-D----------------------DLD---L--P-WERL-DK-V-----AELK----A----AVK-----------------

>Thermotogae_Fusobacterium_nucleatum

---------M-KKF---------------TYFTSEFVSPGHPDKISDQISDAILDACLKDDP-----NSRVACEVFCTTGLVVVGGEITTST-YI--------DVQDIVRKKIDEIGYRPG----MGFDSN-CGTLSCIHAQSPDIAMGV-DI-G-------------------------GAGDQGIMFGGAVRETEELMPLALVLSREILVKLTNMMKSN--E-IEWARPDQKSQVTLAYD-E-N--G--K-VDHVDSI-VVSVQH-DEDTT---------HD----EIEK-IVIEKVVKPV-----L---E-------K-YNLSS-DNI--KYYINPTGRFVIGGPHGDTGVTGRKIIVDTYGGYFKHGGGAFSGKDPSKVDRSAAYAARWVAKNIVAAELADKCEIQLSYAIGVPKPVSVKVDTFGTSK--VDED----KISEAVSKVFDLSPRGIEKALELRE-GNF-----KYQDLAAFGHIGR-T----------------------DID---T--P-WERL-NK-V-----DELK----K----AIEL----------------

>Verrucomicrobia_Coraliomargarita_akajimensis

---------M-SK------------N--F-IFSSESVGEGHPDKVADYISDSVLDACLAQDP-----KSRVACETLVKSNCVFLAGEITTNA-KL--------NYEEIARQAIREIGYVN--DD-DVFHADKVFVSNILTAQSSDIAQGV-DA-AAAE-----G--------KDTE-EQ-GAGDQGIMFGYACNQTPELMPAPVMFAHRLLREMARQRKEI--K-VPWLRPDVKSQVALEY--V-D--G--K-PVAIKNV-VISTQH-AADIK---------HA----AIKE-FCIDEVIRKV-----L-P-A-------D-L-LK--DET--EFLINPTGNFVIGGPQGDAGLTGRKIIVDTYGGWARHGGGAFSGKDPSKVDRSAAYFTRWVAKNIVAAGLADECELEVAYAIGHPYPTSIHVDTFGTGK--ADDG----KIAEAAQKVFSFKPADIVSQLDLLR-P-------IYRESTHYGHYAK--------------------------E--QL--P-WEST-AK-A-----EELK----A----AL------------------

>Verrucomicrobia_Geminisphaera_colitermitum

---------M-SQ------------E--F-VFSSESVGEGHPDKVADLISDSVLDACLAADP-----HSRVACETMVKSNMVILAGEITTSA-KP--------NYEAVVREAIRDIGYVN--ND-DVFHADSVFINNYLTRQSPDIAQGV-DA-RAAE-----G--------KDTA-EQ-GAGDQGLMFGYACNETPEMMPTAIMYAHRLGRELTKLRKSG--V-VSWLRPDAKSQVSIRY--V-N--D--K-PVAVENV-VISTQH-QDGVA---------HG----AIRE-FLIENVIRKV-----I-P-A-------D-M-IA--PAT--KFLINPTGRFVIGGPQGDSGLTGRKIIVDTYGGWGRHGGGAFSGKDPSKVDRSAAYMCRWVAKNIVAAGLADICELQVAYAIGYPDPVSVWVDAMGTAK--VPEA----RIVAAVKKVFDFKPAAIIRQLDLLR-P-------IYRSTTNYGHFGK--------------------------D--DL--P-WEQT-NR-T-----AELI----A----AAKS----------------

>Verrucomicrobia_Lacunisphaera_limnophila

---------M-AN------------S--F-VFSSESVGEGHPDKVADLISDSVLDACLTQDK-----HSRVACETMVKSNMVILAGEITTSA-KL--------NYEAIVRAAIRDIGYVN--ND-DVFHADQVFINNYLTRQSPDIAQGV-DA-KKAK-----G--------KKTA-EQ-GAGDQGIMFGYACNETPELMPTPIMFAHRLGRELTKIRKSG--K-APWLRPDAKSQVSVRY--E-N--D--I-PVEVVNV-VISTQH-TADVA---------HS----TIES-YLIENVIKKV-----I-P-S-------R-M-LT--KKT--EYLINPTGRFVIGGPQGDSGLTGRKIIVDTYGGWGRHGGGAFSGKDPSKVDRSAAYMGRWVAKNIVAAGLATHAEIQFAYAIGHPQPVSVRVETFGTAKLGISDE----QITAAVTKTFSFKPADIVSELNLLR-P-------IYRQTTNYGHFGK--------------------------S--GL--P-WEQT-NK-A-----ALLR----A----AIK-----------------

>Verrucomicrobia_Ereboglobus_luteus

---------M-AK------------T--F-VFSSESVGEGHPDKVADYISDSVLDACLAQDK-----TSRVACETMVKSNVVILAGEITTKA-RL--------NYETIVRDAIREIGYTN--ND-DVFHADTVFINNYLTSQSPDIAQGV-DK-RKAK-----G--------KKTA-EQ-GAGDQGLMFGYACDETPELMPLPIMLAHRLGRELTKIRKSGGKK-WAWLRPDAKSQVSIRY--E-N--D--R-PIEVVNV-VISTQH-TADVE---------HA----QIES-ALIDNVIRKV-----I-P-A-------D-L-LN--GNT--QYLINPTGRFVVGGPQGDSGLTGRKIIVDSYGGWGRHGGGAFSGKDPSKVDRSAAYMARWAAKNIVAAGLAKYAELQLAYAIGYPHPVSVFVDTFGTGK--VEDE----KIARAVQKVFSFKPADIIKQLNLLR-P-------IYRETTNYGHFGK--------------------------A--GL--P-WEQT-NK-T-----AALR----A----AVK-----------------

>Verrucomicrobia_Pedosphaera_parvula

---------M-SK------------N--F-IFSSESVGEGHPDKVCDTISDAVLDACLAQDP-----RSRVACETYAKSNLVVIGGEITTRA-KL--------DYNAIAREAIREIGYVN--DD-DVFHADRVLIMNAITSQSADIAQGV-DA-KAAE-----G--------KDTD-EQ-GAGDQGLMFGYACNETPELMPAPIMYAHQLGRELTRIRKSG--K-VAWLRPDAKSQVSVQY--E-N--G--K-PVRITNV-VISTQH-AEDVK---------HK----EIKD-FIIKEVIRKV-----L-P-A-------Q-M-LD--KKT--QFLINPTGRFVVGGPQGDTGLTGRKIIVDSYGGMGRHGGGAFSGKDPSKVDRSAAYMGRYVAKNIVASGLATCAEIQFAYAIGHPDPVSVCVNTFGTGA--VSDE----AIEKAVREVFSFKPAAIVKQLNLLR-P-------IYSKTTNYGHFGKVD----------------------DTN--SI--T-WEKT-DK-A-----AALK----R----AAK-----------------

>Verrucomicrobia_Chthoniobacter_flavus

---------M-SR------------R--F-IFSSESVGEGHPDKVCDTISDAVLDACLRVDK-----HSRVACETYAKSNIVIVGGEITTKA-KL--------DYVQIVRDAVREIGYVN--DD-DVFHADRIFVQNIITAQSPDIAQGV-DA-AEAE-----G--------KGHA-EQ-GAGDQGLMFGYACNETPELMPAPIMFAHRLGRALTRIRKSG--Q-VGWLRPDAKSQVSVIY--D-G--N--D-VVGISNV-VISTQH-AADVK---------HK----EIRD-FLISEVIRKE-----L-P-A-------E-L-IT--DKT--EFLINPTGRFVVGGPQGDTGLTGRKIIVDTYGGMGRHGGGAFSGKDPSKVDRSAAYMGRWVAKNVVAAGLATSCEVQFAYAIGHPDPVSIHIDTFGTAK--VDEE----KIEKGVREVFSFKPGRIVEQLNLLR-P-------IYGKTTNYGHFGK------------------------DDP--EI--T-WERT-DK-V-----EALK----A----AVK-----------------

>Verrucomicrobia_Akkermansia_muciniphila

---------M-SR------------NTPH-IFTSESVGEGHPDKVADYISDSILDACLAQDK-----TSRVACETLVKSNMVIIAGELTTKA-VI--------DPEKIARQAIREIGYCNRQDD-DVFHADTVFFTNLLTEQSPDIAQGV-DA-REAE-----G--------KGHA-EQ-GAGDQGIMFGFATNETPELLPAPIVFAHKLLIELARRRKRG--H-VDWLRPDCKSQVAVAYD-E-D--G--R-PAHIENV-VISTQH-TEDVD---------HD----TIYS-YCVK-LIKNV-----L-P-A-------E-L-LD--ERT--EYFINPTGKFVVGGPHGDSGLTGRKIIVDTYGGMGRHGGGAFSGKDPSKVDRSAAYMCRWVAKHIVAAGLADKCELQVAYAIGYPAPVSIRVDTFGTGK--VEEI----SIENALENIFSFKPADMVEQLNLLR-P-------IYRKTTHYGHFTN--------------------------P--EL--P-WEQL-DE-T---RLASLK----Q----LLH-----------------

>Verrucomicrobia_Rubritalea_profundi

---------M-NQ------------S--Y-IFSSESVTEGHPDKISDTISDYIVDRCLEQDP-----NSRVACETLVKDNMVVLAGEIRTDA-KF--------NYQTAVYEAIKEIGYTH--DD-CDFHAEKFVFINALGQQSTDIAQGV-DD-VEAE-----G--------KDHD-EQ-GAGDQGLMFGYASNETKELMPAPIIYSHRLAQEVTRIRKAG--E-LAWLRPDAKTQVSCRY--V-D--G--K-VTEITAV-VISTMH-ADGIS---------HA----EIVE-AMTEKVIKKV-----L-P-Q-------E-L-IS--ENT--ELLINPTGNFVIGGPVGDCGLTGRKIIVDTYGGMGRHGGGAFSGKDPSKVDRSAAYMCRWVAKNIVAAELADKCEIQVAYAIGHPHPVSISIDCFETNK--VDED----AIIAAVNEVFSFKPADIVSQLNLLR-P-------IYSRTTNYGHFGREN------------------------A--DL--P-WEET-DK-V-----DALK----A----AIK-----------------

>Verrucomicrobia_Rubritalea_squalenifaciens

---------M-NR------------P--Y-IFSSESVTEGHPDKVCDTISDVILDACLEQDI-----NSRVACETFVKDNVVGLAGEITTHA-DF--------DSRSLVEKAIREIGYVH--DD-CKFNANSFFFVNLIGQQSPDIAQGV-DA-AAAQ-----D--------KLIA-EQ-GAGDQGIMFGYACNETPELMPAPISYSHKLGRELTLLRKSG--Q-HKWLRPDSKTQVSMEY--V-D--G--K-PSRVTAV-VVSTMH-SSDIT---------TG----EIRQ-ILKKDLIQRV-----I-P-A-------N-L-LT--DDT--ELLINPTGLFVIGGPEGDAGLTGRKIIVDTYGGTGRHGGGAFSGKDPSKVDRSAAYMCRWVAKNIVAAGLAEKAEIQVAYAIGYPHPVSISVNTFGTGT--VDEA----KIEAAVKEVFSFKPADIITQLDLVR-P-------IYRHTTNYGHFGRED----------------------NLS--AL--T-WEKK-NK-V-----DDLK----A----AIS-----------------

>Verrucomicrobia_Methylacidiphilum_fumariolicum

---------M-SK------------S--Y-VFASESVTEGHPDKVCDTISDTVLDHCLQQDK-----FSRVACETLVKENLVVLAGEITTKA-NL--------DYIKIVKDTVRQIGYND--PK-SLFYPEKLHIVCAISKQSPDIALGV-DG-KKERH-N-MG--------RNL--DQ-GAGDQGMMFGFACTETPELMPAPISFAHRLSRRLAELRKKE--G-CPWLRPDGKTQVSLYY--E-D--N--K-PMRVEAI-VVSTQH-TEEVS---------NK----EIEE-IIKKEVIQKA-----I-P-E-------E-F-LH--KKT--VFYINPTGRFVAGGPDADSGVTGRKIIVDTYGGMGRHGGGAFSGKDPSKVDRSAAYMARYIAKNIVAAKIANKVEVQIAYAIGKADPVSVAVDTFETGL--VEDQ----KIERAIREVFRLKPAEIIEELNLLR-P-------IYSKITNYGHFGRND----------------------EFD--IF--C-WEKT-DK-V-----NDLL----T----AVGLE---------------

>Alphaproteobacteria_Paracoccus_seriniphilus

---------M-PE------------Y--S-LFTSESVSEGHPDKIADQISDAILDAILAEDP-----RARVACETMVKTGVAIISGEITTSA-WV--------DLESIVRDVINDIGYTS--SD-VGFDGATCSVINIIGKQSPEINQGV-DR-ASL---E----------------EQ-GAGDQGLMFGYASDETDVLMPAPITYAHRLVERQSKVRRDG--T-LNWLRPDAKSQVTLRYG-E-D--G--R-PTGIDAV-VLSTQH-NPDIS---------LA----DLRE-AVIEEIIKPV-----L-P-A-------E-W-LS--AET--KYFINPTGKFVIGGPVGDCGLTGRKIIVDSYGGMARHGGGAFSGKDPSKVDRSAAYAGRWVAKNIVAAGLASRCEIQVSYAIGEAQPTSISLNTFGTET--VAHD----RIVAAVREVFDLRPFAIIRDLDLLH-P-------IYRPTAAYGHFGR-EPYALA----------------NATA---F--S-WERI-DR-A-----EALK----A----ALS-----------------

>Alphaproteobacteria_Rickettsia_akari

------------M------------KN-F-IFTSESVSEGHPDKMADQISDTVLDEILKHDP-----SGRVACETFVTTGLVLVGGEITTST-YV--------DIEQIVRSKIQEIGYNN---PNYGFDGSCCAVISSIMKQSPDIAMGV-DN-AND---D----------------EI-GAGDQGMVFGYACNETDSLMPAPIYYAHLLMRRQAYLRKQN--V-LPWLRPDAKSQVTLRY--E-N--N--K-PVAIDAV-VLSTQH-HPEIQ---------QK----DLIE-AVIEEIIKPT-----L-P-S-------S-W-LN--KDT--KYLINPTGRFVIGGPVADCGLTGRKIIVDSYGGMARHGGGCFSGKDPTKIDRSAAYMARYIAKNVVGAGLADRCEIQISYAIGVAAPVSVYTETFGTSK--LSNE----QITKLITQHFDMRPGKIIKHLALRT-P-------CYQKTASYGHFGR-E----------------------DAD---F--T-WEKL-DK-V-----DILK-----------------------------

>Alphaproteobacteria_Limimonas_halophila

---------M-AD------------S--Y-ILTSESVGPGHPDKIADQISDAVLDTVLSQDP-----GGRVACETLVNTGLIMLAGEITTTA-NV--------DYTQLARDVVTDIGYDD--SA-LGFDGNSAAVLLALDRQSPDIAQGV-DE-GAGLD-L----------------DQ-GAGDQGLMFGYACRETDELMPMPIQLAHRLTRRQAEVYRNG--K-LPWLRPDAKSQVSVRY--E-N--D--K-PAAVETV-VLSTQH-TPEVD---------HE----TLRE-GVIEEIVKPV-----M-P-A-------E-M-IA--RGT--EYLVNPTGRFVVGGPHGDCGLTGRKIIVDTYGGVGRHGGGAFSGKDPSKVDRSAAYAARYAAKNVVAAGLADVCEVQLAYAIGVARPVSVHVNTMGTAR--IPES----RIEALLRAHFDFRPKGIVQMLDLLR-P-------IYRQTASGGHFGR-S----------------------DID---L--S-WERT-DR-A-----ETLR----A----EAGETVAG------------

>Alphaproteobacteria_Fodinicurvata_sediminis

---------M-PK------------E--Y-IFSSESVGAGHPDKLADNISDAVLDEILKQDP-----AARVACETLVNTGLCILSGEITANA-TV--------DYTQIARDTILDIGYDR--SE-LGYDGNTCGVLLSIDKQSPDIAQGV-DE-GAGLD-R----------------SQ-GAGDQGMMFGYACRESDELMPLPILLSHKLTERQSIVRKEG--K-ISWLRPDVKSQVSVAY--E-N--D--K-PTHIDTV-VLSTQH-DPDVE---------YK----DLEE-AVVEEIIKPA-----L-P-K-------H-M-IS--DKT--RYLVNPTGRFVIGGPVGDCGLTGRKIIVDTYGGMGRHGGGAFSGKDSTKVDRSAAYATRYVAKNVVAAGLADKCEVQIAYAIGVAEPMSIHVNTFGTGR--IDET----QIEKLIREHFDLRPKAIVEDLDLRR-P-------IFRKTASNGHFGR-N----------------------DPD---F--T-WERT-DK-A-----EALA----N----DAGLMAAE------------

>Alphaproteobacteria_Amorphus_coralli

---------M-SR------------N--Y-IFTSESVGAGHPDKVADNVSDAVLDACLSVDP-----HSRVACETLVNTGLVVLSGEITTKA-QL--------DYSDIARETILDIGYDN--PA-YGFDGKSCAVLVALDKQSPDIAQGV-DE-GAGLD-L----------------EQ-GAGDQGLMFGYACRETDELMPLPIQLAHRLTAKQAEVRKSG--K-LPWLRPDVKSQVSVAY--D-D--N--K-PTHVDTI-VLSTQH-DEDVS---------YE----DLKA-AVIEEVVMPE-----V-K-A-------F-G-VN--KDI--KFHINPTGRFVIGGPVGDCGLTGRKIIVDTYGGMGRHGGGAFSGKDPTKVDRSAAYAARYVAKNIVAAGLADTCEVQIAYAIGVARPVSININTFGTGK--IGED----RIANLVREHFDLRPKGIVQMLDLLR-P-------IYRPTAANGHFGR-T----------------------EDS---F--S-WEKT-DK-A-----EALA----A----DAGVKIAS------------

>Alphaproteobacteria_Minwuia_thermotolerans

---------M-SR------------N--Y-IFTSESVGEGHPDKMADNISDAILDALLAEDP-----KSRSACETMLNTGMAVISGEITTTA-KV--------DYTDVVRQTIRDIGYDD--PR-YGFDADGCAVLVALDKQSPDIAQGV-DE-GTGLD-L----------------DQ-GAGDQGLMFGFACRETEHLMPLPIDLAHKLTRKQAEVRRAG--K-LPWLRPDVKSQVSVRY--E-N--D--R-PSAVDAI-VLSTQH-DDGVD---------HA----TIRE-AVIEEIVKPV-----L-P-G-------R-Y-DA--GDI--TYHINPTGKFVIGGPVGDCGLTGRKIIVDTYGGMGRHGGGAFSGKDPSKVDRSAAYAARYVAKNIVAAELAEMCEVQLSYAIGVSQPVSVAVNTFGTGR--IAED----TISALVREHFDLRPKGIVQMLDLLR-P-------IYRKTAAYGHFGR-D----------------------DAD---F--T-WEKT-DR-A-----AALR----Q----DAGVAAAE------------

>Alphaproteobacteria_Magnetococcus_marinus

---------M-PR------------N--Y-LFSSESVSEGHPDKMADQISDAILDALLAQDP-----LSRVACETMVSTGFCTIAGEITTKA-VI--------DYQKIARETINAIGYTS--ADTMGYSGDTAAIFVALDKQSVDIAQGV-NE-GEGID-L----------------DQ-GAGDQGIMFGYACTETDVLMPMPIYYAHRLMEKHAELRKSG--E-LAWARPDAKSQVTVRY--V-D--D--K-PVSVEAV-VISSQH-SPDVD---------HD----TIEK-EIIEKVIRAV-----I-P-A-------E-L-LH--EGT--QYFINPTGRFVIGGPVGDAGVTGRKIIVDTYGGMGSHGGGAFSGKDPSKVDRSSAYMGRYVAKNIVAAGLAEKCEIQVAYAIGVSQPMSVMVDTFGTGK--LDDE----AITELVKKHFDLRPKAIVQQLDLLR-P-------IYKKSAAYGHFGR-E----------------------LPE---F--T-WERT-DK-A-----AALR----A----DAGL----------------

>Alphaproteobacteria_Magnetofaba_australis

---------M-SR------------N--Y-LFTSESVSEGHPDKVADQVSDAILDALLAQDP-----KSRVACETMVSTGFVTLAGEITTNA-VV--------DYQNVARDTIREIGYTS--CDAMGFDADSCAVFVSLDKQSVDIAQGV-NE-GEGID-L----------------DQ-GAGDQGLMFGFACNETEHLMPAPIYYAHRLVERQAHVRKNG--A-LSWLRPDAKSQVSFRY--E-N--G--K-PVAIDAV-VLSTQH-GPEIE---------HK----TLTE-AVMEEIIKPI-----L-P-A-------E-M-LH--AGT--EYHINPTGRFVIGGPVGDCGLTGRKIIVDTYGGMGRHGGGAFSGKDPSKVDRSAAYMGRYVAKNIVAAGLAERCEVQVAYAIGVSKPMSLMVETFGTGA--VTDE----RIAELVKEHFDLRAGSIVKQLDLLR-P-------IYKPTAAYGHFGR-------------------------PG---F--S-WENT-DK-A-----DALR----Q----AAGL----------------

>Alphaproteobacteria_Jannaschia_seohaensis

-----M---T-V-------------RKTH-SFTSESVSEGHPDKVCDRISDAVLDALLAEEP-----EARVACETFATTNRVVIGGEI-GLA-DQSNLAHQMERIEQIARDCIKDIGYEQ-----DKFHHATCEVTNLLHEQSAHIAQGV-T--AAENR------------------DE-GAGDQGIMFGYACDETPELMPAPILYAHAILRRLAEVRKNG--T-EPSLGPDAKSQLTVRY--E-N--G--R-PVGVSSV-VLSTQHLDEDMT---------SD----DVRS--VVEPYIRET-----L-P---------EGW-LT--EAT--EWWVNPTGKFVIGGPDGDAGLTGRKIIVDTYGGAAPHGGGAFSGKDPTKVDRSAAYAARYLAKNIVAAGLASRATLQLSYAIGVAKPLSIYVDTHGTGE--VDEA----EIERAVSQVMDLTPRGIRRHLQLNR-P-------IYQRTAAYGHFGR-AP---------------------DAD-GGF--S-WEKT-D-LA-----EALK----A----AV------------------

>Alphaproteobacteria_Nioella_nitratireducens

---------M-S-------------RNHY-IFTSESVSEGHPDKVCDRISDAVLDALLAEEP-----EARVACETFATTNRVVIGGEI-GLS-DQDKLHYYMRNIDQITRECIKDIGYEQ-----DKFHWNTVEITNLLHEQSAHIAQGV-T--GKDNR------------------DE-GAGDQGIMFGFATNETPDLMPAPIQYAHAILRRLAEVRKSG--A-EPQLGPDAKSQLSVRY--E-N--G--K-PVEVTSV-VLSTQHFDETLT---------SD----DVRV--IVEPYIRGC-----L-P---------EGW-LT--AAT--EWHVNPTGKFVIGGPDGDAGLTGRKIIVDTYGGAAPHGGGAFSGKDPTKVDRSAAYAARYLAKNVVAAGMAERCTLQLSYAIGVAKPLSIYIDTHGTGQ--VEEA----AIEKAVAQCMDLTPRGIRTHLELNK-P-------IFQRTAAYGHFGR-KP---------------------EAD-GGF--S-WEKT-D-LV-----EALK----R----AV------------------

>Alphaproteobacteria_Pseudodonghicola_xiamenensis

---------M-S-------------RKNY-TFTSESVSEGHPDKVCDRISDAVLDAFLAEEP-----EARVACETFATSGMVVIGGEV-GLK-DEDLLKKYMGSIGQIARDCIKDIGYEQ-----EKFHWNTCHVLNFLHEQSAHIAQGV-D--AADNK------------------DE-GAGDQGIMFGYATDETPALMPAPIQYSHAILRRLAEVRKSG--V-EPTLRPDAKSQLSVVY--E-D--G--K-PVRVSSI-VLSTQHESESQT---------SD----DIRQ--IVEPYVREV-----L-P---------SGW-IT--EET--EWWVNPTGTFVIGGPDGDAGLTGRKIIVDTYGGAAPHGGGAFSGKDPTKVDRSAAYAARYLAKNVVAAGLAHKCTLQLSYAIGVSKPLSIYVDTHGTGA--VPDE----VIEKAVAQSMDLSPRGIRQHLQLNK-P-------IYQRTAAYGHFGR-DP---------------------EAD-GGF--S-WEKT-D-LV-----ETLK----K----AV------------------

>Alphaproteobacteria_Sulfitobacter_donghicola

---------M-S-------------RKSY-VFTSESVSEGHPDKVCDRISDAVLDAFLSEEP-----EARVAAETFATTNRVVIGGEV-GLS-DRSKLHEYMGSIEQIARDCIKDIGYEQ-----DKFHHETVEVTNLLHEQSAHIAQGV-D--SATDK------------------DE-GAGDQGIMFGYATDETDMLMPAPIHYSHAILRRLAEVRKDG--T-EPTLRPDAKSQLSVRY--E-N--G--K-PVGVSSI-VLSTQHAHESQT---------SS----DIRD--IVEPYIREV-----L-P---------EGW-IT--ADT--EWWVNPTGTFVIGGPDGDAGLTGRKIIVDTYGGAAPHGGGAFSGKDPTKVDRSAAYVARYLAKNVVAAGMAARCTIQLSYAIGVSKPLSIYCDTFGTGD--VDPE----AIEKAIGQVIDLTPRGIRNHLQLNR-P-------IYQRTAAYGHFGR-TP---------------------DAD-GGF--S-WERT-D-LV-----DALK----A----AV------------------

>Alphaproteobacteria_Roseivivax_isoporae

---------M-S-------------RKSY-YFTSESVSEGHPDKVCDRISDAVLDALLAEEP-----LARVACETFATTNRVVIGGEV-GLA-DQHKLKDYMERIPEIARACIKDIGYEQ-----DAFHHATCDISNYLHEQSAHIAQGV-T--GQAGR------------------DE-GAGDQGIMFGYATNETEALMPAPILFAHQILLKLAEARKSG--K-EPLLRPDAKSQLTLRY--E-D--G--K-PVGVTQI-VLSTQHADESQS---------SA----DIRA--IVEPYIREV-----L-P---------AEW-FT--DTT--EWWVNPTGTFVIGGPDGDAGLTGRKIIVDTYGGAAPHGGGAFSGKDPSKVDRSAAYAARYLAKNVVAAGLAERCTIQLSYAIGVARPLSIYCDTHGTDR--VDPE----AIEAAIPRVMDLTPRGIRTHLDLNR-P-------IYERTAAYGHFGR-EP---------------------DAD-GGF--S-WERT-D-LA-----EALQ----K----AL------------------

>Alphaproteobacteria_Defluviimonas_denitrificans

---------M-S-------------RQNY-VFTSESVSEGHPDKVCDRISDAVLDAFLTEDP-----HSRVACETFATTDRVVIGGEVRGPG-A------VIERVEEIARECVKDIGYEQ-----DGFHWANLKVDSYLHAQSADIAQGV-D--ASGNK------------------DE-GAGDQGIMFGYAVDETPELMPAPILYAHAMLRRLAEVRKNG--T-EPKLGPDAKSQLSIRY--E-G--G--K-PVGVTSL-VLSTQHIDPALS---------SA----DVRA--IVEPHIRDV-----L-P---------EGW-LT--AET--EWHVNPTGKFVIGGPDGDAGLTGRKIIVDTYGGAAPHGGGAFSGKDPTKVDRSAAYAARYLAKNVVAAGLATRCTLQLSYAIGVAKPLSIYVDTHGTGM--VDEA----AIERAVAQAMDLTPRGIRTHLGLNK-P-------IYQRTAAYGHFGR-AP---------------------EAD-GGF--S-WEKT-D-LA-----EVLK----K----SV------------------

>Alphaproteobacteria_Actibacterium_pelagium

---------M-S-------------RQNY-IFTSESVSEGHPDKVCDRISDAVLDAFLSEDP-----YARVACETFATTDRVVIGGEVRGPG-S------VIERVEDIARACVKDIGYEQ-----KGFHWANMNVDNYLHAQSADIAQGV-D--AAANK------------------DE-GAGDQGIMFGYAVNETDALMPAPILYSHAILRRLAEVRKSG--V-EPKLGPDAKSQLSVRY--E-N--G--K-PVEVTSI-VLSTQHLDPELS---------SG----DVRE--IVEPYIREE-----L-P---------EGW-IT--GNT--VWHVNPTGKFVIGGPDGDAGLTGRKIIVDTYGGAAPHGGGAFSGKDPTKVDRSAAYASRYLAKNIVAAGLADRCCLQLSYAIGVAKPLSIYVDTYGTGK--VPEA----EIEKAVAQVMDLTPRGIREHLQLNK-P-------IFERTAAYGHFGR-AP---------------------DAD-GGF--S-WEAT-D-LV-----DTLK----K----AI------------------

>Alphaproteobacteria_Aliiroseovarius_crassostreae

---------M-T-------------RKSH-VFTSESVSEGHPDKVCDRISDAVLDAFLAEDP-----HSRVACETFATTDRVVIGGEVRGPA-S------VIERVEEIARDCVKDIGYEQ-----KGFHWANMKVSNYLHAQSADIAQGV-D--ASEDK------------------DE-GAGDQGIMFGYAAVETDALMPAPVHYSHAILRRLAEVRKNG--T-ESWMGPDSKSQLSVRY--E-N--D--L-PVEVTSL-VLSTQHLDESMT---------PN----DVRD--AVAPYIRDV-----L-P---------DGW-LT--DNT--VWHVNPTGKFVIGGPDGDAGLTGRKIIVDTYGGAAPHGGGAFSGKDPTKVDRSAAYASRYLAKNIVAAGLAKRCTLQLSYAIGVARPLSILVDTHRTGE--VDES----AIEKAVMEAMDLTPRGIREHLGLNK-P-------IYQRTAAYGHFGR-AP---------------------SDD-GGF--S-WERT-D-LV-----DALK----N----AL------------------

>Alphaproteobacteria_Rhodovulum_sulfidophilum

---------M-SN------------RGDY-IFASESVSEGHPDKVCDRISDAVLDAFLSADP-----EARVACETFATTDRVVVGGEVRGPG-D------VLARVEDIVRDCVKDIGYEQ-----KGFHWATLHVHNYIHGQSEHIAQGV-D--AGDGK------------------EE-GAGDQGIMFGYAVNETPELMPAPIHYAHAILRRLAEVRKDG--T-EARLGPDAKSQLSLRY--E-N--G--V-PVEVSSL-VLSTQHLDEDMT---------SA----DVRA--MVEPYIREV-----L-P---------EGW-LT--DAT--AWHVNPTGRFVIGGPDGDAGLTGRKIIVDTYGGAAPHGGGAFSGKDPTKVDRSAAYAARYLAKNVVAAGLAHRCVIQLSYAIGVAKPLSIYADTFGTGE--VPEA----VIEAALTNVMDLTPRGIRTHLGLNK-A-------IYQRTSAYGHFGR-AA---------------------DAD-GGF--S-WERT-D-LT-----EALK----K----AV------------------

>Alphaproteobacteria_Roseovarius_gaetbuli

---------M-A-------------RQNY-LFTSESVSEGHPDKVCDRISDAILDAFLAEEA-----NARVACETFATTNFVVVGGEV-GLS-DPQRLKTFMGGVEAIVRECVRDIGYEQ-----EKFHWKHLSFQNLLHPQSAHIAQGV-D-------------------------RD-GAGDQGIMFGYAVDETKELMPAPILYAHAILKRLAEARKSG--A-EPTLGPDAKSQLTLRY--E-D--G--R-PVEVTQL-VLSTQHTDESQT---------SD----DIRA--IVEPYIKEV-----L-P---------VGW-LT--EKT--EWWVNPTGTFVIGGPDGDAGLTGRKIIVDTYGGAAPHGGGAFSGKDPTKVDRSAAYAARYVAKNVVAAGLAARCTLQLSYAIGVAKPLSIYVDTHGTGQ--VEDM----RIEKAVEACMDLTPRGIREHLDLCR-P-------IYSRTAAYGHFGR-AP---------------------EAD-GGF--S-WERT-D-LI-----EALK----K----AV------------------

>Alphaproteobacteria_Rubellimicrobium_thermophilum

---------M-S-------------RLDY-VFTSESVSEGHPDKLCDRVSDAVLDAFLAEDP-----RARVACEAFATTNFLAIGGEV-GLT-DRAKMKDLVDRVEDIAREAIRDIGYEQ-----EKFHWRTCEIRNLLHEQSAHIAQGV-T--GEKGR------------------EE-GAGDQGIMFGYATRETPELMPAPIQYAHAILRRLAEARKSG--A-EPLLRPDAKAQLTLRY--A-G--G--R-PVEVTSI-VVSTQHESEAQS---------SD----DIRA--IVEPYVREV-----L-P---------EGW-IT--PAT--KWWVNPTGAFVIGGPDGDTGLTGRKIIVDTYGGAAPHGGGAFSGKDPTKVDRSAAYAARYLAKNIVAAGLADRCTIQLSYAIGVARPLSIYADLHGTGA--VDAA----AIERAIPEVMDLTPRGIREHLGLGR-P-------IYARTAAYGHFGR-TP---------------------DAD-GGF--S-WERT-D-LA-----DSLR----R----AVARAA--------------

>Alphaproteobacteria_Cohaesibacter_gelatinilyticus

---------M-A-------------RKEF-LFTSESVSEGHPDKICDRISDAIVDKFLEQDP-----YSRIAVETMATTNQVTLAGEVRGPS-SINA-----EVMSETARRVIKDIGYEQ-----DGFHWKNANIDVHVHEQSADIAQGV-D--EAEGK------------------DE-GAGDQGIMFGYAVNETPELMPAPIFYAHKILRDLAEARHSG--A-EPALGPDAKSQLTLRY--V-E--G--K-PVEVSSL-VLSTQHLDENLS---------SD----DIRQ--IVAPYIEKA-----L-P---------EGW-LT--DQT--VWHVNPTGKFVIGGPDGDAGLTGRKIIVDTYGGAAPHGGGAFSGKDPTKVDRSAAYAARYLAKNVVAAGLADRCSIQLSYAIGVSEPLSLYVDSYGTGK--VRDS----QLEKALWNCMSLTPRGIRTHLQLNR-P-------IYERTAAYGHFGR-QP---------------------NED-GGF--S-WERT-D-LV-----DKII----A----ELA-----------------

>Alphaproteobacteria_Pannonibacter_carbonis

---------M-A-------------RQDY-LFTSESVSEGHPDKVCDRISDAVVDAYLREMP-----EARVACETLATTNRIVIAGETRGPA-TITK-----DYIAHLARLAVKDIGYSQ-----EGFHWENCDISVYLHGQSAHIAQGV-D--AAGNK------------------DE-GAGDQGIMFGYACRETPEFMPAPIYYAHKMLRILAEARKSG--R-EPVLGPDAKSQVTVRY--V-N--G--K-PVGVTSI-VLSTQHLDATLS---------SS----DVRA--IVEPYIRSA-----L-P---------DGW-ID--PST--VWHVNPTGAFVIGGPDGDCGLTGRKIIVDTYGGAAPHGGGAFSGKDPTKVDRSAAYAARYLAKNVVAAELADRCTIQLSYAIGVAEPLSVYVDLHGTGT--CDEA----KLEKTLRDVMRLTPRGIREHLKLNA-P-------IYERTSAYGHFGR-EP---------------------DAD-GGF--T-WEKI-D-LV-----SALR----S----ALA-----------------

>Alphaproteobacteria_Neomegalonema_perideroedes

---------M---------------RKDF-LFTSESVSEGHPDKICDQIADSIIDVILAREP-----EARTAVEVAATTNHVVLLGEVRAHV-KR-------EEMIATARETIRDIGYEQ-----KGFHWDSCQIECLVHQQSADIAQGV-D--SSGAK------------------DE-GAGDQGIMFGYAADETPELMPAPILYAHKMLRLISEARRDG--S-AAMLGPDAKSQLTVEY--R-D--G--K-PVRAHTI-VLSTQHLDEDMT---------SA----DVRA--VVEPYVRKA-----L-P---------EGW-ID--SGT--VWHVNPTGRFVIGGPDGDAGLTGRKIIVDTYGGAAPHGGGAFSGKDPTKVDRSAAYAARYVAKNVVAAGLARRCLVQLAYAIGVAEPLSIYVDDYGTGE--VSPE----KIEAAIRASMSLTPRGIRTHLGLNK-P-------IYRRTAAYGHFGR-AP---------------------DAD-GGF--S-WEKT-D-LA-----EALR----K----AV------------------

>Alphaproteobacteria_Emcibacter_congregatus

---------M-S-------------RANY-LFTSESVSEGHPDKVADCISDSIVDLFLAADP-----ESRVAVETLTTTNRVVLAGEVRGPA-SVDA-----HAMEAAARRVVKKIGYEQ-----DGFHWENMAVDVHVHEQSADIAMGV-D--SGADK------------------DE-GAGDQGIMFGFATNETDAYMPAPIYFSHKILESLADVRHGG--L--TELGPDSKSQVTLRY--E-N--G--V-PVAATSV-VVSTQHSAET-S---------QD----QVKE--IVRAHIEKN-----L-P---------QGW-MS--SET--ELYINPTGNFVIGGPDGDAGLTGRKIIVDTYGGAAPHGGGAFSGKDPTKVDRSAAYATRYLAKNVVAAGLADRCTIQLSYAIGVSHPLSLYVDFHGTGK--VDPA----VLEKLLYEVMDLSPRGIREHLNLNR-P-------IYERTAAYGHFGR-TP---------------------DAD-GGF--S-WEKL-D-LV-----DRLK----S----QF------------------

>Alphaproteobacteria_Tistrella_mobilis

---------M-A-------------RSNY-LFTSESVSEGHPDKICDRISDAVLDHLLAADP-----YSRVACETLATTNRVVIAGEIRSHG-DTAP-----ATIERVARDVIRDIGYEQ-----DGFHWNTAKVEVLLHEQSADIAMGV-D--AAGNK------------------DE-GAGDQGIMFGYACRETDALMPAPIHYAHTILRNLRTARRSG--D-IAVLGPDAKSQVTLEY--V-D--G--K-PVRATSI-VLSTQHLASA-S---------QE----EVRA--AVLPVIEAS-----L-P---------AGW-MC--DDA--GLYINPTGNFVIGGPDGDAGLTGRKIIVDTYGGAAPHGGGAFSGKDPTKVDRSAAYAARYLAKNVVAAGLADRCTIQLAYAIGVSHPLSVYVDTYGTGQ--VDEA----KLEKALREAMNLTPRGIREHLKLNR-P-------IFGRTAAYGHFGR-DP---------------------EAD-GGF--S-WEKT-D-LA-----ETIK----Q----LVL-----------------

>Alphaproteobacteria_Terasakiella_pusilla

---------M-A-------------LDKY-LFTSESVSEGHPDKVADRISDSIIDAFLELDP-----YSRVAVETMVTTNFVALAGEVRGPD-EMNH-----DRFKEIAREAIREIGYEQ-----RGFHWKDCEITSQIHSQSADIAQGV-DEGSGTDK------------------EE-GAGDQGIMFGYACNETPELMPAPIMYAHKILRRMAEDRHSG--K-VKGFGPDSKSQVTLAY--E-N--G--K-PVRATSL-VVSTQH-DESLS---------LD----DVRE--LVLPYFEEV-----L-P---------EGW-LP--SDD--EIYVNPTGRFVIGGPDGDAGLTGRKIIVDTYGGAAPHGGGAFSGKDPTKVDRSAAYAARYLAKNIVAAGLADKCTIQLSYAIGISKPLSVYVDTAGTAQ--VDEL----KLAKIASEIMNLSPRGIREHLKLNA-P-------IYKETSAYGHFGR-TP---------------------TEQ-GHF--S-WEKT-D-LV-----DELK----R----QF------------------

>Alphaproteobacteria_Komagataeibacter_pomaceti

---------M-RN------------KGDF-LFTSESVSEGHPDKVADRISDTVLDAFLTADG-----ESRVACETLVTTNRIVLAGEVRGPA-SVTP-----ELLIEGAREAVRDIGYDQ-----PGFSWKNAEVEYYLHAQSADIAVGV-D--SADNK------------------DE-GAGDQGIMFGFATRETDSLMPAPLHYAHGILHRIRDLRKAGDPR-AAALQPDAKSQVTLRY--V-D--G--K-PVGATSV-VISTQH-VEGAS---------QE----TIRE--ELRGVVKDV-----L-P---------DGW-MC--PED--EFYVNPTGVFVIGGPDGDCGLTGRKIIVDTYGGAAPHGGGAFSGKDPTKVDRSAAYACRYLAKNIVAAGLADACTLQISYAIGVSHPLSVYVDLAETGK-DVDEE----KLGRVLREVMDLSPRGIRKHLRLNR-P-------IYTQTSAYGHFGR-TP---------------------DAAKDDF--T-WEQT-D-LV-----DALR----S----AFNR----------------

>Alphaproteobacteria_Roseomonas_rhizosphaerae

---------M-RD------------KGEY-LFTSESVSEGHPDKVADRISDTVLDAFLEADP-----YARVACETLVTTNRIILAGETRGPA-SVTP-----ELLMHLARMAVHDIGYDQ-----EGFSWKNAEVACHLHAQSAHIAQGV-D--AAGEK------------------DE-GAGDQGIMFGYACTETPDLMPAPLYYAHLILRRIAERRRTNDKS-VAGLLPDAKSQVTLRY--V-D--G--K-PVGVASV-VVSTQH-EEGMT---------QE----QVRE--LVRPIVETS-----L-P---------AGW-SV--PEE--EFYVNPTGTFVIGGPDGDCGLTGRKIIVDTYGGAAPHGGGAFSGKDPTKVDRSAAYACRYLAKNVVAAGLAERCTIQVSYAIGVSKPLSVYFDLHGTGR-DIDEV----KLAKVVNEMVNLSPRGIREHLHLNR-A-------IYAPTSAYGHFGR-TP---------------------DLEKGTF--T-WEKT-D-LA-----AELK----R----AFGR----------------

>Alphaproteobacteria_Dankookia_rubra

---------M-RD------------KGEY-LFTSESVSEGHPDKVADRISDTVIDAFLAADP-----YARVACETLVTTNRIILAGEVRGPD-GVM------QGLEEKVRAAVKDIGYDQ-----EGFSWRNAEFQNYLHGQSADIAVGV-D--AAGNK------------------DE-GAGDQGIMFGYATNETPDLMPAPLLYAHKILLKMAQYRKAGDAR-AKGLEPDAKSQVTLRY--V-D--G--K-PVGVTCV-VVSTQH-AEGME---------QA----EIKE--LIRPIVKDV-----L-P---------AGW-VV--PEA--EFYVNPTGKFVIGGPDGDTGLTGRKIIVDTYGGAAPHGGGAFSGKDPTKVDRSAAYAARYVAKNVVAAGLADRCTIQVSYAIGVSKPLSVYFDLHGTGK-DIDEA----KLAAVVQDVVNLSPRGIREMLRMNR-P-------IYAVTSAYGHFGQ-AP---------------------DLKADTF--T-WERT-DVLA-----PELK----R----AFGR----------------

>Alphaproteobacteria_Salinihabitans_flavidus

---------M-S-------------DKTW-LFTSESVSEGHPDKVCDRISDAVLDAYLSADP-----QARVACETLATTDHVTIAGEVRGPE-HVR------QHVEELARQAIREIGYQQ-----KGFSWKTCAVNNLLHEQSADIAMGV-D--EGDKG------------------EE-GAGDQGIMFGYACTETDELMPAPIQFAHRILRRMAEDRKSG--A-QPNFGPDAKSQVTLLY--R-N--G--K-PVGLDTV-VVSTQH-LEGVS---------QD----EVRE--MVKPYIEKA-----F-P---------DDW-TL--PDD--KLIVNPTGKFVIGGPDGDAGLTGRKIIVDTYGGAAPHGGGAFSGKDPTKVDRSAAYVARYLAKNVVAAGLAEKCQIQLAYAIGMPEPVALYVDTLGTGE--VSEE----QLSKRLREMVRLTPRGIREHLDLLR-P-------IYARTAAYGHFGR-EP---------------------DAD-GGF--S-WEKT-D-LA-----AELA----R----SFGA---TAAAE--------

>Alphaproteobacteria_Roseivivax_jejudonensis

---------M-S--------------KTW-LFTSESVSEGHPDKVCDRISDTILDAYLAEAP-----DARVAVETLATTNHVTIAGEVRGPD-SVR------KAVEEHVRAAIRDIGYDQ-----LGFDWNTVEVINRLHAQSGDIAMGV-D--EGDKG------------------EE-GAGDQGIMFGYACRETEELMPAPIQFAHRILRMMAEDRKSG--V-RPEFGPDAKSQVTLKY--Q-D--G--K-PVGIDAI-VVSTQH-DEDVS---------RD----EVRE--LVKPYLSKA-----S-L---------EGW-TV--TDD--RLFVNPTGRFVLGGPEGDAGLTGRKIIVDTYGGAAPHGGGAFSGKDPTKVDRSAAYAARYLAKNVVAAELADRCQIQLAYAIGVPEPVAVYVETFGTAR--VDEV----KLGETLRQMVRLTPRGIREQLDLQR-P-------IYSRTAAYGHFGR-PV---------------------EDD-GGF--G-WERT-D-LA-----RDLA----S----TFGH---KIAAE--------

>Alphaproteobacteria_Sphingomonas_paucimobilis

-----------MS------------ER-K-LFTSESVSEGHPDKIADQISDAILDAILAKDP-----EAHVAAETAVYTGSVHVFGEISTNA-YV--------DINRVVRDTIAEIGYTN--TE-YGFSAETVGVHPSLVEQSPDIAQGV-NE-ALEVRGN--A-DQ-----DPLD-LI-GAGDQGLMFGFAVDETEELMPLPIALSHKLVRRLAELRKSG--E-ISYLRPDAKSQVTVEYD-E-N--D--R-PVRVDTV-VISTQH-DPEAT---------NE----QIHQ-DVIDKVIKEV-----I-P-S-------S-Y-LD--DKT--KFFINPTGRFVIGGPQGDSGLTGRKIIVDTYGGYSRHGGGAFSGKDATKVDRSASYAARYIAKNIVAADLAKKAEVQLAYAIGVAQPVSVRIDTFGTGT--VAES----QLEKAARQIFDLRPAGIIQMLDLKR-P-------IYRQTSAYGHMGR-T----------------------DID---L--P-WERL-DK-V-----DALK----E----AVK-----------------

>Alphaproteobacteria_Puniceibacterium_sediminis

---------M-F-------------TNTY-LFSSESVSEGHPDKICDMISDAILDSYLAQDP-----DARVACETLATTGHVTIAGEVRTRA-KDH------PPAADIARNVIRSIGYDQ-----DAFSWRTVDIVNRLHTQSADIARGV-D--DAGNG------------------EE-GAGDQGIMFGYACRETPALMPAPIVYAHGILQNLATDRKAG--I-LPQLGPDAKSQVTLRY--R-D--G--K-PVGVETV-VLSTQH-IEGLS---------AA----DIKD--IVAPYVMDI-----F-P---------KGW-AI--DPN--RLLVNPTGNFVIGGPDGDTGLTGRKIIVDTYGGAAPHGGGAFSGKDPTKVDRSAAYVARYLAKNVVEAGLADTCLIQLAYAIGVAEPVSVYLDLKGTGR--VDPV----RLAKVLREMVPLTPRGIRSHLDLAR-P-------IYAPTASYGHFGR-SP---------------------RDS-GSF--S-WEST-D-IA-----DQLA----R----AFGAIAQPVSAQSGH-----

>Alphaproteobacteria_Acidiphilium_cryptum

---------M-RD------------KGDY-LFTSESVSEGHPDKVADRISDTVLDAFLAADP-----YARVACETLVTTNRIVLAGETRGPS-TITR-----EYLAHLARLAVHDIGYEQ-----EGFSWRDAKIDVLLHEQSVDIAVGV-D--AAGNK------------------DE-GAGDQGIMFGYACSETDALMPAPIYYAHLILRRMTELRKIGDAR-AAGLLPDAKSQVTLRY--A-D--G--K-PVGTTSI-VVSTQH-EDGMS---------QD----EIKA--MLRPLVTEL-----L-P---------DGW-MC--PDD--QFYVNPTGKFVIGGPDGDCGLTGRKIIVDTYGGAAPHGGGAFSGKDPTKVDRSAAYMCRYLAKNVVAAGLATRCTIQVSYAIGVSHPLSVYVDMHGTER-DVDHA----RLETVLRELVNLTPRGIREHLHLNR-P-------IYVPTSAYGHFGR-EP---------------------DDRLGTF--T-WEKT-D-LA-----PALK----A----AFGR----------------

>Alphaproteobacteria_Rhodobacter_sphaeroides

--------------------------MNY-VFTSESVSEGHPDKLCDRVSDAVLDTFLAEEP-----TARVACETFATTGRVVVGGEV-GLS-DPQK-DEFMERVDGIVRDCVKDIGYEQ-----QEFHWRTIEVQNFLHRQSAHIAQGV-D-------------------------KD-GAGDQGIMFGYACRETPELMPAPIQYSHAILRRLAEVRKSG--Q-EPDLRPDAKSQLSLRY--E-N--G--K-PVEVRSI-VLSTQHAHEEQT---------SD----DIRA--IVEPYIREV-----L-P---------EGW-IT--EAT--EWWVNPTGTFVIGGPDGDAGLTGRKIIVDTYGGAAPHGGGAFSGKDPTKVDRSAAYAARYLAKNVVAAGLAERCTLQVSYAIGVAKPLSIYVDTHGTGQ--VDAA----QIEKAVADCMDLTPRGIREHLNLCR-P-------IYARTSAYGHFGR--P---------------------EAD-GGF--S-WERT-D-LT-----DALL----K----AV------------------

>Alphaproteobacteria_Rickettsia_typhi

------------M------------EN-F-VFTSESVSEGHPDKIADQISDAVLDEILKHDP-----NGRVACETFVTTGLVLVGGEITTNT-YV--------DIEQVVRNKIHEIGYNN--SPSYGFDGSCCAVISSIVKQSPDIAMGI-DN-ANE---E----------------EI-GAGDQEMVFGYACNETKSLMPAPIYYAHLLMKRQAYLRKQN--I-LSWLRPDAKSQVTLRY--E-N--N--K-PIVIDSV-VLSTQH-HPEIQ---------QK----DLIE-AVIEEIIKPT-----L-P-T-------H-L-LH--KDT--KYLINPTGRFVIGGPVADCGLTGRKIIVDSYGGMAKHGGGCFSGKDPTKIDRSAAYMARYIAKNIVGSGLADRCEIQISYAIGVASPVSVYAETFGTSK--LSNE----QTTKLITEHFDMRPGKIIKNLKLHT-Q-------CYQKTASYGHFGR-D----------------------ENF---F--T-WEQL-DK-V-----DIFK----S----I-------------------

>Betaproteobacteria_Thiomonas_bhubaneswarensis

---------M-AN------------S--F-LFTSESVSEGHPDKVADQISDAILDANLAQDP-----MSRVAAETLANTGLIVLAGEITTNA-VV--------DYIQVARDTLKRIGYDD--AD-FGIDHKSCAVLVAYDKQSPDIAQGV-NK-AHDDE-L----------------DM-GAGDQGLMFGYACDETPDLMPAPIYYAHRLVERQSQIRRDG--R-LSWLRPDAKSQITFRY--E-N--G--Y-PVAIDTV-VLSTQH-APDIA---------LA----DLRE-AVIEHIIKPV-----L-P-A-------E-F-MR--GEV--KYLINPTGRFVVGGPQGDCGLTGRKIIVDTYGGAAPHGGGAFSGKDPTKVDRSAAYAARYVAKNIVAAGLAKKCLVQISYAIGVAQPTSIMVTTQGTGV--IDDA----KLEQLVRRHFDLRPRGIIEMLDLRR-P-------IYAKTAAYGHFGR-D----------------------EPE---F--S-WEAT-DR-A-----AKLR----D----DAGLGALQA-MAAA------

>Betaproteobacteria_Sulfurisoma_sediminicola

---------M-SN------------E--Y-LFTSESVSEGHPDKVADQISDAILDAILAQDK-----HSRVAAETLCNTGLVVLAGEITTSA-NV--------DYIGVARDTIKRIGYDN--TE-YGIDYKGCAVLVAYDKQSPDIAQGV-NK-AYDDD-L----------------GQ-GAGDQGLMFGYACDETPSLMPLPIYLAHRLMERQAMLRKDG--R-LPWLRPDAKSQVTVRY--E-N--G--K-PAAIDTV-VISTQH-APEMS---------LE----DIRE-ATIEEIIKPI-----L-P-K-------E-L-VK--GEI--KYLVNPTGRFVVGGPQGDCGLTGRKIIVDTYGGAAPHGGGAFSGKDPSKVDRSAAYAGRYVAKNIVAAGLASRCLIQVSYAIGVAQPTSVWVTTYGTGK--VSDE----TIAELVKKHFDLRPKGIVNMLDLLR-P-------IYQKTAAYGHFGR-D----------------------EPE---F--T-WENT-DK-A-----ALLR----N----DAGL----------------

>Betaproteobacteria_Parvibium_lacunae

---------M-SN------------D--F-LFTSESVSEGHPDKVADQISDAVIDAIFAQDK-----YARVAAETLCNTGLVVLAGEITTTA-NV--------DYIQVARDTIKRIGYDN--TD-YGIDYKGCAVLVAYDKQSPDIAQGV-DH-ASDDY-L----------------NT-GAGDQGLMFGYACDETPELMPAPIYYAHRLVERQSLLRKDG--R-LPWLRPDAKSQVTFRY--V-D--G--K-PVSIDTV-VLSTQH-APDIT---------QE----QIRE-AVIEEIIKPA-----L-P-S-------H-F-LQ---DT--KYLVNPTGRFVIGGPQGDCGLTGRKIIVDTYGGACPHGGGAFSGKDPTKVDRSAAYAARYVAKNIVAAGLAKQCQIQVSYAIGVAKPINITVYTEGTGV--VSDE----KLAALVNEHFDLRPKGIIQMLDLLR-P-------IYSKTAAYGHFGR-E----------------------EPE---F--S-WERT-DK-A-----AALR----A----AAGL----------------

>Betaproteobacteria_Collimonas_pratensis

---------M-AH------------E--Y-LFTSESVSEGHPDKVADQISDAILDAIFTQDP-----HARVAAETLCNTGLVVLAGEVTTFA-NV--------DYIAVARETIKRIGYDN--AD-YGIDYKSCSVLVGYDKQSPDIAQGV-DE-GKGLD-L----------------DQ-GAGDQGLMFGYACDETPELMPAAIYYAHRIVERQSQLRKDG--R-LPWLRPDAKSQVTLKY--V-D--G--I-PVAIDTV-VLSTQH-APEMQ---------HK----AIEE-AAIEEIIKPV-----V-P-K-------E-W-LQ---NT--RYLINPTGRFVIGGPQGDCGLTGRKIIVDTYGGAAPHGGGAFSGKDPSKVDRSAAYAGRYVAKNIVAAGLATRCQIQVSYAIGIAKPTSVMVTTFGTGK--ISDE----KLAQLVLEHFDLRPKGIVQMLDLLR-P-------IYQKTAAYGHFGR-E----------------------EPE---F--T-WERT-DK-A-----AALK----A----AAL-----------------

>Betaproteobacteria_Comamonas_badia

---------M-AS------------D--F-LFTSESVSEGHPDKVADQISDAVLDAILQVDP-----RGRVAAETLTNTGLVVLAGEISLRS-DAPA-----PDYIQVARDTIKRIGYDN--TE-YGIDYKGCAVLVAYDKQSNDIAQGV-DQ-ASDDH-L----------------NT-GAGDQGLMFGYACDETDTLMPAPIYYAHRLMERQAQQRKSG--A-LPFLRPDAKSQVTMRY--I-D--G--K-VDSIDTV-VLSTQH-SPDQSETPTKM---KA----SFIE-AVVEQIIKPV-----L-P-R-------E-W-LR---NT--KYLINPTGRFVVGGPQGDCGLTGRKIIVDTYGGSCPHGGGAFSGKDPTKVDRSATYACRYVAKNIVAAGLARQCQIQVAYAIGVARPMNVTVYTEGTGV--IADE----KIAQLVNDHFDLRPKGIIQMLDLQR-P-------IYARTAAYGHFGR-D----------------------EPE---F--T-WERT-DK-A-----QALR----A----AAGL----------------

>Betaproteobacteria_Brachymonas_denitrificans

---------M-AN------------D--F-LFTSESVSEGHPDKVADQISDAILDALLEQDP-----RARVAAETLTNTGLVVLAGEITANA-NV--------DYIQVARDTIKQIGYDD--TA-YGIDYKGCAVMVCYDKQSNDIAQGV-DH-ASDDH-L----------------NT-GAGDQGLMFGYACDETPELMPAPIHYAHRLMERQAELRKNG--T-LPFLRPDAKAQVTMRY--L-D--G--K-PQSVQTV-VISSQH-TPEMSVG-DKM---KP----EFIE-AIVESIIKPV-----I-P-A-------E-M-LV---DT--EFLINPTGRFVVGGPQGDCGLTGRKIIVDTYGGACPHGGGAFSGKDPTKVDRSAAYAARYVAKNIVAAGLARQCQIQVSYAIGVARPINVTVYTEGTGV--IEDH----AIEKLVQDHFDLRPKGIIEMLNLQR-P-------IYTKTAAYGHFGR-S----------------------EPE---F--S-WEQT-DK-A-----ALLR----A----AAGL----------------

>Betaproteobacteria_Brackiella_oedipodis

----MA---Q-NN------------E--F-LFTSESVSEGHPDKVADQISDAVLDAILAEDP-----NARVAAETLCNTGLVVLAGEISTTA-YL--------DYIQIARDTIKRIGYDD--GD-YGIDFKSCAVLVAYDKQSPDIAQGV-DR-SHEEL-L----------------NQ-GAGDQGLMFGYACDETPEFMPAPIWYAHRIVQRQSELRKDG--R-IAWLRPDAKSQVTFRY--V-N--G--V-AQEVDTV-VLSTQH-HPDVT---------QA----TIKE-AAIEEIIKPC-----F-P-E-------G-L-LT--PNT--KYLINPTGKFVIGGPQGDCGLTGRKIIVDTYGGACPHGGGAFSGKDPSKVDRSAAYAARYVAKNIVAAGLAKQCQIQVSYAIGVAEPINITVYTENTGV--IPDE----EIAKLVREHFDLRPRGIIDMLDLMR-P-------LYAKSAAYGHFGR-S----------------------EPE---F--T-WEHT-DK-V-----ELLK----N----AIK-----------------

>Betaproteobacteria_Duodenibacillus_massiliensis

---------M-AS------------D--F-LFTSESVSEGHPDKVADQISDAVLDACLAQDP-----MSRVAAETMCSTGLVVLAGEITTDA-QV--------NYIDLTRDVLKRIGYDN--SE-YGIDHKGCSVLVGYDKQSEDIKQGV-DE-KNHDP-L----------------TQ-GAGDQGLMFGFACDETPTLMPAAIYYAHRLVQQQSIVRRNG--V-MPYLRPDAKSQVTLRY--V-D--G--K-PVAADTI-VLSTQH-SPEWSVG-DHM---KP----EFVE-AVVENIIRPV-----M-P-A-------E-W-LK---NT--RFLVNPTGRFVVGGPQGDCGLTGRKIIVDTYGGACPHGGGAFSGKDPTKVDRSAAYACRYVAKNIVAAGLARQCQIQVSYAIGVAEPMNITVLTNGTGV--IADE----KIAELVRKFFDLRPGGIIQMLDLRR-P-------IYSKTAAYGHFGR-E----------------------EPE---F--T-WEKT-DK-A-----MLLK----E----AAGL----------------

>Betaproteobacteria_Turicimonas_muris

---------M-SK------------NE-Y-LFTSESVSEGHPDKVADQISDAILDAILEQDP-----TARVAAETLCTTGTVMLAGEISTTA-NV--------NYTDIVRATLKRIGYDD--SS-YGIDHKGCAVLVAYDKQSPDIAQGV-NN-AQDDP-L----------------EK-GAGDQGLMFGYACDETDALMPAPIYYAHRLVEQQSLMRRSG--E-MPYLRPDAKSQVTMRY--V-D--G--K-PDSVETV-VLSTQH-APEMSDG-EKM---KP----EFVQ-AVIDKIIRPV-----F-P-P-------E-M-LK---DT--KFLINPTGRFVIGGPQGDCGLTGRKIIVDTYGGACPHGGGAFSGKDPTKVDRSAAYACRYVAKNIVAAGLARQCQIQVAYAIGVAKPVNITVYTEGTGV--IPDE----KIAELVREHFDLRPAGIIKMLDLQR-P-------IYSKTAAYGHFGR-E----------------------EPE---F--T-WERT-DK-A-----AELR----K----AAGLDK--------------

>Betaproteobacteria_Dakarella_massiliensis

---------M-GS------------N--Y-LFTSESVSEGHPDKVADQISDAVLDAILEQDP-----YGRVAAETLCATGLVVLAGEITTSA-QV--------DYIGVTRDTLRQIGYDN--TE-YGIDHRGCSVLVGYDKQSADIKQGV-DK-ASDDP-L----------------ET-GAGDQGLMFGYACDETDTLMPAPIYYAHRLMQQQSILRKNG--T-LPFLRPDAKSQLTVRY--V-D--G--R-PQSVDTV-VLSTQH-SPEMSDG-DKM---KP----EFTE-AVIESIIKPV-----L-P-A-------E-W-IT--PET--KFLINPTGRFVVGGPQGDTGLTGRKIIVDTYGGSCPHGGGAFSGKDPTKVDRSAAYACRYVAKNIVAAGLAHRCQVQVAYAIGVARPMNITVHTFGTGA--IPDE----EIPRIVGESFDLRPNGIIRMLDLRR-P-------IYRKTACYGHFGR-E----------------------EPE---F--T-WEKT-DK-V-----EILR----S----KAGL----------------

>Betaproteobacteria_Deefgea_rivuli

-----------MK------------D--F-LFTSESVSEGHPDKVADQISDAILDAIFTQDK-----YARVAAETLVNTGLVVLAGEITTNA-NI--------DYIQIARDTIKRIGYDH--SD-IGFDYKTCAVLVAYDKQSPDIAQGV-NE-GEGID-L----------------DM-GAGDQGLMFGYACDETPQLMPAPIYYSHRLMQRQAELRKDG--R-LPWLRPDAKSQVTLRYDGA-T--G--K-VKEVDTV-VLSTQH-HPDVS---------HV----QLSE-AVIEQIIKPV-----L-P-P-------E-W-IT--ENT--KFLVNPTGRFVIGGPMGDCGLTGRKIIVDTYGGAAPHGGGAFSGKDPSKVDRSAAYAMRYVAKNIVAAGIAKQCLVQVSYAIGVAQPVSIMVDTWDTGV--IPND----QIVDIIKEHFDLRPKGIIKMLDLLR-P-------IYSRTAAYGHFGR-E----------------------EPD---F--T-WERT-DK-V-----EQLK----A----AAGLK---------------

>Betaproteobacteria_Chitinimonas_taiwanensis

-----------MR------------E--F-LFSSESVSEGHPDKVADQISDAILDAILAQDK-----HARVAAETLVNTGLCVLAGEITTHA-NV--------DYIQVARETIRRIGYDS--SE-LGFDYKGCAVLVGYDKQSPDIAQGV-NE-GQGLD-L----------------DQ-GAGDQGLMFGYACDETPTLMPAAIYLSHRLVQRQSELRKDG--R-LPWLRPDAKSQVTLRYDAD-T--G--R-PIGIDTI-VLSTQH-HPDIA---------HK----TLEE-AVIEDIVKPV-----L-P-P-------E-L-MK--GDI--KYFINPTGRFVIGGPQGDCGLTGRKIIVDTYGGAAPHGGGAFSGKDPSKVDRSAAYMGRYIAKNIVAAGIARQCQVQVAYAIGIAKPVSIMVDTWETGK--ISNE----KIVELIERHFDLRPKGIVQTLDLLR-P-------IYSKTAAYGHFGR-E----------------------EPE---F--S-WEAT-DK-A-----EALR----A----DAGL----------------

>Betaproteobacteria_Azoarcus_olearius

---------M-AN------------E--F-LFTSESVSEGHPDKVADQISDGVLDAILAEDP-----KARVACETLVSTGLVVISGEITTSA-HP--------NYREIAQDVVRRIGYDN--SD-IGFDYKSCAVLAAINRQSSDIAQGV-NE-GEGLD-L----------------DQ-GAGDQGLMFGYATNETPSLMPLPIYYAHRIMQRQAEVRKDG--R-LPWLRPDAKSQLTVKY--V-D--G--K-PVAIDTV-VVSTQH-NPEVS---------HA----QISE-AVIEEIIKPV-----L-P-K-------E-L-MQ--GEV--RYLINPTGRFVIGGPHGDCGLTGRKIIVDTYGGAAHHGGGAFSGKDPSKVDRSAAYAGRYVAKNVVAAGLADKCEVQVAYAIGVAKPVSLMVNTFGTGK--VADD----KIVDLIRAHFDLRPKAIIQTLDLLR-P-------IYSRTAAYGHFGR-D----------------------EPE---F--T-WEQT-DK-A-----AALR----A----AAGL----------------

>Betaproteobacteria_Nitrosomonas_cryotolerans

-----------MS------------E--Y-LFTSESVSEGHPDKVSDQISDAVLDAILSQDV-----NARVACETLCSTGLIVLSGEITTHA-SV--------DYNIIARETVKNIGYTS--SE-IGFDSSTCAVLTAFNKQSPDIAQGV-NR-SKEEE-M----------------DQ-GAGDQGLMFGYACDETPQLMPMPIFYAHRLVERQAELRKKG--Q-LAWLRPDAKSQVSVRY--Q-D--G--K-PQRIETV-VISTQH-DPDIS---------QA----ALTE-AVIEEIIKPV-----L-P-K-------E-M-LS--EQI--QYLVNPTGRFVVGGPMGDCGLTGRKIIVDTYGGAAHHGGGAFSGKDPSKVDRSATYAGRYVAKNIVAAGIANKCEIQVAYAIGVARPVSLLVNTFGTGK--ISDE----AIAKLIEQHFDLRPRAIIHTLNLLR-P-------IYAKTAAYGHFGR-D----------------------DTD---F--T-WEAT-DK-V-----AQLR----A----DAGI----------------

>Betaproteobacteria_Derxia_gummosa

---------M-GR------------E--Y-LFTSESVSEGHPDKVADQISDAVLDAILAQDP-----TARVAAETLVTTGLCVLAGEITTSA-KV--------DYAKVAREAIRRIGYND--PE-LRFDADGCSVHVCYGQQSPDIAQGV-DE-GKGLH-L----------------DQ-GAGDQGLMFGYACDETPELMPFPIYYAHRLVERQSQLRRDG--R-LPWLRPDAKSQLTLRY--V-D--G--K-PVSVHTV-VLSTQH-HPSKT---------HK----EIEE-AVIEEIIKPV-----L-P-P-------E-M-LQ---DT--RYLVNPTGAFIIGGPHGDAGLTGRKIIVDTYGGAAPHGGGAFSGKDPTKVDRSAAYAARYVAKNIVAAGLAKQCVVQVSYAIGVARPINITVDTKGTGR--IDDA----KIAELVEANFDLRPKGIIQMLDLPR-P-------IYRKTAAYGHFGR-S----------------------EPE---F--T-WEAT-DK-A-----EILR----N----FL------------------

>Betaproteobacteria_Caballeronia_sordidicola

---------M-SN------------D--Y-FFTSESVSEGHPDKVADQISDAILDAILAQDK-----YSRVAAETLCNTGLVVLAGEITTQA-NV--------DYIQVARDTIKRIGYDN--TE-YGIDYRGCAVLVAYDKQSRDIAQGV-DA-AHDDN-L----------------DQ-GAGDQGLMFGYACDETPELMPLPIHLAHRLVERQANMRRDG--R-LNWLRPDAKSQVTVRY--V-D--G--K-AHSIDTV-VLSTQH-APDIE---------LK----ALRE-AVIEEIIKPV-----L-P-A-------E-L-IK--GDI--KFLVNPTGRFVIGGPQGDCGLTGRKIIVDTYGGAAPHGGGAFSGKDPSKVDRSAAYAGRYVAKNIVAAGLASRCLIQVSYAIGVARPTSVMVNTFGTGR--VSDA----RIQELVLEHFDLRPKGIIQMLDLLR-P-------IYEKSAAYGHFGR------------------------EPE---F--S-WEAT-DK-A-----LLLA----E----AAGTEPVVE-TA--------

>Betaproteobacteria_Nitrosomonas_eutropha

-----------MS------------N--Y-LFTSESVSEGHPDKVADQISDAILDAILQQDP-----HARVACETMCSTGLIVLSGEITTHA-TI--------DYNVIPRDTVREIGYTS--SE-IGFDASTCAVLTAFNKQSPDIALGV-NR-SKEEE-M----------------DQ-GAGDQGLMFGYACDETPQLMPLPIYYAHRLVEQQAKLRKSG--Q-LSWLRPDAKSQVSVRY--I-D--G--K-PKNIETV-VISTQH-DPDIS---------NG----DLFE-GVVEEIIKPV-----L-P-A-------E-M-LS--SDI--RYLVNPTGRFVVGGPMGDCGLTGRKIIVDTYGGTAHHGGGAFSGKDPSKVDRSAAYAARYVAKNIVAAGLARKCEVQVAYAIGVAKPVSLMLETFGTGK--VSDE----KLAELITRNFDLRPRAIIHELNLLR-P-------IYSKTAAYGHFGR-E----------------------EPS---F--T-WEKT-DI-A-----EQLI----A----DAGI----------------

>Betaproteobacteria_Kinetoplastibacterium_galatii

---------M-SD------------ND-F-LFTSESVSEGHPDKIADQISDAVLDAILAVDP-----SARVAAETLCGYGLIVLAGEITTCA-NV--------DYVKIARDTIKSIGYDS--SD-YGMSYDSCTVISSYGKQSSDIAAGI-SS-SNDKNSL----------------DQ-GAGDQGLMFGYACDETTCLMPAPIWYAHRLVQRQSELRKDG--R-LPWLRPDAKSQVTFRY--V-D--G--K-PYSVDTV-VFSTQH-SPDIS---------HK----IIVE-SVIEEIIRPS-----F-P-E-------N-I-IT--NNT--KFLVNPTGRFVIGGPQGDCGITGRKIIVDTYGGACPHGGGAFSGKDPSKVDRSASYVARYIAKNIVASGLARQCQVQISYAIGVSDPINVTVYTEGTGI--IPDR----EISRIVLEVFDLRPKSIIDMLDLLR-P-------IYLKTATYGHFGR-S----------------------EPE---F--T-WEKT-DK-V-----EKIK----N----FI------------------

>Betaproteobacteria_Profftella_armatura

---------M-PN------------D--Y-LFTSESVSEGHPDKIADQISDAILDEIFLKDL-----KARVAAETLCNNNLIVLAGEITTDA-NI--------DYVNIVRKTIKDIGYNN--ID-YGIDYKSCAVLLIYNKQSLDIAQGV-NE-GEGLN-F----------------DQ-GAGDQGLMFGYACDETPELMPSAIYYSHLIVKRQSELRKNG--N-LPWLRPDAKSQVTLRY--D-N--G--K-PVAIDTI-VLSTQH-DPEIP---------YR----SIKE-AAIEEIIRPI-----I-P-K-------N-L-LK---NT--RYLINPTGRFVIGGPKGDCGLTGRKIIVDTYGGAAPHGGGAFSGKDPSKIDRSAAYVGRYIAKNIVAANLAKRCQIQISYAIGVAKPTSIMVTSFGSGK--ISDE----KLTMLVKKHFDLRPKNIIKMLNLLR-P-------IYKKTSVYGHFGR-K----------------------EPE---F--T-WELT-DK-A-----EVLK----N----SAGL----------------

>Deltaproteobacteria_Desulfurivibrio_alkaliphilus

-----------MA------------N--Y-LFTSESVSEGHPDKVADQVSDAVLDAILAQDK-----MSRVACETLVTTGMALIAGEITTNA-VV--------DYPSIVRETIKEIGYNS--SD-MGFDWQSCAILTSIDKQSPDIAQGV-DE-GKGVD-L----------------DQ-GAGDQGLMFGYACDETKVLMPMPITYSHRLVKRQAEMRKSG--R-LPWLRPDAKSQVTIEY--E-N--D--K-PKRVEAV-VLSTQH-SPEVD---------YE----DLKE-AVMEEIIKPI-----L-P-E-------N-M-VD--KNT--KYFINPTGRFVIGGPVGDCGVTGRKIIVDTYGGVGSHGGGAFSGKDPSKVDRSSSYMGRYVAKNVVAAGLASQCEVQVAYAIGISQPVSINVVTFGTGK--ISDD----EIRKLITEHFDLRPKAIVQHLDLLR-P-------IYRKTAAYGHFGR-E----------------------REE---F--T-WERT-DK-A-----EALR----A----AAGIK---------------

>Deltaproteobacteria_Desulfofustis_glycolicus

-----------MA------------Q--Y-LFTSESVAEGHPDKVADQISDAILDAIIAKDK-----NARVACETMVTTGMILIAGEITTIS-WV--------DMPDVARQTVREIGYNS--SE-MGFDWQSCAVLTSIDKQSPDIAQGV-NE-GSGLD-L----------------DQ-GAGDQGLMFGYACDDTDVFMPMPIHFAHRLTRRQAEVRKSG--L-LPWLRPDAKSQVTIEY--V-D--G--S-PSRIDAV-VLSTQH-APSID---------YE----DLKE-GVMEEIIKPV-----L-P-S-------N-M-LD--RNT--KYFINPTGRFVIGGPVGDCGVTGRKIIVDTYGGKGSHGGGAFSGKDPSKVDRSSSYMGRYIAKNLVAAGIAKEIEVQVAYAIGISQPVSVNINSFHTGR--ISDD----QIRQLVLDHFDLRPKAIIQHLDLLR-P-------IYKATAAYGHFGR-R----------------------RDD---F--T-WERT-DK-A-----ADLK----A----AAGL----------------

>Deltaproteobacteria_Desulfopila_aestuarii

-----------MS------------N--Y-LFTSESVSEGHPDKVADQISDAILDSILEQDT-----YARVACETLVTTGMALIAGEITTSA-WV--------DMPEVVRQTIKEIGYNS--ST-MGFDWQSCAVLTSIDKQSPDIAQGV-DE-GTGLD-L----------------NQ-GAGDQGLMFGYACNETSVLMPMPIYFAHKLTKQQSLVRKSG--T-LPWLRPDAKSQVTIEY--E-N--R--V-PKRIEAI-VLSTQH-DESIS---------YE----KLKE-AVMQEIISPV-----L-P-A-------E-M-ID--SNT--KFFINPTGRFVIGGPVGDCGVTGRKIIVDTYGGKGSHGGGAFSGKDPSKVDRSSSYMGRYVAKNIVAAGIADEVEVQVAYAIGISQPVSINVNSFGTGR--LPDS----KIKELIVEHFDLRPKAIIQHLNLLR-P-------IYKKTAAYGHFGR-E----------------------DAD---F--T-WEHT-DK-A-----DALR----K----SANI----------------

>Deltaproteobacteria_Desulfobulbus_oralis

-----------MS------------D--Y-IFTSESVSEGHPDKMADQISDAVLDAILTQDK-----YGRVACETLVTTGMVLVAGEITTTA-RI--------DIPQIVRETIEDIGYTS--SD-MGFDSRSCAVLTSLDRQSPDIAQGV-NE-GSGLD-L----------------DQ-GAGDQGLMFGYACDETAGLMPLPITLSQDLVRQQAGVRKSG--R-LSWLRPDAKSQVTVEY--M-D--G--R-PKRIDAV-VLSTQH-AAEVD---------YD----TIRA-GVMEEIIKPA-----L-P-A-------A-M-LD--KDT--KYFINPTGRFVIGGPVGDCGLTGRKIIVDTYGGRGHHGGGAFSGKDPSKVDRSSAYMGRYVAKNIVAAGLAREVEVQVAYAIGIARPVSINVETFGTGR--IPEA----RLVQIVDEIFDLRPKAIIQQLDLLR-P-------IYRKTAAYGHFGR-E----------------------LPE---F--T-WERT-DK-A-----EALK----S----AAGL----------------

>Deltaproteobacteria_Desulfobacter_postgatei

----MS---A-NK------------S--F-LFTSESVTEGHPDKVADAISDSILDAIMAEDK-----KCRVACETLVTTGLVMVAGEITTEC-YV--------DIPEVVRATIKDIGYNS--SN-MGFDWQTCSVITSIDHQSPDIAQGV-NE-GDGLY-K----------------EQ-GAGDQGLMFGFATNETEELMPMPIIYAHKLTRRLAQVRKNG--A-LDFLRPDGKSQVTIEY--V-D--G--V-PKRVDTV-VVSTQH-SPDVS---------YD----DLKA-AVIKEVIKKV-----I-P-G-------E-M-MD--GDT--RFFINPTGRFVVGGPMGDCGVTGRKIIVDTYGGQGSHGGGCFSGKDPSKVDRSASYMGRYVAKNLVAAGLADKCEVQIAYAIGVAEPVSMMVDFMGTGK--ISEA----KAREIVKEVFDLRPAGIIKELDLLR-P-------IYRKTSAYGHFGR-K----------------------DPD---F--Y-WERT-VK-A-----DQIR----S----LAGL----------------

>Deltaproteobacteria_Desulfonema_ishimotonii

---------M-PE------------R--F-YFTSESVTEGHPDKVADAISDSILDAIMTEDK-----NCRVACETLVTTGMAMIAGEITTDC-YV--------DMPQVVRETIREIGYHS--SQ-MGFDWQTCAVLTSIDHQSPDIAQGV-NT-GEGLF-K----------------DQ-GAGDQGLMFGFATDETPELMPMPISYAHKLTHRLATVRKNG--A-LDFLRPDGKAQVTVEY--D-N--G--T-PKRVDTI-VIAAQH-KPDVT---------YE----ELKE-AVIEEVIKKV-----I-P-K-------K-M-ID--GDT--RYFINATGKFVIGGPMGDCGLTGRKIIVDTYGGQGSHGGGCFSGKDPSKVDRSASYMGRHIAKNVVAAGLAKKCEIQVAYAIGVPEPVSLMVDLMGTGV--IPKE----QVKQIVRESFDLRPAAIIEYLDLLR-P-------IYRKTSAYGHFGR-E----------------------EPE---F--T-WEKT-NM-A-----KVLR----E----KAGI----------------

>Deltaproteobacteria_Desulfoluna_spongiiphila

-----M---T-NA------------N--Y-FFTSESVTEGHPDKVADAISDSILDAIMAQDT-----RCRVACETLVTTGMAFIAGEITTSC-YV--------DMTQIVRDTIREIGYDS--SD-MGFDWQTCAVISSIDKQSEDIAQGV-NE-GEGLF-K----------------DQ-GAGDQGLMFGYAIDETPELMPMTVVFAHKLTQRLAEVRKTG--V-LPYLRPDGKAQVTIEY--E-D--G--K-ATRVDTV-VVSSQH-TPEVS---------HE----DLQE-GIIKEVVLKV-----I-P-L-------E-M-ID--DKT--RFFINPTGRFVVGGPMGDCGLTGRKIIVDTYGGRGSHGGGCFSGKDPSKVDRSASYMGRYIAKNIVAAGLAKECEVQVAYAIGVAEPVSVLVDTMGTGV--IPQE----RIAEIVREVFDLRPAAIIEQLDLLK-P-------IYKMTSAYGHFGR-T----------------------DSE--GF--T-WERT-DK-V-----DVLR----E----KAGI----------------

>Deltaproteobacteria_Desulforegula_conservatrix

-----M---S-KE------------K--F-LFTSESVTEGHPDKVADAISDSILDAIMAQDT-----KCRVACETLVTTGLAFIAGEITTSC-YV--------DMPSIVRQTIKDIGYNS--SE-MGFDWQTCSVITSIDQQSPDIAQGV-DE-GEN---K----------------EQ-GAGDQGLMFGYAADETPELMPMPVMMAHKLTKGLADARKSG--R-ASIFRPDGKAQVTIEY--E-D--D--I-PKRIDTI-VVSTQH-TPEAT---------HA----MVKE-AVMEEVIRKV-----L-P-L-------H-M-LD--DKT--KYFINPTGKFVVGGPMGDCGLTGRKIIVDTYGGQGSHGGGCFSGKDPSKVDRSASYMGRYVAKNIVASGLAKRCEVQIAYAIGVAEPVSVLVNTMGTGI--VPND----VIAQAVRKVFELKPAGTIKQLDLLR-P-------IYRQTSAYGHFGR-T----------------------EPE---F--S-WEKT-DL-A-----KKLR----D----VVGV----------------

>Deltaproteobacteria_Desulfuromusa_kysingii

-----M---A-MT------------D--F-LFTSESVGEGHPDKVADQISDAVLDAILEQDP-----KARVACETLVTTGMAVIAGEITTTA-YA--------DLPAIVRQTIKDIGYND--SA-IGFDYKTCAVLTSIDQQSPDISQGV-SE-GEGLF-V----------------EQ-GAGDQGLMFGFACDETPELMPMPITLAHKLTKKLADVRKNN--T-LDFLRPDSKSQVSIEY--V-D--D--K-PTRIDTV-VISTQH-SPEVA---------YE----DLRD-AIIEEVIKPL-----L-P-A-------D-L-LD--SQT--RYLINPTGRFVVGGPMGDCGLTGRKIIVDTYGGQGSHGGGAFSGKDPSKVDRSASYMARYVAKNIVAAGLASKCEVQLAYAIGVAEPVSVMINAFGTGK--VPSD----KIAKIVREEFDMRPAAIIQQLDLLR-P-------IYRQTAAYGHFGR-E----------------------LPG---F--T-WEVT-DR-V-----DSLR----S----RAGL----------------

>Deltaproteobacteria_Pelobacter_carbinolicus

-----M---P-MT------------D--F-LFTSESVSEGHPDKVADQVSDAILDAILDQDR-----QSRVACETMVTTGMAVIAGEITTNA-RV--------EYPKVVREVIRDIGYND--SA-MGFDWETCAVLTSIDRQSPDISQGV-TE-GAGLF-K----------------EQ-GAGDQGLMFGYACDETSVLMPMPITYAHQLTQRMSEVRKSG--L-LTFLRPDSKSQVSVQY--I-N--D--K-PVRVDTV-VVSSQH-APDVA---------YE----TLRE-ALIEEVIKKV-----V-P-A-------D-M-LD--ENT--KFYVNPTGRFVVGGPQGDCGLTGRKIIVDTYGGQGSHGGGAFSGKDPSKVDRSASYMARYVAKNVVAAKLARKCEVQIAYAIGVAEPVSIMVNTFGTGV--LPSN----EIARIVREEFDMRPAAIIETLDLLR-P-------IYRKTAAYGHFGR-E----------------------LPE---F--T-WERT-DR-V-----DSLR----G----RAGL----------------

>Deltaproteobacteria_Geobacter_metallireducens

-----M---E-MK------------D--Y-IFTSESVSEGHPDKVADQVSDSILDAILAQDP-----RARVACETLVTTGMAVIAGEITTSA-VV--------DYPKVVRETIREIGYND--SA-MGFDWETCAVLVSIDKQSPDISQGV-TE-GEGMF-K----------------EQ-GAGDQGLMFGYACTETPELMPMSITYAHKLTQKLAEVRKNG--V-LDFLRPDSKSQVSVEY--V-D--D--K-PVRVDTV-VISSQH-TPEVS---------YE----AIKE-GIIEEVVKKI-----I-P-A-------H-L-MD--ERT--RMLINPTGRFVVGGPMGDCGLTGRKIIVDSYGGHGAHGGGAFSGKDPSKVDRSAAYMGRYVAKNLVAAGLCEKCEVQVAYAIGVAEPVSVMVDTAGTGK--ISSK----RIAEIVREVFDLRPRAIIEQLDLLR-P-------IYRKTAAYGHFGR-E----------------------LPE---F--T-WERT-DK-V-----YMIR----Q----KAGI----------------

>Deltaproteobacteria_Geobacter_lovleyi

-----M---A-EK---------------F-IFTSESVSEGHPDKMADQISDSILDAILAQDP-----KARVACETMVTTGMAVIAGEITTTA-VV--------NYAEIVRNTIKEIGYCG--SE-TGFDYETCSVLVSLDRQSPDISQGV-TE-GEGMF-K----------------EQ-GAGDQGLMFGYACNETPELMPMPIQLSHQLVKRLADVRKSG--L-LKFLRPDAKSQVSVEY--D-N--G--K-PVRVNTV-VISTQH-TPDVT---------HE----TIVE-GVMDEVIKKV-----I-P-A-------H-L-MD--EQT--RFFINPTGRFVVGGPMGDCGLTGRKIIVDTYGGMGRHGGGAFSGKDPSKVDRSAAYMGRYVAKNLVAAGLCERCEVQVAYAIGVAEPVSIMVNAFGTGV--VSEH----RLSELVREVFDMRPRAITEQLDLLR-P-------IYQKTAAYGHFGR-E----------------------LPE---F--T-WEKT-DK-A-----EILK----Q----KAGL----------------

>Deltaproteobacteria_Desulfuromonas_thiophila

-----M---P-MT------------D--F-MFTSESVSEGHPDKMADQISDAILDAILAQDK-----TARVACETLITTGMVMLAGEITTHA-RI--------DHADVVRQTIREIGYVS--SQ-MGFDADTCAVMTSLDRQSPDISQGV-TE-GQGLH-L----------------EQ-GAGDQGLMFGYACIDTPQLMPMPIVFAHELTKRLAQVRKEG--I-LDFLRPDSKSQVSIQY--I-D--D--K-PTRIDAV-VLSTQH-TPEVS---------QR----QIEE-AVMEEVIKKV-----L-P-A-------Q-L-LD--GGT--KYFVNPTGRFVIGGPMGDCGLTGRKIIVDTYGGQGSHGGGAFSGKDPSKVDRSASYMGRYVAKNVVAAGLADKCEVQVAYAIGVAEPVSVMVNAFGTGR--IPSN----EIARIVLEEFDLRPAGIIRTLDLLR-P-------IYRQTAAYGHFGR-E----------------------LPD---F--T-WERT-DR-V-----ESLR----S----RAGL----------------

>Deltaproteobacteria_Desulfococcus_oleovorans

---------M-GR------------T--H-LFTSESVTEGHPDKVADCISDAVLDALISQDK-----NCRVACETLVTTGVAFIAGEITANA-TV--------NFPDIVRDTIRRIGYTS--SD-MGFDWQTCSVVTSIDKQSPDIAQGV-NE-GDGLF-K----------------EQ-GAGDQGLMFGFACDETPELMPAPISYAHKLTKQLADVRKNG--V-LDFLRPDGKSQVTVQY--E-D--G--Q-PKRIDTI-VISSQH-SPDVT---------YD----ELKD-RIIAEVILPV-----I-P-G-------E-L-LD--EQT--KYFINPTGRFVVGGPMGDCGLTGRKIIVDTYGGMGRHGGGCFSGKDPSKVDRSASYMGRHVAKNIVAAGIAKRCEVQVAYAIGVAQPVSVMVDLMGTGR--IPEA----DAERIVREVFDLRPAAIIDYLDLKR-P-------IYGPTAAYGHFGR-T----------------------GDS---F--P-WEST-RR-A-----DEIR----G----KAGI----------------

>Deltaproteobacteria_Desulfomonile_tiedjei

-----------MG------------S--Y-LFSSESVTEGHPDKVADRISDTVLDAMLARDP-----KSRVACETLVTTGLALVAGEITTTA-IV--------DIPQLVRQTVRDIGYTD--AA-MGFDADTCAVMVTLDKQSPDISMGV-TA-GEGLF-S----------------EQ-GAGDQGLMFGFACTETPEYMPMAISYAHQLTERLSLVRKDR--T-LSFLRPDGKSQVTIEY--K-N--G--K-PTRVDTI-VVAAQH-TPDVT---------YD----QIKE-GIIEEVIKKV-----I-P-A-------D-L-LD---NT--RYLINATGRFVVGGPLGDCGLTGRKIIVDTYGGYGAHGGGAFSGKDPSKVDRSASYMARYVAKNVVAAGLAEKCLLQVAYAIGYPEPVSLMVNSYGTGK--IGDQ----KIAQAVSKVFSFKPAAIIEYLDLLR-P-------IYFKTSAHGHFGR-E----------------------DPD---F--T-WERT-DK-V-----QELK----E----AAGV----------------

>Deltaproteobacteria_Syntrophobacter_fumaroxidans

-----M---S-MS------------N--F-LFTSESVTEGHPDKVADQISDSILDAIITEDK-----TARVACETLVTTGLAFVAGEITTSS-WV--------DIPDIIRSTIKGIGYND--SS-MGFDWSTCAVITSIDKQSPDIAQGV-NP-GEGLF-E----------------EQ-GAGDQGLMFGFACNETPVFMPMPIYYAHRITRKLAEVRKNG--V-LEFLRPDGKSQVTVEY--D-D--H--R-PKRIDTI-VVAAQH-APNVS---------YS----MIRE-SIIEEVIKKV-----F-P-P-------E-L-ID--DKT--KYFINSTGRFVIGGPMGDCGLTGRKIIADTYGGQGSHGGGCFSGKDPSKVDRTASYMARYVAKNIVAAGVADKVEVQVAYSIGVAEPVSLMIDTFGTGK--IPSD----RIAEIVRKLFSFKPANMIKQLRLLR-P-------IFKKTACYGHFGR-N----------------------DPD---F--T-WEKT-DM-V-----EPIR----E----LAGI----------------

>Deltaproteobacteria_Syntrophorhabdus_aromaticivorans

---------M-GM------------TK-F-LFTSESVAEGHPDKVADQISDAVLDAIIEQDK-----KARVACETYVTTGLALVGGEITTTC-YA--------NVPDIVRQTVKEIGYND--SS-MGFDWETCAVLTAIDEQSPDIALGV-LE-RED---H----------------EQ-GAGDQGLMIGYASNETAEFMPMPIIYAHKLVKRLAEARKNN--I-LTFLRPDGKSQVTIEYI---D--R--I-PVRVDTV-VIAAQH-NPDVT---------SD----TIRE-GIIEEVIKKI-----I-P-P-------E-M-MD--DKT--RFFVNSTGRFVVGGPKGDCGMTGRKIIVDTYGGMASHGGGAFSGKDPSKVDRSSSYMARYIAKNLVAAGLADRLELQVAYTIGVAHPVSTMINTFGTSK--ISPD----RISEIVGELFDLRPGKVIERLDLLR-P-------IYKKTAVGGHFGR-S----------------------EPE---F--T-WERL-DM-V-----EEIR----K----KAGI----------------

>Deltaproteobacteria_Desulfobacca_acetoxidans

-----M---A-IT------------D--F-LFTSESVTEGHPDKVSDQISDAILDSIIAQDK-----YARVACETLVTTGLAFIAGEITTTA-IV--------DIPSIVRQTIKEIGYND--SS-MGFDWETCAVLTSLDKQSPDIAMGI-D--GRGIF-P----------------EQ-GAGDQGLMFGFACDETEELMPMPIWYAHRLAERLAKVRKEG--I-VPFLRPDGKTQVTVRY--E-N--G--A-PQFIHTV-VIAAQH-NPEVS---------YN----QVKE-AVIEEVIKKT-----V-P-A-------E-L-LH--SDT--RFLVNTTGRFVVGGPLGDCGMTGRKIIVDTYGGAGYHGGGAFSGKDPSKVDRTSSYMLRHVAKNIVAAGLARRCEVQVAYSIGVADPLSIVTNTYGTGV--IPEL----QLLEIIRNTFSFKPAAMISYLDVLR-P-------IFKKTAAYGHFGR-P----------------------DPD---F--T-WEQT-NR-V-----DLLR----E----KAGL----------------

>Deltaproteobacteria_Desulfarculus_baarsii

---------M-GS------------N--Y-MFTSESVTEGHPDKVADQISDAILDAMLTDDP-----ESRVACETLVTTGMAIVAGEVTTKT-YV--------DIPQIVRGTIREIGYSN--SS-MGFDWETCAVMTSLDKQSPDIAMGV-DA-DTAMF-G----------------EQ-GAGDQGLMFGYACDETPELMPMPIYYAHKLARRLAAVRKNG--S-LGFLRPDGKTQVTIEY--E-D--D--R-PKRVEAV-VVAAQH-SPDVK---------QE----RLRE-AIIEEVVRKI-----L-P-A-------E-M-ID--GDT--KMFINTTGRFVVGGPHGDCGLTGRKIIVDTYGGQGSHGGGCFSGKDPSKVDRSGSYFSRYVAKNIVAAGLARKCEVQIAYAIGVPQPVSILVHTYGTET--VDND----RIVAAVRKVFDFRPAMMIQKLQLKQ-P-------MYRRTAAYGHFGR-E----------------------EDG--GF--T-WERR-DM-V-----EALK----A----AV------------------

>Deltaproteobacteria_Hippea_alviniae

-----M---N-AN------------ET-F-LFTSESVTEGHPDKVADQISDAILDAIIAEDK-----NCRVACETMLTTGIVFVAGEISTTT-YA--------EIPEIVRQTIKEIGYTN--AD-YGLDYKSCAVVTSIDRQSPDIAMGV-DK-GEK---------------------L-GAGDQGMMFGYATDETEEYLPLTIVLAHKLSKRLADVRKQG--L-LPYLRPDGKTQVTVRY--K-G--F--N-PDKVTAV-VVSAQH-DPNIE---------LE----QLRK-DIVQYVIRYV-----I-P-Q-------E-L-WDP--DI--KLYINPTGRFVLGGPHADTGLTGRKIIVDTYGGVAAHGGGAFSGKDPTKVDRSGAYMARYIAKNIVAAGIAKRCEVQLAYAIGVEEPVSIMLHTYGTGK--LPKE----DIKKLIVDTFDMTPKGMIETLDLLR-P-------IYKKTAAYGHFGR-N----------------------DKD---F--T-WERT-DK-A-----EELR----K----KAGL----------------

>Deltaproteobacteria_Desulfurella_acetivorans

-----------MK------------NS-F-YFTSESATEGHPDKLADQISDAILDAIIEKDK-----NCRVACETLLTTGLVLCAGEITTES-YV--------EIPDVVRETIKEIGYVN--AD-YGLDYKSCAVITSIHRQSPDIAIGVTKK-GGR---------------------V-GAGDQGMMFGYACNETADFMPLTLSLAHKLSKRLADVRKQG--I-LPYLRPDGKTQVTVRY--D-D--G--V-PTKVTAV-VVSAQH-EPYVE---------LE----RFRK-DIVESVIEHV-----I-P-K-------D-L-WDK--DI--NFYINPTGRFVIGGPQGDTGLTGRKIIVDTYGGAAPHGGGAFSGKDPTKVDRSASYMARYIAKNIVASNIAQKCLVQLAYAIGVEEPVSVMVQTYGTSK--LPES----LIQKAILENLDLTPKGIIDTLDLLR-P-------IYKKTASYGHFGR-K----------------------EKE---F--S-WEKT-DL-V-----EKFK----K----YL------------------

>Deltaproteobacteria_Mailhella_massiliensis

-----M---I-PSK-----------GR-Y-LFTSESVTEGHPDKVADQISDAVLDALLAQDP-----DSHVACETLVTTGMAIVAGEITTKG-YA--------DLPSIVRRTIREIGYIG--SD-MGFDAQTCAVLSSIDKQSPDIAQGV-IR-ENP---E----------------DQ-GAGDQGMMFGFASNETDTLMPAPIYWAHQLSLQLSRVRKNG--I-VDFFRPDGKTQVSFEY--I-D--G--K-PVRINNV-VVSTQH-SPKVS---------HD----DIVE-AVKKEVIRPI-----LEP-T-------G-Y-FD--EKN-CEIFINTTGRFVIGGPMGDCGLTGRKIIQDTYGGMGNHGGGAFSGKDPSKVDRSGAYMARYVAKNIVAAGLADRCEVQIAYCIGVAEPVSVLVHTFDTGK--LPDE----RLTQIVREVFDLRPYFICKRLDLKR-P-------IYSKTSCYGHFGR-E----------------------LPE---F--T-WEKR-DA-V-----ADLL----T----AAKI----------------

>Deltaproteobacteria_Desulfonatronovibrio_hydrogenovorans

-----M---I-ASN-----------SR-Y-LFTSESVTEGHPDKVADQISDSVLDTIISQDP-----NARVACETLVTTGLAFIAGEISTEA-YA--------DFPSIVRETVREIGYNS--SD-MGFDYETCAVISSVDKQSPDIAVGV-DR-KSP---E----------------EQ-GAGDQGMMFGFAVNETKTLMPAPIYYAHKLSRRLAYVRKDN--I-LDFLRPDGKTEVSVEY--I-N--G--R-PNRIHDV-VVACQH-NENIS---------YE----DLVE-AVKEEVIFKV-----L-P-Q-------D-M-VD--KAM--RIFINTTGRFVEGGPKADCGLTGRKIIQDTYGGMGNHGGGAFSGKDPSKVDRSGAYMARYIAKNIVAAELAERCEVQVAYAIGVAEPVSVLVTSWGTGK--VPDE----TLTQAVKEVFDLRPYHIIKKLNLLR-P-------IYKKSSCYGHFGR-E----------------------DVD---F--S-WEKT-DA-V-----DDLL----T----ACKV----------------

>Deltaproteobacteria_Desulfonatronum_lacustre

-----M---H-GNT-----------SS-Y-LFTSESVTEGHPDKVADQISDAVLDCIIAQDP-----TARVACETLVTTGLAFIAGEISTNA-YA--------DLPKIVRETVREIGYTS--SE-MGFDADTCAVISSIDKQSVDIAMGV-DR-TKP---E----------------EQ-GAGDQGMMFGFAVNETATLMPAPIFYSHKLSRRLAYVRKNN--I-LDFLRPDGKTEVCIQY--E-Q--G--K-PKRVDNV-VIACQH-NENIA---------YA----DLVE-AIKKEVIFKS-----L-P-E-------E-M-LD--EKT--RIFINTTGRFVLGGPLADCGLTGRKIIQDTYGGMGNHGGGAFSGKDPSKVDRSGAYMARYIAKNVVAAGLADRCEVQVAYAIGVAEPISTLVTSWGTGV--VSDE----VLTKAVREVFDLRPFYISKRLDLQR-P-------VYKKASIYGHFGR-E----------------------DVD---F--T-WETT-DA-V-----ADLR----T----AAKI----------------

>Deltaproteobacteria_Desulfoplanes_formicivorans

-----M---I-MTT-----------DR-Y-LFTSESVTEGHPDKIADQISDAILDSLLDQDP-----NSRVACETLVTTGMAFIAGEISTSA-YA--------DFPSIVRDTIREIGYTG--SD-MGFDADTCAVVSSVDKQSADIAQGV-DR-KAP---E----------------EQ-GAGDQGMMFGFATNETKTLMPAPIYYAHKLSRRLAYVRKNN--I-LDFLRPDGKTQVSVEY--A-G--G--K-PVHIDNV-VVACQH-NENIA---------YA----DLAD-AVREEVILKS-----L-P-E-------D-L-LD--DAT--RIFINTTGRFVTGGPVADCGLTGRKIIQDTYGGMGNHGGGAFSGKDPSKVDRSGAYMARYIAKNVVAAGLAPTCEVQIAYAIGVADPVSVLVTTGGKGE--VSDE----ALSKAVREVFDLRPYYIVKQLDLLR-P-------IYKNTACYGHFGR-E----------------------RVE---F--S-WEKT-DR-I-----EDLK----T----AVKL----------------

>Deltaproteobacteria_Desulfohalobium_retbaense

-----M---I-ASM-----------EK-Y-LFTSESVTEGHPDKVADQISDAVLDSILAQDK-----NARVACETLVTTGLAFIAGEISTTA-YA--------DFPSIVRDTVREIGYTS--SE-MGFDADTCGVISSVDKQSPDIAQGV-NR-EKP---E----------------EQ-GAGDQGMMFGFAVNETRTLMPAPIYYAHKLSRRLSYVRKKS--I-LDFLRPDGKTEVSVEY--Q-G--G--V-PQRIDTI-IVAAQH-DDQIA---------QD----DLVD-AIKSEVIGKA-----L-P-E-------E-M-ID--KNT--RIYINTTGRFVTGGPLADCGLTGRKIIQDTYGGMGNHGGGAFSGKDPSKVDRSAAYMGRYIAKNIVAAELAQQCEVQLAYVIGVAEPVSVLVNTGGTGE--VPDE----RLTKAVRDVFDLRPYHIVQRLDLLR-P-------IYKKSACYGHFGR-D----------------------NTD---F--T-WERT-DA-T-----EDLR----T----ACKI----------------

>Deltaproteobacteria_Desulfomicrobium_apsheronum

-----M---I-LNA-----------DH-Y-LFTSESVTEGHPDKIADQISDAVLDCLLAQDP-----RSRVACETLVTTGMAVIAGEITTEA-YA--------DLPEIVRSTVREIGYVS--SD-MGFDANTCAVLSSIDKQSPDIAMGV-DR-AKP---E----------------DQ-GAGDQGMMFGYATTETSALMPAPIYYAHGLSRRLAEVRKSG--I-LNFLRPDGKTQVSVEY--R-K--G--K-PVRIDNV-VVSSQH-TPEVT---------YE----EIVE-GIKREVIAKV-----M-P-A-------D-M-MD--ANT--RIYINTTGRFVAGGPLADCGLTGRKIINDTYGGMGNHGGGAFSGKDPSKVDRSGAYMARYVAKNIVAAGLAGTCEVQIAYAIGVAEPVSVLVTTGGTGV--VPDE----VLTKAVRTVFDLRPYYIIKRLNLIQ-P-------IYRKSACYGHFGR-E----------------------DFI---F--P-WEVT-DA-V-----ADLK----T----AAKV----------------

>Deltaproteobacteria_Desulfovermiculus_halophilus

-----M---F-GTP-----------GK-Y-MFTSESVTEGHPDKVADKISDSILDNLIVQDP-----QSRVACETLVTTGLVFVAGEITSNG-FA--------DFPQIARNTVKEIGYDS--SD-MGFDWETCAVISSIDKQSADIAQGV-DR-KSP---E----------------EQ-GAGDQGMMFGYACKETEPLMPAPIHYAHKLSRRLTYTRKKG--I-LDFLRPDGKTQVAMEY--E-N--G--R-PVRIDNV-VVSSQH-DDKVS---------YE----DLKE-GIKREVILKT-----L-P-E-------D-L-ID--DKL--RIFINPTGRFVVGGPLGDCGVTGRKIIQDTYGGMGGHGGGAFSGKDPSKVDRSAAYMARYVAKNVVAADLAEICEVQLAYAIGMADPVSVLVTARGTGE--VDDT----TLTNAVREVFDLRPYYILERLNLKR-P-------IYKDTSCYGHFGR-E----------------------NPN---F--S-WEQT-DA-T-----KDLR----T----ACKV----------------

>Deltaproteobacteria_Halodesulfovibrio_spirochaetisodalis

-----M---I-NNK-----------GK-Y-HFTSESVTEGHPDKVADAISDAILDTIMAQDP-----ECRVACETLVTTGMAVIAGEISTTG-YA--------DFPTVVRDTIREIGYTH--SD-MGFDADTCAVISSIDKQSPDIAQGV-DQ-TSP---E----------------EQ-GAGDQGMMFGYACNETPTLMPAPIYWSHKLSERLTAARKNN--D-LDFLRPDGKTEVSFEY--I-D--G--K-PTRIDTV-VISSQH-KDGVS---------QD----DIVE-GIRGEVINKT-----L-P-A-------E-L-ID--SAT--RIFINTTGRFVIGGPMGDCGLTGRKIIQDTYGGMGHHGGGAFSGKDPSKVDRSAAYMGRYIAKNVVAAGLAPTCEVQIAYVIGVAEPVSVLATSFGTSE--VPDE----ILTKAVKEVFDLRPYGITKTLDLKR-P-------IYKKSACYGHFGR-E----------------------LPE---F--R-WEAT-DA-V-----DALR----T----AAKV----------------

>Deltaproteobacteria_Bradymonas_sediminis

-----------MS------------KN-Y-VFTSESVSEGHPDKVADQISDGILDAILEQDK-----VARVACETIVNTGLALVFGEITTSA-YV--------DVPEIVRETIAKIGYND--PK-LGFDHRSCSVMSAIDEQSMDIAQGV-VT-DIGVV-E----------------EQ-GAGDQGLMFGYACDEMPELMPMPIMYAHKLVQRLADVRKNE--M-PNFFGPDSKSQVTVRY--E-D--N--E-PVAIDAV-VISTQH-LEEIS---------TE----QVRE-AVMETTIKKV-----I-P-A-------E-L-LH--AGT--KYHINPTGRFVAGGPLADSGLTGRKIIVDTYGGMGRHGGGAFSGKDPSKVDRSAAYMARYIAKNIVAAGLARRCEVQLAYAIGVAAPVSIMVNTFGTAT--VDET----KIEQTIPEFFGLKPADIVSTLDLLR-P-------IYRQTAAYGHFGR-D--------------L-------------F--T-WEKT-DK-A-----QALK----D----ALL-----------------

>Deltaproteobacteria_Pajaroellobacter_abortibovis

-------------M-----------NH-Y-RFTSESVTEGHPDKICDAISDAILDAILEQDR-----YAKVACESLAKTGMVVVAGEITTSA-WV--------DIPTIVRNTVREIGYVH--SD-MGFDGNTCAVLTAIEQQSQDIAQGV-LE-GQGLY-H----------------EQ-GAGDQGLVFGYATDETPHLMPAPIYYAHQLAKRLAEVRKEK--E-VLFLRPDGKTQVTLEY--Q-Q--G--V-PVRVDTV-VVSAQH-DESVS---------FE----ALRD-AIVSLVIEHV-----I-P-S-------T-L-VD--KNT--KFYINPTGRFVIGGPMGDCGLTGRKVIVDTYGGIGRHGGGAFSGKDPSKVDRSACYYARYIAKNVVASGLASRCEIQIAYAIGVAKPVGVYINTFGTGV--VEDE----ILEKYITEHFDMRPKSIIHELNLLS-S-------IYKPTATYGHFGR------------------------DI----F--R-WEQT-DR-V-----AKIRE-DFK----SKHYGRAFSTVSTQ------

>Deltaproteobacteria_Myxococcus_fulvus

---------M-P-------------TD-F-LFTSESVTEGHPDKIADQISDGVLDAIIAKDP-----QARVAVETLVKTGLAIVAGEVTTNT-YV--------DIPRIVRSTICKIGYTD--SS-MGYDGNTCGVMVAIEGQSQDIARGV-DN-KK---------------------DQ-GAGDQGMMFGFACDETPELMPAPLHYAHALTRKLADVRRKQ----HDWLRPDGKSQVTVEY--R-E--G--R-PVRIDAV-VVSTQH-AEDIS---------NK----RIQE-AIREDVIAKA-----L-P-K-------K-L-ID--NKT--KFFINPTGRFVVGGPMGDSGLTGRKIIVDTYGGMGRHGGGAFSGKDPSKVDRSAAYMGRYIAKNVVAAGLARRCEVQVSYAIGVAEPVSVMVETFGTAT--VPEE----RIAKAIRQTFGLRPREITEGLDLLR-P-------IYQKTAAYGHFGR-S----------------------EKE---F--T-WERT-DR-K-----DALR----D----AANSTSSS--RRLKAV----

>Deltaproteobacteria_Nannocystis_exedens

------------M------------AS-R-LFTSESVTEGHPDKMCDKISDSILDAILAQDP-----RARVACESLVKTGYVNVAGEITTST-YV--------DIPRIVRATVRTIGYTS--SD-MGFDADSCGVLVAIEGQSPDIAMGV-DE-GAEKE-Q-------------------GAGDQGMMFGYACTETDELMPLPISLAHKLTRQLTEARRAD--K-NSFLRPDGKSQVTVEYD---N--G--R-PVRVDTV-VISTQH-SAEVS---------QA----NLRE-YVMESVIKKV-----V-P-A-------N-L-LD--ART--KFHINPTGRFVVGGPMGDSGLTGRKIIVDTYGGMGRHGGGAFSGKDPSKVDRSAAYYARYIAKNLVASRLCSRAEVQLAYAIGVARPVSIAVDTFGTGK--LADE----KLAQIVEAHFDARPAKLIKELDLLR-P-------IYAQTAAYGHFGR-T----------------------EPD---F--T-WERT-DR-A-----EALA----A----AAAH----------------

>Deltaproteobacteria_Anaeromyxobacter_dehalogenans

---------M-PL------------QD-F-LFTSESVTEGHPDKMADQISDAVLDAVLRQDP-----KGRVACETLLKTGYVMIAGEITTKA-RI--------DYPKLARETVRRIGYTS--GD-MGFDANTCAVLVAVDQQSPDIGQGV-DT-G-------------------------GAGDQGMMFGYACDETPELMPAPIQYAHAVTKQLAKARRAG----LDLLRPDGKSQVSVEY--R-D--G--R-PVRIDTV-VVSTQH-AESVS---------NK----RLHE-AVREQVIAKA-----L-P-K-------R-L-LD--RKT--RILINPTGRFVIGGPMGDTGVTGRKIIVDTYGGMGRHGGGAFSGKDPSKVDRSAAYMGRYIAKNVVAAGLASRCEVQVAYAIGVAEPVSVMVDTFGTAK--VPEG----KIARAVREVFGLTPRAIIEGLDLLR-P-------VYEKTAAYGHFGR-T----------------------EKT---F--T-WERT-DK-K-----DALA----D----AAGL------SKIRAVASV-

>Deltaproteobacteria_Myxococcus_xanthus

---------M-P-------------TD-F-LFTSESVTEGHPDKIADQISDGVLDAIIAKDP-----QARVAVETLVKTGLAIVAGEVTTNC-YV--------DIPKLVRSTICRIGYTD--SS-MGYDGNTCGVMVAIEGQSQDIARGV-DN-KK---------------------DQ-GAGDQGMMFGFACDETPELMPAPIHYAHAITRRLADVRRKQ----HPWIRPDGKSQVTVEY--R-D--G--R-PARIDAV-VVSTQH-SDEVS---------NK----KIQE-AIREDVIANA-----L-P-K-------K-L-ID--NKT--KFFINPTGRLVAGGPMGDSGLTGRKIIVDTYGGMGRHGGGAFSGKDPSKVDRSAAYMGRHIAKTVVAAGLARRCEVQVSYAIGVAEPVSVMVETFGTAT--VPEE----RIALAVRKTFGLRPREITEYLNLLR-P-------IYQKTAAYGHFGR-T----------------------EKE---F--T-WERVEEK-K-----DALR----D----AAKSATPSGGRRLKAV----

>Deltaproteobacteria_Bdellovibrio_bacteriovorus

------------M------------KN-Y-LFTSESVSEGHPDKMADQISDGILDAILAQDP-----KGRVACETLLTTGLVVVAGEITTSA-KV--------NFSEVARDVVKRIGYDH--SD-KGFDYKTCGVMIAVGQQSPDIAVGV-KE-TLS---D----------------NQ-GAGDQGLMFGYAVNETPELMPLSIAMSHKLVKDLAALRKAN--K-VDWLRPDAKSQVTVQY--E-N--G--A-IKRIDAV-VISTQH-ADSVS---------NS----TIQE-FITEELIKKS-----I-P-G-------N-W-ID--SKT--KFFINPTGRFVTGGPMGDAGLTGRKIIVDTYGGHGAHGGGAFSGKDPSKVDRSAAYASRHIAKNIVGAGLAERCLVQVAYAIGVAEPVSITVNDYGTSK--VGPE----VLEKAVRQVFDLRPARITKDLDLLR-P-------IYSPTAAYGHFGR-N----------------------EES---F--T-WERL-NK-V-----DQLK----D----AVKTLA--------------

>Epsilonproteobacteria_Helicobacter_hepaticus

-----------MK------------KS-F-LFTSESVTEGHPDKMADQISDAVLDYIIERDK-----KARVACETLVSNGFCVIAGELKTSV-YA--------PMQEIARKVVQEIGYTD--AL-YGFDYRSAAVLNGIGEQSPDINQGV-DR-E-----D--G-------------EI-GAGDQGLMFGYACKETPSLMPLPIWLSHRLTEGLAKKRKDG--T-LPFLRPDGKSQVTVRY--E-D--G--K-PVSIDTI-VISTQH-SPETQ---------QS----HLKD-AVIEEIVQKV-----L-P-Q-------E-Y-LN--DNI--RYFVNPTGKFVIGGPQGDAGLTGRKIIVDTYGGSCPHGGGAFSGKDPSKVDRSAAYAARYVAKNLVASGVCDKAIVQVAYAIGVVEPVSILVDTQGTGK--VEDS----KLTECVKAVFRLTPKGIIESLDLLR-P-------IYRKTAAYGHFGR-E----------------------LNE---F--S-WEKT-DK-V-----EAIK----D----FCGIK---------------

>Epsilonproteobacteria_Sulfuricurvum_kujiense

-----------MS------------KE-Y-IFTSESVTEGHPDKMADQISDAILDYIIEHDP-----KARVACETLVSNGFCVIAGELKTTT-YA--------PMQEIARQVVREIGYTD--AT-YGFDYRSCAVLNGIGEQSPDINQGV-DQ-K-----S--G-------------EI-GAGDQGLMFGYACRETDVLMPLPIYLSHRLAERLAKVRKEG--I-IPYLRPDGKTQVSVRY--V-D--D--K-PVSVETV-VVSTQH-APEIS---------QE----KLHA-DVIEEVIKAV-----I-P-A-------E-L-MS--PNI--VYHINPTGKFVIGGPQGDAGLTGRKIIVDTYGGACPHGGGAFSGKDPTKVDRSAAYAARYVAKNLVASGACERATIQVSYAIGVVHPISIMVNAHGTAV--VPEE----KLEACVKELFNLTPKGIIESLDLLR-P-------IYRKTATYGHFGR-E----------------------LSE---F--T-WEKT-DK-V-----DAIR----N----YLGL----------------

>Epsilonproteobacteria_Nitratifractor_salsuginis

-----------MA------------KE-Y-IFTSESVTEGHPDKMADQISDAILDYIIERDK-----GARVACETLLSNGYCVIAGELKTHA-YA--------PMQEIAREVVREIGYTD--AS-FGFDYRSAGVLNGIGEQSPDINQGV-DQ-A-----S--G-------------EI-GAGDQGLMFGYACKETEELMPLPISLAHKITARLAQVRKDG--T-LPYLRPDGKAQVSVRY--R-D--G--K-PVEVTTV-VVSTQH-APEIE---------QK----QLHR-DVLQEVIHAV-----I-P-E-------E-L-RA--EDI--VYHINPTGRFVIGGPQGDAGLTGRKIIVDTYGGSCPHGGGAFSGKDPTKVDRSAAYAARWVAKNLVAAGVAERVTIQIAYAIGVVEPVSIMVDTHGTAN--VEES----RLEACVRDVFDLRPAGIIKSLDLLR-P-------IYRKTAAYGHFGR-E----------------------LPE---F--T-WERT-DR-V-----EEIR----S----YLGL----------------

>Epsilonproteobacteria_Sulfurimonas_hongkongensis

-----------MT------------KE-Y-IFTSESVTEGHPDKMADQISDAILDYIIEHDT-----SARVACETMVSNGFCVIAGELKTTA-YA--------PMQEIARRVVQEIGYTD--AT-YGFDYRSAAVLNTIGEQSPDINQGV-DQ-A-----D--G-------------EI-GAGDQGLMFGYACSETPVLMPLPIYLAHRITRRLAEVRKEC--I-VPYLRPDGKAQISVKY--I-G--D--K-PVFVDTI-VISTQH-APDVS---------QE----QIRK-DMVSEVIEHV-----I-P-K-------E-L-MG--EAT--KIHINPTGKFVIGGPQGDAGLTGRKIIVDTYGGSCPHGGGAFSGKDPTKVDRSAAYAARWVAKNLVASGACTKATIQISYAIGIVQPTSIYVDTHSTGV--VDEQ----VIESCVKELFDLSPKGIIESLDLLR-P-------IYRKTASYGHFGR-E----------------------EEG---F--T-WELT-NR-V-----DEIK----R----YLGI----------------

>Epsilonproteobacteria_Nitratiruptor_tergarcus

-----------MA------------RD-Y-LFTSESVTEGHPDKMADQISDAILDYIIQRDP-----HARVACETLLSNGFAIIAGELKTHT-YA--------PMQDIVREVIREIGYTD--AL-YGFDYRSAGVLNAVGEQSPDINQGV-DK-A-----S--G-------------EI-GAGDQGLMFGYACTETDVLMPLPITMAHRLTYELARARKDG--V-LPFLRPDGKAQVTVRY--E-D--G--K-PKEIKTI-VISTQH-DPDVS---------YN----RLKD-AVIEEIVYKV-----I-P-K-------E-L-IA--DDI--VYHINPTGRFVIGGPQGDAGLTGRKIIVDTYGGSCPHGGGAFSGKDPTKVDRSGAYAARYVAKNLVASGVCERVTIQIAYAIGVVEPVSIMVDTHGTGK--VADE----KIEECVKELFDLTPKGIIETLDLLR-P-------IYRKTAAYGHFGR-E----------------------LPE---F--T-WEMT-NK-A-----EEIA----E----FLRISKV-------------

>Epsilonproteobacteria_Sulfurovum_riftiae

-----------MA------------NE-Y-IFTSESVTEGHPDKMADQISDAILDYIIEKDP-----KARVACETLLSNGFCVIAGELKTTA-YA--------PMQEIAREVVREIGYTD--AA-YGFDYRSAGVLNGIGEQSPDINAGV-DQ-E-----G--G-------------EI-GAGDQGLMFGYACRETKELMPLPISLAHHITSKLAEVRKNG--T-VPFLRPDGKAQVSVKY--M-D--G--I-PVAIDTI-VVSTQH-HETVS---------LE----QVQK-AVREEVIDPV-----L-A-T------YD-I-DI--SDI--TYHINPTGRFVIGGPQGDAGLTGRKIIVDTYGGSCPHGGGAFSGKDPTKVDRSAAYAARYVAKNLVAAGACDKATLQVAYAIGVAKPVSIYVDTHGTAH--VDEE----KIVACVESLFDLTPKGIIDSLDLLK-P-------IYKKTAAYGHFGR-E----------------------DMG---F--T-WERT-DK-A-----EEIK----A----FLGL----------------

>Epsilonproteobacteria_Cetia_pacifica

------------M------------RE-Y-LFSSESVTEGHPDKMADQISDAILDYIIERDP-----NAKVACETLLSNGYCIIAGELKTTT-YA--------PMQEIAREVIREIGYTD--AR-YGFDYRTAGVLNGVGEQSPDIRQGV-ER-G-----E----------------EI-GAGDQGMMFGYACTETPELMPLPIMLAHKLTKRLAVARKEA--I-IPWLRPDGKAQVSVKY--V-D--G--K-PVSVEKV-VVSTQH-EPDIN---------YS----EIKE-AVIEEVIKKV-----I-P-A-------N-M-LS--KNV--EYFINPTGKFVIGGPQGDAGLTGRKIIVDTYGGAAPHGGGAFSGKDPTKVDRSGAYAARYVAKNLVAAGVAEKLTVQIAYAIGVVEPVSIYIDTHGTAK--VDET----KIEEAVRKIFNLTPKGIIETLDLLK-P-------IYRKTAAYGHFGR-E------------------------E---F--S-WEKL-DK-V-----EEIK----D----YLNLK---------------

>Epsilonproteobacteria_Arcobacter_halophilus

-----------MT------------NN-Y-LFTSESVTQGHPDKIADQISDAILDYILKEDK-----NARVACETLLTNGLCLIAGELKTLA-YA--------PIQEIARDVIREIGYTD--SA-FGLDYRSAGVLNAIAEQSIDISVGV-DK-K-----S--G-------------EL-GAGDQGMMFGYACNETKELMPLPIQLAHKLTKRLTEVRKKG--I-LPYLRPDGKAQVTVEY--I-N--N--K-PTKVTTI-VISAQH-SDTID---------LK----LLKE-DIIEEVIKEV-----I-P-E-------Q-L-LH--KDV--VYHINPTGRFVIGGPQADAGLTGRKIIVDSYGGSCPHGGGAFSGKDPTKVDRSAAYMARYIAKNLVASEVCDKIMVQLSYAIGVSQPVSIMINTYNSNK--IEEN----KILEIINSCFDLSVEGIIKQLDLLR-P-------IYKQTACYGHFGR-D----------------------DLD---L--P-WEKT-NK-I-----EEIK----K----LIK-----------------

>Gammaproteobacteria_Azotobacter_chroococcum

---------M-SE------------Y--S-LFTSESVSEGHPDKIADQISDAVLDAIIAEDK-----HARVACETLVKTGVAIVAGEITTSA-WV--------DLEQLVRDVIVDIGYDS--SD-VGFDGATCGIVNIIGKQSVDINQGV-DR-ARP---E----------------DQ-GAGDQGLMFGYASNETDVLMPAPICFSHRLVERQAEARKSG--L-LPWLRPDAKSQVTCRY--E-N--G--K-VVGIDAV-VLSTQH-NPEIS---------QA----DLQE-AVMELIIKHT-----L-P-A-------E-L-LH--KDT--QFHINPTGKFVIGGPVGDCGLTGRKIIVDSYGGMARHGGGAFSGKDPSKVDRSAAYAGRYVAKNIVAAGLAERCEIQVSYAIGVALPTSISVNTFGTGK--ISDE----RIVQLVREHFDLRPYAITKMLDLLH-P-------MYRPTAAYGHFGR-TPVEMTVG------------DDTFTA---F--T-WERT-DK-A-----EALR----A----AAGL----------------

>Gammaproteobacteria_Escherichia_coli

---------M-AK------------H----LFTSESVSEGHPDKIADQISDAVLDAILEQDP-----KARVACETYVKTGMVLVGGEITTSA-WV--------DIEEITRNTVREIGYVH--SD-MGFDANSCAVLSAIGKQSPDINQGV-DR-ADP---L----------------EQ-GAGDQGLMFGYATNETDVLMPAPITYAHRLVQRQAEVRKNG--T-LPWLRPDAKSQVTFQY--D-D--G--K-IVGIDAV-VLSTQH-SEEID---------QK----SLQE-AVMEEIIKPI-----L-P-A-------E-W-LT--SAT--KFFINPTGRFVIGGPMGDCGLTGRKIIVDTYGGMARHGGGAFSGKDPSKVDRSAAYAARYVAKNIVAAGLADRCEIQVSYAIGVAEPTSIMVETFGTEK--VPSE----QLTLLVREFFDLRPYGLIQMLDLLH-P-------IYKETAAYGHFGR-E------------------------H---F--P-WEKT-DK-A-----QLLR----D----AAGLK---------------

>Gammaproteobacteria_Marinospirillum_celere

---------M-SE------------Y--A-LFTSESVSEGHPDKLADQISDAVLDALLARDK-----QARVACETLVKTGVAVVGGEITTNA-WV--------DLEDLVRGVIKEIGYTS--SD-VGYDGDTCGVINIIGKQSVDIAQGV-DR-QKP---E----------------DQ-GAGDQGLMFGYASNETDVLMPAPITFAHRLVERQAEARKSG--L-LNWLRPDAKSQVTCRY--E-N--G--K-VVGIDAV-VLSTQH-NPEVS---------QK----DLKE-AVMELIVKQT-----L-P-A-------E-F-LH--KDT--QFHINPTGKFVIGGPVGDCGLTGRKIIVDTYGGMARHGGGAFSGKDPSKVDRSAAYAGRYVAKNLVAAGIADRCEIQVSYAIGVAEPTSVSVNTFGTGK--VSDE----VITKLIRQHFDLRPYAITRMLDLLH-P-------MYQLTAAYGHLGR-EPFESSYSWTDTEGKA---FTETFTA---F--P-WEKT-DK-A-----ELLR----T----EAGL----------------

>Gammaproteobacteria_Colwellia_beringensis

---------M-ST------------H----LFTSESVSEGHPDKIADQISDAVLDAIIAKDK-----HARVACETMVKTGVAIISGEVSTTA-WV--------DLERITRDVISDIGYTS--SD-VGFDGETCGIMNLIGQQSPEIAQGV-DR-VKP---E----------------DQ-GAGDQGLMFGYATNETETLMPAPLYYSHRLVERQAEARKSG--I-LPWLRPDAKSQVTFIY--E-D--N--K-PVAIDTV-VLSTQH-NPDIK---------QE----DLHD-AVMENIIKHV-----L-P-A-------E-L-LT--KDT--KYHINPTGRFVIGGPVGDCGLTGRKIIVDTYGGMARHGGGAFSGKDPSKVDRSAAYAGRYVAKNIVAAGLADRCEIQISYAIGVAEPTSISIDSFGTGK--VSEE----RLVEIVREHFDLRPYGITKMLDLLH-P-------MYKQTAAYGHFGR-EPFEMTVG------------DDTFTA---F--S-WEKT-DK-A-----EALR----L----SAGI----------------

>Gammaproteobacteria_Salinicola_peritrichatus

---------M-SE------------Y--S-LFTSESVSEGHPDKIADQISDAVLDAIIARDK-----QARVACETLVKTGVAIVAGEISTTA-WV--------DLEELVRKVILEIGYTS--SD-VGFDGETCGVINLIGKQSVDIAQGV-DR-SKP---E----------------DQ-GAGDQGLMFGYATNETPSYMPAPIHYAHRLVERQSELRKNG--T-LSWLRPDAKSQVTFRYG-D-D--G--K-PVAVDAV-VLSTQH-DESIS---------QE----ELRH-AVEELVIRDV-----L-P-A-------E-W-IT--EST--RFHINPTGKFLIGGPVGDCGLTGRKIIVDTYGGMARHGGGAFSGKDPSKVDRSAAYAGRYVAKNIVAAGLADMCEIQVSYAIGVAEPTSVSINTFGTGK--VSDD----KIIELVREHFDLRPYAITRMLDLLH-P-------MYQLTAAYGHFGR-EPFEHSYAWTDVSGEQ---QTETFTA---F--P-WEKT-DK-A-----EALR----S----AAGQ----------------

>Gammaproteobacteria_Kushneria_indalinina

---------M-SE------------Y--S-LFTSESVSEGHPDKIADQISDAVLDALIARDK-----QARVACETLVKTGVAIVAGEITTSA-WV--------DLEDLVRRVISDIGYTS--SE-VGFDGATCGVLNLIGKQSIDIAQGV-DR-SKP---E----------------DQ-GAGDQGLMFGYATNETPSYMPAPIHYSHRLVERQSQLRRNG--T-LSWLRPDAKSQVTFRYD-E-N--G--K-PVAVDAV-VLSTQH-DDSIS---------QE----ELRH-AVEELIIRDV-----L-P-A-------E-W-IT--EST--RFHINPTGKFVIGGPVGDCGLTGRKIIVDTYGGMARHGGGAFSGKDPSKVDRSAAYAGRYVAKNVVASGIADKCEIQISYAIGVAQPTSVSINTFGTGK--ISDE----QIIELVREHFDLRPYAITRMLDLLH-P-------MYQLTASYGHFGR-EPFEHTYTWRDVNGEE---QTETFTA---F--P-WEKI-DR-A-----EALR----D----AAGL----------------

>Gammaproteobacteria_Spongiibacter_marinus

---------M-SE------------Y--S-LFTSESVSEGHPDKLADQISDAVLDAIIARDK-----HARVAVETLVKTGMAVVAGEVTTSC-YV--------DLEDIIRDVITGIGYNS--SD-VGFDGASCAVLNAIGKQSVDINQGV-DR-AKP---E----------------DQ-GAGDQGLMFGYATNETEHLMPAPLFYSHRLVERQAYLRKNG--V-LPWLRPDAKSQVTLRY--E-N--G--V-PVAVDAV-VLSTQH-NPDIS---------QA----DLQE-AVREEVIKHV-----L-P-A-------N-L-LH--AGT--QFHINPTGNFVIGGPVGDCGLTGRKIIVDTYGGMARHGGGAFSGKDPSKVDRSAAYAGRYVAKNIVAAGLADRCEIQVSYAIGVAEPTSISVNTFGTGK--VSDE----KLVAAVRQVFDLRPYGITNMLDLAH-P-------MYQPTAAYGHFGR-EPYEHTYQWTE-NGEA---RSETSTA---F--S-WEKT-DR-A-----EALK----A----AV------------------

>Gammaproteobacteria_Thalassolituus_oleivorans

---------M-SE------------Y--S-IFTSESVSEGHPDKVADQISDAVLDAIIARDP-----YARVAVETLVKTGMAVVAGEVTTSC-YV--------DLEEIVRDVITGIGYNS--SE-VGFDGATCAVLNGIGKQSVDINQGV-DR-AKP---E----------------DQ-GAGDQGLMFGYATNETPSLMPAPVYYAHLLVQRQSELRRNG--T-LPWLRPDAKSQVTINW--E-S--G--S-P-KVDAV-VLSTQH-SPSIS---------LE----ELRA-EVLEHIIKPV-----I-P-A-------E-W-LH--EGT--LYHINPTGNFVIGGPVGDAGLTGRKIIVDTYGGMARHGGGAFSGKDPSKVDRSAAYMGRYVAKNIVAAGLADRCEIQVSYAIGVAQPTSISVNTFGTGK--ITDI----QLAKVIREVFDLRPYAIQNQLELLN-P-------MYQITAAYGHFGR-EPFETSYTYVD-QGVS---KTKTFTA---F--T-WERT-DK-V-----DALK----A----AAGL----------------

>Gammaproteobacteria_Gynuella_sunshinyii

---------M-SE------------Y--S-LFTSESVSEGHPDKMADQISDAILDAIIKDDP-----NSRVAVETLVKTGMAVIAGEVRTNT-YV--------DLEDIVRQVILDIGYDS--SD-VGFDGASCAVLNAIGKQSSDIAMGV-DE-AES---K----------------DL-GAGDQGLMFGYASNETDVLMPAPIYFSHRLVERQAQLRKSK--V-LPWLRPDAKSQVTLRY--E-G--N--K-PVAVDAV-VLSTQH-SPEVS---------QA----DIQE-AIMEEVIKHV-----L-P-A-------E-W-LH--KDT--RYHINPTGQFIIGGPVGDCGLTGRKIIVDTYGGMARHGGGAFSGKDPSKVDRSAAYAGRYVAKNIVAAGLADRCEIQVSYAIGVAEPTSISINTFGTNT--IDEA----RIAELVREHFDLRPQGIIDMLNLRR-P-------IYRPTASYGHFGR-T----------------------GED---F--T-WEQT-DK-A-----EALK----A----AATL----------------

>Gammaproteobacteria_Pseudohongiella_nitratireducens

---------M-S-------------S--H-LFTSESVSEGHPDKIADQISDAILDALLRQDP-----AARVACETLVKTGMVVVAGEVTTEA-YV--------DLEDIARKVVIDIGYDH--SD-KGFDGHSCAVLNGIGKQSPEIAMGV-DE-TED---H----------------EQ-GAGDQGLMFGYASNETDVLMPAPITYSHRLVKRQAEIRKNG--V-LPWLRPDAKSQITFRY--E-D--G--K-PAGIDAV-VLSTQH-DPEIA---------QK----DLQE-AVMEEIIKPV-----L-P-A-------E-W-LS--ADT--KYFINPTGRFVIGGPYGDCGLTGRKIIVDTYGGMARHGGGAFSGKDPSKVDRSAAYAARYVAKNIVAAGIADRCEVQVSYAIGVAEPTSISVDTFGTGK--ISDA----RIVELVREHFELRPKGLIKMLDLIR-P-------IYLPTAAYGHFGR-E----------------------EPE---F--T-WERT-DK-A-----DALR----D----AAGL----------------

>Gammaproteobacteria_Beggiatoa_alba

---------M-SE------------A--V-LFTSESVSEGHPDKIADQISDAVLDALLAQDK-----KARVACETLVKTGMVVVAGEITTNT-WV--------DVEALVRNTIKEIGYNS--SE-MGFDWESCAVLSAIGKQSVDIAVGV-DE-TEN---H----------------EQ-GAGDQGLMFGYATNETDVLMPAPITYAHRLVKRQAELRKNG--T-LPWLRPDAKSQVTFRY--I-D--G--K-PVGVDAV-VLSTQH-SPDIS---------NK----SLQE-AIMDDVIKHV-----I-P-S-------E-W-LD--KNT--RYYINPTGRFVIGGPMGDCGLTGRKIIVDTYGGMARHGGGAFSGKDPSKVDRSAAYACRYVAKNIVAAGLAQRCEVQVSYAIGVAEPTSILVETFGTGK--IDDA----KLVRLVREHFDLRPRGLIAMLNLLR-P-------IYGKTASYGHFGR-E----------------------DAD---F--T-WEQT-DK-A-----DALR----E----AAGIK---------------

>Gammaproteobacteria_Spiribacter_curvatus

---------M-GS------------Q--Y-LFTSESVSEGHPDKVADQISDAILDAILKDDP-----AARVACETLVKTGMVIVAGEITTSA-WI--------DLEDLVRRRIVDIGYNS--AE-VGFDGATCAVLNAIGKQSPDINQGV-DR-ELP---E----------------EQ-GAGDQGMMFGYACRETDVLMPAPITYAHRLVKRHSNVRRSG--V-LPWLRPDAKSQVSFVY--E-D--G--R-PVAVDTI-VLSSQH-DDSVS---------QA----DLRE-AIMEEVIRPV-----I-P-A-------E-W-IS--DRT--RFFINPTGQFVIGGPVGDCGLTGRKIIVDTYGGMARHGGGAFSGKDPSKVDRSAAYACRYVAKNIVAAGLADKCEIQVAYAIGVAEPMSISIDTFGTGQ--ISET----RLVELVREHFDLRPYGILRMLDLVR-P-------IYQPTASFGHFGR-E----------------------EET---F--T-WERT-DR-A-----DSLR----D----AAGL----------------

>Gammaproteobacteria_Woeseia_oceani

---------M-TQ------------S--F-LFTSESVSEGHPDKISDQISDAVLDAILEQDK-----SARVACETLVKTGMVMVAGEVTTTA-WV--------DIEELVRKTVIGIGYTD--SS-MGFDGASCAVINALGKQSPDIAQGV-DR-GDP---E----------------SQ-GAGDQGLMFGYATNETDVLMPAPVTFAHRLVRRQAEVRKNG--T-LPWLRPDAKSQVTFRY--E-N--N--K-PVSIDAV-VLSSQH-HADIS---------MK----DLRD-GIMEEIIKPV-----I-P-A-------E-W-LS--NDT--KYHINPTGRFEIGGPMGDCGLTGRKIIVDTYGGSARHGGGAFSGKDPSKVDRSAAYACRYVAKNIVAAGLAERCEIQVSYAIGVAEPTSISVHTFGTGK--VSED----KLTALVREHFDLRPYGILKMLDLIK-P-------IYQPTAAYGHFGR-E----------------------DLD---L--S-WERT-DR-A-----DALR----G----AAAA----------------

>Gammaproteobacteria_Thiomicrospira_microaerophila

---------M-T-------------T--T-VFTSESVSEGHPDKIADQISDAMLDAILHQDP-----RARVACETFVKTGMVLLGGEITTSA-WV--------DQEELVRNVVKEIGYDS--AD-LGFDGDTCAVLSAIGKQSPEIAMGV-DE-FDD---H----------------EQ-GAGDQGLMFGYASNETDVLMPAPIYYAHRLMERQAAVRKSG--E-LAWLRPDAKSQVTLRY--E-N--G--K-PVAIDAV-VLSTQH-SPDIG---------NA----ELRE-AIMETIIKPT-----L-P-T-------E-W-LH--ANT--QYHINPTGRFVIGGPVGDAGLTGRKIIVDTYGGMARHGGGAFSGKDPSKVDRSAAYAGRYVAKNIVAAGLADKCEIQVSYAIGVAQPTSISIDTFGTEK--VAVA----KIEQLVAQHFDLRPKGLIAMLDLYR-P-------IYQKTAAYGHFGR-E----------------------LPE---F--T-WEKT-DK-A-----EALR----A----DAGL----------------

>Gammaproteobacteria_Shigella_dysenteriae

---------M-AK------------H----LFTSESVSEGHPDKIADQISDAVLDAILEQDP-----KARVACETYVKTGMVLVGGEITTSA-WV--------DIEEITRNTVREIGYVH--SD-MGFDANSCAVLSAIGKQSPDINQGV-DR-ADP---L----------------EQ-GAGDQGLMFGYATNETDVLMPAPITYAHRLVQRQAEVRKNG--T-LPWLRPDAKSQVTFQY--D-D--G--K-IVGIDAV-VLSTQH-SEEID---------QK----SLQE-AVMEEIIKPI-----L-P-A-------E-W-LT--SAT--TFFINPTGRFVIGGPMGDCGLTGRKIIVDTYGGMARHGGGAFSGKDPSKVDRSAAYAARYVAKNIVAAGLADRCEIQVSYAIGVAEPTSIMVETFGTEK--VPSE----QLTLLVREFFDLRPYGLIQMLDLLH-P-------IYKETAAYGHFGR-E------------------------H---F--P-WENT-DK-A-----QLLR----D----AAGLK---------------

>Gammaproteobacteria_Tolumonas_lignilytica

---------M-AR-----------------LFTSESVAEGHPDKIADQISDAVLDAILEQDP-----KARVACETFVKTGMVLVGGEITTSA-WV--------DIEELVRKTVCDIGYTH--SD-MGFDAHSCAVLNAIGKQSPDINQGV-DR-ADP---K----------------EQ-GAGDQGLMFGYASNETDVLMPAPITYAHRLVKRQSEVRKNG--T-LPWLRPDAKSQITFIY--DKQ--G--K-IEGIDAV-VLSTQH-APDIS---------QS----DLIE-AVHEVIIKPV-----L-P-A-------E-W-VS--KNT--KYFINPTGRFVIGGPMGDCGLTGRKIIVDTYGGMARHGGGAFSGKDPSKVDRSAAYAARYVAKNIVAAGLAERCEIQVSYAIGVAEPTSISVETFGTGK--VSEE----LLTQLVRDQFDLRPYGLIEMLDLIQ-P-------IYKATAAYGHFGR-E------------------------E---F--P-WEKT-DK-A-----ALLR----D----LAGLK---------------

>Gammaproteobacteria_Gilliamella_apis

---------M-SE------------F----LFTSESVSEGHPDKIADQISDAVLDAILEQDP-----KARVACETYVKTGMALVGGEITTSA-WV--------DIEELTRKTINDIGYTS--SE-MGFDANSCAVLNAIGKQSPDINQGV-DR-KNP---L----------------EQ-GAGDQGIMFGYATNEMPNLMPAAISYAHDLMRRQAEVRKNG--C-LPWLRPDAKSQVTLIY--Q-D--G--K-IQGVDTI-VLSTQH-SEDIE---------QK----ALHE-AVMEEIIKPT-----L-P-S-------E-W-LT--SRT--KYFINPTGRFVIGGPMGDCGLTGRKIIVDTYGGAARHGGGAFSGKDPSKVDRSAAYAARYVAKNIVAAGLADKCELQISYAIGVAHPTSIYVNTFGTEK--IAQD----KIISLIKEFFDLRPYGLIQMLDLIQ-P-------IYQKTASYGHFGR-D------------------------I---F--P-WEKT-DK-A-----AILR----D----AAGL----------------

>Gammaproteobacteria_Enterovibrio_norvegicus

---------M-AK------------H----LFTSESVSEGHPDKIADQISDAVLDAILEQDP-----KARVACETYVKTGMVMVGGEVTTSA-WV--------DIEEITRETVREIGYVH--SD-MGFDADSCAVLNTIGKQSPDINQGV-DK-ADP---K----------------DQ-GAGDQGIMFGYATNETPILMPAPITYSHLLVKKQAEVRKSG--K-LDFLRPDAKSQVTFQY--D-Q--G--K-IVGIDAV-VLSTQH-CDSVT---------TE----YLRE-AVMEEIIKPV-----L-P-I-------E-W-LN--KDT--KYFINPTGRFVIGGPMGDCGLTGRKIIVDTYGGAARHGGGAFSGKDPSKVDRSAAYAARYVAKNIVAAGMADRCEIQLSYAIGVADPTSIMVETFGTEK--VSQE----IIIEAVRQNFDLRPYGLQEMLDLLQ-P-------IYKKTAAYGHFGR-E------------------------E---F--P-WEKT-DK-V-----AALR----D----FANIK---------------

>Gammaproteobacteria_Aliidiomarina_minuta

---------M-AQ------------H----LFTSESVSEGHPDKIADQISDAVLDAILATDP-----KARVACETYVKTGMVLVGGEINTHA-WV--------DVEDLVRTTVRKIGYTN--SE-MGFDAGSCAVLNAIGKQSADINQGV-DR-GNP---E----------------EQ-GAGDQGLMFGYASDETEALMPAPITYAHKLVQRQAEVRRS---S-LPWLRPDAKSQLSFIY--E-D--G--K-PIGIDAV-VLSTQH-AEEYS---------LK----QVQE-AVMETIIKPV-----L-P-E-------E-W-LR--PDT--KYHINPTGKFVIGGPMGDCGLTGRKIIVDTYGGMARHGGGAFSGKDPSKVDRSAAYAARYVAKNLVAAGLAKRCELQVSYAIGVAQPTSISIETFGTSR--YDEE----TLIKLVRQHFDLRPYGLIKMLDLER-P-------IYLETAAYGHFGR-D------------------------Q---F--P-WEKT-DK-A-----EALR----A----AIK-----------------

>Gammaproteobacteria_Nitrosococcus_wardiae

---------M-KE------------T--R-QFTSESVSEGHPDKIADQISDAILDAILAKDK-----KARVACETLVKTGMVLVAGEITTQA-QV--------DYEQIIRELIIKIGYDS--SE-MGFDGATCAVLNAIGKQSPDIAQGV-DR-ELE---E----------------EQ-GAGDQGMMFGYASDETDVLMPAPITYSHRLVQRQAEVRRSG--E-LPWLRPDAKSQVTLLY--E-D--D--V-PVGIDAV-VLSTQH-SPEIK---------QT----TLRE-AVIETIIKPV-----L-P-A-------E-W-LARCKSE--NIHVNPTGSFAIGGPMGDCGLTGRKIIVDTYGGMARHGGGAFSGKDPSKVDRSAAYAGRYVAKNLVAAGLAERCEVQISYAIGVAEPTSVSVNTFGTGR--VAES----RLVQLIRAHFDLRPAGLLRMLDLLQ-P-------IYRKTAAYGHFGR-E----------------------EPE---F--T-WEQT-DK-A-----EALR----D----AAGLGPITA-EVVNARSESH

>Gammaproteobacteria_Suttonella_ornithocola

---------M-SH------------Y----LFTSESVSEGHPDKIADQISDAVLDAILEQDK-----NARVACETLIKTGMVLVAGEVSTSA-WV--------DLEDIVRQTIVNIGYNS--SD-VGFDGATCAVLNAIGKQSSDIAQGV-DR-EEK---R----------------QQ-GAGDQGLMFGYACDETDTLMPAPITYAHRLVERQTQVRKDK--V-LPWLRPDAKSQITFRY--K-N--G--V-ISGIDAV-VLSTQH-DPDIS---------QK----DLRE-AVRELIIDKV-----L-P-A-------E-W-LD--NQT--KFHINPTGNFVTGGPVGDCGLTGRKIIVDTYGGAAHHGGGAFSGKDPSKVDRSAAYAGRYVAKNIVAAGLAKRCEVQVSYAIGVAEPTSIMVNTFGTGT--IPDE----QIVQRVREVFDLTPYGIIEMLDLVR-P-------IYQQTATYGHFGR-E----------------------LPE---F--T-WEKT-DK-A-----ESLK----L----A-------------------

>Gammaproteobacteria_Rudaea_cellulosilytica

---------M-ST------------T----LFTSESVSEGHPDKVADQISDAVLDAILAQDK-----KARVACETLVKTGVAIVAGEVTTSA-WI--------DLEGITRKVILDIGYNS--SD-VGFDGATCGVLNLIGKQSPDINQGV-DR-KSP---E----------------EQ-GAGDQGLMFGYATNETRDYMPAAIYYSHRLVEQQTKVRKKG--K-LKWLRPDAKSQVTLRY--E-D--G--K-AVAIDAV-VLSTQH-DPSVK---------QK----DLIA-GVREEILNPV-----L-P-A-------K-W-LH--KGT--KFHINPTGKFVIGGPVGDCGLTGRKIIVDTYGGWARHGGGAFSGKDPSKVDRSAAYAARYVAKNIVAAGLADRCEIQVSYAIGVAEPTSISVTTFGTGK--ISDE----KIEKLVRKHFDLRPYGIIKMLDLIH-P-------MYQQTAAYGHFGR-TPQEIKLP----NG-------EKYTT---F--S-WEKT-DK-A-----EQLR----A----DAKLK---------------

>Gammaproteobacteria_Ignatzschineria_indica

---------M-TY-----------------LFTSESVSEGHPDKVADQISDAVLDALIKEDK-----GARVACETLVKTGVAIVAGEVTTNA-WV--------DLEDLVRRVITDIGYDS--SK-VGFDGKTCGVLNIIGKQSSEIAQGV-DR-KLP---E----------------EQ-GAGDQGLMFGYASNETDVLMPAPITFAHRLMECQSEARKSR--Q-LSWLRPDAKSQVTFAYN-N-D--G--S-ISHVDAV-VLSTQH-DEEVK---------AK----DLEE-AVMELIIKKT-----L-P-A-------E-W-LT--KET--KYHINPTGSFTIGGPVGDCGLTGRKIIVDTYGGMARHGGGAFSGKDPSKVDRSAAYAGRYVAKNIVAAGLADKCEIQVSYAIGVAEPTSISITTFGTNK--VPEA----EIETLVRKHFDLRPYGIITMLDLLH-P-------IYLPTASYGHFGR-HPYEIEYEAAD----G---TTTTATA---F--S-WEKT-DK-A-----DILR----E----EAGLK------------ES-

>Gammaproteobacteria_Francisella_hispaniensis

---------M-SK------------N--Y-LFTSESVSEGHPDKLADQISDAILDEILKQDK-----NARVACETLVKTGMALVAGEITTSA-WV--------DIEELVRNVITETGYDN--AN-KGIDGRTCSVINAIGKQSRDIAQGV-DR-GSL---E----------------DL-GAGDQGLMFGFATNETPTLMPSAIYYSHLLMRKQAELRKSG--K-LAWLRPDAKAQVTLAY--E-N--D--K-PKFIDTI-VLSTQH-NESIS---------QK----ELHD-AVIEEIVKKV-----I-P-N-------E-L-IT--KNT--KYHINPTGVFLIGGPQGDCGLTGRKIIVDTYGGAAHHGGGAFSGKDPSKVDRSGAYMGRYIAKNIVAAGLADKCEVQVAYAIGVAKPVSLMVNTFGTGK--ITDN----QIEKLVAEIFDLRVGKIIENLDLLR-P-------IYRKTSNYGHFGR-E----------------------LPE---F--T-WEKI-DK-A-----DILK----S----AARI----------------

>Gammaproteobacteria_Cysteiniphilum_litorale

---------M-AN------------N--F-LFTSESVSEGHPDKIADQISDAVLDAILIQDK-----NARVACETLVKTGMALVAGEITTSA-WV--------DIEEIVRNVVIDIGYDK--DD-LGFDGNCCAVINAIGKQSPEIAQGV-DR-SNP---L----------------DQ-GAGDQGLMFGFATNETPTLMPAAIYYSHKLMQRQAFLRKNK--T-LSWLRPDAKSQVTLRY--V-N--G--K-PVAVDTV-VISTQH-APEIR---------QN----VIKE-AVIEEIILPV-----L-P-K-------E-W-IT--DKT--KYFVNPTGKFVIGGPVGDCGLTGRKIIVDTYGGAAHHGGGAFSGKDPSKVDRSAAYMGRYIAKNIVASGLADKCEIQVSYAIGVANPTSIMVNTFGTGK--LDDH----IIEKLVLEHFDLRPYAITHQLDLLR-P-------IYQKTAAYGHFGR-E----------------------DAD---F--T-WEKT-DK-A-----AILA----Q----ALN-----------------

>Gammaproteobacteria_Legionella_rowbothamii

---------M-NE------------S--F-VFTSESVSEGHPDKIADQISDAILDAILEQDP-----KARVACEVFVKTGMVLVGGEITTKA-WV--------DVEAVTRNVVKDIGYNS--SQ-MGFDWESCAVLSAIGKQSPDIAQGV-DN-TTT---K----------------LQ-GAGDQGIMFGYASRETDVYMPAPIAYAHRLMEKQAQLRKSG--D-LSWLRPDAKSQVTLRY--E-H--G--M-PVEVDTV-VFSTQH-SEEVS---------HQ----DLVE-AVREDIIKTT-----L-P-A-------E-W-LT--DKT--RYFINPTGRFVIGGPLGDCGLTGRKIIVDTYGGTARHGGGCFSGKDPSKVDRSAAYAARYVAKNIVAAGLADKCEIQVSYAIGVAEPTSIFVETFGTGR--LANK----EIIELIHAHFDLTPQGIIEHHDLLR-P-------IYRETATYGHYGR-E-----------Q----------------F--P-WERL-DK-V-----AQLQ----K----AL------------------

>Gammaproteobacteria_Succinivibrio_dextrinosolvens

-------------------------MS-R-LFTSESVSEGHPDKICDQVSDAILDAILEQDK-----TAHVACETLVKTGLILVAGEVTTTA-NV--------DFEAVARKTVCDIGYNN--SE-VGFDGHNCAFLNALGKQSPDINQGV-SR-SLP---E----------------DQ-GAGDQGLMFGYATDETPQFMPAAITYAHELMKLQAKLRKYG--T-ISWLRPDAKSQVTMAYK-E-D--G--S-IDYVDTI-VLSTQH-NEDVS---------QE----IVHE-AVYEEIIKKV-----I-P-A-------E-Y-LR--KET--KIFINPTGRFVIGGPVGDAGVTGRKIIVDTYGGAARHGGGAFSGKDPSKVDRSAAYAARHVAKNIVAAGLAHKAEVQVSYAIGVAQPVSISVNTFGTGK--IDDN----KIAEIVPHVFDLRPYGLIKDLDLLR-P-------IYQKTASYGHFGR-D------------G---------------F--S-WEEL-DK-V-----DALK----Q----AL------------------

>Gammaproteobacteria_Steroidobacter_denitrificans

---------M-SS------------S--Y-LFTSESVSEGHPDKVSDQISDAILDAILAQDK-----HSRVAAETLCNTGLVILAGEITSNA-AV--------DYQSIARETIRRIGYDN--TD-FGIDYKGCAVLVAYDKQSPDIAQGV-DE-GRGLD-L----------------DQ-GAGDQGLMFGYACDETPELMPLPIYLAHRLVERQAQLRHSG--A-LSWLRPDAKSQVTVRY--V-D--G--R-PKEIDTV-VLSTQH-HPDVD---------HA----TLSQ-AVIEEIIKPV-----L-P-Q-------E-M-IG--KKI--NYLVNPTGRFVIGGPQGDCGLTGRKIIVDTYGGAAPHGGGAFSGKDPSKVDRSAAYAARYVAKNIVASGLAAKAQVQVSYAIGVARPTSIMVTTFGTGK--IADE----QLEELVTKHFDLRPKGIVQMLDLLR-P-------IYEKTAAYGHFGR-S----------------------EPG---F--T-WEKT-DK-A-----EALA----L----DAGVKRAVN-G---------

>Gammaproteobacteria_Acinetobacter_baumannii

---------M-RE------------Y--A-VFTSESVSEGHPDKMADQISDAILDAILKEDP-----YARVACETLVKTGAVVLAGEITTTA-NI--------DVEAVVRQTVNGIGYHH--SD-LGFDGSTCAVINMIGKQSPEIAQGV-DR-QKP---E----------------DQ-GAGDQGLMFGYASRETDVLMPAPISYAHRLMERQAELRRSG--A-LPWLRPDAKSQVTFAY--E-N--G--K-PVRLDAV-VLSTQH-DPEIT---------QT----QLKE-AVIEEIIKPI-----I-P-A-------E-M-FH--AAT--KFHINPTGMFVIGGPVGDCGLTGRKIIVDTYGGMARHGGGAFSGKDPSKVDRSAAYAGRYVAKNIVAAGLADKCEIQVSYAIGVAEPTSISINTFGTAK--VSDE----LIIQLVREHFDLRPFGITRMLNLIQ-P-------MYKQTAAYGHFGR-G---------------------SNTA---F--T-WEKT-DK-V-----EALK----D----AAGL----------------

>Gammaproteobacteria_Yersinia_pestis

---------M-AK------------H----LFTSESVSEGHPDKIADQISDAVLDAILEQDP-----KARVACETYVKTGMVLVGGEVTTNA-WV--------DIEEITRRTIREIGYVH--SD-MGFDANSCAVLSAIGKQSPDINQGV-DR-ENP---L----------------EQ-GAGDQGLMFGYATNETSVLMPAPITYAHRLVERQAEVRKNG--A-LPWLRPDAKSQVTFQY--D-D--G--K-IVGIDAV-VLSTQH-SEDIN---------QK----DLHE-AVMEEIIKPV-----L-P-A-------E-W-IT--AHT--KYFINPTGRFVIGGPMGDCGLTGRKIIVDTYGGMARHGGGAFSGKDPSKVDRSAAYAARYVAKNIVAAGLADRCEIQVSYAIGVAEPTSIMVEAFGTEK--IPAD----QLTLLVREFFDLRPYGLIKMLDLLH-P-------IYRETAAYGHFGR-E------------------------H---F--P-WEKT-DK-A-----ALLR----D----AAGLK---------------

>Gammaproteobacteria_Gullanella_endobia

---------M-AR------------H----LFTSESVSEGHSDKIADQISDTVLDAILAQDP-----KARVACETYVKTGMVLVGGEITTSA-WI--------DIEELTRNTIREIGYTH--SD-MGFDANSCAILSAFGKQSPDINQGV-DR-TDP---L----------------EQ-GAGDQGLMFGYATNETDVLMPAPITYAHRLVERQSQVRKNG--I-LPWLRPDAKSQITFVY--E-N--G--K-VIGIDTV-VLSTQH-AEDIA---------LP----QLKK-AVMEEIIKPV-----L-P-S-------E-W-LF--AQT--KYFINPTGQFIIGGPMGDCGLTGRKIIVDTYGGIAHHGGGAFSGKDPSKVDRSAAYAARYVAKNIVAAGLAERCEIQISYAIGVAEPISITIETFGTEK--ISVD----NLSNLVRRFFDLRPYGLISMLNLLQ-P-------IYRKTATYGHFGR-D----------------------------F--P-WEKT-DK-A-----ELLR----D----AAGLK---------------

>Gammaproteobacteria_Arsenophonus_lipoptenae

---------M-TS------------H----LFTSESISEGHPDKIADQISDAVLDAILEQDT-----TAKVACETYVKTGMVIVGGEITTNA-WI--------DIEEIARSTIREIGYTS--SD-MGFDANSCAVICAIGKQSLDIYQGI-NH-KNV---L----------------EQ-GAGDQGIMFGYANNETDVFMPAPIIYAHRLVKRQSEVRKNG--T-LPWLRPDAKSQVTFQY--E-N--N--K-IVGIDTI-VLSTQH-SEEIK---------SK----ILSE-AVMDEIIKPV-----L-P-T-------K-W-LH--KNT--KYFINPTGRFVIGGPMGDCGLTGRKIIADTYGGMARHGGGAFSGKDPSKVDRSAAYAARYVAKNIVAAGLADRCEIQISYVIGIAKPISIMIETFGTEK--ISND----IFIHVVNEIFDLRPYGLIKMLNLLR-P-------IYQPTATYGHFGR-S------------------------Q---F--P-WEKI-NK-V-----ADLR----S----AVGFN---------------

>Gammaproteobacteria_Riesia_pediculicola

---------M-TGS----------------LFTSESVSEGHPDKVADQISDAVLDEILRQDK-----NSKVACEVYVKNGMVMIGGEVKTQS-NI--------DLENTVRKTLSDIGYTD--SK-IGFDARSCAIINMISKQSPDILQGI-NK-KNPIE-Q-------------------GAGDQGIVFGYADIETEDLMPAPITYAHKLMIRQSEVRKSK--I-LPWIRPDAKSQITFRY--E-N--G--K-IIGIHSI-VISTQH-SEEID---------RN----SLKE-GVIEEIILPI-----I-P-K-------K-W-IK--KYT--KYFINPTGRFVIGGPMGDCGLTGRKIVVDTYGGTSRHGGGAFSGKDPSKVDRSAAYAARYIAKNIVASGIAEKCEIQISYIIGIPEPNSLTIETFGTEK--VDIK----YLKNIINEIFDLRPYGLIKMLDLLR-P-------IYLKTASYGHFGR-S--------------I-------------F--P-WEKI-DK-V-----KQLKS-IFR----IAN-----------------

>Gammaproteobacteria_Methylococcus_capsulatus

---------M-SN------------S--F-IFTSESVSEGHPDKIADQVSDAVLDAILAQDP-----KARVAVETLVKTGMVVLAGEVTTSA-WV--------DTEELVRKVVHEIGYDN--PE-IGFDWQSCAVLTAIGKQSPDIAMGV-DE-FDD---H----------------EQ-GAGDQGLMFGYATNETDVLMPAPITYAHRLVQRQAEVRKHK--V-LPWLRPDAKSQVTFRY--E-E--G--K-PVAVDAV-VLSTQH-APDVA---------QK----DIRE-AVMEEIIYHV-----L-P-R-------E-W-LH--KDT--KYFINPTGSFVIGGPVGDCGLTGRKIIVDTYGGMARHGGGAFSGKDPSKVDRSAAYMGRYVAKNIVAAGLAERCEIQVSYAIGVAEPTSISIETFGTGR--IAEE----TLVKLVREHFDLRPKGLITMLDLLR-P-------IYRPTAAYGHFGR-T----------------------EGT---F--T-WERT-DK-A-----DLLR----E----AAGLPIA-------------

>Zetaproteobacteria_Mariprofundus_ferrinatatus

---------M-SR------------N--F-VFTSESVSEGHPDKVADRISDGILDAILEQDR-----YARVACETMVTTGLALIAGEITTSA-VI--------DYQDVVRSAIKEIGYNS--SA-MGFDWESCAVLVSLDKQSPDIAMGV-NE-GEGLD-L----------------DQ-GAGDQGLMFGYASNETEVLMPTPIHLSHLLVAKQAEKRKNG--T-LNFLRPDAKSQVTVRY--E-N--Y--K-PVAIDAV-VLSTQH-NPDIE---------HK----VLCE-AIMDEVINPV-----L-G-S-------TGL-LH--SGT--EYHINPTGRFVIGGPVGDCGVTGRKIIVDTYGGFGHHGGGAFSGKDPTKVDRSACYMMRYVAKNIVAAGLADRCEVQVAYAIGVAHPLSVMVNTFGTGK--IDEG----KLSGIVREVFDLRPKGIVQALDLLR-P-------IYAQTAAYGHFGR-E----------------------LPD---F--T-WEKT-DK-V-----DALK----A----AAGV----------------

>Zetaproteobacteria_Ghiorsea_bivora

---------M-SR------------N--F-VFTSESVGEGHPDKVADQISDSVLDAILKQDP-----TARVACETMVNTGMVILSGEITTSA-VI--------DYQEIARNTIKEIGYNS--SD-MGFDYASCAVLVTMDKQSVDIAAGV-NE-GEGLD-L----------------DQ-GAGDQGLMFGYASNETDVLMPMPVHLSHQLMEKQAEVRKTG--V-LKYLRPDAKSQVTVRY--E-N--S--K-PVAIDAV-VISTQH-TADVS---------HE----DLVA-GVMQEIVHPV-----L-D-K-------TGL-LH--DKT--EYHINPTGRFVIGGPVGDCGVTGRKIIVDTYGGFGHHGGGAFSGKDPTKVDRSACYMMRYVAKNIVAAELADRCEVQVAYAIGVARPLSVMVNTFGTGK--VDEA----KLAEAVREVFDLRPKGIVQELDLLR-P-------IYAKTAAYGHFGR-E----------------------LPE---F--T-WEKT-DK-V-----DALK----A----AVKS----------------
