# Supplementary material for: Implications of divergence of methionine adenosyltransferase in archaea
Source: FEBS Open Bio. 2021 Nov 5;12(1):130–45. doi: 10.1002/2211-5463.13312 (PMC8727953; doi:10.1002/2211-5463.13312)
Supplement: Supplementary file 3 — Appendix S2. Archaea. [file FEB4-12-130-s004.docx]

>MAT2_Human

M------------NGQ--L----N-------GFH--E-AF-IEEGTFLFTSESVGEGHPDKICDQISDAVLDAHLQQDPDAKVACETVAKTGMILLAGEITSRAAVDYQKVVREAVKHIGYDDSSKGFDYKTCNVLVALEQQSPDIAQGVHL---DRNEEDIGAGDQGLMFGYATDETEECMPLTIVLAHKLNAKLAELRRNGTLPWLRPDSKTQVTVQYMQDRGAVLPIRVHTIVISVQHDEEVCLDEMRDALKEKVIKAVVPAKYLDEDTIYHLQPSGRFVIGGPQGDAGLTGRKIIVDTYGGWGAHGGGAFSGKDYTKVDRSAAYAARWVAKSLVKGGLCRRVLVQVSYAIGVSHPLSISIFHYGTSQ----K---SERELLE-IVKKNFDLRPGVIVRDLDLKKP---I---YQRTAAYGHFGRD------SFPWEVPKKLKY---------------------------

>MAT1_Human

M------------NGP--V----D-------GLC--DHSL-S-EGVFMFTSESVGEGHPDKICDQISDAVLDAHLKQDPNAKVACETVCKTGMVLLCGEITSMAMVDYQRVVRDTIKHIGYDDSAKGFDFKTCNVLVALEQQSPDIAQCVHL---DRNEEDVGAGDQGLMFGYATDETEECMPLTIILAHKLNARMADLRRSGLLPWLRPDSKTQVTVQYMQDNGAVIPVRIHTIVISVQHNEDITLEEMRRALKEQVIRAVVPAKYLDEDTVYHLQPSGRFVIGGPQGDAGVTGRKIIVDTYGGWGAHGGGAFSGKDYTKVDRSAAYAARWVAKSLVKAGLCRRVLVQVSYAIGVAEPLSISIFTYGTSQ----K---TERELLD-VVHKNFDLRPGVIVRDLDLKKP---I---YQKTACYGHFGRS------EFPWEVPRKLVF---------------------------

>MAT1_Rat

M------------NGP--V----D-------GLC--DHSL-SEEGAFMFTSESVGEGHPDKICDQISDAVLDAHLKQDPNAKVACETVCKTGMVLLCGEITSMAMIDYQRVVRDTIKHIGYDDSAKGFDFKTCNVLVALEQQSPDIAQCVHL---DRNEEDVGAGDQGLMFGYATDETEECMPLTIVLAHKLNTRMADLRRSGVLPWLRPDSKTQVTVQYVQDNGAVIPVRVHTIVISVQHNEDITLEAMREALKEQVIKAVVPAKYLDEDTIYHLQPSGRFVIGGPQGDAGVTGRKIIVDTYGGWGAHGGGAFSGKDYTKVDRSAAYAARWVAKSLVKAGLCRRVLVQVSYAIGVAEPLSISIFTYGTSK----K---TERELLE-VVNKNFDLRPGVIVRDLDLKKP---I---YQKTACYGHFGRS------EFPWEVPKKLVF---------------------------

>MAT2_Rat

M------------NGQ--L----N-------GFH--E-AF-IEEGTFLFTSESVGEGHPDKICDQINDAVLDAHLQQDPDAKVACETVAKTGMILLAGEITSRAAIDYQKVVREAIKHIGYDDSSKGFDYKTCNVLVALEQQSPDIAQGVHL---DRNEEDIGAGDQGLMFGYATDETEECMPLTIVLAHKLNAKLAELRRNGTLPWLRPDSKTQVTVQYMQDRGAVIPIRVHTIVISVQHDEEVCLDEMRDALKEKLIKAVVPAKYLDEDTIYHLQPSGRFVIGGPQGDAGLTGRKIIVDTYGGWGAHGGGAFSGKDYTKVDRSAAYAARWVAKSLVKGGLCRRVLVQVSYAIGVSHPLSISIFHYGTSQ----K---SERELLE-IVKNNFDLRPGVIVRDLDLKKP---I---YQRTAAYGHFGRD------SFPWEVPKKLKY---------------------------

>MAT1_Bovine

M------------NGP--V----D-------GLC--DHSL-SEEGAFMFTSESVGEGHPDKICDQISDAVLDAHLKQDPNAKVACETVCKTGMVLLCGEITSMAMVDYQRVVRETIQHIGYDDSAKGFDFKTCNVLVALEQQSPDIAQCVHL---DRNEEDVGAGDQGLMFGYATDETEECMPLTIMLAHRLNARMAELRRSGQLPWLRPDSKTQVTVQYTQDNGAVIPMRVHTVVISVQHNEDITLEDMRRALKEQVIRAVVPARYLDEDTIYHLQPSGRFVIGGPQGDAGVTGRKIIVDTYGGWGAHGGGAFSGKDYTKVDRSAAYAARWVAKSLVKAGLCRRVLVQVSYAIGVAEPLSISIFTYGTSQ----K---TERELLD-VVNKNFDLRPGVIVRDLDLKKP---I---YQKTACYGHFGRS------EFPWEVPKKLVF---------------------------

>MAT2_Bovine

M------------NGQ--L----N-------GFH--D-AF-IEEGTFLFTSESVGEGHPDKICDQISDAVLDAHLQQDPDAKVACETVAKTGMILLAGEITSRAAVDYQKVVRETIKHIGYDDSSKGFDYKTCNVLVALEQQSPDIAQGVHL---DRNEEDIGAGDQGLMFGYATDETEECMPLTIVLAHKLNAKLAELRRNGTLPWLRPDSKTQVTVQYMQDRGAVLPIRVHTIVISVQHDEEVCLDEMRDALKEKVIKAVVPAKYLDEDTIYHLQPSGRFVIGGPQGDAGLTGRKIIVDTYGGWGAHGGGAFSGKDYTKVDRSAAYAARWVAKSLVKGGLCRRVLVQVSYAIGVSHPLSISIFHYGTSQ----K---SERELLE-IVKKNFDLRPGVIVRDLDLKKP---I---YQRTAAYGHFGRD------SFPWEVPKKLKY---------------------------

>MAT2_Gorilla

M------------NGQ--L----N-------GFH--E-AF-IEEGTFLFTSESVGEGHPDKICDQISDAVLDAHLQQDPDAKVACETVAKTGMILLAGEITSRAAVDYQKVVREAVKHIGYDDSSKGFDYKTCNVLVALEQQSPDIAQGVHL---DRNEEDIGAGDQGLMFGYATDETEECMPLTIVLAHKLNAKLAELRRNGTLPWLRPDSKTQVTVQYMQDRGAVLPIRVHTIVISVQHDEEVCLDEMRDALKEKVIKAVVPAKYLDEDTIYHLQPSGRFVIGGPQGDAGLTGRKIIVDTYGGWGAHGGGAFSGKDYTKVDRSAAYAARWVAKSLVKGGLCRRVLVQVSYAIGVSHPLSISIFHYGTSQ----K---SERELLE-IVKKNFDLRPGVIVRDLDLKKP---I---YQRTAAYGHFGRD------SFPWEVPKKLKY---------------------------

>MAT1_Gorilla

M------------NGP--V----D-------GLC--DHSL-S-EGVFMFTSESVGEGHPDKICDQISDAVLDAHLKQDPNAKVACETVCKTGMVLLCGEITSMAMVDYQRVVRDTIKHIGYDDSAKGFDFKTCNVLVALEQQSPDIAQCVHL---DRNEEDVGAGDQGLMFGYATDETEECMPLTIILAHKLNARMADLRRSGLLPWLRPDSKTQVTVQYMQDNGAVIPVRIHTIVISVQHNEDITLEEMRRALKEQVIRAVVPAKYLDEDTVYHLQPSGRFVIGGPQGDAGVTGRKIIVDTYGGWGAHGGGAFSGKDYTKVDRSAAYAARWVAKSLVKAGLCRRVLVQVSYAIGVAEPLSISIFTYGTSQ----K---TERELLD-VVHKNFDLRPGVIVRDLDLKKP---I---YQKTACYGHFGRS------EFPWEVPRKLVF---------------------------

>MAT1A_Neomonachus_schauinslandi

M------------NGP--V----D-------GLC--DHSL-SEEGAFMFTSESVGEGHPDKICDQISDAVLDAHLKQDPNAKVACETVCKTGMVLLCGEITSTATVDYQRVVRGAIRHIGYDDSAKGFDFKTCNVLVALEQQSPDIAQCVHL---DRNEQDVGAGDQGLMFGYATDETEECMPLTIILAHKLNARMADLRRSGVLPWLRPDSKTQVTVQYTQDNGAVIPVRIHTIVISVQHNEDITLEDMRKALKEQVIKAVVPAKYLDEDTIYHLQPSGRFVIGGPQGDAGVTGRKIIVDTYGGWGAHGGGAFSGKDYTKVDRSAAYAARWVAKSLVKAGLCRRVLVQVSYAIGVAEPLSISIFTYGTSQ----K---TERELLD-VVSKNFDLRPGVIVRDLDLKKP---I---YQKTACYGHFGRS------EFPWEIPKKLVF---------------------------

>MAT2A_Neomonachus_schauinslandi

M------------NGQ--L----N-------GFH--E-AF-IEEGTFLFTSESVGEGHPDKICDQISDAVLDAHLQQDPDAKVACETVAKTGMILLAGEITSRAAVDYQKVVRETIKHIGYDDSSKGFDYKTCNVLVALEQQSPDIAQGVHL---DRNEEDIGAGDQGLMFGYATDETEECMPLTIVLAHKLNAKLAELRRNGTLPWLRPDSKTQVTVQYMQDRGAVLPIRVHTIVISVQHDEEVCLDEMRDALKEKVIKAVVPAKYLDEDTIYHLQPSGRFVIGGPQGDAGLTGRKIIVDTYGGWGAHGGGAFSGKDYTKVDRSAAYAARWVAKSLVKGGLCRRVLVQVSYAIGVSHPLSISIFHYGTSQ----K---SERELLE-IVKKNFDLRPGVIVRDLDLKKP---I---YQRTAAYGHFGRD------SFPWEVPKKLKY---------------------------

>MAT2A_Orcinus_orca

M------------NGQ--L----N-------GFH--D-AF-IEEGTFLFTSESVGEGHPDKICDQISDAVLDAHLQQDPDAKVACETVAKTGMILLAGEITSRAAVDYQKVVRETIKHIGYDDSSKGFDYKTCNVLVALEQQSPDIAQGVHL---DRNEEDIGAGDQGLMFGYATDETEECMPLTIVLAHKLNAKLAELRRNGTLPWLRPDSKTQVTVQYMQDRGAVLPIRVHTIVISVQHDEEVCLDEMRDALKEKVIKAVVPAKYLDEDTIYHLQPSGRFVIGGPQGDAGLTGRKIIVDTYGGWGAHGGGAFSGKDYTKVDRSAAYAARWVAKSLVKGGLCRRVLVQVSYAIGVSHPLSISIFHYGTSQ----K---SERELLE-IVKKNFDLRPGVIVRDLDLKKP---I---YQRTAAYGHFGRD------SFPWEVPKKLKY---------------------------

>MAT1A_Orcinus_orca

M------------NGP--V----D-------GLC--DHSL-SEEGAFMFTSESVGEGHPDKICDQISDAVLDAHLKQDPSAKVACETVCKTGMVLLCGEITSMAMVDYQRVVRDTIKHIGYDDSAKGFDFKTCNVLVALEQQSPDIAQCVHL---DRNEEDVGAGDQGLMFGYATDETEECMPLTIILAHRLNARMAELRRSGQLPWLRPDSKTQVTVQYTQERGAVIPVRIHTIVISVQHNEDVTLEDMRRALKEQVIRAVVPAQYLDKDTIYHLQPSGRFVIGGPQGDAGVTGRKIIVDTYGGWGAHGGGAFSGKDYTKVDRSAAYAARWVAKSLVKAGLCRRVLVQVSYAIGVAEPLSISIFTYGTSQ----K---TERELLD-VVNKNFDLRPGVIVRDLDLKKP---I---YQKTACYGHFGRS------EFPWEVPKKLVF---------------------------

>MAT1A_Loxodonta_africana

M------------NGP--V----D-------GLC--DHSL-SEEGAFMFTSESVGEGHPDKICDQISDAVLDAHLKQDPNAKVACETVCKTGMVLLCGEITSVAMVDYQRVVRDTIKHIGYDDSAKGFDFKTCNVLVALEQQSPDIAQCVHL---DRNEEDIGAGDQGLMFGYATDETEECMPLTIVLAHKLNAQMADLRRSGVLPWLRPDSKTQVTVQYTQERGAVIPVRVHTIVISVQHNEDITLEDMRTALKEQVIWAVVPAKYLDKNTIYHLQPSGRFVIGGPQGDAGVTGRKIIVDTYGGWGAHGGGAFSGKDCTKVDRSAAYAARWVAKSLVKAGLCRRVLVQVSYAIGVAEPLSISIFTYGTSQ----R---TERELLD-VVNKNFDLRPGVIVRDLDLKKP---I---YQKTACYGHFGRS------EFPWEIPKKLVF---------------------------

>MAT2A_Loxodonta_africana

M------------NGQ--L----N-------GFH--E-AF-IEEGTFLFTSESVGEGHPDKICDQISDAVLDAHLQQDPDAKVACETVAKTGMILLAGEITSRAAVDYQKVVRETIKHIGYDDSSKGFDYKTCNVLVALEQQSPDIAQGVHL---DRNEEDIGAGDQGLMFGYATDETEECMPLTIVLAHKLNAKLAELRRNGTLPWLRPDSKTQVTVQYMQDRGAVLPIRVHTIVISVQHDEEVCLDEMRDALKEKVIKAVVPAKYLDEDTIYHLQPSGRFVIGGPQGDAGLTGRKIIVDTYGGWGAHGGGAFSGKDYTKVDRSAAYAARWVAKSLVKGGLCRRVLVQVSYAIGVSHPLSISIFHYGTSQ----K---SERELLE-IVKKNFDLRPGVIVRDLDLKKP---I---YQRTAAYGHFGRD------SFPWEVPKKLKY---------------------------

>MAT1A_Equus_caballus

M------------NGP--V----D-------GLC--DHSL-SEEGAFMFTSESVGEGHPDKICDQISDAVLDAHLKQDPDAKVACETVCKTGMVLLCGEITSMAAVDYQRVVRDTIKHIGYDDSAKGFDFKTCNVLVALEQQSPDIAQCVHL---DRNEEDVGAGDQGLMFGYATDETEECMPLTIVLAHRLNARMAELRRAGVLPWLRPDSKTQVTVEYVQDKGAVIPVRVHTIVISVQHDEDITLEDMRRALKEQVIRAVVPAKYLDGDTIYHLQPSGRFVIGGPQGDAGVTGRKIIVDTYGGWGAHGGGAFSGKDYTKVDRSAAYAARWVAKSLVKAGLCRRVLVQVSYAISVAEPLSISIFTYGTSE----K---TERELLD-VVNKNFDLRPGVIVRDLDLKRP---I---YQKTACYGHFGRS------EFPWEVPKKLVF---------------------------

>MAT2A_Equus_caballus

M------------NGQ--L----N-------GFH--E-GF-IEEGTFLFTSESVGEGHPDKICDQISDAVLDAHLQQDPDAKVACETVAKTGMILLAGEITSRAAVDYQKVVRETIKHIGYDDSSKGFDYKTCNVLVALEQQSPDIAQGVHL---DRNEEDIGAGDQGLMFGYATDETEECMPLTIVLAHKLNAKLAELRRNGTLPWLCPDSKTQVTVQYMQDRGAVLPIRVHTIVISVQHDEEVCIDEMRDALKEKVIKAVVPAKYLDEDTIYHLQPSGRFVIGGPQGDAGLTGRKIIVDTYGGWGAHGGGAFSGKDYTKVDRSAAYAARWVAKSLVKGGLCRRVLVQVSYAIGVSHPLSISIFHYGTSQ----K---SERELLE-IVKKNFDLRPGVIVRDLDLKKP---I---YQRTAAYGHFGRD------SFPWEVPKKLKY---------------------------

>MAT1A_Eptesicus_fuscus

M------------NGP--V----D-------GLY--DTSL-SEEGAFMFTSESVGEGHPDKICDQISDAVLDAHLRQDPNAKVACETVCKTGMVLLCGEITSMAMVDYQRVVRDTIKHIGYDDSAKGFDFKTCNVLVALEQQSPDIAQCVHL---DRNEEDVGAGDQGLMFGYATDETEECMPLTIILAHKLNARMAELRRSGVLPWLRPDSKTQVTVQYMQNNGAVVPVRVHTIVISVQHNEDITLEDMRKALQEQVIRAVVPAKYLDKDTIYHLQPSGRFVIGGPQGDAGVTGRKIIVDTYGGWGAHGGGAFSGKDYTKVDRSAAYAARWVAKSLVKAGLCRRVLVQVSYAIGVAAPLSISIFTYGTSQ----K---TERELLD-VVNKNFDLRPGVIVRDLDLKKP---I---YQKTACYGHFGRS------EFPWEVPKKLVF---------------------------

>MAT2A_Eptesicus_fuscus

M------------NGQ--L----N-------GFH--D-AF-IEEGTFLFTSESVGEGHSDKICDQISDAVLDAHLLQDPDAKVACETVAKTGMILLAGEITSRAAIDYQKVVRETIKHIGYDDSSKGFDYKTCNVLVALEQQSPDIAQGVHL---DRNEEDIGAGDQGLMFGYATDETEECMPLTIVLAHKLNAKLAELRRNGTLPWLRPDSKTQVTVQYMQDRGAVIPIRVHTIVISVQHDEEVCLDEMRDALKEKVIKAVVPAKYLDEDTIYHLQPSGRFVIGGPQGDAGLTGRKIIVDTYGGWGAHGGGAFSGKDYTKVDRSAAYAARWVAKSLVKEGLCRRVLVQVSYAIGVSHPLSISIFHYGTSQ----K---SERELLE-IVKKNFDLRPGVIVRDLDLKKP---I---YQRTAAYGHFGRD------SFPWEVPRKLKY---------------------------

>MAT1A_Pseudopodoces_humilis

M------------NGP--V----D-------GLC--DYTL-DDEGAFMFTSESVGEGHPDKICDQISDAVLDAHLKQDPNAKVACETVCKTGMVLLCGEITSRAIVDYQRVVRDAIRHIGYDDSAKGFDYKTCNVLVALEQQSPDIAQGVHL---HRNEEDVGAGDQGLMFGYATDETEECMPLTIILAHKLNARLAELRRSGELPWLRPDSKTQVTVQYIQKNGAVIPVRVHTIVISVQHDETISLESMRRTLKERVIQVVVPAKYLDDKTVYHLQPSGRFVIGGPQGDAGVTGRKIIVDTYGGWGAHGGGAFSGKDYTKVDRSAAYAARWVAKSLVKAGLCRRVLVQVSYAIGVAHPLSISLFTYGTSQ----K---TEKELLD-IVHKNFDLRPGVIVRDLDLKKP---I---YQKTACYGHFGRQ------EFSWEVPKKLVF---------------------------

>MAT2A_Pseudopodoces_humilis

M------------NGQ--L----N-------GFH--E-VF-IEEGTFLFTSESVGEGHPDKICDQISDAVLDAHLKQDPDAKVACETVAKTGMILLAGEITSRANVDYQKVVRDTIRHIGYDDSSKGFDYKTCNVLVALEQQSPDIAQGVHL---DRSEEDIGAGDQGLMFGYATDETEECMPLTIVLAHKLNAKLAELRRSGALPWLRPDSKTQVTVQYMQDRGAVIPIRVHTIVISVQHDEDVCLDEMRDALKEKVIKAVVPPKYLDDDTIYHLQPSGRFVIGGPQGDAGLTGRKIIVDTYGGWGAHGGGAFSGKDYTKVDRSAAYAARWVAKSLVKAGLCRRVLVQVSYAIGVSHPLSISIFHYGTSQ----K---SERELLE-IVKKNFDLRPGVIVRDLDLKKP---L---YQRTAAYGHFGRD------SFPWEVPKKLKY---------------------------

>MAT2A_Aquila_chrysaetos

M------------NGQ--L----N-------GFH--E-VF-IEEGTFLFTSESVGEGHPDKICDQISDAVLDAHLKQDPDAKVACETVAKTGMILLAGEITSRAAVDYQKVVRDTIKHIGYDDSSKGFDYKTCNVLVALEQQSPDIAQGVHL---DRSEEDIGAGDQGLMFGYATDETEECMPLTIVLAHKLNAKLAELRRNGTLPWLRPDSKTQVTVQYMQDRGAVIPIRVHTIVISVQHDEEVCLDEMRDALKEKVIKAVVPAKYLDEDTIYHLQPSGRFVIGGPQGDAGLTGRKIIVDTYGGWGAHGGGAFSGKDYTKVDRSAAYAARWVAKSLIKAGLCRRVLVQVSYAIGVSHPLSISIFHYGTSQ----K---SERELLE-IVKKNFDLRPGVIVRDLDLKKP---L---YQRTAAYGHFGRD------SFPWEVPKKLKY---------------------------

>MAT1A_Aquila_chrysaetos

M------------NGP--V----D-------GLC--DYTL-DDEGAFMFTSESVGEGHPDKICDQISDAVLDAHLKQDPNAKVACETVCKTGMVLLCGEITSHAIVDYQRVVRDAIRHIGYDDSAKGFDYKTCNVLVALEQQSPDIAQGVHL---HRDEEDVGAGDQGLMFGYATDETEECMPLTIILAHKLNARLAELRRNGELPWLRPDSKTQVTVQYIQENGAVIPVRVHTIVISVQHDETISLENMRRTLKDRVIQAVVPAKYLDEKTIYHLQPSGRFVIGGPQGDAGVTGRKIIVDTYGGWGAHGGGAFSGKDYTKVDRSAAYAARWVAKSLVKAGLCRRVLVQVSYAIGVAHPLSISLFTYGTSQ----K---TEKELLD-IVHKNFDLRPGVIVRDLDLKKP---I---YQKTACYGHFGRN------EFSWEVPKKLVF---------------------------

>MAT1A_Lonchura_striata

M------------NGP--V----D-------GLC--DYTL-DDEGAFMFTSESVGEGHPDKICDQISDAVLDAHLKQDPNAKVACETVCKTGMVLLCGEITSRAIVDYQRVVRDAIRHIGYDDSAKGFDYKTCNVLVALEQQSPDIAQGVHL---HRNEEDVGAGDQGLMFGYATDETEECMPLTIILAHKLNARLAELRRSGELPWLRPDSKTQVTVQYTQKNGAVVPVRVHTVVISVQHDETISLENMRRTLKERVIQVVVPAKYLDDKTVYHLQPSGRFVIGGPQGDAGVTGRKIIVDTYGGWGAHGGGAFSGKDYTKVDRSAAYAARWVAKSLVKAGLCRRVLVQVSYAIGVAHPLSISLFTYGTSQ----K---TEKELLD-IVHKNFDLRPGVIVRDLDLKKP---I---YQKTACYGHFGRQ------EFSWEVPKKLVF---------------------------

>MAT2A_Lonchura_striata

M------------NGQ--L----N-------GFH--E-VF-IEEGTFLFTSESVGEGHPDKICDQISDAVLDAHLKQDPDAKVACETVAKTGMILLAGEITSRANVDYQKVVRDTIRHIGYDDSSKGFDYKTCNVLVALEQQSPDIAQGVHL---DRSEEDIGAGDQGLMFGYATDETEECMPLTIVLAHKLNAKLAELRRGGALPWLRPDSKTQVTVQYMQDRGAVIPIRVHTIVISVQHDEDVCLDEMRDALKEKVIKAVVPPKYLDDDTIYHLQPSGRFVIGGPQGDAGLTGRKIIVDTYGGWGAHGGGAFSGKDYTKVDRSAAYAARWVAKSLVKAGLCRRVLVQVSYAIGVSHPLSISIFHYGTSQ----K---SERELLE-IVKKNFDLRPGVIVRDLDLKKP---L---YQRTAAYGHFGRD------SFPWEVPKKLKY---------------------------

>MAT1A_Pantholops_hodgsonii

M------------NGP--V----D-------GLC--DHSL-SEEGAFMFTSESVGEGHPDKICDQISDAVLDAHLKQDPNAKVACETVCKTGMVLLCGEITSVAMVDYQRVVREAIKHIGYDDSAKGFDFKTCNVLVALEQQSPDIAQCVHL---DRNEEDVGAGDQGLMFGYATDETEECMPLTIMLAHRLNARMAELRRSGQLPWLRPDSKTQVTVQYRQDNGAVIPMRVHTIVISVQHNEDITLEDMRRALKEQVIRAVVPAQYLDEDTIYHLQPSGRFVIGGPQGDAGVTGRKIIVDTYGGWGAHGGGAFSGKDYTKVDRSAAYAARWVAKSLVKAGLCRRVLVQVSYAIGVAEPLSISIFTYGTSQ----K---TERELLD-VVNKNFDLRPGVIVRDLDLKKP---I---YQKTACYGHFGRS------EFPWEVPKELVF---------------------------

>MAT2A_Pantholops_hodgsonii

M------------NGQ--L----N-------GFH--D-AF-IEEGTFLFTSESVGEGHPDKICDQISDAVLDAHLQ---DAKVACETVAKTGMILLAGEITSRAAVDYQKVVRETIKHIGYDDSSKGFDYKTCNVLVALEQQSPDIAQGVHL---DRNEEDIGAGDQGLMFGYATDETEECMPLTIVLAHKLNAKLAELRRNGTLPWLRPDSKTQVTVQYMQDRGAVLPIRVHTIVISVQHDEEVCLDEMRDALKEKVIKAVVPAKYLDEDTIYHLQPSGRFVIGGPQGDAGLTGRKIIVDTYGGWGAHGGGAFSGKDYTKVDRSAAYAARWVAKSLVKGGLCRRVLVQVSYAIGVSHPLSISIFHYGTSQ----K---SERELLE-IVKKNFDLRPGVIVRDLDLKKP---I---YQRTAAYGHFGRD------SFPWEVPKKLKY---------------------------

>MAT1A_Ficedula_albicollis

M------------NGP--V----D-------GLC--DYTL-DDEGAFMFTSESVGEGHPDKICDQISDAVLDAHLKQDPNAKVACETVCKTGMVLLCGEITSRAIVDYQRVVRDAIRHIGYDDSAKGFDYKTCNVLVALEQQSPDIAQGVHL---HRNEEDVGAGDQGLMFGYATDETEECMPLTIILAHKLNARLAELRRSGELPWLRPDSKTQVTVQYIQKNGAVVPVRVHTIVISVQHDESISLENMRRTLKERVIQVVVPAKYLDDKTVYHLQPSGRFVIGGPQGDAGVTGRKIIVDTYGGWGAHGGGAFSGKDYTKVDRSAAYAARWVAKSLVKAGLCRRVLVQVSYAIGVAHPLSISLFTYGTSQ----K---TEKELLD-IVHKNFDLRPGVIVRDLDLKKP---I---YQKTACYGHFGRQ------EFSWEVPKKLVF---------------------------

>MAT2A_Ficedula_albicollis

M------------NGQ--L----N-------GFH--E-VF-IEEGTFLFTSESVGEGHPDKICDQISDAVLDAHLKQDPDAKVACETVAKTGMILLAGEITSRANVDYQKVVRDTIRHIGYDDSSKGFDYKTCNVLVALEQQSPDIAQGVHL---DRSEEDIGAGDQGLMFGYATDETEECMPLTIVLAHKLNAKLAELRRSGALPWLRPDSKTQVTVQYMQDRGAVIPIRVHTIVISVQHDEDVCLDEMRDALKEKVIKAVVPPKYLDDDTIYHLQPSGRFVIGGPQGDAGLTGRKIIVDTYGGWGAHGGGAFSGKDYTKVDRSAAYAARWVAKSLVKAGLCRRVLVQVSYAIGVSHPLSISIFHYGTSQ----K---SERELLE-IVKKNFDLRPGVIVRDLDLKKP---L---YQRTAAYGHFGRD------SFPWEVPKKLKY---------------------------

>MAT1A_Sturnus_vulgaris

M------------NGP--V----D-------GLC--DYTL-DDEGAFMFTSESVGEGHPDKICDQISDAVLDAHLKQDPNAKVACETVCKTGMVLLCGEITSRAIVDYQRVVRDAIRHIGYDDSAKGFDYKTCNVLVALEQQSPDIAQGVHL---HRNEEDVGAGDQGLMFGYATDETEECMPLTIILAHKLNARLAELRRSGELPWLRPDSKTQVTVQYIQKNGAVIPVRVHTIVISVQHDESISLENMRRTLKERVIQVVVPAKYLDDKTVYHLQPSGRFVIGGPQGDAGVTGRKIIVDTYGGWGAHGGGAFSGKDYTKVDRSAAYAARWVAKSLVKAGLCRRVLVQVSYAIGVAHPLSISLFTYGTSQ----K---TEKELLD-IVHKNFDLRPGVIVRDLDLKKP---I---YQKTACYGHFGRQ------EFSWEVPKKLVF---------------------------

>MAT2A_Sturnus_vulgaris

M------------NGQ--L----N-------GFH--E-VF-IEEGTFLFTSESVGEGHPDKICDQISDAVLDAHLKQDPDAKVACETVAKTGMILLAGEITSRANVDYQKVVRDTIRHIGYDDSSKGFDYKTCNVLVALEQQSPDIAQGVHL---DRSEEDIGAGDQGLMFGYATDETEECMPLTIVLAHKLNAKLAELRRSGALPWLRPDSKTQVTVQYMQDRGAVIPIRVHTIVISVQHDEDVCLDEMRDALKEKVIKAVVPPKYLDDDTIYHLQPSGRFVIGGPQGDAGLTGRKIIVDTYGGWGAHGGGAFSGKDYTKVDRSAAYAARWVAKSLVKAGLCRRVLVQVSYAIGVSHPLSISIFHYGTSQ----K---SERELLE-IVKKNFDLRPGVIVRDLDLKKP---L---YQRTAAYGHFGRD------NFPWEVPKKLKY---------------------------

>MAT2A_Pseudonaja_textilis

M----------------------N-------GFR--D-SR-IEEGTFLFTSESVGEGHPDKICDQISDAVLDAHLKQDPNAKVACETAAKTGMILLAGEITSAAAVDYQKIVRETIKHIGYDDSSKGFDFKTCNVLVALEQQSPDIAQGVHL---DRKEEDIGAGDQGLMFGYATDETDECMPLTLLLAHQLNAKLAELRRNHILTWLRPDSKTQVTVQYKQDRGAVIPIRVHTIVISVQHDDRIGLDEMRDALKEKVVRAVVPAKYLDDDTIYHLQPSGRFVIGGPQGDAGLTGRKIIVDTYGGWGAHGGGAFSGKDYTKVDRSASYAARWVAKSLVKSGLCRRVLVQVSYAIGVAHPISVSIFHYGTSQ----K---TEHELLD-IVKKNFDLRPGVIVRELDLKKP---I---YQKTAAYGHFGRN------NFPWEVPKKLKY---------------------------

>MAT1A_Pseudonaja_textilis

M------------NGT--L----D-------CLQ--DHTLNEEQGAFMFTSESVGEGHPDKICDQISDAVLDAHLKQDPDAKVACETVCKTGMVLLCGEITSRAIVDYQQIVRDTIMNIGYDDSAKGFDYKTCNVLVALEQQSPDIAQGVHL---HRNEEDVGAGDQGLMFGYATDETEECMPLTIILAHKLNAKMAELRRNGVLPWLRPDSKTQITVQYIQEDGAVIPMRVHTIVISVQHDEGISLDSMRKALQEHVIKAVVPAHYLDDNTVYHLQPSGRFVIGGPQGDAGVTGRKIIVDTYGGWGAHGGGAFSGKDYTKVDRSAAYAARWVAKSLVKARLCRRVLVQVAYAIGVAEPLSISLFTYGTSA----Y---TEQELLN-IVHQNFDLRPGAIVKDLDLKKP---I---YQKTACYGHFGRS------EFSWEVPKKLAF---------------------------

>MAT2A_Notechis_scutatus

M----------------------N-------GFR--D-SR-IEDGTFLFTSESVGEGHPDKICDQISDAVLDAHLKQDPNAKVACETAAKTGMILLAGEITSAAAVDYQKIVRETIKHVGYDDSSKGFDFKTCNVLVALEQQSPDIAQGVHL---DRKEEDIGAGDQGLMFGYATDETDECMPLTLLLAHQLNAKLAELRRNHILTWLRPDSKTQVTVQYKQDRGAVIPIRVHTIVISVQHDDRIGLDEMRDALKEKVVRAVVPAKYLDDDTIYHLQPSGRFVIGGPQGDAGLTGRKIIVDTYGGWGAHGGGAFSGKDYTKVDRSASYAARWVAKSLVKSGLCRRVLVQVSYAIGVAHPISVSIFHYGTSQ----K---TEHELLD-IVKKNFDLRPGVIVRELDLKKP---I---YQKTAAYGHFGRN------NFPWEVPKKLKY---------------------------

>MAT1A_Notechis_scutatus

M------------NGT--L----D-------CLQ--DHTLNEEQGAFMFTSESVGEGHPDKICDQISDAVLDAHLKQDPDAKVACETVCKTGMVLLCGEITSRAIVDYQQIVRDTIMNIGYDDSAKGFDYKTCNVLVALEQQSPDIAQGVHL---HRNEEDVGAGDQGLMFGYATDETEECMPLTIILAHKLNAKMAELRQNGVLPWLRPDSKTQITVQYIQEDGAVIPMRVHTIVISVQHDEGISLDSMRKALQEHVIKAVVPAHYLDDNTVYHLQPSGRFVIGGPQGDAGVTGRKIIVDTYGGWGAHGGGAFSGKDYTKVDRSAAYAARWVAKSLVKARLCRRVLVQVAYAIGVAEPLSISLFTYGTSA----Y---TEQELLN-IVHQNFDLRPGAIVKDLDLKKP---I---YQKTACYGHFGRS------EFSWEVPKKLAF---------------------------

>MAT2A_Pogona_vitticeps

M----------------------N-------GFR--D-SR-IEEGTFLFTSESVGEGHPDKICDQISDAVLDAHLKQDPNAKVACETAAKTGMILLAGEITSTAAVDYQKIVRETIKHIGYDDSSKGFDYKTCNVLVALEQQSPDIAQGVHL---DRKEEDVGAGDQGLMFGYATDETEECMPLTLLLAHQLNAKLAELRRNHILPWLRPDSKTQVTVQYRQDRGAVIPIRVHTIVISVQHDEKIGLDEMRDALKEKVVRAVVPAQYLDDDTVYHLQPSGRFVIGGPQGDAGLTGRKIIVDTYGGWGAHGGGAFSGKDYTKVDRSASYAARWVAKSLVKSGLCRRVLVQVSYAIGVAHPLSVSIFHYGTSQ----K---SEHELLE-IVKRNFDLRPGVIVRDLDLKKP---I---YQKTAAYGHFGRN------SFPWEVPKKLKY---------------------------

>MAT1A_Pogona_vitticeps

M------------NGT--L----G-------DLH--DYILNEDEGAFMFTSESVGEGHPDKICDQISDAVLDAHLKQDPDAKVACETVCKTGMVLLCGEITSRAVVDYQQIVRDTIRNIGYDDSAKGFDYKTCNVLVALEQQSPDIAQGVHL---HRSEEEVGAGDQGLMFGYATDETEECMPLTIMLAHKLNAKMADLRRNGILPWLRPDSKTQITVQYIQKDGAVIPVRVHTIVISVQHDETISLDAMRKALREQVIKIVVPARYLDDKTVYHLQPSGRFVIGGPQGDAGVTGRKIIVDTYGGWGAHGGGAFSGKDYTKVDRSAAYAARWVAKSLVKAGLCRRVLVQVAYAIGVAEPLSISLFTYGTST----K---SEQELLN-IVHQNFDLRPGAIVKDLDLKTP---I---YQKTACYGHFGRS------EFSWEVPKKLAF---------------------------

>MAT1A_Terrapene_carolina_triunguis

M------------NGP--V----D-------GLC--DHTV-NDEGAFMFTSESVGEGHPDKICDQISDAVLDAHLQQDPDAKVACETVCKTGMVLLCGEITSLAVVDYQRVVRDTIKQIGYDDSAKGFDYKTCNVLVALEQQSPDIAQGVHL---YRNEEDVGAGDQGLMFGYATDETEECMPLTIVLAHKLNNKLAELRHSGDLPWLRPDSKTQVTVQYIQKNGAVIPVRVHTIVISVQHDETISLEYMRKTLRDCVIKPVVPAKYLDEKTIYHLQPSGRFVIGGPQGDAGVTGRKIIVDTYGGWGAHGGGAFSGKDYTKVDRSAAYAARWVAKSLVKAGLCHRVLVQVSYAIGVAQPLSISLFTYGTSQ----K---TEKELLD-IVHKNFDLRPGVIVRDLDLKKP---I---YQKTACYGHFGRN------EFSWEVPKKLVF---------------------------

>MAT2A_Terrapene_carolina_triunguis

M------------NGQ--L----N-------GFH--E-AF-IDEGSFLFTSESVGEGHPDKICDQISDAVLDAHLKQDPDAKVACETVAKTGMILLAGEITSRAAVDYQKVVRDTIKHVGYDDSSKGFDYKTCNVLVALEQQSPDIAQGVHL---DRNEEDIGAGDQGLMFGYATDETEECMPLTIVLAHKLNAKLADLRRNGTLPWLRPDSKTQVTVQYMQDRGAVIPIRVHTIVISVQHDEDVCLDEMRDALKEKVIKAVVPAKYLDDDTIYHLQPSGRFVIGGPQGDAGLTGRKIIVDTYGGWGAHGGGAFSGKDYTKVDRSAAYAARWVAKSLVKAGLCRRVLVQVSYAIGVSHPLSISIFHYGTSP----K---SERELLE-IVKKNFDLRPGVIVRDLDLKKP---I---YQRTAAYGHFGRD------SFPWELPKKLKY---------------------------

>MAT1A_Anolis_carolinensis

M------------NGT--L----D-------SLE--DYTFNEEAGAFMFTSESVGEGHPDKICDQISDAVLDAHLKQDPNAKVACETVCKTGMVLLCGEITSTAVVDYQQIVRDTIMNIGYDDSSKGFDYKTCNVLVALEQQSPDIAQGVHL---HRSEEEVGAGDQGLMFGYATDETEECMPLTIMLAHKLNAKMAELRRNGILPWLRPDSKTQITVQYVKKDGVAIPLRVHTIVISAQHDETISLDAMRKALREHVIKAVVPPRYLDDKTVYHLQPSGRFVIGGPQGDAGVTGRKIIVDTYGGWGAHGGGAFSGKDYTKVDRSAAYAARWVAKSLVKAGLCRRVLVQVAYAIGVAEPLSVSLFTYGTST----R---TEQELLN-IVHQNFDLRPGAIVKDLDLKKP---I---YQKTACYGHFGRS------EFSWEVPKKLAF---------------------------

>MAT2A_Anolis_carolinensis

M----------------------N-------GLR--D-SR-IEEGTFLFTSESVGEGHPDKICDQISDAVLDAHLKQDPNAKVACETAAKTGMILLAGEITSSAAVDYQKIVRETIKHIGYDDSSKGFDYKTCNVLVALEQQSPDIAQGVHL---DRKEEDIGAGDQGLMFGYATDETEECMPLTLLLAHQLNAKLAELRRNHTLPWLRPDSKTQVTVQYRQDRGAVIPIRVHTIVISVQHDEKIGLDEMRDALKEKVVRAVVPSRYLDDDTVYHLQPSGRFVIGGPQGDAGLTGRKIIVDTYGGWGAHGGGAFSGKDYTKVDRSASYAARWVAKSLVKSGLCRRVLVQVSYAIGVAHPLSVSIFHYGTSQ----K---SEHELLE-IVKKNFDLRPGVIVRDLDLKKP---M---YQKTAAYGHFGRN------NFPWEVPKKLKY---------------------------

>MAT1A_Chelonia_mydas

M------------NRL--V----D-------GLC--DHTV-NEEGAFMFTSESVGEGHPDKICDQISDAVLDAHLQQDPDAKVACETVCKTGMVLLCGEITSLAIVDYQRVVRDTIKQIGYDDSAKGFDYKTCNVLVALEQQSPDIAQGVHM---YRNEEDVGAGDQGLMFGYATDETEECMPLTIVLAHKLNTKLAELRHSGDLPWLRPDSKTQVTVQYIQKNGAVIPVRVHTIVISVQHDETISLEYMRKTLRDCVIKPVVPAKYLDEKTIYHLQPSGRFVIGGPQGDAGVTGRKIIVDTYGGWGAHGGGAFSGKDYTKVDRSAAYAARWVAKSLVKAGLCHRVLVQVSYAIGVAQPLSISLFTYGTSQ----R---TEKELLD-IVHKNFDLRPGVIVKDLDLKKP---I---YQKTACYGHFGRN------EFSWEVPKKLVF---------------------------

>MAT2A_Chelonia_mydas

M------------NGQ--L----N-------GFH--E-AF-IDEGSFLFTSESVGEGHPDKICDQISDAVLDAHLKQDPDAKVACETVAKTGMILLAGEITSRAAVDYQKVVRDTIKHVGYDDSSKGFDYKTCNVLVALEQQSPDIAQGVHL---DRNEEDIGAGDQGLMFGYATDETEECMPLTIVLAHKLNAKLADLRRNGTLPWLRPDSKTQVTVQYMQDRGAVIPIRVHTIVISVQHDEEVCLDEMRDALKEKVIKAVVPAKYLDDDTIYHLQPSGRFVIGGPQGDAGLTGRKIIVDTYGGWGAHGGGAFSGKDYTKVDRSAAYAARWVAKSLVKAGLCRRVLVQVSYAIGVSHPLSISIFHYGTSP----K---SERELLE-IVKKNFDLRPGVIVRDLDLKKP---I---YQRTAAYGHFGRD------SFPWELPKKLKY---------------------------

>MAT1A_Gavialis_gangeticus

M------------NGP--I----D-------GLY--DHTL-DDEWEFMFTSESVGEGHPDKICDQISDAVLDAHLKQDPNAKVACETVCKTGMVLLCGEITSLAVVDYQRVVRDTIRHIGYDDSAKGFDYKTCNVLVALEQQSPDIAQGVHL---HRNEEDVGAGDQGLMFGYATDETEECMPLTIILAHKLNAKLAEMRRSGELPWLRPDSKTQVTVQYVQEKGAVIPVRVHTIVISVQHDEGISLETMQKALKDRVISAVVPAKYLDEKTVYHLQPSGRFVIGGPQGDAGVTGRKIIVDTYGGWGAHGGGAFSGKDYTKVDRSAAYAARWVAKSLVKAGLCRRVLVQVSYAIGVAEPLSISLFTYGTSQ----K---TERELLG-IVHKNFDLRPGVIVRDLDLKKP---I---YQKTACYGHFGRN------EFSWEIPKKLAY---------------------------

>MAT2A_Gavialis_gangeticus

M------------NGQ--L----N-------GFH--E-AF-IEEGSFLFTSESVGEGHPDKICDQISDAVLDAHLKQDPDAKVACETVAKTGMILLAGEITSRAAVDYQKVVRDTIKHVGYDDSSKGFDYKTCNVLVALEQQSPDIAQGVHL---DRSEEDIGAGDQGLMFGYATDETEECMPLTIVLAHKLNAKLAELRRNGTLPWLRPDSKTQVTVQYMQDRGAVLPIRVHTIVISVQHDEDVSLDEMRDALKEKVIKAVVPARYLDDDTIYHLQPSGRFVIGGPQGDAGLTGRKIIVDTYGGWGAHGGGAFSGKDYTKVDRSAAYAARWVAKSLIKAGLCRRVLVQVSYAIGVSHPLSISIFHYGTSQ----K---TERELLD-IVKKNFDLRPGVIVRELDLKKP---I---YQRTAAYGHFGRD------SFPWEVPKKLKY---------------------------

>MAT2A_Nanorana_parkeri

M------------NGQ-LL----N-------GFH--D-AL-IDEGSFLFTSESVGEGHPDKICDQISDAVLDAHLKQDPDAKIACETVAKTGMILLAGEITSRAVVDYQKIVRDTIKHIGYDDSSKGFDYKTCNVLVALEQQSPDIAQGVHL---DRNEEDVGAGDQGLMFGYASDETEECMPLTIMLAHKLNAKMADLRRNGTLPWLRPDSKTQVTVQYMQDRGAVIPVRVHTVVVSVQHDDGICLDEMRDALKEKVVKAVIPAKYLDDDTIFHIQPSGRFVIGGPQGDAGLTGRKIIVDTYGGWGAHGGGAFSGKDYTKVDRSAAYAARWVAKSLVKAGLCRRVLVQVAYAIGVAHPLSISIFHYGTSQ----K---SERELLA-IVNKNFDLRPGVIVRDLELKKA---N---YQRTASYGHFGRE------NFPWEVPRKLEY---------------------------

>MAT1A_Nanorana_parkeri

M------------NGP--V----D-------GLH--DHSK-EDVGAFMFTSESVGEGHPDKICDQISDAVLDAHLRQDPDAKVACETVCKTGMVLLCGEITSRAVVDYQKVVRDTIKYIGYDDSEKGFDYKTCNVLVALEQQSPDIAQGVHL---DRIEEDIGAGDQGLMFGYATDETKECMPLTIVLAHKLNSKLAELRRNGELPWLRPDSKTQVTVKYIQKNGAVIPVRVHTIVISVQHDETISLSDMQEALKEKVIKAVVPDKYLNEKTIYHLQPSGRFVIGGPQGDAGVTGRKIIVDTYGGWGAHGGGAFSGKDSTKVDRSAAYAARWVAKSLVHAKLCHRVLVQVAYAIGVAYPLSVSLFTYGTSE----K---TEKELLD-IVNKNFDLRPGVIVRDLDLKRP---I---YQNTACYGHFGRE------DFPWEVPKELVF---------------------------

>Mat2a_Xenopus_tropicalis

M------------NGQ-ML----N-------GFH--D-DL-IDEGSFLFTSESVGEGHPDKICDQISDAVLDAHLKQDPDAKVACETVAKTGMILLAGEITSRASVDYQKIVRDTIKHIGYDDSSKGFDYKTCNVLVALEQQSPDIAQGVHL---DRNEEDVGAGDQGLMFGYATDETEEGMPLTIVLAHKLNARMAELRRNGTLPWLRPDSKTQVTVQYMQDRGAVIPIRVHTIVVSVQHDETICLDEMRDALKEKIVKAVVPAKYLDDDTIYHLQPSGRFVIGGPQGDAGLTGRKIIVDTYGGWGAHGGGAFSGKDYTKVDRSAAYAARWVAKSLVKSGLCRRVLVQVAYAIGVSHPLSISIFHYGTSQ----K---SERELLA-VVKKNFDLRPGVIVRDLELKKP---I---YQRTAAYGHFGRD------SFPWEVPKKLEY---------------------------

>Mat1a_Xenopus_tropicalis

M------------NGP--V----D-------YIQ--EHAV-EDVGAFMFTSESVGEGHPDKICDQISDAVLDAHLSQDPDAKVACETVCKTGMVLLCGEITSRAVVDYQKIVRDTIKYIGYDDSEKGFDYKTCNVLVALEQQSPDIAQGVHL---DRTEEDIGAGDQGLMFGYATDETEECMPLTIVLAHKLNSKLAELRRNGVLPWLRPDSKTQVTVKYIQKNGAVIPVRVHTIVISVQHDETISLSDMQEALKEHVIKAVVPAKYLDEKTVYHLQPSGRFVIGGPQGDAGVTGRKIIVDTYGGWGAHGGGAFSGKDYTKVDRSAAYAARWVAKSLVHAKLCHRVLVQVSYAIGVAYPLSVSLFTYGTSE----K---TEKELLD-IVNANFDLRPGVIVRDLDLKKP---L---YQKTACYGHFGRE------DFPWEVPKELFF---------------------------

>Mat2ab_Danio_Rerio

M------------NGQ--I----N-------GFH--N-SL-IEEDCFLFTSESVGEGHPDKICDQISDAVLDAHLKQDPDAKVACETVAKTGMILLAGEVTSRAVVDYQKVVRDTIKHIGYDDSTKGFDYKTCNVLVALEQQSPDIAQGVHL---ERDEQDVGAGDQGLMFGYATDETEECMPLTIVLAHKLNSKMAELRRNGTLPWLRPDSKTQVTVQYRQEHGAMLPIRVHTIVISVQHDEDICLDEMRDALKDKVINTVVPSMYLDDDTIYHLQPSGRFVIGGPQGDAGLTGRKIIVDTYGGWGAHGGGAFSGKDYTKVDRSAAYAARWVAKSLVKAKLCRRVLVQVSYAIGVAHPLSISIFHYGTSQ----K---SEQELLK-IVKKNFDLRPGVIVRELELKKP---I---YQKTAAYGHFGRE------SFSWEVPKKLHY---------------------------

>Mat2aa_Danio_Rerio

M------------NGQ--L----N-------GFH--N-SL-IDEDCFLFTSESVGEGHPDKICDQISDAVLDAHLKQDPDAKVACETVAKTGMILLAGEITSHAVVDYQKVVRDTIKHIGYDDSSKGFDYKTCNVLVALEQQSPDIAQGVHL---DRNEEDVGAGDQGLMFGYATDETEECMPLTIVLAHKLNAKMAELRRNGTLPWLRPDSKTQVTVQYRQDRGAMLPVRVHTIVVSVQHDDVVCLDEMRDALKEKVVKAVVPNVYLDDDTIYHLQPSGRFVIGGPQGDAGLTGRKIIVDTYGGWGAHGGGAFSGKDYTKVDRSAAYAARWVAKSLVKAGLCKRVLVQVSYAIGVAHPLSVSIFHYGTSQ----R---SEKELLE-IVKKNFDLRPGVIVRELDLKKP---I---YQRTAAYGHFGRE------SFPWEVPKKLKY---------------------------

>Mat1a_Danio_Rerio

M------------EGL--N----D---------------V-HDDGSFMFTSESVGEGHPDKICDQISDAVLDAHLKQDPDAKVACETVCKTGMVLLCGEITSRANVDYQKIVRDTIKHIGYDNSEKGFDYKTCNVLVALEQQSPDIAQGVHV---DRHEEDIGAGDQGLMFGYATDETEECMPLTIVLAHKLNSKMAELRRDGTIPWLRPDSKTQVTVHYKQENGAVIPLRVHTVVISVQHDDNISLEEQQRILKEKVIKAVVPARYLDDKTVYHLQPSGRFVIGGPQGDAGVTGRKIIVDTYGGWGAHGGGAFSGKDYTKVDRSAAYAARWVAKSLVKSKLCRRVLVQVSYAIGVAHPLSISLFTYGSSE----K---TEKELLH-IVNKNFDLRPGVIVRDLNLKRP---F---YQNTACYGHFGRS------EFPWEMAKTLKV---------------------------

>Mat1a_Latimeria_chalumnae

M------------NGP--V----D-------GLC--------NDSTFMFTSESVGEGHPDKICDQISDAVLDAHLKQDPDAKVACETVCKTGMVLLCGEITSRAIVDYQKVVRDAIKAIGYDDSAKGFDFKTCNVLVALEQQSPDIAQGVHL---DRHEEDIGAGDQGLMFGYATDETEEFMPLTIMLAHKLNTKLAELRRDGTLPWLRPDSKTQVTVQYNQKNGAVIPIRVHTVVISVQHDEDISLEEMQKALKEKVIRAVVPAKYLDDKTVYHLQPSGRFVIGGPQGDAGVTGRKIIVDTYGGWGAHGGGAFSGKDYTKVDRSAAYAARWVAKSLVKAGLCRRLLVQVSYAIGVAEPLSISLFTYGTSA----K---TEKELLE-IVHKNFDLRPGVIVRDLDLKRP---I---YQHTACYGHFGRN------EFPWEVPKDIVF---------------------------

>Mat2a_Latimeria_chalumnae

M------------NGI--L----N--------------SL-IEEGSFLFTSESVGEGHPDKICDQISDAVLDAHLLQDPNAKVACETVAKTGMILLAGEITSGAIVDYQKVVRDTIKHVGYDDSSKGFDYKTCNVLVALEQQSPDIAQGVHL---DRSEEDVGAGDQGLMFGYATDETEESMPLTIMLAHKLNAKMAELRRNGTLPWLRPDSKTQVTVQYMQDRGAVIPIRVHTIVISVQHDEEIQLDEMRDALKEKIVKSVVPSKYLDEDTIFHLQPSGRFVIGGPQGDAGVTGRKIIVDTYGGWGAHGGGAFSGKDYTKVDRSAAYAARWVAKSLVKAGLCRRVLVQVSYAIGVAHPLSISIFHYGTSQ----K---SERELLD-IVVKNFDLRPGVIVRELDLKKP---I---YQRTAAYGHFGRE------NFPWEVPRKLKY---------------------------

>MAT_Ciona_intestinalis

MPETM-----ISQNGF-------N-------G------DG-HEEETFLFTSESVGEGHPDKLCDQVSDAILDAHLAIDPNAKVACETFAKTGMVLVGGEITSKAVIDYQKVIRDTIKHIGYDDSSKGFDCKTCNVLLAVEQQSPEIADAVHI---GKKEEDIGAGDQGLMFGYATDETEECMPLTVTLAHRLNEKLSELRRDGTLAWLRPDSKTQVTIEHRLEHGAVVPIRVHTIVISTQHDEQISLEKMRKEILEKIIKAVVPSQYLDENTIYHIQPSGKFITGGPMGDAGLTGRKIIVDTYGGWGAHGGGAFSGKDPSKVDRSAAYAARWVAKSFVKSGICRRVLVQVSYAIGIAEPMAVYVHSYGTSK----Y---TNPQLHD-IAMHNFDLRPGIITKSLNLKTP---I---YFKTSVNGHFGSS------EFSWEVPKKIVI---------------------------

>Metazoa_Strongylocentrotus_purpuratus

MAQEN----------------------------H--NSSV-AEGDTFLFTSESVGEGHPDKICDQVSDAVLDAHLAIDPNARVACECASKTGMIMVFGEVTSNAHVDYQTCVRDAIKAIGYDDSSQGFDYDTCNVLVAIEQQAREIANSVHV---GKAEDDIGAGDQGLMFGYASDETEECMPLTCVLAHALTQKLAEIRHGGD-RRLRPDCKSQVTVEYKMDRGACVPLRVHTIVISTQHSEDITLPDLQKLLREQVIDVVIPKKYLDENTVYHLNPSGSFLIGGPKGDAGLTGRKIIVDTYGGWGAHGGGAFSGKDFSKVDRSAAYAARWVAKSLVKAQLCKRVLVQVAYSIGISEPLSITVFHYGTSE----Y---SEKELLR-IVNTNFDLRPGAIVKALDLKKP---I---YRKTATFGHFGRS------EFTWEQPKKLVL---------------------------

>Metazoa_Apostichopus_japonicus

ME------------------------------------------DSSVCT---------NKICDQVSDAVLDAYLEQDPNAKVACETASKTGMIMVFGEITSSGIVDYQKVVRQTIKEIGYDSSDKGFDYKTCNVLVAIEKQAAEIANTVHI---DKAEDDIGAGDQGLMFGYATDETESAMPLTVELAHGLTRRLAELRRSSE-PRLRPDCKSQVTVEYRMDKGACVPIRVHTIVISTQHSPDISLKELQEVLREDVVKYVIPPKYLDGDTILHLNPSGSFIIGGPQGDAGLTGRKIIVDTYGGWGAHGGGAFSGKDFSKVDRSAAYAARWVAKSLVKAGLAKRVLVQVAYSIGIAEPLSITVFHYGTSI----H---SEKQLLA-IVKKNFDLRPGSIVKELGLKKP---I---YRKTASYGHFGRK------EFSWEQPKKLIL---------------------------

>Insecta_Lucilia_cuprina

MPQTL-------TNGH--S----NGCNG---NGT--QYDM-EDGQSFLFTSESVGEGHPDKMCDQISDAILDAHLKQDPDAKVACETVAKTGMILLCGEITSKAVVDYQKVVRETVKHIGYDDSSKGFDYKTCNVLLALDQQSPEIAAGVHI---NRCEEEIGAGDQGIMFGYATDETEECMPLTVVLAHKLNEKIAELRRSGDFWWARPDSKTQITCEYLFDQGAAVPKRVHTIVVSLQHSEKIALEDLRSEVMNKVIKVVIPAKYLDANTVVHINPCGLFVIGGPMGDAGLTGRKIIVDTYGGWGAHGGGAFSGKDYTKVDRSAAYAARWVAKSLVKAGLCKRCLVQVSYAIGLAEPLSITVFDYGTSH----K---SQKELLQ-IVKKNFDLRPGMIVKDLNLKHP---I---YQRTSTYGHFGRD------GFAWEQAKSLQIN--------------------------

>Insecta_Harpegnathos_saltator

MPETA-----HHMNGY--A----N-------GHT--PPEL-QQGTTFLFTSESVGEGHPDKMCDQISDAILDAHLKQDPDAKVACETVTKTGMILLCGEITSKAVVDYQKIVRDTVNHIGYDDSSKGFDYKLCNVLLALDAQSPNIAAGVHE---NRSDEEVGAGDQGLMFGYATDETDECMPLTVVLAHKLNQKIAELRRSGELWWARPDSKTQVTCEYIMDHGACVPIRVHTVVVSLQHSEKIGLDELRKAVMEKVIKEVIPARYLDDRTIFHVNPCGLFIIGGPQSDAGLTGRKIIVDTYGGWGAHGGGAFSGKDFTKVDRSAAYAARWVAKSLVKAGLCRRCLVQVSYAIGVAEPLSITVFDYGTST----R---SQNELLD-IVNKNFDLRPGKIVKELNLRNP---I---YQQTSTYGHFGRD------GFTWEQPKKLILD--------------------------

>Insecta_Cryptotermes_secundus

MPETS----SPYINGF-QQ----N-------GHM--EPEL--IEDTFLFTSESVGEGHPDKMCDQISDAILDAHLSQDPDAKVACETVTKTGMILLCGEITSKANVDYQKVVRETVKHIGYDDSSKGFDYKICNVLLALDQQSPNIAAGVHL---NRSDDDVGAGDQGLMFGYATDETEECMPLTVVLAHKLNQRIAELRRSGEFWWARPDSKTQVTCEYCMYHGACIPLRVHTVVVSVQHSEKISLEELRADVMTKVIRVVIPEKYLDDGTTFHINPCGLFVVGGPQSDAGLTGRKIIVDTYGGWGAHGGGAFSGKDFTKVDRSAAYAARWVAKSLVKSGLCRRCLVQVSYAIGVAEPLSITLFDYGTSK----K---TQKELLA-IVKKNFDLRPGKIVKELNLRNP---I---YQQTSTYGHFGRD------MFPWEQPKKLVD---------------------------

>Insecta_Bactrocera_dorsalis

MPLTS-----AATNGH--T----NGCNGK--TTD--SYDM-EDGQTFLFTSESVGEGHPDKMCDQISDAILDAHLSQDPNAKVACETVAKTGMILLCGEITSQAVIDYQKVVRETVKHIGYDDSSKGFDYKTCNVLLALDQQSPEIAAGVHI---NRADEEIGAGDQGIMFGYATDETEECMPLTVVLAHKLNEKLAELRRSGEFSWARPDSKTQVTCEYLFNQGAAVPKRVHTIVVSMQHSEKIALEDLRKEVMNKVIKEVIPAKYFDENTIVHINPCGLFVIGGPMGDAGLTGRKIIVDTYGGWGAHGGGAFSGKDFTKVDRSAAYAARWVAKSLVKAGLCRRCLVQVSYAIGLAEPLSITVFDYGTSH----K---SQKELLD-IVKRNFDLRPGMIVKDLKLRQP---I---YQRTSTYGHFGRD------GFTWEQAKPLQIN--------------------------

>Insecta_Temnothorax_curvispinosus

MPETA-----HHMNGY--A----N-------GHA--PPEL-QQDTSFLFTSESVGEGHPDKMCDQISDAILDAHLTQDPDAKVACETVTKTGMILLCGEITSKAVVDYQKIVRDTVQHIGYDDSSKGFDYKLCNVLLALDAQSPNIAAGVHE---NRSDEEVGAGDQGLMFGYATDETDECMPLTVVLAHKLNQKIAELRRSGELWWARPDSKTQVTCEYIMDHGACVPIRVHTVVVSLQHSEKIGLDELRKAVMEKVIKEVIPARYLDERTIFHVNPCGLFIIGGPQSDAGLTGRKIIVDTYGGWGAHGGGAFSGKDFTKVDRSAAYAARWVAKSLVKAGLCRRCLVQVSYAIGVAEPLSITVFDYGTST----R---SQNELLD-IVNKNFDLRPGKIVKELNLRNP---I---YQQTSTYGHFGRD------GFTWEQPKKLILD--------------------------

>Insecta_Monomorium_pharaonis

MPETA-----HHMNGY--A----N-------GHA--PPEL-QQDTAFLFTSESVGEGHPDKMCDQISDAILDAHLKQDPDAKVACETVTKTGMILLCGEITSKAVVDYQKIVRDTVKHIGYDDSSKGFDYKLCNVLLALDAQSPNIAAGVHE---NRSDEEVGAGDQGLMFGYATDETDECMPLTVVLAHKLNQKIAELRRSGELWWARPDSKTQVTCEYIMDHGACVPIRVHTVVVSLQHSEKIGLDELRKAVMEKVIKEVIPARYLDERTVFHVNPCGVFNIGGPQSDAGLTGRKIIVDTYGGWGAHGGGAFSGKDFTKVDRSAAYAARWVAKSLVKAGLCRRCLVQVSYAIGVAEPLSITVFDYGTST----R---SQNELLD-IVNKNFDLRPGKIVKELNLRNP---I---YQQTSTYGHFGRD------GFTWEQPKKLILDD-------------------------

>Insecta_Nylanderia_fulva

MPETA-----HHMNGY--A----N-------GHA--PPEL-QQDTTFLFTSESVGEGHPDKMCDQISDAILDAHLKQDPDAKVACETVTKTGMILLCGEITSKAVVDYQKIVRDTVKHIGYDDSSKGFDYKLCNVLLALDAQSPNIAAGVHE---NRSDEEVGAGDQGLMFGYATDETDECMPLTVVLAHKLNQKIAELRRSGELWWARPDSKTQVTCEYIMDHGACVPIRVHTVVVSLQHSEKIGLDELRKAIMEKVIKEVIPARYLDDRTIFHVNPCGLFIIGGPQSDAGLTGRKIIVDTYGGWGAHGGGAFSGKDFTKVDRSAAYAARWVAKSLVKAGLCRRCLVQVSYAIGVAEPLSITVFDYGTST----L---SQNELLD-IVNKNFDLRPGKIVKELNLRNP---I---YQQTSTYGHFGRD------GFTWEQPKKLILD--------------------------

>Insecta_Sitophilus_oryzae

MPDTV-------TNGY--T----N-------GHM--PYDM-EDGSNFLFTSESVGEGHPDKMCDQISDAVLDAHLQQDPDAKVACETVTKTGMILLCGEITSKANVDYQKVVRETVKHIGYDDSSKGFDYKTCSVMLALDAQSPNIAAGVHE---HRNEEEIGAGDQGLMFGYATDETEECMPLTVVLAHKLNEKIAELRRAGEFWWARPDSKTQVTCEYTFNHGACVPQRVHTIVVSLQHSEKINLDELRDAIKTKVIKEVIPAKYLDDQTIVHINPCGLFIIGGPQSDAGLTGRKIIVDTYGGWGAHGGGAFSGKDFTKVDRSAAYAARWVAKSLVKAGVCRRCLVQVSYAIGLAEPLSISVFDYGTSK----L---NQKQLLE-IVHNNFDLRPGKIVKELNLRHP---I---YQKTSTYGHFGRS------GFTWETPKPLKLGNLANLI--------------------

>Insecta_Zeugodacus_cucurbitae

MPLTS-----ATTNGH--S----NGCNGK--TTD--SYDM-EDGQTFLFTSESVGEGHPDKMCDQISDAILDAHLKQDPNAKVACETVAKTGMILLCGEITSQANIDYQKVVRETVKHIGYDDSSKGFDYKTCNVLLALDQQSPEIAAGVHI---NRADEEIGAGDQGIMFGYATDETEECMPLTVVLAHKLNEKLAELRRSGEFSWARPDSKTQVTCEYLFNQGAAVPKRVHTIVVSMQHSEKITLEDLRKEVMNKVIKEVIPAKYFDANTIVHINPCGLFVIGGPMGDAGLTGRKIIVDTYGGWGAHGGGAFSGKDFTKVDRSAAYAARWVAKSLVKAGLCRRCLVQVSYAIGLAEPLSITVFDYGTSH----K---SQKELLD-IVKRNFDLRPGMIVKDLKLRQP---I---YQRTSTYGHFGRD------GFAWEQAKPLQIN--------------------------

>Insecta_Ceratina_calcarata

MPETA-----HHMNGY--A----N-------GHT--PPEL-QQGTSFLFTSESVGEGHPDKMCDQISDAILDAHLTQDPDAKVACETVTKTGMILLCGEITSKAVVDYQKIVRDTVKHIGYDDSSKGFDYKLCSVLLALDAQSPNIAAGVHE---NRSDEEVGAGDQGLMFGYATDETDECMPLTVVLAHKLNQKIAELRRSGELWWARPDSKTQVTCEYLMDHGACVPMRVHTVVVSLQHSEKIGLDELRKAVMEKVIKEVIPARYLDERTIFHVNPCGLFIIGGPQSDAGLTGRKIIVDTYGGWGAHGGGAFSGKDFTKVDRSAAYAARWVAKSLVKAGLCRRCLVQVSYAIGVAEPLSITVFDYGTSK----R---PQNELLD-IVNKNFDLRPGKIVKELNLRNP---I---YQQTSTYGHFGRD------GFTWEQPKTLTLD--------------------------

>Insecta_Formica_exsecta

MPETA-----HHMNGY--T----N-------GHA--PPEL-QQDTTFLFTSESVGEGHPDKMCDQISDAILDAHLKQDPDAKVACETVTKTGMILLCGEITSKAVVDYQKIVRDTVKHIGYDDSSKGFDYKLCNVLLALDAQSPNIAAGVHE---NRSDEEVGAGDQGLMFGYATDETDECMPLTVVLAHKLNQKIAELRRSGELWWALPDSKTQVTCEYIMDHGACVPIRVHTVVVSLQHSEKIGLDELRKAIMEKVIKEVIPARYLDDRTVFHVNPCGLFIIGGPQSDAGLTGRKIIVDTYGGWGAHGGGAFSGKDFTKVDRSAAYAARWVAKSLVKAGLCRRCLVQVSYAIGVAEPLSITVFDYGTST----R---SQNELLD-IVNKNFDLRPGKIVKELNLRNP---I---YQQTSTYGHFGRD------GFTWEQPKKLILD--------------------------

>Insecta_Ooceraea_biroi

MPETA-----HHMNGY--A----N-------GHA--PPEL-QQDTTFLFTSESVGEGHPDKMCDQISDAILDAHLKQDPNAKVACETVTKTGMILLCGEITSKAVVDYQKIVRDTVNHIGYDDSSKGFDYKLCNVLLALDAQSPNIAAGVHE---NRSDEEVGAGDQGLMFGYATDETDECMPLTVVLAHKLNQKIAELRRSGELWWARPDSKTQVTCEYIMDHGACVPIRVHTVVVSLQHSEKIGLDELRKAVMEKVIKEVIPARYLDDRTVFHVNPCGLFIIGGPQSDAGLTGRKIIVDTYGGWGAHGGGAFSGKDFTKVDRSAAYAARWVAKSLVKAGLCRRCLVQVSYAIGVAEPLSITVFDYGTST----R---SQNELLD-IVNKNFDLRPGKIVKELNLRNP---I---YQQTSTYGHFGRD------GFTWEQPKKLILD--------------------------

>Insecta_Copidosoma_floridanum

MPENA-----HHMNGY--A----N-------GHT--APEL-QQDTSFLFTSESVGEGHPDKMCDQISDAILDAHLKQDPDAKVACETVTKTGMVLLCGEITSKAVVDYQKIVRDTVKHIGYDDSSKGFDWRTLNLLVAIEQQSPNIAGGVHV---DREEVDVGAGDQGLMFGYATDETDECMPLTVVLAHKLNQKIAELRRSGDLWWARPDSKTQVTCEYIMDHGACVPIRVHTVVVSLQHSEKISLDELRKAIMEKVIKEVIPARYLDEKTIFHVNPCGLFIIGGPQSDAGLTGRKIIVDTYGGWGAHGGGAFSGKDFTKVDRSAAYAARWVAKSLVKAGLCRRCLVQVAYAIGVAEPLSITVFDYGTSK----Y---SQSQLLE-IVNKNFDLRPGKIVKELNLRNP---I---YQQTSSYGHFGRD------CFSWEQPKSLTID--------------------------

>Insecta_Bicyclus_anynana

MPETA------KVNGYAKT----N-------GH---SYDM-EDGSVFLFTSESVGEGHPDKMCDQISDAILDAHLSQDPDAKVACETVTKTGMILLCGEITSKANVDYQKVVRETVKHIGYDDSSKGFDYKTCSVMLALDQQSPDIAAGVHE---NRNEEEVGAGDQGLMFGYATDETEECMPLTVVLAHRLNQKIAELRRNGEFWWARPDSKTQVTAEYVFAGGATVPQRVHTVVVSVQHSEKICLETLRDEIKMKVIEEVIPPQYLDARTVIHINPCGNFIIGGPQSDAGLTGRKIIVDTYGGWGAHGGGAFSGKDFTKVDRSAAYAARWVAKSLVRAGLCRRCMVQVAYAIGVAEPLSITVFDYGTSH----K---TQQELLS-IVKKNFDLRPGKIVKDLNLRAP---I---YQKTSTYGHFGRE------GFPWENPKPL-VD--------------------------

>Insecta_Orussus_abietinus

MPETA-----HHMNGY--A----N-------GHT--APEL-QQGTSFLFTSESVGEGHPDKMCDQISDAILDAHLQQDPDAKVACETVTKTGMILLCGEITSKAVVDYQKIVRDTVNHIGYDDSSKGFDWRTLNLLVAIEQQSPNIADGVHL---NREEKNVGAGDQGLMFGYATDETDECMPLTVVLAHKLNQKIAELRRSGELWWARPDSKTQVTCEYIMDHGACVPIRVHTVVVSLQHSEKITLEELRKAVMDKVIRDVIPAKYLDDRTVFHVNPCGLFIIGGPQSDAGLTGRKIIVDTYGGWGAHGGGAFSGKDFTKVDRSAAYAARWVAKSLVKADLCRRCLVQVSYAIGVAEPLSITVFDYGTSK----L---SQNELLD-IVNKNFDLRPGKIVKELNLRNP---I---YQQTSTYGHFGRD------GFTWEQPKTLVLD--------------------------

>Insecta_Pseudomyrmex_gracilis

MPETA-----HHMNGY--A----N-------GHA--PPEL-QQDTTFLFTSESVGEGHPDKMCDQISDAILDAHLMQDPNAKVACETVTKTGMILLCGEITSKAVVDYQKIVRDTVKHIGYDDSSKGFDYKLCNVLLALDAQSPNIAAGVHE---NRSDEEVGAGDQGLMFGYATDETDECMPLTVVLAHKLNQKIAELRRSGELWWARPDSKTQVTCEYIMDHGACVPIRVHTVVVSLQHSEKIGLDELRLAVMEKVIKEVIPARYLDDRTVFHVNPCGLFIIGGPQSDAGLTGRKIIVDTYGGWGAHGGGAFSGKDFTKVDRSAAYAARWVAKSLVKAGLCRRCLVQVSYAIGVAEPLSITVFDYGTST----L---SQNELLD-IVNKNFDLRPGKIVKELNLRNP---I---YQQTSTYGHFGRD------GFTWEQPKKLILD--------------------------

>Insecta_Osmia_bicornis_bicornis

MPETA-----HHMNGY--A----N-------GHT--PPEL-QQDTSFLFTSESVGEGHPDKMCDQISDAILDAHLQQDPDAKVACETVTKTGMVLLCGEITSKAVVDYQKIVRDTVKHIGYDDSSKGFDWRTLNLLVAIEQQSPNIADGVHV---DREEKDVGAGDQGLMFGYATDETDECMPLTVVLAHKLNQKIAKLRRSGELWWARPDSKTQVTCEYVMDHGACVPIRVHTVVVSLQHSEKIGLEELRKAVMEKVIKEVIPARYLDDRTIFHVNPCGLFIIGGPQSDAGLTGRKIIVDTYGGWGAHGGGAFSGKDFTKVDRSAAYAARWVAKSLVKAGLCRRCLVQVSYAIGVAEPLSITVFDYGTSK----L---SQNELLN-IVNKNFDLRPGKIVKELNLRNP---I---YQQTSTYGHFGRE------GFTWEQPKALILD--------------------------

>Insecta_Anoplophora_glabripennis

MPETT------LTNGY--T----N-------GHI--GYEM-EEDSEFLFTSESVGEGHPDKMCDQISDAILDAHLEQDPNAKVACETVTKTGMILLCGEITSKAVVDYQKVVRETVKHIGYDDSSKGFDYKTCNVLLALDQQSPNIAAGVHE---NKNEEEIGAGDQGLMFGYATDETDECMPLTVVLAHRLNHKIADLRRSGEFWWARPDSKTQVTAEYTFDHGACIPQRVHTVVVSLQHSEKISLDELREAVKEKVIKEVIPARYLDEQTVIHINPCGLFIIGGPQSDAGLTGRKIIVDTYGGWGAHGGGAFSGKDFTKVDRSAAYAARWVAKSLVKAGLCRRCLVQVSYAIGLAEPLSITVFDYGTSK----L---TQKQLLD-VVKNNFDLRPGKIVKELNLRHP---I---YQKTSTYGHFGRE------DFTWEQPKVLRLN--------------------------

>Insecta_Athalia_rosae

MPETS-----YHMNGH--A----N-------GHM--PPEL-QQGTSFLFTSESVGEGHPDKMCDQISDAILDAHLQQDPNAMVACETVTKTGMILLCGEITSKAVVDYQKIVRDTVKHIGYDDSSKGFDYKLCNVLLALDAQSPNIAAGVHE---NRSDEEVGAGDQGLMFGYATDETEECMPLTVVLAHRLNQKIAELRRSEELWWARPDSKTQVTCEYIMDHGACVPSRVHTVVVSLQHSEKISLDELRKAVMEKVIKTVIPAKYLDERTVFHVNPCGLFIIGGPQSDAGLTGRKIIVDTYGGWGAHGGGAFSGKDFTKVDRSAAYAARWVAKSLVKAGLCRRCLVQVSYAIGVAEPLSITVFDYGTSK----L---SQTKLLD-IVNKNFDLRPGKIVKELNLRNP---I---YQQTSTYGHFGRE------GFSWEQPKILNLD--------------------------

>Insecta_Apis_mellifera

MPETA-----HHMNGY--A----N-------GHT--PPEL-QQEGSFLFTSESVGEGHPDKMCDQISDAILDAHLKQDPNAKVACETVTKTGMVLLCGEITSKAVVDYQKIVRDTVKHIGYDDSSKGFDFKLCSVLLALDAQSPNIAAGVHE---NRSDEEVGAGDQGLMFGYATDETDECMPLTVVLAHKLNQKIAELRRSGELWWARPDSKTQVTCEYVMDHGACVPIRVHTVVVSLQHSEKICLEDLRKVVMEKIIKEVIPARYLDDKTIFHVNPCGLFIIGGPQSDAGLTGRKIIVDTYGGWGAHGGGAFSGKDFTKVDRSAAYAARWVAKSLVKAGLCRRCLVQVSYAIGVAEPLSITVFDYGTSK----L---SQNELLD-IVNKNFDLRPGKIVKELNLRNP---I---YQQTSTYGHFGRE------GFTWEQPKTLILD--------------------------

>Insecta_Drosophila_hydei

MPQK--------TNGH--STSNANGLNGSNGNGN--SYDM-EDGQTFLFTSESVGEGHPDKMCDQISDAILDAHLRQDPNAKVACETVAKTGMILLCGEITSKAVVDYQKVVRETVQHIGYDDSSKGFDYKTCNVLLALDQQSPEIAAGVHV---NRLEEEIGAGDQGIMFGYATDETEECMPLTVVLAHKLNEKIAELRRSEVFWWARPDSKTQVTCEYLFNQGSAVPKRVHTIVVSMQHSDKIQLDTLRSEIMEKVVKVVIPAKYFDANTIVHINPCGLFVIGGPMGDAGLTGRKIIVDTYGGWGAHGGGAFSGKDFTKVDRSAAYAARWVAKSLVKAGLCKRCLVQVSYAIGLAEPLSITVFDYGTSH----K---SQKELLE-IIKRNFDLRPGMIVKDLNLRQP---I---YQRTSTYGHFGRD------GFSWEQAKHLEIN--------------------------

>Insecta_Aedes_aegypti

MPQTK------ATNGFA-T----N-------GHN--GYEM-EDGCSFLFTSESVGEGHPDKMCDQISDAILDAHLQQDPNAKVACETISKTGMILLCGEITSKAVIDYQKIVRETVKHIGYDDSSKGFDYKTCNVLLALDQQSPNIAAGVHV---NRAEEEVGAGDQGLMFGYATDETEECMPLTVVLAHRLNERIAELRRSGEFWWARPDSKTQVTAEYVFESGACIPQRVHTIVVSLQHSDKIGLEELRKNIMDKVIKEVIPAKYFDSNTIVHINPCGLFIIGGPQGDAGLTGRKIIVDTYGGWGAHGGGAFSGKDFTKVDRSAAYAARWVAKSLVKAGICRRCLVQVAYAIGLAEPLSITVFDYGTSK----Y---SQKELLN-IVSKNFDLRPGKIVKDLKLRTP---F---YQRTSTYGHFGRE------GFPWEEPKELVMN--------------------------

>Insecta_Bombus_terrestris

MPETA-----HHMNGY--A----N-------GHT--PPEL-QQDTSFLFTSESVGEGHPDKMCDQISDAILDAHLKQDPDAKVACETVTKTGMILLCGEITSKAVVDYQKIVRDTVKHIGYDDSSKGFDFKLCSVLLALDAQSPNIAAGVHE---NRSDEEVGAGDQGLMFGYATDETDECMPLTVVLAHKLNQKIAELRRSGELWWARPDSKTQVTCEYVMDRGACVPIRVHTVVVSLQHSEKIGLEELRKAVMEKVIKEVIPARYLDDRTIFHVNPCGLFIIGGPQSDAGLTGRKIIVDTYGGWGAHGGGAFSGKDFTKVDRSAAYAARWVAKSLVKAGLCRRCLVQVSYAIGVAEPLSITVFDYGTSK----L---SQHELLG-IVNKNFDLRPGKIVKELNLRNP---I---YQQTSTYGHFGRE------GFTWEQPKTLILD--------------------------

>Insecta_Trichogramma_pretiosum

MPENA-----HHMNGY--A----N-------GHT--PPEL-QQDTSFLFTSESVGEGHPDKMCDQISDAILDAHLRQDPDAKVACETVTKTGMVLLCGEITSKAVVDYQKIVRDTVNHIGYDDSSKGFDYKLCSILLALDAQSPNIAAGVHE---NRSDEEVGAGDQGLMFGYATDETDECMPLTVVLAHRLNQKIAELRRSGELWWARPDSKTQVTCEYKMDHGACVPIRVHTVVVSLQHSEKVSLEDLRQAIMDKVIKEVIPAKYLDERTVFHVNPCGLFIIGGPQSDAGLTGRKIIVDTYGGWGAHGGGAFSGKDFTKVDRSAAYAARWVAKSLVKAGLCRRCLVQVSYAIGVAEPLSITVFDYGTSK----L---SQNELLA-IVNKNFDLRPGKIVKELNLRNP---I---YQQTSTYGHFGRD------CFTWEQPKTLKLD--------------------------

>Insecta_Ostrinia_furnacalis

MPETS------KMNGYAKT----N-------GH---SFDM-EDGSVFLFTSESVGEGHPDKMCDQISDAILDAHLRQDPDAKVACETVTKTGMVLLCGEITSKANVDYQKVVRDTVKHIGYDDSSKGFDYKTCCVMLALAQQSPNIAAGVHE---NRNDEEVGAGDQGLMFGYATDETEECMPLTVVLAHRLNQKIAELRRNGEFWWARPDSKTQVTCEYNFVGGATVPQRVHTVVVSLQHSEKITLETLREEIKEKVIKEVIPAQYLDDKTVIHINPCGLFIIGGPQSDAGLTGRKIIVDTYGGWGAHGGGAFSGKDFTKVDRSAAYAARWVAKSLVKAGLCRRCMVQVSYAIGVAEPLSISVFDYGTSH----K---RQKELLA-IVKKNFDLRPGKIVKELNLRAP---I---YQKTSTYGHFGRE------GFPWENPKPLVVE--------------------------

>Insecta_Apis_cerana

MPETA-----HHMNGY--A----N-------GHT--PPEL-QQEGSFLFTSESVGEGHPDKMCDQISDAILDAHLKQDPNAKVACETVTKTGMVLLCGEITSKAVVDYQKIVRDTVKHIGYDDSSKGFDFKLCSVLLALDAQSPNIAAGVHE---NRSDEEVGAGDQGLMFGYATDETDECMPLTVVLAHKLNQKIAELRRSGELWWARPDSKTQVTCEYVMDHGACVPIRVHTVVVSLQHSEKICLEDLRKVVMEKIIKEVIPGRYLDDKTIFHVNPCGLFIIGGPQSDAGLTGRKIIVDTYGGWGAHGGGAFSGKDFTKVDRSAAYAARWVAKSLVKAGLCRRCLVQVSYAIGVAEPLSITVFDYGTSK----L---SQNELLD-IVNKNFDLRPGKIVKELNLRNP---I---YQQTSTYGHFGRE------GFTWEQPKTLILD--------------------------

>Insecta_Pieris_rapae

MPETS------KMNGFAKS----N-------GH---SYDM-EDGSLFLFTSESVGEGHPDKMCDQISDAILDAHLAQDPDAKVACETVTKTGMVLLCGEITSKAFVDYQKVVRETVKHIGYDDSSKGFDYKTCSVMLALDQQSPNIAAGVHE---NRNDEEVGAGDQGLMFGYATDETEECMPLTVVLAHRLNQKIAELRRNGEFWWARPDSKTQVTCEYIFAGGATVPQRVHTVVVSLQHSEKISLETLREEIRNKVINEVIPSQYLDERTVVHINPCGLFIIGGPQSDAGLTGRKIIVDTYGGWGAHGGGAFSGKDFTKVDRSAAYAARWVAKSLVKAGLCRRCMVQVAYAIGVAKPLSITVFDYGTSH----K---TQQELLS-IILKNFDLRPGKIVKDLNLKAP---I---YQRTSTYGHFGRE------GFPWETPKPLIVD--------------------------

>Insecta_Manduca_sexta

MPETS------KMNGYAKT----N-------GH---SYDM-EDGSVFLFTSESVGEGHPDKMCDQISDAILDAHLRQDPDAKVACETVTKTGMVLLCGEITSKANVDYQKVVRETVKHIGYDDSSKGFDWRTLNLLVAIEEQSPNIAEGVYQ---DRDEIDIGAGDQGLMFGYATDETEECMPLTVVLAHRLNQKIAELRRNGEFWWARPDSKTQVTCEYIFAGGATVPQRVHTVVVSLQHSEKITLDHLRDEIKEKVIKEVIPSQYLDNNTVIHINPCGLFIIGGPQSDAGLTGRKIIVDTYGGWGAHGGGAFSGKDFTKVDRSAAYAARWVAKSLVKAGLCRRCMVQVAYAIGVAEPLSITVFDYGTSH----K---TQQELLA-IVQKNFDLRPGKIVKELNLRAP---I---YQRTSTYGHFGRE------GFPWENPKPLVVE--------------------------

>Insecta_Trichoplusia_ni

MPETS------KMNGYAKT----N-------GH---SYDM-EDGSVFLFTSESVGEGHPDKMCDQISDAILDAHLRQDPDAKVACETVTKTGMVLLCGEITSKANVDYQRVVRETVKHIGYDDSSKGFDYKTCCVMLALDQQSPNIAAGVHE---NRNEDEVGAGDQGLMFGYATDETEECMPLTVVLAHRLNQKIAELRRNGEFWWARPDSKTQVTCEYVFAGGATVPQRVHTLVVSLQHSEKITLETLREEIKEKVIKEVIPAHYLDEKTVIHINPCGLFIIGGPQSDAGLTGRKIIVDTYGGWGAHGGGAFSGKDFTKVDRSAAYAARWVAKSLVKAGLCRRCMVQVAYAIGVAEPLSITVFDYGTSH----K---TQQQLLA-IVKKNFDLRPGKIVKELNLRAP---I---YQKTSTYGHFGRE------GFPWENPKPLAVE--------------------------

>Insecta_Helicoverpa_armigera

MPETS------KMNGYAKT----N-------GH---SYDM-EEGSVFLFTSESVGEGHPDKMCDQISDAILDAHLRQDPDAKVACETVTKTGMVLLCGEITSKANVDYQKVVRETVKHIGYDDSSKGFDYKTCCVMLALDQQSPNIAAGVHE---NRNDDEVGAGDQGLMFGYATDETEECMPLTVVLAHRLNQKIAELRRNGEFWWARPDSKTQVTCEYVFAGGATVPQRVHTLVVSLQHSEKITLETLREEIREKVIKEVIPAHYIDEKTVIHINPCGLFIIGGPQSDAGLTGRKIIVDTYGGWGAHGGGAFSGKDFTKVDRSAAYAARWVAKSLVKAGLCRRCMVQVAYAIGVAEPLSITVFDYGTSH----K---TQQQLLA-IVQKNFDLRPGKIVKELNLRAP---I---YQRTSTYGHFGRE------GFPWENPKPLVVE--------------------------

>Insecta_Camponotus_floridanus

MPETA-----HHMNGY--A----N-------GHA--PPEL-QQDTTFLFTSESVGEGHPDKMCDQISDAILDAHLKQDPDAKVACETVTKTGMILLCGEITSKAVVDYQKIVRDTVKHIGYDDSSKGFDWRTLNLLVAIEQQSSNIADGVHT---DRQEMDVGAGDQGLMFGYATDETDECMPLTVVLAHKLNQKIAELRRSGELWWARPDSKTQVTCEYIMDHGACVPIRVHTVVVSLQHSEKIGLDELRKAIMEKVIKDVIPPRYLDDRTIFHVNPCGLFIIGGPQSDAGLTGRKIIVDTYGGWGAHGGGAFSGKDFTKVDRSAAYAARWVAKSLVKADLCRRCLVQVSYAIGVAEPLSITVFDYGTST----R---SQNELLD-IVNKNFDLRPGKIVKELNLRNA---I---YQQTSTYGHFGRD------GFTWEQPKKLILD--------------------------

>Insecta_Galleria_mellonella

MPETA------KMNGYAKT----N-------GH---SFDM-EDGSVFLFTSESVGEGHPDKMCDQISDAILDAHLLQDPDAKVACETVTKTGMILLCGEITSKANVDYQKVVRETVKHIGYDDSSKGFDYKTCSVMLALDQQSPNIAAGVHE---NRNEEEVGAGDQGLMFGYATDETEECMPLTVVLAHRLNQKIAELRRNGEFWWARPDSKTQVTCEYVFAGGATVPQRVHTVVVSLQHSEKITLETLREEIREKVIKDVIPAQYLDDRTVVHINPCGLFIIGGPQSDAGLTGRKIIVDTYGGWGAHGGGAFSGKDFTKVDRSAAYAARWVAKSLVKAGLCRRCMVQVAYAIGVAEPLSITVFDYGTSH----K---TQQELLA-IVLKNFDLRPGKIVKELNLRAP---I---YQKTSTYGHFGRE------GFPWENPKPLVVE--------------------------

>Insecta_Sipha_flava

MPEET---KYAYTNGF--ANGHAN-------GGL--PPES-PDGEMFLFTSESVGEGHPDKMCDQISDAILDAHLQQDPDAKVACETVTKTGMVLLCGEITSKANVDYQKVVRDTVKHIGYDDSSKGFDYKTCNVLVTVDEQSSNIADGVHL---NRIEDAIGAGDQGLMFGYATDETEECMPLTVVLAHKLNEKVAELRRNGVLWWARPDTKTQVTCEYCLVGGACIPQRVHTVVISVQHSEKITLENLRQEVMSKVVKTVIPSQYLDERTVFHINPCGNFVMGGPQCDAGLTGRKIIVDTYGGWGAHGGGAFSGKDFTKVDRSAAYAARWVAKSLVKSGLCKRCLVQVSYAIGVAEPISITLFHYGTST----K---SQKELLK-IVNSNFDLRPGKIVRDLNLRNP---I---YQKTSTYGHFGRD------IFPWEKPKILVD---------------------------

>Insecta_Photinus_pyralis

MPQNN------LTNGY--A----N-------GHI--EYEM-ENGSNFLFTSESVGEGHPDKMCDQISDAILDAHLQQDPNAKVACETVTKTGMILLCGEITSKAVVDYQKVVRETVEHIGYDDSSKGFDYKTCNVLLALDQQSSNIAAGVHE---NRPEEELGAGDQGLMFGYATDETEECMPLTVVLAHKLNHRIAELRRSGEFWWARPDSKTQVTCEYNFDHGACIPVRVHTVVVSLQHSDKITLEELRTSVYNKVIKAVIPARYLDDQTTVHINPCGLFIIGGPQSDAGLTGRKIIVDTYGGWGAHGGGAFSGKDFTKVDRSGAYAARWVAKSLVKAGLCRRCLVQVAYAIGVSEPLSITVFDYGTSK----L---TQKELLA-VVNNNFDLRPGRIVKDLNLRQP---I---YQKTSIYGHFGRD------GFAWEQPKVLRLN--------------------------

>Insecta_Myzus_persicae

MPEEK---KYAHTNGF--ANGHGN-------GGL--PPES-PDGEMFLFTSESVGEGHPDKMCDQISDAILDAHLEQDPDAKVACETVTKTGMVLLCGEITSKANVDYQRVVRDTIKHIGYDDSSKGFDYKTCSVMLAIDQQSPNIAAGVHI---NRTAEEVGAGDQGLMFGYATDETEECMPLTVVLAHKLNEKVAELRRNGVLWWARPDTKTQVTCEYCLVGGACIPQRVHTVVISVQHSEKISLEDLRQEVMSKVVKTVIPSQYLDERTVFHINPCGQFVMGGPQCDAGLTGRKIIVDTYGGWGAHGGGAFSGKDFTKVDRSAAYAARWVAKSLVKSGLCKRCLVQVSYAIGVAEPISITLFHYGTSI----K---SQKELLK-IVNNNFDLRPGRIVRDLNLRNP---I---YQKTSTYGHFGRD------IFPWEKPKVLVE---------------------------

>Insecta_Halyomorpha_halys

MPETS-----PYMNGF--Q----N-------GHT--PPEF-PNGEVFLFTSESVGEGHPDKMCDQISDAILDAHLSQDPDAKVACETVTKTGLILLCGEITSKAVVDYQKVVRETIKHIGYDDSSKGFDYRTCNVLVAIEQQSPEIAGGVYH---NRDDLDIGAGDQGLMFGYATDETEECMPLTVVLAHKLNERVAELRRNGVFWWARPDTKAQVTCEYKFIGGACVPLRVHTVVISVQHSEKVSLSELRSAVKKDVIHQVIPAKYLDSDTIVHINPCGEFVLGGPQCDAGLTGRKIIVDTYGGWGAHGGGAFSGKDFTKVDRSAAYAARWVAKSLVKAALCKRCLVQVSYAIGVAEPISITLFDYGTST----K---TQKELLT-IVKKNFDLRPGKIVKDLNLRNP---I---YQKTSTYGHFGRE------IFPWEQPKELIE---------------------------

>Insecta_Diabrotica_virgifera_virgifera

MPETT------STNGY--S----N-------GHS--GYEM-ADGAEFLFTSESVGEGHPDKMCDQISDAILDAHLEQDPNAKVACETVAKTGMILLAGEITSKAVVDYQKVVRQTVKHIGYDDSSKGFDWRTLNLLVALEEQSPNIAEGVHG---NRDELEIGAGDQGLMFGYATDETEECMPLTVVLAHRLNHKIADLRRSGEFWWARPDSKTQVTAEYTFQRGACIPQRVHTVVVSLQHSDKISLEELRTEIKEKVIKEVIPAKYLDDQTVIHINPCGLFIIGGPQSDAGLTGRKIIVDTYGGWGAHGGGAFSGKDFTKVDRSAAYAARWVAKSLVKAGLCRRCLVQVSYAIGLAEPLSITVFDYGTSK----L---TQKELLD-IVNKNFDLRPGKIVKELNLRQP---I---YQKTSTYGHFGRD------GFTWEQPKTLQLN--------------------------

>Insecta_Hyposmocoma_kahamanoa

MPETS------KMNGYAKT----N-------GH---SYDM-EDGSVFLFTSESVGEGHPDKMCDQISDAILDAHLRQDPDAKVACETVTKTGMILLCGEITSKANVDYQKVVRETVKHIGYDDSSKGFDWRTLNLLVALEQQSPNIADGVHT---SRAELEIGAGDQGLMFGYATDETEECMPLTVALAHKLNQKIAELRRNGEFWWARPDSKTQVTCEYTFAGGATVPQRVHTVVVSLQHSEKITLDTLRDEIREKVIKEVIPPQYLDEKTVVHINPCGLFIIGGPQSDAGLTGRKIIVDTYGGWGAHGGGAFSGKDFTKVDRSAAYAARWVAKSLVKAGLCRRCMVQVAYAIGVAEPLSITVFDYGTSH----M---TQQQLLA-IVQKNFDLRPGKIVKELNLRAP---I---YQRTSTYGHFGRD------GFPWENPKPLVVE--------------------------

>Insecta_Leptinotarsa_decemlineata

MPETT------VTNGF--A----N-------GHS--GYET-ADGQEFLFTSESVGEGHPDKMCDQISDAILDAHLQQDPYAKVACETVTKTGMILVCGEISSKAVVDYQKVVRDTVKHIGYDDSSKGFDHKTCNVLLALDQQSPDIAAGVHI---NKPEEEVGAGDQGLMFGYATDETEESMPLTVVLAHRLNHKIADLRRSGEFWWARPDTKTQVTAEYVFEQGACIPKRVHTVVVSVQHSEKISLEDLRNAVTEQVIKEVIPSKYLDDKTVIHINPCGLFIIGGPQSDAGLTGRKIIVDTYGGWGAHGGGAFSGKDFTKVDRSAAYAARWVAKSLVKAGLCRRCLVQVSYAIGVAEPLSISVFDYGTSK----K---TQKELLD-IVKNNFDLRPGKIVKDLDLRQP---I---YQRTSTYGHFGRE------GFSWEQPKPLLLN--------------------------

>Insecta_Onthophagus_taurus

MPNTA------LTNGY--T----N-------GHM--PFDM-EDGSNFLFTSESVGEGHPDKMCDQISDAILDAHLEQDPDAKVACETVTKTGMVLLCGEITSKANVDYQKIVRQTVAHIGYDDSSKGFDYKTCSVMIALDQQSPNIAAGVHE---NRNEEEVGAGDQGLMFGYATDETEECMPLTVVLAHRLNQKIADLRREGTFWWARPDSKTQVTCEYKFEHGACIPQRVHTVVVSLQHSEKISLDELRNEVLHKVIKEVIPTKYLNGQTIVHINPCGLFIIGGPQSDAGLTGRKIIVDTYGGWGAHGGGAFSGKDFTKVDRSAAYAARWVAKSLVKAGLCRRCLVQVAYAIGVAEPLSITVFDYGTSK----L---SQGELLS-VIRNNFDLRPGKIVKELNLRQP---I---YQKTSTYGHFGRD------GFAWEQPKVLRLN--------------------------

>Insecta_Vanessa_tameamea

MPETS------KMNGYAKT----N-------GH---SYDM-EDGSVFLFTSESVGEGHPDKMCDQISDAILDAHLKQDPDAKVACETVTKTGMILLCGEITSKANVDYQKVVRETVKHIGYDDSSKGFDWRTLNLLVAIEEQSPNIADGVYR---DRDEIDIGAGDQGLMFGYATDETEECMPLTVVLAHRLNQKIAELRRNGEFWWARPDSKTQVTCEYVFAGGATVPQRVHTVVVSLQHSEKIAVETLREEIKNKVINEVIPAQYLDERTVIHINPSGLFIIGGPQSDAGLTGRKIIVDTYGGWGAHGGGAFSGKDFTKVDRSAAYAARWVAKSLVRAGLCRRCMVQVAYAIGVAEPLSITVFDYGTSH----K---TQQELLA-IVQKNFDLRPGKIVKELNLRAP---I---YQKTSTYGHFGRE------GFPWESPKPLVVD--------------------------

>Insecta_Scaptodrosophila_lebanonensis

MPQK--------TNGH--VVA--NGFNGS--NGN--SYDM-EDGQTFLFTSESVGEGHPDKMCDQISDAILDAHLRQDPNAKVACETVAKTGMILLCGEITSKAVVDYQKVVRETVQHIGYDDSSKGFDFKTCNVLLALDQQSPEIAAGVHV---NRAEEEIGAGDQGIMFGYATDETEECMPLTVVLAHKLNEKIAELRRSGVFWWARPDSKTQVTCEYLFNQGSAVPKRVHTIVVSMQHSEKITLDTLRTEVMEKVVKVVIPAKYFDSNTIVHINPCGLFVIGGPMGDAGLTGRKIIVDTYGGWGAHGGGAFSGKDFTKVDRSAAYAARWVAKSLVKAGLCKRCLVQVSYAIGLAEPLSITVFDYGTSH----K---TQKELLD-IIKRNFDLRPGMIVKDLNLRQP---I---YQRTSTYGHFGRD------GFSWEQAKPLENN--------------------------

>Insecta_Agrilus_planipennis

MPETV------HVNGF--I----N-------GHN--PYDM-EDGSNFLFTSESVGEGHPDKMCDQISDAILDAHLEQDPNAKVACETVTKTGMILLCGEITSKAVVDYQKVVRETIKHIGYDDSSKGFDWRTLNLLIAIEEQSPNIANGVYE---NKEERDIGAGDQGLMFGYATDETEECMPLTVVLAHKLNSRIAELRRSGEFWWARPDSKTQVTCEYNFSRGACVPLRVHTVVVSLQHSDKISLEDLRTEISNKVISQVIPERYLDDRTVVHINPCGLFIIGGPQSDAGLTGRKIIVDTYGGWGAHGGGAFSGKDFTKVDRSAAYAARWVAKSLVKAGLCRRCLVQVSYAIGLAEPLSITVFDYGTSK----L---SQKKLLE-IVRKNFDLRPGKIVKELNLKQP---I---YQKTSVYGHFGRD------GFTWEQPKQLRID--------------------------

>Insecta_Drosophila_navojoa

MPQK--------TNGH--STATVNGLNGSNGNGN--SYDM-EDGQTFLFTSESVGEGHPDKMCDQISDAILDAHLRQDPNAKVACETVAKTGMILLCGEITSKAVVDYQKVVRETVQHIGYDDSSKGFDWKTLNLLVAIEQQSPDIANGVHI---NRDEEDVGAGDQGIMFGYATDETEECMPLTVVLAHKLNEKIAELRRSEVFWWARPDSKTQVTCEYLFNQGSAVPKRVHTVVVSMQHSDKISLDTLRSEVMEKVVKVVIPAKYFDANTIVHINPCGLFVIGGPMGDAGLTGRKIIVDTYGGWGAHGGGAFSGKDFTKVDRSAAYAARWVAKSLVKAGLCKRCLVQVSYAIGLAEPLSITVFDYGTSH----K---SQKELLE-IIKRNFDLRPGMIVKDLNLRQP---I---YQRTSTYGHFGRD------GFSWEQAKHLEIN--------------------------

>Insecta_Rhopalosiphum_maidis

MPEEK---KYAHTNGF--ANGHGN-------GGL--PPES-PDGEMFLFTSESVGEGHPDKMCDQISDAILDAHLEQDPDAKVACETVTKTGMVLLCGEITSKANVDYQRVVRDTIKHIGYDDSSKGFDYKTCNVLVTVDEQSSNIADGVHV---NKIEDAIGAGDQGLMFGYATDETEECMPLTVVLAHKLNEKVAELRRNGVLWWARPDTKTQVTCEYCLVGGACIPQRVHTVVISVQHSEKISLEDLRQEVMSKVVKTVIPSQYLDDRTVFHINPCGQFVMGGPQCDAGLTGRKIIVDTYGGWGAHGGGAFSGKDFTKVDRSAAYAARWVAKSLVKSGLCKRCLVQVSYAIGVAEPISITLFHYGTST----K---TQKELLK-IVNSNFDLRPGRIVRDLNLRNP---I---YQKTSTYGHFGRD------IFPWEKPKILVE---------------------------

>Insecta_Acyrthosiphon_pisum

MPEEK---KYAHTNGF--ANGHGN-------GGL--PPES-PDGEMFLFTSESVGEGHPDKMCDQISDAILDAHLEQDPDAKVACETVTKTGMVLLCGEITSKANVDYQRVVRDTIKHIGYDDSSKGFDYKTCNVLVSVDEQSSNIADGVHV---NKIEDAIGAGDQGLMFGYATDETEECMPLTVVLAHKLNEKVAELRRNGVLWWARPDTKTQVTCEYCLVGGACIPQRVHTVVISVQHSEKISLEDLRQEVMSKVVKTVIPSQYLDDRTVFHINPCGQFVMGGPQCDAGLTGRKIIVDTYGGWGAHGGGAFSGKDFTKVDRSAAYAARWVAKSLVKSGLCKRCLVQVSYAIGVAEPISITLFHYGTST----K---TQKELLK-IVNSNFDLRPGRIVRDLNLRNP---I---YQKTSTYGHFGRD------IFPWEKPKVLVE---------------------------

>Insecta_Melanaphis_sacchari

MPEEK---KYAHTNGF--ANGHGN-------SGL--PPES-PDGEMFLFTSESVGEGHPDKMCDQISDAILDAHLEQDPDAKVACETVTKTGMVLLCGEITSKANVDYQRVVRDTIKHIGYDDSSKGFDYKTCNVLVTVDEQSSNIADGVHV---NKIEDAIGAGDQGLMFGYATDETEECMPLTVVLAHKLNEKVAELRRNGVLWWARPDTKTQVTCEYCLVGGACIPQRVHTVVISVQHSEKISLEDLRQEVMSKVVKTVIPSQYLDDRTVFHINPCGQFVMGGPQCDAGLTGRKIIVDTYGGWGAHGGGAFSGKDFTKVDRSAAYAARWVAKSLVKSGLCKRCLVQVSYAIGVAEPISITLFHYGTST----K---TQKELLK-IVNSNFDLRPGRIVRDLNLRNP---I---YQKTSTYGHFGRD------IFPWEKPKILVE---------------------------

>Insecta_Diachasma_alloeum

MPETA---SSHRMNGY--A----N-------GHT--PPEL-QQATTFLFTSESVGEGHPDKMCDQISDAILDAHLEQDPDAKVACETVTKTGMILLCGEITSKAVVDYQKIVRDTVNHIGYDDSSKGFDWRTLNLLVAIEQQSPNIAGGVHV---DREEKDLGAGDQGLMFGYATDETDECMPLTVVLAHKLNQKIAELRRNGELWWARPDSKTQVTCEYMMDQGACVPVRVHTVVVSLQHSEKITLDELRRAVMDKVIKEVIPAKYLDEKTIFHVNPCGLFIIGGPQSDAGLTGRKIIVDTYGGWGAHGGGAFSGKDFTKVDRSAAYAARWVAKSLVKAGLCRRCLVQVSYAIGVAEPLSITVFDYGTSK----L---PQEKLLG-IVNKNFDLRPGKIVKELNLRRP---I---YQQTSTYGHFGRD------GFTWEQPKQLILD--------------------------

>Insecta_Cephus_cinctus

MPETA-----HHMNGY--A----N-------GHT--APEL-QQGTSFLFTSESVGEGHPDKMCDQISDAILDAHLQQDPNAKVACETVTKTGMVLLCGEITSKAVVDYQKIVRDTVKHIGYDDSSKGFDWRTLNLLVAIEQQSPNIADGVHV---NREDMDVGAGDQGLMFGYATDETEECMPLTVVLAHRLNQKIAELRRSGELWWARPDSKTQVTCEYIMDHGACVPVRVHTVVVSLQHSEKVTLEDLRKAVMEKVIKEVIPGKYLDERTIFHVNPCGLFIIGGPQSDAGLTGRKIIVDTYGGWGAHGGGAFSGKDFTKVDRSAAYAARWVAKSLVKADLCRRCLVQVSYAIGVAEPLSITVFDYGTSK----L---SQNELLE-IVRKNFDLRPGKIVKELKLRNP---M---YQQTSTYGHFGRD------GFAWEQPKSLILD--------------------------

>Nematoda_Strongyloides_ratti

MVTDAKRLKMSNETNNKAG----------------------NKEYKFLFTSESVSEGHPDKMCDLISDAVLDAHLAQDPNAKVACETVTKTGMVLLAGEITSTAIVDYQTVVRNVIKDIGFDDSSKGFDYKTCNLLVALEQQAPEIAQGVHV---NRSEEDVGAGDQGLMFGYATDETEEAMPLSLLLAHKLVAYLHKLRREGLLPWARPDSKSQVTVEYEFENGACIPVRVHTIVLSCQHDPEIDLDFLRSEITEKVIKHVIPPKLMDDNTILYLNPCGSFAIGGPQSDAGLTGRKIIVDTYGGWGAHGGGAFSGKDPTKVDRSAAYGARWVAKSLVKAGVCRRCLVQVSYAIGIAHPLSITVMDYGTSP----L---NESELLT-IVNDNFDLRPGMIIKTLGLKRP---I---YKATAANGHFGHI------SFPWEEVKELTIRDEFLKKLNAVKNSKTSDA--------

>Nematoda_Trichinell_nativa

MEPPVEAEINTEDHYVAYNSRYSLECGQ-------------DDVDTFLFTSESVGIGHPDKLCDQVSDAVLDAHLTVDPNAKVACETVTKTGMIMLLGEISSTATVDYHNIVRRVVKKIGFDDSSKGFDYKTCNVLVALEQQSPEISAGVHL---NRDDEHLGAGDQGLMFGYATDETEECMPLSLALAHKMNNLYRTLRERKILWWARPDSKSQVTVEYKFDRGACIPQRIHSVIMSCQHHQDISIEELRSQVMEFIIKPVIPQKYLDDRTIYHLNPCGSFVLGGPLGDAGLTGRKIIVDTYGGWGAHGGGAFSGKDASKVDRSAAYAARWIAKSLVKAGLCKRCLVQISYAIGIAEPVSVTIFSYGTSS----L---SERDLLE-VVDKNFDLRPGKIIKELNLRRP---I---YEKTAENGHFGHD------EFPWEKPKKLILPPHLVEKVQHSENIITVNEKCLNGMNA

>Nematoda_Trichuris_trichiura

MVLPN-------SHYVAYNDHYSAESGQ-------------DDIGNFLFTSESVGVGNADKLCDQVSDAILDAHLEQDPNAKVAIETVAKTGMVMVMGEITSKATVDYQNLVRRVVRKVGFDNSSKGFDYSTCNVMVVIGEQSAEISAGVHL---NRDDEHIGAGDQGLMFGYATDETEECMPLTLSLAHRMNARYRTLRENGTLPWARPDSKCQITMEYNFNCGACIPRHLQSVIFSCQHSPEVTLEQVEKQVIELVIRPVVPEKYINVTSIIKVNPDGAYTHGGPYAGVGLTGRKIIVDTYGGWGAHGGGAFSGKDPTKVDRSAAYACRWIAKSLVKSGLCRRCMVQVAYAIGVAEPVSLTIFSYGTSI----L---GEQDLLE-IVKANFDLRPGMIIRSLNLRRP---I---YEKTAENGHFGHD------DFPWEKPKELVIPPKVLKILKSVQQF-------------

>Nematoda_Brugia_malayi

MAHDGFGMHRNSSEL--------------------------FRNQRFLFTSESVSEGHPDKMCDMISDAVLDAHLAQDPNAKVACETVAKTDMLMLCGEITSSANVDYYQIVRDTVQQIGFDDSSKGFDYKTCNVIIALEQQAPEIAAGVHL---NRKMEEIGAGDQGLMFGYATDETEERMPLSLLLAHKLLARLHELRRNGTLEWALPDSKSQVTVEYKFEYGACIPVRVHTVVISAQHKPTASLEKIRGDLIKHVINEVVPQHFMDTATKFYLNPCGNFTVGGPRSDAGLTGRKIIVDTYGGWGAHGGGAFSGKDPSKVDRSAAYGARWVAKSLVDAGICKRCLIQVSYAIGIAEPLSITVVDYGTSN----L---TEDELLT-IVNDNFDLRPGVIIRELDLLKP---I---YKETARNGHFGKS------LFAWEKSKKLAIRPEFMNKLRSSELSNGDIKRNSFNVA-

>Rotifera_Brachionus_plicatilis

MENVP----------------------------K--KMKP-DQTDTFLFTSESVGEGNPDKICDQISDAILDAHLEKDPDSRVACETFCKTGMILVGGEVSSRAHIDYQKVIRDTIKHIGYDDDRKGFDYRICSVLLSIDEQSPDIAQCLHL---DQKAENLGAGDQGLMFGYATDETEECMPLTIMLAHKLNKKIADLRRDGTLPWSRPDSKTQVTVEYRISDGSCLPVRVHTIVISAQHSPDVTLEQVRRDLMEKVIKPSIPSGYLDERTIYHLNPIGRFVIGGPQCDTGLTGRKIIVDTYGGWGAHGGGAFSGKDYTKVDRSGAYAARWVAKSLVKAGLCRRVLVQLSYAIGVAHPLSITIFSYGSST----K---SELELLE-IVKNNFDLRPGKIIEQLDLKKP---I---YKKTSCYGHFGRD------EFTWEKAKKLVY---------------------------

>Platyhelminthes_Schistosoma_bovis

MPSFT------------------N-------G------GN-AESDTFLFTSESVGEGHPDKVCDQISDAILDEFLRQDPEAKVACESFVKSGLILVGGEISSNAVVDIQKTVRDVVKHIGYDDTHKGLDYKNCNVLTVIEQQSADIAKGVHE---GRKIEDIGAGDQGLMFGYATDETEECMPLTVTLSHALNKRIATCRRNGILPWAGPDSKTQVTVEYLNDHGACRPLRVHTVVISTQHDEHVTLEKVRADLMEYVIKEVIPTNLLDEKTKYHLNPAGRFVIGGPQSDAGLTGRKIIVDTYGGWGAHGGGAFSGKDYSKVDRSAAYAARWVAKSLVKSGLCRRVLVQLSYAIGIAEPLAINVNSYGTAK----I---SDKKLLD-IIVNNFDLRPGVIVKDLDLRTP---R---YLQTAVYGHFGRP------EFPWEQCKKLTF---------------------------

>Platyhelminthes_Fasciola_gigantica

MPGLT------------------N-------G------DI-DDSDTFLYTSESVGEGHADKLCDQVSDAILDEFLRHDPEAKVACETFTKTGMILVGGEISSTAAVDLPSIIRKVVRDIGYDDPRKGFDYKTCNILTAIEQQSPDIADGVHK---GRKAEEIGAGDQGLMFGYATDETEECMPLTVVFSRMLNKTSCRIRRDGTLPWAGPDSKSQVTVEYRHDGGACRPLRVHTIVVSAQHDANVTLEKVRSDLMEHVIRAVIPENLLDDQTIYHLNPAGRFVVGGPHGDAGLTGRKIIVDTYGGWGAHGGGAFSGKDYSKVDRSAAYAARWVAKSLVKAGLCRRVLVQLSYAIGISEPLSIYLDSYGTAK----V---SDRELLD-IVKRNFDLRPGVIVNELDLRNP---R---YLQTAVYGHFGRP------EFPWEQCKKLQV---------------------------

>Cnidaria_Exaiptasia_pallida

MSHIHSNNDPFALNGQ-PP----E----KKFKME--N-PD-TQNNTFLFTSESVGEGHPDKMCDQISDAILDAHLKEDPNAKVACETVAKTGMILLCGEITSNAQIDYQQVVRDCIKDIGYDDSSKGFDYKTCNVLVALEQQSADIAQGVHL---GRHEDDVGAGDQGLMFGYATDETEELMPLTVVLAHGMNRKLADCRRDGTMPWVRPDSKTQVTVEYKFENGSAIPLRVHTIVISVQHDPSITVEKLRKELMEKIVKTVVPAKYLDEETVFHLQPSGLFVIGGPQGDAGLTGRKIIVDTYGGWGAHGGGAFSGKDYTKVDRSAAYAARWIAKSLVAAKLCRRVLVQISYAIGIAQPLSVTVFSYGTST----K---SEDELLQ-IVRENFDLRPGRIVKDLDLKKP---V---YQRSCVYGHF-KS------GFTWEVPKELSS---------------------------

>Choanoflagellata_Salpingoeca_rosetta

MSS---------------------------------------ENDTFLFTSESVGEGHPDKICDQVSDAVLDACLKQDPESRVACETASKTGMIMVFGEITSSAHIDYQKVIRDTIKEIGYDSDEKGFDYKTCNVLVAIEQQSADISQGVDKDGRGKSEENIGAGDQGIMFGYATDETPECMPLTCQLAHALNRRLAECRRDGELPWARPDTKTQVTIEYKLDNGACVPLRVHTIVISTQHADFVSNEQIHADLMEKVIKAVVPSNLLDDKTQYHLNPSGRFVIGGPQGDAGLTGRKIIVDTYGGWGAHGGGAFSGKDYSKVDRSAAYAARWVAKSLVKAGLVRRCLVQVSYAIGIAEPISLHVEDYGTGK----V---SPNELLK-IIRKNFDLRPGVIVRDLDMKKP---I---FSKTAKYGHFGRE------EFPWEQPKKLEL---------------------------

>Ichthyospora_Capsaspora_owczarzaki

MAT-----------------------------------------ERFLFTSESVGEGHPDKICDQVSDAILDACLAQDPDSKVGCETASKTGMIMAFGEITSKAVLDYQKIIRGVIEKIGYDSSDKGFDYKTCNVLVAVEQQSPDIAQGVHI---NRALEDIGAGDQGIMFGYATDETPEFMPLTLSLAHKLNARLSECRRNSQMPYLRPDTKTQVTVEYKHADGTLEPLRVHTIVISTQHAEDVSNDTIRADLHEKVIKSVVPAKYLDDKTIYHLQPSGRFVIGGPQGDAGLTGRKIIVDSYGGWGAHGGGAFSGKDFSKVDRSAAYAARWIAKSMVAAGLAKRLLVQVSYAIGVAKPLSVFVDAYGTSK----K---SHAELVE-IINKNFDLRPGFIVRDLDLKKP---I---YQNTACYGHFGRP------EFTWEQPKKLDF---------------------------

>Fungi_Smittium_mucronatum

MS------------------------------------------ETFLFTSESVGEGHPDKICDQVSDAILDACLKQDPMSKVACETAAKTGMIMVFGEITTKAVLDYQLIIRNVIKDIGYDSSEKGFDYKTCNVLVAIEQQSPDIYQGLFQN--GLDVENIGAGDQGIMFGYATDETPELMPLTIVLAHKLNMKLAELRRDGTLPYLRPDTKTQVTVEYKNDNGAMVPLRVHTVVISTQHSPDVDNDTLRKDLREKVVNAVIPAKYLSEDTILHLQPSGRFIIGGPQGDAGLTGRKIIVDTYGGWGAHGGGAFSGKDFSKVDRSAAYSSRWIAKSLVKAGIARRCLVQVSYAIGVAEPLSIHVDTYGTST----M---SDSEILE-IIKKNFDLRPGVIVKSLDLVNP---I---YQKTACYGHFGRE------EFTWEAPRSLEL---------------------------

>Fungi_Hesseltinella_vesiculosa

MS-----------------------------------------DNTFLFTSESVGEGHPDKICDQVSDAILDACLAQDPMSKVACETAAKTGMIMVFGEITTKAVLDYQKIIRNAIKQIGYDSSEKGFDYKTCNVLVAIEQQSPDISQGLVQK--SFNIEDIGAGDQGIMFGYATDETPEMMPLTVVLSHKLNRRMADLRRDGTLGWLRPDSKTQVTIEYKNDNGAMIPLRVDTIVISVQHAEEISTEDLRKALMEHVIKPVVPAHLLNDKTIYHLQPSGKFIIGGPQGDAGLTGRKIIVDTYGGWGAHGGGAFSGKDWSKVDRSGAYAARWVAKSLINAKLCRRCLVQFSYAIGVAEPLSVFVDTYGTGV----R---PESEILE-IVKKNFDLRPGVIVKELDLFKP---V---YQQTAAYGHFGRE------EFTWDQPKELTF---------------------------

>Fungi_Thamnocephalis_sphaerospora

MS------------------------------------------NTFLFTSESVGEGHPDKICDQVSDAILDACLAEDPRSKVACETAAKTGMIMVFGEITTSAKLDYQRVIRDAVKAIGYDDSEKGFDYKTCNVLVAIEQQSPEIAQGLVQR--GDAIEDTGAGDQGIMFGYATDETPEGMPFTIMMAHKLNMKMSELRRSGELGWLRPDSKTQVTAEYRNEDGAMVPVRVHTIVISTQHAPEVTNEEIRKSLLEKVVRAVIPAKFLDDKTIFHLQPSGKFIIGGPQGDAGLTGRKIIVDTYGGWGAHGGGAFSGKDWSKVDRSAAYAARWIAKSLVRAGLVRRCLVQLSYAIGVAEPLSIFIDTYGTGR----R---SDDELLK-IINDNFDLRPGVIVRELKLTNP---V---YRRTACYGHFGRE------EFTWEQPKQLKF---------------------------

>Fungi_Batrachochytrium_salamandrivorans

MT---------------------------------------VEGEVFLFTSESVGEGHPDKICDQVSDAILDACLAEDKFSRVACETAAKTGMIMVLGEITTSAKLDYQKVIRETIKEIGYDDSEKGFDYKTCNVLVAIEQQSPDIAGGLVQD--GTDIEKIGAGDQGIMFGYATDETPELMPLTIMLAHKLNIKMTELRNNGVCGWLRPDSKTQVTVEYKNIDGVPVPQRVHTIVISTQHAPEIALADLQKELLEKVIKTVIPAEYLDDKTVYHLQPSGRFIIGGPQGDAGVTGRKIIVDTYGGWGAHGGGAFSGKDWSKVDRSAAYTARWVAKSLVAAGLARRVLVQFSYAIGVAEPTSVYVDTYGTGK----K---SNAELLT-IIKQNFDLRPGAIVKELDLWRP---I---YRATAKYGHFGRS------EFSWEQPKTLKF---------------------------

>Fungi_Meira_miltonrushii

MVSAQN------------P----Q-------TNG--SRAP-LPDGQFLFTSESVGEGHPDKICDQVSDAILDACLEQDPNSKVACETATKTGMIMVFGEITTKAQLDYQKVIRNKIKEIGYDSSEKGFDYNTCNVLVAIEQQSPDIAQGLDH----GDLEAHGAGDQGIMFGYATDETPEMMPATIVLAHKLNTAMSAARRSGALAWLRPDTKTQVTIQYKKDGGAMIPVRVDTVVVSAQHSDDISTEDLRKEILEKIVKQVIPANLLDDHTVYHIQPSGRFVIGGPQGDAGLTGRKIIVDSYGGWGAHGGGAFSGKDFSKVDRSAAYTARWVAKSLVASGLARRCLVQLSYAIGVAEPLSVFVDSYGTSK----K---TDAELVN-IVRSNFNLKPGVIVRDLGLQKP---I---YAKTAYGGHFGRS------EFSWEQPKQLQH---------------------------

>Fungi_Catenaria_anguillulae

MS-----------------------------------------NDTFLFTSESVGEGHPDKIADQVSDAILDACLEQDKLSKVACETATKTGMIMVLGEITTKANLDYQKIIRSTIKKIGYDDSDKGFDYKTCNVLVAIEQQSPDIAQGLVQE--GFDVEKIGAGDQGIMFGYATEETPELMPLTLVLAHKLNARLAECRRNGEMSYLRPDTKTQVTVEYKLDNGQPIPLRVDAIVISTQHSEDVTNDKLRADLKSKVVAHVVPEKFLDANTKYHLQPSGRFVIGGPQGDAGVTGRKIIVDSYGGWGAHGGGAFSGKDWSKVDRSAAYAARWVAKSLVAAGLARRALIQVSYAIGVAEPVSIHVETYGTGK----L---SDAELLR-IIKNNFDLRPGKIVQELDLWRP---I---YSATACYGHFGRS------EFPWEQPKKLNLE--------------------------

>Fungi_SAM1_Saccharomyces_cerevisiae

MA------------------------------------------GTFLFTSESVGEGHPDKICDQVSDAILDACLAEDPHSKVACETAAKTGMIMVFGEITTKAQLDYQKIVRDTIKKIGYDDSAKGFDYKTCNVLVAIEQQSPDIAQGVHE---EKDLEDIGAGDQGIMFGYATDETPEGLPLTILLAHKLNMAMADARRDGSLAWLRPDTKTQVTVEYKDDHGRWVPQRIDTVVVSAQHADEITTEDLRAQLKSEIIEKVIPRDMLDENTKYFIQPSGRFVIGGPQGDAGLTGRKIIVDAYGGASSVGGGAFSGKDYSKVDRSAAYAARWVAKSLVAAGLCKRVQVQFSYAIGIAEPLSLHVDTYGTAT----K---SDEEIID-IISKNFDLRPGVLVKELDLARP---I---YLPTASYGHFTNQ------EYPWEKPKTLKF---------------------------

>Fungi_SAM2_Saccharomyces_cerevisiae

MS----------------------------------------KSKTFLFTSESVGEGHPDKICDQVSDAILDACLEQDPFSKVACETAAKTGMIMVFGEITTKARLDYQQIVRDTIKKIGYDDSAKGFDYKTCNVLVAIEQQSPDIAQGLHY---EKSLEDLGAGDQGIMFGYATDETPEGLPLTILLAHKLNMAMADARRDGSLPWLRPDTKTQVTVEYEDDNGRWVPKRIDTVVISAQHADEISTADLRTQLQKDIVEKVIPKDMLDENTKYFIQPSGRFVIGGPQGDAGLTGRKIIVDAYGGASSVGGGAFSGKDYSKVDRSAAYAARWVAKSLVAAGLCKRVQVQFSYAIGIAEPLSLHVDTYGTAT----K---SDDEIIE-IIKKNFDLRPGVLVKELDLARP---I---YLPTASYGHFTNQ------EYSWEKPKKLEF---------------------------

>Fungi_Cylindrobasidium_torrendii

MSNG----------------------------HA--VRAA-LEPGHFLFTSESVGEGHPDKICDQVSDAILDACLAQDPWSKVGCETASKTGMIMVFGEISTQAKLDYQKIIRDTIKEIGYDDSEKGFDYKTCNILVAIEQQSPDIAQGLDH----GDLENHGAGDQGIMFGYATDETEECMPLTVVLSHKLNAAMAAARRSGLLPWLRPDTKTQVTVEYKKDGGATIPLRVDTIVISAQHSEDVTLAQLQKDLLEKVIKQVIPANLLDDKTVYHINPSGRFVIGGPQGDAGLTGRKIIVDSYGGWGAHGGGAFSGKDWSKVDRSAAYTARWIAKSLVTSGFARRILVQLSYAIGVAEPLSVYVDSYGTGK----I---DDDAIVQ-LIRKNWDLRPGVMVRELTLQEP---K---YRRTACYGHFGNP------DYTWEQPKKIVA---------------------------

>Fungi_Glomus_cerebriforme

MS----------------------------------------DETTFLFTSESVGEGHPDKIADQVSDAILDACLKVDPFSKVACETATKTGLIFVFGEITSKAVLDYQKIVRDTIKRIGYDDSSKGFDYKTCNVLVAVEQQSPDIAQGLVQR--GQNLEEIGAGDQGIMFGYATDETPELMPLSLVLAHNLNKKMAELRRNQTLSWLRPDSKTQVTVRYKKENGALIPLEVDSIVISAQHSPDIDTPQLRVQLKEHVIDKVIPAKYLTDRTIYHIQPSGLFIIGGPQGDAGLTGRKIIVDTYGGHGAHGGGAFSGKDWSKVDRSAAYTARWIAKSIVAAKLARRILVQLSYAIGVAEPLSIFVDTYGTST----K---KDSEILD-IIKKNFDLKPGIIVQQLGLHKP---VGWNYEQTAAYGHFGRE------EFPWEKVKHLDI---------------------------

>Fungi_Neolecta_irregularis

MSIG----------------------------------------DTFLFTSESVGEGHPDKICDQISDAILDACLEQDPMSRVACETAAKTGMIMVFGEISTRAVLDYQSIIRNTIKRIGYDESSKGFDYKTCNVLVAIEQQSPEIAAGVQI---NGALEDLGAGDQGIMFGYATDETEELLPLTVILAHRLNAALAQARRNGSLGYLRPDSKTQVTVEYRSEGGAVIPLRVDTVVVSTQHAEEVTTEDIRKDILQHIIKKVIPSKFLDEKTVYHIQPSGRFVIGGPQGDAGLTGRKIIVDTYGGWGAHGGGAFSGKAKPDIDRSAAYAARWVAKSLVAAGLARRALVQLSYAIGVAEPLSIYVDSYGTSD----K---TSNELVK-VIRENFDLRPGVIVKELGLAKP---I---YSETCTYGHFTNQ------NNAWEKPRPLIF---------------------------

>Fungi_Leucosporidium_creatinivorum

MA------------------------------------------SKFLFTSESVGEGHPDKICDQVSDAIVDACLANDPWSKVAVETAAKTGMIMVFGEITTKSHLDYQKIIRDTIKRIGYDDSEKGFDYKTCNVLVAIEQQSPDIAQGLDH----GALENHGAGDQGIMFGYATNETEELMPLTLMLSHKLNKKMSDLRRSGELGWLRADSKTQVTIEYKQPSGAVRPLRVDTVVISTQHAEEITTANLQSELMEKVVKAVIPANLLNDKTIYHIQPSGRFVIGGPQGDAGVTGRKIIVDTYGGWGAHGGGAFSGKDWSKVDRSGAYAGRWIAKSLIAAGLAERILVQLSYAIGVAEPLSIFVDSYGTNK----E-GYSDEDITK-IITKNFDLRPGVIVKELGLQRP---I---YSQTACYGHFGNN------TYPWEQVKKLDISK-------------------------

>Fungi_Eremothecium_gossypii

MG------------------------------------------KRFLFTSESVGEGHPDKICDQVSDAILDACLREDPLSKVACETAAKTGMIMVFGEITTSAQLDYQKIVRDTVKQIGYDSSEKGFDYKTCNVLVAVEQQSPDIAQGLHY---EKALEELGAGDQGIMFGYATDETPEGLPLTILLAHRLNMAMADARRDGSLPWLRPDTKTQVTVEYEEDNGRWVPQRIDTIVISAQHADDISTEDLRSALRDRIVGKVIPADMLDENTKYYLQPSGRFVIGGPQGDAGLTGRKIIVDAYGGAAAVGGGAFSGKDYSKVDRSAAYAARWVAKSLLAAGLCKRVQVEFAYAIGIAEPLSIHVETYGTST----K---SDDELIE-IIRNNFDLRPGVLVKELDLAKP---I---YLPTASYGHFTNQ------EYSWEKPKPLRF---------------------------

>Fungi_Cryptococcus_neoformans

MTAVN-------------V----N-------GNG--PATD-LAPGHFLFTSESVGEGHPDKICDQVSDAILDACLAEDPFSKVACETAAKTGMIMVFGEITTRAQIDYQKVIRDTIKKIGYDSSEKGFDYKTCNVLVAIEQQSPDIAQGLDH----GALEKIGAGDQGIMFGYATDETPEMMPLTIMLAHKLNAAMATARRDGSMGWLRPDSKTQVTVEYKKEDGAMIPLRVDTVVISTQHAEEISTEQLREEILEKIIKKVIPKNLLDDRVIYHIQPSGRFVIGGPQGDAGLTGRKIIVDTYGGWGAHGGGAFSGKDWSKVDRSAAYTARWIAKSLVAAGLARRALVQLSYAIGVAEPLSIFVDTYGTGK----K---SDAELVG-VIRQNFDLRPGLIVQALNLQQP---Q---YLKTAAYGHFGNP------AYSWEQPKQLNF---------------------------

>Fungi_Schizosaccharomyces_pombe

MA------------------------------------------QTFLFTSESVGEGHPDKICDQISDAILDACLKDDPFSKVACETASKTGMVMVFGEITTRSQIDYQKVIRNTIKSIGYDDSEKGFDYKTCNVLVAIEQQSPDIAQGLHY---EKALEELGAGDQGIMFGYATDETPEKLPLTILLAHKLNAAMSVARRDGSLPWLRPDTKTQVTIEYEEENGAVIPRRVDTIVVSAQHADSISTEDLRSEILEKIIKPTVPAHLLDEKTVYHIQPSGRFVVGGPQGDAGLTGRKIIVDTYGGWGAHGGGAFSGKDYSKVDRSAAYAARWIAKSLVAAGLARRCLVQLSYAIGVAEPLSIFVNTYGTSS----K---TSAELVE-IIRKNFDLRPGVLVKSLKLQTP---F---YLSTASYGHFTDQ------SKPWEQPKELKF---------------------------

>Fungi_Rozella_allomycis_CSF55

MPSDF-------------------------------NVTE-QMDSTFMFTSESVGEGHPDKICDQISDAILDACLAQDPHSKVACETATKTGMIMVLGEITTKANLDYQKIIREKVREIGYDSSEKGFDYKTCNVLVAIEQQSPDIAQGLIQT--STRIEDIGAGDQGIMFGYATDETEEMMPLTIILAHKINMKLSEYRRNGTLPWVWPDCKSQVTIEYKNENGAMIPLRVHTIVISTQHSPDVSLKQMREELIEKVIKSVVPSKYLDESTIYHLQPSGRFIIGGPQGDAGLTGRKIIVDSYGGWGAHGGGAFSGKDFSKVDRSAAYAARWVAKSLVAAGLCRRCLVQVSYAIGVAKPVSIYVDTYGTGV----K---SNLELLR-IVEENFDLRPGIIVRELDLFKP---I---YSKTAVYGHFGRN------EFPWEKPKQLKY---------------------------

>Fungi_Neocallimastix_californiae

M------------------------------------------AETFLFTSESVGEGHPDKICDQISDAILDACLEQDKFSHVACETAAKTGMIMVFGEITTNAKIDFQKVVRSTIKKIGYDDSSKGFDYKTCNVLVAIEQQSPDIAQGLVQV--SNNVEDIGAGDQGIMFGYATDETPELMPLSHILAHKLNYKLAELRRDGTLPWLRPDTKTQVTVEYKIENGVTTPLRVHNVVISTQHSPEITTTELRKELIEKVVKAVIPANLLTEETIYHMQPSGKFIIGGPQGDAGLTGRKIIVDTYGGWGAHGGGAFSGKDWSKVDRSAAYAARWVAKSLVNAKLCRRCLVQISYAIGVSEPCSIFVDTYGTGV----K---SNAEILE-IIKKNFDLRPGVIVQELNLWRP---I---YQETACYGHFGRP------QFTWEKPKELKF---------------------------

>Fungi_Dimargaris_cristalligena

MTH----------------------------------AIP-EQSETFLFTSESVGEGHPDKICDQVSDAILDACLEQDPLSKVACETATKTGMIMVLGEITTKATLDYQRIIRETIKEIGYDDSAKGFDYKTCNVLVAIEQQSPEIAQGLAQN--GVALEDTGAGDQGIMFGYATDETPELMPLTAVLSHKLNAKMSELRRSGELSWLRPDSKTQVTVEYRNENGAMVPQRVHTIVISTQHGPEATNEVIRRELKDKVIDAVVPAHYLSDRTVYHLQPSGQFIIGGPQGDAGVTGRKIIVDTYGGWGAHGGGAFSGKDWSKVDRSAAYAARWIAKSLVHAGLARRALVQVSYAIGVAHPLSIFVDTYGTGK----R---SDADLLK-IINNNFDLRPGVIVKELNLFKP---M---YKRTACYGHFGRE------EFPWEQAKPLKF---------------------------

>Fungi_Zancudomyces_culisetae

MA------------------------------------------ETFLFTSESVGEGHPDKICDQISDAILDACLEQDPMSKVACETAAKTGMIMVFGEITTKAVLDYQKIIRGVIKKIGYDDSSKGFDYKTCNILVGIEQQSPDIYQGLVQN--GSEIENIGAGDQGIMFGYATDETPELMPLTIVLAHKLNMKMAELRRSGDMAWLRPDSKTQVTVEYKNEAGAMIPLRVDTIVISTQHSPDISTEELRKQLKEKVIDTVIPAEYMNEKTILHLQPSGKFVIGGPQGDAGLTGRKIIVDTYGGWGAHGGGAFSGKDFSKVDRSAAYASRWIAKSIVSSGIARRCLVQLSYAIGVAEPLSVYVDTYGTEK----I---PKEKILQ-LIKENFDLRPGVIVQKLDLFKP---I---YQDTACYGHFGRE------EFSWEQPKQLKY---------------------------

>Fungi_Pneumocystis_jirovecii

MT------------------------------------------SRFLFTSESVGEGHPDKICDQISDAILDACLRDDPYSRVACEVATKTGMIMVFGEITTKAVLNYQDIVRNTIKKIGYDSPEKGFDYKTCNILVAIEEQSPDIAQGLHL---ESSLENIGAGDQGIMFGYATNETEELLPLTVLLAHRLNIALAKARRSGEMDWLRPDSKTQVTVEYEQKDGELTPIRVDTIVVSTQHSESITTEEIRREVLEKIIKKVIPSNYLDDKTIFHIQPSGRFVIGGPQGDAGLTGRKIIVDTYGGWGAHGGGAFSGKDFSKVDRSAAYAARWIAKSLVASGLCKRCLVQLSYAIGISYPLSIFIEHYGTSS----K---TSHELVE-IIKKNFDLRPGVIVKELELQKP---I---YSKTSCYGHFTDQ------SSLWEKPKKLAF---------------------------

>Fungi_Malassezia_restricta

MSNKQ----------------------------------V-LPEGHFLFTSESVGEGHPDKICDQISDAILDACLQQDPRSKVACETAAKTGLILVFGEITSKAQLDYQKIIRDTIKEIGYDASEKGFDYKTCNVMVAIEQQSPDIAQGLDH----GSLDDIGAGDQGIMFGYASDESPELMPLTVVYSHKLNHAMASARRNGHLPWLLPDTKTQVTIEYKKDGGAVVPVRVDTIVVSTQHTDDISTEDIRKLIKSEIIDKVIPAHMLDERTVYHIQPSGRFVIGGPQGDAGLTGRKIIVDTYGGWGAHGGGAFSGKDFSKVDRSAAYTARWIAKSLVHAGLARRALVQLSYAIGVAEPLSVYVDTYGTSS----K---SDAELVQ-IIKNNFNLKPGVIVRDLELQKP---I---YRQTAFGGHFGRS------EFSWEQPKELKF---------------------------

>Fungi_Microdochium_bolleyi

MTATNGTNGHAATNTKP------------------------RNEGAFLFTSESVGEGHPDKICDQVSDAILDACLAEDPLSKVACESAAKTGMIMVFGEITTKANLDYQKVVRGAIKDIGYDSSAKGFDYNTCNVLVAIEKQSPDIAQGLHL---DDALENLGAGDQGLMFGYATDETPELHPLSHLLAHKLNKAMSDARRDGTIPWLRPDTKTQVTMEYKNEGGAVVPLRVHTVVISAQHDESVTTEVVRKELKSKIVEKVIPSQYLDEHVILHLQPSGLFIIGGPQGDAGVTGRKIIVDTYGGWGAHGGGAFSGKDFSKVDRSAAYLARWVAKSLVAAGLCRRVLVQLSYAIGVAEPCSVHVDSYGTSD----K---STEELVE-IVRQNFDMRPGPIAKQLNLTRP---I---YNQTAKFGHFGTNQ-----EFTWEQPKQLKF---------------------------

>Fungi_Rhizophagus_irregularis

MS----------------------------------------DDGTFLFTSESVGEGHPDKIADQVSDAILDACLKIDPFSKVACETATKTGLIFVFGEITSKATLDYQQIVRDTIKRIGYDDSSKGFDYKTCNVLVAIEQQSPDIAQGLVQK--GENLEEIGAGDQGIMFGYATDETPELMPLSLVLAHKLNKKMADLRRDQTLNWLRPDSKTQVTVRYKKENGALIPIEVDSIVISIQHSPEIDTPQLRAQIKEHVVNAVIPAKYLTERTIYHMQPSGKFIIGGPQGDAGLTGRKIIVDTYGGHGAHGGGAFSGKDWSKVDRSAAYTARWIAKSLVAAKLARRVLVQLSYAIGVAEPLSIFVDTYGTST----I---KESEILN-IIKKNFNLKPGVIVQQLGLHKP---S---YQQTAAYGHFGRE------EFPWEKVKSLDI---------------------------

>Fungi_Basidiobolus_meristosporus_CBS

MS----------------------------------------DQDTFLFTSESVGEGHPDKICDQISDAILDACLEQDPYSKVACEAASKTGMIMVLGEITTKANLDFQALVRRTVRDIGYTDSEVGFDYKTCNVLIAVEQQSPDIAQGLVRV--SENIEDIGAGDQGIMFGYATDETKELMPLTIMLSHQLNMKLSELRRDGTLPWLRPDTKTQVTVEYKNDHGAMVPLRVDTVVISTQHAPEVDNDTIRSKLKEHVIEKVIPRQFVDEKTVYHLNPSGKFIIGGPQGDAGLTGRKIIVDTYGGWGAHGGGAFSGKDWSKVDRSAAYTARWIAKSLVAAKLARRCLVQLSYAIGVAEPLSVFVDTYGTSK----K---TNEELLQ-IIRDNFDLRPGIIVKELDLWKP---I---YQKTACYGHFGRE------EFSWEKPKNLKF---------------------------

>Fungi_Coemansia_reversa_NRRL_1564

MS---------------------------------------TDSDTFLFTSESVGEGHPDKICDQISDAILDACLEQDPYSKVACETAAKTGMIMVFGEITTKADLDYQKIIRNTIKEIGYDSSDKGFDYKTCNVLVAIEQQSPDIYQGLVQN--GDALENVGAGDQGIMFGYASDETPEYMPITLVFSHKLNQRMADLRRSGELKWLRPDSKTQVTIEYRKENGALVPVRVDTVVISVQHAPEVDNETLRRELRERVINHVIPASLLDERTIYHLQPSGRFIIGGPQGDAGLTGRKIIVDTYGG-GHHGGGCFSGKDYTKVDRSAAYAARYVAKSLVAAKLARRVTVQFSYAIGVAEPLSVYVDTYGTSS----K---SDKELVE-IIRNNFDLRPGAIAKELDLFKP---I---YRQTACYGHFGRS------IFTWEKPKALKF---------------------------

>FUngi_Jaminaea_rosea

MAQN--------------------------------GKAI-LPEGHFLFTSESVGEGHPDKICDQVSDAILDACLAQDPNSKVACETASKTGMIMVFGEITTKAQLDYQKIIRNTIKEIGYDDSEKGFDYKTCNVLVAIEQQSPDIAQGLDH----GDIENHGAGDQGIMFGYATDETPELMPLTLSLAHKLNAALAVARRNGTLGWLRPDSKTQVTVEYKKDGGALIPVRVDTVVVSTQHAEEISTEELRKQVLEKVVKQVIPAKYIDERTVYHIQPSGRFVIGGPQGDAGLTGRKIIVDTYGGWGAHGGGAFSGKDFSKVDRSGAYINRLIAKSIVASGLARRVLVQSAYAIGVARPVGLFVETYGTSK----H---SDSDLVN-LIEKNFDLRPGVMVKTLDLQKP---I---YRKTAAYGHFGHD------DYSWEKPVSLKF---------------------------

>Fungi_Wallemia_mellicola

MPAA-------------------V-------THQ--EKKT-LPDGHFLFTSESVGEGHPDKICDQVSDAILDACLQEDPFSKVACETAAKTGMIMVFGEITTKANLDYQKVIRNTIKEIGYDDSEKGFDYKTCNVLVAIEQQSPDIAQGLDH----GSLENLGAGDQGIMFGYASDETPECMPLTISLAHKLNQALAVARRSGELPWVRPDTKTQVTVEYKKEGGAVIPKRVDTVVASLQHSDDITTEKLREEVLEKIIKRVIPADLLDDDTIYHIQPSGRFVIGGPQGDAGLTGRKIIVDTYGGWGAHGGGAFSGKDFSKVDRSAAYTGRWIAKSLVKAGLVQRCLVQLSYAIGVAEPLSVFVDSYGTAK----KG-HSDAQLVE-IVRNNFNLKPGVIVKSLDLQRP---I---YNQTAKYGHFGHE------GDSWEQPAEIKY---------------------------

>Fungi_Puccinia_sorghi

MLAAATSMLS--------------------------SKPT-LPKDHFLFTSESVGEGHPDKICDQVSDAILDACLKNDPWSKVACETASKTGMIMVFGEITTRSPIDFQKVIRDTIKRIGYDHSDKGFDYKTCNVLVAIEQQSPDIAQGLDH----GSLENHGAGDQGIMFGYATDETAELMPLTLVLAHQLNAKLAEERREGGLNWLRPDSKTQVTIEYKKDNGAVVPIRVDTIVVSTQHAEEISTEDLRVAVMEKIVKQVIPAHLLDDRTIYHIQPAGRFVIGGPQGDAGLTGRKIIVDSYGGWGAHGGGAFSGKDWSKVDRSGAYVARWIAKSLIAAGLARRVLVQLSYAIGVAEPLSIHVDSYGTSQ----K---TDAELVE-IIQRNFDLRPGVVVKELGLQKP---I---YTQTASYGHFGNP------NYPWEQPKQLKF---------------------------

>Fungi_Caulochytrium_protostelioides

MLP--------------------------------------QEGETYLFTSESVGEGHPDKICDQVSDAILDACLAQDKFSRVAVETATKTGMIMVFGEISTNATIDFQKVIRETIKNIGYDDSEKGFDYKTCNVLVAIEQQSPEIAQGLVQQ--GDNIENIGAGDQGIMFGYATDETPEFMPLTLILAHKLNIKLSELRRNGGLSYLRPDSKTQVTVEYKVIGGVPVPQKVHTVVISTQHAGEITTQTVRTDLLEKVVKSVIPAKYLTAETIYHLQPSGRFVVGGPQGDAGCTGRKIIVDTYGGWGAHGGGAFSGKDWSKVDRSAAYTARWVAKSIVAAGLARRALVQLSYAIGVAEPTSVFVDTYGTGT----K---TNAEILA-IIKKNFNLRPGVIVKELDLFQP---I---YGETAKYGHFGRD------QFNWEKPKQLQL---------------------------

>Fungi_Gonapodya_prolifera

MAANG------------------------------------APVESFLFTSESVGEGHPDKICDQVSDAILDACLAQDKFSRVACETATKTGMIMVLGEITTKAVIDYQKVIRETIKKIGYDDSNKGFDYKTCNVLVAIEQQSPDIAQGLVQE--GFDIEKIGAGDQGIMFGYASDETPELMPLTISLAHKLNQRLSETRRNGECSWLRPDTKTQVTVEYHIIDGVPVPKRVDAVVISTQHSEEIDQATLQKELKAKVIDKVIPANLIDDKTKYFLNPSGRFVIGGPQGDAGVTGRKIIVDSY-------GGAFSGKDYTKVDRSAAYAARWIAKSLVAAGLARRALVQCGYAIGVAEPVSIYVDTYGTGK----K---SNTELLE-IIKKNFDLRPGVIIKELDLWRP---I---YTPTACYGHFGRS------EFPWEQPKKLNF---------------------------

>Fungi_Talaromyces_marneffei

MGSLS----------------------------------N-GKTGTFLFTSESVGEGHPDKIADQVSDAVLDACLAEDPLSKVACETATKTGMVMVFGEITTKAKLDYQKIIRGAIQDIGYDDSSKGFDYKTCNVLVAIEQQSPDIAQGLHY---DEALEKLGAGDQGIMFGYATDETPELLPLTLLLSHKLNRTMKEGRLDGTLPWVRPDTKTQVTIEYAHDNGAVKPLRVDTVVISAQHSEDVTTETVRKELLEKVIKKAIPAELLDDRTVYHLQPSGLFIIGGPQGDAGLTGRKIIVDTYGGWGAHGGGAFSGKDYSKVDRSAAYVARWIAKSLVNAKLARRALVQLSYAIGVAEPLSIFVETYGTSE----K---SSDELVK-IIRDNFDLRPGVIVKELDLAKP---I---YFQTAKNGHFTNQ------DFAWEKPKTLKF---------------------------

>Fungi_Brettanomyces_bruxellensis

MST--------------------------------------IDDDHFLFTSESVGEGHPDKICDQVSDAILDACLAEDPSSKVACETAAKTGMIMVFGEITTKAKLDFQKVVRDTIKRIGYDSSEKGFDYNTCNVLVAIEQQSPDIAQGLHL---DESLEDLGAGDQGIMFGYATDETKEKLPLTILLAHKLNKAMADARRSGELPWLRPDTKTQVTIEYKHDGGAVIPLRVHTVVISAQHAPEISTEDIRKQLKSHIIE---------------------------KGDAGLTGRKIIVDTYGGWGAHGGGAFSGKDYSKVDRSAAYAGRWVXKSLVQAGLCKRCLVQLSYAIGVAQPLSIYVDTYGTSK----Y---SSKQLVD-IVRKNFDLRPGALVKALNLARP---I---YQETARYGHFTNQ------SFPWEQPKKLEL---------------------------

>Fungi_Paramicrosporidium_saccamoebae

MSNGHLPTIQIPV----------------------------LEAGHFLFTSESVGEGHPDKICDQISDAVLDACLEQDPFSRVACETAVKTGMVMVFGEITSTAKLDFQKICRDTIRRIGYDDSQKGFDYKTCNVLVAVEEQSPDISQSLTKV--DGDVVDTGAGDQGIMFGYATDETPELMPLSLVLSHKLAAKLAHARRSGELSWLRPDCKSQVTVEYKMENGATVPLRVHTVVLSAQHSEDVSLSEMKELLLESVIKAVIPRQYLDDNTIYHIQPSGKFVIGGPQSDAGLTGRKIIVDTYGGWGAHGGGAFSGKDWTKVDRSAAYAARWIAKSI------------LSYSIGLVMPLSIHVDTYGTSK----L---STPQLVN-LIHQNFDLRPGAIARDLHLDKP---I---FQQTACYGHFGRD------EFAWERPKPLSF---------------------------

>Fungi_Mitosporidium_daphniae

MVPPPSILAHYPQTAFSTD----------------------KHANTFLFTSESVGEGHPDKICDQISDAILDACLAQDPKSRVGVETAAKTGMIVVLGEITSNAVLDVQALVRSVVRDIGYDDSSKGFDYKTCSVLSAIEQQSPDIAQSLMG---HSDIDDIGAGDQGLMFGYATDETPELMPLTVVLAHKIVEALAVARKTKQVEWILPDCKSQVTIEYEMQDGVPVPLRVHTIVLSTQHSPSISLAAMKESLEMEIVKKVIPTHLIDERTILHIQPSGKFVIGGPQGDAGLTGRKIIVDSYGGWGAHGGGAFSGKDPTKVDRSGAYAARWVAKSIVHAGLAKRCLIQLSYAIGIPEPLSIYIETYGTGK----L---SNAELLR-VVKKNFNLRPGAIIRELDLLRP---I---YQATSCYGHFGRA------QFSWEVPKELSLAD-------------------------

>Fungi_Phialophora_attae

MTTGA--------------------------ALN--GVNG-GKKSTFLFTSESVGEVILTRLTDQVSDAVLDACLQIDPKSRVACETATKTGMIMIFGEITCPGKIDYQKVVRDAIKDIGYDDSAKGFDYKTCNLLVAIEEQSPDIAQGLHY---EKSDEELGAGDQGIMFGYATDETPEYLPLTILLAHKLNSAMTDARK----------------------SGAVVPLRVDTVVVSAQHSDDISTESLRKEIKEKIIKKVIPEKYLDEKTIYHIQPSGRFVIGGPQGDAGLTGRKIIVDTYGGWGAHGGGAFSGKDYNKVDRSAAYLARWIAKSLVHAKLARRALVQLSYAIGVAEPLSLFVETYGTSD----K---SSEELVE-IIKQNFDMRPGVIVRELGLDKP---I---YRQTAKNGHFTNQ------SFSWEQPKQLKA---------------------------

>Fungi_Enterocytozoon_bieneusi

MEIG----------------------------------------KKFLFTSESVGEGHPDKLCDRISDAILDAHLKIDPLSKVAVETIVNGHTVFICGEITSHGKVDYKSIVRRVLKDSGYDRDDLGLDYKTVDIQIYLKNQSLDISQSVGY----KGGIETNAGDQGIMFGYATNETSELMPASIMWAHRLVKKLKELRA--TVKFLGPDCKSQVTVEYIQRNGVIVPQRIHTIVISTQHTNDISTEDLRSYLIEYLIKSCFPEKLL-QNTLYYIQSSGRFVIGGPEADAGLTGRKIIVDSYGGFGCHGGGCFSGKDWTKVDRSGAYAARWIAKSLVHAGICKRVLIQLSYAIGVASPLSIFVDTYGTSE----I---DDSEIVN-IIEHNFDLRPGAIAKDLKLDQP---I---FEQTATFGHFGRN------EFPWEIPKQLITNFNTYPDSK------------------

>Fungi_Edhazardia_aedis

MIPL-------------------------------------LLMKRFLFTSESVGEGHPDKICDQISDAIVDYCLGEDPDSKVAIETMVKSNTVILCGEISTNAKPNYAEIVKRVIKHIGYHFDD-VFDYKTVNVLTFINRQSKDIAQAVQL---D-PDEIIGAGDQGIMFGYATNETEEKMPLSLVLAHDIVRMIKELTK--KVKWLRPDCKSQVTLEYENNNGELIPIRVDNIVVSCHHSDEMDVIEVRDFLKKEVLEKVIDKDLL-FNTKFFIQPSGRFLIGGPTADSGLTGRKIIVDTYGGFGAHGGGCFSGKDCSKVDRSGAYAARWIAKSLVYSGLCKRVLVQISYAIGISEPTSLYLDTYGTGI----F---SNDKLLN-IIKENFDLRPNSIVRALKLREP---I---FQNTACFGHFGNP------KYTWERPKKLKYESESPIDN-------------------

>Fungi_Spraguea_lophii

MKPMC---------------------------------------LKYLFTSESVSEGHPDKICDQISDAILDAYLEIDPYAKVAIEATISPNLLLICGEVNSDVHINIEKIAKDTIAEIGYNKTN-GFNIEESTIIVNVNKQSDEINRAVGG-----LDENMGAGDQGMMFGYATDETEERMPLSLMLSHKLIMKLSEMRRSEQIKWLRPDAKTQVTVEYDEINGILTPFRVDTIVVSAQHDEHISLDEIRTTIMEKIIKKVIPVEYL-INTKYHILPSGKFVKGGPQADCGLTGRKIIVDTYGGFGCHGGGCFSGKDPSKVDRSGAYAARWIAKSLVDSKICKRALIQISYAIGIPEPVSIYINTYNTSK----L---LDAEIIE-IIRKNFKLNPGDIIKSLELNKP---I---YKKTAVFGHFGRN------EFSWEKSKKLEFDKTIER---------------------

>Fungi_Nematocida_displodere

MHK--------------------------------------DSGRKFLFTSESVGKGHPDKLCDQISDGIVDACLAQDKFSRVAVDAVIKSGMVMLVGELTTKAVIDHEEITRKVLREAGYTHEW-GLDPEACTILSLITKQSLDISDGLERV--QKDPWDLGAGDQGIMFGYATDETDSRMPQTAALSHALAQGLYELRK--TVDWLGPDSKTQVTMEYEEGIKSLTPMRVDTIVISTQHKEDKGTEEICAFLLEHLIKKVVPSHLL-VNTRYVLQPSGKFVVGGPKADSGLTGRKLVVDSYGGWGAHGGGAYSGKDWSKVDRTGAYAARWIAKSLVDAGICKRVLVQVSYAIGVKDPISLYVDVYGSSP----F---SNEYILT-IIKQNWDLRLGAVVQELSLDRP---I---YQQTSVFGHFGRE------GFPWEVSKAINTPSGKDSGSKVWAVEDCGMCSG------

>Fungi_Penicillium_camemberti

MLLKTE-----------------------------------ITEPTFLFTSESVGEGHPDKVCDQIADAILDACLTQDPLSKVAIEAAARPGLIFVFGVLDTQAQIDVDVIVRMVLKDIGYDSAYQELDYKTCKVMDHIERRTPEVAAPLVF---LSTSDTEAAGDQGIIFGYASNETPQSLPLTIDLSHRITRQMKTSRLDGTLPWLLPDTKTQVTIEYRRKNGETVPLRVHTIVLTAQHTPDVTVEELRREVFEKVICKAVPAQYLDDQTVYYIQPTGDLGVTPSGKFAGVTGRKIVVDTYGGWGAHGGGAFSGKDYRQVDRSAAYMARWIAKSIIHAGLAQRCLIQLSYSIGVAEPLSIFVDAYGTGK----M---TNLQLEK-VVFENFDMRPAFIAKKLGLTKP---I---YYQTSKNGHFTNP------LFPWENPKDLVL---------------------------

>Fungi_Aspergillus_mulundensis

MTIQ-------------------------------------HEPETFLFTSESVGEGHPDKICDQIADAILDECLRHDPLSKVAIEVAVRPGLVIVFGVVDSLACLDVDGIVREVLRDIGYDSPDQELDYRTCDVMDYVEVRSVKLVSGSF----PSRAEKEVAGDQGMVFGYATDETPQLLPLTIDLAHRISRALKAACIEGTLPWLRPDTKVQVTAEYKQEGGHIAPLRVHTVVVTAQHTPDVTVDELRGAIREMVVLKSIPAMYLDARTVYHIQPTGDVGVTPSGKFAGVTGRKIVVDTYGGWGAHGGGAFSGKDFRQVDRSAAYMARWIAKSLVHAGLAQRCLIQLSYSSGIAEPLSIFIDTFGTSQ----V---SVERLKQ-IVRMNFDLRPAGIAMELGLMSP---I---YYATAKNGHFTSG------LFPWERVRELVL---------------------------

>Amoeba_MAT2_Amoeba_proteus

MSTPG------------------------------------APLETYFFTSECVTEGHPDKLCDQVSDAVLDACLSEDPFSKVACETSTKTGLVMIFGEITTRGHPDYQKVVRDAVKHIGFDNGEIGFDYKTCNVMVCIEQQSPDIAGGVHI---GKSDEDLGAGDQGHMFGYATNETPEMMPLTHVLAAKLAKALSDARRSGLVPWLRPDAKTQVTIEYKNDQGRAVPQRVHTVVISAQHSPDVSVEQIRKDLKEKIIIPTIPAQYLDDQTIYHLNPSGRFVIGGPQGDAGTTGRKIIVDTYGGWGAHGGGAFSGKDPTKVDRSAAYACRWIAKSLVAAGLCDRCLVQVSYSIAVAHPMSLFVNSYGTGK----K---SDAELME-IIKANFDLRPGMIVRALNLRRP---I---YFKTASFGHFGRDD----DDFEWEKPKKLVF---------------------------

>Amoeba_Cavenderia_fasciculata

MGGEIFY--------------------------RMNEWGE-MSAQTVLFTSESVTEGHPDKICDQVSDAILDACLAQDPYSKVACETCTKTGLVMVFGEITTKAVVDYQKVVRDTIKKIGFDDSSKGFDYKTCNVLVGIEQQSPDIAQGVHV---GKNLEDIGAGDQGHMFGYATNETVEMMPLTHYLASELVNKLTELRHNGTLAWARPDAKTQVTVEYLKEGGRMTPVRVHTVVISTQHDEDVTNEVIRSELLEKVVKEVIPAKYLDDKTIYHLNPSGRFVIGGPMGDSGLTGRKIIIDSYGGWGAHGGGAFSGKDPSKVDRSGAYAARWIAKSIVAAGLAERCLVQVSYAIGVAKPLSVFVDSYGTGK----K---SDTELLD-IINKNFDLRPGVLTRELDLLRP---I---YQLTAAGGHFGRAL----KEFTWETPKTLTQ---------------------------

>Amoeba_Dictyostelium_purpureum

MS------------------------------------------TTFLFTSESVTEGHPDKLCDQVSDAVLDACLAQDPLSKVACETASKTGMVMILGEITTKAVVDYQTVVRNAVKKIGFDDSSKGFDYKTCNVLVAIEQQSPDIAQGVHI---NKAPEDIGAGDQGHMFGYATNETATLMPLTHFLASELVNKLTELRHNGTLAWARPDAKTQVTVEYEKVGNSIKPKRVHTVVISTQHDENVTNEVIRSELLEKVIKAVIPAEYLDDQTIYHLNPSGRFVIGGPMGDSGLTGRKIIIDTYGGWGGHGGGAFSGKDPTKVDRSGAYAARWVAKSIVAAGLAERCLVQVSYAIGVAKPLSVYVDTYGTGK----K---SNEELTE-IVNNNFDLRPGCLMRDLQLTRP---I---FQKTAAGGHFGRND----PDFTWEQPKQLKF---------------------------

>Amoeba_Tieghemostelium_lacteum

MS-----------------------------------------SQTFLFTSESVTDGHPDKLCDQVSDAVLDACLAQDPHSKVACETCTKTGLVMVFGEITTKAVVDYQKIVRDTVKKIGFDDSSKGFDYKTCNVLVGIEQQSPDIAQGVHI---DKKVEDIGAGDQGHMFGYATNETPELMPLTHYLASELVNKIIECRNNGSLPWARPDAKTQVTVEYKKEGGRLTPLRVHTIVISTQHDEDVTNEVIRNELKEKVIKAVVPSKYLDDNTIYHLNPSGRFVIGGPMGDSGLTGRKIIIDSYGGFGAHGGGAFSGKDPTKVDRSGAYAARWIAKSLVAAGLAERCLVQVSYAIGVAKPLSVFVDTYGTGN----K---SDEEILK-IVENNFDLRPGCLIRDLDLLRP---I---YQLTAVGGHFGRNL----PEFTWEHPKTLKL---------------------------

>Amoeba_Planoprotostelium_fungivorum

MA---------------------------------------APATDFLFTSESVSEGHPDKLCDQVSDSILDACLAQDPDSKVACESAAKTGMVFVFGEITTKAQVDFQARVRQTVKRIGFDDSSKGFDYKTCNVLVAIEQQSPEIAQAVHI---NKNAEDIGAGDQGHMFGYATDESEELMPLTHVLSTRLIQKLSELRNNGALPWLRPDAKTQVTIDYKRINGAVVPQRVHTVVISTQHDENVTNDVLRAELLEKVIKTTIPANLLDDKTIYHLNPSGRFVIGGPQGDAGLTGRKIIIDSYGGWGAHGGGAFSGKDPSKVDRSGAYAARWIAKSLVAAKLAKRVLVQVSYAIGVAHPLSVFVDTYGTGA----R---SDDEILK-IVSANFDLRPGVLIRDLKLKRP---I---YEKTAYFGHFGRND----PDFTWEHPKELKF---------------------------

>Amoeba_Heterostelium_album

MS-----------------------------------------SNTVLFTSESVTEGHPDKICDQVSDAVLDACLAQDPASKVACETCAKTGLIMVFGEITTKAVIDYQKVVRETIKKIGFDDSSKGFDYKTCNVLVAIEQQSPDIAQGVHV---DKNPEDIGAGDQGHMFGYATNETPELMPLTHYLASELVTKLTELRKNGTLPWARPDAKTQVTVEYLKDGGRMIPQRVHTIVISTQHDENVTNEQIRADLKEKVINAVIPAKYLDDATIYHLNPSGRFVIGGPMGDAGLTGRKIIIDSYGGWGAHGGGAFSGKDPSKVDRSGAYAARWIAKSLISAGLADRCLVQVSYAIGVAKPLSVHVDTYGTGK----K---SDTEILQ-IVLNNFDLRPGVLTRDLQLLRP---V---YHKTASGGHFGRND----PDFTWETPKVLKF---------------------------

>Plast_Pyropia_tenera

MAA----------------------------------------MKNFLFTSESVNEGHPDKLCDQVSDAVLDACLAQDPHSKVACETASKTGMVMCFGEITTTAKVNYEEVIREAIKSIGFDAEEKGLDYKTCRVLVELHAQSPEIAAGVHE---GRTSDDLGAGDQGIMFGYATNESEDLMPLTHSLSTKLGHRLTVVRKEGICPWVRPDGKTQVTVEYERDGGALKPKRVHTIVISTQHDDEVSNEKIRSDLMTHVIKEVIPSQYLDDDTIYHLNPSGRFVIGGPEGDAGLTGRKIIIDTYGGWGAHGGGAFSGKDPTKVDRSAAYAARWVAKSMVAAGLAERLLVQVSYGIGIAEPISVFVDSYGTGT----K---PDDELLA-IVKKNFDLRPGVLIKELDLLKP---I---FKKTSAYGHFGRD------GFTWETVKKREL---------------------------

>Plast_Gracilariopsis_chorda

MAE-----------------------------------------SSFLFTSESVNEGHPDKLCDQVSDAVLDACLAQDEWSKVACETASKTGMVMCFGEITTKAKINYEEVIREAIKKIGFDAEEKGLDYKTCRVLVELHAQSPDIAQGVHE---GRTAEDVGAGDQGIMFGYATNESPDYMPLTSSFANKLGYRLTQVRKSGICPWVRPDGKTQVTMEYKSADGKLTPVRVHTVLISTQHDEGVSNEKIRADVIEKVVKHVIPEEYLDDNTIYHINPSGRFVIGGPEGDAGLTGRKIIIDTYGGWGAHGGGAFSGKDPTKVDRSAAYAGRWVAKSVVAAGLADRCLVQVSYGIGIAEPLSIFVDTFGTGK----K---TDAEITD-IVKKNFDLRPGRIIRDLNLLRP---I---YSKTAAYGHFGRDD----EDFTWETPKKLEY---------------------------

>Plast_Chondrus_crispus

MA------------------------------------------STFLFTSESVNEGHPDKLCDQVSDAVLDACLEQDEWSKVACETASKTGMVMCFGEITTKGSIDYERVIRDAIQKIGFDAEEKGLDYKTCRVLVELHAQSPDIAQGVHE---GRTAEDVGAGDQGIMFGYATNESPDYMPLTSSFANKLGYRLTQVRKDGVCPWVRPDGKTQVTMEYKSDAGKLTPLRVHTILISTQHCEDVSNEKIRADLMEKVIKKVIPDKYLDDRTIYHLNPSGRFVIGGPEGDAGLTGRKIIIDTYGGWGAHGGGAFSGKDPTKVDRSAAYAGRWVAKSVVAAGLADRCLVQVSYGIGIAEPLSIFVDTYGTGA----K---SDAEITD-IVKANFDLRPGHIIRELDLLRP---I---YSKTAAYGHFGRDD----SDFTWEKPKTLKF---------------------------

>Plast_Cyanidioschyzon_merolae

MTAT-------------------------------------EEVRRFFFTSESVNEGHPDKLCDQVSDAVLDACLAQDPFSKVACETATKTGLIMVFGEISTKATVDYEKVVRETVREIGFDDEEKGLDFRSCRVLQEIHEQSREIAASVYG---GRDTLDIAAGDQGIMFGYATNETPELMPLSHSLATKLGKRLTDVRKQGILPYIRPDGKTQVTVEYERRGGALKPLRVDTIVISTQHAPHTSQEKLRADLREHVIRPVVPAEFLDDRTRYVLNPSGSFTVGGPAGDAGLTGRKIIIDTYGGWGGHGGGAFSGKDPSKVDRSAAYMARWMAKSVVAAGLAARCLIQLSYSIGVAEPTSIFLDTYGTAK----PD-WTDDQILK-VVERNFDCRPGAIIRELDLLKP---K---YRKTAAYGHFGREDD---PDFTWEIPKRLQVD--------------------------

>Plast_Coccomyxa_subellipsoidea

MGDI----------------------------------------ETFLFTSESVNEGHPDKLADQVSDAVLDACLEQDPYAKVACETATKTNMVMIFGEITTSAKVDYEKVVRDTCREVGFTSDDVGLDADKCKVLVHIEEQSPDIGQGVHGLG-TKTLEEIGAGDQGHMFGYATDETPELMPLTHVLATQLGFRLTEVRKNGTVGWLRPDGKTQVTVEYKKEGGALTPIRVHTILISTQHSPDVTNDKIHADLMEHVIKPVVPEKYLDDKTIFHLNPSGRFVIGGPHGDAGLTGRKIIIDTYGGWGAHGGGAFSGKDPTKVDRSGAYIARQAAKSIVGSGLARRALVQVSYAIGVAQPLSVHVDTYGTGT----I---GDKEILE-AVLKNFDFRPGMIARNLDLNRA---R---YKRTAAYGHFGREPT---SDFTWEKIIDLKAKV-------------------------

>Plast_Chlorella_sorokiniana

MAD-----------------------------------------TTFLFTSESVNEGHPDKLCDQVSDAILDACLEQDPESKVACETATKTNMVMVFGEITTRAKVDYEAVVRKTVKEIGFISDDVGLDSDKCKVLVHLEEQSPDIGQGVHGMG-TKTLEEIGAGDQGHMFGYATDETPELMPLTHVLATQLGYKLTEVRKNGTCPWLRPDGKTQVTVEYKKDGGAVVPLRVHTILISTQHNPDVSNEKIKEDLMEHVIKPVVPEKYLDDKTIFHLNPSGRFVIGGPHGDAGLTGRKIIIDTYGGWGAHGGGAFSGKDPTKVDRSGAYIARQAAKSVVAAGLARRCLVQVSYAIGVPEPLSVFVDSYGTGT----I---PDTDILA-KVKATFDFRPGMIGKALDLKRGGD-R---YIHTAAYGHFGRNDR---PDFTWEKVMPLKDA--------------------------

>Plast_Micractinium_conductrix

MSD-----------------------------------------DHFLFTSESVNEGHPDKLCDQVSDAILDACLEQDPESKVACETATKTNMVMVFGEITTRAKVDYEAVVRKTCREIGFISDDVGLDADKCKVLVHLEEQSPDIGQGVHGMG-TKTLEEIGAGDQGHMFGYATDETPELMPLTHMLATQLGYKLTEVRKNGTCPWLRPDGKTQVSVEYRKEGGAVVPIRVHTILISTQHNPDVSNDKIKEDLMEHVIKPVVPENYLDDKTIFHLNPSGRFVIGGPHGDAGLTGRKIIIDTYGGWGAHGGGAFSGKDPTKVDRSGAYIARQAAKSVVAAKLARRCLVQVSYAIGVPEPLSVFVDSYGTGT----I---PDADILK-KVKATFDFRPGMIGKALDLKRGGD-R---YIHTAAYGHFGRTDR---PDFTWEKVLPLKDA--------------------------

>Plast_Helicosporidium_sp

MTL-----------------------------------------PPHLSCTESVNEGHPDKLADQVSDAILDACLEQDRDSKVACETATKTNMVMVFGEITTTAKVDYEQVVRDTVRKIGFTSDDVGLDADKCKVLVHLEEQSPEIGQGVHGMG-TKTMEEIGAGDQGHMFGYATDETEELMPLTHVLATKLGYRLTEARKNGTLAWLRPDGKTQVTVEYRKEGGAVVPVRVHTVLISTQHSPDVSNDKISEDLMEHVIKPVIPAKYLDDKTIFHLNPSGRFVIGGPHGDAGLTGRKIIIDTYGGWGAHGGGAFSGKDPTKVDRSGAYIARQAAKSVVASGLARRALVQVSYAIGVAMPLSVFVDTYGTGT----V---PDGEILE-KVLSNFDFRPGMIAKALDLRRGGD-R---YIHTAAYGHFGRTDR---PDFTWEKVKDLADKKDKPAGEDLA----------------

>Gymno_MAT1_Quercus_suber

M-------------------------------------------DTFLFTSESVNEGHPDKLCDQISDAVLDACLEQDPESKVACETCTKTNMVMVFGEITTKANVNYEKIVRDTCRTIGFVSDDVGLDADNCKVLVNIEQQSPDIAQGVHGHL-TKRPEEIGAGDQGHMFGYATDETPEFMPLSHVLATKLGARLTDVRKNGTCPWLRPDGKTQVTVEYFNDNGAMVPVRVHTVLISTQHDETVTNDEIAADLKEHVIKPVIPEKYLDEKTIFHLNPSGRFVIGGPHGDAGLTGRKIIIDTYGGWGAHGGGAFSGKDPTKVDRSGAYVVRQAAKSIVANGLARRCIVQVSYAIGVPEPLSVFVDTYGTGK----I---PDKEILK-IVKESFDFRPGMIAINLDLKRGGNGR---FLKTAAYGHFGRDD----TDFTWEVVKPLKWEK---P-QE------------------

>Gymno_MAT2_Quercus_suber

M-------------------------------------------ETFLFTSESVNEGHPDKLCDQISDAVLDACLAQDPESKVACETCTKTNMVMVFGEITTKANVDYEKIVRDTCRDIGFVSDDVGLDADNCKVLVNIEQQSPDIAQGVHGHL-TKRPEEIGAGDQGHMFGYATDETPELMPLSHVLATKLGARLTEVRKNGTCPWLRPDGKTQVTVEYYNEKGAMVPIRVHTVLISTQHDETVTNDEIAADLKEHVIKPVIPEKYLDEKTIFHLNPSGRFVIGGPHGDAGLTGRKIIIDTYGGWGAHGGGAFSGKDPTKVDRSGAYIVRQAAKSIVANGLARRCIVQVSYAIGVPEPLSVFVDTYGTGK----I---PDKEILK-IVKESFDFRPGMISINLDLKRGGNGR---FLKTAAYGHFGRDD----TDFTWEVVKPLKWDK---V-QA------------------

>Gymno_MAT3_Quercus_suber

M-------------------------------------------ETFLFTSESVNEGHPDKLCDQVSDAILDACLEQDPESKVACETCTKTNMVMVFGEITTKAKVDYEKIVRDTCRGIGFVSADVGLDADKCNVLVNIEQQSPDIAQGVHGHL-TKKPEEIGAGDQGHMFGYATDETPELMPLTHVLATKLGAKLTEVRKNKTCPWLRPDGKTQVTVEYQNENGAMVPIRVHTVLISTQHDETVTNDEIAKDLKEHVIKPVIPSKYLDDKTIFHLNPSGRFVIGGPHGDAGLTGRKIIIDTYGGWGAHGGGAFSGKDPTKVDRSGAYIVRQAAKSVVASGLARRCLVQVSYAIGVPEPLSVFVNSYKTGK----I---PDRDILA-LIKENFDFRPGMMAINLDLKRGGNFR---FQKTAAYGHFGRED----PDFTWETVKLLKP-------KA------------------

>Gymno_MAT1_Arachis_duranensis

MAT-----------------------------------------ETFLFTSESVNEGHPDKLCDQVSDAVLDACLQQDPDSKVACETCTKTNMVMVFGEITTKANVDYEKIVRDTCRNIGFVSDDVGLDADKCKVLVNIEQQSPDIAQGVHGHF-TKRPEEVGAGDQGHMFGYATDETPELMPLSHVLATKLGARLTEVRKNGTCAWLRPDGKTQVTVEYYNDNGAMVPVRVHTVLISTQHDETVTNDQIAADLKEHVIKPVIPEKYLDDRTIFHLNPSGRFVIGGPHGDAGLTGRKIIIDTYGGWGAHGGGAFSGKDPTKVDRSGAYIVRQAAKSIVASGLARRCLVQVSYAIGVPEPLSVFVESYGTGK----I---PDKEILQ-IVKENFDFRPGMITINLDLKRGGN-R---FLKTAAYGHFGRDD----ADFTWEIVKPLKWDK---P-QA------------------

>Gymno_MAT3_Arachis_duranensis

M-------------------------------------------ETFLFTSESVNEGHPDKICDQVSDAILDACLEQDPESKVACETCTKTNMVMVFGEITTKANVNYEKIVRDTCRGIGFVSADVGLDADNCKVLVKIEQQSPDIAQGVHGHM-TKKPEEIGAGDQGHMFGYATDETPELMPLTHVLATKLGAKLTEVRKNKTCPWVRPDGKTQVTVEYKNDNGAMIPIRVHTVLISTQHDETVTNDKIASDLKEHVIKPVIPAKYLDDKTIFHLNPSGRFVIGGPHGDAGLTGRKIIIDTYGGWGAHGGGAFSGKDPTKVDRSGAYIVRQAAKSVVASGLARRCLVQVSYAIGVPEPLSVFVDTYKTGK----I---PDKDILA-LIKENFDFRPGMIAINLDLMRGGNFR---YQKTAAYGHFGRDD----PDFTWETVKMLKP-------KA------------------

>Gymno_MAT1_Prosopis_alba

M-------------------------------------------DTFLFTSESVNEGHPDKICDQVSDAILDACLEQDPESKVACETCTKTNMVMVFGEITTKAKVDYEKIVRDTCRGIGFVSADVGLDADNCKVLVNIEQQSPDIAQGVHGHL-TKKPEEIGAGDQGHMFGYATDETPELMPLTHVLATKLGAKLTEVRKNKTCPWLRPDGKTQVTVEYKNDNGAMVPIRVHTVLISTQHDEAVSNEKIAADLKEHVIKPVIPAEYLDDKTIFHLNPSGRFVIGGPHGDAGLTGRKIIIDTYGGWGAHGGGAFSGKDPTKVDRSGAYIVRQAAKSVVASGLARRCIVQVSYAIGVPEPLSVFVDTYKTGK----I---PDRDILA-LIKENFDFRPGMISINLDLKRGGKFR---FQKTAAYGHFGRDD----PDFTWETVKLLKP-------KA------------------

>Gymno_MAT2_Prosopis_alba

M-------------------------------------------ETFLFTSESVNEGHPDKLCDQISDAVLDACLEQDPDSKVACETCTKTNMVMVFGEITTKANVDYEKIVRDTCRNIGFVSDDVGLDADNCKVLVNIEQQSPDIAQGVHGHL-SKRPEEIGAGDQGHMFGYATDETPELMPLSHVLATKLGARLTEVRKNGTCPWLRPDGKTQVTVEYYNDKGAMVPIRVHTVLISTQHDETVTNDEIAADLKEHVIKAVIPEKYLDEKTIFHLNPSGRFVIGGPHGDAGLTGRKIIIDTYGGWGAHGGGAFSGKDPTKVDRSGAYIVRQAAKSIVANGLARRCIVQVSYAIGVPEPLSVFVDTYGTGK----I---PDKEILK-IVKENFDFRPGMISINLDLKRGGNGR---FLKTAAYGHFGRDD----ADFTWEVVKPLKWEK---P-TA------------------

>Gymno_MAT1_Coffea_arabica

M-------------------------------------------DTFLFTSESVNEGHPDKLCDQISDAVLDACLEQDPDSKVACETCTKTNMVMVFGEITTKANVDYEKIVRDTCRNIGFISDDVGLDADNCKVLVNIEQQSPDIAQGVHGHL-TKRPEEIGAGDQGHMFGYATDETPELMPLSHVLATKLGARLTEVRKNGTCPWLRPDGKTQVTVEYCNDNGAMVPVRVHTVLISTQHDETVTNDEIAKDLKEHVIKTVIPEKYLDEKTIFHLNPSGRFVIGGPHGDAGLTGRKIIIDTYGGWGAHGGGAFSGKDPTKVDRSGAYIVRQAAKSIVASGLARRCIVQVSYAIGVPEPLSVFVDTYGTGK----I---PDKEILK-IVKENFDFRPGMIAIHLDLKRGGNSR---FLKTAAYGHFGRDD----GDFTWEVAKSLKWEK---P-QN------------------

>Gymno_MAT2_Coffea_arabica

M-------------------------------------------ETFLFTSESVNEGHPDKLCDQISDAVLDACLAQDPESKVACETCTKTNMVMVFGEITTKAQVDYEKIVRDTCRAIGFVSDDVGLDADNCKVLVNIEQQSPDIAQGVHGHL-TKCPEEVGAGDQGHMFGYATDETPELMPLSHVLATKLGARLTEVRKNGTCPWLRPDGKTQVTVEYYNENGAMVPVRVHTVLISTQHDETVTNDEIAADLKEHVIKPVIPKKYLDEKTVFHLNPSGRFVIGGPHGDAGLTGRKIIIDTYGGWGAHGGGAFSGKDPTKVDRSGAYIVRQAAKSIVGNGLARRCIVQVSYAIGVPEPLSVFVDTYGTGR----I---PDKEILK-IVKDNFDFRPGMIAINLDLKRGGNNR---FLKTAAYGHFGRDD----PDFTWEVVKPLKWEK---G-LA------------------

>Gymno_MAT3_Coffea_arabica

M-------------------------------------------DTFLFTSESVNEGHPDKLCDQISDAVLDACLEQDPDSKVACETCTKTNMVMVFGEITTKANVDYEKIVRDTCRNIGFISDDVGLDADNCKVLVNIEQQSPDIAQGVHGHL-TKRPEEIGAGDQGHMFGYATDETPELMPLSHVLATKLGACLTEVRKNGTCPWVRPDGKTQVTVEYYNDNGAMVPVRVHTVLISTQHDETVTNDEIAKDLKEHVIKTVIPEKYLDEKTIFHLNPSGRFVIGGPHGDAGLTGRKIIIDTYGGWGAHGGGAFSGKDPTKVDRSGAYIVRQAAKSIVASGLARRCIVQVSYAIGVPQPLSVFVDTYGTGK----I---PDKEILK-IVKENFDFRPGMIAIHLDLKRGGNSR---FLKTAAYGHFGRDD----GDFTWEVAKPLKWEK---P-QN------------------

>Gymno_MAT1_Sesamum_indicum

M-------------------------------------------ETFLFTSESVNEGHPDKLCDQISDAVLDACLEQDPDSKVACETCTKTNMVMVFGEITTKANIDYEKIVRDTCRNIGFTSDDVGLDADKCKVLVNIEQQSPDIAQGVHGHL-TKRPEEIGAGDQGHMFGYATDETPEFMPLSHVLATKLGARLTEVRKNGTCPWLRPDGKTQVTVEYYNENGAMVPIRVHTVLISTQHDETVTNDEIARDLKEHVIKPVIPAKYLDEKTIFHLNPSGRFVIGGPHGDAGLTGRKIIIDTYGGWGAHGGGAFSGKDPTKVDRSGAYIVRQAAKSIVANGLARRCIVQVSYAIGVPEPLSVFVDTYGTGK----I---PDKEILK-IVKENFDFRPGMISINLDLKRGSNGR---FLKTAAYGHFGRDD----PDFTWEVVKPLKWEK---P-QN------------------

>Gymno_MAT2_Sesamum_indicum

MRLQEM--------------------------------------ETFLFTSESVNEGHPDKLCDQVSDAVLDACLAQDPESKVACETCTKTNMVMVFGEITTKANVDYEKIVRDTCRAIGFVSDDVGLDADNCKVLVNIEQQSPDIAQGVHGHL-TKRPEEIGAGDQGHMFGYATDETPELMPLSHVLATKLGSRLTEVRKNGTCPWLRPDGKTQVTVEYYNDNGAMVPIRVHTVLISTQHDETVTNDEIAADLKEHVIKPVIPEKYLDEKTIFHLNPSGRFVIGGPHGDAGLTGRKIIIDTYGGWGAHGGGAFSGKDPTKVDRSGAYIVRQAAKSIVAAGLARRCIVQVSYAIGVPEPLSVFVDTYGTGK----I---PDKEILK-IVKENFDFRPGMISINLDLKRGSGNR---FLKTAAYGHFGRDD----PDFTWEVVKPLKWDK---N-QA------------------

>Gymno_MAT3_Sesamum_indicum

M-------------------------------------------ETFLFTSESVNEGHPDKLCDQVSDAILDACLEQDPESKVACETCTKTNMVMVFGEITTKANLNYEKIVRDTCRGIGFTSPDVGLDADNCKVLVNIEQQSPDIAQGVHGHL-TKKPEEIGAGDQGHMFGYATDETPELMPLTHVLATKLGAKLTEVRKNKTCPWLRPDGKTQVTVEYRNDGGAMVPIRVHTVLISTQHDETVTNDKIASDLKEHVIKPVIPAQYLDDKTIFHLNPSGRFVIGGPHGDAGLTGRKIIIDTYGGWGAHGGGAFSGKDPTKVDRSGAYIVRQAAKSVVASGLARRCIVQVSYAIGVAEPLSVFVDTYKTGK----I---PDKDILA-LIKESFDFRPGMIAINLDLKRGGNFR---YQKTAAYGHFGRDD----PDFTWETVKILKP-------KA------------------

>Gymno_MAT1_Cajanus_cajan

M-------------------------------------------DTFLFTSESVNEGHPDKICDQVSDAILDACLEQDPQSKVACETCTKTNMVMVFGEITTKAKVNYEKIVRDTCRGIGFVSAEVGLDADKCNVLVNIEQQSPDIAQGVHGHM-TKKPEEIGAGDQGHMFGYATDETPELMPLTHVLATKIGAKLTEVRKNKTCPWLRPDGKTQVTVEYRNDNGAMVPVRVHTVLISTQHDETVTNEQIGKDLKEHVIKPVVPAEYLDDKTIFHLNPSGRFVIGGPHGDAGLTGRKIIIDTYGGWGAHGGGAFSGKDPTKVDRSGAYIVRQAAKSVVASGLARRCLVQVSYAIGVPEPLSVFVDSYKTGK----I---PDKDILA-LIKENFDFRPGMIAINLDLMRGGNFR---YQKTAAYGHFGRDD----PDFTWETVKMIKP-------SA------------------

>Gymno_MAT2_Cajanus_cajan

MAQ-----------------------------------------ETFLFTSESVNEGHPDKLCDQISDAVLDACLEQDPDSKVACETCTKTNMVMVFGEITTKANVDYEKIVRDTCRNIGFVSDDVGLDADNCKVLVNIEQQSPDIAQGVHGHL-TKRPEEIGAGDQGHMFGYATDETPELMPLSHVLATKLGARLTEVRKNGTCPWLRPDGKTQVTVEYYNDKGAMVPIRVHTVLISTQHDETVTNDEIAADLKEHVIKPVIPEKYLDEKTIFHLNPSGRFVIGGPHGDAGLTGRKIIIDTYGGWGAHGGGAFSGKDPTKVDRSGAYIVRQAAKSIVANGLARRAIVQVSYAIGVPEPLSVFVDTYGTGK----I---PDKEILS-IVKENFDFRPGMISINLDLKRGGNGR---FLKTAAYGHFGRDD----PDFTWEVVKPLKGDK---V-SS------------------

>Gymno_MAT1_Herrania_umbratica

M-------------------------------------------ETFLFTSESVNEGHPDKLCDQISDAVLDACLAQDPDSKVACETCTKTNMVMVFGEITTKANIDYEKIVRDTCRTIGFVSDDVGLDADNCKVLVNIEQQSPDIAQGVHGHF-TKRPEEIGAGDQGHMFGYATDETPEFMPLSHVLATKLGARLTEVRKNGTCPWLRPDGKTQVTVEYYNDNGAMVPVRVHTVLISTQHDETVTNDEIAADLKEHVIKAVIPEKYLDEKTIFHLNPSGRFVIGGPHGDAGLTGRKIIIDTYGGWGAHGGGAFSGKDPTKVDRSGAYIVRQAAKSIVANGLARRCLVQVSYAIGVPEPLSVFVDSYGTGK----I---PDKEILQ-IVKENFDFRPGMITINLDLKRGGNGR---FLKTAAYGHFGRDD----PDFTWEVVKPLKWEK---P-QS------------------

>Gymno_MAT4_Herrania_umbratica

M-------------------------------------------DTFLFTSESVNEGHPDKLCDQVSDAILDACLEQDPESKVACETCTKTNMVMVFGEITTKAKVNYEKIVRDTCRGIGFTSADVGLDADNCKVLVNIEQQSPDIAQGVHGHL-SKKPEEIGAGDQGHMFGYASDETPELMPLTHVLATKLGAKLTEVRKNKTCSWLRPDGKTQVTVEYRNEGGAMVPIRVHTVLISTQHDETVTNEQIAADLKEHVIKPVIPAKYLDDNTIFHLNPSGRFVIGGPHGDAGLTGRKIIIDTYGGWGAHGGGAFSGKDPTKVDRSGAYIVRQAAKSVVASGLARRCIVQVSYAIGVPEPLSVFVDTYKTGK----I---PDKDILE-LIKENFDFRPGMISINLDLMRGGSFR---YQKTAAYGHFGRED----PDFTWEVVKPLKP-------KA------------------

>Gymno_MAT1_Cucurbita_moschata

M-------------------------------------------ETFLFTSESVNEGHPDKLCDQISDAVLDACLAQDPDSKVACETCSKTNMVMVFGEITTKANVDYEKIVRDTCREIGFVSDDVGLDADNCKVLVNIEQQSPDIAQGVHGHF-TKRPEEIGAGDQGHMFGYATDETPELMPLSHVLATKLGARLTEVRKNGTCPWLRPDGKTQVTVEYYNDNGAMVPVRVHTVLISTQHDETVTNDEIAADLKEHVIKPVIPEKYLDEKTIFHLNPSGRFVIGGPHGDAGLTGRKIIIDTYGGWGAHGGGAFSGKDPTKVDRSGAYIVRQAAKSIVASGLARRCIVQVSYAIGVPEPLSVFVDTYKTGK----I---PDKEILE-IVKENFDFRPGMITINLDLKRGGNGR---FLKTAAYGHFGRED----PDFTWEIVKPLKWEK---P-QS------------------

>Gymno_MAT2_Cucurbita_moschata

M-------------------------------------------ETFLFTSESVNEGHPDKLCDQISDAVLDACLAQDPDSKVACETCTKTNMVMVFGEITTKADVDYEKIVRDTCRSIGFISDDVGLDADNCKVLVNIEQQSPDIAQGVHGHF-TKRPEDIGAGDQGHMFGYATDETPELMPLSHVLATKLGARLTEVRKNGTCPWLRPDGKTQVTVEYYNDKGAMVPVRVHTVLISTQHDETVTNDEIAADLKEHVIKPVIPEKYLDEKTIFHLNPSGRFVIGGPHGDAGLTGRKIIIDTYGGWGAHGGGAFSGKDPTKVDRSGAYIVRQAAKSIVASGLARRAIVQVSYAIGVPEPLSVFVDTYGTGK----I---PDKEILK-IVKENFDFRPGMITINLDLKRGGNGR---FLKTAAYGHFGRDD----PDFTWEVVKPLKWEK---P-QS------------------

>Gymno_MAT3_Cucurbita_moschata

M-------------------------------------------DTFLFTSESVNEGHPDKICDQVSDAILDACLEQDPESKVACETCTKTNMVMVFGEITTKANVNYEKIVRDTCRGIGFISADVGLDCDNCKVLVNIEQQSPDIAQGVHGHM-TKKPEEIGAGDQGHMFGYATDETPELMPLTHVLATQLGAKLTEVRKNRTCPWLRPDGKTQVTVEYKNENGAMVPARVHTVLISTQHDETVTNEQIAKDLKEHVIKPVIPAKYLDDNTIFHLNPSGRFVIGGPHGDAGLTGRKIIIDTYGGWGAHGGGAFSGKDPTKVDRSGAYIVRQAAKSVVASGLARRCIVQVSYAIGVAEPLSVFVDTYKTGK----I---PDKDILV-LIKENFDFRPGMIAINLDLKRGGNSR---YQKTAAYGHFGRDD----PDFTWETVKLLKP-------NA------------------

>Gymno_MAT1_Triticum_urartu

MAA-----------------------------------------ETFLFTSESVNEGHPDKLCDQVSDAVLDACLAQDPDSKVACETCTKTNMVMVFGEITTKATVDYEKIVRDTCRNIGFISDDVGLDADHCKVLVNIEQQSPDIAQGVHGHF-TKRPEEIGAGDQGIMFGYATDETPELMPLTHMLATKLGARLTEVRKNGTCAWLRPDGKTQVTIEYLNEGGAMVPVRVHTVLISTQHDETVTNDEIAADLKEHVIKPVIPGKYLDENTIFHLNPSGRFVIGGPHGDAGLTGRKIIIDTYGGWGAHGGGAFSGKDPTKVDRSGAYIARQAAKSIIASGLARRCIVQISYAIGVPEPLSVFVDSYGTGK----I---PDREILK-LVKENFDFRPGMISINLDLKKGGN-R---FIKTAAYGHFGRDD----ADFTWEVVKPLKFDK---T-SA------------------

>Gymno_MAT1_Glycine_max

MAQ-----------------------------------------ETFLFTSESVNEGHPDKLCDQISDAVLDACLEQDPDSKVACETCTKTNMVMVFGEITTKANVDYEKIVRDTCREIGFISDDVGLDADKCKVLVNIEQQSPDIAQGVHGHF-TKRPEEVGAGDQGHMFGYATDETPEYMPLSHVLATKLGARLTEVRKNGTCAWLRPDGKTQVTVEYYNDNGAMVPVRVHTVLISTQHDETVSNDQIAADLKEHVIKPVIPEKYLDEKTIFHLNPSGRFVIGGPHGDAGLTGRKIIIDTYGGWGAHGGGAFSGKDPTKVDRSGAYIVRQAAKSVVANGLARRCIVQVSYAIGVPEPLSVFVDTYGTGK----I---PDKEILQ-IVKENFDFRPGMITINLDLKRGGH-R---FLKTAAYGHFGRDD----ADFTWEVVKPLKSEK---P-QA------------------

>Gymno_MAT3_Glycine_max

M-------------------------------------------ETFLFTSESVNEGHPDKICDQVSDAILDACLEQDPESKVACETCTKTNMVMVFGEITTKAKVNYEKIVRDTCRGIGFVSADVGLDADKCNVLVNIEQQSPDIAQGVHGHL-TKKPEEIGAGDQGHMFGYATDETPELMPLTHVLATKLGAKLTEVRKNKTCPWLRPDGKTQVTVEYRNDGGAMVPIRVHTVLISTQHDETVTNDQIAKDLKEHVIKPVIPAEYLDDNTIFHLNPSGRFVIGGPHGDAGLTGRKIIIDTYGGWGAHGGGAFSGKDPTKVDRSGAYIVRQAAKSVVASGLARRCIVQVSYAIGVPDPLSVFVDTYKTGK----I---PDSDILA-LIKEHFDFRPGMISINLDLMRGGNFR---YQKTAAYGHFGRDD----PDFTWETVKILKP-------SA------------------

>Gymno_MAT1_Morus_notabilis

M-------------------------------------------DTFLFTSESVNEGHPDKLCDQISDAVLDACLEQDPDSKVACETCSKTNMVMVFGEITTKAKVEYEKIVRDTCRSIGFVSDDVGLDADNCKVLVYIEQQSPDIAQGVHGHL-TKRPEEIGAGDQGHMFGYATDETPEFMPLSHVLATKLGARLTEVRKNGTCPWLRPDGKTQVTVEYINDHGAMVPVRVHTVLISTQHDETVTNDKIAADLKEHVIKPVIPEKYLDEKTIFHLNPSGRFVIGGPHGDAGLTGRKIIIDTYGGWGAHGGGAFSGKDPTKVDRSGAYIVRQAAKSIVASGLARRCIVQVSYAIGVPEPLSVFVDTYGTGK----I---PDKEILK-IVKENFDFRPGMIAINLDLKRGGDVR---FLKTAAYGHFGRDD----PDFTWEVVKPLKWEK---P-QE------------------

>Gymno_MAT2_Morus_notabilis

M-------------------------------------------ETFLFTSESVNEGHPDKLCDQISDAVLDACLAQDPDSKVACETCTKTNMVMVFGEITTKANVDYEKIVRETCRNIGFVSDDVGLDADNCKVLVNIEQQSPDIAQGVHGHF-TKRPEEIGAGDQGHMFGYATDETPEFMPLSHVLATKLGARLTEVRKNGTCPWLRPDGKTQVTVEYYNDNGAMVPVRVHTVLISTQHDETVTNDEIAADLKEHVIKPVIPEKYLDEKTIFHLNPSGRFVIGGPHGDAGLTGRKIIIDTYGGWGAHGGGAFSGKDPTKVDRSGAYIVRQAAKSIVANGLARRCIVQVSYAIGVPEPLSVFVDTYGTGK----I---PDKEILK-IVKENFDFRPGMITINLDLKRGGNDR---FLKTAAYGHFGRDD----PDFTWEVVKPLKWEK---P-ES------------------

>Gymno_MAT3_Morus_notabilis

M-------------------------------------------DTFLFTSESVNEGHPDKLCDQVSDAILDACLEQDPESKVACETCTKTSMVMVFGEITTKAEVDYEKIVRDTCRGIGFTSPDVGLDADNCKVLVNIEQQSPDIAQGVHGHL-TKKPEEIGAGDQGHMFGYATDETPELMPLTHVLATKLGARLTEVRKNKTCPWLRPDGKTQVTVEYRNENGAMIPLRVHTVLISTQHDESVSNEEIARDLKEHVIKPVIPAKYLDDKTIFHLNPSGRFVIGGPHGDAGLTGRKIIIDTYGGWGAHGGGAFSGKDPTKVDRSGAYIVRQAAKSVVASGLARRCIVQVSYAIGVPEPLSVYVDTYKTGK----I---PDTEILA-LIKENFDFRPGMMSINLDLKRGGNFR---FQKTAAYGHFGRDD----PDFTWETVKILKP-------KA------------------

>Gymno_MAT1_Durio_zibethinus

M-------------------------------------------ETFLFTSESVNEGHPDKLCDQISDAVLDACLAQDPDSKVACETCTKTNMVMVFGEITTKANVDYEKIVRDTCRSIGFVSDDVGLDADNCKVLVNIEQQSPDIAQGVHGHF-TKRPEEIGAGDQGHMFGYATDETPELMPLSHVLATKLGARLTEVRKNGTCTWLRPDGKTQVTVEYYNDNGAMVPVRVHTVLISTQHDETVTNDEIAADLKEHVIKPVIPEKYLDEKTIFHLNPSGRFVIGGPHGDAGLTGRKIIIDTYGGWGAHGGGAFSGKDPTKVDRSGAYIVRQAAKSIVANGLARRCLVQVSYAIGVPEPLSVFVDSYGTGK----I---SDKEILQ-IVKENFDFRPGMITNNLDLKRGGNGR---FLKTAAYGHFGRDD----PDFTWEVVKPLKWEK---P-QS------------------

>Gymno_MAT2_Durio_zibethinus

M-------------------------------------------ENFLFTSESVNEGHPDKLCDQISDAVLDACLAQDPDSKVACETCTKTNMVMVFGEITTKANVDYEKIVRDTCRSIGFVSDDVGLDADNCKVLVNIEQQSPDIAQGVHGHF-TKRPEEIGAGDQGHMFGYATDETPELMPLSHVLATKLGARLTEVRKNGTCPWLRPDGKTQVTVEYYDDNGAMVPVRVHTVLISTQHDETVTNDEIAADLKEHVIKPVIPEKYLDEKTIFHLNPSGRFVIGGPHGDAGLTGRKIIIDTYGGWGAHGGGAFSGKDPTKVDRSGAYIVRQAAKSIVANGLARRCLVQVSYAIGVPEPLSVFVNSYGTGK----I---LDKEILQ-IVKENFDFRPGMITNNLDLKRGGNSR---FLKTAAYGHFGRDD----PDFTWEVVKPLKWEK---P-QS------------------

>Gymno_MAT3_Durio_zibethinus

M-------------------------------------------DTFLFTSESVNEGHPDKICDQVSDAILDACLEQDPESKVACETCTKTNMVMVFGEITTKAKVNYEKIVRDTCRGIGFTSPDVGLDADNCKVLVNIEQQSPDIAQGVHGHL-TKKPEEIGAGDQGHMFGYATDETPELMPLTHVLATKLGAKLTEVRKNKTCPWLRPDGKTQVTVEYRNEGGAMVPIRVHTVLISTQHDETVTNEQIAADLKEHVIKPVIPAEYLDDNTIFHLNPSGRFVIGGPHGDAGLTGRKIIIDTYGGWGAHGGGAFSGKDPTKVDRSGAYIVRQAAKSVVASGLARRCIVQVSYAIGVPEPLSVFVDTYKTGK----I---PDKDILE-LIKENFDFRPGMISLNLDLMRGGKFR---YQKTAAYGHFGRDD----PDFTWEIVKPLKP-------KA------------------

>Gymno_MAT1_Spatholobus_suberectus

M-------------------------------------------DTFLFTSESVNEGHPDKLCDQISDAVLDACLEQDPESKVACESCTKTNLVMVFGEITTKAKVDYEKIVRETCRSVGFVSSEVGLDADNCKVLVYLEQQSPDIAQGVHGHL-TKRPEDIGAGDQGHMFGYATDETPELMPLSHVLATKLGARLTEVRRNGTCPWLRPDGKTQVTVEYANDHGAVVPVRVHTVLISTQHDETVTNDEIAADLKEHVIKPVIPEKYLDEEAIFHLNPSGRFVIGGPHGDAGLTGRKIIVDTYGGWGAHGGGAFSGKDPTKVDRSGAYIARQAAKSIVANGLARRCIVQISYAIGVPEPLSVFAETFGTGK----I---PDKEILK-LVKENFDFRPGMISINLDLKRGGNGR---FLKTAAYGHFGRDD----PDFTWEVVKPLKWEE---G-QE------------------

>Gymno_MAT2_Spatholobus_suberectus

M-------------------------------------------ETFLFTSESVNEGHPDKICDQVSDAILDACLEQDPESKVACETCTKTNMVMVFGEITTKAKVNYEKIVRDTCRGIGFVSAEVGLDADKCNVLVNIEQQSPDIAQGVHGHL-TKKPEEIGAGDQGHMFGYATDETPELMPLTHVLATKLGAKLTEVRKNKTCPWLRPDGKTQVTVEYRNDNGAMVPIRVHTVLISTQHDETVTNDQIAKDLKEHVIKPVIPAEYLDDKTIFHLNPSGRFVIGGPHGDAGLTGRKIIIDTYGGWGAHGGGAFSGKDPTKVDRSGAYIVRQAAKSVVASGLARRCIVQVSYAIGVPEPLSVFVDTYKTGK----I---PDKDILA-LIKENFDFRPGMIAINLDLMRGGNFR---YQKTAAYGHFGRDD----PDFTWETVKILKP-------SA------------------

>Gymno_MAT1_Manihot_esculenta

M-------------------------------------------ETFLFTSESVNEGHPDKLCDQISDAVLDACLEQDPDSKVACETCTKTNMVMVFGEITTKAKVDYEKIVRDTCRNIGFVSDDVGLDADKCKVLVNIEQQSPDIAQGVHGHF-TKRPEEIGAGDQGHMFGYATDETPEYMPLSHVLATKLGARLTEVRKNGTCPWLRPDGKTQVTVEYYNDNGAMVPVRVHTVLISTQHDETVTNDEIAADLKEHVIKAVIPEKYLDEKTIFHLNPSGRFVIGGPHGDAGLTGRKIIIDTYGGWGAHGGGAFSGKDPTKVDRSGAYIVRQAAKSIVANGLARRCIVQVSYAIGVPEPLSVFVDTYGTGK----I---PDKEILK-IVKENFDFRPGMMTINLDLKRGGN-R---FLKTAAYGHFGRDD----PDFTWEVVKPLKWDK---P-QA------------------

>Gymno_MAT2_Manihot_esculenta

M-------------------------------------------ETFLFTSESVNEGHPDKLCDQVSDAILDACLEQDPDSKVACETCTKTNMVMVFGEITTQANVDYEKIVRDTCRSIGFVSDDVGLDADKCKVLVYIEQQSPDIAQGVHGHL-TKRPEEIGAGDQGHMFGYATDETPELMPLSHVLATKLGARLTEVRKNGTCPWLRPDGKTQVTVEYYNDNGAMVPVRVHTVLISTQHDETVTNDEIAADLKEHVIKPVIPEKYLDEKTIFHLNPSGRFVIGGPHGDAGLTGRKIIIDTYGGWGAHGGGAFSGKDPTKVDRSGAYIVRQAAKSIVANGLARRCIVQVSYAIGVPEPLSVFVDTYGTGK----I---PDKEILK-IVKENFDFRPGMISINLDLKRGGSGR---FLKTAAYGHFGRDD----PDFTWEVVKPLKWEK---P-QA------------------

>Gymno_MAT3_Manihot_esculenta

M-------------------------------------------ETFLFTSESVNEGHPDKLCDQVSDAILDACLEQDPESKVACETCTKTNMVMVFGEITTKAKVNYEKIVRDTCRGIGFTSADVGLDADKCKVLVNIEQQSPDIAQGVHGHL-TKKPEEIGAGDQGHMFGYATDETPELMPLTHVLATKLGAKLTEVRKNKTCPWLRPDGKTQVTVEYKNENGAMVPVRVHTVLISTQHDETVTNDQIAADLKEHVIKPVIPAQYLDDKTIFHLNPSGRFVIGGPHGDAGLTGRKIIIDTYGGWGAHGGGAFSGKDPTKVDRSGAYIVRQAAKSVVASGLARRCIVQVSYAIGVPEPLSVFVDTYKTGK----I---PDKDILA-LIKENFDFRPGMIAINLDLKKGGNFR---YQKTAAYGHFGRDD----PDFTWETVKLLKP-------KA------------------

>Gymno_MAT1_Zea_mays

MAA----------------------------------------LDTFLFTSESVNEGHPDKLCDQVSDAVLDACLAEDPDSKVACETCTKTNMVMVFGEITTKANVDYEKIVRETCRNIGFVSNDVGLDADHCKVLVNIEQQSPDIAQGVHGHF-TKRPEEIGAGDQGHMFGYATDETPELMPLSHVLATKLGARLTEVRKNGTCPWLRPDGKTQVTVEYRNEGGAMVPIRVHTVLISTQHDETVTNDEIAADLKEHVIKPVIPEQYLDEKTIFHLNPSGRFVIGGPHGDAGLTGRKIIIDTYGGWGAHGGGAFSGKDPTKVDRSGAYVARQAAKSIVASGLARRAIVQVSYAIGVPEPLSVFVDTYGTGA----I---PDKEILK-IVKENFDFRPGMIIINLDLKKGGNGR---YLKTAAYGHFGRDD----PDFTWEVVKPLKSEK---P-SA------------------

>Gymno_MAT1_Panicum_miliaceum

MAA----------------------------------------VDTFLFTSESVNEGHPDKLCDQVSDAVLDACLAEDPDSKVACETCTKTNMVMVFGEITTKANVDYEKIVRETCRNIGFVSADVGLDADHCKVLVNIEQQSPDIAQGVHGHF-TKRPEEIGAGDQGHMFGYATDETPELMPLSHVLATKLGARLTEVRKNGTCPWLRPDGKTQVTVEYRNEGGAMVPIRVHTVLISTQHDETVTNDEIAADLKEHVIKPVIPEQYLDEKTIFHLNPSGRFVIGGPHGDAGLTGRKIIIDTYGGWGAHGGGAFSGKDPTKVDRSGAYIARQAAKSIVANGLARRAIVQVSYAIGVPEPLSVFVDTYGTGT----I---PDKEILK-IVKENFDFRPGMIIINLDLKKGGNGR---YLKTAAYGHFGRDD----PDFTWEVVKPLKWEK---P-SA------------------

>Gymno_MAT1_Jatropha_curcas

M-------------------------------------------ETFLFTSESVNEGHPDKLCDQISDAVLDACLEQDPDSKVACETCTKTNMVMVFGEITTKAKVDYEKIVRQTCRGIGFISDDVGLDADKCKVLVNIEQQSPDIAQGVHGHF-TKRPEEIGAGDQGHMFGYATDETPEYMPLSHVLATKLGAKLTEVRKNGTCPWLRPDGKTQVTVEYYNDNGAMVPVRVHTVLISTQHDETVTNDEIAADLKEHVIKPVIPEKYLDEKTIFHLNPSGRFVIGGPHGDAGLTGRKIIIDTYGGWGAHGGGAFSGKDPTKVDRSGAYIVRQAAKSIVANGLARRCIVQVSYAIGVPEPLSVFVDSYGTGK----I---PDKEILK-IVKENFDFRPGMMTINLDLKRGGN-R---FLKTAAYGHFGRDD----PDFTWEVVKPLKWEK---P-QA------------------

>Gymno_MAT2_Jatropha_curcas

M-------------------------------------------ETFLFTSESVNEGHPDKLCDQISDAVLDACLAQDPDSKVACETCTKTNLVMVFGEITTKANIDYEKIVRDTCRSIGFTSDDVGLDADHCKVLVNIEQQSPDIAQGVHGHL-TKRPEEIGAGDQGHMFGYATDETPELMPLSHVLATKLGARLTEVRKNGTCPWLRPDGKTQVTVEYYSDNGAMVPVRVHTVLISTQHDETVTNDEIAADLKEHVIKPIIPAKYLDEKTIFHLNPSGRFVIGGPHGDAGLTGRKIIIDTYGGWGAHGGGAFSGKDPTKVDRSGAYIVRQAAKSIVASGLARRCIVQVSYAIGVPEPLSVFVDTYGTGK----I---PDKEILK-IVKENFDFRPGMISINLDLKRGGNGR---FLKTAAYGHFGRDD----ADFTWEVVKPLKWEK---P-QA------------------

>Gymno_MAT1_Hevea_brasiliensis

M-------------------------------------------ETFLFTSESVNEGHPDKLCDQISDAVLDACLAQDPDSKVACETCTKTNMVMVFGEITTKAEVDYEKIVRDTCRYIGFVSDDVGLDADKCKVLVNIEQQSPDIAQGVHGHF-TKRPEEIGAGDQGHMFGYATDETPEYMPLSHVLATKLGARLTEVRKNGTCPWLRPDGKTQVTVEYYNDNGAMVPVRVHTVLISTQHDETVTNDEIAADLKEHVIKPVIPEKYLDEKTIFHLNPSGRFVIGGPHGDAGLTGRKIIIDTYGGWGAHGGGAFSGKDPTKVDRSGAYIVRQAAKSIVANGLARRCIVQVSYAIGVPEPLSVFVDTYGTGK----I---PDKEILK-IVKENFDFRPGMMTINLDLKRGGN-R---FLKTAAYGHFGRDD----PDFTWEVVKPLKWDK---P-QA------------------

>Gymno_MAT3_Hevea_brasiliensis

M-------------------------------------------ETFLFTSESVNEGHPDKLCDQVSDAILDACLEQDPESKVACETCTKTNMVMVFGEITTKAKVNYEKIVRDTCRGIGFTSADVGLDADNCKVLVNIEQQSPDIAQGVHGHL-TKKPEEIGAGDQGHMFGYATDETPELMPLTHVLATKLGAKLTEVRKNKTCPWLRPDGKTQVTVEYKNESGAMIPVRVHTVLISTQHDETVTNEQIAADLKEHVIKPVIPAQYLDDKTIFHLNPSGRFVIGGPHGDAGLTGRKIIIDTYGGWGAHGGGAFSGKDPTKVDRSGAYIVRQAAKSVVASGLARRCIVQVSYAIGVPEPLSVFVDTYKTGK----I---PDKDILA-LIKENFDFRPGMIAINLDLKKGGNFR---YQKTAAYGHFGRDD----PDFTWETVKLLKP-------KA------------------

>Gymno_MAT1_Brachypodium_distachyon

MAA-----------------------------------------ETFLFTSESVNEGHPDKLCDQVSDAVLDACLAQDADSKVACETCTKTNMVMVFGEITTKATVDYEKIVRDTCRNIGFISDDVGLDADRCKVLVNIEQQSPDIAQGVHGHF-TKRPEDIGAGDQGIMFGYATDETPELMPLSHVLATKLGARLTEVRKNGTCAWLRPDGKTQVTVEYLNEGGAMVPVRVHTVLISTQHDETVTNDEIAADLKEHVIKPVIPEKYLDEKTIFHLNPSGRFVIGGPHGDAGLTGRKIIIDTYGGWGAHGGGAFSGKDPTKVDRSGAYIARQAAKSIIASGLARRCIVQISYAIGVPEPLSVFVDSYGTGT----I---PDKEILK-IVKENFDFRPGMISINLDLKKGGN-R---FIKTAAYGHFGRDD----ADFTWEVVKPLKFDK---A-SA------------------

>Gymno_MAT3_Brachypodium_distachyon

MAM-----------------------------------------ETFLFTSESVNEGHPDKLCDQVSDAVLDACLAQDPDSKVACETCTKTNMVMVLGEITTKATVDYEKIVRDTCRNIGFVSDDVGLDADRCKVLVNIEQQSPDIAQGVHGHF-TRRPEEIGAGDQGIMFGYATDETPELMPLSHVLATKLGSRLTDVRKDGTCAWLRPDGKTQVTVEYRNESGAMVPVRVHTVLISTQHDETVTNDEIAADLKEHVIKPVIPAKYLDEKTIFHLNPSGRFVIGGPHGDAGLTGRKIIIDTYGGWGAHGGGAFSGKDPTKVDRSGAYIARQAAKSIIASGLARRCIVQISYAIGVPEPLSVFVDSYGTGK----I---PDREILR-LVKENFDFRPGMISINLDLKKGGN-R---FIKTAAYGHFGRDD----ADFTWEVVKPLKFDK---PASA------------------

>Gymno_MAT4_Brachypodium_distachyon

MAE----------------------------------------IDTFLFTSESVNEGHPDKLCDQISDAVLDACLAEDPDSKVACETCTKTNMVMVFGEITTKANVDYEKIVRDTCRGIGFVSNDVGLDADHCKVLVNIEQQSPDIAQGVHGNF-TKRPEEIGAGDQGHMFGYATDETPELMPLSHVLATKLGARLTEVRKNGTCPWLRPDGKTQVTVEYHNDNGAMVPIRVHTVLISTQHDETVTNDEIAADLKEHVIKPVIPEQYLDEKTIFHLNPSGRFVIGGPHGDAGLTGRKIIIDTYGGWGAHGGGAFSGKDPTKVDRSGAYVARQAAKSIVASGLARRCIVQVSYAIGVPDPLSVFVDTYGTGK----I---PDKEILK-IVKENFDFRPGMIIINLDLKRGGNDR---YLKTAAYGHFGRDG----PDFTWEVVKPLEWKK---P-SA------------------

>Gymno_MAT1_Sorghum_bicolor

MAA-----------------------------------------ESFLFTSESVNEGHPDKLCDQVSDAVLDACLAQDPDSKVACETCTKTNMVMVFGEITTKATVDYEKIVRDTCREIGFVSDDVGLDADRCKVLVNIEQQSPDIAQGVHGHF-TKRPEEIGAGDQGHMFGYATDETPELMPLSHVLATKLGARLTEVRKNGTCAWLRPDGKTQVTVEYVNEGGAMVPVRVHTVLISTQHDETVTNDEIAADLKEHVIKPVIPEKYLDEKTIFHLNPSGRFVIGGPHGDAGLTGRKIIIDTYGGWGAHGGGAFSGKDPTKVDRSGAYIARQAAKSIVASGLARRCLVQVSYAIGVPEPLSVFVDSYGTGS----I---PDKEILK-IVKENFDFRPGMITINLDLKKGGN-R---FIKTAAYGHFGRDD----ADFTWEVVKPLKFDK---A-SA------------------

>Gymno_MAT4_Sorghum_bicolor

MAE----------------------------------------LDTFLFTSESVNEGHPDKLCDQISDAVLDACLAEDPDSKVACETCTKTNMVMVFGEITTKANVDYEKIVRDTCRGIGFVSNDVGLDADHCKVLVNIEQQSPDIAQGVHGHF-TKRPEEIGAGDQGHMFGYATDETPEMMPLSHVLATKLGARLTEVRKNGTCPWLRPDGKTQVTVEYHNDNGAMVPIRVHTVLISTQHDETVTNDEIAADLKEHVIKPVIPEQYLDEKTIFHLNPSGRFVIGGPHGDAGLTGRKIIIDTYGGWGAHGGGAFSGKDPTKVDRSGAYIARQAAKSIVASGLARRCIVQVSYAIGVPEPLSVFVDTYGTGK----I---PDKEILK-IVLENFDLRPGMIIIDLDLKRGGNGR---YLKTAAYGHFGRDD----PDFTWELVKPLKWEN---P-SA------------------

>Gymno_MAT1_Prunus_avium

M-------------------------------------------ETFLFTSESVNEGHPDKLCDQISDAVLDACLAQDPDSKVACETCTKTNMVMVFGEITTKANVDYEKIVRETCRTIGFVSDDVGLDADNCKVLVNIEQQSPDIAQGVHGHF-TKRPEEIGAGDQGHMFGYATDETPELMPLSHVLATKLGARLTEVRKNATCPWLRPDGKTQVTVEYYNENGAMVPVRVHTVLISTQHDETVTNDEIAADLKEHVIKPVVPEKYLDEKTIFHLNPSGRFVIGGPHGDAGLTGRKIIIDTYGGWGAHGGGAFSGKDPTKVDRSGAYIVRQAAKSIVANGLARRALVQVSYAIGVPEPLSVFVDTYGTGK----I---PDREILK-IVKETFDFRPGMITINLDLKRGGGGR---FLKTAAYGHFGRDD----PDFTWEVVKPLKWEK---P-QS------------------

>Gymno_MAT3_Prunus_avium

M-------------------------------------------ETFLFTSESVNEGHPDKLCDQVSDAVLDACLEQDPESKVACETCTKTNMVMVFGEITTQAKVDYEKIVRDTCRGIGFTSADVGLDADNCKVLVNIEKQSPEIAEGVHGHL-TKKPEEIGAGDQGHMFGYATDETPELMPLTHVLATKIGAKLTEVRKNKTVPWLRPDGKTQVTVEYRNENGAMVPIRVHTILISTQHDETVKNEQIASDLKEHVIKPVVPAQFIDDKTIYHLNPSGRFVIGGPHGDAGLTGRKIIIDTYGGWGAHGGGAFSGKDPTKVDRSGAYIVRQAAKSVVASGLARRCIVQVSYAIGVPEPLSVFVDTYKTGK----I---PDKEILV-LIKENFDFRPGMIANNLDLKRGGNFR---YQKTAAYGHFGRDD----PDFTWETVKILKP-------KA------------------

>Gymno_MAT5_Prunus_avium

M-------------------------------------------ETFLFTSESVNEGHPDKLCDQISDAVLDACLAQDPESKVACETCTKTNMVMVFGEITTKANVDYEKIVRDTCRTIGFVSADVGLDADNCKVLVNIEQQSPDIAQGVHGHL-TKRPEEIGAGDQGHMFGYATDETPELMPLSHVLATKLGAKLTEVRKNGTCAWLRPDGKTQVTVEYYNDKGAMVPIRVHTVLISTQHDETVTNDEIAADLKEHVIKPVIPEKYLDEKTIFHLNPSGRFVIGGPHGDAGLTGRKIIIDTYGGWGAHGGGAFSGKDPTKVDRSGAYIVRQAAKSIVASGLARRCIVQVSYAIGVPEPLSVFVDSYGTGK----I---PDKEILK-IVKESFDFRPGMIAINLDLKRGGNGR---FLKTAAYGHFGRDD----TDFTWEVVKPLKWDK---V-QA------------------

>Gymno_MAT1_Arachis_ipaensis

M-------------------------------------------DTFLFTSESVNEGHPDKLCDQISDAVLDACLEQDPESKVACETCTKTNLVMVFGEITTKANVDYEKIVRDTCRGIGFVSDDVGLDADNCNVLVYLEQQSPDIAQGVHGNL-TKRPEEIGAGDQGHMFGYATDETPEFMPLSHVLATQLGARLTEVRKNGTCTWLRPDGKTQVTVEYYNDNGAMVPVRVHTVLISTQHDETVTNDEIAADLKEHVIKHVIPEKYLDEKTIFHLNPSGRFVIGGPHGDAGLTGRKIIVDTYGGWGAHGGGAFSGKDPTKVDRSGAYIARQAAKSIVASGIARRCIVQISYAIGVPDPLSVFIDTYGTGK----V---PDREILK-IVKENFDFRPGMISNDLDLKRGGNGR---FLKTAAYGHFGRDD----PDFTWEIVKPLHWEK---P-QE------------------

>Gymno_MAT3_Arachis_ipaensis

M-------------------------------------------ETFLFTSESVNEGHPDKICDQVSDAILDACLEQDPESKVACETCTKTNMVMVFGEITTKANVNYEKIVRDTCRGIGFVSADVGLDADNCKVLVNIEQQSPDIAQGVHGHM-TKKPEEIGAGDQGHMFGYATDETPELMPLTHVLATKLGAKLTEVRKNKTCPWVRPDGKTQVTVEYKNDNGAMIPIRVHTVLISTQHDETVTNDKIASDLKEHVIKPVIPAKYLDDKTIFHLNPSGRFVIGGPHGDAGLTGRKIIIDTYGGWGAHGGGAFSGKDPTKVDRSGAYIVRQAAKSVVASGLARRCLVQVSYAIGVPEPLSVFVDTYKTGK----I---PDKDILA-LIKENFDFRPGMIAINLDLMRGGNFR---YQKTAAYGHFGRDD----PDFTWETVKMLKP-------KA------------------

>Gymno_MAT1_Solanum_lycopersicum

M-------------------------------------------ETFLFTSESVNEGHPDKLCDQISDAVLDACLEQDPESKVACETCTKTNLVMVFGEITTKAIVDYEKIVRDTCRNIGFVSDDVGLDADNCKVLVYIEQQSPDIAQGVHGHL-TKRPEEIGAGDQGHMFGYATDETPELMPLSHVLATKLGARLTEVRKNGTCAWLRPDGKTQVTVEYSNDNGAMVPIRVHTVLISTQHDETVTNDEIARDLKEHVIKPVIPEKYLDENTIFHLNPSGRFVIGGPHGDAGLTGRKIIIDTYGGWGAHGGGAFSGKDPTKVDRSGAYIVRQAAKSIVASGLARRCIVQVSYAIGVPEPLSVFVDTYGTGK----I---PDREILK-IVKENFDFRPGMMSINLDLKRGGNRR---FLKTAAYGHFGRDD----PDFTWEVVKPLKWEK---P-QD------------------

>Gymno_MAT2_Solanum_lycopersicum

M-------------------------------------------ETFLFTSESVNEGHPDKLCDQVSDAVLDACLAQDPESKVACETCTKTNLVMVFGEITTKANIDYEKIVRDTCREIGFVSPDVGLDADNCRVLVNIEQQSPDIAQGVHGHL-TKRPEEIGAGDQGHMFGYATDETPELMPLSHVLATKLGARLTEVRKNGTCSWLRPDGKTQVTVEYHNDNGAMVPLRVHTVLISTQHDETVTNDEIARDLKEHVIKPVIPEKYLDENTIFHLNPSGRFVIGGPHGDAGLTGRKIIIDTYGGWGAHGGGAFSGKDPTKVDRSGAYIVRQAAKSIVANGLARRCIVQVSYAIGVPEPLSVFVDTYGTGK----I---PDKEILN-IVKENFDFRPGMISINLDLLRGGNGR---FLKTAAYGHFGRDD----PDFTWEVVKPLKWDK---P-EA------------------

>Gymno_MAT3_Solanum_lycopersicum

M-------------------------------------------ETFLFTSESVNEGHPDKLCDQVSDAILDACLEQDPESKVACETCTKTNMVMVFGEITTKATVDYEKIVRDTCRGIGFVSADVGLDADNCKVLVNIEQQSPDIAQGVHGHL-TKKPEEIGAGDQGHMFGYATDETPELMPLTHVLATKLGAKLTEVRKNKTCPWLRPDGKTQVTVEYKNDNGAMVPIRVHTVLISTQHDETVTNDQIAQDLKEHVIKPVIPAKYLDENTIFHLNPSGRFVIGGPHGDAGLTGRKIIIDTYGGWGAHGGGAFSGKDPTKVDRSGAYIVRQAAKSVVASGLARRCIVQVSYAIGVAEPLSVFVDTYKTGT----I---PDKDILV-LIKENFDFRPGMMSINLDLLRGGNYR---YQKTAAYGHFGRDD----PDFTWETVKVLKP-------KA------------------

>Gymno_MAT1_Populus_trichocarpa

M-------------------------------------------ETFLFTSESVNEGHPDKLCDQISDAVLDACLEQDPDSKVACETCTKTNMVMVFGEITTKGKIDYEKIVRDTCRNIGFISDDVGLDADKCKVLVNIEQQSPDIAQGVHGHF-TKRPEEIGAGDQGHMFGYATDETPEYMPLSHVLATKLGARLTEVRKNGTCPWLRPDGKTQVTVEYYNDNGAMVPVRVHTVLISTQHDETVTNDEIAADLKEHVIKPVIPEKYLDEKTIFHLNPSGRFVIGGPHGDAGLTGRKIIIDTYGGWGAHGGGAFSGKDPTKVDRSGAYIVRQAAKSIVANGLARRCIVQVSYAIGVPEPLSVFVDTYGTGK----I---PDKEILK-IVKENFDFRPGMMTINLDLKRGGN-R---FLKTAAYGHFGRDD----PDFTWEVVKPLKWEK---P-QA------------------

>Gymno_MAT2_Populus_trichocarpa

M-------------------------------------------ETFLFTSESVNEGHPDKLCDQVSDAILDACLEQDPDSKVACETCTKTNMVMVFGEITTKANVDYEKIVRSTCRSIGFVSDDVGLDADKCNVLVNIEQQSPDIAQGVHGHL-TKRPEEIGAGDQGHMFGYATDETPELMPLSHVLATKLGARLTEVRKNGTCPWLRPDGKTQVTVEYFNENGAMVPIRVHTVLISTQHDETVTNDEIAADLKEHVIKPVIPEKYLDEKTIFHLNPSGRFVIGGPHGDAGLTGRKIIIDTYGGWGAHGGGAFSGKDPTKVDRSGAYIVRQAAKSIVASGLARRCIVQVSYAIGVPEPLSVFVDTYGTGK----I---PDKEILN-IVKEKFDFRPGMIAISLDLKRGGNGR---FLKTAAYGHFGRDD----PDFTWEVVKPLKSEK---P-QQ------------------

>Gymno_MAT3_Populus_trichocarpa

MA------------------------------------------ETFLFTSESVNEGHPDKLCDQISDAVLDACLAQDPDSKVACETCTKTNMVMVFGEITTKADVDYEKIVRDTCRNIGFTSADVGLDADNCKVLVNIEQQSPDIAQGVHGHF-SKRPEEIGAGDQGHMFGYATDETPELMPLSHVLATKLGARLTEVRKNGTCAWLRPDGKTQVTVEYYNENGAMVPVRVHTVLISTQHDETVTNDEIAADLKEHVIKPVIPEKYLDEKTIFHLNPSGRFVIGGPHGDAGLTGRKIIIDTYGGWGAHGGGAFSGKDPTKVDRSGAYIVRQAAKSIVASGLARRCIVQVSYAIGVPEPLSVFVDTYGTGK----I---PDKEILQ-IVKESFDFRPGMISINLDLKRGGNSR---FLKTAAYGHFGRDD----PDFTWEVVKPLKWDNK--V-QA------------------

>Gymno_MAT4_Populus_trichocarpa

M-------------------------------------------DTFLFTSESVNEGHPDKLCDQVSDAILDACLAQDPESKVACETCSKTNMVMVFGEITTKANVDYEKIVRDTCRGIGFTSADVGLDADNCKVLVNIEQQSPDIAQGVHGHL-TKKPEEIGAGDQGHMFGYATDETPELMPLTHVLATKLGAKLTEVRKNKTCPWLRPDGKTQVTVEYKNEGGAMVPIRVHTILISTQHDENVTNEQIAADLKEHVIKPVIPVQYLDDKTIFHLNPSGRFVIGGPHGDAGLTGRKIIIDTYGGWGAHGGGAFSGKDPTKVDRSGAYIVRQAAKSVVASGLARRCIVQVSYAIGVPEPLSVFVDTYKTGK----I---PDRDILE-LIKENFDFRPGMIAINLDLMRGGNSR---YQKTAAYGHFGRDD----PDFTWETVKLLKP-------NA------------------

>Gymno_MAT1_Betula_luminifera

M-------------------------------------------ETFLFTSESVNEGHPDKLCDQISDAVLDACLAQDLESKVACETCTKTNMVMVFGEITTKANVDYEKIVRDTCRAIGFVSDDVGLDADNCKVLVNIEQQSPDIAQGVHGHL-TKRPEEIGAGDQGHMFGYATDETPELMPLSHVLATKLGARLTEVRKNGTCPWLRPDGKTQVTVEYYKDNGAMVPVRVHTVLISTQHDETVTNDEIAADLKEHVIKPVVPEKYLDEKTIFHLNPSGRFVIGGPHGDAGLTGRKIIIDTYGGWGAHGGGAFSGKDPTKVDRSGAYIVRQAAKSIVASGLARRCIVQVSYAIGVPEPLSVFVDTYGTGK----I---PDKEILK-IVKENFDFRPGMIAINLDLKR-GNGR---FLKTAAYGHFGRDD----ADFTWEVVKPLKWEK---G-QA------------------

>Gymno_MAT2_Betula_luminifera

M-------------------------------------------DTFLFTSESVNEGHPDKLCDQISDAVLDACLEHDPDSKVACETCTKTNMVMVFGEITTKSNVDYEKIVRDTCRSIGFVSDDVGLDADNCKVLVNIEQQSPDIAQGVHGHL-TKRPEEIGAGDQGHMFGYATDETPELMPLSHVLATKLGARLTDVRKNGTCPWSRPDGKTQVTVEYYNDHGAMVPVRVHTVLISTQHDETVTNDEIAADLKEHVIKPVVPEKYLDEKTIFHLNPSGRFVIGGPHGDAGLTGRKIIIDTYGGWGAHGGGAFSGKDPTKVDRSGAYIVRQAAKSIVANGLARRCIVQVSYAIGVPEPLSVFVDTYATGK----I---PDKEILK-IVKENFDFRPGMIAINLDLKRGGNGR---FLKTAAYGHFGRDD----TDFTWEVVKPLKWEK---P-LE------------------

>Gymno_MAT3_Betula_luminifera

M-------------------------------------------DTFLFTSESVNEGHPDKLCDQVSDAILDACLEQDPESKVACETCTKTNMVMVFGEITTKAKVNYEKLVRDTCRGIGFVSPDVGLDADNCKVLVNIEQQSPDIAQGVHGHL-TKKPEEIGAGDQGHMFGYATDETPELMPLTHVLATKLGARLTDVRKNNTCPWLRPDGKTQVTVEYKNENGAMVPLRVHTILISTQHDETVTNEQIAKDLKEHVIKPVVPSQYLDDNTIFHLNPSGRFVIGGPHGDAGLTGRKIIIDTYGGWGAHGGGAFSGKDPTKVDRSGAYIVRQAAKSVVASGLARRCIVQVSYAIGVPEPLSVFVDTYKTGK----I---PDKDILA-LIKESFDFRPGMIAINLDLKRGSNFR---YQKTAAYGHFGRDD----PDFTWETVKLLKP-------KA------------------

>Gymno_MAT1_Spinacia_oleracea

MAS---------------------------------------AVDTFLFTSESVNEGHPDKLCDQISDAVLDACLEQDPESKVACETCTKTNLVMVFGEITTKGNVDYEKIVRQTCRNIGFISPDVGLDADNCKVLVYIEQQSPDIAQGVHGHL-TKRPEEIGAGDQGHMFGYATDETPELMPLSHVLATKLGARLTEVRKNGTCPWLRPDGKTQVTVEYFNENGAMVPIRVHTVLISTQHDETVTNDEIAADLKEHVIKPVIPEKYLDEKTIFHLNPSGRFVIGGPHGDAGLTGRKIIIDTYGGWGAHGGGAFSGKDPTKVDRSGAYIARQAAKSIVASGLARRCITQISYAIGVPEPLSVFVDTYGTGK----I---PDKEILK-IVKESFDFRPGMIAINLDLLKGGS-R---YLKTAAYGHFGRDD----PDFTWEVVKSLKWDK---P-QA------------------

>Gymno_MAT2_Spinacia_oleracea

M-------------------------------------------ETFLFTSESVNEGHPDKLCDQVSDAVLDACLEQDPDSKVACETCTKTNMVMVFGEITTKANVDYEKIVRDTCRSIGFISDDVGLDADKCKVLVNIEQQSPDIAQGVHGHL-TKRPEEIGAGDQGHMFGYATDETPELMPLSHVLATKLGARLTEVRKNGTCAWLRPDGKTQVTVEYYNENGAMVPVRVHTVLISTQHDETVTNDQIAADLKEHVIKPVIPEKYLDDKTIFHLNPSGRFVIGGPHGDAGLTGRKIIIDTYGGWGAHGGGAFSGKDPTKVDRSGAYVVRQAAKSIVANGLARRAIVQVSYAIGVPEPLSVFVDTYGTGK----I---HDREILK-IVKENFDFRPGMISINLDLKRGGNGR---FQKTAAYGHFGRDD----ADFTWEVVKPLKWEK---I-PA------------------

>Gymno_MAT1_Eutrema_salsugineum

M-------------------------------------------ETFLFTSESVNEGHPDKLCDQISDAVLDACLEQDPDSKVACETCSKTNMVMVFGEITTKAKVDYEKIVRDTCRAIGFVSDDVGLDADNCKVLVNIEQQSPDIAQGVHGHF-TKRPEDIGAGDQGHMFGYATDETPELMPLSHVLATKLGARLTEVRKNGTCAWLRPDGKTQVTVEYYNDKGAMVPIRVHTVLISTQHDETVTNDEIARDLKEHVIKPVIPEKYLDEKTIFHLNPSGRFVIGGPHGDAGLTGRKIIIDTYGGWGAHGGGAFSGKDPTKVDRSGAYIVRQAAKSVVANGMARRALVQVSYAIGVPEPLSVFVDTYGTGL----I---PDKEILK-IVKESFDFRPGMMTINLDLKRGGNGR---FLKTAAYGHFGRDD----PDFTWEVVKPLKWDK---P-QA------------------

>Gymno_MAT2_Eutrema_salsugineum

M-------------------------------------------ESFLFTSESVNEGHPDKLCDQISDAILDACLEQDPESKVACETCTKTNMVMVFGEITTKANVDYEKIVRKTCREIGFVSDDVGLDADNCKVLVNIEQQSPDIAQGVHGHL-TKKPEEIGAGDQGHMFGYATDETPELMPLSHVLATKLGAKLTEVRKNGTCAWLRPDGKTQVTVEYFNENGAMVPVRVHTVLISTQHDETVTNDEIAADLKEHVIKPVIPEKYLDEKTIFHLNPSGRFVIGGPHGDAGLTGRKIIIDTYGGWGAHGGGAFSGKDPTKVDRSGAYIVRQAAKSIVASGLARRCIVQVSYAIGVPEPLSVFVDSYGTGK----I---PDKEILE-IVKESFDFRPGMISINLDLKRGGNGR---FLKTAAYGHFGRDD----PDFTWEVVKPLKSNK---V-QA------------------

>Gymno_MAT3_Eutrema_salsugineum

M-------------------------------------------ETFLFTSESVNEGHPDKLCDQVSDAILDACLEQDPESKVACETCTKTNMVMVFGEITTSAKVDYEKIVRSTCREIGFISADVGLDADKCNVLVNIEQQSPDIAQGVHGHL-TKKPEDIGAGDQGHMFGYATDETPELMPLTHVLATKLGAKLTEVRKNKTCPWLRPDGKTQVTVEYKNDGGAMIPIRVHTVLISTQHDETVTNDEIAADLKEHVIKPVIPAKYLDDKTIFHLNPSGRFVIGGPHGDAGLTGRKIIIDTYGGWGAHGGGAFSGKDPTKVDRSGAYIVRQAAKSVVAAGLARRCIVQVSYAIGVPEPLSVFVDTYKTGT----I---PDKDILV-LIKEAFDFRPGMMAINLDLKRGGNFR---FQKTAAYGHFGRDD----PDFTWEVVKPLKP-------KA------------------

>Gymno_MAT1_Malus_domestica

M-------------------------------------------ETFLFTSESVNEGHPDKLCDQISDAVLDACLAQDPDSKVACETCTKTNMVMVFGEITTKANVDYEKIVRDTCRAIGFVSDDVGLDADNCKVLVNIEQQSPDIAQGVHGHF-TKRPEEIGAGDQGHMFGYATDETPELMPLSHVLSTKLGARLTEVRKNGTCPWLRPDGKTQVTVEYYNENGAMVPVRVHTVLISTQHDETVTNDEIAADLKEHVIKPVVPEKYLDEKTIFHLNPSGRFVIGGPHGDAGLTGRKIIIDTYGGWGAHGGGAFSGKDPTKVDRSGAYIVRQAAKSIVANGLARRAIVQVSYAIGVPEPLSVFVDTYGTGK----I---PDREILK-IVKETFDFRPGMITINLDLKRGGGGR---FLKTAAYGHFGRDD----PDFTWEVVKPLKWEK---P-QS------------------

>Gymno_MAT2_Malus_domestica

M-------------------------------------------ETFLFTSESVNEGHPDKLCDQISDAVLDACLAQDPDSKVACETCTKTNMVMVFGEITTKANVDYEKIVRDTCRTIGFVSDDVGLDADNCKVLVNIEQQSPDIAQGVHGHF-SKKPEEIGAGDQGHMFGYATDETPELMPLTHVLATKLGAKLTEVRKNGTCAWLRPDGKTQVTIEYVNDKGAMVPIRVHTVLISTQHDETVTNDEIAADLKEHVIKPVIPEKYLDEKTIFHLNPSGRFVIGGPHGDAGLTGRKIIIDTYGGWGAHGGGAFSGKDPTKVDRSGAYIVRQAAKSIVASGLARRCIVQVSYAIGVPEPLSVFVDSYGTGK----I---PDKEILK-IVKESFDFRPGMISINLDLKRGGNDR---FLKTAAYGHFGRDD----PDFTWEVVKPLKWEK---P-QA------------------

>Gymno_MAT3_Malus_domestica

M-------------------------------------------ETFLFTSESVNEGHPDKLCDQVSDAVLDACLEQDPESKVACETCTKTNMVMVFGEITTKAKVDYEKIVRDTCRGIGFTSADVGLDADNCKVLVNIEKQSPEIAEGVHGHL-TKKPEEIGAGDQGHMFGYATDETPEFMPLTHVLATKIGAKLTEVRKNKTVPWLRPDGKTQVTVEYQNENGAMVPIRVHTILISTQHDETVTNEQIAADLKEHVIKPVVPAQFIDDKTIYHLNPSGRFVIGGPHGDAGLTGRKIIIDTYGGWGAHGGGAFSGKDPTKVDRSGAYVVRQAAKSVVASGLARRCIVQVSYAIGVPDPLSVFVDTYRTGK----I---PDKDILV-LIKENFDYRPGMIALNLDLKRGGNFR---YQKTAAYGHFGRDD----PDFTWETIKILKP-------KA------------------

>Gymno_MAT4_Malus_domestica

M-------------------------------------------ETFLFTSESVNEGHPDKLCDQVSDAVLDACLEQDPESKVACETCTKTNMVMVLGEITTKAKVDYEKIVRDTCRGIGFTSADVGLDADNCKVLVNIEKQSPEIAEGVHGHL-TKKPEEIGAGDQGHMFGYATDETPEFMPLTHVLATKIGAKLTEVRKNKTVPWLRPDGKTQVTVEYKNENGAMVPIRVHTILISTQHDENVTNEQIAADLKEHVIKPVVPAQFIDDKIIYHLNPSGRFVIGGPHGDAGLTGRKIIIDTYGGWGAHGGGAFSGKDPTKVDRSGAYIVRQAAKSVVASGLARRCIVQVSYAIGVPEPLSVFVDTYKTGK----I---PDKDILV-LIKENFDFRPGMIAIDLDLKRGGNLR---YQKTAAYGHFGRDD----PDFTWETVKILKP-------KA------------------

>Gymno_MAT1_Vigna_unguiculata

M-------------------------------------------DTFLFTSESVNEGHPDKICDQVSDAVLDACLEQDPDSKVACETCTKTNMVMVFGEITTKAKVNYEKIVRDTCRGIGFVSADVGLDADKCNVLVNIEQQSPDIAQGVHGHL-SKKPEEIGAGDQGHMFGYATDETPELMPLTHVLATKLGAKLTEVRKNKTCPWLRPDGKTQVTVEYRNDGGAMIPIRVHTVLISTQHDETVKNEQIAKDLKEHVIKPVIPAEYLDDKTIFHLNPSGRFVIGGPHGDAGLTGRKIIIDTYGGWGAHGGGAFSGKDPTKVDRSGAYIARQAAKSVVASGLARRCLVQVSYAIGVPEPLSVFVDSHKTGK----I---PDRDILA-LIKENFDFRPGMISINLDLMRGGKFR---YQKTAAYGHFGRDD----PDFTWETVKMLKP-------KA------------------

>Gymno_MAT2_Vigna_unguiculata

M-------------------------------------------ETFLFTSESVNEGHPDKLCDQVSDAILDACLEQDPDSKVACETCTKTNMVMVFGEITTKANVNYEKIVRDTCRGIGFVSADVGLDADNCKVLVNIEQQSPDIAQGVHGHM-TKKPEEIGAGDQGHMFGYATDETPELMPLTHVLSTKLGAKLTEVRKNKTCPWLRPDGKTQVTVEYKNDNGAMIPIRVHTVLISTQHDETVTNEKIAEDLKEKVIKPVIPAKYIDDKTIFHLNPSGRFVIGGPHGDAGLTGRKIIIDTYGGWGAHGGGAFSGKDPTKVDRSGAYIVRQAAKSVVASGLARRCLVQVSYAIGVPEPLSVFVDTYQTGK----I---PDKDILA-LIKEKFDFRPGMIAINLDLMRGGKCR---YLKTAAYGHFGRDD----PDFTWETVKILKP-------NA------------------

>Gymno_MAT1_Citrus_clementina

M-------------------------------------------ETFLFTSESVNEGHPDKLCDQISDAVLDACLEQDPESKVACETCTKTNMVMVFGEITTKAKVDYEKIVRDTCRSIGFVSDDVGLDADHCKVLVNIEQQSPDIAQGVHGHF-TKRPEDIGAGDQGHMFGYATDETSEFMPLSHVLATKLGARLTEVRKNSTCSWLRPDGKTQVTVEYYNDNGAMVPVRVHTVLISTQHDETVTNDEIAADLKEHVIKPVIPEKYLDEKTIFHLNPSGRFVIGGPHGDAGLTGRKIIIDTYGGWGAHGGGAFSGKDPTKVDRSGAYIVRQAAKSIVANGLARRCIVQVSYAIGVPEPLSVFVDSYGTGK----I---PDKEILK-IVKESFDFRPGMMTINLDLKRGGNGR---FLKTAAYGHFGRDD----PDFTWEVVKPLKWEK---P-QA------------------

>Gymno_MAT2_Citrus_clementina

M-------------------------------------------ETFLFTSESVNEGHPDKLCDQISDAILDACLEQDPDSKVACETCTKTNLVMVFGEITTKANVNYEKIVRDTCRNIGFVSDDVGLDADNCKVLVYIEQQSPDIAQGVHGHL-SKRPEEIGAGDQGHMFGYATDETPEFMPLSHVLATQLGAKLTEVRKNGTCPWLRPDGKTQVTVEYYNENGAMVPVRVHTVLISTQHDETVTNDEIAADLKEHVIKPVIPEKYLDEKTIFHLNPSGRFVIGGPHGDAGLTGRKIIIDTYGGWGAHGGGAFSGKDPTKVDRSGAYIVRQAAKSIVASGLARRCIVQVSYAIGVPEPLSVFVDTYRTGK----I---PDKEILR-IVKENFDFRPGMIAINLDLKRGGNSR---FLKTAAYGHFGRED----PDFTWEVVKPLKYEK---P-QE------------------

>Gymno_MAT3_Citrus_clementina

MM------------------------------------------DTFLFTSESVNEGHPDKLCDQISDAILDACLEQDPESKVACETCAKTNMVMVFGEITTKAKVDYEKVVRDTCRGIGFVSADVGLDADKCKVLVNIEEQSPEIAQSVHGNL-SKRPEEIGAGDQGHMFGYATDETPELMPLTLVLATKLGARLTEVRKNKTCPWLRPDGKTQVTVEYRNEGGAMVPQRVHTVLISTQHDETVTKEQISEDLKEHVIKPVIPAHFLDEKTIFHLNPSGRFVIGGPHGDAGLTGRKIIIDTYGGWGAHGGGAFSGKDSTKVDRSAAYIVRQAAKSVVASGLARRCLVQVSYAIGVPEPLSVFVDTYGTGK----I---SDKDILA-LIKENFDFRPGMIAINLDLKRGGNFR---YQKTAAYGHFGRDD----PDFTWETVKLLKP------TEA------------------

>Gymno_MAT1_Arabidopsis_thaliana

M-------------------------------------------ETFLFTSESVNEGHPDKLCDQISDAVLDACLEQDPDSKVACETCTKTNMVMVFGEITTKATVDYEKIVRDTCRAIGFVSDDVGLDADKCKVLVNIEQQSPDIAQGVHGHF-TKCPEEIGAGDQGHMFGYATDETPELMPLSHVLATKLGARLTEVRKNGTCAWLRPDGKTQVTVEYYNDKGAMVPIRVHTVLISTQHDETVTNDEIARDLKEHVIKPVIPEKYLDEKTIFHLNPSGRFVIGGPHGDAGLTGRKIIIDTYGGWGAHGGGAFSGKDPTKVDRSGAYIVRQAAKSVVANGMARRALVQVSYAIGVPEPLSVFVDTYETGL----I---PDKEILK-IVKESFDFRPGMMTINLDLKRGGNGR---FLKTAAYGHFGRDD----PDFTWEVVKPLKWDK---P-QA------------------

>Gymno_MAT2_Arabidopsis_thaliana

M-------------------------------------------ETFLFTSESVNEGHPDKLCDQISDAVLDACLEQDPDSKVACETCTKTNMVMVFGEITTKATIDYEKIVRDTCRSIGFISDDVGLDADKCKVLVNIEQQSPDIAQGVHGHF-TKRPEDIGAGDQGHMFGYATDETPELMPLSHVLATKIGARLTEVRKNGTCRWLRPDGKTQVTVEYYNDNGAMVPVRVHTVLISTQHDETVTNDEIARDLKEHVIKPIIPEKYLDDKTIFHLNPSGRFVIGGPHGDAGLTGRKIIIDTYGGWGAHGGGAFSGKDPTKVDRSGAYIVRQAAKSVVANGMARRALVQVSYAIGVPEPLSVFVDTYGTGL----I---PDKEILK-IVKETFDFRPGMMTINLDLKRGGNGR---FQKTAAYGHFGRDD----PDFTWEVVKPLKWDK---P-QA------------------

>Gymno_MAT3_Arabidopsis_thaliana

M-------------------------------------------ETFLFTSESVNEGHPDKLCDQISDAILDACLEQDPESKVACETCTKTNMVMVFGEITTAAKVDYEKIVRSTCREIGFISADVGLDADKCNVLVNIEQQSPDIAQGVHGHL-TKKPEDIGAGDQGHMFGYATDETPELMPLTHVLATKLGAKLTEVRKNKTCPWLRPDGKTQVTVEYKNDGGAMIPIRVHTVLISTQHDETVTNDEIAADLKEHVIKPVIPAKYLDDNTIFHLNPSGRFVIGGPHGDAGLTGRKIIIDTYGGWGAHGGGAFSGKDPTKVDRSGAYIVRQAAKSVVAAGLARRCIVQVSYAIGVPEPLSVFVDTYKTGT----I---PDKDILV-LIKEAFDFRPGMMAINLDLKRGGNFR---FQKTAAYGHFGRDD----PDFTWEVVKPLKP-------KA------------------

>Chrom_Blastocystis_sp

MES--------------------------------------LPEGHFLFTSESVNEGHPDKLCDQISDAVLDACLAQDPNAKVACETAAKGEMVMIFGEITTTAKVEWEKVVRDTIREIGYDSDAKGLDCDHCNVIVAIEHQSPDIAQGVHI---NKKVEEIGAGDQGIMFGYASNETKELMPLSHLLATKLGKRLTDVRKDGTLKWVRPDGKTQVTVEYKDDHGHMIPIRVHTVVISTQHDDDVSNEQIHKDLMEHVIKPIIPAELLDDKTIYHLNPSGRFVIGGPSGDAGLTGRKIIVDTYGGWGAHGGGAFSGKDSSKVDRSAAYAARWIAKSLVNAGLCKRCLIQLSYAIGVNHPLSIHVDTYGTSE----K---SEAELVD-IINKNFDLRPGCIIRELGLDKP---I---FKATASYGHFGRD------EFPWEKCKELVM---------------------------

>Chrom_Nannochloropsis_gaditana

MSL--------------------------------------EDGTEFLFTSESVNEGHPDKICDQVSDAILDACTKDDENSRVACETCTKTGMVMIFGEISTAATVNYEQVIRDTIKEIGYDDPGKGLDYNTMNVIVAVEEQSPDIAQSVDSR--TGNVEDIGAGDQGIMFGYATDETDELMPLTHLLATSIGARLTEVRKKGILDWVRPDGKTQVTVEYKVEGGRPVPQRVHTVVISTQHSEDVTLEKIREDLLEHVIKPVVPAKYLDDKTIYHLNPSGRFVIGGPHGDAGLTGRKIIIDTYGGWGAHGGGAFSGKDTTKVDRSAAYAARWVAKSLVAAGLCHRVLVQLSYAIGVAHPLSVFVDSYGTAKTRSGK---TDTELCK-IVEKNFDLRPGCIQRDLNLKRP---I---MTKTAAYGHFGRND----PDFTWEQVKTLEI---------------------------

>Chrom_Hondaea_fermentalgiana

MAT-----------------------------------------REYLFTSESVNEGHPDKLADVVSDAVLDACLAQDPDSKVACETATKTKLVMIFGEITSKATVNFEQVVRDAVKNVGYTSPEIGIGHNSCSVVVGIEEQSPDIAQGVHL---GKALEDIGAGDQGIMFGYACDETEELMPASHLWATKIGKRLTDVRKDGTLGWVRPDGKVQVTVAYKEVNGVTEPQYVHTVLISTQHDEGVSQEQIAEDLKREVISKVIPEKYITPETRYVLNPSGRFVIGGPHGDAGLTGRKIIVDTYGGWGAHGGGAFSGKDPTKVDRSAAYAGRWVAKSLVAAGLCRRCLVQLSYGIGVAEPISLHVETYGTVK----EG-MTEAQLKE-IVKNNFDLRPGCLIRDLKLKRP---I---YSKTTCYGHFGRND----PDFTWEQPKELKY---------------------------

>Chrom_Besnoitia_besnoiti

MNA----------------------------------VKP-PRPGHFLFTSESVNEGHPDKLCDQVSDAILDACLEQDPDSKVACETCAKTGMIMIFGEITTKASVNYEQIVRDTVKEIGYDDESKGLDYKTMNVVVAIEEQSPDIAQCVHV---NKKEEDVGAGDQGHMFGYACDETEEFMPLSHSLATRLGKRLTDVRKQGILPYIRPDGKTQVTVEYENRNGVPIPKRVHTIVISTQHAPEASNEQLRADLMEHVVKAVVPAQYLDEDTVYHLNPSGKFVIGGPHGDAGLTGRKIIIDTYGGWGAHGGGAFSGKDSTKVDRSAAYAARQAAKSLVANGFCKRCLVQVSYSIGVSHPLSLFVDSYNTAA----KG-YTDEMLLK-IVMENFDLRPGVIQRRLRLKEP---G---FKRFAAYGHFGRGE----DECTWEKVADLSHCKIPGHETA------------------

>Chrom_Toxoplasma_gondii

MSFLS-----FPLFSS--R----D-------KMN--SVKP-PRPGHFLFTSESVNEGHPDKLCDQVSDAVLDACLAQDPDSKVACETCAKTGMIMIFGEITTKASVNYEQIVRDTVKEIGYDDESKGLDYKTMNVVVAIEEQSPDIAQCVHV---NKKEEEVGAGDQGHMFGYACDETEEFMPLSHSLATRLGKRLTEVRKQGILPYIRPDGKTQVTVEYEDRNGVPVPKRVHTIVISTQHAPEASNEQLRADLMEHVVKYVVPPQYLDEDTVYHLNPSGKFVIGGPHGDAGLTGRKIIIDTYGGWGAHGGGAFSGKDSTKVDRSAAYAARRAAKSLVANGFCKRCLVQVSYSIGVSHPLSLFVNSYNTAA----EN-YTDEMLLK-IVMENFDFRPGVILRELRLKEP---G---FKRFAAYGHFGRTE----EECAWEKVVDLSHCKVPSK-SA------------------

>Chrom_Gregarina_niphandrodes

MAP-----------------------------------------RSFLFTSESVNEGHPDKLCDQVSDAVLDACLKVDPNAKVACETCAKTGFVMIFGEITVGGTVDYEQVVRDAVKRIGYDDPSKGLDYKTMDVRIAIEEQSPEIASGVHI---NKKVEDIGAGDQGHVFGFACNETEDLMPLTHHLANRLAFRLSEVRKNGTLPWVRPDGKTQVTVQYQEEQGHIKPVRVHTILISTQHNPGISQEQIAADLKREVIEPVVPKQFIDDKVLYYLNPSGSFVVGGPHGDAGLTGRKIIVDTYGGWGSHGGGAFSGKDTTKVDRSAAYMARWAAKSLVAADLCDRCTVQVSYAIGVAKPLSMCVNSQNSVK----HG-LKDSDLEA-ILERNFDFRPGCIQEALDLKKP---V---FSETAAYGHFGRP------QFTWEKPKDLSHELKALGI--------------------

>Chrom_Plasmodium_gonderi

MSHLK------------------------------------IKRGNFLFTSESVNEGHPDKICDQISDAILDACLREDPESKVACEVCAKKNFIFILGEITTKAKVDYDKVGRDVLKHIGYDDESKGLDYKTADIKIYIDEQSPDIAQCVHE---NKKPELIGAGDQGIMFGYATDEAENYMPLTHHYATLLGKRLTEVRKLGILPYLGPDGKTQITIEYKNKGGHMEPLRVHTILISTQHTENVKYEQLKSDLIENVVKYVIPEKLLDEDTVYYLNPSGKFVLGGPAADAGLTGRKIICDTYGGWGAHGGGAFSGKDASKVDRSAAYYLRYIAKSLVANKFCRRVLVQASYSIGIANPISLNVNSYGTVT----TG-YTDYDLEQ-IILRNFDLRPGFIIEELKLKEP---I---FSKTSAYGHFGRND----SSFTWEKIKDLTHEKNVLKN--------------------

>Chrom_Cryptosporidium_ubiquitum

MEDSSF-------SGN--I----S-------AY---GDFK-TISEQFLFSSESVCSGHPDKLCDQISDAILDACLEQDPESFVACETCTKTGFIMVFGEITTKANIDYEKIVRETVKEIGYDSEEKGLDYRTMDVIIKLEQQSNQIAGCVHI---NRNVEDIGAGDQGMMFGYATNETKELMPLTHVLATSITRELDHIRMKGIFPWLRPDGKAQVTVEYSCREGELVPKRIHTILVSVQHDEDTKNEEIREFVLNNVIKKVCPSNLMDEKTRILINPSGRFTIGGPAADAGLTGRKIIVDTYGGWGAHGGGAFSGKDATKVDRSGAYMARLVAKSIVFSGLCSRCLVQVSYGIGIAKPLSLYINTFGTAK----NG-YNDTKLLE-IVNEVFDFRPGILIQQLNLKLP---I---FRKTSSGGHFGRTE----EEFLWEKPIKLQ----------------------------

>Chrom_Theileria_orientalis

MTLPKYIN---------------------------------QSSENFLFTSESVSEGHPDKLCDQISDAILDACLEQDPESKVGCEVCATFDTIMVFGEISTKAKVNYENVVRELIREVGYTSQDKGLDYEKVNIILKIKTQSAEIANNVHV---GRSIEQVGAGDQGIMFGYATNETPEAFPLSHLLATKLVQRLAYVRKNNILPYLRPDSKTQVTLEYKKNNDVVEPKRIHTVLLCTQHDPGVDYXEMKKDLMEHVVMKVMPNKLIDENTDFLFNVAGSFVVGGPSSDAGLTGRKIIVDTYGGWGAHGGGCFSGKDATKVDRSGAYYARWVAKSLVANGFCKRVIVQVSYSIGLVDPVSLYVNSYGTVE----KG-YTDGDLEK-IILRNFNFRVGHIIEELKLRRP---I---FRKTSVYGHFGREE----PEFLWEQAKDLSHEKKTNNHTMNGF---------------

>Heterokont_Cylindrotheca_closterium

MS------------------------------------------NTFLFTSESVNEGHPDKLSDQVSDAVLDACLAGDELSRVACETCCKTGMVMIFGEITTSATVNYEQVIRDAVKDIGYDDPAKGFDYKTCNVIVAIEEQSPDIAQSVDA----QKVEDLGAGDQGIMFGYATDETETLMPLTHMLATQLGSKITEVRKNGTCDWVRPDAKTQVTCEYKMENGVPVPVRVHTIVISCQHSEEVTNEQIASDLMEYVIKPVVPEKYLDDNTVYHLNPSGRFVIGGPHGDAGLTGRKIIIDTYGGWGAHGGGAFSGKDTTKVDRSAAYAARWVAKSLVAAGLCKRALVQLSYAIGISHPLSVYVDTYGTTK--EGM---VDSDLIK-IIEKNFDLRPGGIIRDLDLRRP---I---FRKTAAYGHFGRED----PDFTWEKPKKLDL---------------------------

>Heterokont_Ectocarpus_siliculosus

MANIKAPGA--------------------------------PAPGTFLFTSESVNEGHPDKICDQVSDAVLDACLKDDERSRVACETCTKTGMVMIFGEITTNSNVNYEQVIRDALKDIGYDDTAKGLDYKTCNVVVAVEEQSPDIAQSVDA----TRIEDIGAGDQGIMFGYATDETDSLMPLSHELSTQIGARLTEVRKKGICEWCRPDGKTQVTVAYKEENGRMVPQRVHTVVISTQHDDDVSNEKIRADLQEHVIKHVVPAKYLDDDTIYHLNPSGRFVIGGPHGDAGLTGRKIIIDTYGGWGAHGGGAFSGKDPTKVDRSAAYAARWVAKSLVHAGLCHRVLVQLSYAIGVPYPLSIHVDSYGTAKTRSGK---TDEELVE-IINNNFDLRPGCLLRDLQLRKP---I---FRKTAAYGHFGRSD----PEFTWETPKDLTL---------------------------

>Heterokont_Undaria_pinnatifida

MANVKAPGA--------------------------------PEPGAFLFTSESVNEGHPDKICDQVSDAVLDACLKDDERSRVACETCTKTGMVMIFGEITTNANVNYEQVIRDALKEIGYDDTAKGLDYNTCGVVVAVEEQSPDIAQSVDA----ARVEDIGAGDQGIMFGYATDETESLMPLSHELATQLGARLTEVRKKGICDWCRPDGKTQVTVAYKEENGRMVPQRVHTVVISCQHDDDVSNEKIRADLMEHVIKHVVPAKYLDDNTIYHLNPSGRFVIGGPHGDAGLTGRKIIIDTYGGWGAHGGGAFSGKDPTKVDRSAAYAARWVAKSLVSAGLCHRVLVQLSYAIGVPYPLSIHVDSYGTGKTRSGK---SDEDLVE-IINNNFDLRPGCLLRDLSLRRP---I---FQKTAAYGHFGRTD----ADFTWEVPKDLTV---------------------------

>Heterokont_Achnanthes_kuwaitensis

MAS-----------------------------------------STFLFTSESVNEGHPDKLSDQVSDAVLGACIAGDASSRVACETCCKTGMVMIFGEITTSATVNYEQVIRDAIKDIGYDDPAKGLDYKTCNVIVAIEEQSPDIAQSVDA----TKMEDLGAGDQGIMFGYATDETESLMPLTHMLATQLGSKITEVRKNGVCSWVRPDAKTQVTCEYKMEGGVPVPQRVHTVVISCQHSEEVTNEQIAADLMEHVIKVVVPEKYLDDKTVYHLNPPGRFVIGGPHGDAGLTGRKIIIDTYGGWGAHGGGAFSGKDTTKVDRSAAYAARWVAKSLVTAGFCRRCLVQLSYAIGVSHPLSVFVDTYGTIK--EGM---TEEQLVE-IINKNFDLRPGGIIRDLDLRRP---V---MRKTAAYGHFGRED----PDFTWEVPKKLEV---------------------------

>Heterokont_Fragilariopsis_cylindrus

MS-------------------------------------------TFLFTSESVNEGHPDKLSDQVSDAVLDACLAGDEASRVACETCCKTGMVMIFGEITTSATVNYEQVIRDAVKDIGYDDPAKGFDYKTCNVIVAIEEQSPDIAQSVDA----TKMEDLGAGDQGIMFGYATDETETLMPLTHMLATQLGSKITEVRKNGTCDWIRPDAKTQVTCEYKNDNGVPIPQRVHTVVISCQHSEDVTQEQISSDLMEHVVKQVIPEKYLDDNTVYHLNPSGRFVIGGPHGDAGLTGRKIIVDTYGGWGGHGGGAFSGKDSTKVDRSGAYAARWVAKSLVAAGLCKRCVVQLSYAIGVAHPLSVYVDTHGTAK--EGM---TDADLMT-IVEKNFDLRPGCIIRDLELRRP---I---FRKTAAYGHFGRED----PDFTWEKPKKLEL---------------------------

>Heterokont_Fistulifera_solaris

MS-------------------------------------------TFLFTSESVNEGHPDKFCDQVSDAVLDACIAGDEMSRVACETCCKTGMVMIFGEITTSSTVNYEQVIRDAIKDIGYDDPAKGFDYKTCNVIVAIEEQSPDIAQSVDA----VKPEDIGAGDQGIMFGYATDETPELMPLTHVLATQIGAKLTEVRKNGTCDWVRPDGKTQVTCEYKLVDGVPVPQRVHTIVISTQHSEDVTNDQIKADLMEHVIKPVVPEKYLDDNTIYHLNPSGRFVIGGPHGDAGLTGRKIIIDTYGGWGAHGGGAFSGKDTTKVDRSAAYAARWVAKSLVHAKLCKRALVQLSYAIGVPYPLSIFVDSYGTVK--EGM---TDADLTE-VIKKNFDLRPGCIIRDLNLRRP---F---MRKTAAYGHFGRDD----PDFTWEQPKDLNL---------------------------

>Exca_Angomonas_desouzai

MSV-----------------------------------------HTIMFSSEHVTEGHPDKLCDQVSDAVLDACLAGDPFSKVACETCSKTGMVMVFGEITTKTPLDYQKIVRDAVKEIGFDDASKGLDYKSCNLLIAIEQQSPDICQGLG----NFDSEELGAGDQGMMFGYATDETETLMPITYELARGLAVKYSQLRRDGTLPWARPDAKTQVTVQYEYGKQLLTPQRVAVVLISAQHDEAVTNEKIHSDLVEKVIKAVIPANMLDNETKFWINPSGRFVIGGPHGDAGLTGRKIIVDTYGGWGAHGGGAFSGKDPSKVDRSAAYAARWIAKSLVAGGYARRVLVQLAYAIGVSEPLSIFVESYGTGK----Y---DDAKLLE-IVKKNFKLRPYDIIKDLDLRRP---I---YYETSRFGHFGRKDETGKGSFTWEVPKKIVE---------------------------

>Exca_Crithidia_acanthocephali

MSL-----------------------------------------HTILFSSEHVTEGHPDKLCDQVSDAVLDACLAGDPFSKVACETCSKTGMVMVFGEITTKTILDYQKIIRDTIKDIGFDSADKGLDYESCNVLVAIEQQSPDIFQGLG----DFEGENLGAGDQGMMFGYATDETESLMPLTYELARGLAKKISELRRDGTLAWSRPDAKTQVTVEYDYGKQVLTPKRVAVVLISAQHDEHTTNDKIHEDLMEKVVKVVIPANMMDADTKYWLNPSGRFVRGGPHGDAGLTGRKIIVDTYGGWGAHGGGAFSGKDPSKVDRSAAYAARWIAKSIVAGGLARRCLVQLAYAIGVAEPLSMHVESYGTGK----Y---DDAKLLE-IVKKNFKLRPYDIIQELNLRRP---I---YYETSRFGHFGRTDETGKGGFTWEVPKKLVE---------------------------

>Exca_Trypanosoma_congolense

MSV-----------------------------------------RQILFSSEHVSEGHPDKLCDQVSDAVLDACLTLDPLSKVACETCSKTGMVMIFGEITTNAVLDYQSVVRDAVKDIGFDDGEKGLDYRSCNLLIAIEHQSPEIFQGLG----DFDGEELGAGDQGMMFGYATDETETLMPLTYELSRGLAMKYSELRRNGTLPWARPDAKTQVTVQYEYGRQLLTPKRVAVVLISAQHDAEVTNETLRKDLMEKVIKAVVPANMLDADTTYHLNPSGSFVIGGPHGDAGLTGRKIIVDTYGGWGAHGGGAFSGKDPSKVDRSAAYAARWIAKSLVAAGLARRCLVQLAYAIGIAEPLNIHIETYGTGK----Y---DDGRILE-IVKRNFKLRPYDIIKQLDLRRP---I---YHMTSRFGHFGREDKSGQGGFTWEIPKKLDESF-------------------------

>Exca_Herpetomonas_muscarum

MSV-----------------------------------------HTIMFSSEHVTEGHPDKLCDQISDAVLDACLTGDPTSKVACETCSKTGMVMVFGEITTKTVLDYQKVVRDAIKDVGFDNADKGLDYKSCNLLIAIEQQSPDIFQGLG----DFDGEDLGAGDQGMMFGYATDETETLMPLTYELARALAVKYSELRRDGTLAWARPDAKTQVTVQYEYGKQLLTPQRVSVVLISAQHDEQVTNEQIRADLMEKVIKAVIPANMLDADTKYWLNPSGRFVIGGPHGDAGLTGRKIIVDTYGGWGAHGGGAFSGKDPSKVDRSAAYAARWIAKSIVAAGLARRCLVQLAYAIGVAEPLSIFVETYGTGK----Y---DDARLLE-IVKKNFKLRPYDIIKELDLRRP---I---YFQTSRFGHFGRVDTTGKGGFTWENPKTLVE---------------------------

>Exca_Strigomonas_oncopelti

MSV-----------------------------------------HTIMFSSEHVSEGHPDKLCDQISDAVLDACLAGDPFSKVACETCSKTGMVMVFGEITTKTVLDYQKIVRDAVLEIGFDNADKGLDYKSCNLLIAIEHQSPDICQGIG----EFDSESLGAGDQGMMFGYATDETETLMPITYELARQLAMKYSQLRRDGTLAWARPDAKTQVTVQYEYGKQILTPQRVSVVLISAQHDEHVTNVQIKDDLMEKVIRAVIPANMLDADTKYWINPTGRFVIGGPHGDAGLTGRKIIVDTYGGWGAHGGGAFSGKDPSKVDRSAAYAARWIAKSIVAAGLARRCLVQLAYAIGVAEPLSIFVETYGTGK----Y---DDAKLLD-IVKKNFQLRPYDIIKALDLRRP---I---YYETARFGHFGRTDTTGKGSFTWENPKKIVE---------------------------

>Exca_Leishmania_donovani

MSV-----------------------------------------HSILFSSEHVTEGHPDKLCDQVSDAVLDACLAGDPFSKVACESCAKTGMVMVFGEITTKAVLDYQKIVRNTIKDIGFDSADKGLDYESCNVLVAIEQQSPDICQGLG----NFDSEDLGAGDQGMMFGYATDETGTLMPLTYELARGLAKKYSELRRDSSLEWARPDAKTQVTVEYDYGKQVLTPKRVAVVLISAQHDEHVTNDKISVDLMEKVIKAVIPANMLDAETKYWLNPSGRFVRGGPHGDAGLTGRKIIVDTYGGWGAHGGGAFSGKDPSKVDRSAAYAARWIAKSIVAGGLARRCLVQLAYAIGVAEPLSMHVETYGTGK----Y---DDAKLLE-IVKQNFKLRPYDIIQELNLRRP---I---YYETSRFGHFGRKDELGTGGFTWEVPKKMVE---------------------------

>Exca_Stygiella_incarcerata

MSL-----------------------------------------KHFLFSSESVTEGHPDKLCDQVSDAVLDECLRYDPNSRVACETATKTGMVMVFGEITSSARLDFQKIVREKVKEIGFTDSRIGFDYKTCNILVAIEQQSNEIAQSVYL---AKSEEDLGAGDQGIMFGYATDETEEMMPLSHLLATKLARRLSEVRKDGTIPWLRPDGKTQVTVEYERNGHEIRPVRVHTILISSQHDPDVTKEEMTRAFQEHIVIPVC-GKYVDDKTIYHFNPSGSFIIGGPQGDAGLTGRKIIVDTYGGWGAHGGGAFSGKDPTKVDRSASYAARWIAKSVIASGFASRCLVQISYAIGLAEPLSVHVDTYGTGK----I---SDEEILE-KIKANFNLRPYHILHDLQLLRP---I---YSLTASYGHFGRTE----PEFTWEVPRLMK----------------------------

>Exca_Tritrichomonas_foetus

MA------------------------------------------ERFMFTSESVTEGHPDKLCDRVSDTILDECLRQDPNSKVACETSTKTGLVSVFGEITSSADIDYQKLVRTAVADIGYTSSDVCFDAKTCNVMVSVEHQSPDIAQAVHE---NKAEEDLGAGDQGIMFGYATDETKEMMPLTHLLAHNLCRRLTEVRKNGTCPFLGPDGKSQVTAEYERNGSEQKPVRIHTVLISTMHNDSISLDDLRAEVKKHVVDPILPKELVDAETNIYINPGGRFVIGGPLGDAGLTGRKIIVDGYGGWGAHGGGAFSGKDPSKVDRSACYAARWVAKSLVNAGLCHRCLVQLSYAIGVSHPLSIFVDTYGTGK----R---PNADILK-IINKNFDLRPFAIIRDLDLRRP---I---YAQTSAYGHFGRDD----IDLPWEHPKVLDLNI-------------------------

>Diplomonadida_Giardia_lamblia_P15

MSP-----------------------------------------RTFLFTSEQVSEGHPDKICDQISDRILDTILAQDKIARVAADVAIKDNTVFLLGEISTTAVVDYNQIVRDVIREIGYEDEKTSCDWRTVKIINHISSQSPDIYQAVF------RQDELCAGDQGIMIGYASQETKALMPATHLFANLLAMRLSRVRKTGVLPFLRPDSKTQVTIKYEEDTKELRPIFLDTLIISTMHTEEVDNITLRTAIFKHVVIPTLKQLKEDEGTKLLINPSDRFVIGGPKSDCGLTGRKVIADSYGSFGSHGGGAFSGKDASKVDRSGAYLARNIAMSLCSPNICKRCMVQIGYAIGVAEPVSIFVEMYGSEL----KPEYADQQLIERAIRKTFGLTPGKIIQEYSLDTP---I---FFNTSKYGHFGRP------EFPWEQPRRLTLD--------------------------

>Diplomonadida_Giardia_intestinalis

MSP-----------------------------------------RTFLFTSEQVSEGHPDKICDQISDRILDTILAQDKIARVAADVAIKDNTVFLLGEISTTAVVDYNQIVRDVLREIGYEDEKTSCDWRTVEIINRISSQSPDIYQAVF------RQDELCAGDQGIMIGYASQETKALMPATHLFANLLAMRLSRVRKTGVLPFLRPDSKTQVTIKYEEDTKELRPIFLDTLIISTMHAEEIGNTALRTAIFKHVVIPTLKQLKEDEGTKLLINPSDRFVIGGPKSDCGLTGRKVIADSYGSFGSHGGGAFSGKDASKVDRSGAYLARNIAMSLCSPNICKRCMVQIGYAIGVAEPVSIFVEMYGSEL----KPEYADQQLIERAIRRTFGLTPGKIIQEYALDTP---I---FFNTSRYGHFGRS------EFPWEQPRRLTLD--------------------------

>Jakobida_Stygiella_incarcerata

MSL-----------------------------------------KHFLFSSESVTEGHPDKLCDQVSDAVLDECLRYDPNSRVACETATKTGMVMVFGEITSSARLDFQKIVREKVKEIGFTDSRIGFDYKTCNILVAIEQQSNEIAQSVYL---AKSEEDLGAGDQGIMFGYATDETEEMMPLSHLLATKLARRLSEVRKDGTIPWLRPDGKTQVTVEYERNGHEIRPVRVHTILISSQHDPDVTKEEMTRAFQEHIVIPVC-GKYVDDKTIYHFNPSGSFIIGGPQGDAGLTGRKIIVDTYGGWGAHGGGAFSGKDPTKVDRSASYAARWIAKSVIASGFASRCLVQISYAIGLAEPLSVHVDTYGTGK----I---SDEEILE-KIKANFNLRPYHILHDLQLLRP---I---YSLTASYGHFGRTE----PEFTWEVPRLMK----------------------------
