# Supplementary material for: Implications of divergence of methionine adenosyltransferase in archaea
Source: FEBS Open Bio. 2021 Nov 5;12(1):130–45. doi: 10.1002/2211-5463.13312 (PMC8727953; doi:10.1002/2211-5463.13312)
Supplement: Supplementary file 4 — Appendix S3. Eukarya. [file FEB4-12-130-s002.docx]

>Cren_Sulfolobus_solfataricus

M----RNINVQLNPLSDIEKLQVELVERKGLGHPDYIADAVAEEASRKLSLYYLKKYGVILHHNLDKTLVVGGQATPRFKGGDIIQPIYIIVAGRATTEVSGDQIPVGTIIIESVKEWIRNNFRYLDAERHVIVDYKIGKGSSDLVGIFEASK--RVPLSNDTSFGVGFAPLTKLEKLVYETERHLNSKQFKAKLPEVGEDIKVMGLRRGNEVDLTIAMATISELIEDVNHYINVKEQVRNQILDLASKIAPGYNVRVYVNTGDKIDKNILYLTVTGTSAEHGDDGMTGRGNRGVGLITPMRPMSLEATAGKNPVNHVGKLYNVLANLIANKIAQEVKDVKFSQVQVLGQIGRPIDDPLIANVDVITYDGKLTDETKNEISGIVDEMLSSFNKLTELILEGKATLF

>Cren_Thermofilum_pendens

MSA--KNIVVEKSSYIPPSRLPVEIVERKGTGHPDYIADSISEAASRELSRYYLEHYGAILHHNLDKVLVVGGQSSPRFGGGEVVQPIYILVSGRATTEVGGESVPVGPIILKATRDWIKSNIRFLDPDTHVIVDYRVGKGSADLVDIYNRRG--SYPGANDTSMGIGYAPLSPTERAVLETERLLNSEKVKKELPAVGEDVKVMGVRKGNKLTLTVAMAVISRFVHSTEEYLSLKEEVKKLVKEHAASITD-LDVEVYVNTGDDGDKGGLYLTVTGTSAEHGDDGATGRGNRANGLITPFRPMSLEATAGKNPVSHIGKLYNVVAFQAASEIC-GLDHVNEVYIKLISQIGKPINQPLLAYIAINAPDDV-LARVKHQAEEVLAKHLDRINVLWESILKGNVSLF

>Cren_Caldivirga_maquilingensis

MA---RNIVVSGIRRPPTEDLPVELVERKGLGHPDYIADSISEYVSRELSKYYMENFGTILHHNVDKVLVIGGNAQVKFGGGEIIEPIRIIVSGRATTEVTGVKVPIGSIILSAARKFIIDNFRFLNPDQHLVIDYKVGQGSVDLVGVYELGV-SSVPLANDTSIGVGFAPLTVTERLVYETERLLNSREFKARYPEVGEDVKVMGLRRGRKITLTVASALVSRLIKDKDHYISVKEDVVNAIYDNAVKLANGYEVEVHLNTADNPEHGIYYLTYTGTSAEHGDDGMTGRGNRANGLITPMRPMSMEATAGKNPVSHIGKIYYVLANMIAKRIHDEVKGTREVYVYLLSQIGKPIDNPLIANVEIITNEGEVTSEMKREAEAITDEEISRVTRLTSMFVKGEITPF

>Cren_Vulcanisaeta_distributa

MGMA-RTITINTIDRKPVSELNVELVERKGLGHPDYIADASAEAVSKALSNYYLEKYGVILHHNVDKVLVVGGQANPRFGGGDIIQPIYILVAGRVTTEVTGEKVPIGTIIISAIKDWIRSNFRFLDPDRHVVIDYRVGMGSTDLVGVYELGV-KAVPLANDTSIGVGFAPLTDTERLVLETERLLNSREFKAKLPEVGEDVKVMGLRIGKDVKLTIASAIISSLVKDKDHYLNVKEEVKNKVLDLAAKITPNLTVDVTVNAADKPEHGIFYLTVTGTSAEHGDDGATGRGNRANGLITPMRPMTMEATAGKNPVSHIGKLYNVLANRIAERIYGEVKGLKEVYVYLLSQIGKPINEPLVANVEVLPEPGVLSGEIRNEIDGIVNEELDNITSLTELVIKSKVSLF

>Cren_Pyrobaculum_arsenaticum

M-----TVVVEQVDKTPVARRLVEIVERKGQGHPDYIADGISEWVSRYLSRYYLQRFGVILHHNVDKTLVVGGQAAPRFGGGEVLQPIYVLVSGRATYEVDGVKIPLGPVVIQAARDWIKQHFRFLDPDAHVVIDYRIGQGSADLVGIYDLGV-TGVPLANDTSVGVGYAPFTPLEELVYKTERLLNSRDFKAKYPEVGEDVKVMGVRVGKDVRLTVAAAMISRFVKDKSHYLSVKEEVKKVIEDLAAKIAPDYNVDVTINAADKPEFDIFYLTVTGTSAEHGDDGMTGRGNRANGLITPMRSMSLEAAAGKNPVSHVGKIYNVVAQRIADRVYKEVKNIIEVYVEIVSQIGKPINEPKILNVEVIKE-GALTSDTRNEIEAIAREELQRITKVTDLILSGEVSLY

>Cren_Thermoproteus_tenax

M------ITVTKANRIPVEQQRVEIVERKGVGHPDYIADGISEYASVFLSNYYLKKYGLILHHNLDKTLVVGGQARPVFGGGEVIQPIYIIVSGRATVEVNEEKVPIGPIILEAAKRWIRDHFRFLDPERHVVIDYRIGQGSADLTGVYDLGA-KSIPLANDTSIGVGYAPLSTLETLVYQIERTLNSAEFKSRLPEVGEDVKVMGVRVERDVKITIAAAMISSLVKDKDHYLSVKEEVKRRVEDLASKIAPGYNVSVDVNTADKPEHNIYYLTVTGTSAEHGDDGMTGRGNRANGLITPMRVMSMEAAAGKNPVSHVGKIYNILAQKIADRIYKEVKGVVEVSVEVVSQIGRPITEPKILNVELIPEGGVVSSEMKREIEAIAAEELARVTGITQLVLEGKVSLF

>Cren_Metallosphaera_cuprina

M----KNIHIDSFRTLEPDSLPVELAERKGTGHPDFIADSASEEASRKLSLYYLKNYGTVLHHNLDKTLLVGGQASPKFKGGEVIHPIYIVVAGRATTEVDGDNVPVGTIIMDSVKNWIKENFRYLDPEKHVIVDYKIGKGSADLVGIFDKGK-KSVPLSNDTSFGVGFAPYSKLERLVFQTERTLNSKQIKTQIPEVGEDIKVMGLRRDKDVTLTIAMAAISPLIEDRSHYIAVKEQVKDTVLKLASEICPDLNVKVNVNTGDREDQGILYLTVTGTSAEHGDDGMTGRGNRGIGLITPMRPMSLEATAGKNPVNHVGKLYNVVASLIAKKASEQVKDVRNVQIEVLGQIGRPINDPLIINVEVATHSGTITSDTKAEISGIAEEYLDSFDKITQMILEGKVMLF

>Cren_Sulfolobus_tokodaii

M----RNINVQLSHWVDIDSLEVELVERKGTGHPDYIADSASEEASRKLSLYYLKRYGTILHHNLDKTLVVGGQASPRFKGGEVLQPIYIIVAGRATTEVSGESIPVGTIIIESVKEWIKEHFRYLDPEKHVIVDYKIGKGSADLVGIFEVAK-KSVPLSNDTSFGVGFAPYSKLENLVYQTERYLNSKEMKAKIPEIGEDIKVMGLRKGKTIELTIAMAVISQLVSDLNHYIAVKEEAKQAILDLASKLVPDYDVKVNINTGDKIDKGIVYLTVTGTSAEHGDDGMTGRGNRATGLITPMRPMSLEATAGKNPVNHVGKIYNIVANLIAQKVSTEVKGVKNVQVEVLGQIGRPIDDPLIANVQVTTENGSLTSEMKREIEGISDEILGSITKISDLILENKVMLF

>Cren_Acidilobus_saccharovorans

MT---RNIVVRQLSQQPIEDTEVELVERKGLGHPDYISDAVAEEASRQLSAYYKEHFGTIMHHNLDKVLLVGGQASPKWGGGDVITPIYIIVSGRATTEVSGEEVPVGRIIIKAVKDWISRNLRFLDPEVHVVVDYKVGKGSADLRGIFESRT--ASYRANDTSFGAGFAPLSTLERLVFETERLLNSKEFKSRVPASGEDVKVMGLRRGKSIELTVADAIVSRFVNGPEEYMSVKEEIKDAVLNLASKLAPGYDVKVYINSGDIPEEEVFYLTVTGTSAEHGDDGATGRGNRVNGLITPMRPMSLEAAAGKNPVTHVGKIYNVAAMEIAQLIYAKVPNIREVYVRLLGQIGRPIDDPLIADVSVIPDKGALTSDSRQEIASLIDEYMSRLLTLSDRFLKGEVQLY

>Cren_Aeropyrum_pernix

MT---RRIVVESYPYPRVEDLQVELVERKGLGHPDTICDAAAEAVSRELSKHYLDRFGKILHHNVDKVLLVGGQAAPRLGGGEVLQPIYILVSGRVTTEVGGESVPVGPIILRAVKNYIRENFRFLDPEEHVIVDYRVGRGSVDLVGIFEAED--KVPLANDTSIGSGYAPLSTLERLVLETERTLNSRETKERLPAVGEDVKVMGVRDGKSITLTVAMAVVSSQVGSVSDYLAVKEEAESLILDLASRIAPDYDVRVNINTGDIPEKKILYLTVTGTSAEHGDDGATGRGNRVNGLITPMRPMSMEAAAGKNPVNHVGKIYNVVANEMAALIHREVKGVEEVYVKLVSQIGKPIDRPLIVDVKVRMEGGRVTADAKREIEAIANSVLDGITGYTEKLVRGDITVY

>Cren_Desulfurococcus_fermentans

MLKPVRNIEIELLRMTSVSRMKVELVERKGVGHPDYIADSASEAASRALSKYYLKEFGTILHHNLDKTLLVGGQSNPRFGGGTLLEPIYIVVAGRATTEVDGVKVPFGKIIIDAVKDWIKRNMRYLDPERHVIVDYMVRKGSTDLVTVFDAGR-SSVPLANDTSIGVGYAPLSTLEKLVLQSERLLNSPEFKSRVPAVGEDVKVMGLRRNKDIVLTIAAAIVDSEVRDKYEYLKVKEDIVNVVSDLAGRIAPDYNIKIHVNTADIPEKNSFYLTVTGTSAEHGDDGATGRGNRANGLIPPMRPISLEATAGKNPVNHVGKIYNVVARRIAERVYEVYNGFSDVYVELLSQIGKPIDQPLVASVKLIPSDNDIPSHIMSEIRGIADEELTNIKRVTEMVLNDQVTLY

>Cren_Thermosphaera_aggregans

ME---RNIEVELLKMQSVKAMKVELVERKGLGHPDYIADSASEVASRALSRYYLKHFGTILHHNLDKTLLVGGQSSPKFGGGELLEPIYIIVAGRATTEVDGEKIPFGKIIVEAVKDWIRNNFRFLDPERHVIVDYMVRKGSTDLVTVFEAGK-KFIPLANDTSIGVGYAPLTTLEKLVYETERLLNSQQFKNKYPAVGEDIKVMGLRRGKTIALTIAAAIIDSQVRDMGEYINTKDHVKEAVLDLASKIAPDYDTKVFVNTADLPDKGSVYLTVTGTSAEHGDDGATGRGNRANGLIPPMRPISLEATAGKNPVNHVGKIYNVVARRIAERVYDQFNYFSDVYVEILSQIGKPIDKPLVASIKLIPLDAEPPSHVLNEIRRIADEELENIVKVTELVLNDKVSLY

>Cren_Staphylothermus_hellenicus

MTK--RNIVVEALKYQSVRDMKVELVERKGVGHPDYIADSASEWASRALSKFYLKKFGTILHHNLDKTLLVGGQSHPRFGGGYLIEPIYIVVAGRATTEVDGVKVPFGALIVEAVREWIKKNMRYLDPDKHVIIDYKVRKGSADLVSVFEAGK-KTVPLANDTSIGVGYAPLSTLEKLVLETEKMLNSREYKSRNPAVGEDVKVMGLRQDRKIVLTIAAAIIDSLVRDLDEYLNIKEQIKEDVLNLASKIAGDYDVEIFINTADMPNKEVVYLTVTGTSAEHGDDGATGRGNRANGLIPPMRPISLEATAGKNPVNHVGKIYNIVAKRIAEKVYESIDGFKDVYVELLSQIGKPIDQPLVASIKIIPDNLELPGHVVEEIKAIADEELANITKITMDILEGKEILY

>Cren_Ignisphaera_aggregans

M----RNIIVNEYLWKGIENLNVEMVERKGLGHPDHIADAIAEHVSKELSKYYLKYFGVILHHNVDKVLVVGGQSKPQFGGGEVLHPIYIIVSGRATAYVEGEFVPIGRLVLKASKEWIKNNFRYLDPEQHVIIDYKIGQGSVDLVGIYELGV-KKIPLANDTSFGIGYAPLSTLENIVLQTEKLLNSTEIKQKIPAVGEDVKVMGLRIGKKIKLTIAIAMVSRHILNKEEYISVKEEVKDVILDRISKYSLDYDIDVNINVADKPEEGIFYLTVTGTSAEHGDDGATGRGNRVNGLITPMRPMSLEAAAGKNPVSHVGKIYNVVARIIAEKCYAEVKGIKEIYVGLLSQIGRPIDDPLIVDVKVVPEKA-LTLDMVNEIQSIADEVMSKITNITEDILEGKVSLY

>Cren_Ignicoccus_hospitalis

M----KNVTVTKLPLPPQDELDVEIVERKGTGHPDYIADAVSEEASRQLSLYYLKEFGHVFHHNLDKTLLVGGQASPRFGGGDVLQPIYIIISGRAVTEVDGVDVPLGTVLLKAAKDWLKKNLPHLDVEHHVVVDYKVGKGSADLVGVFELGK--TVPLANDTSVGVGFAPFSTLEKLVYTVERKLNSAETKAKIKALGEDIKVMGLRMKNKIKLTVAAAIVSEYVRDAGEYAAVKEEVKEIVEAVAHEVAPNHEVEVFINTADNPEKGIFYLTVTGTSAEHGDDGATGRGNRVNGLITPMRPMSLEAAAGKNPVNHVGKLYNVLANIAAQKVVEEVPETKEVYLEILSQIGRPITDPLIAAVRVK---GELTPTIEKRVKEIIEAELDRLPMITKEILEGKISVF

>Cren_Pyrolobus_fumarii

M----R-IVVEEVHWQPVEELSVELVERKGVGHPDYIADAIAEAVSRELSKFYLERYGYILHHNVDKVLVVGGQAEPRFGGGEVTQPIYILVSGRVTTEVDGERIPVGSIILRAAREWIRNSFRFLDPNEHVIIDYRVGRGSVDLVSVYEAGK--KVPLANDTSVGVGYAPLSTLERLVYETERMLNSREFKEKVPESGEDVKVMGLRQGKKIVLTVANAMISRLVKDKDHYASVKEEIKNAVEDLAAKLAPNYDVEVHVNTADMPERNIFYITVTGTSAEAGDDGATGRGNRVNGLITPMRPMSMEAAAGKNPVSHVGKIYNVMARIAAEKIHREVKGVREVYVQLLSQIGKPITEPLVANVKILPEE--LTSDMIREAEAIVGEVMGSYREITERIVRGEIELF

>Cren_Pyrodictium_delaneyi

MA---RNIVVESIRWQPIEEHGVELVERKGLGHPDTIADAAAEEASRILSQFYLERYGTVLHHNLDKVLVVGGQAAPKFGGGEVLQPIYIIVSGRATTEVDGEKVPIGPIIMKAVKGWIKEHFRFLDPENHVIVDYKIGHGSTDLVGIFDLGK-GQVPLANDTSVGVGFAPFSHLEQLVYETEKLLNSKEFKQKTPEVGEDIKVMGLRKGKKIELTIAAAIISSLVSDMDHYLSVKDAIREAVLDLASRIAPDYDVEVYVNTADKPDKGIVYLTVTGTSAEHGDDGMTGRGNRSYGLITPLRPMSLEAAAGKNPINHVGKIYNVMAMNIARRVYENVKGLKEVYVELLSQIGRPLNDPLVANVKVLTEKGELPSDAVREIEAIVEEELDNYHKITKLFIEGKISVF

>Cren_Hyperthermus_butylicus

MP---RNIVVESVKLQPVEEQSVELVERKGLGHPDTIADAAAEISSQYLSRYYIEKYGTILHHNLDKVLVVGGQAAPRFGGGEVLQPIYIIVSGRATTEVDGERIPIGTIILRAVKEWIREKFRFLDPESHIIVDYKVGQGSADLVGIYELGK-DSVPLANDTSVGVGFAPFSTLEQLVLETERLLNSKEFKEKNPEVGEDIKVMGLRRGRKIELTIAAAIISSLVQDLDHYLSVKEAIKEAVLDLASRIAPDYDVEVYVNTADKPDKGIVYITVTGTSAEHGDDGMTGRGNRSYGLITPLRPMSLEAAAGKNPVSHVGKIYNVMALNIARRIYDNVSGIREVYVKLLSQIGRPINDPLIANIKVVSEKGELPSNALREIEAIVEEELDKYQELTKLFVEGKITIF

>Eury_Halorubrum_lacusprofundi

MD---RNIQVSRLDRRAVEDQEVEIVERKGIGHPDSICDGIAESVSRALSQLYLDRVGKVLHYNTDETQLVAGRAAPAYGGGEVVEPLYVLIVGRATKEYEGEQLPVDSTALAAARDYLSEAIPELEYGTDVIIDVKLGEGSGDLQDVFGEET-QQVPMANDTSFGVGHAPLTETETIVYEAEHELNTT-YHADHPELGPDVKIMGKREGDRIDITVAAAMVDRYVDGLDEYDDAVENVREFVTELAESRTD-REVHVDVNTADDYDEGSVYLTVTGTSAEQGDDGSVGRGNRANGLITPNRPMSMEATSGKNPVNHIGKIYNLLSTRIAESVTEEVDGIRDLQVRLLSQIGRPIDEPHVADAQLVTEDGVALGDIEPKVLEIVDRELANVTDVTRSVIDGDVSTF

>Eury_Halogeometricum_borinquense

MTE--RNIKIEAVERLAVEDQSVEIVERKGIGHPDSIADGIAESVSRALSNLYLDRVGKVLHYNTDETQLVAGESAPAFGGGEVIEPMYILLVGRATKKYDGEKLPVDSVALEAARDYLRENIPELEFGTDVIVDVKLGEGSGDLQDVFGEDN-VEVPMSNDTSFGVGHAPLTETENIVLNVERQLNGP-YAERHPYLGPDVKVMGKREGDYIDITVAAAMVDAHIDDMADYKDAVEHVREYVSDVATDYTD-REVNVDVNTADNYDEGSIYLTVTGTSAEMGDDGSVGRGNRANGLITPNRPMSMEATSGKNPVNHIGKIYNLLSTDIAEAVVADVDGIRDLQVRLLSQIGRPIDEPHVADAKVITEDGVELSDIEADVQAIIDERLADVTNVTRRVIDGEMTTF

>Eury_Natrialba_aegyptia

MSE--RNIRVEPIDRQAVEDQEVEIVERKGIGHPDSICDGVAESVAGALAREYLDRVGEVLHFNTDETQLVAGEAAPAFGGGEVVDPIYLLIVGRATKHYEGQTIPTETIALRAAREYLEKTIPQLEYGEDIVVDVKLGEGSGDLQDVFGEDE-VRVPMANDTSFGVGHAPLTETERIVSEAESRLNGE-FAAENPYLGPDVKIMGKREGDTIDVTVAAAMVDEFIADLDAYADAVESVRAFVAEVAGDHTD-REVNVHVNTADDYEEGSIYLTVTGTSAEQGDDGSVGRGNRANGLITPNRSMSMEATSGKNPVNHIGKIYNLLSTQIAEEVVAEVDGIRDLRVRLLSQIGRPIDQPHVADVHVVTADGVALEDVEDEINAIVDTELANVTEITRKVIDGELTTF

>Eury_Natronobacterium_gregoryi

MTE--RNIRVEPIDRQAVEDQEVEIVERKGIGHPDSICDGIAESVAGALAREYLDRVGEVLHFNTDETQLVAGEAVPAFGGGEVVDPIYLLIVGRATKHYDGQVIPTERIALTAAREYLEETIPQLEVGEDIVVDVRLGEGSGDLQDVFGEDE-VSVPMANDTSFGVGHAPLTETERIVSEAERRLNGE-FVDENPYLGPDVKIMGKREGDEIDVTVAAAMIDEYIPTMDAYRDAVESVREFVEAVASEHTD-RDVTVYVNTADDYDEGSIYLTVTGTSAEQGDDGSVGRGNRANGLITPNRSMSMEATSGKNPVNHIGKIYNLLSTEIGKSVVDEVDDIRDLRVRLLSQIGRPIDQPHVADVHVVTDDGVTIEDVEADVEAIVDRELADVTEITRRVIDGELSTF

>Eury_Halobacterium_salinarum

MTD--RNIQVQSLDRSAVEDDAVEIVERKGLGHPDSICDGIAEHVCETLAREYRDRVGHVLHFNTDETQLVAGDAAPAFGGGNVIDPIYILVVGRATSHYDGHHIPVESIALEAAREYLRETLPHLDLETDVIVDVKLGEGSGDLQDVFTDDDGPAVPMANDTSFGVGHAPLTETERIVLEAERSLNGP-YAEHTPAVGEDVKVMGKREDDHIDLTIAAALVDAHVPDMDAYIAQVEAIREHVFDLATEHTD-REVTVHVNTADDYESGSIYLTTTGTSAEQGDDGSVGRGNRANGLITPNRAMSMEATSGKNPVNHIGKIYNLLSTQIAEAVVAEVDGIRDLRVRLLSQIGRPIDEPHVADVEVVTEDGTAVTDVDAEIERIVDAQLASVTDLTRRVIDGERTTF

>Eury_Halorhabdus_tiamatea

MSE--RNIRITQASGQAVEDQRVEVVERKGIGHPDSICDGLAEAVSQALAQAYLDRVGKVLHYNTDETQLVAGTSAPAFGGGEVIEPIYLLLVGRATKEYQGQRIPTDTIALEAAREYLDETFPHLDVGSDVIVDVRLGEGSGDLQEVFGEDG--AVPMANDTSYGVGHAPLTETEQIVLNTERRINGD-YTDEHPEIGQDIKVMGKREGDAIDVTVAAATVDAHVNDIHEYRDAVEGVREYVADLATEYTD-RDVRVQVNTADDYEEGSIYLTTTGTSAEQGDDGSVGRGNRANGLITPNRPMSMEATSGKNPVNHIGKIYNLLSTEIAKSVVSEVDGIRQLQVRLLSQIGSPIDDPHVADATIVTEGGLAITDVEDEVEAIIDDELEDITGVTERVIEGDLSTF

>Eury_Haloarcula_marismortui

MTE--RNIHVQPASGLAVEDQDIEVVERKGIGHPDTICDGIAETVSRALAQTYIDRFGTVLHYNTDETQLVAGTAAPAYGGGEVLEPIYILVVGRATKKFDGERIPAESIALRAARDYLDEQFPHLDLGSDVIVDVQFGEGSGDLQTVFGEEA--AIPMANDTSYGVGHAPLTETEQIVRNTEQKLTGE-YAESNPVVGQDVKVMGKREGDHIDVTVAVAMVDEHVPDLDAYKTAVSDVRAFVTDLAEEYTD-RDVTVHVNTADDYDAESIYLTTTGTSAEQGDDGSVGRGNRANGLITPNRPMSMEATSGKNPVNHIGKIYNLLSTEIAQSVANEVDGIRQVQMRLLSQIGSPIDEPHVADATVVTEDGVAVGDVEADIQATIDDELADVTDITRQVIEGDLSTF

>Eury_Natronomonas_pharaonis

MSD--RNIRVEPVVGRAVEEQDVEIVERKGLGHPDSLCDGIAEHVSQALARAYIDRVGKVLHYNTDETQLVAGTAAPAFGGGEVVDPIYLLITGRATKEYEGTKIPAETIALRAAREYINETLPFLEFGTDVVVDVKLGEGSGDLQEVFGEDG-KQVPMSNDTSFGVGHAPLTETERIVLEAERALNGD-YSDDNPAVGQDIKVMGKREGDDIDVTVAVAMVDRYVDDLDGYEAAVAGVREFVADLATDYTD-RNVSVHVNTADDYDEGAIYLTTTGTSAEQGDDGSVGRGNRSNGLITPNRSMSMEATSGKNPVNHIGKIYNLLSTEIARTVVDEVDGIREIRIRLLSQIGQPIDKPHVADANLVTEDGIEIADIEDEVEAIIDAELENVTSITERVIDGELTTF

>Eury_Methermicoccus_shengliensis

M----KNIEVEAIHQVPMEEQKIEIVERKGIGHPDSIADGIAEAMSRALCKEYKKRFGVILHHNTDETQIVAGRSLPAFGGGEVISPIYILLVGRATNEFDGHAIPTNRVVVRAARDYLHENFPNL-LDEYVIIDCRLGVGSSDLRDVFNRKH--NAPLANDTSFGVAHAPLSETESLVYHTERFLIEK-LRKRLPAIGEDIKVMGLRREDRISLTVACAMIDKYVEDIDHYVQIKEQIRDEIEGIVPMYTQ-REVDVFVNTADDIDKGSCYLTVTGTSAEMGDDGSVGRGNRCNGLITPFRPMSMEATSGKNPLNHVGKLYNLLSREIANEIAAEVEGVKEVYVRVLSQIGKPIDEPLLASVQFIPDGKQHTERIRRQSEEIVDRWLDNIPQITDMVADGKLSTF

>Eury_Methanosarcina_mazei

MA---RNIKVEELLQTPIEKQQIELVERKGIGHPDSISDGLAEAVSRALCREYITKCGAVLHHNTDETQIVAGRSSPKFGGGEVLQPIYMLLVGRATKEFEGAELATESVALKAARNYLRNTMVNMDLERDVIIDCKLGTGSSDLRDVFKRDR---VPMANDTSFGVGHAPFSELENIVYNTERQLLTD-LKSRMPAIGEDMKIMGLRDGDDISLTICSGMIGRYVDDLDSYINMTQEMKTYTEELAARYTE-RNVNVFVNTADNLKASCVFLTVTGTSAEMGDDGSVGRGNRCNGLITPNRPMSMEATSGKNPINHIGKIYNLLSTQMARDIVKQVPDVQDVYIRLLSQIGKPIDQPLVASAQIIPKEGTSFANVKSEAEVVIDDWLSNVTKITEMVIRGELNTF

>Eury_Methanolobus_profundi

MI---RNIKVEHLIETPVEKQQIELVERKGVGHPDSISDGLAEAVSRALCKEYIEKCGVVLHHNTDETQIVAGRSNPQFGGGEVTQPIYTLLVGRATKEFEGVEIPAESVALRAARDYIRNTIVDIDLESDIIVDCKLGTGSSDLRDVFNRDR---VPVANDTSFGVGHAPFSELEQIVYESERMLITD-LKKKIPGIGTDIKVMGLRDNDDINLTICCGMVGRHVDDMDHYINIKEEMADYVTDLALKRTD-RKVNTFINAADNEKCDSVFLTVTGTSAEMGDDGSVGRGNRCNGLITPNRPMSMEATSGKNPINHIGKIYNLLSTQMAKDIVKAVPEVEDVYIKLLSQIGQPIDQPLVASAQIIPADGANFASIRAETEVVIDDWLADITKITQMVISGELDTF

>Eury_Methanococcoides_burtonii

MI---RNIKVEHLHETPIEKQETELVERKGVGHPDSISDGLAEAVSRALCKEYIDKCGAILHHNTDETQIVAGRSRPEFGGGEVLKPIYTLLVGRATMEFDGMEIPAETVALQAAREYVRNTIPAMDLERDMIIDCKLGTGSSDLRDVFTRDH---VPMANDTSFGVGHAPFSELEQVVYNTERQLLTD-LKKKIPGIGEDIKVMGLRENNDISLTICCGMVGRHIDDMDHYINAKEEMTEYVLDLATKYTD-RTVSARINAADKVDGGCVFLTVTGTSAEMGDDGSVGRGNRSNGLITPSRPMSMEATSGKNPINHIGKIYNLLSTQMARDVVSAVDEVSDVHIKLLSQIGMPIDQPLVASAQVIPEDGANFAHIQSEAVVVIDDWLENITKITDMVVKGELDTF

>Eury_Methanosalsum_zhilinae

MM---RNIKVEQLMQTPVEDQKVEIVERKGVGHPDSIADGLAEAVSRALCREYIKKCGTVLHHNTDETQIVAGKSHPEYGGGEVIKPIYTLLVGRATTEFDGEEFAADAIAIEAARQYIRENFANINLERDMIIDCRFGTGSSDLRDVFSRNK---MPVANDTSFGIGHAPFSELESIAYNTERMLNTD-LKKKIPGIGEDIKVMGLREQDDISLTIGCATIGRYVDDLDHYINIKEEVEDYVAGLAAEHTE-RNVNIYVNTADQIEKESVFLTVTGTSAEMGDDGSVGRGNRCNGLITPGRPMSMEATSGKNPINHIGKIYNLLATQVAQDISKTIPEVDEAYVRLLSQIGKPIDQPFIASVQIIPEEGARLDSILSEAEGITDDWLASADRVIEMLINGKINTF

>Eury_Methanocorpusculum_labreanum

MK---RNISITHLSQTPMEKQKVELVERKCIGHPDSIADGVAEAISRALCREYMEECGAVLHHNTDQGEVVAGESLPQFGGGKIIKPIYFLLTGRATRQFGNKVFATDAIAIEAARAYLSETIPTFNMDTDVIVDCRMGTGSTDLRDVFHTKKHTAVPRANDTSFGVGHAPFSQVEQIILGLDEYISKE-FRPKNPMVGYDLKLMGLRDINTITITVASAMVDRYCSGIDEYVEMKEKMVESFTQVARQYTD-RKVKVEINTADIIRKKSVFLTVNGTSAEMGDDGSVGRGNRCNGLITPNRPMSMEATSGKNPINHIGKIYNLLSTEIAKEACQKVDGIEEMYVRLLSQIGHPIDYPHIASVQCITKRGYNFKDFAPEVEEIVNKRLENITDITRLVIDGKLKTF

>Eury_Methanosphaerula_palustris

MSS--RNIRVEALDQIPLEQQQIELVERKGIGHPDSLADGIAESVSQALCKAYMEECGAYLHHNSDQGEIVAGESQPKYGGGSIIKPIYVLLDGRATKEFEGITFPTDAIAIEAARKYLGSTLGELNLERDLIVDCRLGVGSTDLRDVFRPCD-SKLPRANDTSFGVGHAAFSETENIVKGVSDYIDGD-LRKKYPMIGRDVKVMGLRQKDEITLTIGCAMVDRYCAGLSEYIEMKEILNEAVLKVAKTYTS-RSIKIQINTADDIERGSVYLTVTGTSAEMGDDGSVGRGNRCNGLITPNRPMSMEATSGKNPINHIGKIYNLLSTQIARECVKKVDGIQEIYIRLLSQIGMPIDHPLVASAQVLPACGADFTSISRDIEGIIDEQLADVTCVTEKVIRGELNTF

>Eury_Methanospirillum_hungatei

MS---RNITIEMLNQIPVQDQQIELVERKCLGHPDSIADGIAEAVSRALCNTYIDQFGGVLHHNTDQGEIVAGESMPKFGGGKIIKPIFVLLDGRATKEFNGEKIAADTVALKAAKDYLRTILPELNLDQHLIMDCRLGTGSTDLRDVFKPEA-GQIPRANDTSFGVSYAPFSDIEKCIREISSYIDTT-LRPKYPVYGTDIKIMGLRQGNTIKLTICCAMVDRYVSSLSDYVNYREKLAEEALKVAKTCTD-KKVEVSVNTADCDIECSLFLTVTGTSAEMGDDGSVGRGNRANGLITPHRPMSMEATSGKNPINHIGKIYNLLSNELAHTCVEKVDGIAEIQVRLLSQIGSPIDQPLVASAQIIPKPSFTVKDIERDVYEIIDSGLENITSVTERVIRGELKTF

>Eury_Methanoregula_boonei

MK---RNIQIEALNQIPLEKQRIELVERKCLGHPDSIADGIAESISQALCREYLKEFGAVLHHNTDQGEVVAGESCPKFGGGKMIRPIYVLIDGRATKQFNGVTIPTDTVAVEAAHEYLHKILPELNLQRDVMIDSRLGTGSTDLRDVFKPRQ-GKVPRSNDTSFGVGHAPFSDVETIIRNTSEYIDTK-LRKKYPAIGQDIKIMGLRDGNTITLTVACAIVDRYCADIREYQEYMGLLTEEIGKVAKKSTK-RKVVVNLNTADDIKSKSVFLTVTGTSAEMGDDGSVGRGNRCNGLITPNRPMSMEATSGKNPINHIGKIYNLLSTQIAQESIKKVDGIEEMYVRLLSQIGKPIDQPLVASVQVLPRKGVTLQEINGEIQAIVDDNLANVTSITEKVIRGELKTF

>Eury_Methanobrevibacter_smithii

M----RNIIVKELNQTYIEDIDIEIVERKGIGHPDSISDGIGETVSEALCKMYMDELGGVLHHNTDEVQITAGESNPVFGGGKILKPIDILLTGRGVSEYDGVKFPLDRVAIEAAKNFLDETIINLDVELDTVVECKIGHGSGDLVDVFKREG---APSSNDTSFGVGYAPFSETENLVKATEELLNSKSFKAKHPAVGEDIKVMGLREGEKITLTIGCAMVSKFVANREEYIAVREELKDIVSDLATKYTN-REVEVFVNTADNTDESGYYLTVTGTSAEMGDDGSVGRGNRANGLITPCRPMSMEASSGKNPINHVGKIYNILSNEIANDVVENVEGIKQMNVMILSQIGKPIDQPKAASTQLILEDGVKLEDVDKKVEQIVDRWLEDISIITENVVQGKTRTF

>Eury_Methanothermobacter_marburgensis

M----RNIIVEPLNQTPIEDQKVEIVERKGIGHPDSISDGIAESVSRALCNAYLDRFGAIMHHNTDEVQITAGESAPQFGGGEVIKPIEILLTGRGIAEVDGEKIGLDRIAISAAKEYLRDNIINLDVETCTVVECKIGHGSGDLRDVFARKG--RAPLSNDTSFGVGFAPFSETERIVMEAENLLNSPEFKKKYPAVGEDIKVMGLRENDNITLTVACAMVDRYVSDLEEYLEIKNVVKDEVFKLASGITE-RNLEVFVNTADRDDEPSVYITVTGTSAEMGDDGSVGRGNRANGLITPNRPMSMEATSGKNPINHVGKIYNLLSNQMAADIVESIEGVKQVHIMILSQIGKPIDHPKAATAQVILEDGYTMDDITGKVSGVMDAWLEDIPSITEMLVKGQLRTF

>Eury_Methanosphaera_stadtmanae

M----RNIKIEKAVQRPIEQNEIEVVERKGIGHPDSISDGIAEAVSRVLSQTYKEKAGHVLHHNTDEVQITAGESDPKFGGGQIIKPIQILLTGRAANEFSGKKIGVDTIAIEAAKKFLQDTIINLDVEYGTVVECKIGQGSADLRDVFQRPN--TIPSSNDTSFGVGYAPFSQTENLVLKTEELLNSKDFKKQYPFVGEDIKVMGLREKEDITLTIAAAFVSKYVDDVDAYLNMKDELKNIVNDLAAKETD-LSVKTLINTADDKDESGYYLTVTGTSAEMGDDGSVGRGNRANGLITPNRPMSMEATSGKNPINHVGKIYNLLSNEITREVVSDVEGVKSIDMIILSQIGKPIDQPRTATAHIQTEEGYSINDVEEDVTKIINKWLENITDIKEFMLEGKLRTF

>Eury_Ferroplasma_acidarmanus

MESQLRNIQVEAIKQSPTMSREVEIVERKGIGHPDSVADGIAESVSRALSKYYLKQYGRILHHNTDQVEVVGGQADPKFKGGNVLDPTYILLSGRATASVGNERIPVKSLAIKSAKDYLREHFPDLDIDSDVMIDSRIGNGSVDLRGLYDTRK----FKANDTSFGVGFAPFTDTETLVKLTEKYINGD-LKKSLPAIGYDIKVMGFRKGRTINLTVAAAYVDKYVKDPSEYYSIKDELVSKIKDNAVKYTD-EDVQVFVNTGDVQSDSVYYLTVTGLSMENGDDGSVGRGNRVNGIITPYRPMSMEAAAGKNPVTHVGKLYNVLSNQIADKVVKEEGGIKEVLVRIVSQIGRPVDEPHVASLQLIYEDNVDPSKHKNNIRAIADDKLAHIDQLTQMFVDGKADVF

>Eury_Cuniculiplasma_divulgatum

MV---RNISVEAINQVPTHLRGVELVERKGLGHPDSVSDGIAESVSRELSKYYIKHFGKILHHNTDQVELVGGQSAPKFGGGVVLDPVYILLSGRATTTVDGERIPVKAVAIKAAKEYLQSNFKHLDMDSDIMIDSRIGHGSVDLRDVYDTTK----HRANDTSFGVGFAPFTDTEKLVKGTEKFINGE-MKKKLPAIGYDIKVMGYRQDKTINLTVAAAYVDKYVKDSNEYFAIKEQFQQLVEDNASKISN-EDVNIFINTADKDGDKSHYLTVTGLSQENGDDGSVGRGNRVNGIITPYKPMSMEAAAGKNPVTHVGKLYNVLSNKIAEHVVKEEGGVVEVLVRIVSQIGRSIDNPHVASLQIIYGDNVDPSKHKKNIEAITDHELEHIFDLTQGFVEGKIPVF

>Eury_Thermoplasma_acidophilum

ME---RNISVEELHQIPTPKKEVEIVERKGIGHPDSVADGIAEAVSRSLSKYYIEHYGRILHHNTDQVEVVGGQSAPKYGGGLVLEPTYILLSGRATTKVGNDRVPFKSITIKAARDYLREHFRHLDVDADVMIDSRIGQGSVDLVEVYDTKK----LEANDTSFGVGFAPLSETETMVLNTEKYLNGE-LKKKMPMVGYDIKVMGFRQKDTINLTVAAAMVDKYIHDADEYFNIKDELKQLVLDNAVEYTD-KEVKVYINTADIKEDGVGYLTVTGMSMENGDDGSVGRGNRVNGLITPYRAMSMEAAAGKNPVTHVGKLYNVLSNKIANDIVKEEGNIAEVLVRIVSQIGRPIDDPHVASVQVIYEGNVDHSKHKNNIRNLVDDRLAHISDLTMEFVEGKIPVF

>Eury_Aciduliprofundum_boonei

MT---RNIVVEDIIATPVEERDVEIVERKGIGHPDSVADGIAESVSRALSKYYLEHYGRILHHNTDQMEIVGGQAKPKFGGGQVLEPVYILLSGRATTTVNGERIPYRTIAKRAAADFLKKHYRHLDIEEDVIIDCMIGQGSVDLRGLYDTQK----YLANDTSFGVGYAPMSDLEKVVYETERYINGP-LKDKLPEIGEDIKVMGFRNKDKINITVAAAFVGKHTPDKDHYLNVKDELRDLLTDYAAKFTD-KEVNFFINTGDLVNEGVFYLTVTGLSMENGDDGSVGRGNRVNGLITPYRPMSMEASAGKNPVTHVGKLYNILAFKIAEDVAKAAEGVKEVHVRIVSQIGKPVDEPQVASIQVIPAEGVNIARYNEEFRNIADEWLANIHKITEMLVNGEVNVF

>Eury_Methanocaldococcus_jannaschii

M----RNIIVKKLDVEPIEERPTEIVERKGLGHPDSICDGIAESVSRALCKMYMEKFGTILHHNTDQVELVGGHAYPKFGGGVMVSPIYILLSGRATMEINEIKLPVGTTAVKAAKEYLKKVLRNVDVDKDVIIDCRIGQGSMDLVDVFERQK-NEVPLANDTSFGVGYAPLSTTERLVLETERFLNSDELKNEIPAVGEDIKVMGLREGKKITLTIAMAVVDRYVKNIEEYKEVIEKVRKKVEDLAKKIADGYEVEIHINTADDYERESVYLTVTGTSAEMGDDGSVGRGNRVNGLITPFRPMSMEAASGKNPVNHVGKIYNILANLIANDIA-KLEGVKECYVRILSQIGKPINEPKALDIEIITEDSYDIKDIEPKAKEIANKWLDNIMEVQKMIVEGKVTTF

>Eury_Methanococcus_maripaludis

MA----NIVVKRLERTPIDETPVEIVERKGIGHPDSICDGVAESVSVALCKMYKEKMGVVLHHNTDQVELVGGYAYPELGGGCMVSPIYILLSGRATMEVGKIKLPVNTTAVNAARDYLKKALRNMDLEKDVVVDCRIGQGSVDLVEVFDRKR-SEIPHANDTSFGVGHAPLSTTEKIVLETEKLLNSDALKAEIPAVGEDIKVMGLREGKKITLTIAMAAVDKYVNSCADYVKVKELAKAKVEENAKKYLDGHELEVCINTADD-DEDCIFLTVTGTSAEMGDDGSVGRGNRANGLITPFRPMSMEATSGKNPINHIGKIYNILSNIIAEDVA-KIEGVRECQIRILSQIGKPITEPKILDIEMIPENGFELEDLSPKAKEIAQKWLDNITEVTERIVSGNVTTF

>Eury_Methanothermococcus_okinawensis

MA----NIVVKKLDTTPIEERPTEIVERKGLGHPDSICDGIAESVSAALCKMYKEKMGTILHHNTDQVELVGGHAYPKLGGGNMVSPIYILLSGRATMEINEIKLPVGTVAVNAARNYLKKVLRNADLEKDVVVDCRIGQGSVDLMDVFDRKK-TDIPLANDTSFGVGHAPFSVTEKLVFETEKLLNSDELKKELPAVGEDIKVMGLREGKKITLTIAMAVVDRYVNSVEEYNEVKEKAKAKVEELAKKLADGYEVEVCINTADD--DNCIFLTVIGTSAEMGDDGSVGRGNRANGLITPFRPMSMEATSGKNPINHIGKIYNILANIIAHDVA-ELDGVKECYVRILSQIGKPINEPKILDIEVITEDNCDLKDIEPKAKEIAERWLDNIPEVRNKIINGEIKTF

>Eury_Ferroglobus_placidus

M----RNIVVEELVHTPIEEQKIEIVERKGIGHPDSLADGIAEAMSRALCKEYIKKVGAVLHHNTDETQIVAGKAKPSFGGGELIQPIYILLVGRATKYFDGIDIPADRIALKAAKNYVREAMRYLDPETDIIFDVRLGEGSTDLRDVFERQK-GKIPLANDTSFGIGYAPLSETENLVYNVERRIYEE-FRKKEKAMGEDVKVMALREKDTIKLTVAAATVDRHLSNIQEYLAVKEELSNFIKDIANEYTE-RKVEIFVNTADDVDRGVVYLTVTGTSAESGDDGSVGRGNRCNGLITPGRPMSMEATSGKNPINHVGKIYNILANLIAKDCYEAVEGIKEVHVRILSQIGKPIDQPKVCSIQVIPERGYSVEKMESKIKTVAEEWLAEITKVTEMVINGEIRTF

>Eury_Archaeoglobus_fulgidus

MP----NIFVEELVHTPIEKQVIEIVERKGIGHPDSLADGMAEAMSRELSREYIRRFGAVLHHNTDETQIVAGRSNPQFGGGEVIEPIYVLLVGRATKFFNGEYIPTDKIALKAARDYIRQHMQNLDPELDVVFNVRLGEGSTDLQDVFRRKS-GNVALANDTSFGIGFAPLSETERLVFNVERRIYEE-FRKKNPAIGEDVKVMGLREKDRISLTIAAAFVDRYVANIKEYDAIKEELENFVKEISSEYTE-REVEVFVNTADDYETGCVYLTVTGTSAENGDDGSVGRGNRCNGLITPGRPMSMEASSGKNPINHVGKIYNLLANQIAARIAEEVEGVEEVYVRILSQIGKPINEPKALSVQVIPKSGYDISKLERPARDIAEEMIANVGKITDMVIEGKVRTF

>Eury_Pyrococcus_furiosus

MA---RNIVVEEIVRTPVEMQQVELVERKGIGHPDSIADGIAEAVSRALCREYIRRYGVILHHNTDQVEVVGGRAYPRFGGGEVVKPIYILLSGRAVELVDQELFPVHEVAIKAAKNYLKNAIRHLDVENHVIIDSRIGQGSVDLVSVFNKARENPIPLANDTSFGVGYAPLSETERLVLETEKLLNSEKFKKEYPAVGEDIKVMGLRRGNEIDLTIAAAIVDSEVATPKEYLEVKDKIKEAVEELAKEITS-RKVNIYVNTADDPERGIYYITVTGTSAEAGDDGSVGRGNRVNGLITPNRHMSMEAAAGKNPVSHVGKIYNILAMLIAEDIAKTLP-VEEVYVRILSQIGKPIDQPLVASIQVIPKPGHSVKEFEKDAYSIADEWLANITKVQKMILEDKISVF

>Eury_Thermococcus_kodakarensis

MAGKVRNIVVEELVRTPVEMQKVELVERKGIGHPDSIADGIAEAVSRALSREYVKRYGIILHHNTDQVEVVGGRAYPQFGGGEVIKPIYILLSGRAVEMVDREFFPVHEIALKAAKDYLRKAVRHLDLEHHVIIDSRIGQGSVDLVGVFNKAKKNPIPLANDTSFGVGYAPLSETEKIVLETEKYLNSDEFKKKYPAVGEDIKVMGLRKGDEIDLTIAAAIVDSEVDNPDDYMAVKEAIYEAAKGIVESHTE-RPTNIYVNTADDPKEGIYYITVTGTSAEAGDDGSVGRGNRVNGLITPNRHMSMEAAAGKNPVSHVGKIYNILSMLIANDIAEQVEGVEEVYVRILSQIGKPIDEPLVASVQIIPKKGYSIDVLQKPAYEIADEWLANITKIQKMILEDKVNVF
